# Universal annotation of the human genome through integration of over a thousand epigenomic datasets

**DOI:** 10.1186/s13059-021-02572-z

**Published:** 2022-01-06

**Authors:** Ha Vu, Jason Ernst

**Affiliations:** 1 Bioinformatics Interdepartmental Program, University of California, Los Angeles, CA 90095 USA; 2 Department of Biological Chemistry, University of California, Los Angeles, CA 90095 USA; 3 Eli and Edythe Broad Center of Regenerative Medicine and Stem Cell Research at University of California, Los Angeles, CA 90095 USA; 4 Computer Science Department, University of California, Los Angeles, CA 90095 USA; 5 Jonsson Comprehensive Cancer Center, University of California, Los Angeles, CA 90095 USA; 6 Molecular Biology Institute, University of California, Los Angeles, CA 90095 USA; 7 Department of Computational Medicine, University of California, Los Angeles, CA 90095 USA

## Abstract

**Background:**

Genome-wide maps of chromatin marks such as histone modifications and open chromatin sites provide valuable information for annotating the non-coding genome, including identifying regulatory elements. Computational approaches such as ChromHMM have been applied to discover and annotate chromatin states defined by combinatorial and spatial patterns of chromatin marks within the same cell type. An alternative “stacked modeling” approach was previously suggested, where chromatin states are defined jointly from datasets of multiple cell types to produce a single universal genome annotation based on all datasets. Despite its potential benefits for applications that are not specific to one cell type, such an approach was previously applied only for small-scale specialized purposes. Large-scale applications of stacked modeling have previously posed scalability challenges.

**Results:**

Using a version of ChromHMM enhanced for large-scale applications, we apply the stacked modeling approach to produce a universal chromatin state annotation of the human genome using over 1000 datasets from more than 100 cell types, with the learned model denoted as the full-stack model. The full-stack model states show distinct enrichments for external genomic annotations, which we use in characterizing each state. Compared to per-cell-type annotations, the full-stack annotations directly differentiate constitutive from cell type-specific activity and is more predictive of locations of external genomic annotations.

**Conclusions:**

The full-stack ChromHMM model provides a universal chromatin state annotation of the genome and a unified global view of over 1000 datasets. We expect this to be a useful resource that complements existing per-cell-type annotations for studying the non-coding human genome.

**Supplementary Information:**

The online version contains supplementary material available at 10.1186/s13059-021-02572-z.

## Background

Genome-wide maps of histone modifications, histone variants, and open chromatin provide valuable information for annotating the non-coding genome features, including various types of regulatory elements [1–5]. These maps—produced by assays such as chromatin immunoprecipitation followed by high-throughput sequencing to map histone modifications or DNase-seq to map open chromatin—can facilitate our understanding of regulatory elements and genetic variants that are associated with disease [6–12]. Efforts by large-scale consortia as well as many individual labs have resulted in these maps for many different human cell and tissue types for multiple different chromatin marks [1, 8, 13–20].

The availability of maps for multiple different chromatin marks in the same cell type motivated the development of methods such as ChromHMM and Segway that learn “chromatin states” based on the combinatorial and spatial patterns of marks in such data [21–23]. These methods then annotate genomes in a per-cell-type manner based on the learned chromatin states. They have been applied to annotate more than a hundred diverse cell and tissue types [3, 16, 24]. Previously, large collections of per-cell-type chromatin state annotations have been generated using either (1) independent models that learn a different set of states in each cell type or (2) a single model that is learned across all cell types, resulting in a common set of states across cell types, yet generating per-cell-type annotations (in some cases per-tissue-type annotations are generated, but we will use the terms cell-type and tissue interchangeably for ease of presentation). This latter approach is referred to as a “concatenated” approach (Additional File 1: Fig. S1) [22, 25]. Variants of the concatenated approach attempt to use information from related cell types to reduce the effect of noise, but still output per-cell-type annotations [26, 27]. These models that produce per-cell-type annotations tend to be most appropriate in studies where researchers are interested in studying individual cell types.

A complementary approach to applying ChromHMM to data across multiple different cell types referred to as the “stacked” modeling approach was also previously suggested (Additional File 1: Fig. S1) [22, 25]. Instead of learning per-cell-type annotations based on a limited number of datasets available in each cell type, the stacked modeling approach can learn a single universal genome annotation based on the combinatorial and spatial patterns in datasets from multiple marks across multiple cell types. This approach differs from the concatenated and independent modeling approaches as those approaches only identify combinatorial and spatial patterns present among datasets within one cell type.

Such a universal annotation from stacked modeling provides potential complementary benefits to existing concatenated and independent chromatin state annotations. First, since the model can learn patterns from signals from the same assay across cell types, a stacked model may help differentiate regions with constitutive chromatin activities from those with cell-type-specific activities. Previously, subsets of the genome assigned to individual chromatin states from “concatenated” annotations were post hoc clustered to analyze chromatin dynamics across cell and tissue types [3, 16]. However, such an approach does not provide a view of the dynamics of all the data at once, which the stacked modeling provides. Second, the stacked modeling approach bypasses the need to pick a specific cell or tissue type when analyzing a single partitioning and annotation of the genome. Focusing on a single cell or tissue type may not be desirable for many analyses involving other data that are not inherently cell-type-specific, such as those involving conserved DNA sequence or genetic variants. For example, when studying the relationship between chromatin states and evolutionarily conserved sequences, if one uses per-cell-type chromatin state annotations from one cell type, many bases will lack an informative chromatin state assignment (e.g., many bases are in a quiescent state), while subsets of those bases will have a more informative annotation in other cell types. Third, if one tries to analyze per-cell-type annotations across cell types, one would need a post hoc method to reason about an exponentially large number of possible combinations of chromatin states across cell types (if each of *K* cell types has *M* states, there are *M*^*K*^ possible combinations of states for a genomic position) many of which would likely lack biologically meaningful distinctions. In contrast, for the stacked model, there will be a single annotation per position out of a possibly much smaller fixed number of states (compared to *M*^*K*^). These states are directly informative of cross-cell type activity, though the state definitions can be more complex. Finally, annotations by the stacked modeling leverage a larger set of data for annotation, and thus have the potential to be able to identify genomic elements with greater sensitivity and specificity.

Despite the potential complementary advantages of the “stacked” modeling approach, it has only been applied on a limited scale to combine data from a small number of cell types for highly specialized purposes [28, 29]. No large-scale application of the stacked modeling approach to many diverse cell and tissue types has been previously demonstrated. This may have in part been due to large-scale applications of stacked modeling raising scalability challenges not present in modeling approaches for concatenated and independent annotations.

Here, we present a large-scale application of the stacked modeling approach with more than a thousand human epigenomic datasets as input, using a version of ChromHMM for which we enhanced the scalability. We conduct various enrichment analyses on the states resulting from the stacked modeling and give biological interpretations to them. We show that compared to the per-cell-type annotations from independent and concatenated models, the stacked model’s annotation shows greater correspondence to various external genomic annotations not used in the model learning. We analyze the states in terms of enrichment with different types of variation, and highlight specific states of the stacked model that are enriched with phenotypically associated genetic variants, cancer-associated somatic mutations, and structural variants. We expect the stacked model annotations and detailed characterization of the states that we provide will be a valuable resource for studying the epigenome and non-coding genome, complementing existing per-cell-type annotations.

## Results

### Annotating the human genome into universal chromatin states

**Fig. 1:**
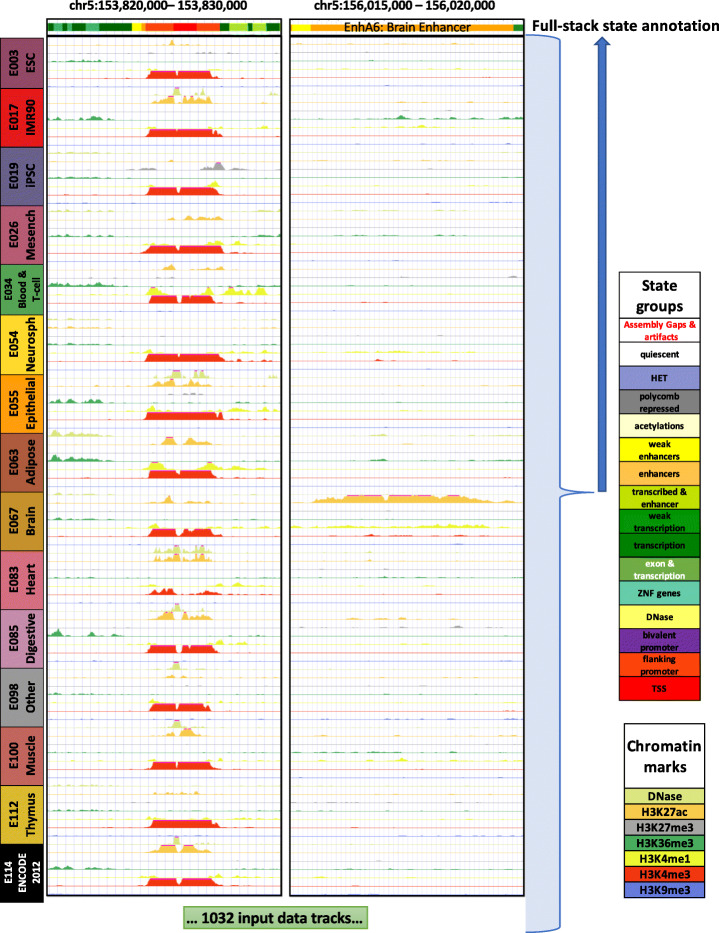
Illustration of full-stack modeling annotations. The figure illustrates the full-stack modeling at two loci. The top track shows chromatin state annotations from the full-stack modeling colored based on the legend at right. Below it are signal tracks for a subset of the 1032 input datasets. Data from seven (DNase I hypersensitivity, H3K27me3, H3K36me3, H3K4me1, H3K4me2, H3K4me3, and H3K9me3) of the 32 chromatin marks are shown, colored based on the legend at right. These data are from 15 of the 127 reference epigenomes each representing different cell and tissue groups. The loci on left highlight a genomic region for which a portion is annotated as constitutive promoter states (TSS1-2). The loci on the right panel highlight a region for which a portion is annotated as a brain enhancer state (EnhA6), which has high signals of H3K27ac in reference epigenomes of the group Brain. The concatenated model annotations for these loci from these and additional reference epigenomes can be found in Additional File 1: Fig. S24

We used the stacked modeling approach of ChromHMM to produce a universal chromatin state annotation of the human genome based on data from over 100 cell and tissue types from the Roadmap Epigenomics and ENCODE projects (Fig. 1) [14, 16]. In total, we applied ChromHMM to 1032 datasets for 30 histone modifications, a histone variant (H2A.Z), and DNase I hypersensitivity (Additional File 1: Fig. S2). The set of cell and tissue types were the same as those for which per-cell-type annotations were previously generated by applying the “concatenated” modeling approach of ChromHMM [16, 22, 25]. We note that not all chromatin marks were profiled in all cell or tissue types, but the stacked modelling can still be applied directly.

We focused our analysis on a model with 100 states (“Methods”). The number of states is larger than typically used for models that generate per-cell-type annotations, which reflects the greater information available when defining states based on data from many cell types. This number of states was large enough to be able to capture some relatively cell-type-specific regulatory activity, while being small enough to yield distinct biological interpretations for each state (Additional File 1: Fig. S3). We denote the model’s output chromatin state annotation the “full-stack” genome annotation.

### Major groups of full-stack states

**Fig. 2:**
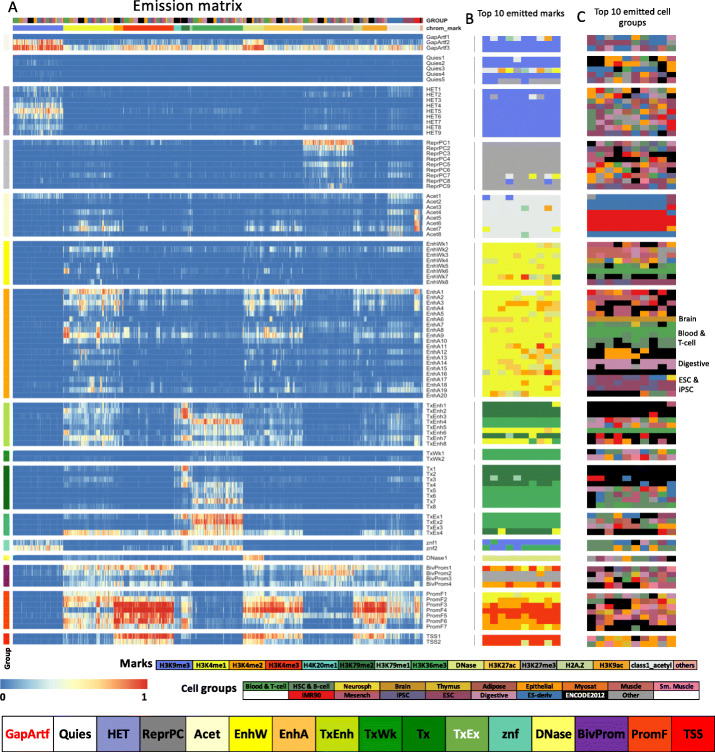
Full-stack state emission parameters. **A** Each of the 100 rows in the heatmap corresponds to a full-stack state. Each of the 1032 columns corresponds to one dataset. For each state and each dataset, the heatmap gives the probability within the state of observing a binary present call for the dataset’s signal. Above the heatmap, there are two rows, one indicating the cell or tissue type of the dataset and the other indicating the chromatin mark. The corresponding color legends are shown towards the bottom. The states are displayed in 16 groups with white space between each group. The states were grouped based on biological interpretations indicated by the color legend at the bottom, with group abbreviations defined in the main text. Full characterization of states is available in Additional Files 2-6. The model’s transition parameters between states can be found in Additional File 1: Fig. S6. Columns are ordered such that datasets profiling the same chromatin marks are next to each other. **B** Each row corresponds to a full-stack state as ordered in **A**. The columns correspond to the top 10 datasets with the highest emission value for each state, in order of decreasing ranks, colored by their associated chromatin marks as in **A**. **C** Similar to **B**, but datasets are colored by the associated cell or tissue type group. On right, the cell or tissue groups primarily associated with some of the enhancer states are noted

**Fig. 3:**
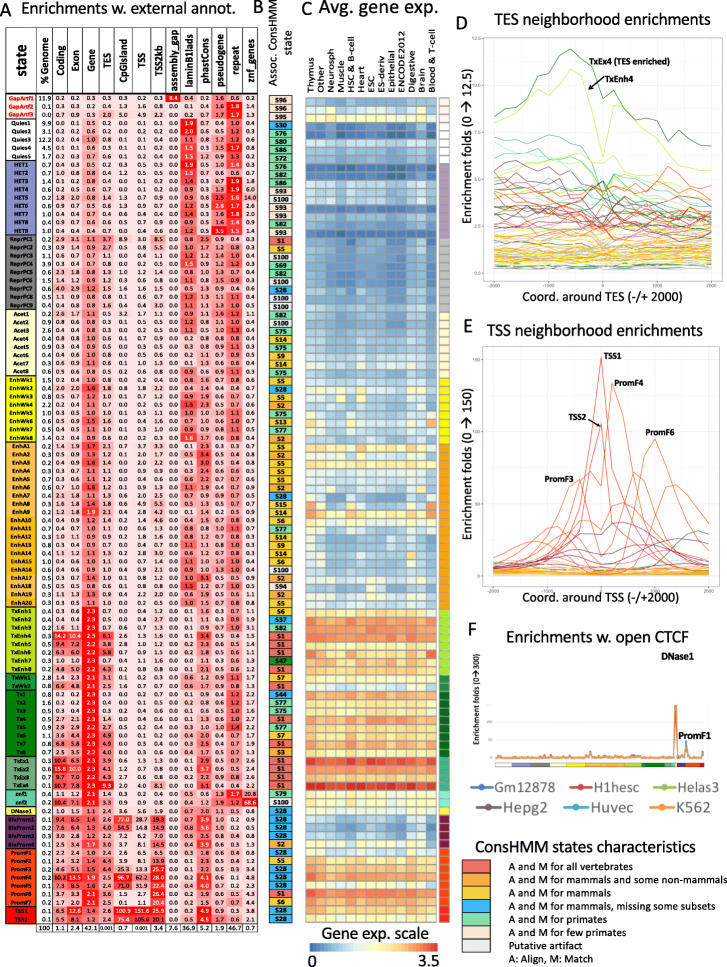
Full-stack states enrichments for external genomic annotations. **A** Fold enrichments of full-stack states with external genome annotations (“Methods”). Each row corresponds to a state and each column corresponds to one external genomic annotation: CpG Islands, Exons, coding sequences, gene bodies (exons and introns), transcription end sites (TES), transcription start sites (TSS), TSS and 2 kb surrounding regions, lamina-associated domains (laminB1lads), assembly gaps, annotated ZNF genes, pseudogenes, repeat elements, and PhastCons constrained element (“Methods”). The last row shows the percentage of the genome that each external genome annotation covers. The heatmap colors are column-normalized, i.e., within each column, the color of the cells are such that highest values are colored red and lowest values are colored white. **B** Each row indicates the ConsHMM state [30] that has the highest enrichment fold in each full-stack state as ordered in **A**. Legends of the ConsHMM state groups indicated with different colors are shown below the heatmap in **A**, and descriptions of select ConsHMM states curated from [30] are available in Additional File 7. **C** Average weighted expression of genes that overlap each full-stack state in different groups of cells (“Methods”). Each column corresponds to a cell group indicated at the bottom. Each row corresponds to a state, as ordered in **A. D,E** Positional enrichments of full-stack states relative to annotated **D** transcription end sites (TES) and **E** transcription start sites (TSS). Positive coordinate values represent the number of bases downstream in the 5′ to 3′ direction of transcription, while negative values represent the number of bases upstream. Each line shows the positional enrichments in a state. Lines are colored as indicated in **A. F** Enrichments of full-stacks states with concatenated chromatin states associated with CTCF and open chromatin, but limited histone modifications in six cell types [31] (“Methods”). The six cell types are indicated along the bottom of the figure. States are displayed horizontally in the same order as **A**. The DNase1 state showed the strongest enrichment for the concatenated chromatin states associated with CTCF and open chromatin in all six cell types

We characterized each state of the model by analyzing the model parameters (emission probabilities and transition probabilities) and state enrichments for other genome annotations (Figs. 2 and 3A, Additional File 1: Fig. S4-7). The other genomic annotations include previous concatenated chromatin state annotations (Additional File 1: Fig. S8-9), cell-type-specific gene expression data (Additional File 1: Fig. S10-11), and various independent existing genomic annotations (Fig. 3A). These independent genomic annotations included annotated gene features, evolutionary constrained elements, and assembly gaps, among others (“Methods”).

These analyses led us to group the 100 full-stack states into 16 groups (Fig. 2A). One group includes states associated with assembly gaps (GapArtf1) and alignment artifacts (GapArtf2-3). Some other groups are associated with repressive or inactive states, including quiescent states (Quies1-5) (low emissions for all datasets, except possibly weak signals in H3K9me3), heterochromatin states associated with H3K9me3 (HET1-9), and polycomb repressed states associated with H3K27me3 (ReprPC1-9). There is an acetylations group marked primarily by high emission of various acetylation marks profiled only in IMR90 or ESC and ESC-derived cells, while having weaker signals for enhancer- or promoter-associated marks such as H3K4me1/2/3, H3K27ac, and H3K9ac (Acet1-8). We also identified weak and active candidate enhancer groups (EnhW1-8 and EnhA1-20, respectively) associated with H3K4me1, DNase, H2A.Z, and/or H3K27ac. Four groups are associated with transcriptional activities, including a group of transcribed candidate enhancers (TxEnh1-8), two groups of weak or strong transcription (TxWk1-2, Tx1-8, respectively), and one group associated with exons and transcription (TxEx1-4). These transcriptional activities groups are associated with at least one of these marks: H3K36me3, H3K79me1, H3K79me2, or H4K20me1. Another group consists of two zinc finger (ZNF) gene states associated with H3K36me3 and H3K9me3 (znf1-2). A DNase group consists of one state (DNase1) with strong emission of *only* DNase I hypersensitivity in all profiled cell types. Three groups are associated with promoter activities, marked by emission of some promoter marks such as H3K4me3, H3K4me2, and H3K9ac. One promoter group was of bivalent states associated with promoter marks and H3K27me3 (BivProm1-4). The other two promoter groups were flanking promoter states (PromF1-7) and transcription start site (TSS) states (TSS1-2) where the flanking promoter states also show emission of H3K4me1.

Enrichments for external annotations supported these state groupings (Fig. 3A), as well as further distinctions or characterizations among states within each group. For example, the state GapArt1 had ~ 8 fold enrichment for assembly gaps and contained 99.99% of all assembly gaps in hg19 (Fig. 3A). In previous concatenated models based on the Roadmap Epigenomics data [16], no specific state was associated with assembly gaps, likely due to the limited number of input chromatin mark signals compared to the number of states, leading to assembly gaps being incorporated in the general quiescent state. The states in the zinc finger gene group, znf1-2, had 20.8 and 68.6 fold enrichment for ZNF named genes, respectively (Fig. 3A). States in the Acet group had a lower average expression of proximal genes compared to states in the enhancer and promoter groups (Fig. 3C) while higher compared to ReprPC and HET groups. States in the transcription groups (TxEnh1-8, TxWk1-2, Tx1-8, TxEx1-4) were all at least 2.1 fold enriched for annotated gene bodies; these gene bodies covered 88.8–97.5% of the states. These states are associated with higher expression of genes across different cell types, particularly when downstream of their TSS (Fig. 3A, C, Additional File 1: Fig. S10-11). Distinctions were seen among these states, for example, in terms of their positional enrichments relative to TES (Fig. 3A, D, Additional File 1: Fig. S12). States in the flanking promoter group (PromF1-7) showed 6.5–28-fold enrichment for being within 2 kb of annotated TSS, with distinctions among states in terms of their relative distance from the TSS (Fig. 3A, E, Additional File 1: Fig. S12A). Genes whose TSS regions overlapped these states had higher average gene expression across different cell types (Fig. 3A, C, Additional File 1: Fig. S10-11). These states differed among each other in their enrichments with upstream or downstream regions of the TSS (Fig. 3E, Additional File 1: Fig. S12). The states in the transcription start site group (TSS1-2) had enrichment values that peaked at the TSS (≥ 100 fold enrichment) (Fig. 3A, E). States in promoter-associated groups (TSS, PromF, BivProm), along with those in other groups, show various enriched Gene Ontology (GO) terms based on genes overlapping or proximal to each state (Additional File 1: Fig. S13, Additional File 2, Methods). For example, among biological process terms, BivProm1 is most enriched for “embryonic organ morphogenesis” genes, while TSS1 is most enriched for “nucleic acid metabolic process,” consistent with the bivalent [32] and the constitutively active nature of the two states, respectively. The DNase-specific state DNase1 showed distinct enrichment for CTCF-specific chromatin states defined in a concatenated model for six cell types, compared to other full-stack states [31] (Fig. 3F, Additional File 1: Fig. S14). These CTCF-specific states have previously been suggested to be candidate insulators and may have other roles that CTCF has been implicated in such as demarcating TAD boundaries [33–36]. The DNase1 state may correspond to similar roles, particularly where the CTCF-binding is relatively stable across cell types.

Compared to other full-stack state groups, those associated with promoters (TSS, flanking promoters, bivalent promoters) and the DNase group showed lower average DNA methylation levels across cell types (Additional File 1: Fig. S15). Among promoter-associated states, those showing stronger enrichments with CpG Islands also showed lower methylation levels (Fig. 3A, Additional File 1: Fig. S15), consistent with previous studies [37, 38]. Some promoter-associated states (TSS1-2, PromF3-5, BivProm1-2) are among the most enriched states at the center of binding regions of polycomb repressed complex 1 and 2 (PRC1 and PRC2) subunits. In addition, several ReprPC and BivProm states are among the most enriched states in windows surrounding binding regions of the EZH2 and SUZ12 subunits of PRC2, consistent with these states’ association with H3K27me3 (Additional File 1: Fig. S16-17). A detailed characterization of all states in terms of associated chromatin marks, genomic elements, and different associated per-cell-type chromatin states across cell groups can be found in Additional File 3-5. We expect it will serve as a resource for future applications using the full-stack annotations.

We verified that enrichments computed based on hg19 were highly similar to those computed for full-stack annotations mapped to hg38 (average correlation 0.99; Methods, Additional File 1: Fig. S18), ensuring the applicability of state annotations in hg38. We also confirmed that the full-stack annotations were generally more predictive of the positions overlapping a variety of external genome annotations considered in Fig. 3A than two sets of per-cell-type annotations, a previous 18-state per-cell-type chromatin state annotation based on concatenated models from 127 cell types [16], denoted the concatenated annotations, and 100-state per-cell-type annotations learned independently in each cell type, denoted the independent annotations (“Methods”). As expected, since the full-stack model uses more data representing more cell types, the full-stack annotations had greater predictive performance in most cases (Additional File 1: Fig. S19-22, Additional File 6). One of the exceptions to this was lamin B1-associated domains from Tig3 human lung fibroblasts [39], where six independent annotations were more predictive, three of which were annotations for fibroblasts. We note that these evaluations were done under the assumption that a chromatin state annotation that is more predictive of well-established external genomic elements will also be more informative of less well-established classes of elements. These results suggest that the full-stack annotations will, in most cases, have greater information than any single concatenated or independent annotation about localization patterns of some target genomic elements, with likely exceptions when the target of interest is specific to a certain cell type. In such cases, the corresponding cell type’s concatenated or independent chromatin state annotation may be more predictive.

### Stacked model differentiates cell-type-specific from constitutive activity

While the major groups of states outlined above can correspond to states from concatenated models [3, 16], the full-stack states provide additional information. For example, the states can differentiate cell-type-specific from constitutive activities. This cell-type specificity in the full-stack states is reflected in the emission parameters of cell types from different tissue groups (Fig. 2B,C, Additional File 3) and the overlap of concatenated chromatin state annotations from different cell types [40] (Additional File 1: Fig. S8-9, Additional File 5).

Consistent with previous findings that enhancers tend to be relatively cell-type-specific while promoters tend to be shared across cell types [3, 41], enhancer states exhibited clearer cell-type-specific associations than those of the promoter states (Fig. 2C, Additional Files 3 and 5). On average, states of active enhancer and weak enhancer groups (EnhA1-20, EnhW1-8) showed at least twofold higher coefficients of variations, in terms of emission probabilities for various marks, compared to states in the TSS, flanking and bivalent promoter groups (Additional File 1: Fig. S23). The enhancer states differed among each other in their associations with different cell/tissue types such as brain (EnhA6), blood (EnhA7-9 and EnhWk6), digestive tissue (EnhA14-15), and embryonic stem cells (EnhA18) (Figs. 1 and 2, Additional File 1: Fig. S24-25). These differences in cell-type-specific activities are also associated with different gene expression levels of overlapping genes with the states. For example, some blood enhancer states (EnhA8, EnhA9, EnhWk6) overlapped genes with higher average gene expression in cell types of the blood group, while some enhancer states specific to digestive group or liver tissues (EnhA14, EnhA15) showed higher gene expression in the corresponding cell or tissue types (Fig. 3C, Additional File 1: Fig. S10).

Other groups of states besides enhancers also had individual states with cell-type-specific differences. For example, four of the nine states in the heterochromatin group (HET1-2,4,9) showed higher emission probabilities of H3K9me3 in only subsets of cell type groups (states HET1-2 with IMR90 and epithelial cells; state HET4 with adipose, mesench, neurospheres, ESC, HSC&B cells). State HET9 showed strong association for heterochromatin in ESC/iPSC groups, while being mostly quiescent in other cell types based on concatenated annotations (Fig. 2C, Additional File 1: Fig. S26, Additional File 3 and 5). State PromF5 is associated with putative bivalent promoter chromatin states in some blood and ESC-related cell types, but with flanking promoter states in most other cell types (Additional File 1: Fig. S27, Additional File 3 and 5). In addition, some quiescent states (Quies1-2, Quies4-5) show weak signals of H3K9me3 in specific groups of cell types (Additional File 3). States in the polycomb repressed and bivalent promoter groups (ReprPC1-9, BivProm1-4) also showed differences in signals across cell groups, such as state ReprPC9, which showed H3K27me3 signals in only ESC/iPSC cell types (Additional File 3). The ability of the stacked modeling approach to explicitly annotate both cell-type-specific and constitutive patterns for diverse classes of chromatin states highlight a complementary advantage of this approach relative to approaches that provide per-cell-type annotations.

### Full-stack states show distinct enrichments for repeat elements

**Fig. 4:**
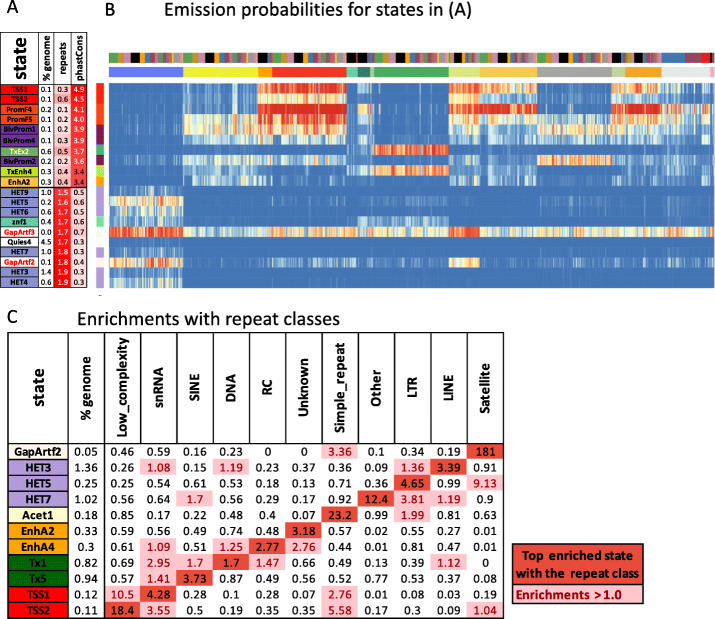
Full-stack states enrichments with conserved elements and repeat classes. **A** The first ten rows show the states most enriched with PhastCons elements and concurrently least enriched with RepeatMasker repeat elements, ordered by decreasing enrichments with PhastCons elements. The bottom ten rows show the states most enriched with repeat elements and concurrently least enriched with PhastCons elements, ordered by increasing enrichments with repeat elements. The columns from left to right list the state ID, the percent of the genome that each state covers, and the fold enrichments for repeat elements and PhastCons elements. **B** Heatmap of the state emission parameters from Fig. 2A for the subset of states highlighted in panel **A**. The colors are the same in Fig. 2A. **C** Fold enrichments of full-stack states with different repeat classes (“Methods”). Rows correspond to states and columns to different repeat classes. Only states that are most enriched with at least one repeat class are shown. Fold enrichment values that are maximal for a given repeat class are shown in dark red. Other fold enrichments greater than one are shaded light red

As the full-stack model showed greater predictive power for repeat elements than cell-type-specific models (Additional File 1: Fig. S19-21), we next analyzed which states contributed most to this power. The full-stack state enrichments for bases in repeat elements ranged from 10-fold depletion to 2-fold enrichment (Fig. 3A). The top ten states most enriched with repeat elements were chromatin states associated with H3K9me3 marks and in the heterochromatin, artifact, quiescent, or ZNF genes groups (Fig. 4A, B). Repeats being consistently enriched in H3K9me3-marked states is consistent with a natural mechanisms of cells to reduce the repeats’ risks to genome integrity, since H3K9me3 is characteristic of tightly packed DNA (heterochromatin) that is physically inaccessible [42].

We also observed that individual full-stack states had distinct enrichments for different repeat classes (Fig. 4C, Additional File 1: Fig. S28). For example, Acet1, a state associated with various acetylation marks and H3K9me3 had a 23-fold enrichment for simple repeats, largely driven by (CA)n and (TG)n repeats which were 72 and 76 fold enriched in this state and comprised 74% of all bases within this state that overlapped simple repeats (Additional File 1: Fig. S28). As (CA)n and (TG)n repeats are known to be highly polymorphic in humans [43], this suggests the possibility that signal detected in these regions may in part be due to technical issues related to deviations from the reference genome. The two artifact associated states in the GapArt group, GapArtf2-3, had a particularly high enrichment for satellite (181 and 145 fold, respectively) and rRNA repeat classes (75 and 580 fold, respectively) (Fig. 4C, Additional File 1: Fig. S28), likely associated with sequence mapping artifacts. States in the transcription start site group, TSS1-2, were most strongly enriched with low complexity repeat class (10.5-18 fold) and most notably GC-rich repeats (195–303 fold), consistent with these states being most enriched for windows of high GC content (Additional File 1: Fig. S28). Moreover, the TSS1-2 states are also most enriched with tRNA class (50-61 fold) (Additional File 1: Fig. S28), consistent with tRNAs being short genes [44].

**Fig. 5:**
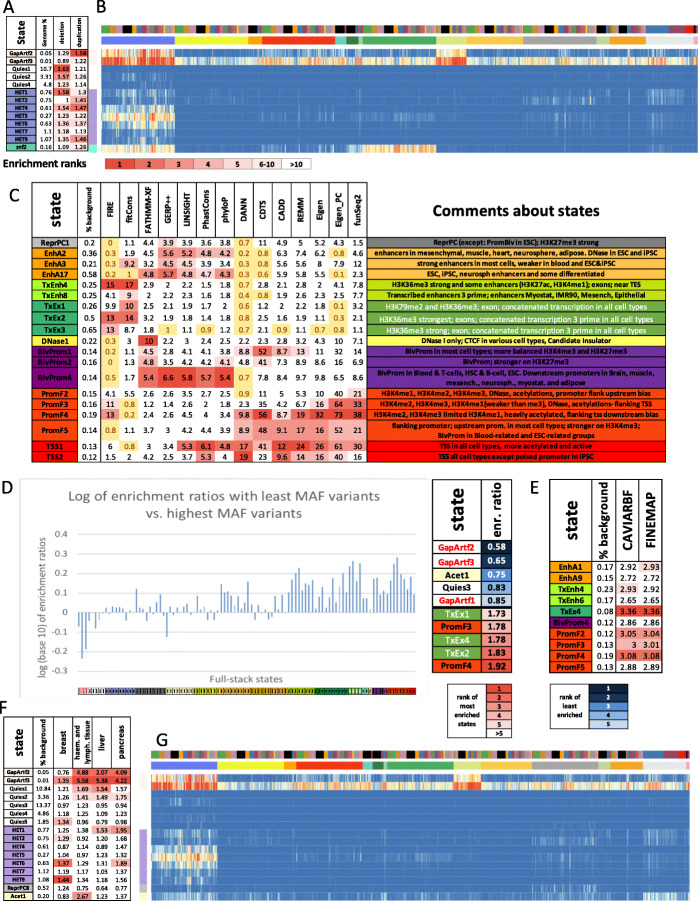
Full-stack states’ relationship with human genetic variants. **A** Enrichments of full-stack states with duplication and deletion structural variants from [45]. Only states that are in the top ten most enriched states for either duplications or deletions are shown. Top five fold enrichments for each class of structural variants are colored in increasing darker shades of red for higher ranked enrichments. . The columns from left to right are the state label, percent of genome the state covers, the fold enrichment for deletions, and fold enrichment for duplications. **B** Emission probabilities corresponding to states in **A.** The coloring is the same as Fig. 2A. The figure highlights how states most associated with structural variants generally had higher emission of H3K9me3 compared to other chromatin marks. **C** Enrichments of full-stack states with top 1% prioritized bases in the non-coding genome by 14 variant prioritization scores previously analyzed [30]. Only states that are among the top five most enriched states by at least one score are shown. The top five enrichment values for each score are colored in increasing darker shades of red for higher ranked enrichment values. Enrichment values below one, corresponding to depletions, are colored in yellow. The columns from left to right are the state label, percent of the genome covered, the 14 score enrichments, and a detailed description of the state. **D** Log base 10 of ratios of states’ enrichment with GNOMAD variants [46] with the lowest MAFs (< 0.0001) vs. GNOMAD variants with the highest MAFs (0.4–0.5). States are ordered as in Fig. 2A. Top five states with the highest and lowest enrichment ratios are labeled to the right. **E** States most enriched with fine-mapped phenotypic variants against the background of common variants. Fine-mapped phenotypic variants were identified by either CAVIARBF [47] or FINEMAP [48] (“Methods”). **F** State enrichments with somatic mutations associated with four cancer types in the non-coding genome. Only states that are among the ten most enriched with variants from at least one cancer type are shown. States in the top five are colored according to their ranks. The top five enrichment values for each cancer type are colored in increasing darker shades of red for higher ranked enrichment values. The columns are the state label, the percent of the genome the state covers, and the fold enrichments of variants from breast, haematopietic and lymphoid, liver, and pancreas cancer types. **G** Emission probabilities corresponding to states in **F**, as subsetted from Fig. 2A. The coloring is the same as Fig. 2A. The figure highlights how states with the greatest enrichments for cancer-associated variants tend to have higher emission probabilities for H3K9me3 compared to other chromatin marks

We also saw specific states associated with the largest repeat classes of the genome, SINEs, LINEs, and LTRs. SINE repeats were most enriched in state Tx5 (3.7 fold) (Fig. 5C), which had high emission of H3K36me3 (Fig. 2A, B, Additional File 1: Fig. S4-5), consistent with previous studies showing that SINEs are more enriched in gene-rich regions and in transcription-related states based on concatenated annotations [21, 49, 50]. In contrast, LINEs are depleted in most transcription-related states, reflecting the negative selection against long-sequence insertions in or near genes [49]. LINEs are most enriched in state HET3 (3.4 folds) (Additional File 1: Fig. S28), and a notable property of this state is it does not show signals of H3K27me3 and acetylation marks across cell/tissue types. This property of HET3 is a pattern that would be difficult to recognize without stacked modeling and was only shared with HET4 and HET9 among states in the heterochromatin group. HET4 was also the second most enriched state in the heterochromatin group for LINEs (2.0-fold) while HET9 was not enriched, but is distinct in that it identifies regions where H3K9me3 is relatively specific to cell types in the ESC and iPSC groups. LTRs are most enriched in state HET5 (4.7 fold), and this state is marked by its highest signals of H3K9me3 compared to other states in the heterochromatin group (Fig. 4C, Additional File 1: Fig. S28). LTRs showing strong enrichment with states associated with strong presence of H3K9me3 is consistent with concatenated model chromatin state analyses [21, 50]. We also confirmed that the increased predictive power of the full-stack model over concatenated and independent annotations, which was previously seen for repeat elements overall, also held for most of the individual repeat classes (Additional File 1: Fig. S29). Overall, results of enrichment of full-stack state annotations for repeat classes offer further details in stratifying the states’ characteristics and concurrently confirm and refine existing knowledge about different repeat classes chromatin state associations.

### Full-stack states show distinct enrichments for constrained elements and conservation states

Sequence constrained elements are another class of genomic elements that are not cell-type-specific and for which the full-stack annotations showed greater predictive power than concatenated and independent annotations (Additional File 1: Fig. S19-21). We next sought to better understand the relationship between full-stack states and sequence conservation annotations. We observed 10 states that had at least a 3.4-fold enrichment for PhastCons elements (Fig. 4A). These states were associated with the TSSs or being proximal to them (TSS1-2 and PromF4-5), transcription with strong H3K36me3 signals (TxEx2 and TxEnh4), or enhancers associated with mesenchymal, muscle, heart, neurosphere, and adipose (EnhA2) (Fig. 4A,B). In contrast, seven states (HET3-4,6-7,9, Quies4, GapArtf2) were more than twofold depleted for PhastCons elements, which all had more than a 1.5 fold enrichment for repeat elements (Fig. 4A).

To gain a more refined understanding of the relationship between the full-stack chromatin states and conservation, we analyzed their enrichment using 100 previously defined conservation states by the ConsHMM method [30]. These conservation states were defined based on the patterns of other species’ genomes aligning to or matching the human reference genome within a 100-way vertebrate alignment. We observed 29 different conservation states maximally enriched for at least one full-stack state (Fig. 3B, Additional File 1: Fig. S30). These conservation states included, for example, ConsHMM state 1, a conservation state corresponding to bases aligning and matching through all vertebrates and hence most associated with constraint. ConsHMM state 1 had ≥ 10 fold enrichment for exon-associated full-stack states TxEx1-4 and TxEnh4 (Additional File 1: Fig. S30A). Another ConsHMM state, state 28, which is associated with moderate aligning and matching through many vertebrates and strongly enriched around TSS and CpG islands, had a 44.5 and 47.8 fold enrichment for TSS-associated full-stack states TSS1 and TSS2, respectively (Additional File 1: Fig. S30A). Additionally, this conservation state is consistently the most enriched conservation state for full-stack states associated with flanking and bivalent promoters (Fig. 3B, Additional File 1: Fig. S30A). ConsHMM state 2, which has high aligning and matching frequencies for most mammals and a subset of non-mammalian vertebrates and previously associated with conserved enhancer regions [30], showed > 2.7 fold enrichment for some full-stack enhancer states for Brain (EnhWk4 and EnhA6), ESC and iPSC (EnhA17,19 and EnhWk8), neurosphere (EnhWk4, EnhA2,17), and mesenchymal, muscle, heart, and adipose (EnhA2) (Fig. 3B, Additional File 1: Fig. S30A). ConsHMM state 100, a conservation state associated with alignment artifacts, was 10.9 fold and 2.1 fold enriched for full-stack state znf2 and znf1, respectively (Fig. 3A,B, Additional File 1: Fig. S30A). This is consistent with a previous analysis using concatenated annotations showing that ConsHMM state 100 was most enriched in a ZNF gene-associated chromatin state [30]. State znf2 also showed a 5.4-fold enrichment for ConsHMM state 1 which contrasts with state znf1, which showed a 0.8 fold enrichment for ConsHMM state 1, suggesting a stronger association of state znf1 with newly evolving ZNF genes or those under less constraint. This difference is also consistent with the znf2 state’s larger fold enrichment for coding exons than znf1 (10.4 vs 1.1) (Fig. 3A). The znf2 state also had a greater fold enrichment for ZNF named genes in general (68.6 vs. 20.8 fold), with those enrichment stronger and the difference greater when restricting to C2H2 annotated genes (86.8 vs. 25.1 fold) (Fig. 3A,B, Additional File 1: Fig. S31). Therefore, the full-stack annotation helped distinguish two ZNF gene-associated states, which are associated with distinct conservation states. As this example illustrates, the full-stack annotation captured conservation state enrichments that were generally consistent with those seen in concatenated annotations, but could also identify additional refined enrichment patterns.

### Specific full-stack states show distinct enrichments and depletions for structural variants

We also analyzed the enrichment of the full-stack states for overlap with structural variants (SVs) mapped in 17,795 deeply sequenced human genomes [45], and focused on the two largest classes of SVs, deletions and duplications. Abel et al. [45] analyzed the enrichments of these deletions and duplications with concatenated model chromatin states in 127 reference epigenomes [16] and observed that ZNF gene and heterochromatin states were enriched for deletions and duplications, with the enrichments being stronger in regions annotated as these states (ZNF or heterochromatin) in larger number of cell or tissue types (i.e. more constitutive ZNF or heterochromatin regions). Consistent with those previous results, using the full-stack model, we observed that of the 13 states that were among the top 10 maximally enriched states for either deletions or duplications (1.18 fold or greater), seven were in the heterochromatin group (HET1-2,4-7,9) and one was the znf2 state (Fig. 5A, Additional File 1: Fig. S32). The enrichment of structural variation in HET states is consistent with the notion that potentially larger-effect structural variants would less likely experience negative selection in these regions of the genome. As the znf2 state is most enriched for a conservation state associated with putative alignment artifacts, this raises the possibility that technical issues may be contributing to its SV enrichments (Additional File 1: Fig. S30). The other five states included two artifact states (GapArtf2-3) and three quiescent states (Quies1-2,4) (Fig. 5A). The quiescent states Quies1-2,4, despite the generally low frequencies for all marks, did have higher emission probabilities for H3K9me3 compared to other chromatin marks (Fig. 5B).

The full-stack model was also more predictive of SV than concatenated and independent ones (Additional File 1: Fig. S33-34, Additional File 6). Additionally, we verified that the full-stack annotations had higher AUROC in predicting duplications and deletions compared to annotations obtained by ranking genomic bases based on the number of cell or tissue types that a state was observed, as in the approach of [45] (“Methods,” Additional File 1: Fig. S35). These results show that the full-stack annotation can uncover enrichment patterns with SVs that are consistent with concatenated annotations, yet highlight states with greater predictive power and offer a more refined chromatin annotation of the regions enriched with SVs.

### Full-stack states gives insights into bases prioritized by different variant prioritization scores

Various scores have been proposed to prioritize deleterious variants in non-coding regions of the genome or genome-wide. These scores are based on either conservation or on integrating diverse sets of genomic annotations. Though the scores all serve to prioritize variants, they can vary substantially from each other and it is often not clear the differences among the types of bases that different scores prioritize. To better understand the epigenomic contexts of bases that each score tends to prioritize, we analyzed the full-stack state enrichment for bases they prioritize. As the scores we considered are not specific to a single cell type, the full-stack states have the potential to be more informative for this analysis than concatenated or independent annotations. We considered a set of 14 different variant prioritization scores that were previously analyzed in the context of conservation state analysis [30]. The 14 scores for which we analyzed prioritized variants in non-coding regions were CADD (v1.4), CDTS, DANN, Eigen, Eigen-PC, FATHMM-XF, FIRE, fitCons, FunSeq2, GERP++, LINSIGHT, PhastCons, PhyloP, and REMM [51–64]. For each of these scores, we first analyzed the full-stack state enrichments for the top 1% prioritized non-coding variants relative to the background of non-coding regions on the genome (“Methods”).

In the top 1% prioritized non-coding bases, 19 states were among the top five most enriched states ranked by at least one of the 14 scores (Fig. 5C, Additional File 1: Fig. S36-37). These 19 states include nine states in promoter-associated groups, five states in enhancers-related groups, three states in the exon-associated transcription group, one polycomb repressed state, and one DNase state (Fig. 5C). Seven scores (DANN, Eigen, Eigen_PC, funSeq2, CDTS, CADD, and REMM) had their top five enriched states exclusively associated with promoter and TSS states, with enrichments ranging between 8.6 and 70 fold (PromF2-5, TSS1-2, BivProm1-2,4) (Fig. 5C). In contrast, the fitCons score showed depletions for three of these states and relatively weaker enrichment for the others. This difference might be related to fitCons’ approach of prioritizing bases showing depletion of human genetic polymorphisms, potentially without sufficiently accounting for the increased mutation rates in regions with high CpG content that are observed in promoter-associated states (Additional File 1: Fig. S42) [46]. FIRE’s prioritized variants showed depletions in the bivalent promoter states (BivProm) and PromF5, which have generally lower average gene expression across cell types (Fig. 3C). This depletion is likely reflective of the fact that FIRE was trained to prioritize variants in cis-expression quantitative trait loci (cis-eQTLs) in one cell type (LCL) [57], and few eQTLs are expected to be proximal to genes with limited or no expression in that cell type. Enhancer states EnhA2-3,17 were among the states in the top five most enriched for FATHMM-XF, GERP++, LINSIGHT, PhastCons, and PhyloP prioritized non-coding variants. In contrast, FIRE, DANN, and CDTS were depleted for prioritized variants in all these enhancer states (Fig. 5C). FIRE and fitCons showed strong enrichment for exon states (TxEx1-3), which are associated with coding regions, even though coding bases were excluded in this analysis (Fig. 5C). FATHMM-XF had the greatest relative enrichment (~ 10 fold) for the primary DNase state (DNase1), which is associated with a CTCF state from a concatenated model defined in six cell types [31], and was the only score for which this state was among the top five most enriched states (Fig. 5C, Additional File 1: Fig. S14, 36).

We conducted similar analyses based on top 5% and 10% prioritized non-coding variants and observed relatively similar patterns of enrichments, though there did exist some differences at these thresholds (Additional File 1: Fig. S36, 38-39). One difference was that alignment artifact states GapArtf2-3 were among the top two most enriched states with top non-coding bases prioritized by FATHMM-XF, while a number of other scores showed depletions for these states (Additional File 1: Fig. S36). In addition, we analyzed top 1%, 5%, and 10% prioritized variants genome-wide from 12 of the scores (“Methods”) (Additional File 1: Fig. S37-40). Compared to the non-coding analysis, we saw a majority of scores had exon-associated transcription states (TxEx1-TxEx4) among the top five enriched states with top 1% variants genome-wide, while we saw no enhancer state among the top five enriched states with top 1% variants by any score and only one enhancer state among the top five by one score (GERP++) for top 5% and 10% variants.

Overall, this analysis showed that the scores tend to prioritize bases in different epigenetic contexts. As the scores vary in the genomic features selected as input, and the predictive model for scoring bases, it is expected that different methods may show higher scores for in different classes of genomic contexts. By analyzing the state enrichments, one can gain some expectations for what types of evaluation criteria different scores might perform better, but we note that this analysis is not trying to directly conclude one method is preferred. Also, while in general it is difficult to conclude confidently what enrichments are due to technical or biological biases, by comparing enrichments across scores and considering what else is known about the states, one can still gain insights into this. For example, the inconsistent enrichments of different methods for prioritized variants in GapArtf2-3 states (Additional File 1: Fig. S36), along with these states’ association with sequencing artifacts, are suggestive of technical biases. Similarly, DANN’s top 1% non-coding bases showing enrichments in five heterochromatin states, while not showing any enrichments in enhancer states, and no other scores showing enrichments in heterochromatin states, are also suggestive of technical biases (Additional File 1: Fig. S37).

We verified that the full-stack annotation showed the highest AUROC in recovering the top 1% non-coding variants compared to all 18-state concatenated annotations for all 14 scores (Additional File 1: Fig. S41). Compared to all 100-state independent annotations, the full-stack model showed the highest AUROC for 13 out of 14 scores in all 127 cell types (Additional File 1: Fig. S41).

### Full-stack states show distinct enrichments and depletions for human genetic variation

We next analyzed full-stack states for their enrichment with human genetic sequence variation. We calculated enrichments of full-stack states with genetic variants sequenced in 15,708 genomes from unrelated individuals in the GNOMAD database stratified by minor allele frequencies (MAFs) [46]. Across eleven ranges of MAFs, the state enrichments ranged from a 2-fold enrichment to a 4-fold depletion (Additional File 1: Fig. S42). As expected, the state associated with assembly gaps (GapArtf1) is most depleted with variants, regardless of the MAF range. At the other extreme, state Acet1, which is associated with simple repeats, is the most enriched state with variants for all ten minor allele frequency (MAF) ranges that are greater than 0.0001, with fold enrichments between 1.8 and 2.0 (Additional File 1: Fig. S42). We verified that the high enrichment for state Acet1 was not specific to GNOMAD’s calling of variants as it had a 2.0 fold enrichment with common variants from dbSNP (“Methods”) (Additional File 1: Fig. S42). TSS and promoters associated states, PromF4 and TSS1-2, were maximally enriched for variants in the lowest range of MAF (0 < MAF ≤ 0.0001), 1.5–1.7 fold. The enrichment of variants for these states decreased as the MAF ranges increased, falling to 0.8–1.2 fold for variants of the highest range of MAF (0.4–0.5) (Additional File 1: Fig. S42). The high enrichment for states PromF4 and TSS1-2 for rare variants, despite their being the most enriched states with PhastCons conserved elements, can be explained by these states’ high enrichment of CpG dinucleotides, which are associated with higher mutation rates (Fig. 3A, Additional File 1: Fig. S42) [46]. At the same time, purifying selection can have a weaker impact on larger-effect rare variants than on larger-effect common variants. We also observed the pattern of decreasing enrichments for variants with increasing MAF in other states associated with transcriptional activities, enhancers, DNase, or promoters (Additional File 1: Fig. S42). This pattern was not observed in most states from other groups such as heterochromatin, polycomb repressed, quiescent, and acetylations only (Additional File 1: Fig. S42).

To better identify states with a depletion of common variants that are more likely due to negative selection, we ranked states based on their ratios of enrichments for the rarest variants (MAF < 0.0001) relative to the most common variants (MAF 0.4–0.5) (Fig. 5D). The states with the highest ratio included a number of flanking promoter (PromF3-4) and exon-transcription states (TxEx1,2,4) that were also associated with strong sequence conservation across species (Fig. 3B, Fig. 5D). These results are consistent with previous analyses supporting a depletion of common human genetic variation in evolutionary conserved regions [67]. States associated with assembly gaps and alignment artifacts (GapArtf1-3), quiescent (Quies3), or acetylations and simple repeats (Acet1) were most depleted for rare variants relative to the common variant enrichment (Fig. 5D).

### Full-stack states show enrichment for phenotype-associated genetic variants

We next analyzed the relationship between the full-stack states and phenotypic associated genetic variants. We first evaluated the enrichment of the full-stack state for variants curated into the Genome-wide Association Study (GWAS) catalog relative to a background of common variation [65] (“Methods”). This revealed six states with at least a twofold enrichment (Additional File 1: Fig. S43). Four of these states, TxEx1-2,4 and TxEnh4, were all transcription-associated states that are ≥ 10-fold enriched with coding sequences and ≥ 11 fold for ConsHMM state 1, associated with the most constraint in a sequence alignment of 100 vertebrates (Fig. 3B). This observation is consistent with previous results that GWAS catalog variants show enrichments for coding sequence and sequence constrained bases [30, 66, 67]. The other two states with greater than twofold enrichment for GWAS catalog variants relative to common variants were two promoter states, PromF2-3 (Additional File 1: Fig. S43). On the other hand, four states were more than twofold depleted for GWAS catalog variants and were associated with artifacts (GapArtf2-3) or quiescent and polycomb repressed states with weak signals of H3K9me3 (Quies5) or H3K27me3 (ReprPC8) (Additional File 1: Fig. S43). Both Quies5 and ReprPC8 are highly specific to chrX, 18.3 and 19.1 fold enriched respectively (Additional File 1: Fig. S43).

We also analyzed the full-stack state enrichments for fine-mapped variants previously generated from a large collection of GWAS studies from the UK Biobank database and other public databases [68]. Specifically, we considered separately the fine-mapped variants from two fine-mapping methods, CAVIARBF [47] and FINEMAP [48], for 3052 traits. For each method and trait, we identified the single variants that had the greatest probability of being causal at a set of distinct loci and computed the enrichment of these variants for the full-stack states relative to a background of common variants (“Methods”). Fold enrichment results of full-stack states for the most likely causal variants were highly consistent between fine-mapping methods (FINEMAP and CAVIARBF) (Additional File 1: Fig. S44). The ten states maximally enriched with fine-mapped variants relative to common variants, which were the same states by both methods, included five states associated with flanking and bivalent promoter activities (PromF2-5, BivProm4), an enhancer state associated with blood and thymus (EnhA9) and an enhancer state associated with most other cell types except blood cell types (EnhA1), and three highly conserved transcription-associated states (TxEnh4,6, TxEx4) (Fig. 5E). Notably, five of 10 states maximally enriched with fine-mapped variants, PromF2-5, and BivProm4, were associated with promoter regions and also among the 19 states most enriched with top 1% prioritized variants by at least two of the 14 different variant prioritization scores (Fig. 5C, E). These results show that there are agreements in the types of full-stack states preferentially overlapped by phenotype-associated fine-mapped variants and variants predicted to have greater effects based on variant prioritization scores. We also confirmed that the full-stack model consistently had higher AUROC in predicting locations of fine-mapped variants within a background of common variants, compared to the concatenated and independent annotations in all cell types (Additional File 1: Fig. S45-46).

### Full-stack states show enrichments for cancer-associated variants

In addition to investigating germline variants, we also investigated the enrichment of full-stack states for somatic variants identified from whole-genome sequencing of cancer samples. We analyzed data of variants from four cancer types that have the largest number of somatic variants in the COSMIC database [69]: liver, breast, pancreas, and haematopoietic and lymphoid tissue (“Methods”). Sixteen states were among the top 10 most enriched with at least one type of cancer’s associated variants (1.2–1.4 fold in breast cancer, 1.2–5.6 fold in lymphoid cancer, 1.2–5.4 in liver cancer, 1.4–4.2 in pancreas cancer) (Fig. 5F). Among these 16 states, 15 states showed higher signals of H3K9me3 compared to most other chromatin marks, including seven states in heterochromatin group (HET1-2, 4-7,9), four states in quiescent group with weak emissions of H3K9me3 (Ques1-2,4-5), one state in the polycomb repressed group with weak signals of H3K9me3 and H3K27me3 (ReprPC8), one state in the acetylation group with signals of H3K9me3 and various acetylation marks (Acet1), and two artifact-associated states with higher signals of H3K9me3 and DNase relative to other marks (GapArtf2-3) (Fig. 5G). This pattern of H3K9me3-associated states being enriched with somatic mutations in cancer was previously confirmed in multiple studies where H3K9me3 showed positive association with mutation density across different types of cancer cells [70–72]. One possible explanation for this association is the more limited access of DNA mismatch repair machinery in these regions due to the tightly packed nature of the genome in heterochromatin [73, 74]. Notably, the GapArtf2-3 states, which had strong satellite repeats enrichments (Fig. 4C, Additional File 1: Fig. S28), were the top two most enriched states with somatic variants associated with liver, pancreas, and haematopoietic and lymphoid tissue (haem-lymphoid) cancers (2.0–5.6 folds enriched) (Fig. 5F, Additional File 1: Fig. S47). We suspect that the enrichments in these putative alignment artifact states are driven at least in part by false variant calls due to sequence mapping errors associated with these regions. Similarly, enrichments of somatic mutations in haem-lymphoid cancer in state Acet1 are also suggestive of the possibility of false calls given this state’s combination of H3K9me3 and acetylation signal and enrichment for simple repeats (Figs. 2 and 4C, Additional File 1: Fig. S42). We note that the presence of cancer variants is better recovered by full-stack annotation as compared to the concatenated and independent chromatin state annotations for all four cancer types (Additional File 1: Fig. S48-49).

## Discussion

We demonstrated a large-scale application of the stacked modeling approach of ChromHMM using over a thousand epigenomic datasets to annotate the human genome. In the datasets, 32 chromatin marks and 127 reference epigenomes were represented. We note that even though not every chromatin mark was profiled in every reference epigenome, we were still able to directly apply the stacked modeling to such data. Previously, concatenated models were applied to observed and imputed data [40]; however, we chose not to use imputed data as input to the full-stack model primarily since imputed data would still be based on the same observed input data used in stacked modeling. We conducted extensive enrichment analyses of the states with various other genomic annotations and datasets, including gene features, genetic variation, repetitive elements, comparative genomic annotations, and bases prioritized by different variant prioritization scores. These analyses highlighted diverse enrichment patterns of the states. Using these enrichments along with the model parameters, we provided a detailed characterization of each of the 100 states in the model.

We grouped these 100 states into 16 groups that included promoters, enhancers, transcribed regions, polycomb repressed regions, and zinc finger genes among others. We also highlighted important distinctions among states within the groups. In many cases, identifying these distinctions was enabled by the full-stack modeling using data from multiple cell types for genome annotation. For example, we identified enhancer and repressive states that were active in different subsets of cell types. We also highlighted how different states in some of the groups such as those associated with ZNF genes showed distinct enrichments for conservation states. Overall, the full-stack model showed enrichment patterns supporting observations based on concatenated or independent annotations, while providing a more detailed stratification of genomic regions into chromatin states with more refined associations with other genomic information. We provide extensive characterizations of full-stack states in Additional Files 2-6 that we expect will be a resource in future applications of the full-stack annotations.

The full-stack modeling has advantages to commonly used concatenated and independent chromatin state annotations in several respects. First, the full-stack model can learn patterns of signals of the same or different assays across cell types and hence can provide a unified view of all the data and directly uncover states that correspond to constitutive or cell-type-specific activities. For example, a state from the model, HET9, was associated with only the mark H3K9me3 specifically in ESCs and iPSCs even though this mark is typically associated with constitutive repression. Second, the full-stack annotation consistently showed better recovery of various genomic features compared to concatenated and independent annotations. This improvement is expected since full-stack models can leverage information from multiple cell types for genome annotations. Third, in cases where it is not desirable to focus on only one specific cell or tissue for analysis, the full-stack modeling can bypass the need to pick one such cell or tissue type or to consider a large number of different concatenated or independent chromatin state annotations simultaneously. Such cases may arise when studying other genomic information that is not inherently cell-type-specific such as genome variation and sequence conservation. Overall, the full-stack model provides a universal annotation of the genome that can be viewed as a single track in a genome browser or used for a variety of downstream bioinformatic analyses.

Despite these advantages, there are trade-offs in using the stacked modeling approach, and we emphasize that the full-stack annotations should be considered a complement to and not a replacement of the concatenated or independent annotations. Compared to typical concatenated models, the full-stack model has increased model complexity because of the increased number of parameters from the larger number of states and input features, which can make interpreting some model states relatively more difficult. Additionally, if one is interested in a specific cell type, then corresponding concatenated or independent annotations can have advantages in that all the annotations are directly informative about the chromatin states in the cell type of interest. An additional trade-off is that with the stacked model, it is not possible to incorporate additional data without relearning a model, while for a concatenated model one can annotate a new cell based on an existing model, provided that the set of marks in the new cell type are the same as the existing model. We also note that post hoc concatenated state annotations can also be used for cross-cell-type analyses, particularly on a per-state basis by analyzing the frequency of a specific state across cell types or potentially in other ways. While per-state analyses using concatenated annotations can be relatively straightforward and informative, they give only partial and potentially oversimplified views of all the data, ignoring distinctions among different states. Whether to use concatenated, independent or full-stack annotations will depend on the specific application. Concatenated or independent annotations may be preferable when one is interested in studying a specific cell type, while full-stack annotations may be preferred in joint analyses of multiple cell types.

We expect many applications of the full-stack annotations that we generated here and they have already begun to be applied in other work [75–77], which we expect to further elucidate the biological significance of different states. The full-stack annotation can be used as a resource to interpret genetic variation. A possible avenue for future work is to incorporate the full-stack annotation into scoring methods to better predict genetic variants’ phenotypic influences. Given the increasing availability of epigenomic datasets [18], future work could also learn new stack models to incorporate such data. The state characterizations (Additional Files 2-6) and analyses introduced through this work will be useful in interpreting biological implications of new models’ states. Future work can also include training and deriving the full-stack annotations for key model organisms such as mice. This work provides a new annotation resource for studying the human genome, non-coding genetic variants, and their association with diseases.

## Methods

### Input data and processing

We obtained coordinates of reads aligned to Human hg19 in .tagAlign format for the consolidated epigenomes as processed by the Roadmap Epigenomics Consortium from https://egg2.wustl.edu/roadmap/data/byFileType/alignments/consolidated/. In total, we obtained data for 1032 datasets and their corresponding input control data. The datasets correspond to 127 reference epigenomes, 111 of which were generated by the Roadmap Epigenomics Consortium and 16 were generated by the ENCODE Consortium. Of the 1032 datasets, 979 were ChIP-seq data targeting 31 different epigenetic marks and 53 were of DNase-seq (Additional File 1: Fig. S2). For each of the 127 reference epigenomes there was a single ChIP-seq input control dataset. For the 53 reference epigenomes that had a DNase-seq dataset available, there was an additional DNase control file.

We next binarized the data at 200 base pair resolution using the BinarizeBed command of ChromHMM (v.1.18). To apply BinarizeBed in stacked mode, we generated a cell_mark_file input table for ChromHMM with four tab-delimited columns. The first column had the word “genome” for all datasets, the second column contained entries of the form “<EID > - < mark>” where “EID” is the epigenome ID and “mark” is the mark name, the third column specifies the name of the corresponding file with aligned reads, and the fourth column is the name of the file with the corresponding control reads. Each row in the table corresponds to one of the 1032 datasets.

In order to reduce the memory and time needed to execute BinarizeBed on a large number of datasets, we split the cell_mark_file table into 104 smaller tables with each table having at most 10 entries corresponding to at most 10 datasets to be processed. This was done with a custom script, but the same functionality has been included with the “-splitcols” and “-k” flags of BinarizedBed in ChromHMM v1.22. We then ran BinarizeBed in parallel for each of these smaller cell_mark_file tables and generated output into separate subdirectories. We ran BinarizeBed with the option “-gzip” which generates gzipped files.

To merge data from the 104 subdirectories from the previous step into files containing binarized data of all datasets, we ran the command “MergeBinary,” which we added in v1.18 of ChromHMM. We ran the command with the options “-gzip -splitrows”. The “-splitrows” option generates multiple files of merged binarized data for each chromosome, where, under the default settings that we used, each file contains data for a genomic region of at most 1 MB. Splitting each chromosome into smaller regions allows the model learning step of ChromHMM to scale in terms of memory and time to the large number of input data tracks (i.e., features) that we were using. We used chr1-22, chrX, chrY, and chrM in the binarization and model learning.

We note that we chose not to use imputed data as input to the full-stack model. A main reason is, as noted above, imputed data would still be based on observed data from the 1032 datasets used for stacked modelling. Another reason is that imputed data may have artificially high correlations across the cell types, which can particularly be the case for marks that were experimentally mapped in few cell types. This could potentially cause the stacked modeling to devote many states that correspond to heavily correlated and less informative tracks of imputed data.

### Training full-stack model and generating genome-wide state annotations

We learned the full-stack chromatin state model for the 1032 datasets using the LearnModel command of ChromHMM (v1.18). This version of ChromHMM includes several options that we added to improve the scalability when training with large numbers of features. One of these features was to randomly sample different segments of the genome for training during each iteration, instead of training on the full genome. This sampling strategy was previously used by ConsHMM [30], which was built on top of ChromHMM. We note that this sampling procedure can also be applied to learn concatenated models, in which case there would be no requirement that the same segments are sampled in each cell type. However, sampling can be unnecessary for typical instances of learning concatenated models, given that it usually involves fewer different inputs to the model, fewer number of states, and in training these models, ChromHMM is able to tolerate more parallel cores without reaching memory limits.

To learn the full-stack model with input data processed as outlined above, we used ChromHMM’s LearnModel command with the options “-splitrows -holdcolumnorder -pseudo -many -p 6 -n 300 -d -1 -lowmem -gzip”. The “-splitrows” flag informs ChromHMM that binarized data for a chromosome is split into multiple files, which reduces the memory requirements and allows ChromHMM to select a subset of the genome to train on for each iteration. The “-holdcolumnorder” flag prevents ChromHMM from reordering the columns of the output emission matrix, which saves time when there are a large number of features.

The “-pseudo” flag specifies that in each update of model parameters that ChromHMM adds a pseudo-count of one to the numbers of observations of transition between each pair of states, presence and absence of each mark from each state, and initial state assignments of the training chromatin state sequence. This prevents model parameters from being set to zero, which is needed for numerical stability when some features are sparse and ChromHMM does not train on the full genome in each iteration.

The “-many” flag specifies to ChromHMM to use an alternative procedure for calculating the state posterior probabilities that is more numerically stable when there are a large number of features. The procedure is designed to prevent all states from having zero posterior probability at any genomic position, which can happen due to the limits of floating-point precision. The procedure does this by leveraging the observation that only the relative product of emission probabilities across states are needed at each position to determine the posterior probabilities. Specifically, for each position, the procedure initializes the product of emission probabilities for all features, i.e., the emission product, from each state to one. For each feature, the procedure then multiplies the current emission products from each state by the emission probability of the feature in the state, and divides all the resulting products by their maximum to obtain updated emission products. We iteratively repeat these steps of multiplication and normalization until all features have been included into the calculation of relative emission products across states.

The “-p 6” flag specifies to ChromHMM to train the model in parallel using 6 processors. The “-n 300” flag specifies to ChromHMM to randomly pick 300 files of binarized data, corresponding to 300 regions of 1 MB (or less if the last segment of the chromosome was selected) for training in each iteration. The “-d -1” option specifies to ChromHMM to not require an evaluated likelihood improvement between iterations to continue training since evaluated likelihood decreases are expected, as on each iteration the likelihood is evaluated on a different subset of data. The “-lowmem” flag has ChromHMM reduce main memory usage by not storing in main memory all the input data and instead re-loading from disk when needed. The asymptotic worst-case time and memory of the model learning is discussed in the Additional File 8.

### Choice of number of states

We trained full-stack models with 10–120 states, in 5 state increments, using the data and procedure outlined above. For each of these models, we calculated an estimated Akaike Information Criterion (AIC) [78] and Bayesian Information Criterion (BIC) [79] value based on a subset of the genome (Additional File 1: Fig. S3). AIC and BIC are calculated based on the log likelihood for 300 random 1-Mb regions outputted by ChromHMM from the last training iteration. In general, both the AIC and BIC decrease as the numbers of states increase, but with diminishing improvements. We also applied the CompareModels command of ChromHMM [25] with the 100-state model as a reference model, which reports for each state of the 100-state model, the maximum correlations of emission parameters between the state in the 100-state model and any state for each other model (Additional File 1: Fig. S3C). We conducted a similar analysis with the emission parameters of H3K4me1, hence for each state in each model, we obtained emission probabilities of H3K4me1 in 127 cell types. For this analysis, for each of the 19 tissue groups previously defined [16], we calculated the correlation of each state’s H3K4me1 emission parameters with a binary vector indicating if the cell type in each parameter is in the tissue group (1) or not (0). We then report, for each tissue group, the maximum correlations among all states in each model (Additional File 1: Fig. S3D). These analyses showed, for instance, that a state corresponding to brain-specific enhancers in the 100-state model, EnhA6, was well captured in models with 55 states or more (correlation of ≥ 0.98 with states in models with ≥ 55 states and correlation ≥ 0.78 for H3K4me1-emissions with the Brain binary vector) (Additional file 1: Fig. S3C, D). A state characterized as enhancers specific to Huvec cells in the 100-state model, EnhA20, was well captured in models with 100 or more states (correlation ≥ 1.00 based on all marks’ emission parameters) (Additional file 1: Fig. S3C).

Additionally, for models with 20, 40, 60, 80, 100, and 120 states, we also produced genome annotations and then quantitatively compared the chromatin state annotations from models in terms of their power to predict locations of various other genomic annotations not used in the model training: Exon, Gene Body, TSS, TSS2kb, CpG Islands, TES, laminB1lads elements (listed in section “External annotation sources”). Specifically, we evaluated the predictive power using the AUROCs that are calculated as described in a subsection below. Across different genomic contexts, as the number of full-stack states increased, the AUROC increased, but with diminishing improvements (Additional File 1: Fig. S3A).

To balance the additional information available in models with an increased number of states, while keeping the number of states manageable for interpretation and downstream analysis, we choose to focus on a model with 100 states. We note that this choice is greater than previously used for concatenated models [3, 16, 21], which reflects the additional information available for genome annotation based on the large number of datasets spanning many cell types that we are using.

### Lifting chromatin state annotations to hg38

The full-stack chromatin state annotations were learned directly in hg19, as this was the assembly for which uniformly processed data from the Roadmap Epigenomics integrative analysis was available. Learning full-stack annotation directly in hg19 allowed direct comparison with existing concatenated annotations. We also generated a version of full-stack annotation in hg38 by lifting over the original annotation from hg19 to hg38. To do this, we first wrote the hg19 chromatin state annotation into .bed format such that each line corresponds to a 200-bp interval. We then used the liftOver tool [80] with default parameters to generate the annotation in hg38. We did not annotate bases in hg38 if multiple bases in hg19 mapped to it. In total, there are 1,186,379 200-bp segments that were not mapped from hg19 to hg38, of which 98.7% fall into an assembly gap and 99.6% fall into the full-stack state primarily associated assembly gaps (GapArtf1) (Additional File 1: Fig. S50). In hg38 on chr1-22, X, and Y, 92.9% of bases are annotated to a state, and that number increases to 97.1% when excluding assembly gaps. We verified that we saw highly similar state fold enrichments for similar annotations between hg19 and hg38 (Additional File 1: Fig. S18). The sources of external annotations from hg38 are outlined in section “External annotation sources” below.

### Summary sets of datasets

To construct a summary visualization of the emission parameters with a reduced set of features that approximate the annotation from the full model, we applied a greedy search over the 1032 input datasets as described in Additional File 8. We applied this procedure to reduce the 1032 input datasets to 80 summary datasets.

### Identifying states with differential association of marks for individual tissue groups

For each state, we tested for combinations of the 8 most profiled marks, and 19 tissue groups previously defined [16], whether the emission probabilities of features associated with one chromatin mark and in one tissue group was significantly greater than those of features associated with the same mark and not in the tissue group. The eight marks that we tested were H3K9me3, H3K4me1, H3K4me3, H3K27me3, H3K36me3, H3K27ac, H3K9ac, and DNase. H3K27ac, H3K9ac, and DNase were profiled in 98, 62, and 53 reference epigenomes, respectively, and the remaining five marks in 127 reference epigenomes. For tests involving H3K27ac, H3K9ac, and DNase, we excluded tissue groups for which there were no datasets. In total, there were 14,200 tests among 100 states, 8 chromatin marks, and 19 tissue groups. For each combination of state, chromatin mark, and tissue group being tested, we applied a one-sided Mann-Whitney test to test whether the emission probabilities of the state for the features associated with the tested mark in the tested tissue group are greater than those in other tissue groups. The Bonferroni-corrected *p* value threshold based on a significance level of 0.05 to declare a test significant was 3.5e− 6.

### Computing coefficients of variation across different tissue groups

For each state, we looked into the emission probabilities of datasets associated with six chromatin marks strongly associated with promoter or enhancer activities (DNase, H3K27ac, H3K4me1, H3K4me2, H3K4me3, H3K9ac). We grouped these datasets based on their associated chromatin mark and tissue groups and calculated the average emission probabilities of datasets in each chromatin mark-tissue group combination. For each state and chromatin mark combination, we then calculated the coefficient of variation across different tissue groups, in terms of average emission probabilities from the previous step. For each group of states, we averaged the resulting coefficients of variation across states of the same group. The results show the average coefficients of variation of emission probabilities across different tissue groups for each state group-chromatin mark combination.

### Computing fold enrichments for other annotations

All overlap enrichments for external annotations were computed using the ChromHMM OverlapEnrichment command. We used the “-b 1” flag, which specifies a single base binning resolution of the annotations. This “-b 1” flag is necessary when computing enrichments based on the hg38 liftOver annotations, which no longer respects the 200-bp segment coordinate intervals from hg19. Including this flag gives the same results when applied to annotations from hg19 with 200-bp segments, though with extra computational costs. We also included the “-lowmem” flag to specify the lower memory usage option. The ChromHMM command OverlapEnrichment computes fold enrichment between chromatin states and provided external annotations relative to a uniform genome-wide background distribution. More specifically, the fold enrichments are calculated as:

$$ {FE}_{x,s}=\frac{\frac{\# SX}{\#X}}{\frac{\#S}{\#G}}=\frac{\frac{\# SX}{\#S}}{\frac{\#X}{\#G}}=\frac{\# SX\cdotp \#G}{\#S\cdotp \#X} $$

where

*FE*_*x*, *s*_: fold enrichment of state *s* in genomic context *x*

#*S*: number of genomic positions belonging to the state *S*

#*X*: number of genomic positions where genomic context *X* is present

#*SX*: number of genomic bins that overlap both state *S* and genomic context *X*

#*G*: number of genomic positions in the entire genome.

### Enrichment and estimated probabilities of overlap with 25-state concatenated annotations

We obtained per-cell type chromatin state annotations based on a 25-state ChromHMM model learned using the concatenated approach for 127 reference epigenomes, which we will refer to as cell types for ease of presentation, from the Roadmap Epigenomics project [16, 40]. This model was trained based on imputed data for 12 marks. We hereafter refer to this model as the CT-25-state model. As per the design of the concatenated approach of ChromHMM, CT-25-state model generates per-cell-type chromatin state annotations for each of the 127 cell types, and the 25 states’ characteristics are shared across 127 cell types. For each of these 127 cell types, we calculated overlap enrichments between the 100 full-stack states and the CT-25-states, resulting in 127 tables of size 100-by-25. We summarized this information by reporting, for each of the 100 full-stack states, and 127 cell types, the state in CT-25-state model that is maximally enriched, resulting in a 100-by-127 table (Additional File 1: Fig. S8). We also provided detailed comments about the patterns of maximum-enriched states observed across 127 cell types for each full-stack state in the Additional File 5 to serve as a resource for future applications. We also reported, for each of the 100 full-stack states and each CT-25-state, the maximum and median values of fold enrichments across 127 cell types (Additional File 5).

In addition, we also estimated for each combination of (1) CT-25 state, (2) cell type group, and (3) full-stack state, the probability that a genomic position being annotated as the corresponding full-stack state will overlap with the corresponding CT-25 state in a cell type from the corresponding cell group. The 19 groups of cell types were previously defined by the Roadmap Epigenomics Consortium [16]. To compute the target estimate probabilities, for each full-stack state, we sampled 100 genomic bins (each of length 200 bp) that are assigned to that full-stack state. Second, in each of the 127 cell types, we report the frequency that the sampled regions of each full-stack state overlapped with each CT-25 state. We repeated such a process 21 times. We then calculated the average frequencies of overlap between each full-stack state and each CT-25 state, across 21 random samplings and across the cell types in each group (Example: Blood, ESC). This results in a table of size 100 full-stack states by 475 combinations of CT-25 states and 19 cell groups, with each cell showing the values of estimated probabilities (Additional File 1: Fig. S9). These values, along with detailed comments about patterns of these overlap probabilities for each full-stack state, are available in Additional File 5.

### Receiver operator characteristic curve analysis for predicting external annotations

To evaluate how well the chromatin state annotations from different ChromHMM models can inform us about the position of external genomic annotations, we computed the receiver operator characteristic (ROC) in a procedure as follows: First, we divided the genome into 200-bp bins, and randomly partitioned 50% of the bins for training and the remaining 50% for testing. Second, we computed the enrichment of the target external annotation with each chromatin state on the training data, and ranked states in decreasing order of such enrichments. We used this ranking of states to iteratively add genomic bases assigned to the states as our predictions of bases that overlap the target annotation in the testing dataset. Based on the overlap of the predictions and the target annotation at each iteration, we plotted ROC curves and summarized the information by computing area under the ROC curves (AUROC).

### Concatenated and independent annotations used to compare against full-stack annotations

In evaluating how predictive the full-stack model is at annotating external genomic elements, we compared the full-stack model to two sets of per-cell-type chromatin state annotations in terms of their ability to predict external annotations. One set of annotations was the 18-state ChromHMM annotations from Roadmap Epigenomic Project [16], which was based on a model trained using the concatenated approach and observed data of six chromatin marks (H3K4me1, H3K4me3, H3K9me3, H3K27ac, H3K27me3, and H3K36me3) in 98 cell types. In this model, we have a common set of state definitions across cell types, but unique state annotations for each cell type. The second set of annotations were based on models learned independently in each of the 127 cell types. In learning these models, we partitioned the 1032 datasets used to learn the full-stack model into 127 subsets based on their associated cell type. For each of the 127 cell types, we applied ChromHMM to learn a 100-state ChromHMM model using only the observed data in the corresponding cell type. The number of states is similar to that in the full-stack model, to control for this variable in the evaluation. This process generates 127 models, each used to generate independent annotations in one cell type. The independent model learning approach of ChromHMM differs from the concatenated approach because the model parameters (state emission, transition, and initial probabilities) are different for different cell types, while state parameters in concatenated model are shared across cell types. However, these two approaches both produce chromatin state annotations on a per-cell type basis. We learned these independent models with the same ChromHMM parameters as described above for the full-stack model, with the exception of using the “-init random” flag to randomly initialize models’ parameters. Even when we specified the number of states to ChromHMM as 100, however, we note that due to the large number of states relative to the input tracks, for some of these models, fewer than 100 distinct states ended up being assigned to positions in the genome.

### Computing fine-mapped variant enrichment

To compute enrichment of full-stack states for phenotypically associated fine-mapped variants, we downloaded data on fine-mapped variants for 3052 traits from CAUSALdb [68]. Specifically, we obtained posterior probabilities of variants being causal based on two fine-mapping methods, FINEMAP [48] and CAVIARBF [47], which do not use epigenomic annotations as part of the fine-mapping procedure. For each method and trait combination, we separately partitioned the provided set of potential causal variants into distinct loci. To form the distinct loci, we merged neighboring variants into the same loci until there was at least 1-MB gap between the two closest variants from different loci. Separately for each fine-mapping method, trait, and locus combination, we selected the single variant with the highest posterior probability of being causal. For each fine-mapping method, we took the union of variants across 3052 traits, and then calculated the fold enrichments for the union of these lead variants with stacked ChromHMM states relative to the enrichment with a background set of common variants from dbSNP build 151 (hg19). To do this, we separately computed the enrichments of both of these sets relative to a genome-wide background, and then divided the enrichment of the foreground set (lead fine-mapped variants) by the enrichment of the background set (common variants). The dbSNP variants were obtained from the UCSC genome browser [81].

### Computing structural variant enrichments

To compute enrichment of the full-stack states for structural variant enrichments, we obtained data of structural variants from [45]. We used the B38 call set, which was in hg38 and used for the analysis presented in [45]. We filtered out structural variants that did not pass the quality control criteria of [45]. We then separately considered structural variants annotated as either a deletion or a duplication, for which there were 112,328 and 28,962 sites respectively.

Since the structural variants were defined in hg38, we computed their enrichment for ChromHMM state annotations from full-stack, concatenated and independent models that were lifted over from hg19 to hg38, following the procedure outlined above. Next, we followed the enrichment analysis procedure outlined above to compare full-stack vs. concatenated and independent chromatin state annotations’ power in recovering structural variants.

To compare the power of full-stack state annotations vs. concatenated state annotation frequency, we utilized the 15-state concatenated chromatin state annotation for 127 cell types (reference epigenomes) from the Roadmap Epigenomics Consortium. We followed the analysis outlined in [45], for each of the 15 concatenated model states, we annotated genomic positions based on the number of cell types in which the state is present (ranging from 0 to 127), resulting in 15 state frequency annotations per genomic position. We then ordered positions in decreasing order of frequency and applied the procedure above for computing ROC curves to compare the predictive power of the state’s annotation frequency against the full-stack annotation.

### Computing enrichments with cancer-associated variants

We obtained data of somatic mutations associated with different types of cancer from COSMIC non-coding variants dataset v.88 in hg38 [69]. We selected from this dataset variants that were from whole-genome sequencing. We filtered out variants that overlap with any of the following: the hg38 blacklisted regions from the ENCODE Data Analysis Center (DAC) [82], hg38 dbSNP (v151) set of common variants from the UCSC genome browser database, or regions annotated as coding sequence (“CDS”) based on GENCODE v.30 hg38 [83] gene annotations. We decided to restrict this analysis to the four cancer types with most number of variants present in the dataset in hg38: liver (1,351,417), pancreas (500,930), haematopoietic and lymphoid tissue (354,501), and breast (323,751). We then lifted over these sets of variants from hg38 to hg19, resulting in 1,351,159, 500,798, 354,351, and 323,685, variants respectively. To obtain a background set of genomic locations for the enrichment analysis, we filtered from the genome the same set of hg38 annotations of blacklisted regions, common variants, and coding sequences as we did for the foreground of COSMIC mutations. We then lifted over these remaining positions from hg38 to hg19 to obtain the background. We calculated the enrichment of chromatin states with cancer-associated variants by first calculating the enrichment values of chromatin states with filtered variants associated with each of the four cancer types, and the enrichment values with background set of genomic bases, all relative to the whole genome. We then divided the cancer-associated variant enrichment values by the background base enrichments.

### Gene Ontology enrichments

We calculated the GO enrichments of genes being in proximity to each full-stack state annotation using GREAT [84]. For each full-stack state, we reported the 5 GO biological processes and 5 GO molecular functions with the lowest FDR-corrected *p* values, ranked by GREAT [84]. All bar plots showing the top GO terms and negative log base 10 *p* values of enrichments with full-stack states are available in Additional File 2.

### External annotation sources

The sources for external annotations for enrichments analyses, not given above, were as follows (all download links are listed in Additional File 9):

- Annotations of CpG islands, exon, gene bodies (exons and introns), transcription start sites (TSS), transcription end sites (TES), and 2-kb windows surrounding TSSs (TSS2kb) in hg19 and hg38 were RefSeq annotations included in ChromHMM (v1.18) and originally based on annotations obtained from the UCSC genome browser on July 26, 2015.
- Lamina-associated domains were for human embryonic lung fibroblasts that were included in ChromHMM (1.18), which were lifted over to hg19 from hg18 positions originally provided by [39].
- Annotations of assembly gaps in hg19 and hg38 were obtained from the UCSC genome browser and correspond to the Gap track.
- Coordinates of ZNF named genes correspond to non-overlapping coordinates from GENCODE’s hg19 gene annotation, v30 [83]. ZNF named genes were those whose gene named contained “ZNF.” The list of C2H2-type genes was from https://www.genenames.org/.
- Annotations of coding sequences in hg19 and hg38 correspond to coordinates of genes whose feature type is “CDS” from GENCODE’s hg19 and hg38 gene annotation, v30 [83], respectively.
- Annotations of pseudogenes in hg19 and hg38 correspond to coordinates of genes whose gene type or transcript type contained “pseudogene” from GENCODE’s hg19 and hg38 gene annotation, v30 [83], respectively.
- Annotations of repeat elements were obtained from UCSC genome browser RepeatMasker hg19 tracks.
- Concatenated ChromHMM chromatin state annotations were obtained from the Roadmap Epigenomics Consortium through http://compbio.mit.edu/roadmap [16]. These include data of the 15-state and 18-state models based on observed data and the 25-state chromatin model based on imputed data for 127, 98 and 127 reference epigenomes, respectively.
- CTCF-concatenated chromatin states were based on the ChromHMM chromatin state annotations for six human cell types (GM12878, H1ESC, Helas3, Hepg2, Huvec, K562) for a 25-state model from the ENCODE integrative analysis [22, 31]. We extracted coordinates of regions annotated to the “Ctcf” and “CtcfO,” both associated with CTCF signal and limited histone mark signal.
- Blacklisted regions were those provided by the ENCODE Data Analysis Center (DAC) for hg19 and hg38 [82].
- ConsHMM conservation state annotations for human (hg19) were those from [30].
- Annotations of human genetic variants and their allele frequency were from GNOMAD v2.1.1 [46]. The dataset includes 229 million SNVs and 33 million indels from 15,708 genomes of unrelated individuals, which are aligned against the GRCh37/hg19 reference.
- GWAS catalog variants were obtained from the NHGRI-EBI Catalog, accessed on December 5, 2016 [65].
- Coordinates of CpG sites profiled across cell types were obtained from DNA Methylation data in Roadmap Epigenomic portal.
- Data of G/C content at 5 bp resolution from UCSC Genome Browser, file hg19.gc5Base.txt.gz.
- Data of binding regions of proteins of the polycomb repressive complexes were downloaded from the ENCODE portal [85]. Download links are listed in Additional File 9.

### Analysis of gene expression across states

To analyze the relationship between gene expression and the full-stack states, we downloaded gene expression data from the Roadmap Epigenomics Consortium [16]. Specifically, we downloaded a matrix of gene expression values, in RPKM (reads per kilobase million), for protein coding genes for 56 reference epigenomes that were among the 127 used as part of the full-stack model. In total, we obtained expression values for 19,795 Ensembl protein coding genes.

The gene expression data was obtained from https://egg2.wustl.edu/roadmap/data/byDataType/rna/expression/57epigenomes.exon.RPKM.pc.gz. We also obtained the corresponding genomic coordinates for these genes from https://egg2.wustl.edu/roadmap/data/byDataType/rna/expression/Ensembl_v65.Gencode_v10.ENSG.gene_info. For this analysis, we filtered out genes that are not classified as protein coding. We transformed the gene expression values by adding a pseudo-count of 1 to the raw counts in RPKM, and taking the log of the resulting values.

For each full-stack state and 56 reference epigenomes, we calculated the average gene expression of all genes overlapping with the state, taking into account the genes’ length. For each gene *g*, we denote its length *L*_*g*_ and expression *E*_*g*_. We let *s*_*i*_ denote the state assigned at the 200-bp bin *i* and *G*_*i*_ denote the set of genes overlapping the 200-bp bin *i*. Let *B*_*s*_ denote the set of 200 bp bins that are assigned to state *s*. The average normalized expression with state *s* then becomes:

$$ avgexp\  bp\ {normalized}_s=\frac{\sum_{i\in {B}_s}{\sum}_{g\in {G}_i}\frac{E_g}{L_g}}{\sum_{i\in {B}_s}{\sum}_{g\in {G}_i}\frac{1}{L_g}} $$

We also calculated for each state the average and coefficient of variation of these averages across reference epigenomes. We used the BEDTools [86] *bedtools intersect* command to obtain the chromatin state assignments for 200-bp segments that totally or partially overlap with any gene. To obtain average gene expressions of a state in a cell type group as presented in Fig. 3C, we averaged the reported bp-normalized average gene expressions of the corresponding state across cell types within the group.

We also analyzed average gene expression values for each state as a function of the position of the state annotations relative to TSS, following a procedure similar to what was used previously [3]. We first identified a gene’s outer transcription start site (TSS) based on the reported coordinates of the gene and strand in the gene annotation file noted above. For each 200-bp bin that is within 25 kb upstream or downstream of an annotated TSS, including those that directly overlap with an annotated TSS, we determined the assigned full-stack state at this bin, and the position of the bin relative to those TSSs. Bins directly overlapping an annotated TSS were at position 0. If the gene was on the positive strand, the segments’ genomic coordinates lower than the TSSs’ correspond to upstream regions at negative points (minimum value −25,000), while genomic coordinates higher than the TSSs’ correspond to downstream regions at positive points (maximum value 25,000). If the gene is on the negative strand, the upstream and downstream positions are reversed. For each state and each 200-bp bin position relative to TSS, we determined the subset of genes where there is a 200-bp bin annotated to that state at that position relative to their TSSs and calculated their average expression. This produces a 100-by-251 table for one reference epigenome, corresponding to the number of full-stack states and 200-bp segments intersecting the 50-kb windows surrounding genes’ TSSs and one segment directly overlapping the TSSs. We then smoothed the averaged expression data spatially by applying a sliding window with a window size of 21, i.e., each segment’s smoothed gene expression is the average of data in that segment and 20 surrounding genomic segments. Data of average gene expression in the first and last 10 segments within the 50-kb window are not included in the window of smoothed data. We averaged results of 56 tables corresponding to 56 reference epigenomes as the final output from this procedure.

### Computing average DNA methylation levels

The DNA methylation analysis was conducted based on Whole Genome Bisulfite Sequencing data from Roadmap Epigenomics [16]. The fraction DNA methylation values was obtained from https://egg2.wustl.edu/roadmap/data/byDataType/dnamethylation/WGBS/FractionalMethylation.tar.gz. For each combination of 37 reference epigenomes with DNA methylation available and 100-full-stack states, the average fractional DNA methylation in that reference epigenome was computed for all CpG bases with a non-missing DNA methylation value overlapping the full-stack state annotation.

### Computing enrichment for bases prioritized by variant prioritization scores

To compute state enrichments for bases prioritized by different variant prioritization scores, we followed the approach of [30]. We obtained coordinates of bases containing prioritized variants based on 14 different methods as processed and described in [30]. The scores were Eigen and Eigen-PC version 1.1, funSeq2 version 2.1.6, CADD v1.4, REMM, FIRE, fitCons, CDTS, LINSIGHT, FATHMM-XF, GERP++, PhastCons, phyloP, and DANN [51, 53–64]. For 12 of the 14 scores, we separately considered prioritized variants genome-wide and in non-coding regions only. Two of the variant prioritization scores, LINSIGHT and FunSeq2, were defined only in the non-coding regions, so these scores were only used in the non-coding region analysis. As described in [30], the regions included in the non-coding analysis were defined as the bases where both LINSIGHT and FunSeq2 provided scores, which was 90.4% of the genome. For both the non-coding and whole-genome analysis, we computed the enrichment for bases ranked in the top 1%, 5%, or 10% using the variant prioritization scores. We note that because of ties in some scores, the score-threshold above which we classified the bases as prioritized was chosen to be as close as possible to the target percentage (1%, 5%, or 10%). We also note that if there were any bases with missing values for any particular score, then that base was assigned with the minimum values of such scores.

Enrichment values for the whole genome were computed as described above with the OverlapEnrichment command from ChromHMM. For computing enrichments restricted to non-coding regions, we first calculated enrichment of the non-coding prioritized variants relative to the whole genome and the enrichment of non-coding regions as defined above relative to the whole genome. We then divided these two enrichment values to obtain the enrichment of prioritized non-coding variants within non-coding regions.

### Data availability

Full-stack chromatin state annotation of the genome are available at https://github.com/ernstlab/full_stack_ChromHMM_annotations. The code used to analyze the output of ChromHMM and characterize the states is provided under the open source MIT license at https://github.com/ernstlab/full_stack_ChromHMM_annotations [87]. An archival version of this code is available at 10.5281/zenodo.5759119 [88] under the MIT license. The software ChromHMM version 1.18 is available under the GPL 3 license at https://github.com/jernst98/ChromHMM/releases/tag/v1.18 [89]. The most up-to-date version of ChromHMM is available at https://ernstlab.biolchem.ucla.edu/ChromHMM/. All links to download publicly available data for analyses in this paper are listed in Additional File 9.

## Supplementary Information

**Additional file 1.** Supplementary figures S1–S50.

**Additional file 2.** GO terms associated with each full-stack state. Each figure shows the 5 GO Biological Process and 5 GO Molecular Function terms most significantly enriched in each full-stack state. State Quies3 did not have a list of enriched GO terms because there was no output from GREAT for regions associated with state. Therefore, there are 99 figures corresponding to 99 full-stack states with output from GREAT.

**Additional file 3.** Summary characterizations of full-stack states. Full characterization of the full-stack states, with detailed comments summarizing states’ characteristics. Emission parameters of the full-stack states. Top 100 highly emitted datasets associated with each full-stack state, colored by the marks associated with the datasets (similar to Fig. 2B). Top 100 highly emitted datasets associated with each full-stack state, colored by the cell groups associated with the datasets (similar to Fig. 2C). Different genome context enrichments with full-stack states: Excel version of Fig. 3A-B, Additional File 1: Figure S18. Excel version of Additional File 1 Figure S31: Enrichment of all full-stack states for ConsHMM states. Excel version of Additional File 1 Figure S30: Full-stack states and maximum-enriched ConsHMM state. Excel version of Additional File 1 Figure S7: Statistically significant tissue—group specificity in full-stack states.

**Additional file 4.** Full-stack states enrichments with repeats, prioritized variants, common variants, structural variants, CTCF, PRC1-PRC2, GWAS catalog variants, sex chromosomes, fine-mapped variants, cancer-associated variants. Excel version of Additional File 1: Figure S28: Full stack states enrichments with RepeatMasker classes of repeats and CG content. Excel version of Additional File 1: Figure S37: Enrichment of all full-stack states for top 1% bases prioritized by variant prioritization scores. Excel version of Additional File 1: Figure S32: Full-stack states enrichments with structural variants. Excel version of Additional File 1: Figure S42: Full-stack states enrichments with variants from GNOMAD stratified by minor allele frequencies, common variants and CG dinucleotides. Excel version of Additional File 1: Figure S14: Full-stack states enrichments with CTCF associated chromatin states. Excel version of Additional File 1: Figure S17B: Neighborhood enrichments of full-stack states with binding sites of PRC1 and PRC2 complexes. Excel version of Additional File 1: Figure S16: Full-stack states enrichments with Polycomb Repressive protein complexes PRC1 and PRC2. Excel version of Additional File 1: Figure S43: Full-stack states enrichments with GWAS catalog variants and sex chromosomes. Excel version of Additional File 1: Figure S44: Full-stack states enrichment values for fine-mapped variants at phenotype associated loci. Excel version of Additional File 1: Figure S47: Full-stack states enrichments with cancer-associated somatic mutations in the non-coding genome.

**Additional file 5 **Full stack states’ association with concatenated annotations for multiple cell types. Excel version of Additional File 1: Figure S9: Estimated probabilities of concatenated chromatin states overlapping with full-stack states. *Detailed comments about full-stack states’ characteristics through this analysis are provided.* Excel version of Additional File 1: Figure S8: Full-stack state maximum enrichments with annotated chromatin states in 127 reference epigenomes. *Detailed comments about full-stack states’ characteristics through this analysis are provided.* Maximum enrichments of full-stack states and 25-state concatenated annotations for 127 cell/tissue types (reference epigenomes). Median enrichments of full-stack states and 25-state concatenated annotations for 127 cell/tissue types (reference epigenomes).

**Additional file 6.** Data of AUROC comparison between full-stack annotation and concatenated and independent annotations in predicting different genomic contexts. Data accompanying Additional File 1: Figure S19-21: AUROC comparison of full-stack annotations and concatenated and independent annotations in predicting external genome contexts. Data accompanying Additional File 1: Figure S34-35: AUROC comparison of full-stack model annotations and 18-state concatenated annotations and 100-state independent annotations in predicting structural variants of type deletions and duplications. Data accompanying Additional File 1: Figure S29: AUROC comparison of the full-stack and concatenated and independent chromatin state annotations at predicting different classes of repeat elements. Data accompanying Additional File 1: Figure S45-46: AUROC comparison of full-stack model annotations and the 100-state independent annotations and 18-state concatenated annotations in predicting fine-mapped variants. Data accompanying Additional File 1: Figure S41: AUROC comparison of the full-stack model annotations and concatenated and independent model annotations at predicting top 1% non-coding bases prioritized by various variant prioritization scores.

**Additional file 7.** Summary characteristics of ConsHMM states. Descriptions of select ConsHMM states mentioned in the main text. Excel version of Additional File 1: Figure S30C: Enrichment of all full-stack states for ConsHMM states.

**Additional file 8.** Supplementary Information about the full-stack model analysis. Naïve-Bayes greedy search for representative datasets for characterization of the full-stack states. Asymptotic worst-case time and memory usage of stacked model.

**Additional file 9.** Download links for annotation data used throughout the manuscript.

**Additional file 10.** Peer review history.
