# Supplementary material for: Universal annotation of the human genome through integration of over a thousand epigenomic datasets
Source: Genome Biol. 2022 Jan 6;23:9. doi: 10.1186/s13059-021-02572-z (PMC8734071; doi:10.1186/s13059-021-02572-z)
Supplement: Supplementary file 1 — Additional file 1. Supplementary figures S1–S50. [file 13059_2021_2572_MOESM1_ESM.pdf]

Supplementary Figure File content

Fig. number	Figure Title
**S1**	Illustration of concatenated model training vs. stacked model training
**S2**	Mark and tissue group distribution of the input data tracks
**S3**	Evaluation of full-stack model's number of states
**S4**	Emission probabilities of 80 datasets chosen to summarize the full-stack model
**S5**	Full-stack states emission probabilities, averaged by chromatin marks
**S6**	Full-stack states transition probabilities
**S7**	Statistically significant tissue—group specificity in full-stack states
**S8**	Full-stack states maximum-enrichments with annotated concatenated-model chromatin states in 127 reference epigenomes
**S9**	Estimated probabilities of concatenated-model chromatin states overlapping with full-stack states.
**S10**	Full-stack states' average gene expression in different cell types.
**S11**	Full-stack states' average gene expression as a function of distance from TSS
**S12**	Positional enrichments of full-stack states around annotated transcription start sites and transcription end sites.
**S13**	Top GO terms for states in promoter-associated states.
**S14**	Full-stack states enrichments with CTCF associated chromatin states.
**S15**	Full-stack states' average DNA methylation in different cell types.
**S16**	Full-stack states enrichments with Polycomb Repressive protein complexes PRC1 and PRC2.
**S17**	Enrichments of selected full-stack states with PRC1 and PRC2.
**S18**	Comparison of hg19 and hg38 full-stack states enrichments with annotated genomic contexts
**S19**	ROC comparison of full-stack model annotations and the 18-state concatenated model annotations for predicting various external genomic annotations
**S20**	AUROC comparison of the full- stacked and the concatenated and independent chromatin state annotations at predicting various external genomics annotations.
**S21**	ROC comparison of full-stack model annotations and the 100-state independent model annotations for predicting various external genomic annotations.
**S22**	AUROC comparison of the full-stack and concatenated and independent chromatin state annotations at predicting CTCF-specific chromatin states.
**S23**	Coefficient of variations of emission probabilities across different cell groups.
**S24**	Illustration of the full-stack annotations at two distinct loci.
**S25**	Illustration of full-stack cell-type-specific enhancer states.
**S26**	Illustration of full-stack heterochromatin state HET9.
**S27**	Illustration of full-stack flanking promoter state PromF5.
**S28**	Full stack states enrichments with RepeatMasker classes of repeats, low-complexity repeats and GC content, simple repeats.
**S29**	AUROC comparison of the full-stack, concatenated and independent chromatin state annotations at predicting different classes of repeat elements.
**S30**	Full-stack states and maximum enriched ConsHMM state.
**S31**	Full-stack states enrichments with different subsets of ZNF genes.
**S32**	Full-stack states enrichments with structural variants.
**S33**	Comparison of full-stack model annotations and the 100-state independent model annotations in predicting structural variants of type deletions and duplications.
**S34**	Comparison of full-stack model annotations and 18-state concatenated model annotations in predicting structural variants of type deletions and duplications.
**S35**	Comparison of full-stack states vs. state-specific annotations in predicting structural variants of types deletions and duplications.
**S36**	Enrichment of selected full-stack states with prioritized variants, non-coding genome.
**S37**	Enrichment of all full-stack states for top 1% bases prioritized by variant prioritization scores.
**S38**	Enrichment of all full-stack states for top 5% bases prioritized by variant prioritization scores.
**S39**	Enrichment of all full-stack states for top 10% bases prioritized by variant prioritization scores.
**S40**	Enrichment of selected full-stack states with prioritized variants, whole genome.

**S41**	Comparison of the full-stack model annotations against the concatenated and independent model annotations at predicting top 1% non-coding bases prioritized by various variant prioritization scores.
**S42**	Full-stack states enrichments with variants from GNOMAD stratified by minor allele frequencies, common variants and CpG dinucleotides.
**S43**	Full-stack states enrichments with GWAS catalog variants and sex chromosomes.
**S44**	Full-stack states enrichment values for fine-mapped variants at phenotype associated loci.
**S45**	Comparison of full-stack model annotations and the 100-state annotations from independent models in predicting fine-mapped variants.
**S46**	Comparison of full-stack model annotations and the 18-state annotations from a concatenated model in predicting fine-mapped variants.
**S47**	Full-stack states enrichments with cancer-associated somatic mutations in the non-coding genome.
**S48**	Comparison of full-stack model annotation and the 100-state independent annotations in predicting somatic mutations associated with four cancer types from COSMIC database
**S49**	Comparison of full-stack model annotation and the 18-state concatenated annotations in predicting somatic mutations associated with four cancer types from COSMIC database
**S50**	Full-stack states enrichments of bases that were not lifted over from hg19 to hg38.

Note: We included references to previous publications in the legends to some supplementary figures. The full list of these references is included at the end of this document, and all of these references are already listed in the references list in the main text.

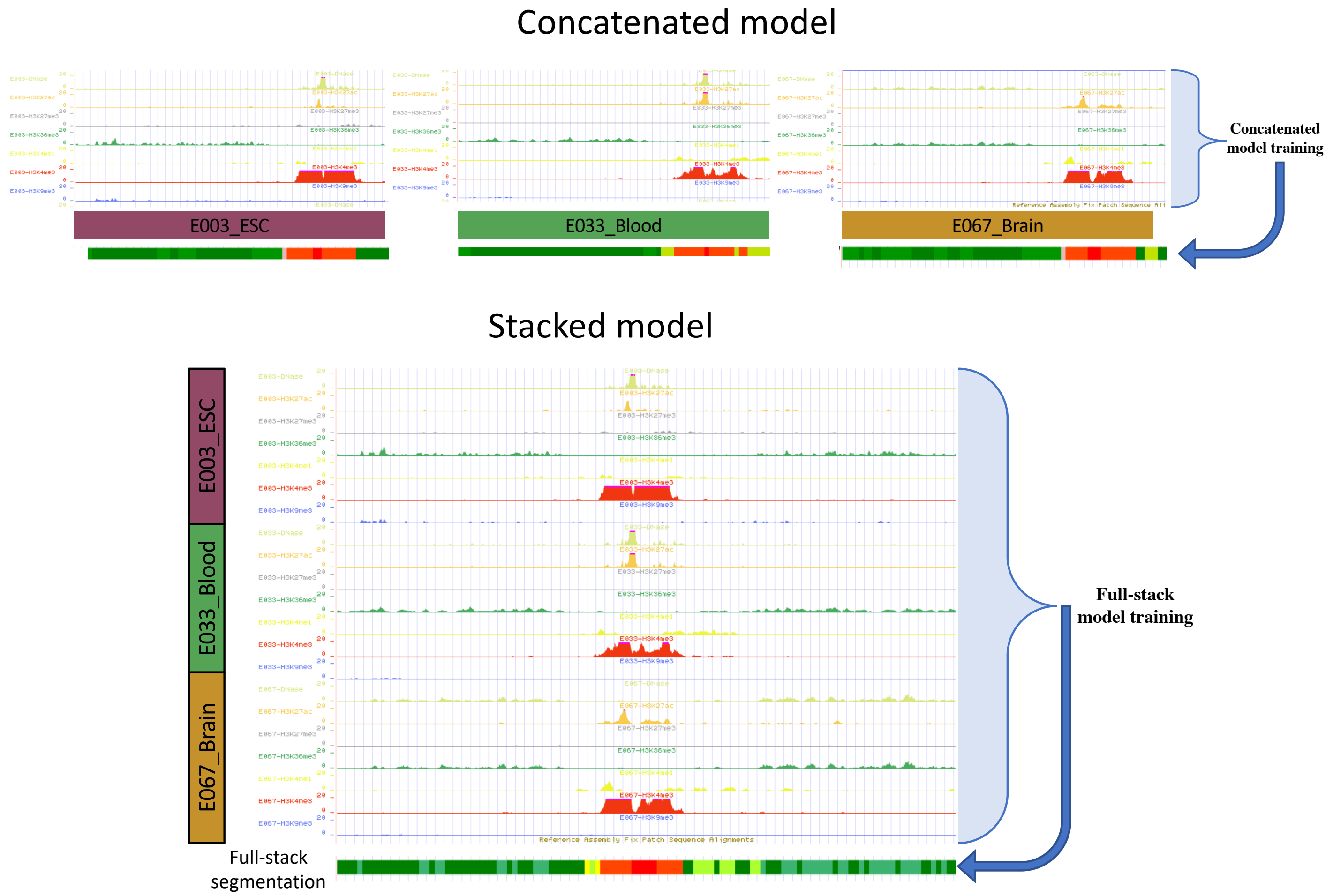

Figure S1: Illustration of concatenated model training vs. stacked model training. The top of the figure illustrates the concatenated modeling approach where a chromatin state annotation is produced for each cell type based on the data in that cell type using a common set of chromatin state definitions. In contrast, the stacked modeling approach produces a single chromatin annotation of the genome based on all the data.

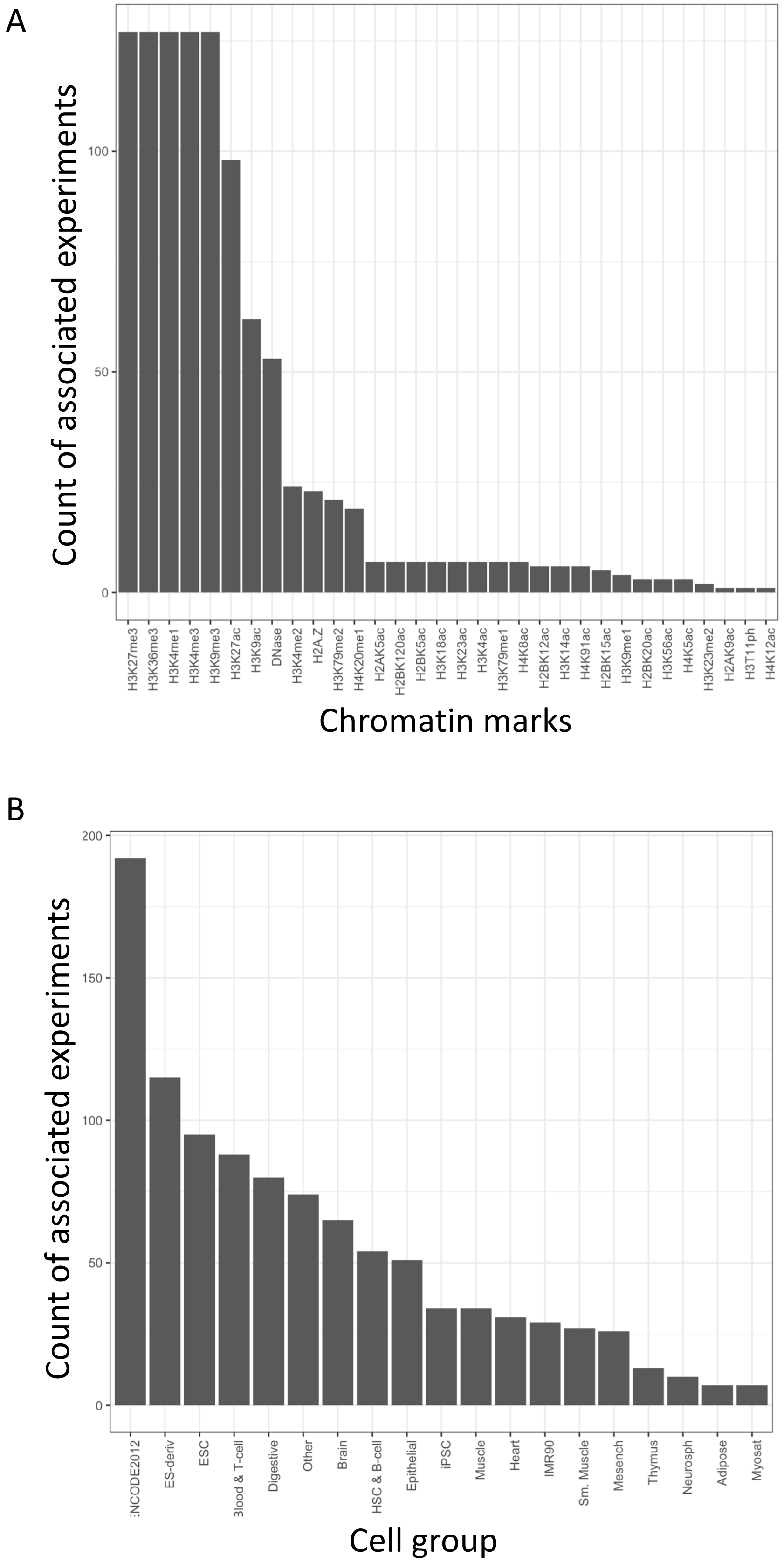

Figure S2: Mark and tissue group distribution of the input data tracks. **(A)** Counts of input tracks associated with different chromatin marks. There are five marks that were profiled in all 127 reference epigenomes, while some marks, largely acetylation marks, were profiled in few reference epigenomes. In total there were 1032 input tracks, including 53 DNase-seq datasets and 979 ChiP-seq datasets. **(B)** Count of input tracks associated with different tissue groups previously defined (Kundaje et al., 2015).

A

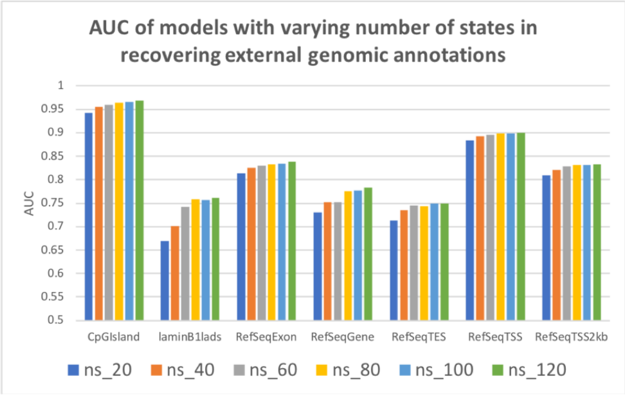

B

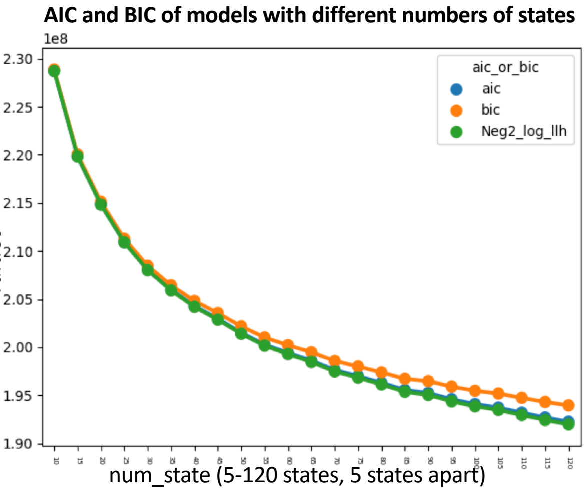

D

Max correlations of emission parameters associated with H3K4me1 with indicators of cell groups

Number of states

Tissue groups	10	15	20	25	30	35	40	45	50	55	60	65	70	75	80	85	90	95	100	105	110	115	120	max_corr
Adipose	0.17	0.18	0.18	0.18	0.18	0.17	0.17	0.17	0.17	0.18	0.19	0.18	0.18	0.18	0.19	0.18	0.18	0.18	0.19	0.21	0.19	0.19	0.19	0.21
Blood & T-cell	0.67	0.67	0.68	0.70	0.69	0.71	0.71	0.76	0.84	0.84	0.86	0.86	0.86	0.86	0.86	0.86	0.85	0.86	0.86	0.84	0.85	0.85	0.86	0.86
Brain	0.01	0.06	0.11	0.10	0.56	0.58	0.57	0.61	0.63	0.80	0.80	0.80	0.80	0.80	0.80	0.77	0.78	0.81	0.81	0.79	0.79	0.80	0.80	0.81
Digestive	-0.05	-0.02	0.07	0.06	0.23	0.33	0.45	0.55	0.54	0.50	0.61	0.54	0.55	0.63	0.64	0.65	0.64	0.65	0.64	0.64	0.64	0.65	0.64	0.65
ENCODE2012	0.39	0.44	0.43	0.45	0.45	0.45	0.46	0.44	0.45	0.44	0.47	0.49	0.48	0.48	0.48	0.50	0.52	0.50	0.52	0.49	0.49	0.52	0.53	0.53
Epithelial	0.34	0.34	0.35	0.36	0.38	0.38	0.38	0.45	0.45	0.45	0.46	0.45	0.45	0.48	0.47	0.48	0.48	0.47	0.48	0.48	0.49	0.49	0.49	0.49
ESC	0.38	0.40	0.62	0.62	0.67	0.67	0.65	0.67	0.68	0.69	0.68	0.69	0.68	0.68	0.68	0.68	0.70	0.70	0.72	0.70	0.71	0.72	0.71	0.72
ES-deriv	0.17	0.32	0.34	0.29	0.29	0.34	0.41	0.45	0.42	0.38	0.42	0.46	0.41	0.43	0.42	0.40	0.43	0.43	0.45	0.43	0.43	0.48	0.45	0.48
Heart	0.13	0.12	0.22	0.20	0.20	0.24	0.24	0.25	0.27	0.28	0.32	0.35	0.36	0.34	0.35	0.37	0.33	0.40	0.39	0.29	0.41	0.39	0.42	0.42
HSC & B-cell	0.52	0.57	0.55	0.57	0.58	0.61	0.60	0.74	0.78	0.75	0.80	0.80	0.80	0.79	0.80	0.79	0.71	0.80	0.65	0.66	0.81	0.79	0.76	0.81
IMR90	0.19	0.19	0.20	0.47	0.48	0.46	0.50	0.52	0.51	0.55	0.52	0.57	0.57	0.58	0.56	0.56	0.60	0.56	0.63	0.56	0.67	0.60	0.62	0.67
iPSC	0.37	0.32	0.44	0.45	0.48	0.48	0.48	0.48	0.49	0.48	0.48	0.48	0.48	0.48	0.48	0.50	0.51	0.52	0.51	0.51	0.51	0.51	0.52	0.52
Mesench	0.45	0.48	0.49	0.48	0.49	0.58	0.53	0.58	0.68	0.62	0.69	0.68	0.71	0.74	0.72	0.65	0.69	0.62	0.76	0.75	0.73	0.77	0.68	0.77
Muscle	0.28	0.28	0.41	0.37	0.35	0.40	0.43	0.41	0.40	0.53	0.50	0.55	0.53	0.55	0.50	0.54	0.52	0.63	0.53	0.54	0.53	0.63	0.60	0.63
Myosat	0.24	0.28	0.28	0.27	0.27	0.33	0.32	0.33	0.32	0.35	0.33	0.34	0.34	0.33	0.36	0.37	0.36	0.38	0.36	0.34	0.33	0.36	0.36	0.38
Neurosph	0.12	0.16	0.15	0.17	0.25	0.28	0.28	0.28	0.29	0.27	0.26	0.27	0.36	0.32	0.31	0.32	0.29	0.47	0.40	0.37	0.34	0.38	0.46	0.47
Other	0.15	0.17	0.17	0.18	0.19	0.23	0.23	0.23	0.24	0.29	0.28	0.29	0.29	0.37	0.35	0.36	0.31	0.29	0.32	0.42	0.45	0.32	0.38	0.45
Sm. Muscle	0.05	0.07	0.06	0.07	0.11	0.10	0.11	0.13	0.11	0.21	0.21	0.20	0.20	0.20	0.21	0.22	0.22	0.23	0.23	0.22	0.23	0.24	0.23	0.24
Thymus	0.06	0.13	0.13	0.13	0.13	0.16	0.16	0.17	0.16	0.17	0.17	0.17	0.17	0.17	0.16	0.18	0.19	0.18	0.20	0.21	0.21	0.22	0.22	0.22

C

Maximum correlations of emission parameters of full-stack states with any state in other models

Number of states

	10	15	20	25	30	35	40	45	50	55	60	65	70	75	80	85	90	95	100	105	110	115	120
GapArtf1	0.50	0.52	0.50	0.51	0.56	0.53	0.54	0.56	0.57	0.61	0.59	0.60	0.60	0.98	0.99	1.00	1.00	1.00	1.00	0.87	0.90	0.97	0.95
GapArtf2	0.69	0.70	0.69	0.70	0.71	0.73	0.94	0.93	0.93	0.93	0.92	0.88	0.89	0.86	0.86	0.85	1.00	1.00	1.00	1.00	1.00	1.00	1.00
GapArtf3	0.43	0.45	0.43	0.43	0.44	0.48	0.89	0.92	0.93	0.93	0.94	0.97	0.96	0.98	0.98	0.98	1.00	1.00	1.00	1.00	1.00	1.00	1.00
Quies1	0.78	0.79	0.81	0.87	0.80	0.77	0.83	0.83	0.95	0.94	0.96	0.95	0.96	1.00	1.00	1.00	1.00	1.00	1.00	0.95	0.96	1.00	0.98
Quies2	0.75	0.70	0.79	0.74	0.96	0.84	0.91	0.99	0.99	0.94	0.99	0.93	0.93	1.00	1.00	1.00	1.00	1.00	1.00	0.96	0.97	0.99	0.97
Quies3	0.59	0.55	0.59	0.60	0.60	0.68	0.77	0.58	0.78	0.90	0.84	0.84	0.96	0.88	0.86	0.95	0.98	0.96	1.00	0.84	0.98	0.97	0.78
Quies4	0.80	0.82	0.81	0.85	0.96	0.91	0.93	0.99	0.99	0.98	0.98	1.00	1.00	0.99	0.99	0.99	1.00	1.00	1.00	0.95	0.90	0.90	1.00
Quies5	0.30	0.32	0.34	0.35	0.35	0.35	0.37	0.93	0.93	0.37	0.93	0.67	0.94	0.92	0.79	0.93	0.99	1.00	1.00	0.98	1.00	1.00	0.99
HET1	0.65	0.72	0.74	0.67	0.95	0.97	0.97	0.94	0.94	0.98	0.94	0.99	0.98	0.97	1.00	0.99	1.00	1.00	1.00	0.95	0.96	1.00	0.97
HET2	0.43	0.47	0.57	0.69	0.81	0.79	0.79	0.88	0.88	0.81	0.90	0.99	0.78	0.86	1.00	1.00	1.00	1.00	1.00	1.00	1.00	0.99	1.00
HET3	0.81	0.80	0.82	0.83	0.90	0.91	0.91	0.95	0.95	0.96	0.96	0.99	0.94	1.00	1.00	1.00	1.00	1.00	1.00	0.99	0.98	1.00	1.00
HET4	0.89	0.88	0.91	0.91	0.94	0.97	0.96	0.99	1.00	0.99	0.99	0.98	1.00	1.00	1.00	1.00	1.00	1.00	1.00	1.00	1.00	1.00	1.00
HET5	0.96	0.98	0.96	0.96	0.97	0.99	0.98	0.99	0.99	0.99	1.00	0.99	1.00	0.99	0.98	0.98	1.00	1.00	1.00	1.00	1.00	1.00	1.00
HET6	0.95	0.91	0.95	0.94	0.93	0.94	0.92	0.98	0.99	0.99	0.99	0.99	1.00	0.99	0.99	0.99	1.00	1.00	1.00	0.98	0.97	1.00	1.00
HET7	0.86	0.84	0.85	0.84	0.82	0.83	0.87	0.95	0.95	0.93	0.94	0.92	0.92	0.91	0.90	0.90	0.92	1.00	1.00	1.00	0.99	1.00	1.00
HET8	0.87	0.83	0.88	0.87	0.86	0.87	0.85	0.88	0.87	0.85	0.89	0.87	1.00	0.99	0.99	0.99	0.99	0.99	1.00	0.95	0.94	0.95	0.97
HET9	0.72	0.75	0.73	0.77	0.87	0.83	0.85	0.88	0.87	0.88	0.88	0.98	0.87	0.94	0.96	0.96	0.99	1.00	1.00	0.99	0.98	0.97	1.00
ReprPC1	0.88	0.97	0.97	0.97	0.96	0.96	0.99	0.98	0.96	0.97	0.97	1.00	1.00	0.99	0.99	0.98	0.98	1.00	1.00	1.00	1.00	1.00	1.00
ReprPC2	0.81	0.91	0.94	0.90	0.91	0.99	0.95	0.99	0.99	0.99	0.98	0.99	0.98	0.98	0.99	0.98	0.98	1.00	1.00	1.00	1.00	1.00	1.00
ReprPC3	0.75	0.87	0.95	0.95	0.96	0.95	0.94	0.96	0.96	0.97	0.97	0.98	0.96	0.97	0.98	0.98	0.97	0.99	1.00	1.00	0.99	0.98	0.99
ReprPC4	0.66	0.83	0.92	0.95	0.95	0.95	0.96	0.96	0.93	0.95	0.94	0.97	0.99	0.98	0.99	0.99	1.00	1.00	1.00	1.00	0.99	1.00	1.00
ReprPC5	0.89	0.96	0.89	0.96	0.94	0.93	0.91	0.95	0.94	0.96	0.96	0.95	0.96	0.94	0.96	0.99	0.99	1.00	1.00	0.98	1.00	1.00	1.00
ReprPC6	0.78	0.91	0.81	0.87	0.81	0.96	0.92	0.96	0.95	0.98	0.97	0.98	0.92	0.98	0.98	0.94	0.93	0.95	1.00	0.99	0.97	0.97	0.95
ReprPC7	0.73	0.75	0.90	0.73	0.86	0.92	0.95	0.91	0.92	0.91	0.88	0.88	0.92	0.89	0.89	0.95	0.96	0.93	1.00	0.89	0.95	0.98	0.98
ReprPC8	0.42	0.49	0.50	0.51	0.51	0.51	0.52	0.98	0.98	0.51	0.98	0.68	0.98	0.99	0.79	0.98	1.00	1.00	1.00	1.00	1.00	1.00	1.00
ReprPC9	0.50	0.57	0.59	0.56	0.55	0.67	0.61	0.69	0.66	0.68	0.64	0.82	0.82	0.72	0.72	0.95	0.94	1.00	1.00	1.00	1.00	1.00	1.00
Acet1	0.46	0.50	0.70	0.84	0.77	0.70	0.79	0.76	0.78	0.88	0.83	0.89	0.96	0.99	0.95	0.93	0.95	0.90	1.00	0.98	0.98	0.99	0.97
Acet2	0.61	0.69	0.79	0.84	0.74	0.79	0.81	0.77	0.80	0.90	0.88	0.91	0.92	0.92	0.93	0.98	0.97	0.95	1.00	0.99	1.00	1.00	1.00
Acet3	0.60	0.58	0.72	0.77	0.71	0.81	0.81	0.79	0.84	0.97	0.92	0.96	0.98	0.97	0.95	0.97	0.96	0.97	1.00	0.99	0.99	0.98	0.98
Acet4	0.74	0.65	0.67	0.93	0.91	0.90	0.94	0.91	0.93	0.87	0.88	0.91	0.94	0.93	0.98	0.96	0.90	0.98	1.00	0.99	1.00	0.99	0.98
Acet5	0.59	0.59	0.63	0.96	0.97	0.96	0.95	0.97	0.96	0.99	0.98	1.00	1.00	1.00	1.00	1.00	1.00	1.00	1.00	1.00	1.00	1.00	1.00
Acet6	0.85	0.90	0.91	0.90	0.90	0.92	0.90	0.89	0.90	0.96	0.93	0.95	0.92	0.93	0.95	0.89	0.96	0.88	1.00	0.98	1.00	0.98	1.00
Acet7	0.94	0.92	0.94	0.92	0.94	0.94	0.97	0.97	0.97	0.96	0.95	0.93	0.92	0.91	0.96	0.94	0.99	0.99	1.00	1.00	0.92	0.99	0.98
Acet8	0.39	0.61	0.63	0.64	0.73	0.88	0.82	0.87	0.87	0.92	0.89	0.94	0.96	0.94	1.00	1.00	1.00	1.00	1.00	1.00	1.00	1.00	1.00
EnhWk1	0.68	0.74	0.74	0.72	0.74	0.82	0.82	0.86	0.91	0.86	0.92	0.90	0.97	0.99	0.95	0.93	0.88	0.91	1.00	0.99	0.99	0.95	0.97
EnhWk2	0.67	0.76	0.85	0.88	0.85	0.93	0.96	0.92	0.90	0.84	0.93	0.95	0.97	0.94	0.99	0.89	0.90	0.94	1.00	0.86	0.95	0.88	0.91
EnhWk3	0.55	0.57	0.58	0.65	0.77	0.75	0.76	0.72	0.70	0.99	0.99	0.99	0.99	0.99	0.99	1.00	1.00	0.93	1.00	1.00	1.00	0.98	0.98
EnhWk4	0.38	0.50	0.53	0.55	0.68	0.71	0.72	0.70	0.70	0.66	0.74	0.58	0.97	0.91	0.88	0.71	0.64	0.89	1.00	0.99	0.98	0.98	0.93
EnhWk5	0.40	0.74	0.74	0.75	0.78	0.79	0.79	0.76	0.73	0.74	0.73	0.73	0.73	0.74	0.74	0.74	1.00	0.74	1.00	0.99	1.00	1.00	0.76
EnhWk6	0.54	0.91	0.91	0.93	0.94	0.87	0.87	0.97	0.99	0.99	1.00	1.00	1.00	1.00	0.99	1.00	1.00	0.99	1.00	1.00	1.00	1.00	1.00
EnhWk7	0.27	0.46	0.46	0.46	0.47	0.52	0.52	0.57	0.57	0.58	0.58	0.59	0.59	0.60	0.59	0.60	1.00	0.59	1.00	1.00	1.00	1.00	1.00
EnhWk8	0.34	0.40	0.86	0.91	0.94	0.99	0.98	0.99	0.99	0.99	0.94	0.99	0.99	0.99	0.98	0.98	0.97	1.00	1.00	1.00	1.00	1.00	0.99
EnhA1	0.90	0.92	0.94	0.96	0.98	0.97	0.95	0.97	0.97	0.98	0.99	1.00	0.99	0.99	1.00	1.00	0.99	1.00	1.00	1.00	0.96	1.00	0.98
EnhA2	0.82	0.79	0.87	0.81	0.82	0.80	0.82	0.81	0.81	0.85	0.87	0.85	0.86	0.86	0.84	0.99	0.99	0.98	1.00	0.98	1.00	0.98	0.99
EnhA3	0.84	0.86	0.91	0.91	0.89	0.89	0.89	0.87	0.88	0.96	0.87	0.94	0.92	0.91	0.95	0.92	0.90	0.99	1.00	0.97	0.95	0.97	0.91
EnhA4	0.85	0.93	0.91	0.95	0.94	0.94	0.97	0.94	0.96	0.94	0.94	0.97	0.97	0.97	0.93	0.98	1.00	0.98	1.00	0.96	0.95	0.95	0.99
EnhA5	0.77	0.85	0.84	0.86	0.93	0.97	0.96	0.97	0.96	0.99	0.98	0.98	0.98	0.98	1.00	0.98	0.99	0.99	1.00	0.96	0.99	0.96	0.98
EnhA6	0.27	0.35	0.37	0.37	0.81	0.82	0.81	0.85	0.87	1.00	1.00	1.00	1.00	1.00	1.00	0.98	0.99	0.99	1.00	0.99	0.99	1.00	1.00
EnhA7	0.54	0.81	0.81	0.80	0.82	0.84	0.83	0.79	0.77	0.75	0.76	0.77	0.78	0.79	0.76	0.73	0.83	0.73	1.00	0.99	0.78	0.97	0.98
EnhA8	0.45	0.94	0.94	0.94	0.90	0.97	0.98	0.95	0.93	0.97	0.97	0.98	0.98	0.99	0.96	0.99	0.99	1.00	1.00	0.99	1.00	1.00	1.00
EnhA9	0.84	0.80	0.88	0.80	0.91	0.88	0.88	0.95	0.96	0.92	0.91	0.91	0.95	0.95	0.97	0.99	0.97	1.00	1.00	0.97	0.99	0.99	0.98
EnhA10	0.74	0.83	0.83	0.81	0.89	0.82	0.81	0.83	0.89	0.83	0.84	0.85	0.91	0.92	0.86	0.98	0.93	0.97	1.00	0.97	0.89	0.92	0.94
EnhA11	0.25	0.50	0.50	0.54	0.53	0.63	0.63	0.83	0.80	0.84	0.85	0.85	0.86	0.90	0.88	0.91	0.98	1.00	1.00	1.00	0.99	0.99	0.95
EnhA12	0.65	0.58	0.59	0.67	0.67	0.72	0.65	0.92	0.92	0.90	0.90	0.89	0.89	1.00	1.00	1.00	1.00	1.00	1.00	1.00	1.00	1.00	0.99
EnhA13	0.43	0.42	0.46	0.58	0.62	0.68	0.62	0.97	0.98	0.99	0.99	1.00	1.00	1.00	1.00	1.00	1.00	1.00	1.00	1.00	1.00	1.00	1.00
EnhA14	0.44	0.44	0.51	0.58	0.58	0.81	0.92	0.96	0.94	0.91	0.99	0.95	0.96	1.00	1.00	1.00	1.00	1.00	1.00	1.00	1.00	1.00	0.99
EnhA15	0.30	0.38	0.46	0.40	0.47	0.73	0.82	0.95	0.97	0.99	0.95	0.99	0.99	1.00	0.99	0.98	1.00	1.00	1.00	0.96	0.96	0.95	0.94
EnhA16	0.25	0.39	0.43	0.44	0.45	0.42	0.44	0.33	0.30	0.36	0.49	0.86	0.88	0.94	0.88	0.87	0.87	0.89	1.00	0.86	0.86	0.85	0.86
EnhA17	0.43	0.64	0.93	0.93	0.90	0.92	0.91	0.91	0.92	0.92	0.89	0.91	0.90	0.91	0.91	0.91	0.99	0.91	1.00	1.00	1.00	1.00	0.95
EnhA18	0.39	0.54	0.88	0.88	0.94	0.94	0.90	0.94	0.96	0.98	0.94	0.98	0.97	0.97	0.97	0.96	0.97	0.97	1.00	0.97	0.98	1.00	0.98
EnhA19	0.46	0.80	0.86	0.79	0.80	0.96	0.98	0.96	0.95	0.90	1.00	0.97	0.93	0.91	0.92	0.94	0.93	0.96	1.00	1.00	0.99	0.99	0.92
EnhA20	0.31	0.34	0.35	0.36	0.37	0.39	0.38	0.40	0.40	0.41	0.45	0.41	0.44	0.48	0.46	0.40	0.71	0.53	1.00	1.00	1.00	1.00	1.00
TxEnh1	0.70	0.79	0.79	0.78	0.82	0.83	0.81	0.78	0.82	0.81	0.84	0.82	0.85	0.85	0.88	0.96	0.90	0.99	1.00	0.84	0.98	0.95	0.97
TxEnh2	0.95	0.88	0.90	0.87	0.95	0.95	0.95	0.93	0.99	0.96	0.95	0.98	0.97	0.95	0.99	0.96	0.94	1.00	1.00	0.98	0.99	0.98	0.99
TxEnh3	0.82	0.88	0.86	0.87	0.86	0.86	0.86	0.90	0.85	0.91	0.87	0.89	0.89	0.90	0.90	0.87	0.90	0.89	1.00	0.97	0.89	0.89	0.99
TxEnh4	0.96	0.97	0.97	0.97	0.96	0.96	0.96	0.97	0.96	0.97	0.99	0.98	0.97	0.99	0.98	0.98	0.98	0.98	1.00	0.98	0.97	0.99	0.98
TxEnh5	0.86	0.86	0.86	0.99	0.99	0.99	0.99	0.99	0.96	0.94	0.94	0.94	0.99	0.98	0.97	0.99	0.99	1.00	1.00	1.00	0.99	1.00	0.98
TxEnh6	0.69	0.70	0.70	0.69	0.79	0.78	0.78	0.79	0.79	0.79	0.78	0.78	0.74	0.78	0.94	0.77	0.78	1.00	1.00	0.79	1.00	1.00	1.00
TxEnh7	0.84	0.89	0.89	0.87	0.83	0.82	0.83	0.99	0.97	0.92	0.94	0.91	0.89	0.88	0.88	0.98	0.94	0.97	1.00	0.97	0.92	0.96	0.93
TxEnh8	0.66	0.67	0.67	0.71	0.84	0.84	0.84	0.79	0.84	0.91	0.93	0.92	0.97	0.92	0.93	1.00	0.93	0.99	1.00	0.99	0.93	0.97	0.97
TxWk1	0.96	0.97	0.93	0.96	0.99	0.99	0.97	0.98	0.97	1.00	0.97	0.99	1.00	0.92	0.99	1.00	0.99	1.00	1.00	0.99	0.99	1.00	0.94
TxWk2	0.71	0.71	0.71	0.88	0.88	0.88	0.87	0.88	0.95	0.98	0.98	0.97	0.99	0.91	0.99	1.00	0.99	1.00	1.00	0.99	0.98	1.00	0.96
Tx1	0.90	0.97	0.98	0.99	0.99	0.99	0.99	0.98	0.99	0.98	0.97	0.98	1.00	0.99	0.99	1.00	1.00	1.00	1.00	1.00	1.00	0.99	1.00
Tx2	0.84	0.95	0.94	0.96	0.98	0.98	0.98	0.99	0.98	0.99	0.99	0.99	1.00	1.00	0.97	1.00	1.00	1.00	1.00	1.00	1.00	1.00	1.00
Tx3	0.77	0.86	0.83	0.83	0.86	0.86	0.85	0.86	0.87	0.86	0.97	0.98	0.97	0.96	0.96	1.00	0.99	1.00	1.00	0.98	0.99	1.00	0.99
Tx4	0.79	0.77	0.92	0.86	0.80	0.80	0.89	0.85	0.99	0.97	0.99	0.99	0.99	0.99	0.99	0.99	0.99	0.99	1.00	0.99	0.99	0.99	0.99
Tx5	0.94	0.94	0.94	0.91	0.93	0.93	0.93	0.92	0.97	0.96	0.97	0.97	0.99	0.97	1.00	1.00	1.00	1.00	1.00	0.99	0.99	0.99	0.99
Tx6	0.92	0.92	0.91	0.95	0.95	0.95	0.94	0.94	0.96	0.97	0.97	0.99	0.99	0.99	1.00	1.00	0.99	1.00	1.00	1.00	1.00	1.00	0.99
Tx7	0.95	0.95	0.97	0.99	0.99	0.99	0.99	0.99	1.00	1.00	1.00	1.00	1.00	0.99	1.00	1.00	0.99	1.00	1.00	1.00	1.00	1.00	1.00
Tx8	0.90	0.90	0.89	0.89	0.90	0.90	0.91	0.91	0.91	0.96	0.93	0.95	0.96	0.96	0.93	0.99	0.94	0.99	1.00	0.96	0.95	0.97	0.98
TxEx1	0.95	0.95	0.95	0.95	0.94	0.94	0.94	0.93	0.94	0.94	0.94	0.94	0.98	0.95	0.96	0.96	0.94	0.97	1.00	0.96	0.98	1.00	0.98
TxEx2	0.99	0.99	0.99	0.99	1.00	1.00	1.00	1.00	1.00	1.00	1.00	1.00	1.00	1.00	1.00	1.00	1.00	1.00	1.00	1.00	1.00	1.00	1.00
TxEx3	0.97	0.97	0.97	0.97	0.98	0.98	0.98	0.98	1.00	1.00	1.00	1.00	0.99	1.00	0.98	1.00	1.00	1.00	1.00	1.00	0.99	1.00	1.00
TxEx4	0.73	0.74	0.82	0.93	0.83	0.80	0.82	0.80	0.80	0.84	0.90	0.89	0.93	0.96	0.94	0.99	0.97	0.99	1.00	0.98	0.99	0.87	0.94
znf1	0.63	0.75	0.86	0.86	0.86	0.91	0.90	0.89	1.00	0.88	0.87	0.85	1.00	1.00	1.00	1.00	1.00	1.00	1.00	1.00	1.00	0.98	0.98
znf2	0.72	0.71	0.94	0.94	0.96	0.99	0.99	0.99	1.00	0.99	1.00	1.00	1.00	1.00	1.00	1.00	1.00	1.00	1.00	1.00	1.00	0.99	0.99
DNase1	0.34	0.34	0.34	0.52	0.54	0.56	0.98	0.99	1.00	1.00	1.00	1.00	1.00	1.00	1.00	1.00	1.00	1.00	1.00	1.00	0.99	1.00	1.00
BivProm1	0.64	0.79	0.97	0.94	0.94	0.99	0.95	0.99	0.99	0.99	0.98	0.93	0.91	1.00	1.00	0.99	0.99	1.00	1.00	1.00	1.00	1.00	1.00
BivProm2	0.86	0.93	0.87	0.94	0.96	0.95	0.93	0.93	0.98	0.95	0.99	0.95	0.97	0.99	0.99	0.97	0.97	1.00	1.00	1.00	1.00	0.99	1.00
BivProm3	0.96	0.92	0.91	0.90	0.90	0.90	0.86	0.90	0.90	0.91	0.96	0.96	0.90	0.92	0.93	0.89	0.87	0.87	1.00	0.97	0.96	0.92	0.97
BivProm4	0.62	0.77	0.75	0.73	0.73	0.72	0.65	0.72	0.89	0.73	0.87	0.79	0.92	0.92	0.94	0.72	0.73	0.89	1.00	0.97	0.96	0.96	0.94
PromF1	0.81	0.77	0.72	0.86	0.86	0.86	0.83	0.85	0.84	0.92	0.83	0.83	0.82	0.85	0.90	0.92	0.91	0.96	1.00	1.00	0.87	0.97	0.88
PromF2	0.85	0.88	0.88	0.87	0.89	0.89	0.90	0.88	0.89	0.96	0.97	0.96	0.99	0.99	0.99	1.00	1.00	1.00	1.00	1.00	0.98	0.99	0.95
PromF3	0.98	0.97	0.98	0.96	0.95	0.98	0.97	0.99	0.99	0.99	0.99	1.00	1.00	0.99	1.00	1.00	1.00	1.00	1.00	1.00	1.00	1.00	0.99
PromF4	0.91	0.98	0.96	0.98	0.99	1.00	1.00	1.00	1.00	1.00	1.00	1.00	1.00	1.00	1.00	1.00	1.00	1.00	1.00	1.00	1.00	1.00	1.00
PromF5	0.95	0.91	0.89	0.93	0.94	0.91	0.97	0.93	0.91	0.92	0.92	0.98	0.97	1.00	1.00	1.00	1.00	1.00	1.00	1.00	1.00	1.00	1.00
PromF6	0.85	0.88	0.81	0.97	0.97	0.92	0.95	0.95	0.93	0.95	0.97	0.96	0.96	0.97	1.00	1.00	1.00	1.00	1.00	1.00	1.00	1.00	1.00
PromF7	0.64	0.76	0.81	0.91	0.87	0.98	0.99	0.95	0.97	0.97	0.95	0.95	0.96	0.95	1.00	0.99	1.00	1.00	1.00	1.00	1.00	0.99	1.00
TSS1	0.92	0.96	0.95	0.96	0.96	0.96	0.96	0.98	0.98	0.98	0.99	0.98	0.98	0.98	0.98	1.00	1.00	1.00	1.00	1.00	0.98	1.00	1.00
TSS2	0.83	0.80	0.80	0.82	0.81	0.96	0.92	0.96	0.95	0.96	0.95	0.96	0.95	0.96	0.96	0.96	0.99	1.00	1.00	1.00	0.95	0.99	0.99

Figure S3: Evaluation of full-stack model's number of states. **(A)** AUCs of full-stack models with varying number of states in recovering external genomic annotations. The figure shows the AUC of full-stack models with 20, 40, 60, 80, 100, and 120 states at predicting the genomic locations of multiple different external genomic annotations (CpG Islands, lamina associated domains (laminB1lads), Exon, Gene body, TES, TSS, and TSS2kb regions) (**Methods**). As the number of chromatin states increases, the AUC increases, but the level of the AUC increases diminishes. **(B)** The estimated AIC-BIC curves for models with the number of states ranging from 10 to 120 (5 states apart). We calculated the AIC and BIC based on ChromHMM's output reporting the log-likelihood of observed data for 300 1-Mb regions. Neg2_log_llh: -2 * negative log likelihood of observed data. (**C**) Maximum correlations of emission parameters between each state in the 100-state model and any state for each other model. This is output from ChromHMM's CompareModels command. Rows correspond to the states of the 100-state model. Columns correspond to models with varying numbers of states. Values are the maximum correlation of any state from the model in the column (with varying number of states) with the state from the 100-state in the row. The 100-state model is boxed. This analysis can be effective at establishing some biologically motivated lower bounds on the number of states. For example, state EnhA20, a HUVEC specific enhancer state, is not captured in models with fewer than 100 states. (**D**) Maximum correlations of emission parameters associated with H3K4me1 (an enhancer mark available in all cell types) and the binary vector indicating whether the cell type associated with an emission parameter is in a tissue group (1) or not (0). The rows correspond to different tissue groups from Roadmap Epigenomics Consortium (Kundaje et al., 2015). The columns correspond to different models with varying numbers of states. The values show the maximum correlations mentioned above across all states within a model. The 100-state model is boxed. The last column shows the maximum correlations observed for each tissue type (each row) across all the models.

Emission matrix of 80 representative experiments used to summarize the states

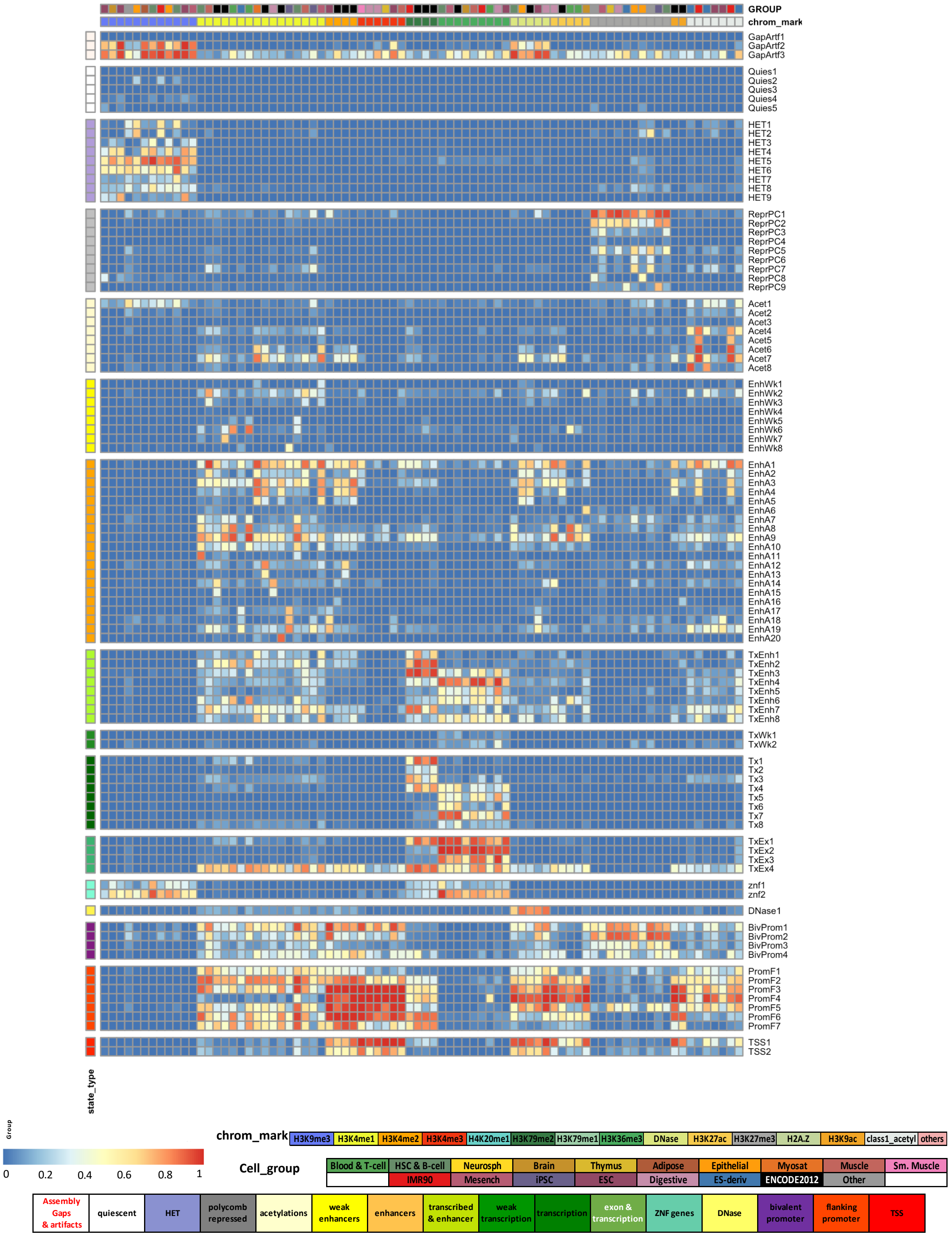

Figure S4: Emission probabilities of 80 datasets chosen to summarize the full-stack model. Each row in the heatmap corresponds to a full-stack state. Each of the 80 columns corresponds to an dataset that has been chosen to represent the space of 1032 datasets. These datasets were chosen through a greedy search of features that optimize prediction of the full-stack annotation using Naïve Bayes with the selected features (**Methods, Additional File 8**). For each state and each dataset, the heatmap gives the probability within the state of observing a binary present call for the dataset's signal. States are displayed in 16 groups as in **Fig. 2A**. Color legends for the emission values, the state groups, chromatin mark, and tissue group are shown at the bottom.

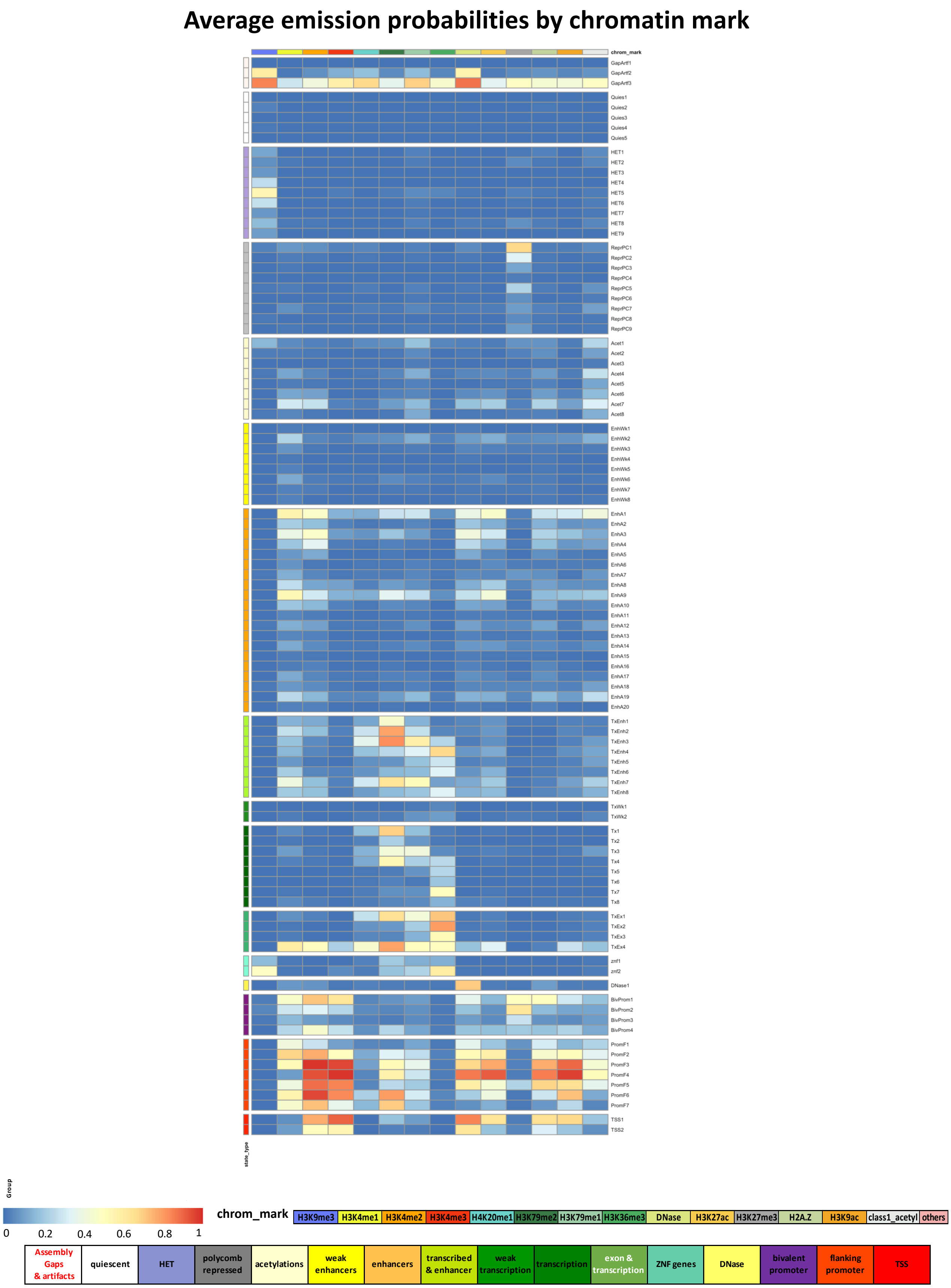

Figure S5: Full-stack states emission probabilities, averaged by chromatin marks. Each column corresponds to an individual chromatin mark or the group of acetylation marks. The heatmap shows for each state the average emission probabilities of datasets associated with each chromatin mark or with the group of acetylations. Color legends for the emission values, the state groups, and chromatin mark are shown at the bottom.

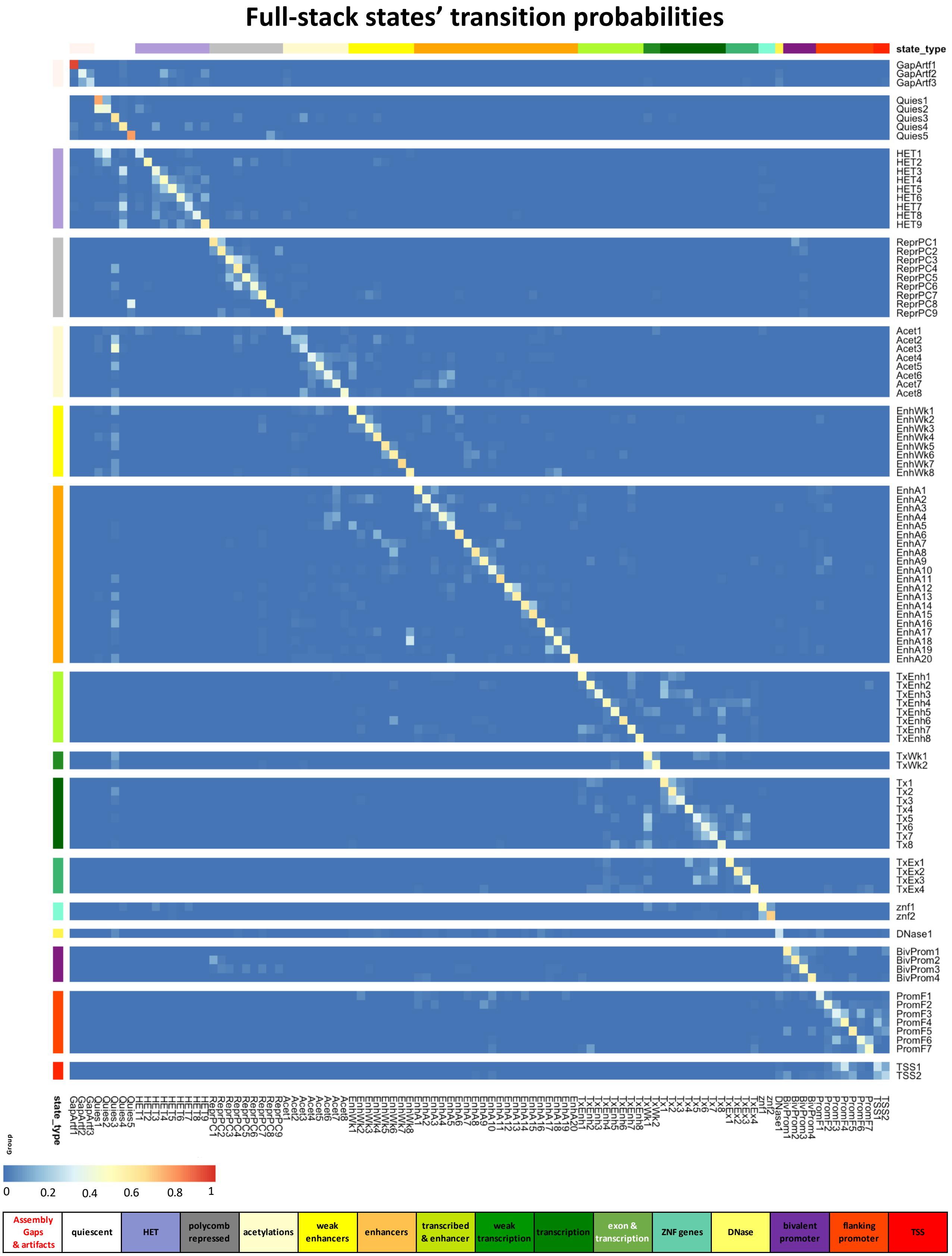

Figure S6: Full-stack states transition probabilities. Each row and each column correspond to a full-stack state, ordered based on their associated state group. The heatmap shows for each state assigned at a current genomic position (rows) the probabilities of transitioning to another state (columns) at the subsequent genomic position. Color legends for the emission values, the state groups are shown at the bottom.

Tissue-group statistically significantly more highly emitted in full-stack states

mneumonics	H3K9me3	H3K4me1	H3K4me3	H3K36me3	H3K27me3	H3K27ac	H3K9ac	DNase
HET9				ESC				
Acet5		ENCODE2012						
Acet6		ENCODE2012	ENCODE2012					
Acet7			ENCODE2012					
EnhWk3					ENCODE2012			
EnhWk4			Brain		ENCODE2012			
EnhWk5		Blood & T-cell	Blood & T-cell					
EnhWk6		Blood & T-cell	Blood & T-cell			Blood & T-cell		
EnhWk7		HSC & B-cell						
EnhA3		ENCODE2012	ENCODE2012					
EnhA4		ENCODE2012	ENCODE2012					
EnhA5		ENCODE2012	ENCODE2012			ENCODE2012		
EnhA6		Brain	Brain		ENCODE2012			
EnhA7		Blood & T-cell,HSC & B-cell		Blood & T-cell				
EnhA8		Blood & T-cell	Blood & T-cell			Blood & T-cell		
EnhA9		Blood & T-cell	Blood & T-cell					
EnhA10		HSC & B-cell						
EnhA11		HSC & B-cell	HSC & B-cell	HSC & B-cell				
EnhA14		Digestive	Digestive			Digestive		Digestive
EnhA15					ENCODE2012	Digestive		Digestive
EnhA16								
EnhA17					ENCODE2012			
EnhA18		ESC						
TxEnh1			ENCODE2012	ENCODE2012	HSC & B-cell			
TxEnh2		Blood & T-cell						
TxEnh6		Blood & T-cell	Blood & T-cell			Blood & T-cell		
TxEnh7								
TxEnh8			ENCODE2012					
Tx1		Blood & T-cell						
Tx4		Blood & T-cell						
TxEx1		Blood & T-cell						
PromF3		HSC & B-cell						
PromF4		HSC & B-cell						
PromF5		HSC & B-cell						
PromF6						ENCODE2012		
TSS1		HSC & B-cell						
TSS2		HSC & B-cell						

Figure S7: Statistically significant tissue—group specificity in full-stack states. The columns correspond to the eight most frequently profiled chromatin marks (H3K9me3, H3K4me1, H3K4me3, H3K36me3, H3K27me3, H3K27ac, H3K9ac, and DNase I hypersensitivity). The rows correspond to states that for at least one chromatin mark show statistically significant higher emission probabilities for one tissue group compared to others (**Methods**). Statistical significance is based on one-sided Mann-Whitney tests at a Bonferroni-corrected p-value threshold of 3.5e-6. The entries in the grid shows the tissue groups reaching significance for each chromatin mark-full-stack state combination.

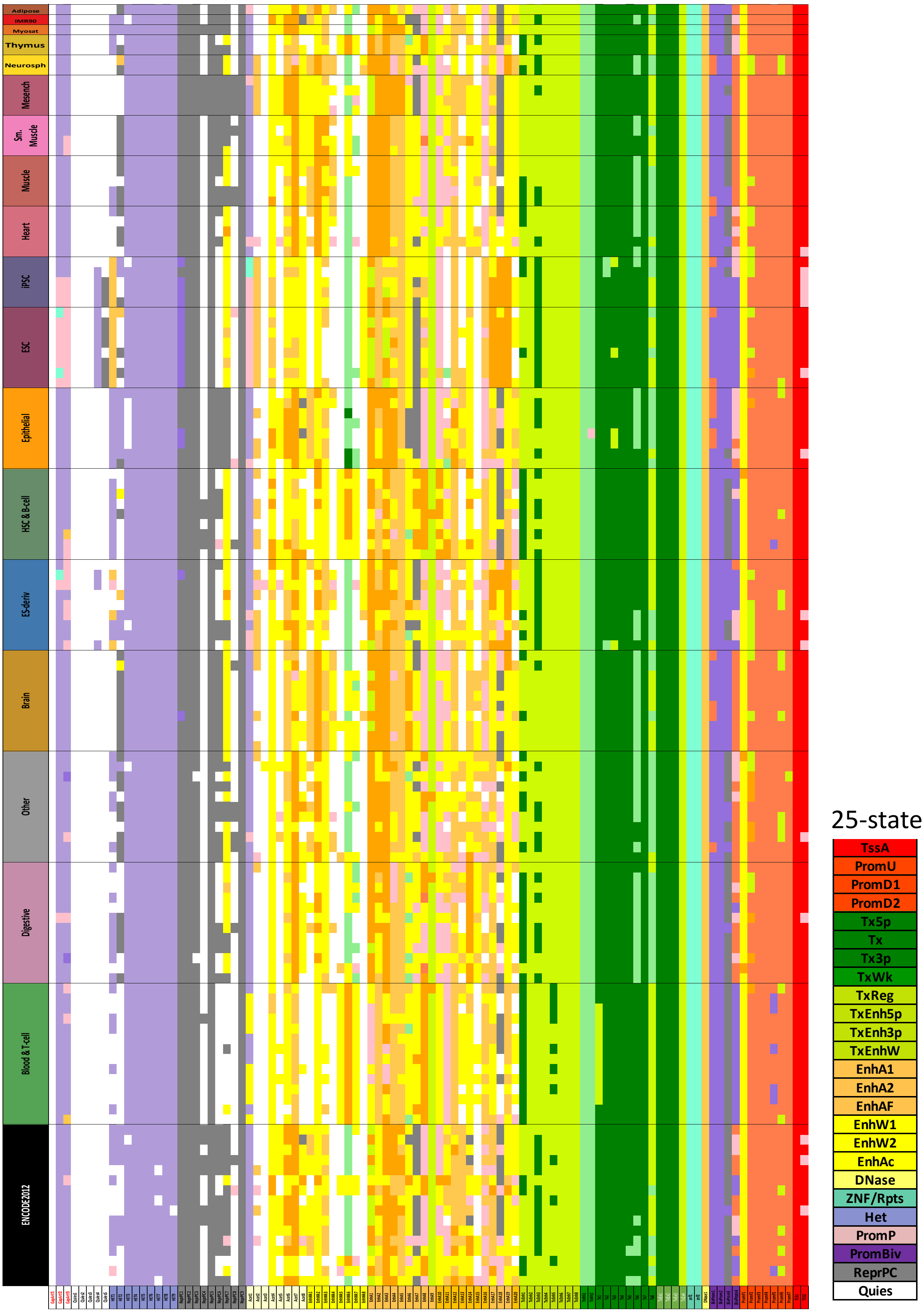

Adipose
IMR90
Myosat
Thymus
Neurosph
Mesench
Sm. Muscle
Muscle
Heart
iPSC
ESC
Epithelial
HSC & B-cell
ES-deriv
Brain
Other
Digestive
Blood & T-cell
ENCODE2012
25-state
TssA
PromU
PromD1
PromD2
Tx5p
Tx
Tx3p
TxWk
TxReg
TxEnh5p
TxEnh3p
TxEnhW
EnhA1
EnhA2
EnhAF
EnhW1
EnhW2
EnhAc
DNase
ZNF/Rpts
Het
PromP
PromBiv
ReprPC
Quies

Figure S8: Full-stack states maximum-enrichments with annotated concatenated-model chromatin states in 127 reference epigenomes. Each row corresponds to one of 127 reference epigenomes from the Roadmap Epigenomics Consortium (**Methods**). Each column corresponds to a state of the full-stack model. Each color entry corresponds to a reference epigenome- full-stack state combination. The color corresponds to the chromatin state from the 25-state model annotating the respective reference epigenome that is most enriched with the respective full-stack state. The figure highlights how some full-stack states are maximally enriched with the same concatenated-model chromatin states across all the reference epigenomes; for example, states znf1 and znf2 are maximally enriched with ZNF Gene state in all 127 reference epigenomes' 25-state concatenated annotation. At the same time, other full-stack states are enriched for distinct concatenated states, for example state EnhA8-- characterized as a blood enhancer state based on emission probabilities-- is most enriched with activate/flanking enhancer in cell types of the groups Blood&Tcell, HSC&B-cell, while being most enriched with poised promoter and weak enhancer states in other cell types. Detailed description of each full-stack state enrichment patterns with concatenated states can be found in **Additional File 5**.

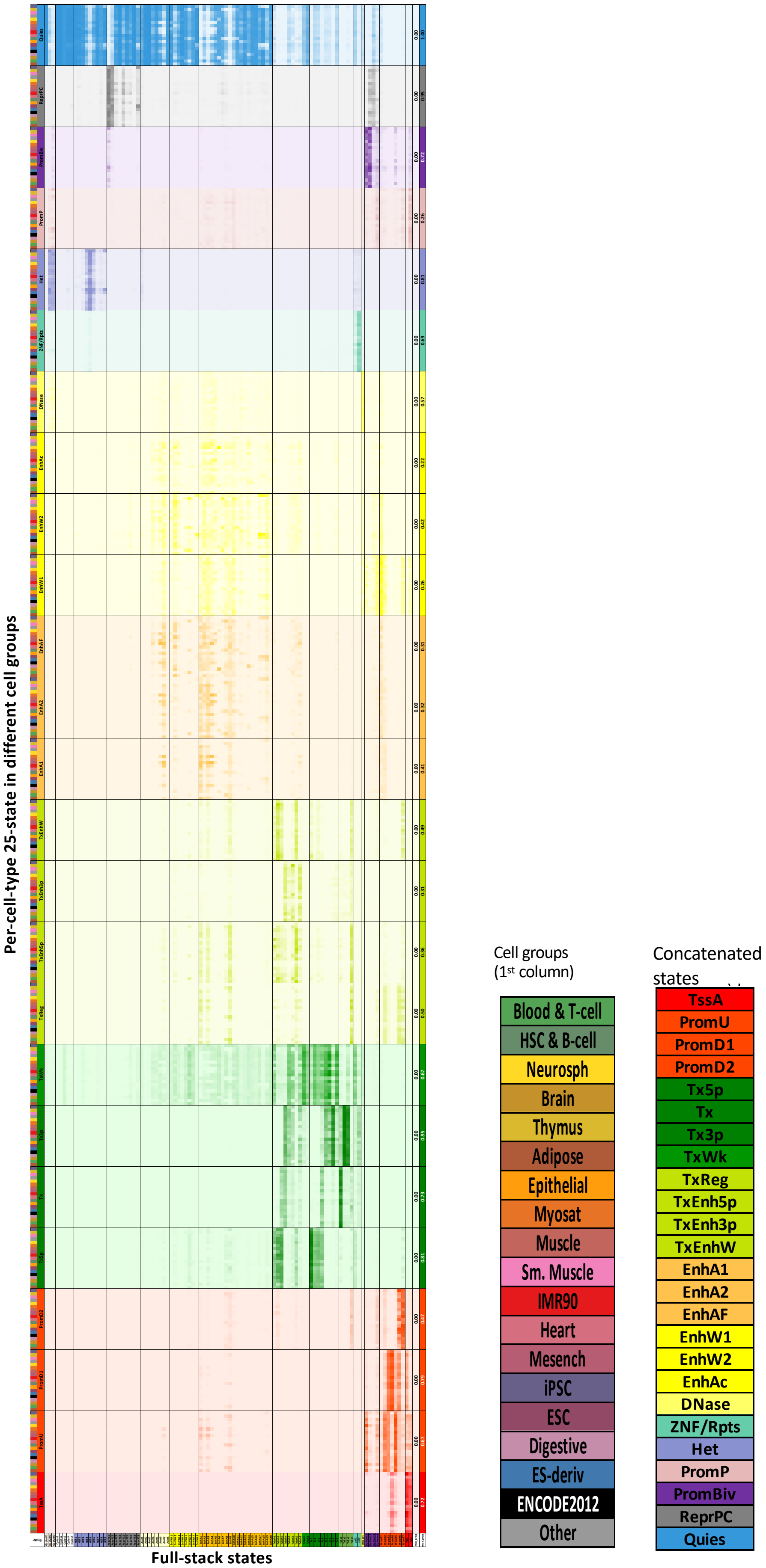
Per-cell-type 25-state in different cell groups
Full-stack states
Cell groups (1st column)
Blood & T-cell
HSC & B-cell
Neurosph
Brain
Thymus
Adipose
Epithelial
Myosat
Muscle
Sm. Muscle
IMR90
Heart
Mesench
iPSC
ESC
Digestive
ES-deriv
ENCODE2012
Other
Concatenated states
TssA
PromU
PromD1
PromD2
Tx5p
Tx
Tx3p
TxWk
TxReg
TxEnh5p
TxEnh3p
TxEnhW
EnhA1
EnhA2
EnhAF
EnhW1
EnhW2
EnhAc
DNase
ZNF/Rpts
Het
PromP
PromBiv
ReprPC
Quies

Figure S9: Estimated probabilities of concatenated-model chromatin states overlapping with full-stack states. The figure shows estimated probabilities of concatenated chromatin state assignments overlapping with full-stack state annotations conditioned on the cell group of the concatenated annotations. This figure is also provided as an excel file in **Additional File 5** where it is accompanied with detailed comments about each full-stack state. The figure is based on a 25-state per-cell type chromatin state model (Ernst & Kellis, 2015), and 19-previously defined tissue groups for the 127 reference epigenomes (Kundaje et al., 2015). Each row corresponds to a combination of per-cell type state (among 25 states) and tissue group, as denoted in the first two columns and legend. Rows corresponding to the same concatenated-model state are grouped together. The first two columns show the colors of tissue groups and concatenated-model state, respectively, as indicated in legends on the right, and matching with the colors in **Fig. S8**, except we changed concatenated-model quiescent 25-state from white to blue for better visibility. The 100 following columns correspond to 100 full-stack states. Values in the heatmap correspond to the estimated probability a genomic position annotated as a full-stack state (column) is also annotated as a concatenated-model state in a cell type from the corresponding tissue group (row) (**Methods**). The last two columns show the minimum and maximum probabilities observed for each per-cell type state for any combination of tissue group and full-stack state. The heatmap colors correspond to the 25-state's colors and are scaled such that the maximum probability values in each block are colored darkest (as seen in the right most column). The figure complements **Fig. S8** in providing information on how each full-stack state can correspond to different 25-per-cell type states in different groups of cells, hence stratifying full-stack states' characteristics in more details. For example, full-stack state ReprPC8 shows high probabilities of overlapping ReprPC state in ESC-related cell groups (ESC, iPSC, and ES-derived), and quiescent state in other cell groups.

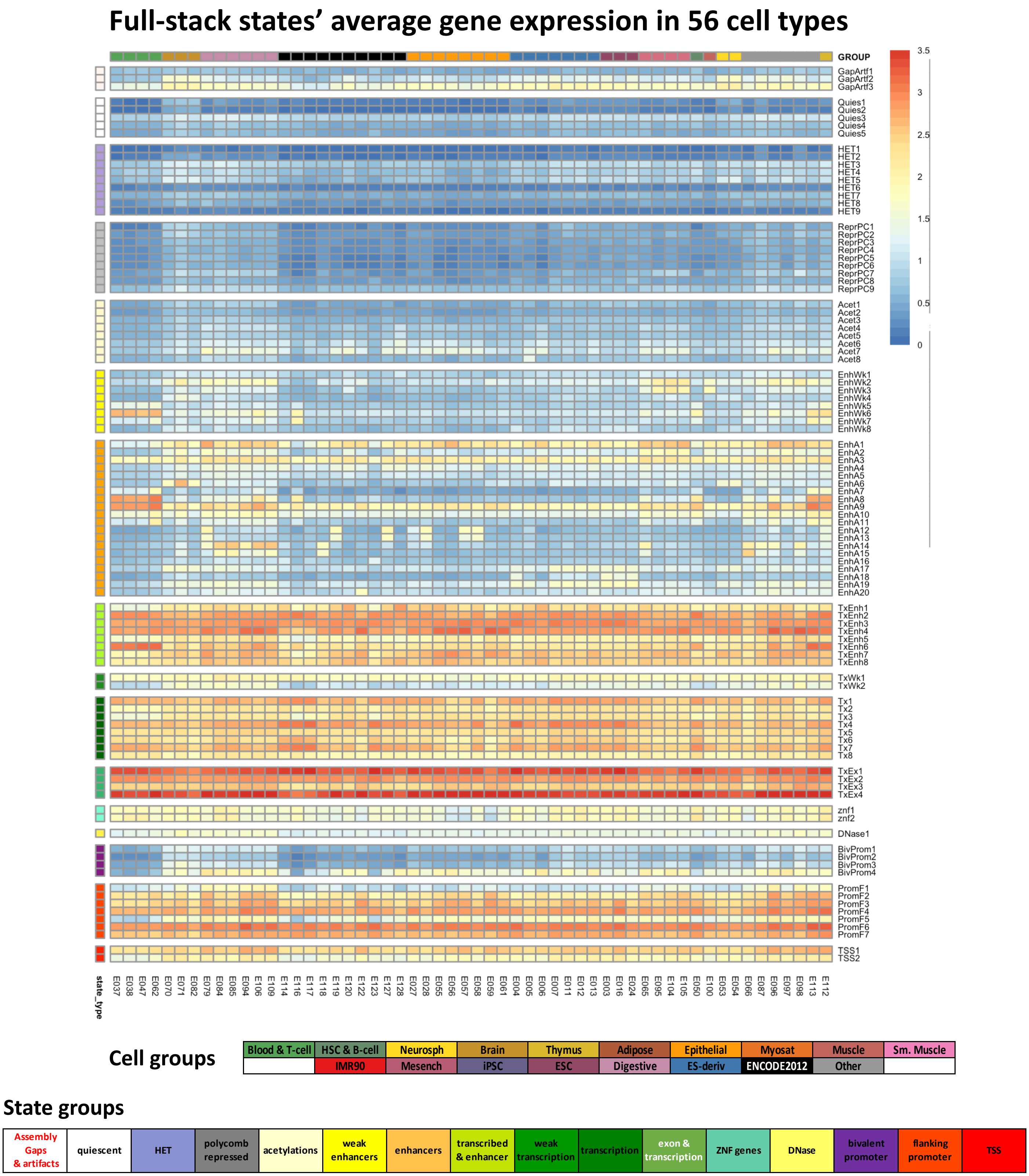

Figure S10: Full-stack states' average gene expression in different cell types. Each row corresponds to one of the 100 full-stack states grouped into state groups as indicated by the legend at the bottom. Each column corresponds to one of 56 cell types whose gene expression data were available from Roadmap Epigenomics (Kundaje et al., 2015). The columns are grouped based on their associated tissue group as indicated by the legend at the bottom. Each column shows the average expression of genes in the respective cell type that overlap with each full-stack state, weighted by the extent of the overlap and the gene length (**Methods**). The figure highlights how states in the transcription and exon group show consistently high gene expression across all cell types, while cell-type-specific enhancer states tend to show higher gene expression in the cell types corresponding to those states.

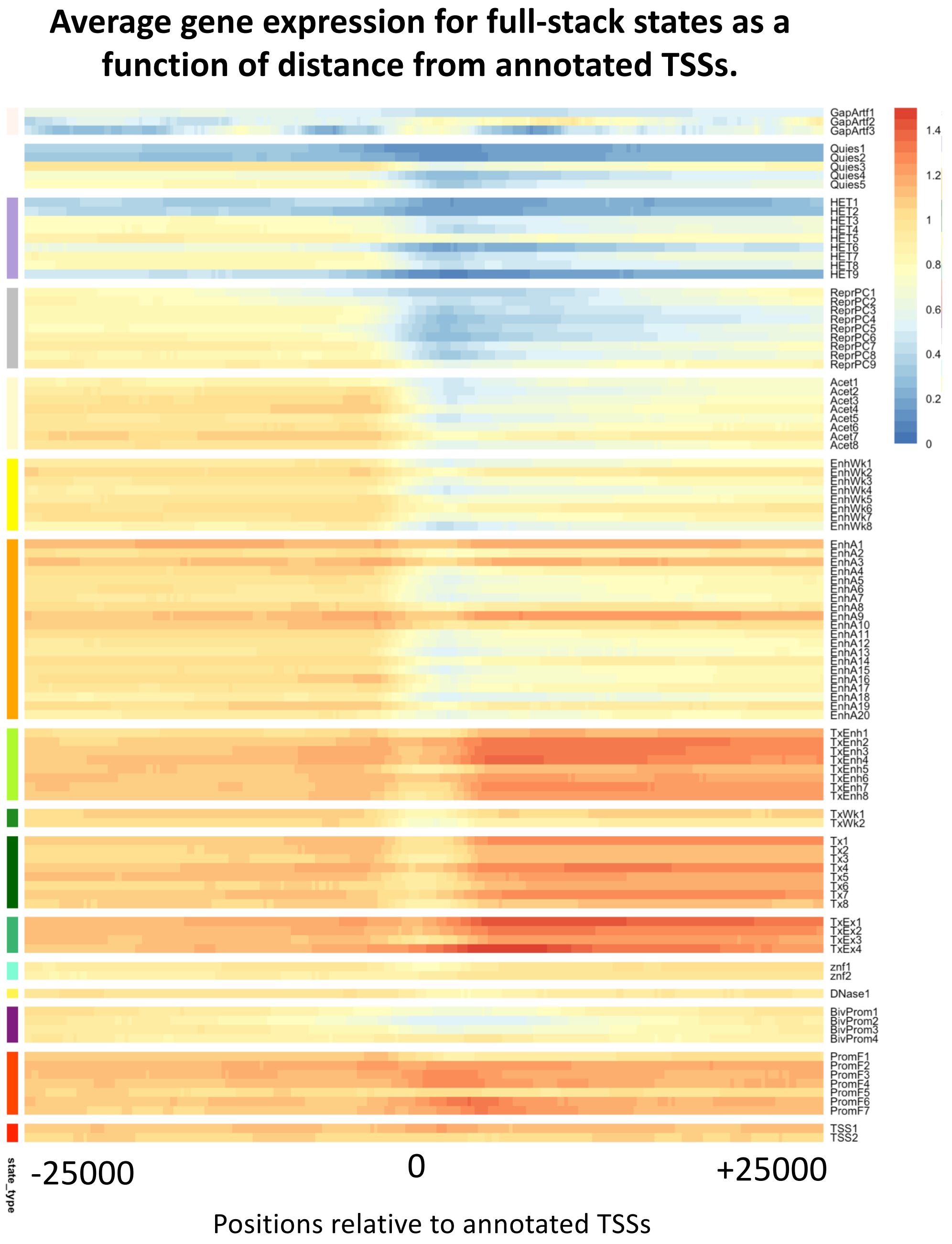

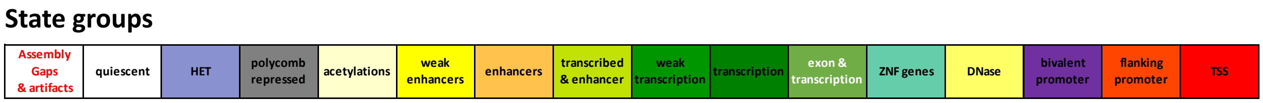

Figure S11: Full-stack states' average gene expression as a function of distance from TSS. Each row corresponds to a full-stack state. Each column corresponds to a 200-bp bin within 25kb relative to annotated TSS, such that TSS is at position 0. Positions downstream of TSS in the direction of transcription have positive coordinate values, and those upstream have negative values. The heatmap shows for each state and position relative to the TSS, the average expression, across 56 cell types, of genes that have the state annotation at such position relative to the TSS (**Methods**). The figure highlights that states in the transcription group tend to have higher gene expression compared to other states, and the average gene expression is usually larger toward the downstream of genes. The figure also shows that for TSS and flanking promoter states, the average gene expression is relatively higher around the TSS compared to other positions.

(A) TSS Neighborhood enrichments (B) TES Neighborhood enrichments

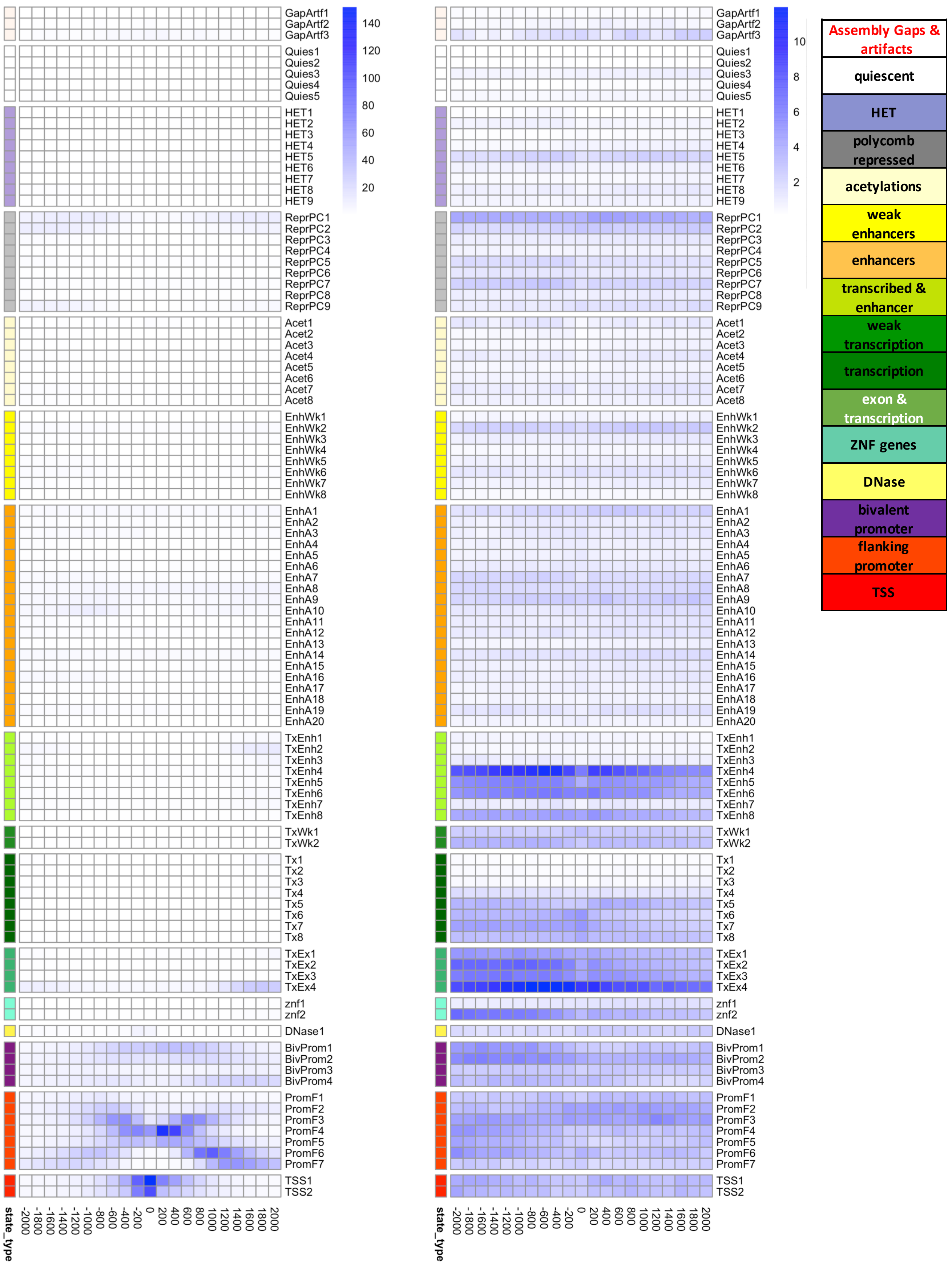

Figure S12: Positional enrichments of full-stack states around annotated transcription start sites and transcription end sites. The figure shows positional fold enrichments for positions within 2kb of annotated **(a)** transcription start sites (TSS) and **(b)** transcription end sites (TES). Each column corresponds to one 200bp window as indicated at bottom. Positive coordinate values represent the number of bases downstream in the 5' to 3' direction of transcription, while negative values represent the number of bases upstream. Enrichments are calculated based on a genome-wide background. Color scale of enrichments is indicated at right for each panel. State groups' color legends are shown at right.

Top gene ontology enrichment terms for bivalent promoter states

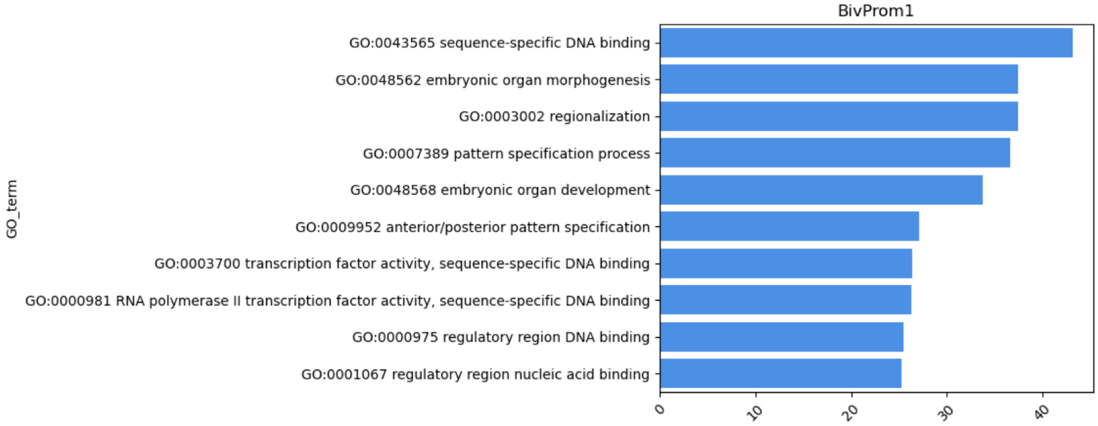

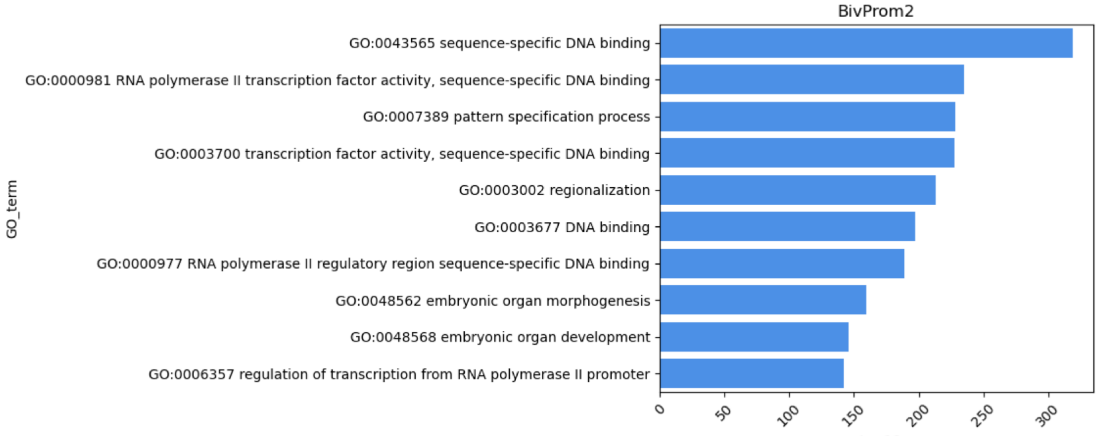

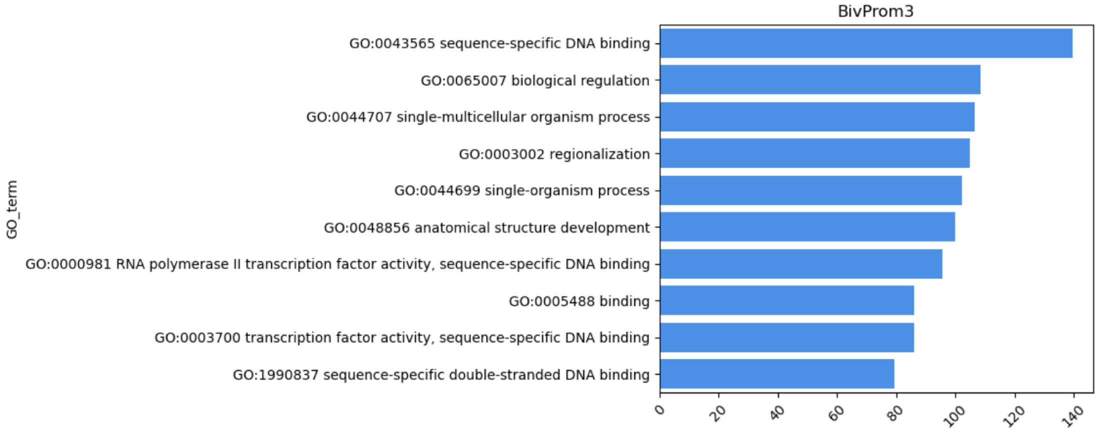

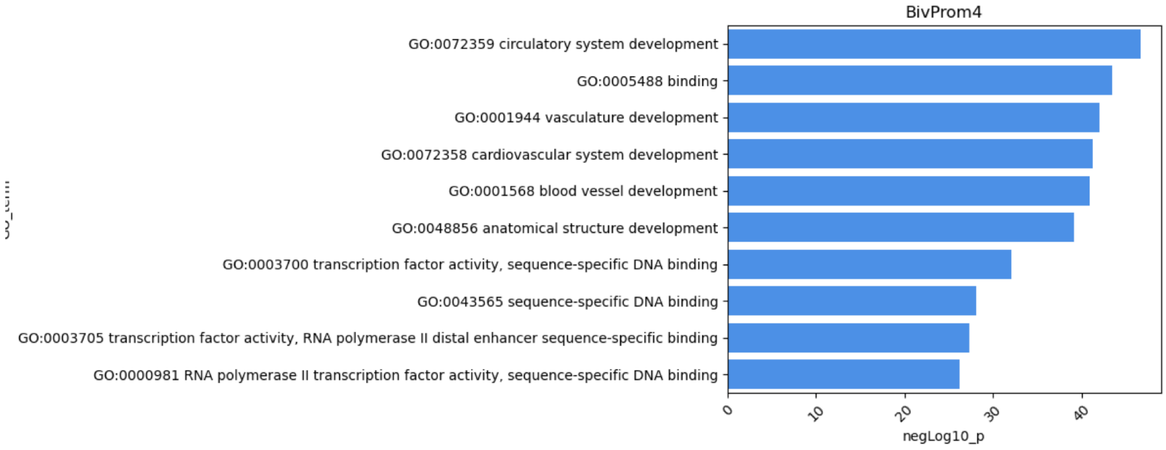

Top gene ontology enrichment terms for flanking promoter states

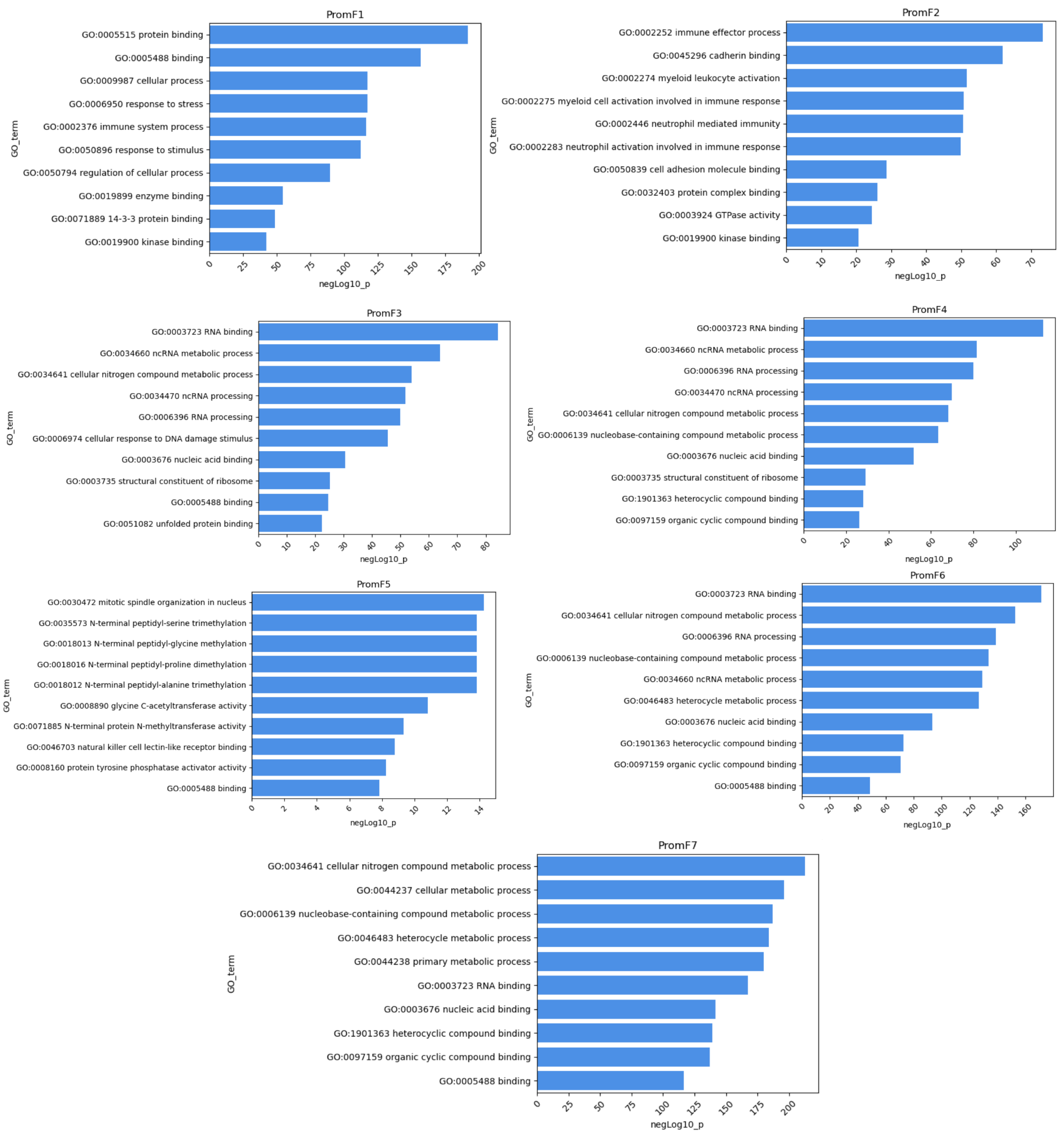

Top gene ontology enrichment terms for TSS states

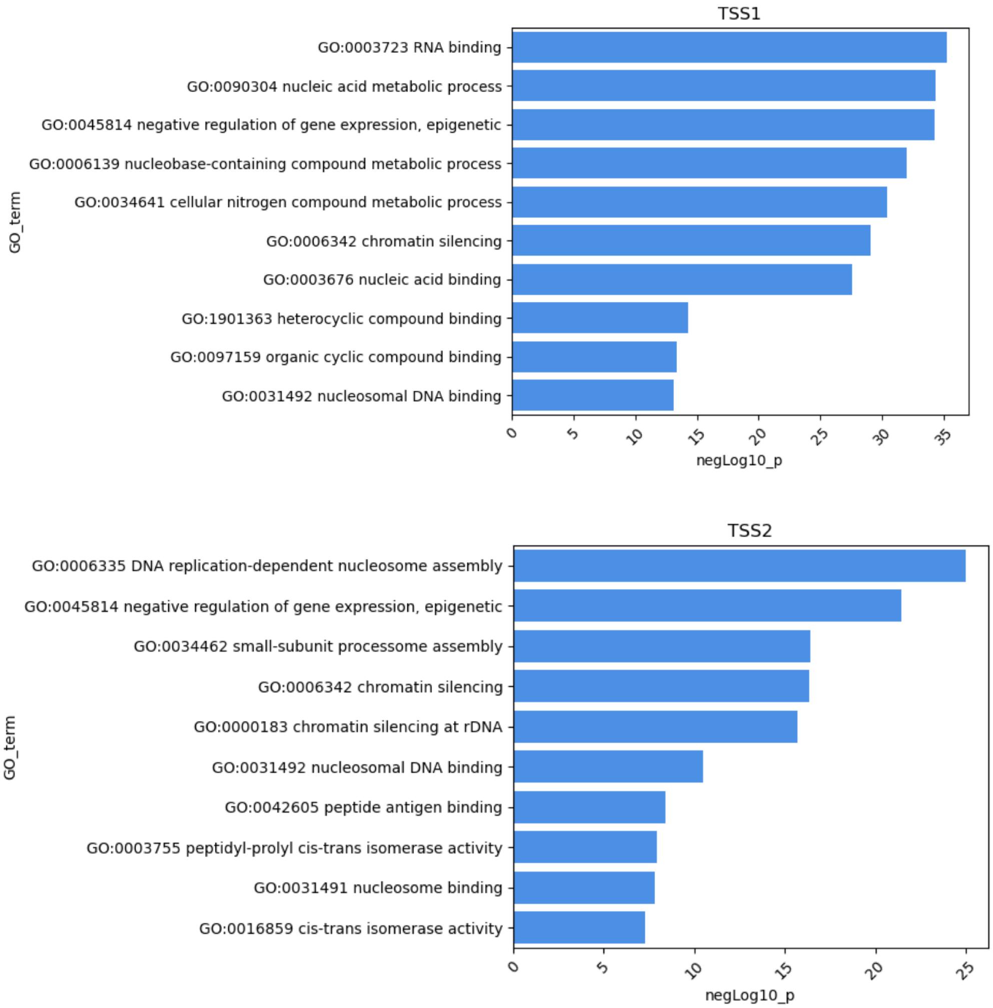

Figure S13: Top GO terms for states in promoter-associated states. Each subpanel corresponds to a full-stack state (state names are shown in the plot title). In each subpanel, the top 5 most significantly enriched GO Biological Process and GO Molecular Function terms are shown on the y-axis (showing a total of 10 GO terms). The length of horizontal bars show the negative log10 (p value) of the GO enrichment, based on the Binomial Test and outputted by GREAT (McLean et al., 2010). The equivalent plots for all full-stack states are available in **Additional File 2.**

CTCF + open chromatin elements enrichments

state	Genome %	Gm12878	H1hesc	Helas3	Hepg2	Huvec	K562
GapArtf1	11.9	0.0	0.0	0.0	0.0	0.0	0.0
GapArtf2	0.1	5.5	3.1	2.7	4.4	3.9	4.0
GapArtf3	0.0	2.7	1.5	1.5	3.8	0.8	1.4
Quies1	9.9	0.0	0.0	0.0	0.0	0.0	0.0
Quies2	3.1	0.0	0.1	0.1	0.0	0.0	0.1
Quies3	12.2	0.0	0.0	0.1	0.0	0.0	0.1
Quies4	4.5	0.0	0.0	0.0	0.0	0.0	0.0
Quies5	1.7	0.0	0.0	0.0	0.0	0.0	0.0
HET1	0.7	0.1	1.6	0.4	0.4	0.1	0.3
HET2	0.7	0.1	2.0	0.2	0.5	0.0	0.4
HET3	1.4	0.0	0.0	0.0	0.0	0.0	0.0
HET4	0.6	0.2	0.1	0.1	0.2	0.2	0.3
HET5	0.2	1.0	2.1	1.1	1.9	1.0	2.0
HET6	0.6	0.1	0.6	0.2	0.5	0.1	0.4
HET7	1.0	0.0	0.3	0.1	0.2	0.0	0.2
HET8	0.4	1.7	1.9	1.6	1.9	1.2	1.9
HET9	1.0	0.1	0.1	0.1	0.1	0.0	0.1
ReprPC1	0.2	0.5	1.0	2.3	1.2	0.3	1.9
ReprPC2	0.3	0.3	0.6	0.7	0.5	0.3	0.6
ReprPC3	1.1	0.1	0.4	0.2	0.2	0.1	0.3
ReprPC4	3.9	0.0	0.1	0.0	0.0	0.0	0.0
ReprPC5	0.6	0.3	3.2	1.0	0.8	0.2	0.9
ReprPC6	1.5	0.1	0.9	0.3	0.4	0.1	0.4
ReprPC7	0.6	1.0	6.5	2.9	3.4	1.1	3.1
ReprPC8	0.5	0.2	0.3	0.3	0.1	0.0	0.3
ReprPC9	0.4	0.1	0.3	0.3	0.3	0.3	0.3
Acet1	0.2	2.0	7.0	2.8	4.1	1.6	3.1
Acet2	0.9	0.4	1.9	0.7	0.9	0.3	0.9
Acet3	2.6	0.0	0.2	0.1	0.1	0.1	0.2
Acet4	0.4	0.6	2.4	2.0	1.3	1.0	1.4
Acet5	0.9	0.1	0.2	0.3	0.2	0.3	0.2
Acet6	0.4	0.2	0.5	0.8	0.3	0.7	0.3
Acet7	0.3	1.6	3.6	3.2	2.5	1.7	1.8
Acet8	0.6	0.2	1.3	0.6	0.6	0.2	0.6
EnhWk1	1.5	0.1	0.0	0.3	0.1	0.5	0.2
EnhWk2	0.4	1.9	5.6	4.3	4.2	2.7	3.4
EnhWk3	0.8	0.2	0.8	0.6	0.6	0.6	0.5
EnhWk4	2.2	0.1	0.1	0.1	0.1	0.3	0.1
EnhWk5	1.0	0.3	0.2	0.2	0.3	0.3	0.3
EnhWk6	0.6	1.0	0.5	0.6	0.5	0.7	0.8
EnhWk7	0.5	0.9	0.3	0.5	0.4	0.4	0.6
EnhWk8	1.4	0.1	1.2	0.1	0.3	0.2	0.2
EnhA1	0.2	6.8	5.3	6.0	5.9	4.4	5.0
EnhA2	0.3	1.6	3.0	3.1	2.6	3.9	2.6
EnhA3	0.2	16.5	9.9	10.7	12.3	10.0	14.0
EnhA4	0.3	1.4	1.4	2.9	1.8	1.9	1.7
EnhA5	0.7	0.6	0.4	2.0	0.8	2.3	0.8
EnhA6	0.6	0.5	1.1	0.8	0.9	0.6	0.9
EnhA7	0.4	3.0	4.8	2.7	3.3	1.5	3.6
EnhA8	0.3	1.4	3.1	2.3	2.0	1.7	2.3
EnhA9	0.2	2.5	5.7	5.7	5.3	4.5	3.1
EnhA10	0.4	3.4	1.9	3.1	2.3	3.4	3.0
EnhA11	0.7	0.4	0.3	0.4	0.5	0.3	0.5
EnhA12	0.3	1.4	3.8	3.9	2.4	1.3	2.0
EnhA13	0.8	0.2	0.3	0.6	0.2	0.2	0.2
EnhA14	0.4	1.0	2.4	2.3	1.7	1.0	1.8
EnhA15	1.0	0.1	0.2	0.3	0.4	0.1	0.3
EnhA16	0.6	5.2	5.7	5.7	5.8	6.2	6.9
EnhA17	0.5	1.5	3.4	1.7	1.9	2.2	1.8
EnhA18	0.5	0.4	2.9	0.7	1.1	0.3	0.9
EnhA19	0.3	3.1	3.9	5.2	4.4	3.4	4.1
EnhA20	0.3	0.1	0.1	0.3	0.2	0.6	0.2
TxEnh1	0.4	0.4	0.3	0.7	0.5	0.9	0.5
TxEnh2	0.4	1.0	1.0	1.3	1.3	1.6	1.4
TxEnh3	0.2	1.1	2.7	1.7	1.6	1.0	0.9
TxEnh4	0.3	3.8	7.9	7.4	6.2	5.3	2.8
TxEnh5	0.5	2.0	6.9	4.2	5.3	3.2	3.7
TxEnh6	0.2	2.9	4.5	4.2	4.1	3.6	2.5
TxEnh7	0.3	3.1	4.9	5.2	4.5	4.1	3.0
TxEnh8	0.2	4.1	4.3	5.2	4.6	4.3	3.5
TxWk1	2.8	0.1	0.1	0.1	0.1	0.1	0.1
TxWk2	0.8	0.3	2.2	0.9	1.3	0.5	1.1
Tx1	0.8	0.2	0.2	0.3	0.2	0.3	0.3
Tx2	1.6	0.1	0.1	0.1	0.1	0.2	0.1
Tx3	0.5	0.5	1.6	1.0	1.0	0.5	0.9
Tx4	0.5	0.3	0.2	0.3	0.3	0.4	0.3
Tx5	0.9	0.1	0.5	0.4	0.3	0.2	0.3
Tx6	1.1	0.1	0.0	0.1	0.1	0.1	0.1
Tx7	0.8	0.2	0.1	0.3	0.2	0.3	0.2
Tx8	0.7	1.6	1.1	1.6	1.7	2.1	1.5
TxEx1	0.3	0.3	0.6	0.4	0.2	0.3	0.2
TxEx2	0.6	0.2	1.2	0.8	0.6	0.3	0.2
TxEx3	0.7	0.2	2.3	1.3	1.3	0.6	0.9
TxEx4	0.1	2.4	3.3	3.4	2.4	2.4	1.1
znf1	0.4	0.2	0.5	0.3	0.4	0.3	0.3
znf2	0.2	0.3	1.2	0.7	0.7	0.6	0.9
DNase1	0.2	260.3	130.8	187.1	202.5	239.9	214.8
BivProm1	0.1	6.2	0.7	4.7	4.3	3.3	5.0
BivProm2	0.2	3.5	1.0	3.9	4.4	2.6	4.4
BivProm3	0.3	4.8	7.7	6.6	6.6	5.1	6.6
BivProm4	0.1	2.9	2.5	2.7	3.1	2.4	3.4
PromF1	0.2	44.4	23.9	31.9	27.5	42.3	28.1
PromF2	0.1	8.0	6.2	6.5	4.8	6.5	5.1
PromF3	0.2	0.1	0.2	0.3	0.4	0.0	0.3
PromF4	0.2	0.0	0.0	0.0	0.1	0.0	0.1
PromF5	0.1	2.8	0.3	2.9	2.2	0.3	2.9
PromF6	0.1	0.0	0.1	0.0	0.1	0.0	0.1
PromF7	0.2	0.2	0.6	0.2	0.2	0.3	0.2
TSS1	0.1	0.3	0.8	0.6	0.5	0.0	0.9
TSS2	0.1	16.3	8.8	8.9	10.9	8.2	12.2
Base	100	0.2	0.4	0.3	0.2	0.3	0.2

CTCF + *limited* open chromatin elements enrichments

state	Genome %	Gm12878_Ctcf	H1hesc_Ctcf	Helas3_Ctcf	Hepg2_Ctcf	Huvec_Ctcf	K562_Ctcf
GapArtf1	11.9	0.0	0.0	0.0	0.0	0.0	0.0
GapArtf2	0.1	0.7	0.6	0.6	0.9	0.6	0.4
GapArtf3	0.0	0.3	0.0	0.4	0.3	0.0	0.1
Quies1	9.9	0.2	0.2	0.2	0.2	0.2	0.1
Quies2	3.1	0.5	1.0	0.5	0.7	0.3	0.5
Quies3	12.2	0.5	0.4	0.5	0.5	0.8	0.6
Quies4	4.5	0.2	0.2	0.2	0.2	0.2	0.2
Quies5	1.7	0.4	0.3	0.4	0.3	0.3	0.4
HET1	0.7	2.0	4.0	1.9	2.8	1.1	1.6
HET2	0.7	1.3	2.9	1.3	2.0	0.5	1.9
HET3	1.4	0.3	0.3	0.3	0.4	0.4	0.4
HET4	0.6	0.6	0.5	0.6	0.7	0.7	0.7
HET5	0.2	2.3	2.5	2.4	2.8	2.6	2.8
HET6	0.6	1.2	1.8	1.4	2.0	1.0	1.7
HET7	1.0	0.8	1.4	1.0	1.4	0.8	1.2
HET8	0.4	2.5	2.5	2.6	2.9	2.5	2.9
HET9	1.0	0.5	0.4	0.6	0.7	0.5	0.7
ReprPC1	0.2	1.1	0.2	2.2	1.0	0.3	1.3
ReprPC2	0.3	1.4	0.7	1.6	1.2	0.8	1.7
ReprPC3	1.1	1.2	1.4	1.3	1.3	1.0	1.5
ReprPC4	3.9	0.5	0.6	0.6	0.6	0.6	0.7
ReprPC5	0.6	2.2	2.9	2.4	2.0	1.8	1.9
ReprPC6	1.5	1.4	2.4	1.6	1.7	1.3	1.8
ReprPC7	0.6	3.9	3.4	3.7	2.9	3.8	2.7
ReprPC8	0.5	1.6	1.6	1.6	1.4	1.3	1.7
ReprPC9	0.4	1.1	0.5	1.1	1.0	1.2	1.1
Acet1	0.2	3.5	4.1	3.3	3.8	3.5	2.8
Acet2	0.9	2.9	3.7	2.8	3.3	2.6	2.8
Acet3	2.6	0.9	1.2	1.0	1.1	1.0	1.1
Acet4	0.4	3.2	3.5	2.9	3.0	3.1	2.8
Acet5	0.9	1.3	1.4	1.4	1.4	1.5	1.4
Acet6	0.4	1.5	1.4	1.4	1.5	1.4	1.5
Acet7	0.3	2.8	2.5	1.7	2.0	1.7	2.4
Acet8	0.6	1.8	2.5	1.9	2.0	1.9	2.1
EnhWk1	1.5	1.3	1.0	1.2	1.2	1.7	1.2
EnhWk2	0.4	3.6	2.8	3.3	2.4	3.7	2.6
EnhWk3	0.8	1.9	2.2	1.9	1.9	2.0	2.1
EnhWk4	2.2	1.4	1.2	1.1	1.3	1.5	1.1
EnhWk5	1.0	1.5	1.2	1.2	1.3	1.7	1.5
EnhWk6	0.6	1.3	1.5	1.7	1.6	2.0	1.8
EnhWk7	0.5	0.5	1.3	1.4	1.6	1.8	1.7
EnhWk8	1.4	2.0	1.9	1.7	2.1	1.9	1.7
EnhA1	0.2	3.0	1.0	1.5	1.2	1.2	1.8
EnhA2	0.3	4.6	3.4	3.6	3.5	3.6	4.1
EnhA3	0.2	3.3	3.6	1.9	3.0	1.0	3.3
EnhA4	0.3	2.7	2.0	1.1	2.2	1.1	2.3
EnhA5	0.7	2.7	2.1	1.9	2.5	2.1	2.2
EnhA6	0.6	2.7	2.3	2.3	2.3	2.7	2.5
EnhA7	0.4	3.3	3.6	4.1	3.7	4.3	3.2
EnhA8	0.3	0.7	2.1	2.4	2.1	2.4	1.8
EnhA9	0.2	0.4	1.7	1.7	1.2	1.2	1.0
EnhA10	0.4	2.6	3.0	2.8	2.8	3.7	2.9
EnhA11	0.7	1.1	1.2	1.2	1.2	1.4	1.4
EnhA12	0.3	3.4	3.1	2.4	2.9	3.0	2.8
EnhA13	0.8	1.2	1.2	1.1	1.2	1.2	1.2
EnhA14	0.4	2.7	2.6	2.5	1.2	2.9	2.2
EnhA15	1.0	1.2	1.1	1.2	0.8	1.3	1.4
EnhA16	0.6	10.0	7.0	7.4	8.9	9.8	7.1
EnhA17	0.5	5.0	2.6	4.2	4.6	4.8	4.4
EnhA18	0.5	2.9	1.1	2.6	3.0	2.6	2.8
EnhA19	0.3	5.2	0.7	4.3	3.8	5.3	3.8
EnhA20	0.3	1.2	0.9	1.0	1.0	0.2	1.1
TxEnh1	0.4	1.4	0.9	1.2	1.0	1.0	1.1
TxEnh2	0.4	0.6	0.9	1.1	0.7	1.0	0.7
TxEnh3	0.2	0.4	1.1	0.9	0.6	0.8	0.5
TxEnh4	0.3	0.4	1.0	1.4	0.2	0.1	0.6
TxEnh5	0.5	2.1	2.2	2.9	1.9	2.1	2.2
TxEnh6	0.2	0.6	1.8	2.1	1.2	1.2	1.2
TxEnh7	0.3	1.6	1.3	1.9	1.2	1.8	1.3
TxEnh8	0.2	0.9	1.5	1.5	0.7	0.8	1.3
TxWk1	2.8	0.5	0.5	0.6	0.5	0.7	0.6
TxWk2	0.8	1.6	2.1	1.9	1.7	1.7	1.8
Tx1	0.8	0.5	0.5	0.6	0.4	0.7	0.5
Tx2	1.6	0.5	0.5	0.6	0.5	0.8	0.6
Tx3	0.5	1.3	1.6	1.5	1.3	1.5	1.3
Tx4	0.5	0.3	0.5	0.5	0.2	0.4	0.3
Tx5	0.9	0.3	0.7	0.7	0.4	0.4	0.6
Tx6	1.1	0.3	0.3	0.4	0.3	0.6	0.3
Tx7	0.8	0.2	0.3	0.3	0.1	0.2	0.2
Tx8	0.7	1.6	1.8	1.9	1.5	1.9	1.8
TxEx1	0.3	0.1	0.2	0.2	0.0	0.0	0.1
TxEx2	0.6	0.0	0.3	0.4	0.0	0.0	0.1
TxEx3	0.7	0.4	1.0	1.0	0.4	0.2	0.6
TxEx4	0.1	0.1	0.3	0.3	0.2	0.1	0.2
znf1	0.4	0.5	0.8	0.9	0.8	0.8	0.8
znf2	0.2	0.4	0.5	0.7	0.4	0.4	0.6
DNase1	0.2	31.7	32.8	26.5	37.6	18.6	28.5
BivProm1	0.1	1.4	0.0	3.3	0.9	0.4	1.2
BivProm2	0.2	1.7	0.1	2.7	1.3	0.8	1.4
BivProm3	0.3	5.9	1.5	4.9	3.3	4.3	3.5
BivProm4	0.1	3.4	1.0	2.4	2.1	1.4	2.8
PromF1	0.2	7.4	2.5	6.4	3.3	7.5	3.8
PromF2	0.1	0.7	0.6	1.4	0.6	0.3	0.9
PromF3	0.2	0.0	0.0	0.1	0.0	0.0	0.0
PromF4	0.2	0.0	0.0	0.0	0.0	0.0	0.0
PromF5	0.1	0.6	0.0	1.7	0.4	0.1	1.0
PromF6	0.1	0.0	0.0	0.0	0.0	0.0	0.0
PromF7	0.2	0.1	0.2	0.1	0.1	0.2	0.3
TSS1	0.1	0.0	0.0	0.3	0.1	0.0	0.1
TSS2	0.1	1.8	1.0	2.7	1.3	0.7	1.6
	100	0.6	0.7	0.8	0.6	0.6	0.7

Figure S14: Full-stack states enrichments with CTCF associated chromatin states. (A) The heatmap shows enrichment values for the full-stack states (rows) and a chromatin state that corresponds to CTCF with open chromatin and limited histone modification signal from concatenated annotations in six different cell types (columns). CTCF signals were included as input for training these concatenated chromatin state models (**Methods**). Coloring of enrichments is column specific. **(B)** Similar to **(A),** except showing enrichments for a state associated with CTCF datasets with limited open chromatin and limited histone modification signals.

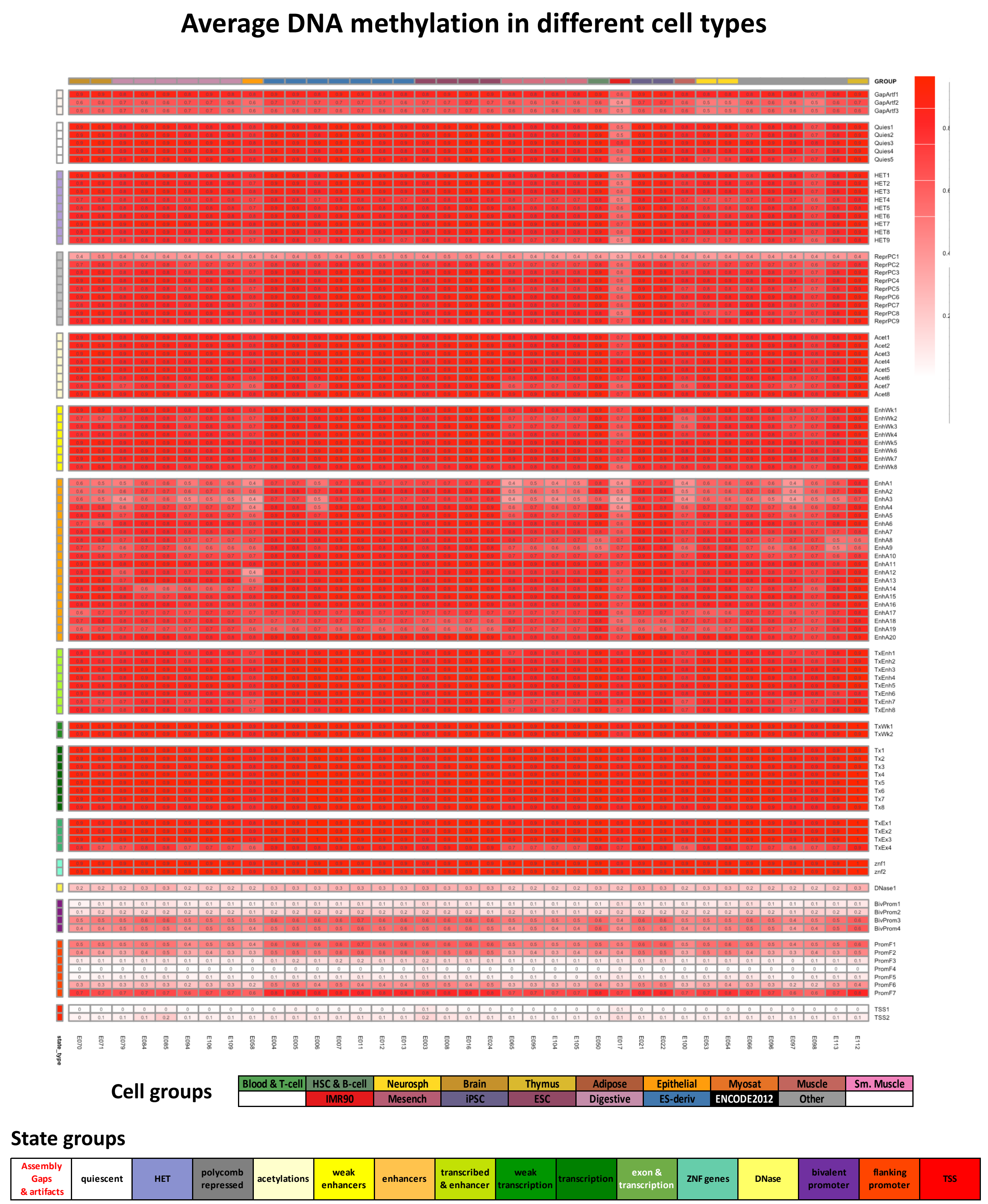

Figure S15: Full-stack states' average DNA methylation in different cell types. Each row corresponds to one of the 100 full-stack states grouped into state groups as indicated by the legend at the bottom. Each column corresponds to one cell type whose DNA methylation data were available from Roadmap Epigenomics. The columns are grouped based on their associated tissue group as indicated by the legend at the bottom. Each column shows the average DNA methylation level in the respective cell type that overlaps with each full-stack state (**Methods**). Among promoters-associated states, those most enriched with CpG islands also show lowest average DNA methylation levels (**Fig. 3A**), consistent with expectation (Jones & Takai, 2001; Weber et al., 2007). The lower DNAme levels of IMR90 compared to other cell types might be related to a technical batch effect since it was one of two original WGBS datasets collected (Lister et al., 2009).

Enrichments with polycomb repressed protein groups PRC1 and PRC2

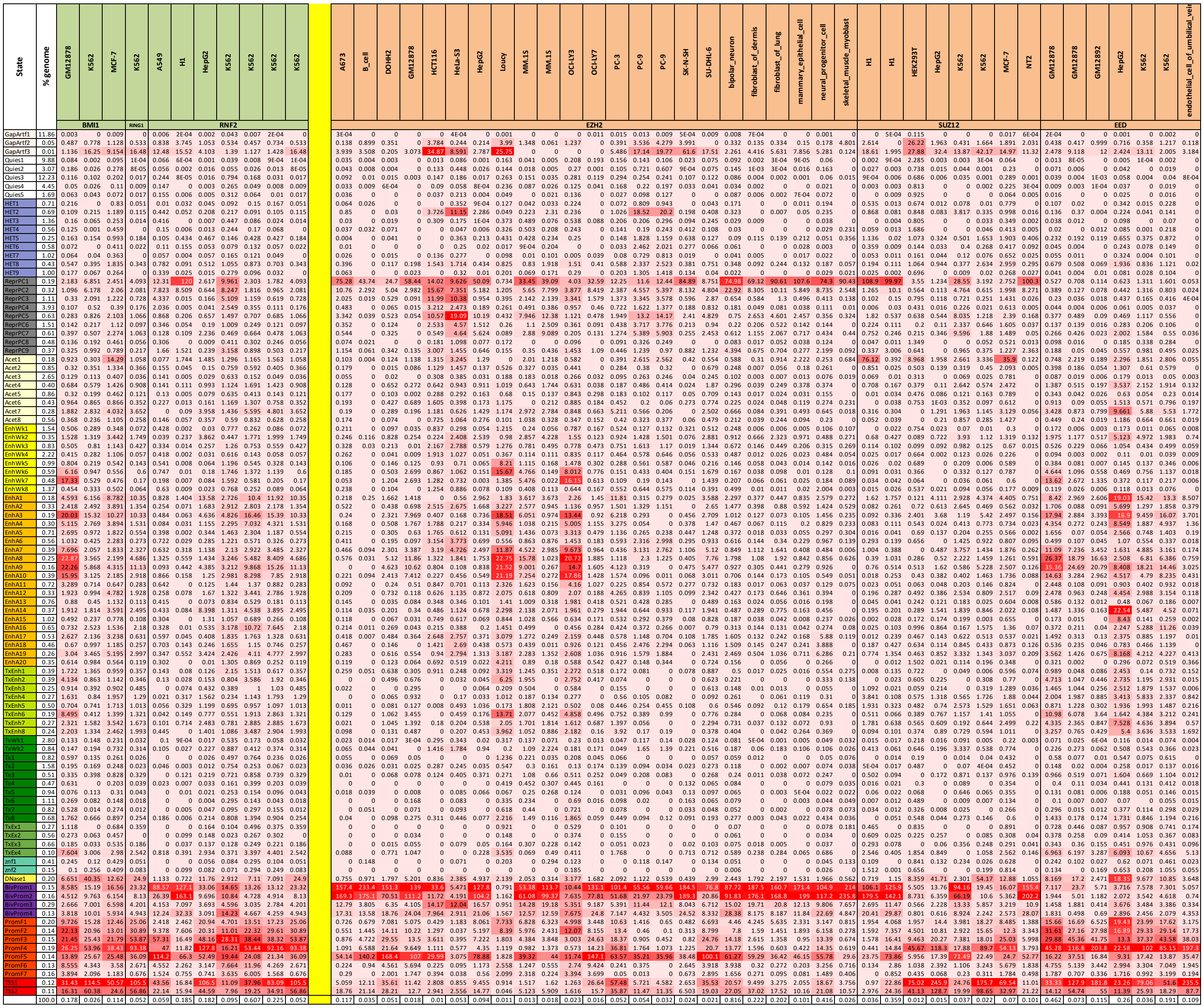

Figure S16: Full-stack states enrichments with Polycomb Repressive protein complexes PRC1 (green column headers) and PRC2 (orange column headers). The heatmap shows enrichment values for the full-stack states (rows) and the binding sites of subunits of PRC1 and PRC2 in different cell types (columns) that were available from ENCODE project (**Methods**). Coloring of enrichments is column specific with highest and lowest enrichment values in each column are colored red and white, respectively. The first two columns show state names, and their percentage of genome coverage. The last row shows the percentage of genome coverage for each type of PRCs.

Top enriched states with PRC1 and PRC2

State	% genome	BMI1 GM12878	BMI1 K562	BMI1 MCF-7	RING1 K562	RNF2 A549	RNF2 H1	RNF2 HepG2	RNF2 K562	RNF2 K562	RNF2 K562	RNF2 K562
GapArtf3	0.01	1.1	16	9.2	16	12	16	4.1	1.4	1.1	1.4	16
HET2	0.69	0.1	0.2	1.2	0.1	0.4	0.1	0.2	0.2	0.1	0.1	0.1
ReprPC1	0.19	2.2	6.9	2.5	4.1	12	120	2.6	10	2.3	1.8	4.1
ReprPC5	0.63	0.3	0.8	2.1	1.1	0.9	0.2	0.7	1.5	0.7	0.7	1.1
Acet1	0.18	0.9	0.3	14	1.1	0.1	1.7	1.5	1.3	1.2	1.6	1.1
EnhWk7	0.48	17	0.5	0.5	0.2	0.2	0	0.1	1.6	0.6	0.2	0.2
EnhA8	0.25	23	3.6	2.2	4.7	1.3	0.6	1.4	3.2	5.5	8.4	4.7
EnhA9	0.16	22	5.9	4.3	11	0.1	0.4	4.4	3.2	9.9	15	11
EnhA10	0.39	16	3.1	2.2	2.9	0.9	0.2	1.2	3	8.3	7.9	2.9
EnhA14	0.37	1.9	1.8	3.6	2.5	0.4	0.1	8.4	1.3	4.5	3.9	2.5
BivProm1	0.15	8.6	15	17	23	89	127	33	15	13	13	23
BivProm2	0.16	4.5	9.8	6.2	8.1	26	163	9.7	11	4.7	4.9	8.1
BivProm3	0.29	2.7	7	6.6	4.2	4.2	7.1	3.7	4.6	3	2.8	4.2
PromF3	0.15	21	25	22	54	57	16	48	28	38	38	54
PromF4	0.19	26	54	38	93	47	12	128	16	53	92	93
PromF5	0.14	14	26	25	36	114	66	52	19	24	21	36
TSS1	0.12	31	115	51	106	44	17	106	11	38	83	106
TSS2	0.11	16	60	25	57	22	16	45	8	19	35	57

State	% genome	EZH2 A673	B_cell	DOHH2	GM12878	HCT116	HeLa-S3	HepG2	Loucy	MM.1S	MM.1S	OCI-LY3	OCI-LY7	PC-3	PC-9	PC-9	SK-N-SH	SU-DHL-6	bipolar_neuron	fibroblast_of_dermis	fibroblast_of_lung	mammary_epithelial_cell	neural_progenitor_cell	skeletal_muscle_myoblast
GapArtf3	0.01	3.9	3.5	0.2	3.1	35	8.6	2.8	26	0	0	0	0	5.5	17	20	62	18	2.3	4.4	5.6	7.9	5.3	0.1
HET2	0.69	0.8	0	0	0	3.7	11	2.3	0	0.2	2.3	0.2	0	1	19	20	0.2	0.4	0.3	0	0	0.1	0.2	0
ReprPC1	0.19	75	44	25	58	14	9.6	50	0.7	33	39	4	33	12	12	12	85	8.8	75	69	91	108	74	90
ReprPC5	0.63	3.3	0	0.5	0.1	11	19	10	0.4	7.9	12	1.1	0.5	1.9	13	14	2.4	4.8	0.7	2.7	4.6	2.5	0.4	0.3
Acet1	0.18	0.1	0	0.1	1.1	1.3	3.2	1.3	0	2	1.2	0.6	0	0.4	2.6	2.6	0.4	0.6	0.6	0.3	0.9	2.2	0.3	0.7
EnhWk7	0.48	0.1	0	1.2	2.7	1.3	0.7	0	1.4	5.5	0	16	0.6	0.1	0.2	0.1	0	1.4	0.2	0.1	0.1	0	0.2	0.1
EnhA8	0.25	0.6	0	5.1	12	1.3	1.8	1.8	23	16	1	21	1.9	1.1	2.3	1.2	0.4	7.8	1.8	1.1	1.9	0.8	0.9	0.6
EnhA9	0.16	0	0	4.6	11	0.8	0.1	0.8	22	9	0.3	15	1.6	4.1	0.3	0	0.5	5.5	0.9	0.3	0.4	0.3	0.9	0
EnhA10	0.39	0.2	0.1	2.4	7.4	0.2	0.5	0.5	21	7.3	0.3	18	1.4	1.6	0.1	0	0.1	3	0.7	0.1	0.2	0.1	0.5	0.1
EnhA14	0.37	0.1	0	0.2	0.3	0.5	1.1	0.7	2.3	2.1	2.1	2	0.3	1.9	0.6	0.9	0.1	1.9	0.5	0.3	0.8	0.2	0.5	0
BivProm1	0.15	157	233	151	139	34	5.5	128	0.8	53	114	10	131	101	56	60	185	77	88	188	161	171	105	214
BivProm2	0.16	169	175	71	111	12	4.2	104	1.2	61	99	7.6	78	52	22	24	189	21	92	176	169	199	117	236
BivProm3	0.29	13	3.8	6.3	4.1	14	12	17	1	14	19	5.4	9.2	5.4	11	12	8	6.7	15	20	21	12	9.8	7.7
PromF3	0.15	0.9	4.7	30	13	3.6	0.4	7.2	1.8	4.4	3.8	3	25	18	0.9	0.5	0.8	25	14	2.6	1.4	0.9	13	0.7
PromF4	0.19	1.1	6.6	22	9.6	1.1	0.6	4.3	1.1	1	1.4	0.6	14	37	1.8	1.1	1.2	21	14	1.6	0.6	0.4	14	0.6
PromF5	0.14	54	140	168	107	30	3.1	79	1.8	39	44	12	147	64	35	36	38	100	61	59	36	46	56	30
TSS1	0.12	5.1	12	36	11	2.8	0.9	9.5	0.9	1.5	1.6	1.3	27	57	5.7	4.6	2.7	36	21	9.5	3.3	2.1	19	3.8
TSS2	0.11	19	21	28	13	2.9	2.6	15	0	5.1	5.9	1.6	16	36	11	11	6.5	19	27	37	18	10	21	11

State	% genome	SUZ12 H1	SUZ12 H1	SUZ12 HEK293T	SUZ12 HepG2	SUZ12 K562	SUZ12 K562	SUZ12 MCF-7	SUZ12 NT2	EED GM12878	EED GM12878	EED GM12892	EED HepG2	EED K562	EED K562	EED endothelial_cell_of_umbilical_vei
GapArtf3	0.01	19	2	28	32	14	42	15	11	2.5	9.1	12	2.4	13	2	3.2
HET2	0.69	0.9	0.1	0.8	0.1	3.8	0.3	1	0	0.1	0.4	0	0.2	0	0.1	0
ReprPC1	0.19	109	100	3.5	1.2	29	3.2	2.8	100	0.5	0.7	0.1	0.6	1.3	1.6	0.1
ReprPC5	0.63	1.8	0.5	0.6	0.5	8	1.2	2.4	0.2	0.4	0.5	0.1	0.5	1.1	0.6	0
Acet1	0.18	76	0.4	9	2	2.7	3.3	36	0.1	0.7	2.2	0.2	2.3	1.9	2.8	0.1
EnhWk7	0.48	0	0	0.1	0	0	0.1	0.6	0	14	2.7	1.3	0.4	0.1	0.2	0
EnhA8	0.25	0.4	1	0.3	0.5	2.2	1.5	1.3	0.6	26	19	17	2.5	6.8	6.4	0.8
EnhA9	0.16	0.8	0.5	0.5	1.6	0.6	5.2	2.5	0.1	35	25	21	8.4	18	14	3
EnhA10	0.39	0	0.3	0.4	0.4	0.4	1.5	1.7	0.1	15	3.3	3	4.5	4.8	8.2	0.4
EnhA14	0.37	0.2	0.2	0.3	1.5	1.8	0.8	2	0.1	1.5	1.3	0.2	23	5.5	4.5	0.1
BivProm1	0.15	106	126	5.5	14	94	19	16	155	7.1	24	5.7	3.7	7.6	7.3	5.1
BivProm2	0.16	180	142	8.7	6.4	66	11	5.4	202	1.9	5	1.2	2.1	3.5	4.6	0.7
BivProm3	0.29	2.7	11	0.6	2.2	13	5.9	3.2	11	1.5	1.9	0.4	3.7	3.5	3.4	0.3
PromF3	0.15	1.6	16	9.5	20	7.4	18	25	6	30	45	42	13	37	44	38
PromF4	0.19	0.4	15	46	118	18	90	54	3.8	45	117	204	23	102	85	197
PromF5	0.14	24	74	6	17	71	22	25	53	16	38	17	9.3	17	14	35
TSS1	0.12	1	23	75	246	25	176	70	11	33	128	182	23	79	52	236
TSS2	0.11	3	24	41	129	20	99	33	21	14	55	55	11	26	18	88

State rank

State rank
1
2
3

Neighborhood enrichments with PRC1 and PRC2: ranked states by descending enrichments

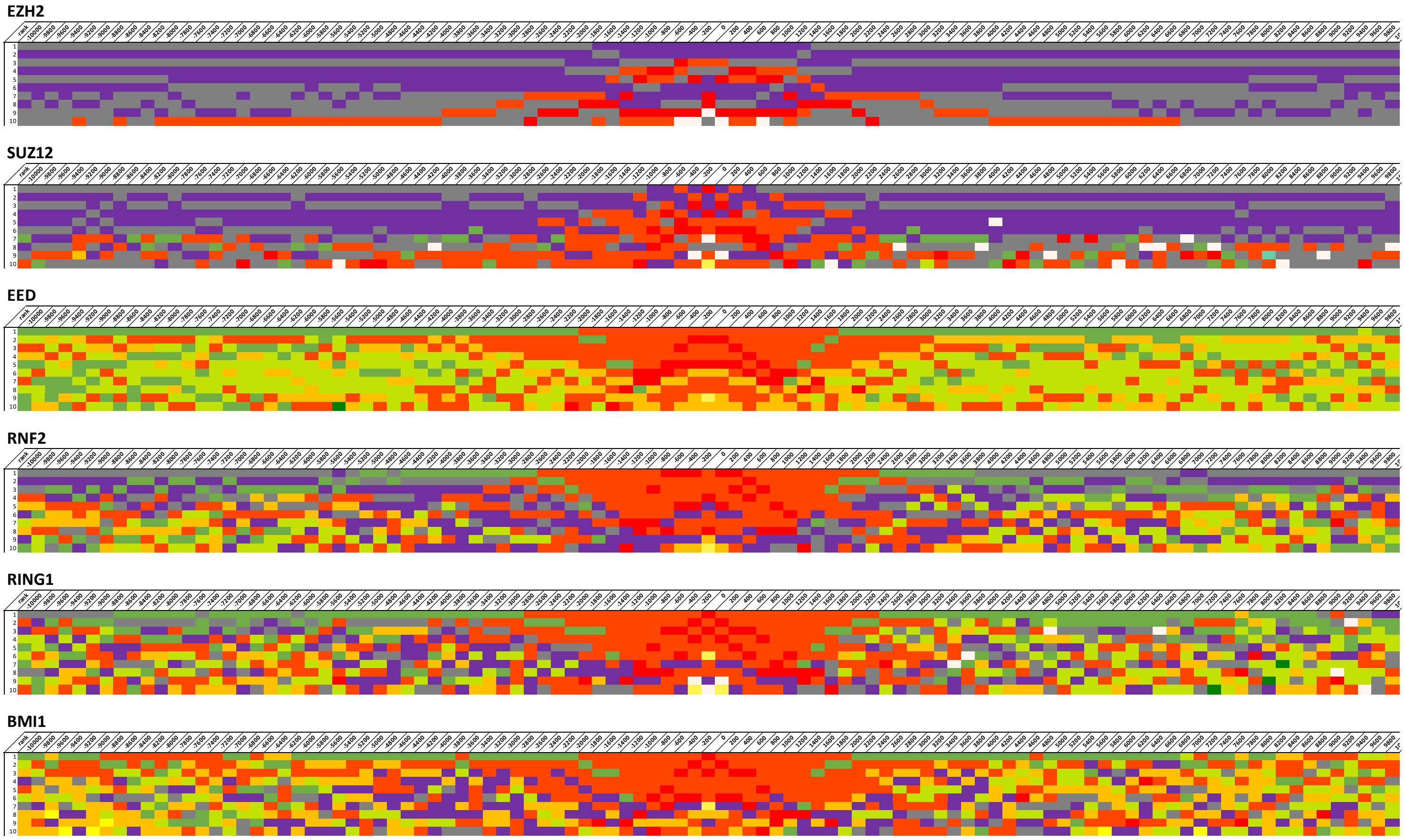

Figure S17: Enrichments of selected full-stack states with PRC1 and PRC2. (A) A subset of **Fig. S16** showing fold enrichment of full-stack states for binding sites of polycomb repressive protein complexes in different cell types from ENCODE (Methods). The column names highlighted in green (PRC1) and orange (PRC2) show the subunits of PRCs (BMI1, RING1 and RNF2 in PRC1, and EZH2, SUZ12, EED in PRC2) and the cell types where the PRCs were profiled in the second and first rows of column names, respectively. Each corresponding row corresponds to a state, and only states that were among the top three with greatest enrichments for at least one category of PRC complexes are shown. Top enrichment values are colored red based on the rank of the state for each score as indicated in the color legend at the bottom.

Some states that show strong consensus across cell types in enrichments with PRC1 and PRC2 include ReprPC1, BivProm1-2, PromF4-5, TSS1-2. ReprPC1 and BivProm1-2 all show strong signals of H3K27me3. (**B**) Neighborhood enrichments of full-stack states with binding sites of PRC1 and PRC2 complexes. In each subpanel, each column corresponds to a 200-bp bin across the 20,000-bp regions overlapping and surrounding annotated PRC1&2 subunit complexes. Within each column, the top 10 states most enriched at the corresponding 200-bp position (within the 20,000bp window) are shown, in descending order of enrichments, and colored based on the state groups as presented throughout the paper. **Additional File 4** accompanies this figure to show full state names and rankings.

hg19 state enrichments with hg19 genome contexts

state	% Genome	GENCODE_coding	RefSeqExon	RefSeqGene	RefSeqTES	CpGIsland	RefSeqTSS	RefSeqTSS2kb	assembly_gap	phastCons_elements	pseudogene	repeat_elements	znf_genes
GapArtf1	11.86	0.23	0.19	0.27	0.31	0.26	0.25	0.27	8.43	0.21	1.59	0.56	0.21
GapArtf2	0.05	0.26	0.33	0.15	0.42	1.25	1.6	0.8	0.02	0.35	1.65	1.85	1.44
GapArtf3	0.012	0.69	0.91	0.3	2.03	5.01	4.87	2.23	0.01	0.71	1.74	1.7	1.26
Quies1	9.883	0.05	0.09	0.54	0.19	0.02	0.06	0.13	0	0.7	0.42	1.03	0.4
Quies2	3.07	0.15	0.17	0.56	0.22	0.04	0.19	0.17	0	0.58	0.52	1.18	0.29
Quies3	12.23	0.22	0.39	1.01	0.76	0.07	0.14	0.44	0	0.82	1.24	1.19	0.55
Quies4	4.452	0.07	0.12	0.59	0.28	0.09	0.11	0.21	0	0.35	1.46	1.73	0.8
Quies5	1.692	0.22	0.33	0.7	0.62	0.06	0.17	0.41	0	1.21	0.85	1.26	0.2
HET1	0.706	0.43	0.34	0.55	0.2	0.29	0.53	0.28	0	0.47	0.95	1.41	0.33
HET2	0.692	1.01	0.79	0.79	0.42	1.25	0.54	0.52	0	0.72	0.58	0.61	0.66
HET3	1.359	0.08	0.16	0.79	0.38	0	0.06	0.17	0	0.31	0.72	1.86	1.77
HET4	0.563	0.23	0.47	0.57	0.74	0	0.08	0.21	0	0.33	1.35	1.87	6.03
HET5	0.249	1.76	1.95	0.8	1.42	1.28	0.65	0.86	0	0.59	2.52	1.58	14
HET6	0.581	0.86	0.67	0.48	0.63	1.28	0.86	0.65	0	0.46	2.85	1.66	2.58
HET7	1.023	0.36	0.35	0.68	0.4	0.64	0.43	0.41	0	0.32	1.63	1.78	2.04
HET8	0.435	0.89	0.72	0.64	0.6	0.47	0.71	0.91	0	0.47	1.57	1.44	0.87
HET9	0.998	0.35	0.37	0.42	0.75	0.02	0.44	0.6	0	0.51	3.53	1.49	1.44
ReprPC1	0.191	2.91	3.07	1.05	3.68	8.86	3.04	8.51	0	2.51	0.89	0.37	0.3
ReprPC2	0.325	0.87	1.4	0.87	2.72	0.52	0.77	5.5	0	1.66	1.19	0.78	0.28
ReprPC3	1.107	0.55	0.68	0.71	1.06	0	0.4	1.14	0	1.16	1.39	1	0.36
ReprPC4	3.935	0.32	0.41	0.69	0.77	0.03	0.21	0.55	0	0.92	1.21	1.16	0.29
ReprPC5	0.628	2.25	1.77	0.84	1.26	1.02	1.24	1.45	0	0.98	1.34	0.68	0.38
ReprPC6	1.513	1.42	1.18	0.85	1.22	0.3	0.62	0.77	0	0.79	1.5	0.91	0.32
ReprPC7	0.614	3.99	2.85	1.21	1.54	1.56	1.64	1.54	0	1.31	0.9	0.4	0.61
ReprPC8	0.477	1.07	0.94	0.8	0.77	0.07	0.57	0.73	0	1.34	1.11	1.12	0.37
ReprPC9	0.375	0.43	0.78	0.79	1.64	0.44	0.36	3.03	0	1.12	1.26	1.02	0.53
Acet1	0.184	2.58	1.68	1.09	0.47	3.23	1.75	1.1	0	1.06	1.64	1.24	1.09
Acet2	0.855	0.77	0.59	0.83	0.25	0.11	0.52	0.55	0	0.81	1.07	1.01	0.37
Acet3	2.649	0.36	0.36	0.83	0.34	0.05	0.24	0.45	0	0.54	1.02	1.29	0.4
Acet4	0.403	0.76	0.81	0.99	0.56	0.1	0.66	0.93	0	0.78	0.82	0.83	0.38
Acet5	0.86	0.29	0.4	0.89	0.48	0.01	0.28	0.48	0	0.75	0.86	1.06	0.32
Acet6	0.428	0.36	0.56	1.04	0.76	0	0.47	0.63	0	1.13	0.68	0.87	0.46
Acet7	0.285	0.63	0.89	1.13	0.61	0.13	0.98	1.29	0	1.25	0.58	0.64	0.39
Acet8	0.562	0.53	0.58	0.76	0.53	0.21	0.58	0.81	0	0.58	0.89	1.09	0.31
EnhWk1	1.54	0.18	0.39	0.95	0.76	0.01	0.16	0.38	0	1.57	0.7	0.81	0.59
EnhWk2	0.352	1.95	2.03	1.64	1.76	0.77	1.85	2.21	0	1.39	0.36	0.42	0.66
EnhWk3	0.83	0.48	0.73	1.25	1.03	0.07	0.69	1.15	0	1.88	0.57	0.69	0.72
EnhWk4	2.216	0.19	0.38	1.12	0.68	0.02	0.22	0.45	0	2.31	0.48	0.66	0.59
EnhWk5	0.994	0.31	0.54	1.04	0.99	0.05	0.27	1.11	0	0.98	0.95	1.06	0.6
EnhWk6	0.588	0.52	0.87	1.46	1.58	0.04	0.41	1.58	0	0.96	0.65	1	0.72
EnhWk7	0.484	0.38	0.55	1.12	0.99	0.05	0.32	0.97	0	0.94	0.84	1.06	0.5
EnhWk8	1.369	0.19	0.35	0.94	0.61	0.01	0.22	0.35	0	1.66	0.57	0.85	0.42
EnhA1	0.179	1.37	1.9	1.67	2.1	0.69	3.72	3.3	0	2.32	0.33	0.32	0.73
EnhA2	0.328	0.47	0.94	1.31	1.2	0.13	1.97	1.72	0	3.36	0.54	0.42	0.8
EnhA3	0.194	0.41	0.87	1.61	1.4	0.01	1.98	2.22	0	3.05	0.46	0.43	0.82
EnhA4	0.301	0.34	0.68	1.14	1.05	0	0.91	0.91	0	2.35	0.61	0.6	0.54
EnhA5	0.714	0.29	0.58	1.09	1.16	0.01	0.43	0.62	0	2.2	0.66	0.68	0.55
EnhA6	0.563	0.74	1.01	1.61	1.19	0.14	0.93	1.31	0	1.85	0.43	0.65	0.91
EnhA7	0.393	2.15	1.83	1.07	1.32	0.58	1.97	2.48	0	0.94	0.89	0.71	0.5
EnhA8	0.255	1.57	1.78	1.45	1.76	0.57	4.94	5.54	0	1.27	0.55	0.67	0.71
EnhA9	0.162	1.24	1.78	1.86	2.12	0.39	2.78	4.24	0	1.44	0.23	0.5	0.89
EnhA10	0.395	0.42	0.89	1.19	1.39	0.21	1.41	4.58	0	1.48	0.74	0.8	0.92
EnhA11	0.715	0.4	0.67	0.98	1.15	0.03	0.58	1.31	0	0.81	1	1.14	0.73
EnhA12	0.332	1	1.08	0.92	0.9	0.22	1.76	1.61	0	1.17	0.74	0.75	0.31
EnhA13	0.764	0.29	0.42	0.88	0.69	0.01	0.43	0.72	0	1.06	0.76	0.96	0.27
EnhA14	0.366	1.09	1.22	1.14	1.28	0.18	2.84	3.02	0	1.16	0.74	0.78	0.34
EnhA15	1.017	0.43	0.6	1.02	1.06	0.03	0.72	1.42	0	1.05	0.95	1.01	0.42
EnhA16	0.645	0.42	0.59	0.91	1.01	0.37	0.89	2.09	0	1.01	1.34	1.04	0.41
EnhA17	0.526	0.3	0.65	1.36	1	0.05	0.82	1.15	0	3.15	0.45	0.53	0.94
EnhA18	0.457	0.47	0.54	0.85	0.61	0.14	0.84	0.85	0	1.23	0.7	1.03	0.49
EnhA19	0.257	1.05	1.27	1.32	0.91	0.43	2.25	1.99	0	1.95	0.47	0.61	1.12
EnhA20	0.346	0.28	0.49	1.08	0.96	0.01	0.19	0.52	0	1.53	0.72	0.76	0.77
TxEnh1	0.391	0.35	0.6	2.3	0.69	0	0.35	1.2	0	1.64	0.31	0.65	1.14
TxEnh2	0.391	0.37	0.45	2.3	0.5	0.01	0.43	2.74	0	1.08	0.27	0.85	1.95
TxEnh3	0.25	1.55	1.14	2.31	0.65	0.58	0.69	1.27	0	0.86	0.68	1.01	3.15
TxEnh4	0.267	14.2	10.4	2.26	6.15	2.58	1.3	1.62	0	3.37	0.51	0.37	1.47
TxEnh5	0.498	9.39	7.17	2.18	3.79	2.76	1.03	1.17	0	2.25	0.56	0.5	1.42
TxEnh6	0.189	6.29	5.96	2.21	5.84	0.7	0.94	1.49	0	1.81	0.46	0.61	1.14
TxEnh7	0.27	1.02	0.99	2.28	0.73	0.42	1.09	1.62	0	1.11	0.26	0.55	1.28
TxEnh8	0.243	4.84	4.96	2.22	4.27	0.23	0.82	0.81	0	2.11	0.48	0.56	1.22
TxWk1	2.8	1.39	2.28	2.15	3.09	0.08	0.18	0.35	0	1.16	0.88	1.11	1.71
TxWk2	0.842	6.61	4.8	2.11	2.51	1.58	0.71	0.72	0	1.66	0.87	0.77	1.27
Tx1	0.824	0.2	0.21	2.31	0.3	0.01	0.18	0.67	0	0.85	0.38	1.15	2.24
Tx2	1.58	0.18	0.25	2.27	0.35	0.04	0.15	0.48	0	0.84	0.61	1.17	1.74
Tx3	0.508	0.3	0.36	2.26	0.29	0.19	0.39	0.63	0	0.57	0.48	1.04	1.64
Tx4	0.47	2.66	2.15	2.31	1.38	0.04	0.39	0.65	0	1.33	0.6	1.01	2.7
Tx5	0.942	2.89	2.87	2.21	2.65	0.48	0.23	0.45	0	0.95	0.96	1.35	2.17
Tx6	1.109	3.6	4.39	2.27	4.91	0	0.14	0.25	0	2.03	0.4	0.69	1.71
Tx7	0.819	6.81	5.82	2.29	4.92	0	0.28	0.37	0	2.46	0.38	0.67	1.91
Tx8	0.681	2.51	3.51	2.2	4.03	0.02	0.34	0.57	0	1.74	0.51	0.74	1.29
TxEx1	0.265	10.4	6.47	2.32	3.88	0.6	1.27	1.3	0	2.9	0.5	0.72	2.61
TxEx2	0.556	15.8	9.97	2.32	4.1	1.19	0.73	0.76	0	3.65	0.57	0.54	2.45
TxEx3	0.663	9.74	7.04	2.25	4.26	2.73	0.6	0.78	0	2.21	0.9	0.83	2.6
TxEx4	0.098	10.7	7.83	2.26	9.28	1.99	4	8.1	0	3.09	0.37	0.41	2.17
znf1	0.406	1.06	1.22	2.13	1.39	0.3	0.23	0.4	0	0.6	1.33	1.67	20.8
znf2	0.152	10.4	7.13	2.14	3.28	0.92	0.93	0.84	0	1.16	1.88	1.21	68.6
DNase1	0.201	0.99	1.51	1.07	2.42	3.59	5.59	1.88	0	2.04	1.1	0.53	0.8
BivProm1	0.145	9.39	8.49	1.43	2.57	77	28.7	19.3	0	3.94	1.02	0.16	0.87
BivProm2	0.159	7.56	6.41	1.32	4.03	54.5	14.8	14.9	0	3.63	1.03	0.18	0.5
BivProm3	0.286	2.95	2.8	1.16	2.23	7.19	6.18	7.02	0	2.47	0.8	0.42	0.82
BivProm4	0.13	2.55	3.39	1.74	2.99	3.75	8.11	14.5	0	3.91	0.6	0.3	1.28
PromF1	0.201	2.16	2.41	1	2.35	2.6	6.51	6.46	0	1.85	0.63	0.45	0.81
PromF2	0.138	2.4	3.21	1.38	4.35	3.88	8.11	13.9	0	2.34	0.46	0.39	1.23
PromF3	0.153	4.63	5.09	1.52	4.37	25.3	13.3	25.7	0	2.19	0.53	0.31	3.39
PromF4	0.19	10.2	13.5	1.89	2.52	96.7	62.2	28	0	4.13	0.57	0.12	4.77
PromF5	0.135	7.33	8.49	1.56	2.42	71	31.9	22.4	0	3.95	0.72	0.16	2.27
PromF6	0.129	3.69	3.25	2.1	3.64	1.55	2.74	26.4	0	1.95	0.61	0.48	4.34
PromF7	0.16	1.72	2.04	2.08	2.5	0.09	1.05	20.4	0	1.53	0.71	0.68	4.41
TSS1	0.123	6.48	12.6	1.44	2.63	101	152	25.9	0	4.89	0.95	0.29	3.75
TSS2	0.114	5.53	8.12	1.15	2.44	75.4	106	20.1	0	4.46	1.74	0.57	2.13
	100	1.15	2.45	42.1	0.001	0.68	0.001	3.41	7.57	5.17	1.94	46.7	0.73

hg38 (liftedOver) state enrichments with hg38 genome contexts

state	% Genome	GENCODE_coding	RefSeqExon	RefSeqGene	RefSeqTES	CpGIsland	RefSeqTSS	RefSeqTSS2kb	assembly_gap	phastCons_elements	pseudogene	repeat_elements	znf_genes
GapArtf1	4.885	0.38	0.39	0.55	0.69	0.57	0.53	0.58	19.9	0.44	3.33	1.29	0.37
GapArtf2	0.053	0.24	0.3	0.14	0.39	1.18	1.45	0.72	0	0.8	1.59	1.4	1.37
GapArtf3	0.012	0.86	0.84	0.28	1.98	4.59	4.25	2.05	0	0.89	1.67	1.39	1.24
Quies1	10.66	0.04	0.08	0.5	0.18	0.02	0.06	0.12	0	0.66	0.4	0.95	0.37
Quies2	3.314	0.14	0.16	0.52	0.21	0.03	0.18	0.16	0	0.55	0.5	1.1	0.27
Quies3	13.17	0.21	0.36	0.94	0.71	0.08	0.12	0.41	0.02	0.76	1.17	1.11	0.52
Quies4	4.795	0.07	0.11	0.55	0.27	0.08	0.11	0.19	0	0.32	1.41	1.6	0.75
Quies5	1.82	0.21	0.3	0.66	0.59	0.05	0.16	0.38	0	1.13	0.78	1.17	0.19
HET1	0.762	0.4	0.32	0.51	0.18	0.27	0.5	0.26	0	0.43	0.91	1.31	0.31
HET2	0.746	0.94	0.73	0.75	0.38	1.17	0.5	0.48	0	0.66	0.55	0.57	0.62
HET3	1.466	0.07	0.15	0.74	0.35	0	0.07	0.16	0	0.29	0.69	1.72	1.65
HET4	0.606	0.21	0.43	0.52	0.69	0	0.08	0.2	0	0.28	1.3	1.73	5.66
HET5	0.267	1.65	1.83	0.75	1.33	1.17	0.62	0.81	0	0.49	2.39	1.46	13.1
HET6	0.627	0.8	0.62	0.45	0.58	1.13	0.83	0.61	0.99	0.37	2.75	1.52	2.41
HET7	1.103	0.34	0.33	0.63	0.38	0.59	0.41	0.38	0	0.28	1.57	1.64	1.9
HET8	0.47	0.83	0.67	0.59	0.56	0.43	0.64	0.84	0.66	0.42	1.49	1.33	0.81
HET9	1.069	0.33	0.35	0.39	0.71	0.02	0.41	0.56	0	0.46	3.41	1.38	1.35
ReprPC1	0.206	2.71	2.84	0.98	3.37	7.93	2.82	7.89	0	2.34	0.85	0.35	0.28
ReprPC2	0.353	0.81	1.29	0.8	2.5	0.74	0.71	5.08	0.88	1.54	1.18	0.72	0.26
ReprPC3	1.194	0.52	0.63	0.65	0.98	0	0.38	1.06	0	1.09	1.34	0.94	0.34
ReprPC4	4.243	0.3	0.38	0.64	0.71	0.03	0.19	0.51	0	0.87	1.16	1.08	0.27
ReprPC5	0.677	2.11	1.65	0.79	1.17	0.94	1.15	1.34	0	0.91	1.31	0.63	0.36
ReprPC6	1.631	1.33	1.09	0.79	1.13	0.28	0.58	0.72	0	0.73	1.45	0.85	0.3
ReprPC7	0.662	3.73	2.65	1.13	1.43	1.45	1.51	1.43	0	1.21	0.86	0.38	0.57
ReprPC8	0.514	1	0.88	0.74	0.71	0.06	0.53	0.68	0	1.25	1.02	1.04	0.35
ReprPC9	0.403	0.4	0.73	0.73	1.54	0.4	0.33	2.81	0	1.04	1.19	0.95	0.5
Acet1	0.204	2.37	1.55	1.05	0.46	3.1	1.57	0.97	3.04	0.81	1.54	1.04	1
Acet2	0.922	0.72	0.55	0.77	0.23	0.11	0.49	0.51	0	0.75	1.03	0.94	0.34
Acet3	2.856	0.34	0.34	0.77	0.32	0.05	0.23	0.42	0	0.5	0.97	1.2	0.37
Acet4	0.434	0.71	0.75	0.92	0.51	0.1	0.63	0.87	0	0.73	0.78	0.78	0.36
Acet5	0.927	0.27	0.37	0.82	0.44	0.01	0.26	0.45	0	0.71	0.82	0.99	0.3
Acet6	0.462	0.34	0.52	0.96	0.7	0	0.43	0.59	0	1.06	0.64	0.81	0.43
Acet7	0.307	0.59	0.82	1.06	0.58	0.11	0.91	1.2	0	1.19	0.55	0.6	0.37
Acet8	0.606	0.49	0.54	0.71	0.49	0.18	0.53	0.74	0	0.54	0.85	1.02	0.29
EnhWk1	1.661	0.17	0.36	0.88	0.7	0.01	0.15	0.35	0	1.47	0.65	0.76	0.55
EnhWk2	0.38	1.82	1.89	1.53	1.63	0.73	1.7	2.05	0	1.29	0.33	0.4	0.62
EnhWk3	0.895	0.44	0.68	1.16	0.95	0.07	0.65	1.07	0	1.78	0.55	0.65	0.68
EnhWk4	2.39	0.17	0.35	1.05	0.63	0.01	0.2	0.42	0	2.18	0.46	0.63	0.55
EnhWk5	1.069	0.29	0.5	0.97	0.92	0.05	0.27	1.04	0	0.91	0.91	0.99	0.56
EnhWk6	0.721	0.47	0.78	1.25	1.37	0.11	0.39	1.36	0	0.85	1.83	0.88	0.59
EnhWk7	0.523	0.35	0.51	1.04	0.91	0.04	0.3	0.9	0	0.89	0.8	1	0.46
EnhWk8	1.476	0.18	0.33	0.87	0.58	0.01	0.21	0.32	0	1.57	0.54	0.8	0.39
EnhA1	0.193	1.28	1.76	1.57	1.95	0.65	3.47	3.07	0	2.18	0.23	0.31	0.69
EnhA2	0.354	0.43	0.87	1.22	1.11	0.11	1.84	1.6	0	3.19	0.5	0.41	0.75
EnhA3	0.209	0.38	0.81	1.5	1.3	0.01	1.84	2.07	0	2.88	0.39	0.42	0.77
EnhA4	0.324	0.32	0.64	1.06	0.97	0	0.83	0.84	0	2.22	0.57	0.57	0.5
EnhA5	0.77	0.27	0.53	1.01	1.07	0.01	0.39	0.57	0	2.07	0.62	0.65	0.52
EnhA6	0.607	0.7	0.93	1.51	1.1	0.13	0.87	1.22	0	1.75	0.41	0.62	0.85
EnhA7	0.423	2	1.71	1	1.2	0.55	1.79	2.3	0	0.87	0.87	0.67	0.47
EnhA8	0.274	1.46	1.66	1.35	1.63	0.51	4.61	5.15	0	1.18	0.52	0.63	0.66
EnhA9	0.175	1.16	1.66	1.73	1.95	0.36	2.6	3.96	0	1.35	0.22	0.48	0.83
EnhA10	0.425	0.39	0.83	1.11	1.3	0.18	1.31	4.27	0	1.39	0.72	0.76	0.86
EnhA11	0.77	0.38	0.62	0.91	1.05	0.04	0.52	1.21	0	0.76	0.93	1.06	0.68
EnhA12	0.358	0.94	1	0.85	0.84	0.2	1.63	1.49	0	1.1	0.73	0.71	0.29
EnhA13	0.824	0.27	0.39	0.81	0.65	0.01	0.41	0.67	0	1	0.73	0.9	0.26
EnhA14	0.395	1.02	1.13	1.07	1.19	0.18	2.64	2.81	0	1.09	0.71	0.73	0.32
EnhA15	1.097	0.4	0.56	0.95	0.99	0.03	0.66	1.32	0	0.98	0.91	0.95	0.4
EnhA16	0.695	0.39	0.55	0.84	0.94	0.38	0.83	1.95	0	0.94	1.26	0.97	0.38
EnhA17	0.567	0.28	0.61	1.27	0.91	0.04	0.77	1.07	0	2.98	0.42	0.51	0.88
EnhA18	0.497	0.43	0.5	0.78	0.58	0.23	0.79	0.78	1.25	1.16	0.67	0.96	0.45
EnhA19	0.277	0.99	1.19	1.24	0.84	0.39	2.08	1.85	0	1.84	0.42	0.57	1.05
EnhA20	0.373	0.26	0.45	1.01	0.9	0.01	0.17	0.47	0	1.44	0.66	0.71	0.72
TxEnh1	0.422	0.32	0.56	2.15	0.64	0	0.34	1.11	0	1.53	0.24	0.62	1.07
TxEnh2	0.422	0.34	0.41	2.16	0.46	0.01	0.41	2.55	0	1.01	0.26	0.8	1.82
TxEnh3	0.269	1.45	1.06	2.16	0.6	0.51	0.63	1.18	0	0.78	0.65	0.94	2.94
TxEnh4	0.288	13.3	9.69	2.11	5.69	2.4	1.22	1.51	0	3.16	0.42	0.34	1.37
TxEnh5	0.537	8.78	6.66	2.04	3.52	2.57	0.96	1.09	0	2.1	0.52	0.47	1.33
TxEnh6	0.204	5.89	5.54	2.07	5.41	0.65	0.87	1.38	0	1.69	0.42	0.58	1.06
TxEnh7	0.291	0.95	0.92	2.14	0.68	0.38	1.01	1.5	0	1.03	0.19	0.52	1.2
TxEnh8	0.262	4.52	4.59	2.08	3.95	0.2	0.76	0.75	0	1.98	0.38	0.53	1.14
TxWk1	3.019	1.3	2.12	2.01	2.87	0.08	0.17	0.33	0	1.07	0.81	1.04	1.6
TxWk2	0.909	6.2	4.46	1.97	2.32	1.46	0.66	0.66	0	1.55	0.82	0.72	1.19
Tx1	0.888	0.18	0.2	2.16	0.27	0.01	0.16	0.63	0	0.78	0.36	1.08	2.09
Tx2	1.704	0.17	0.23	2.13	0.33	0.04	0.14	0.45	0	0.78	0.55	1.09	1.62
Tx3	0.548	0.28	0.33	2.12	0.27	0.2	0.36	0.59	0	0.53	0.45	0.97	1.53
Tx4	0.507	2.48	2	2.16	1.3	0.04	0.39	0.61	0	1.24	0.57	0.94	2.52
Tx5	1.016	2.7	2.66	2.07	2.46	0.45	0.21	0.41	0	0.89	0.9	1.25	2.02
Tx6	1.196	3.37	4.08	2.12	4.56	0	0.14	0.23	0	1.88	0.36	0.65	1.6
Tx7	0.884	6.37	5.41	2.14	4.58	0	0.27	0.34	0	2.31	0.33	0.63	1.78
Tx8	0.735	2.35	3.26	2.06	3.72	0.02	0.32	0.53	0	1.63	0.45	0.69	1.2
TxEx1	0.286	9.69	6.01	2.17	3.63	0.54	1.2	1.22	0	2.73	0.45	0.68	2.43
TxEx2	0.6	14.8	9.27	2.17	3.79	1.06	0.68	0.71	0	3.45	0.52	0.51	2.29
TxEx3	0.716	9.11	6.54	2.1	3.95	2.5	0.56	0.73	0	2.07	0.82	0.77	2.43
TxEx4	0.106	10	7.28	2.12	8.75	1.81	3.83	7.55	0	2.88	0.31	0.39	2.02
znf1	0.438	0.99	1.14	1.99	1.31	0.28	0.22	0.37	0	0.53	1.26	1.54	19.4
znf2	0.164	9.69	6.63	2	3.04	0.92	0.85	0.78	0	0.97	1.77	1.12	64
DNase1	0.216	0.93	1.41	1	2.28	3.89	5.19	1.75	0	1.87	1.04	0.48	0.75
BivProm1	0.156	8.78	7.89	1.33	2.36	70.4	26.7	17.9	0	3.45	0.96	0.16	0.81
BivProm2	0.172	7.04	5.92	1.23	3.72	49.6	13.7	13.8	0	3.24	0.97	0.17	0.46
BivProm3	0.308	2.76	2.6	1.08	2.06	6.76	5.73	6.52	0	2.26	0.75	0.39	0.77
BivProm4	0.14	2.38	3.15	1.62	2.78	3.42	7.52	13.5	0	3.66	0.53	0.29	1.2
PromF1	0.217	2.02	2.24	0.94	2.17	2.43	6.07	6.02	0	1.69	0.6	0.42	0.76
PromF2	0.149	2.24	2.99	1.29	4.09	3.49	7.58	12.9	0	2.15	0.44	0.37	1.15
PromF3	0.165	4.33	4.73	1.42	4.15	23.4	12.4	23.9	0	1.98	0.48	0.3	3.17
PromF4	0.205	9.54	12.5	1.76	2.34	88.3	57.9	26.1	0	3.64	0.52	0.12	4.46
PromF5	0.146	6.85	7.89	1.45	2.2	65.1	29.6	20.8	0	3.48	0.65	0.15	2.12
PromF6	0.139	3.45	3.02	1.96	3.41	1.45	2.56	24.6	0	1.81	0.55	0.45	4.05
PromF7	0.173	1.61	1.89	1.95	2.3	0.08	0.98	18.9	0	1.41	0.66	0.64	4.12
TSS1	0.132	6.08	11.7	1.34	2.82	93.9	141	24.2	0	4.01	0.88	0.24	3.51
TSS2	0.122	5.19	7.58	1.07	2.54	74.2	98.8	18.7	0	3.46	1.57	0.43	2
	100	1.23	2.64	45	0	0.75	0	3.67	0.56	5.42	2.03	50.7	0.78

	GENCODE_coding	RefSeqExon	RefSeqGene	RefSeqTES	CpGIsland	RefSeqTSS	RefSeqTSS2kb	assembly_gap	phastCons_elements	pseudogene	repeat_elements	znf_genes	Avg. acros
Corr. Enr. Hg19 &hg38	1	1	1	1	1	1	1	0.98	0.99	0.93	0.97	1	**0.99**

Figure S18: Comparison of hg19 and hg38 full-stack states enrichments with annotated genomic contexts. The heatmaps show enrichment values for the full-stack states (rows) and different external genome annotations from **Fig. 3A** (columns) in hg19 (A) and hg38 (B) (**Methods**). Panel **(A)** is similar to **Fig. 3A**, but we present it here for better comparison with the hg38 enrichment heatmap. Results in **(B)** are based on (1) lifting over the full-stack annotation from hg19 to hg38 (**Methods**), and (2) doing enrichment analysis with annotated genome contexts derived from various databases in hg38 (**Methods**). In each heatmap, coloring of enrichments is column specific with highest and lowest enrichment values in each column are colored red and white, respectively. The first two columns of each heatmap show state labels and their percentage of genome coverage. The last row of each heatmap shows the percentage of genome coverage for each type of genome contexts. Below the heatmap in **(B)** is the correlation of the enrichments across states based on hg19 and hg38 for each corresponding annotation column as well as the average of them.

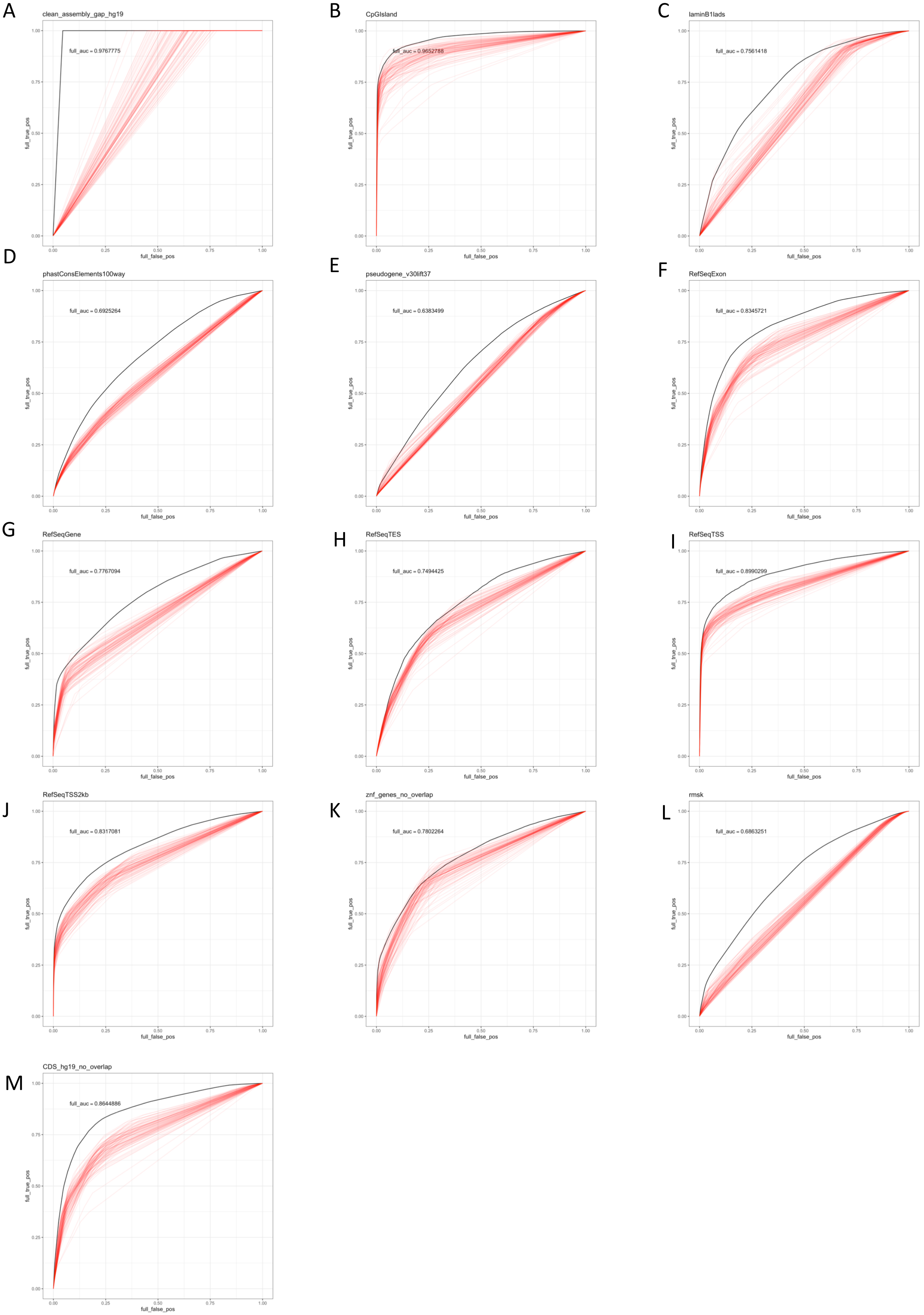
A
clean_assembly_gap_hg19
full_auc = 0.9767775
B
CpGIsland
full_auc = 0.9652788
C
laminB1lads
full_auc = 0.7561418
D
phastConsElements100way
full_auc = 0.6925264
E
pseudogene_v30lift37
full_auc = 0.6383499
F
RefSeqExon
full_auc = 0.8345721
G
RefSeqGene
full_auc = 0.7767094
H
RefSeqTES
full_auc = 0.7494425
I
RefSeqTSS
full_auc = 0.8990299
J
RefSeqTSS2kb
full_auc = 0.8317081
K
znf_genes_no_overlap
full_auc = 0.7802264
L
rmsk
full_auc = 0.6863251
M
CDS_hg19_no_overlap
full_auc = 0.8644886
full_true_pos
full_false_pos

Figure S19: ROC comparison of full-stack model annotations and the 18-state concatenated model annotations for predicting various external genomic annotations. Each panel shows the ROC curves from using the full-stack model annotations and 98 chromatin state annotations from a concatenated model to predict different external genomic annotations (**Methods**). The concatenated annotations are from a previously learned 18-state concatenated model (Kundaje et al., 2015). The full-stack annotations' ROC curves are in black, and 98 concatenated annotations' ROCs are in red. The respective genomic contexts for panels A-M are assembly gaps, CpG Islands, lamina associated domains (laminB1lads), phastCons elements, pseudogenes, exons, gene bodies, transcription end sites (TES), transcription start sites (TSS), 2kb regions surrounding transcription start sites (TSS2kb), ZNF genes, repeat elements in UCSC Genome Browser's repeatMasker track and coding sequences.

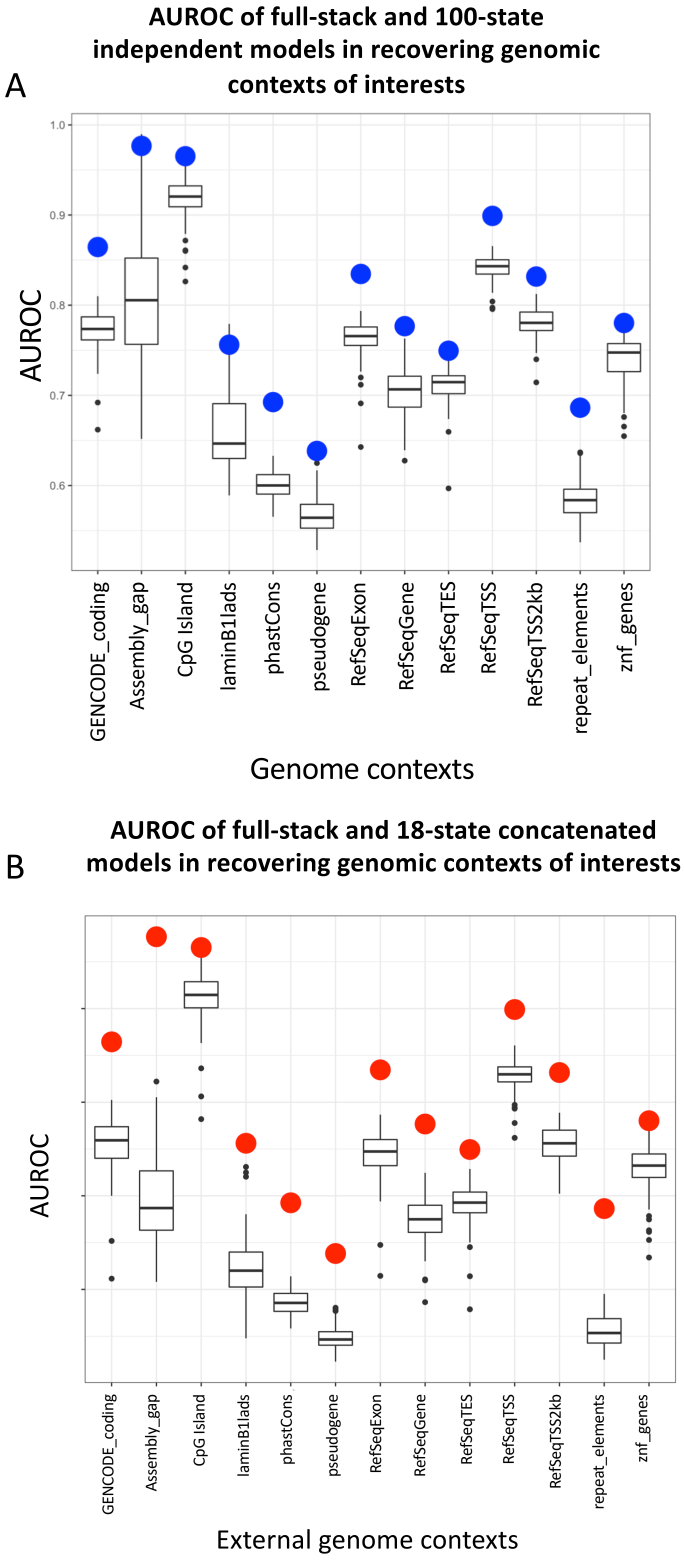

Figure S20: AUROC comparison of the full- stacked and the concatenated and independent chromatin state annotations at predicting various external genomics annotations. (A) AUROC values for ROC curves in **Fig. S19**. The x-axis represents different genomic contexts. The box-plots show AUROC of the 127 100-state annotations based on models learned independently in 127 cell types at predicting locations of the external annotations. The blue dots show the AUROC for the full-stack chromatin state annotations. **(B)** Similar to **(A),** but showing AUROC values for ROC curves in **Fig. S21**. but the boxplots show the AUROC values for 98 18-state annotations based on concatenated models in 98 cell types. The red dots show the AUROC for the full-stack chromatin state annotations.

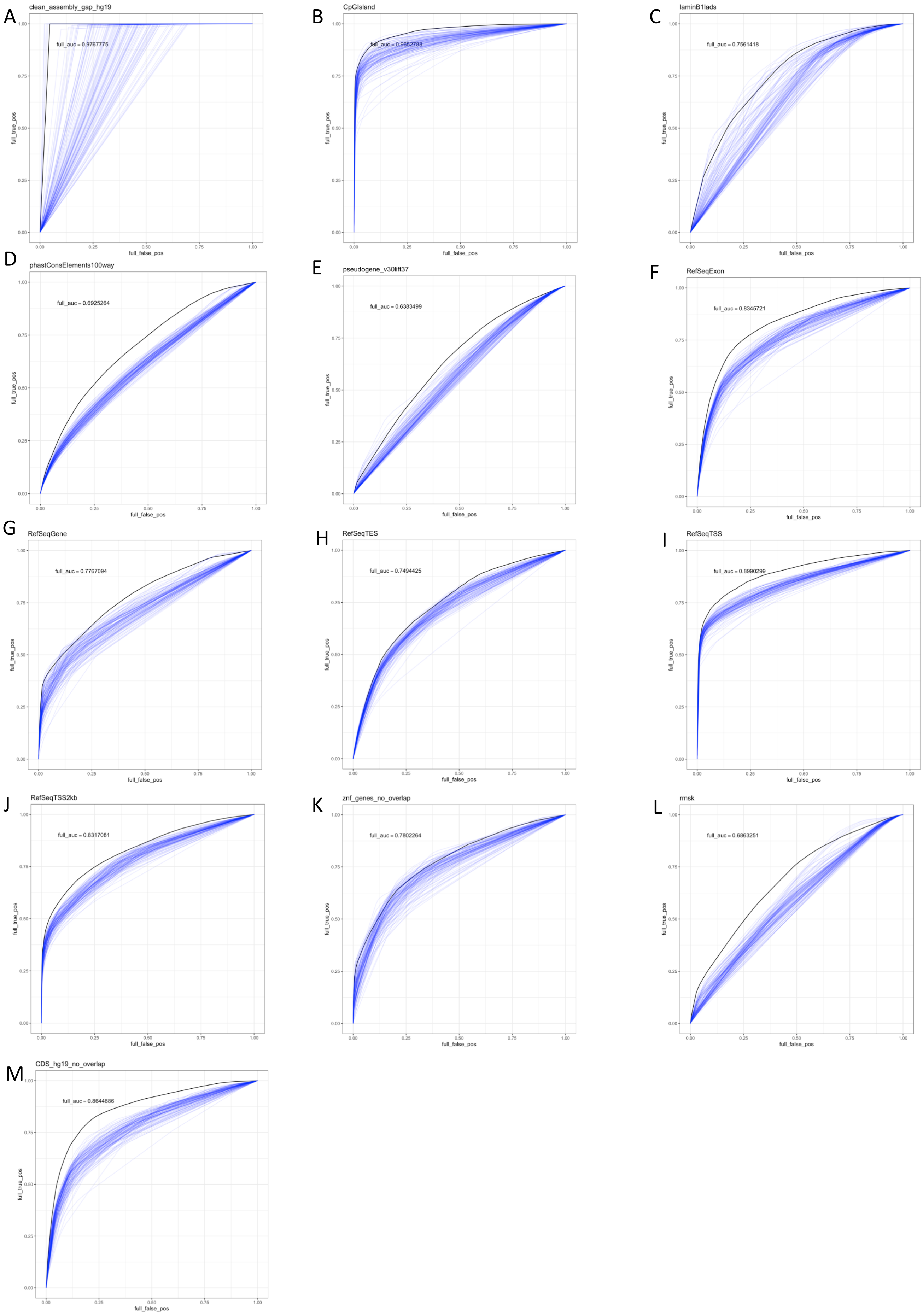

A
clean_assembly_gap_hg19
full_auc = 0.9767775
B
CpGIsland
full_auc = 0.9652788
C
laminB1lads
full_auc = 0.7561418
D
phastConsElements100way
full_auc = 0.6925264
E
pseudogene_v30lift37
full_auc = 0.6383499
F
RefSeqExon
full_auc = 0.8345721
G
RefSeqGene
full_auc = 0.7767094
H
RefSeqTES
full_auc = 0.7494425
I
RefSeqTSS
full_auc = 0.8990299
J
RefSeqTSS2kb
full_auc = 0.8317081
K
znf_genes_no_overlap
full_auc = 0.7802264
L
rmsk
full_auc = 0.6863251
M
CDS_hg19_no_overlap
full_auc = 0.8644886
full_true_pos
full_false_pos
0.00
0.25
0.50
0.75
1.00

Figure S21: ROC comparison of full-stack model annotations and the 100-state independent model annotations for predicting various external genomic annotations. Each panel shows the ROC curves from using the full-stack model annotations and the 127 independent model chromatin state annotations to predict different external genomic annotations (**Methods**). The independent models were 100 state models learned separately using all available data from each cell type. The full-stack annotations' ROC curves are in black, and independent annotations' ROCs are in blue. The respective genomic contexts for panels A-M are assembly gaps, CpG Islands, lamina associated domains (laminB1lads), PhastCons elements, pseudogenes, exons, gene bodies, transcription end sites (TES), transcription start sites (TSS), 2kb regions surrounding transcription start sites (TSS2kb), ZNF genes, repeat elements from all classes and families in UCSC Genome Browser's repeatMasker track and coding sequences.

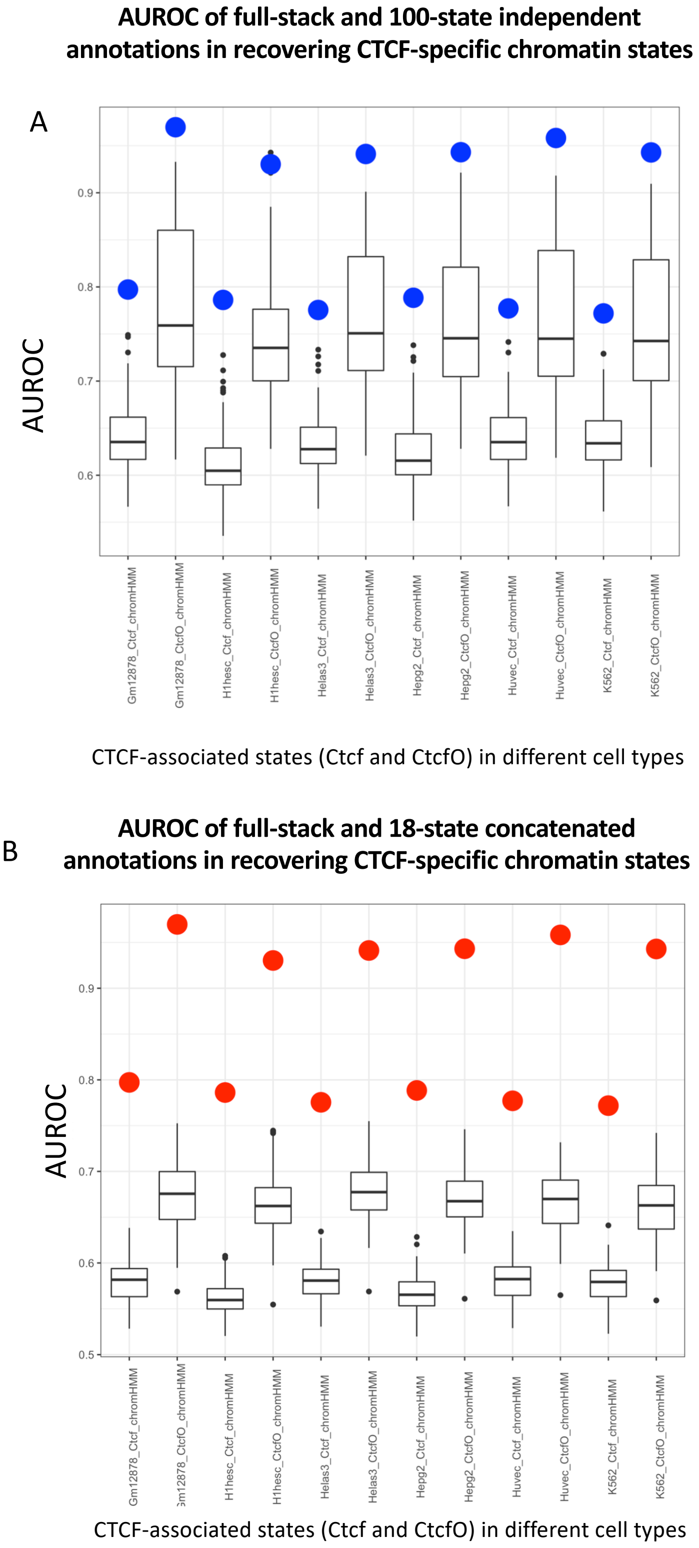

Figure S22: AUROC comparison of the full-stack and concatenated and independent chromatin state annotations at predicting CTCF-specific chromatin states. Box-plots show AUROC of (**A**) 127 100-state independent and (**B**) 98 18-state concatenated model annotations, which did not include CTCF, at predicting bases in sets of CTCF-associated chromatin states. In both panels, the x-axis represents sets of chromatin states associated with CTCF signal and limited histone modification signal in one of six cell types from a previously published chromatin state model that included CTCF (Hoffman et al., 2013) (**Methods**). CtcfO corresponds to a state that also had open chromatin signals, while state Ctcf lacked those signals. The dots colored (**A**) blue and (**B**) red show the AUROC for the full-stack chromatin state annotations, which were not trained using CTCF signals data.

Group	DNase	H3K27ac	H3K4me1	H3K4me2	H3K4me3	H3K9ac
weak enhancers	1.2	1.4	1.3	1.0	1.6	1.2
enhancers	1.0	1.1	1.0	0.9	1.4	1.0
TSS	0.1	0.3	0.7	0.2	0.1	0.4
promoters	0.3	0.3	0.4	0.2	0.2	0.3
bivalent promoters	0.5	0.6	0.4	0.4	0.5	0.5

Figure S23: Coefficient of variations of emission probabilities across different cell groups. Average coefficient of variations for the five enhancer and promoter state groups of full-stack states (rows) and six chromatin marks that are associated with enhancer and promoter activities**.** For a mark and state group combination, the coefficient of variation for the mark emission was computed separately for each state and then averaged among states in the group. The enhancer and weak enhancer group showed greater than two-fold higher coefficient of variations compared to the promoter group.

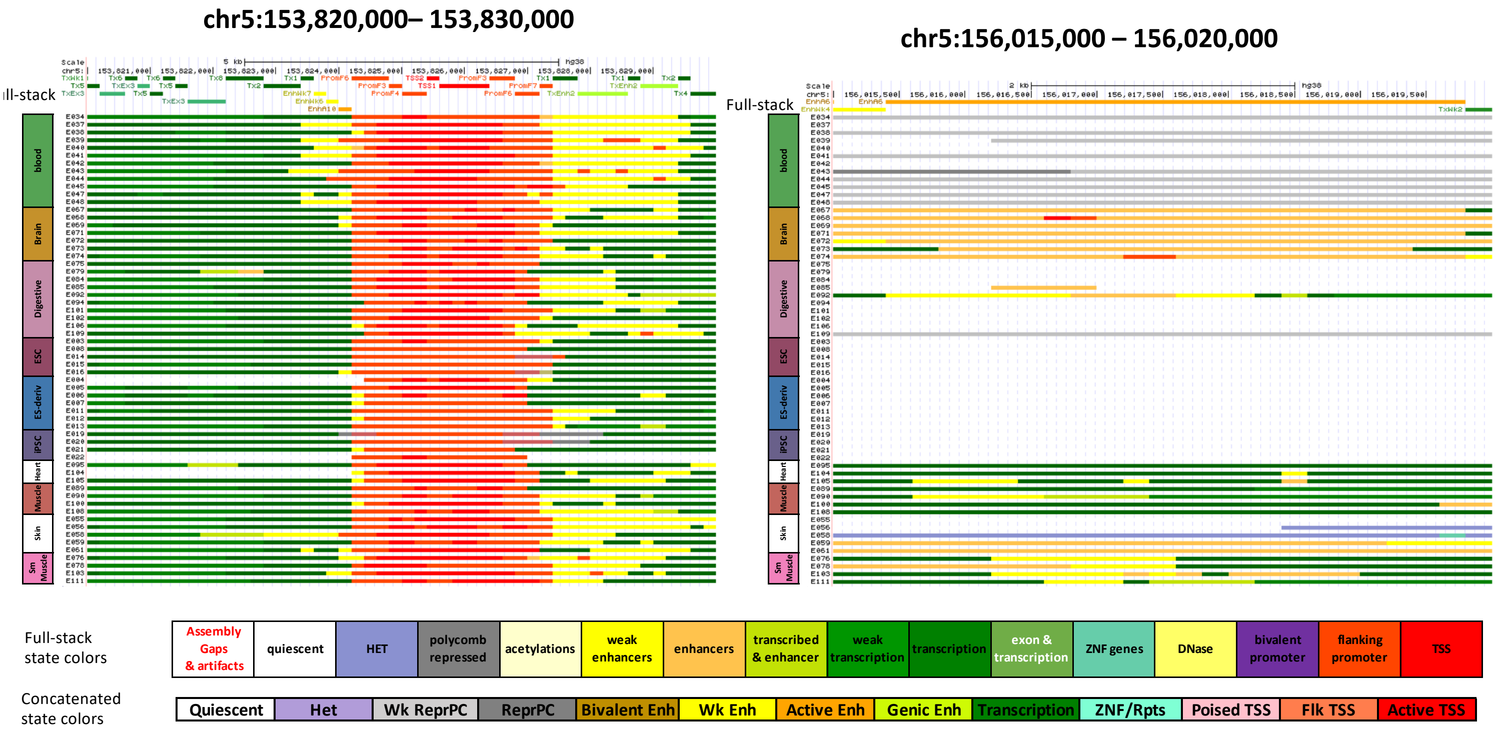

Figure S24: Illustration of the full-stack annotations at two distinct loci. Two loci representing regions that are in transcribed and active promoter states across cell types (left), and in an enhancer state specifically in brain (right). The loci correspond to those presented in **Fig. 1**. The top track shows the full-stack state annotations. The following tracks show concatenated annotations from 18-state models based on observed data (Kundaje et al., 2015). The cell types are ordered based on their associated cell groups. A color legend for the states is shown along the bottom.

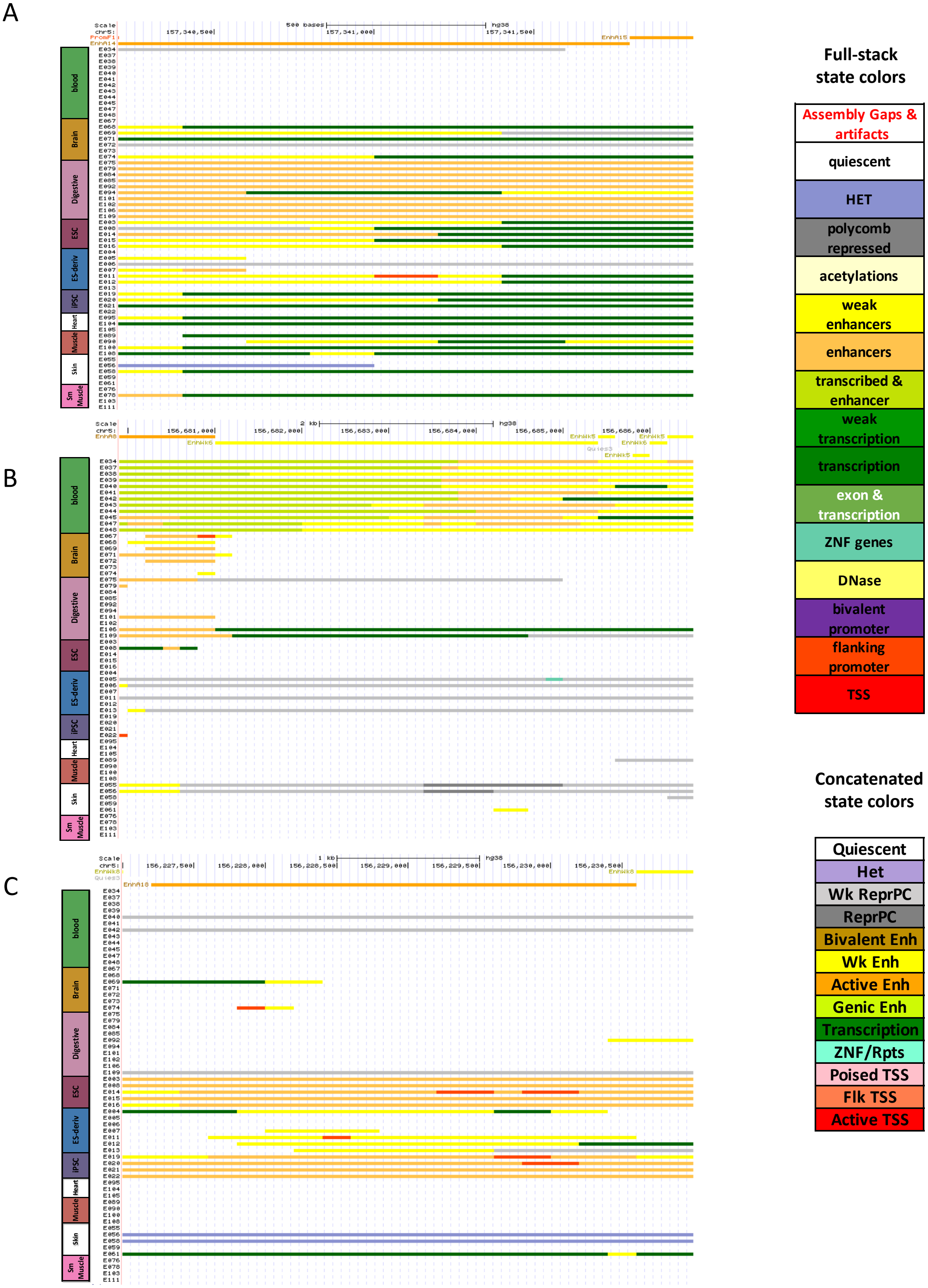

Figure S25: Illustration of full-stack cell-type-specific enhancer states. **(A-C)** The first track in each panel demonstrates the full-stack state annotation. Each of the following tracks show chromatin state annotations from a 18-state concatenated model (Kundaje et al., 2015). The individual reference epigenomes IDs and their tissue groups are labeled on left. The chromatin state coloring is labeled on right. **(A)** A genomic region (chr5:157340200-157342000) annotated to an active enhancer state in digestive cells in the full-stack model (EnhA14). **(B)** A genomic region (chr5:156679900-156686500) annotated to blood enhancer states in the full-stack model (EnhWk6 and EnhA8). **(C)** A genomic region (chr5:156227000-156231000) annotated as an ESC/iPSC-specific enhancer state in the full-stack model (state EnhA18).

Demonstration of the state transitions for full-stack state HET9

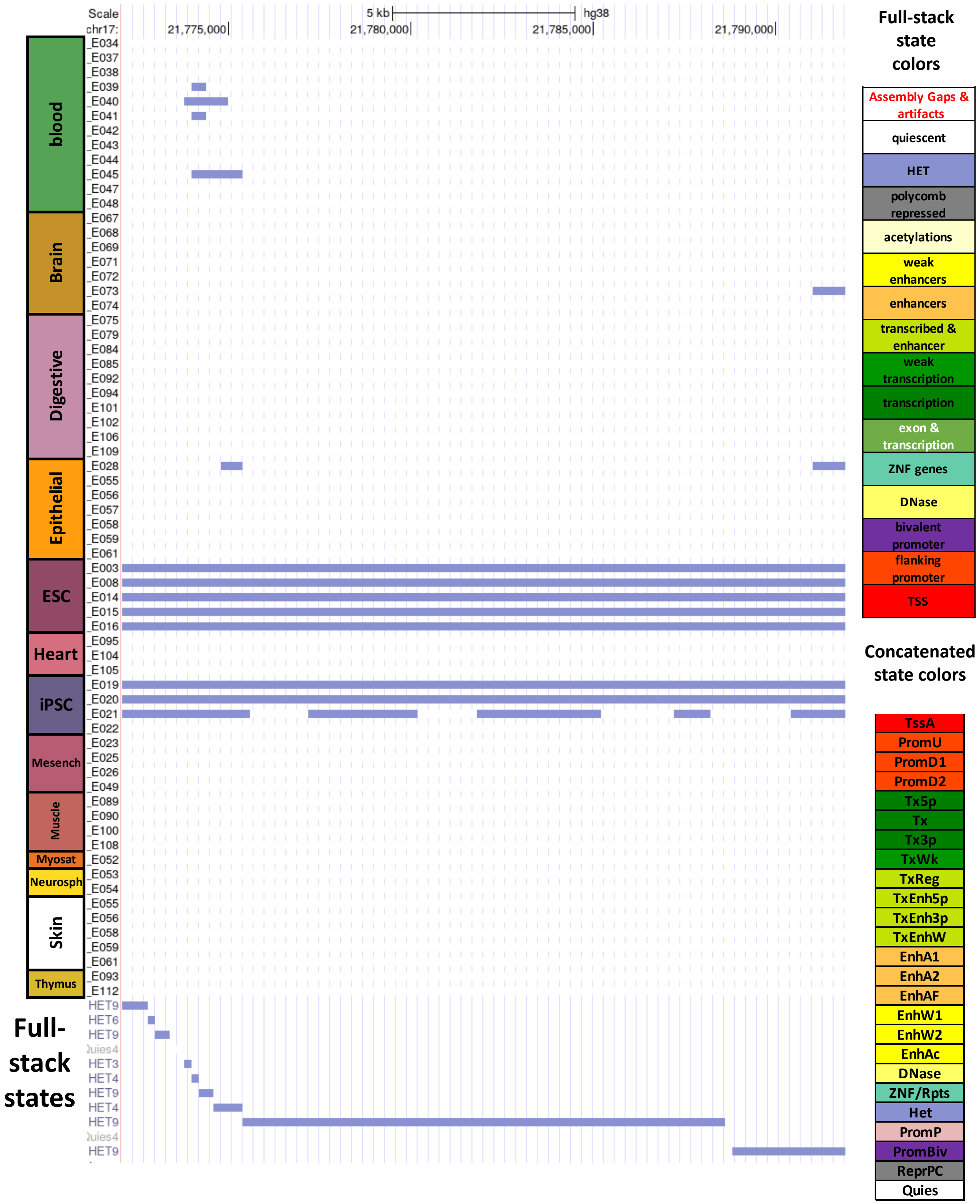

Figure S26: Illustration of full-stack heterochromatin state HET9. The figure captures the concatenated chromatin state maps for various reference epigenomes, and the corresponding full-stack chromatin state maps at region chr17:21772099-21791900. The first 66 tracks show chromatin state annotations from a 25-state concatenated model for 66 reference epigenomes (equivalently, in this paper, cell types) (Ernst & Kellis, 2015). The individual reference epigenomes IDs and their tissue groups are labeled on the left. The chromatin state colors are explained on the right. The last track, shown in full mode to display all state labels on the right, corresponds to the full-stack chromatin state map at this region. State HET9 is characterized, based on our analysis, as an ESC-group-related heterochromatin state (**Fig. 2C, Fig. S8-9, Additional File 3, 5**). Detailed characterizations of all full-stack states are in **Additional File 3**.

Demonstration of the state transitions for full-stack state PromF5

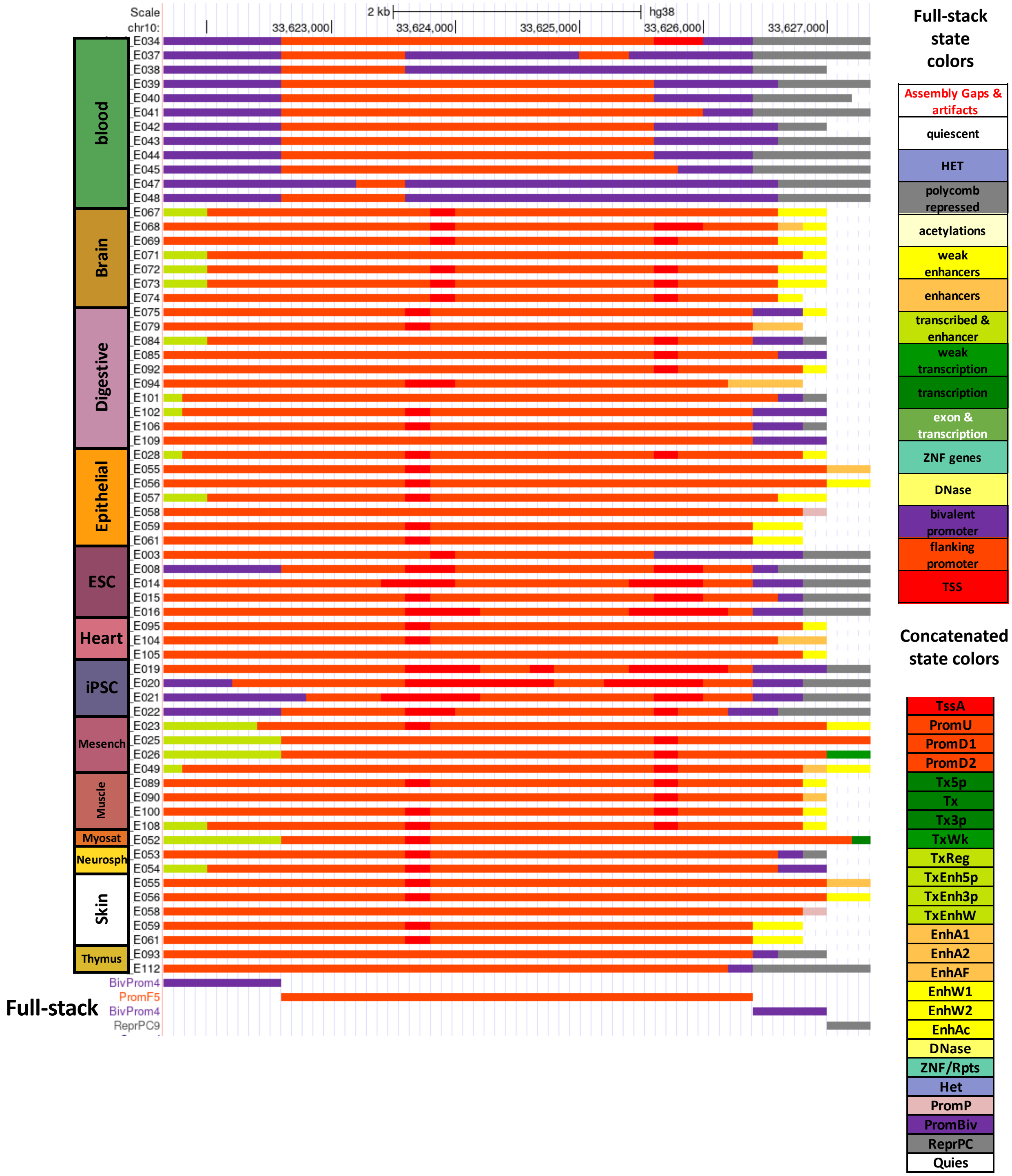

Figure S27: Illustration of full-stack flanking promoter state PromF5. The figure captures the concatenated chromatin state maps for various reference epigenomes, and the corresponding full-stack chromatin state maps at region chr10:33621649-33627350. The first 66 tracks show chromatin state annotations from a 25-state concatenated model for 66 reference epigenomes (equivalently, in this paper, cell types) (Ernst & Kellis, 2015). The individual reference epigenomes IDs and their tissue groups are labeled on the left. The chromatin state coloring is labeled on the right. The last track, shown in full mode to display all state labels on the left, corresponds to the full-stack chromatin state map at this region. State PromF5 is characterized, based on our various analyses as state frequently found at flanking promoter regions with some upstream bias, and sometimes, this state overlaps with regions of bivalent promoters in Blood-related and ESC-related groups (Blood & T cells, HSC & B cells, ESC, iPSC and ES-deriv) (**Fig. S 8-9, Additional File 3, 5**). Detailed characterization of all full-stack states are in **Additional File 3-5**.

A

Enrichments with different classes of repeats

state	Genome %	DNA?	DNA	LINE?	LINE	LTR?	LTR	Other	RC	RNA	SINE?	SINE	Satellite	Unknown?	Unknown	rRNA	scRNA	snRNA	srpRNA	tRNA
GapArtf1	11.9	0.2	0.2	0.0	0.9	0.0	0.3	2.4	0.2	0.1	0.3	0.2	1.3	0.1	0.1	0.3	0.2	0.4	0.4	0.6
GapArtf2	0.05	0.0	0.2	0.0	0.2	0.0	0.3	0.1	0.0	0.0	0.0	0.2	181.0	0.0	0.0	74.9	1.7	0.6	1.4	2.1
GapArtf3	0.01	0.0	0.2	0.0	0.2	0.0	0.3	0.0	0.0	0.0	0.0	0.3	144.8	0.0	0.0	579.7	0.0	1.4	0.0	4.1
Quies1	9.88	1.5	1.1	3.0	1.4	1.2	0.7	0.1	1.4	0.4	1.2	0.6	0.1	0.4	1.2	0.5	0.4	0.6	0.1	0.6
Quies2	3.07	0.9	1.2	3.8	0.9	2.6	2.4	0.0	1.0	1.2	0.5	0.9	0.3	4.7	1.3	1.1	0.2	0.5	0.9	0.8
Quies3	12.2	1.2	1.3	0.6	1.1	0.5	0.6	1.7	1.5	0.4	1.0	1.7	0.1	0.1	1.0	0.6	1.3	1.3	0.6	0.7
Quies4	4.45	0.2	0.8	0.0	2.8	0.0	1.3	4.5	0.5	0.1	0.1	0.6	1.6	0.2	0.4	0.5	0.5	0.7	0.2	0.5
Quies5	1.69	1.8	1.2	0.0	1.5	0.6	1.1	0.2	1.3	1.1	1.0	1.1	0.2	1.1	1.1	0.6	1.2	1.3	0.4	0.8
HET1	0.71	0.3	0.8	0.0	1.3	0.0	3.5	0.1	0.4	6.5	0.0	0.5	0.1	6.2	0.6	2.0	0.0	0.3	2.3	0.4
HET2	0.69	0.8	0.7	0.0	0.5	2.2	1.2	0.1	0.2	2.3	0.9	0.4	0.1	4.9	0.7	0.8	0.0	0.1	1.2	0.2
HET3	1.36	0.1	1.2	1.7	3.4	0.0	1.4	0.1	0.2	0.2	0.4	0.1	0.9	0.0	0.4	0.5	0.6	1.1	0.2	0.6
HET4	0.56	0.2	0.8	0.0	2.0	0.0	2.3	0.1	0.3	0.3	0.0	0.2	56.3	0.0	0.3	1.2	1.6	1.1	0.5	1.5
HET5	0.25	0.0	0.5	0.0	1.0	0.0	4.7	0.4	0.2	4.0	0.0	0.6	9.1	0.0	0.1	3.4	0.6	0.5	3.1	1.8
HET6	0.58	0.1	0.4	0.0	1.1	0.0	4.3	6.1	0.1	2.5	0.0	0.7	12.3	0.0	0.1	6.8	0.5	0.3	0.9	0.9
HET7	1.02	0.0	0.6	0.0	1.2	0.2	3.8	12.4	0.3	1.8	0.0	1.7	0.9	0.0	0.2	1.3	1.1	0.6	1.2	0.4
HET8	0.43	0.2	0.9	0.0	1.1	1.2	4.1	0.4	0.8	4.1	0.0	0.4	0.8	0.3	0.7	3.5	0.7	1.3	1.1	1.1
HET9	1	0.3	0.8	0.0	1.2	0.8	3.3	0.6	0.6	0.4	0.3	0.6	13.0	0.0	0.5	0.9	1.3	0.8	0.2	1.4
ReprPC1	0.19	0.8	0.3	0.0	0.3	0.0	0.3	0.0	0.0	1.6	0.0	0.5	0.2	0.0	0.2	0.6	1.0	1.1	0.0	8.8
ReprPC2	0.32	2.5	0.9	3.7	0.6	0.0	0.7	0.0	0.6	1.8	1.5	1.0	0.7	0.0	1.0	0.8	2.0	1.2	1.3	5.7
ReprPC3	1.11	2.0	1.2	5.8	1.0	1.5	1.3	0.0	1.7	1.8	2.4	0.8	0.6	1.6	1.8	1.3	1.3	1.6	0.9	1.8
ReprPC4	3.93	1.4	1.3	2.4	1.2	1.1	1.0	0.0	1.7	0.8	1.3	1.2	0.3	0.4	1.4	0.8	0.9	1.3	1.0	0.8
ReprPC5	0.63	0.7	0.6	0.0	0.5	0.3	1.1	0.0	0.3	2.4	0.0	0.7	0.2	3.4	0.5	1.3	0.3	0.3	1.8	1.4
ReprPC6	1.51	0.6	0.9	0.6	0.5	1.9	1.4	0.1	0.8	1.8	0.5	1.3	0.2	0.2	1.0	1.2	0.1	0.7	3.5	0.6
ReprPC7	0.61	0.2	0.5	0.0	0.3	1.4	0.6	0.1	0.2	0.6	0.8	0.4	0.1	1.5	0.5	1.7	0.0	0.1	0.3	0.3
ReprPC8	0.48	1.3	1.1	0.0	1.0	0.2	2.0	0.1	0.8	2.1	0.0	0.8	0.1	0.0	1.6	2.0	1.3	1.0	2.4	1.6
ReprPC9	0.37	2.4	1.2	0.0	0.8	0.0	1.2	0.1	1.2	0.2	1.4	1.2	1.0	0.9	1.3	0.8	1.2	1.0	2.0	1.2
Acet1	0.18	0.0	0.5	0.0	0.8	0.0	2.0	1.0	0.4	7.1	0.0	0.2	0.6	2.6	0.1	2.9	0.2	0.2	2.1	0.4
Acet2	0.85	0.5	1.1	0.8	0.7	0.0	2.2	0.1	0.9	9.8	0.5	0.7	0.0	6.4	1.4	1.8	0.6	0.7	6.5	0.9
Acet3	2.65	0.5	1.2	0.0	0.7	0.8	1.9	0.5	0.9	2.1	0.2	2.0	0.1	0.2	1.4	1.5	0.9	0.9	3.2	0.8
Acet4	0.4	0.2	0.9	0.0	0.5	1.5	1.4	0.1	0.8	3.9	1.5	0.9	0.0	0.8	0.9	0.9	0.3	0.7	2.4	0.7
Acet5	0.86	1.1	1.4	0.0	0.7	1.1	1.5	0.2	1.4	2.6	0.5	1.3	0.0	0.9	1.8	1.3	1.5	1.2	1.5	0.5
Acet6	0.43	1.8	1.3	1.6	0.6	0.0	1.2	0.0	1.9	1.9	0.8	1.0	0.0	2.0	2.0	0.9	1.4	0.9	0.3	0.7
Acet7	0.28	0.5	0.8	0.0	0.4	0.0	0.9	0.0	0.6	3.3	0.0	0.8	0.0	1.9	2.1	1.4	0.2	0.2	1.3	0.1
Acet8	0.56	0.4	0.9	0.9	0.5	0.0	2.0	0.3	0.8	0.7	1.0	1.6	0.0	2.4	1.0	0.9	0.9	0.5	5.1	0.6
EnhWk1	1.54	2.1	1.5	1.5	0.8	2.1	0.8	0.2	1.7	0.2	2.7	0.8	0.1	1.6	1.7	0.6	1.2	1.4	0.2	0.4
EnhWk2	0.35	1.1	0.5	0.0	0.3	1.3	0.4	0.0	0.5	1.4	0.2	0.6	0.0	9.8	0.6	0.5	0.6	0.3	1.1	0.3
EnhWk3	0.83	2.7	1.0	3.8	0.4	2.3	0.8	0.1	1.1	2.1	3.6	1.0	0.0	7.8	2.6	0.5	0.7	0.7	0.7	0.4
EnhWk4	2.22	3.5	1.2	2.5	0.6	4.6	0.6	0.1	1.7	1.3	4.8	0.7	0.0	3.7	2.6	0.6	0.4	0.9	0.3	0.4
EnhWk5	0.99	1.2	1.3	1.0	0.8	3.4	1.0	0.2	1.8	1.1	1.3	1.5	0.1	0.9	1.4	0.5	1.4	1.4	0.8	0.3
EnhWk6	0.59	0.9	1.2	0.0	0.7	0.0	0.9	0.1	0.8	0.9	0.5	1.5	0.0	0.0	1.2	1.1	2.1	1.4	1.6	0.6
EnhWk7	0.48	1.4	1.3	0.0	0.8	0.0	1.1	0.1	1.4	1.7	0.0	1.5	0.0	0.0	1.3	0.9	2.3	1.8	1.2	0.9
EnhWk8	1.37	3.0	1.1	2.3	0.7	2.1	1.8	0.0	1.2	0.6	3.7	0.5	0.0	0.8	2.0	0.5	0.7	0.7	0.2	1.0
EnhA1	0.18	0.5	0.4	0.0	0.2	0.0	0.3	0.0	0.5	0.3	0.0	0.5	0.0	4.6	0.9	0.7	0.0	0.2	0.0	0.5
EnhA2	0.33	2.5	0.7	17.2	0.3	7.6	0.5	0.0	0.5	0.2	4.4	0.5	0.0	5.5	3.2	0.7	0.5	0.6	0.1	0.1
EnhA3	0.19	2.3	0.9	0.0	0.3	1.0	0.4	0.0	0.9	0.9	2.8	0.5	0.0	7.7	2.2	0.2	1.3	1.2	0.2	0.3
EnhA4	0.3	2.9	1.2	0.0	0.5	1.6	0.8	0.0	2.8	1.0	2.9	0.5	0.0	0.0	2.8	0.3	0.7	1.1	0.2	0.7
EnhA5	0.71	3.0	1.4	0.0	0.6	3.8	0.8	0.1	1.5	1.6	3.9	0.6	0.0	2.6	2.2	0.5	0.9	1.4	0.3	0.9
EnhA6	0.56	1.8	1.1	0.0	0.5	4.6	0.7	0.0	1.3	0.6	3.3	0.8	0.0	5.9	1.7	1.6	0.5	0.6	0.5	0.5
EnhA7	0.39	0.2	0.8	0.0	0.5	0.0	1.2	0.1	0.2	3.0	0.0	0.8	0.1	6.5	0.8	0.9	0.0	0.8	3.2	1.0
EnhA8	0.25	0.8	0.8	0.0	0.4	2.2	0.7	0.0	0.4	0.4	0.1	1.0	0.0	0.0	1.4	0.1	2.9	0.3	1.3	0.6
EnhA9	0.16	0.2	0.7	0.0	0.3	0.0	0.4	0.0	0.6	1.1	0.2	0.8	0.0	0.0	0.6	0.6	1.2	0.5	2.1	0.4
EnhA10	0.39	1.8	1.1	0.0	0.5	4.2	0.9	0.2	0.9	0.5	2.7	1.2	0.1	0.0	1.7	1.3	3.9	1.6	0.7	0.7
EnhA11	0.72	1.5	1.2	0.0	0.7	0.0	1.1	0.2	1.2	1.2	1.2	1.9	0.1	1.2	1.5	1.4	1.7	1.5	1.1	0.7
EnhA12	0.33	0.9	0.8	0.0	0.5	3.4	1.4	0.0	0.7	1.9	2.0	0.7	0.1	2.3	1.4	1.7	0.3	0.4	1.4	0.2
EnhA13	0.76	2.3	1.4	0.0	0.7	1.5	1.3	0.1	1.0	0.8	1.1	1.0	0.1	1.1	2.0	1.5	1.7	1.5	0.9	0.7
EnhA14	0.37	1.4	0.9	0.0	0.4	0.0	1.2	0.1	2.1	2.0	0.0	1.1	0.1	0.3	1.4	1.1	1.2	0.9	2.1	1.0
EnhA15	1.02	1.6	1.2	2.4	0.6	1.7	1.3	0.2	2.4	0.2	1.9	1.5	0.1	0.5	1.6	1.0	2.4	1.1	0.8	0.7
EnhA16	0.65	1.5	1.2	0.3	0.7	1.9	1.6	0.9	1.9	0.7	1.0	1.1	0.3	1.0	1.6	0.9	1.9	1.7	1.1	3.6
EnhA17	0.53	3.1	0.9	2.6	0.4	4.5	1.0	0.0	1.2	1.4	3.4	0.4	0.0	2.9	2.2	0.6	1.0	0.6	0.3	1.1
EnhA18	0.46	0.7	0.8	0.0	0.6	3.3	2.9	0.0	0.9	1.8	1.9	0.6	0.1	1.1	1.3	0.7	0.4	0.4	0.8	2.2
EnhA19	0.26	0.6	0.6	0.0	0.4	0.0	1.2	0.0	0.7	0.8	0.9	0.5	0.1	2.1	1.2	0.7	1.4	0.2	1.8	0.6
EnhA20	0.35	2.5	1.1	0.0	0.8	4.4	0.7	0.1	1.0	0.9	2.7	0.7	0.0	0.5	1.7	0.4	0.8	1.7	0.5	0.8
TxEnh1	0.39	1.9	1.5	0.0	0.6	2.5	0.3	0.0	1.3	0.3	2.0	0.7	0.0	0.0	1.5	0.4	1.6	1.6	0.2	0.3
TxEnh2	0.39	0.6	1.5	2.0	0.7	0.7	0.4	0.0	0.8	0.4	0.6	1.3	0.0	0.1	0.9	3.6	3.9	1.4	1.2	0.6
TxEnh3	0.25	0.1	1.2	0.0	0.6	0.0	0.9	0.2	0.4	7.4	0.1	1.7	0.1	8.7	0.5	3.4	1.2	0.8	9.1	0.6
TxEnh4	0.27	0.2	0.5	0.0	0.3	0.0	0.3	0.0	0.3	2.0	0.0	0.5	0.0	0.0	0.7	0.9	0.0	0.6	2.1	0.0
TxEnh5	0.5	0.5	0.6	0.0	0.4	0.2	0.6	0.1	0.3	2.1	0.0	0.7	0.1	2.2	0.3	1.2	0.9	0.5	2.7	0.5
TxEnh6	0.19	0.0	0.8	0.0	0.4	0.0	0.5	0.0	0.4	1.3	0.0	1.0	0.1	0.0	0.4	0.3	1.8	1.2	1.7	0.4
TxEnh7	0.27	0.5	0.7	0.0	0.4	9.4	0.5	0.0	0.8	0.7	0.5	0.8	0.0	0.0	0.9	2.8	0.3	0.4	1.9	0.4
TxEnh8	0.24	0.4	1.0	0.0	0.4	0.0	0.5	0.0	0.7	3.7	1.1	0.7	0.0	0.0	1.8	0.6	2.1	0.8	1.1	0.9
TxWk1	2.8	0.7	1.2	0.0	0.8	0.8	0.4	1.1	0.8	0.5	0.8	2.1	0.0	0.0	0.8	0.8	2.1	1.7	0.8	0.6
TxWk2	0.84	0.3	0.9	0.0	0.5	0.4	0.9	1.0	0.7	2.0	0.4	1.2	0.1	0.0	0.8	1.1	0.5	0.5	2.5	0.4
Tx1	0.82	0.7	1.7	1.4	1.1	2.5	0.4	0.1	1.5	0.9	0.8	1.7	0.0	0.4	0.7	1.4	2.9	3.0	1.1	0.5
Tx2	1.58	0.8	1.4	1.4	1.0	0.9	0.4	0.5	1.3	0.5	0.4	1.9	0.0	0.1	0.8	0.7	2.5	1.8	0.8	0.5
Tx3	0.51	0.3	1.2	0.0	0.6	0.2	0.9	0.2	1.7	3.2	0.2	1.9	0.0	2.1	0.9	1.0	1.0	0.6	5.0	0.5
Tx4	0.47	0.2	1.7	0.0	0.9	0.0	0.4	0.1	0.7	1.1	0.0	1.5	0.0	0.5	0.8	2.1	4.2	3.2	2.1	0.9
Tx5	0.94	0.2	0.9	0.0	0.4	0.0	0.5	0.8	0.5	1.6	0.0	3.7	0.1	0.0	0.6	1.2	1.7	1.4	2.9	1.0
Tx6	1.11	0.8	1.5	0.0	0.9	0.0	0.2	0.1	1.8	0.4	0.9	0.5	0.0	0.0	1.0	0.6	3.1	2.4	0.3	1.3
Tx7	0.82	0.2	1.7	0.0	0.7	0.0	0.3	0.0	1.2	0.7	1.6	0.6	0.1	0.0	1.1	1.2	3.4	2.8	0.9	0.8
Tx8	0.68	1.3	1.4	1.2	0.6	0.9	0.5	0.0	1.6	0.6	3.0	1.0	0.0	2.7	1.1	1.3	2.1	1.7	0.9	0.7
TxEx1	0.27	0.4	1.3	0.0	0.5	0.0	0.4	0.0	1.0	0.2	1.2	1.1	0.0	0.0	0.8	2.2	2.3	1.9	6.5	1.6
TxEx2	0.56	0.0	0.8	0.0	0.3	0.0	0.4	0.0	0.4	1.7	0.0	0.9	0.1	0.0	0.5	1.3	1.6	1.1	5.0	1.4
TxEx3	0.66	0.1	0.7	0.0	0.3	0.8	0.5	0.3	0.7	2.0	0.0	2.0	0.1	0.0	0.6	1.3	1.6	0.8	4.2	1.3
TxEx4	0.1	0.5	0.5	0.0	0.3	0.0	0.2	0.0	0.0	0.7	1.2	0.7	0.0	0.0	0.8	3.4	0.1	2.1	3.1	0.0
znf1	0.41	0.1	1.5	0.0	2.0	0.0	2.2	3.6	0.2	1.7	0.0	1.0	0.9	1.1	0.4	2.1	1.3	1.3	0.6	1.4
znf2	0.15	0.0	1.1	0.0	1.1	0.0	2.2	1.2	0.0	0.9	0.0	1.0	0.2	0.0	0.1	4.8	1.6	0.7	0.7	1.8
DNase1	0.2	0.6	0.9	0.0	0.3	0.0	0.7	0.1	0.3	1.7	2.6	0.5	1.0	0.0	1.7	8.5	1.6	2.9	0.2	13.5
BivProm1	0.15	0.3	0.1	0.0	0.1	5.2	0.1	0.0	0.0	0.0	0.0	0.2	0.5	0.0	0.2	1.3	0.0	0.5	0.8	11.6
BivProm2	0.16	0.0	0.2	0.0	0.1	0.0	0.1	0.0	0.0	0.0	1.5	0.2	0.4	0.0	0.1	7.4	0.2	0.9	0.1	24.9
BivProm3	0.29	1.0	0.4	0.0	0.3	0.0	0.4	0.0	0.5	1.3	0.8	0.5	0.3	4.5	0.5	1.5	0.6	0.3	0.0	7.7
BivProm4	0.13	0.5	0.4	0.0	0.2	0.0	0.2	0.1	0.0	0.0	1.5	0.4	0.2	0.0	0.7	0.5	1.8	0.7	1.3	3.1
PromF1	0.2	0.5	0.4	0.0	0.3	2.8	0.6	0.0	0.0	1.8	0.0	0.6	0.1	3.6	0.5	0.7	0.4	1.2	0.1	1.0
PromF2	0.14	1.0	0.4	0.0	0.2	0.0	0.3	0.0	0.7	1.2	0.3	0.7	0.1	0.0	0.6	0.1	1.8	3.0	0.6	1.6
PromF3	0.15	0.0	0.3	0.0	0.2	0.0	0.2	0.0	0.6	1.0	1.6	0.6	0.2	0.0	0.1	0.7	2.5	1.6	0.4	4.5
PromF4	0.19	0.1	0.1	0.0	0.0	0.0	0.0	0.0	0.0	0.0	0.0	0.2	0.1	0.0	0.1	0.6	0.0	0.8	0.9	4.1
PromF5	0.14	0.1	0.1	0.0	0.1	0.0	0.1	0.0	0.0	0.0	0.0	0.2	0.1	0.0	0.0	0.5	0.3	1.2	0.8	2.6
PromF6	0.13	0.0	0.6	0.0	0.3	0.0	0.2	0.0	0.1	0.2	2.9	0.9	0.1	0.0	0.4	0.2	2.5	1.0	0.2	0.9
PromF7	0.16	0.6	1.0	0.0	0.4	0.0	0.3	0.1	0.2	0.9	0.0	1.3	0.0	0.0	0.6	0.6	2.9	0.9	0.3	0.7
TSS1	0.12	0.3	0.1	0.0	0.0	0.0	0.1	0.0	0.3	0.0	0.6	0.3	0.2	0.0	0.1	0.0	2.1	4.3	1.0	49.7
TSS2	0.11	0.0	0.2	0.0	0.1	0.0	0.3	0.2	0.3	1.1	0.0	0.5	1.0	0.0	0.3	0.4	2.0	3.6	1.9	60.7
	100	0.01	3.17	0.0003	20.37	0.001	8.52	0.13	0.01	0.004	0.001	12.65	0.40	0.001	0.04	0.01	0.004	0.01	0.01	0.003

B

Low-complexity repeat enriched in states with high GC content

state	Genome %	GC_content_1per5bp	GC_content_2per5bp	GC_content_3per5bp	GC_content_4per5bp	GC_content_5per5bp	C-rich	G-rich	GC_rich	Low_complexity repeats
GapArtf1	11.9	0.43	0.4	0.36	0.32	0.32	0.37	0.45	0.13	0.4
GapArtf2	0.05	1.07	1.57	0.88	0.56	0.51	0.98	0.27	1.25	0.5
GapArtf3	0.01	0.83	1.43	1.09	1.03	1.58	7.47	4.68	5.35	3.7
Quies1	9.88	1.44	1.09	0.75	0.51	0.36	0.28	0.28	0.01	1.9
Quies2	3.07	1.02	1.18	1.17	1.01	0.72	0.55	0.59	0.01	0.5
Quies3	12.2	1.25	1.03	0.9	0.85	0.81	0.99	0.87	0.05	1.9
Quies4	4.45	1.31	1.13	0.87	0.67	0.55	0.35	0.5	0.04	0.8
Quies5	1.69	1.26	1.11	0.92	0.75	0.59	0.58	0.63	0.08	1.0
HET1	0.71	0.62	1.1	1.55	1.67	1.32	0.49	0.64	0.06	0.2
HET2	0.69	0.49	0.96	1.63	2.24	2.46	2.13	2.51	0.27	0.6
HET3	1.36	1.35	1.22	0.87	0.52	0.25	0.18	0.17	0	0.3
HET4	0.56	1.29	1.27	0.91	0.57	0.29	0.05	0.05	0	0.3
HET5	0.25	0.71	1.1	1.47	1.58	1.3	1.4	0.15	0.02	0.2
HET6	0.58	0.56	0.99	1.58	2.03	2.06	2.49	1.36	0.14	0.4
HET7	1.02	0.68	1.02	1.45	1.78	1.88	1.69	1.76	0.14	0.6
HET8	0.43	0.74	1.13	1.43	1.45	1.14	0.79	0.66	0.16	0.4
HET9	1	1.18	1.17	1.02	0.82	0.55	0.26	0.29	0.02	0.4
ReprPC1	0.19	0.6	0.95	1.48	2.18	3.2	3.3	3.83	2.18	1.3
ReprPC2	0.32	0.94	1.11	1.23	1.26	1.2	1.81	2.84	0.9	1.4
ReprPC3	1.11	1.03	1.19	1.16	0.98	0.65	0.68	0.74	0.02	0.7
ReprPC4	3.93	1.18	1.13	1.01	0.87	0.64	0.74	0.76	0.03	0.9
ReprPC5	0.63	0.45	0.94	1.67	2.32	2.45	2.33	2.07	0.28	0.7
ReprPC6	1.51	0.61	1.02	1.53	1.92	1.93	1.99	1.68	0.16	0.6
ReprPC7	0.61	0.32	0.81	1.73	2.91	3.66	2.57	1.96	0.31	0.5
ReprPC8	0.48	0.85	1.15	1.33	1.28	0.97	2.49	2.05	0.02	1.2
ReprPC9	0.37	1.06	1.11	1.12	1.06	0.95	1.52	1.74	0.57	1.3
Acet1	0.18	0.4	1.03	1.77	2.01	1.87	3.71	4.24	0.14	0.8
Acet2	0.85	0.65	1.13	1.53	1.57	1.12	1.4	1.3	0.01	0.4
Acet3	2.65	0.86	1.1	1.3	1.35	1.08	1.31	1.14	0.05	0.6
Acet4	0.4	0.59	1.06	1.56	1.83	1.53	1.51	1.52	0.02	0.5
Acet5	0.86	0.98	1.15	1.2	1.1	0.76	1.12	1.12	0	0.9
Acet6	0.43	1.01	1.17	1.18	1.04	0.72	0.62	0.54	0	0.7
Acet7	0.28	0.61	1.04	1.53	1.88	1.76	1.25	1.34	0.04	0.4
Acet8	0.56	0.65	1.03	1.5	1.84	1.74	1.32	1.14	0.08	0.4
EnhWk1	1.54	1.31	1.13	0.88	0.65	0.44	0.41	0.47	0.01	1.1
EnhWk2	0.35	0.43	0.89	1.65	2.55	3.02	1.39	1.58	0.22	0.4
EnhWk3	0.83	0.88	1.1	1.29	1.35	1.2	1.1	1.11	0.23	0.6
EnhWk4	2.22	1.24	1.14	0.95	0.73	0.53	0.69	0.62	0.05	0.9
EnhWk5	0.99	1.13	1.09	1.04	0.98	0.84	1.36	1.26	0.05	1.3
EnhWk6	0.59	1.06	1.1	1.11	1.08	0.91	1.09	0.72	0.04	0.9
EnhWk7	0.48	1.08	1.1	1.09	1.05	0.87	1.05	1.33	0.16	1.0
EnhWk8	1.37	1.25	1.14	0.95	0.75	0.58	0.8	0.83	0.01	0.9
EnhA1	0.18	0.51	0.94	1.59	2.32	2.69	1.61	1.51	0.1	0.5
EnhA2	0.33	0.91	1.11	1.26	1.31	1.22	1.04	1.04	0.15	0.6
EnhA3	0.19	1.06	1.17	1.12	0.98	0.81	0.31	0.9	0.03	0.6
EnhA4	0.3	1.14	1.19	1.05	0.83	0.57	0.6	0.31	0	0.6
EnhA5	0.71	1.25	1.16	0.94	0.69	0.46	0.32	0.37	0.06	0.8
EnhA6	0.56	0.94	1.11	1.24	1.27	1.14	1.14	0.99	0.09	0.6
EnhA7	0.39	0.54	0.99	1.58	2.1	2.22	2.05	2.67	0.21	0.7
EnhA8	0.25	0.77	1.04	1.37	1.66	1.81	1.96	1.15	0.31	0.7
EnhA9	0.16	0.63	1	1.48	1.99	2.19	1.34	1.4	0.08	0.4
EnhA10	0.39	1.01	1.11	1.15	1.14	1.1	1.4	1.31	0.57	0.9
EnhA11	0.72	1.03	1.08	1.14	1.15	1	0.71	1.25	0.04	1.0
EnhA12	0.33	0.67	1.06	1.48	1.76	1.66	0.84	1.02	0.06	0.4
EnhA13	0.76	1.13	1.14	1.06	0.9	0.66	0.52	0.38	0.03	0.8
EnhA14	0.37	0.75	1.05	1.4	1.67	1.66	1.35	1.4	0.18	0.5
EnhA15	1.02	1.09	1.1	1.09	1.03	0.84	0.83	0.78	0.05	0.8
EnhA16	0.65	1.13	1.1	1.05	0.99	1.08	4.14	4.43	1.32	1.5
EnhA17	0.53	1.11	1.16	1.08	0.93	0.76	1.16	1.07	0.33	0.7
EnhA18	0.46	0.84	1.11	1.33	1.41	1.27	1.63	1.44	0.3	0.6
EnhA19	0.26	0.66	1.03	1.49	1.86	1.89	2.67	2.24	0.18	0.7
EnhA20	0.35	1.32	1.13	0.87	0.64	0.44	0.36	0.5	0	1.2
TxEnh1	0.39	1.26	1.15	0.94	0.7	0.49	0.23	0.63	0.05	0.7
TxEnh2	0.39	1.11	1.14	1.07	0.96	0.77	0.33	0.31	0.03	0.4
TxEnh3	0.25	0.66	1.03	1.49	1.81	1.71	0.35	0.3	0.09	0.2
TxEnh4	0.27	0.46	0.94	1.65	2.34	2.7	0.8	1	0.21	0.3
TxEnh5	0.5	0.46	0.91	1.63	2.42	2.98	1.4	1.36	0.55	0.4
TxEnh6	0.19	0.69	1.03	1.45	1.81	1.94	1.14	1.2	0.29	0.4
TxEnh7	0.27	0.55	0.99	1.58	2.11	2.22	0.59	1.06	0.1	0.2
TxEnh8	0.24	0.82	1.11	1.34	1.43	1.29	0.45	0.32	0.04	0.3
TxWk1	2.8	1.16	1.04	1	1	0.92	0.55	0.57	0.11	1.3
TxWk2	0.84	0.58	1	1.54	2.03	2.26	1.97	1.57	0.51	0.5
Tx1	0.82	1.22	1.09	0.96	0.86	0.72	0.27	0.4	0.05	0.7
Tx2	1.58	1.18	1.05	0.98	0.94	0.83	0.64	0.51	0.03	1.2
Tx3	0.51	0.68	1.05	1.47	1.71	1.51	1.25	1.28	0.07	0.4
Tx4	0.47	1.15	1.13	1.04	0.92	0.7	0.28	0.26	0.04	0.4
Tx5	0.94	0.73	0.96	1.4	1.8	1.79	0.84	0.77	0.27	0.6
Tx6	1.11	1.45	1.13	0.75	0.46	0.28	0.07	0.08	0.01	1.0
Tx7	0.82	1.28	1.19	0.93	0.64	0.4	0.13	0.17	0	0.4
Tx8	0.68	1.11	1.15	1.07	0.92	0.71	0.33	0.54	0.06	0.5
TxEx1	0.27	0.89	1.14	1.28	1.28	1.09	0.42	0.26	0.04	0.2
TxEx2	0.56	0.68	1.06	1.47	1.74	1.72	0.29	0.37	0.05	0.2
TxEx3	0.66	0.55	0.93	1.55	2.21	2.63	0.99	1.09	0.42	0.3
TxEx4	0.1	0.61	1.01	1.52	1.97	2.11	0.47	0.34	0.27	0.2
znf1	0.41	1.05	1.13	1.13	1.07	0.87	0.44	0.47	0.09	0.3
znf2	0.15	0.88	1.13	1.31	1.28	1.04	0.23	0.36	0.17	0.1
DNase1	0.2	0.87	1.02	1.22	1.54	2.86	12	11.7	10.6	2.6
BivProm1	0.15	0.32	0.64	1.43	3.47	11.1	6.52	7.8	38.7	2.8
BivProm2	0.16	0.39	0.72	1.46	3.13	8.58	8.16	8.38	25.9	2.7
BivProm3	0.29	0.53	0.9	1.52	2.4	4.03	7.66	8.17	14.5	2.1
BivProm4	0.13	0.93	1.05	1.2	1.44	2.06	2.65	3.77	3.16	1.6
PromF1	0.2	0.46	0.88	1.6	2.54	3.43	3.11	2.91	0.89	0.7
PromF2	0.14	0.59	0.94	1.48	2.22	3.12	2.15	2.27	0.96	0.6
PromF3	0.15	0.57	0.89	1.44	2.44	4.88	2.53	1.65	4.2	0.7
PromF4	0.19	0.32	0.62	1.38	3.5	12.8	7.04	6.35	48.7	2.6
PromF5	0.14	0.37	0.67	1.4	3.33	10.5	9.72	8.16	38.4	3.0
PromF6	0.13	1	1.09	1.15	1.24	1.58	0.6	0.66	0.5	0.5
PromF7	0.16	1.15	1.09	1.01	0.97	1.05	0.52	0.26	0.05	0.7
TSS1	0.12	0.3	0.52	1.2	3.71	18.9	29.2	29.6	195	10.5
TSS2	0.11	0.35	0.53	1.13	3.49	20.3	59.6	68.6	303	18.4
	100	21.9	29.9	23	8.49	0.89	0.03	0.03	0.02	0.54

C

Simple repeats enriched in Acet1

State	Genome %	(CA)n	(TG)n	Simple_repeat
GapArtf1	11.9	0.24	0.23	0.6
GapArtf2	0.05	0.48	0.38	3.4
GapArtf3	0.01	2.54	1.79	6.5
Quies1	9.88	0.79	0.8	1.5
Quies2	3.07	1.17	1.15	0.7
Quies3	12.2	0.7	0.68	1.8
Quies4	4.45	0.53	0.56	0.9
Quies5	1.69	0.87	0.85	1.2
HET1	0.71	2.39	2.22	1.1
HET2	0.69	2.08	1.83	1.0
HET3	1.36	0.46	0.42	0.4
HET4	0.56	0.31	0.31	0.4
HET5	0.25	0.78	1.02	0.7
HET6	0.58	0.96	0.93	0.8
HET7	1.02	0.59	0.73	0.9
HET8	0.43	0.91	0.96	0.9
HET9	1	0.67	0.61	0.7
ReprPC1	0.19	2.77	2.77	1.3
ReprPC2	0.32	1.26	1.12	1.1
ReprPC3	1.11	0.72	0.71	0.7
ReprPC4	3.93	0.68	0.69	0.8
ReprPC5	0.63	2.04	2.12	1.0
ReprPC6	1.51	0.83	1.05	0.8
ReprPC7	0.61	1.41	1.59	0.7
ReprPC8	0.48	1.54	1.35	1.2
ReprPC9	0.37	0.92	0.81	1.1
Acet1	0.18	72.2	75.6	23.2
Acet2	0.85	4.26	4.44	1.7
Acet3	2.65	1.56	1.47	1.0
Acet4	0.4	2.98	3.57	1.1
Acet5	0.86	1.12	1.1	0.8
Acet6	0.43	1.24	0.9	0.6
Acet7	0.28	2.36	2.15	0.8
Acet8	0.56	1.09	0.96	0.5
EnhWk1	1.54	0.77	0.78	0.8
EnhWk2	0.35	1.64	1.65	0.7
EnhWk3	0.83	1.12	1	0.6
EnhWk4	2.22	1.07	1.06	0.8
EnhWk5	0.99	0.85	0.85	1.0
EnhWk6	0.59	0.82	0.88	0.8
EnhWk7	0.48	0.73	0.88	0.7
EnhWk8	1.37	0.63	0.63	0.6
EnhA1	0.18	2.03	2.18	0.8
EnhA2	0.33	1.1	1.2	0.6
EnhA3	0.19	0.85	0.9	0.5
EnhA4	0.3	0.69	0.82	0.4
EnhA5	0.71	0.75	0.77	0.6
EnhA6	0.56	1.13	1.1	0.7
EnhA7	0.39	2.32	2.42	1.0
EnhA8	0.25	1.69	1.72	0.8
EnhA9	0.16	1.77	1.81	0.7
EnhA10	0.39	0.82	0.93	0.7
EnhA11	0.72	0.73	0.72	0.8
EnhA12	0.33	1.34	1.24	0.6
EnhA13	0.76	0.76	0.8	0.7
EnhA14	0.37	1.08	1.07	0.5
EnhA15	1.02	0.81	0.84	0.7
EnhA16	0.65	0.85	0.7	0.9
EnhA17	0.53	0.81	0.73	0.5
EnhA18	0.46	1.15	1.24	0.5
EnhA19	0.26	2.26	2.52	0.8
EnhA20	0.35	0.78	0.76	0.7
TxEnh1	0.39	0.75	0.68	0.5
TxEnh2	0.39	0.55	0.61	0.4
TxEnh3	0.25	1.3	2.04	0.7
TxEnh4	0.27	3.12	2.59	1.0
TxEnh5	0.5	2	1.65	0.8
TxEnh6	0.19	1.26	1.15	0.6
TxEnh7	0.27	2.57	2.69	0.9
TxEnh8	0.24	0.89	0.77	0.4
TxWk1	2.8	0.59	0.56	0.8
TxWk2	0.84	1.35	1.18	0.9
Tx1	0.82	0.53	0.54	0.5
Tx2	1.58	0.72	0.76	0.9
Tx3	0.51	1.47	1.64	0.9
Tx4	0.47	0.44	0.45	0.4
Tx5	0.94	0.54	0.59	0.5
Tx6	1.11	0.47	0.41	0.5
Tx7	0.82	0.39	0.39	0.3
Tx8	0.68	0.65	0.64	0.5
TxEx1	0.27	0.49	0.5	0.3
TxEx2	0.56	0.92	0.66	0.4
TxEx3	0.66	0.89	0.83	0.5
TxEx4	0.1	1.33	1.29	0.5
znf1	0.41	0.66	0.61	0.6
znf2	0.15	1.57	1.26	0.6
DNase1	0.2	0.94	1.01	1.7
BivProm1	0.15	1.95	1.72	1.3
BivProm2	0.16	2.6	2.2	1.3
BivProm3	0.29	2.52	2.28	1.3
BivProm4	0.13	1.83	1.79	1.1
PromF1	0.2	2.9	2.83	1.0
PromF2	0.14	1.54	1.92	0.8
PromF3	0.15	0.8	1.22	0.5
PromF4	0.19	0.43	0.29	0.9
PromF5	0.14	1.79	1.73	1.1
PromF6	0.13	0.45	0.46	0.3
PromF7	0.16	0.46	0.45	0.4
TSS1	0.12	0.42	0.52	2.8
TSS2	0.11	0.67	0.52	5.6
	100	0.1	0.1	0.84

Figure S28: Full stack states enrichments with RepeatMasker classes of repeats (A), low-complexity repeats and GC content (B), simple repeats (C). This is an extended version of Fig. 4C. In each panel, the rows correspond to full-stack states. The second column reports the percentage of the genome that each full-stack state occupies. In **(A)**, columns 3-21 correspond to different repeat classes. In **(B)**, columns 3-7 correspond to 5bp windows in the genome are stratified

by the number of G/C bases in them, columns 8-10 correspond to regions enriched with C-rich, G-rich, and GC-rich low complexity sequences, respectively, and column 11 shows enrichments for all low complexity sequences from RepeatMasker. States TSS1-2 are most enriched with Low complexity repeat class, which is consistent with these states having a high enrichment (19-20 fold) for windows in which all bases are a G or C. In **(C)**, columns 3-4 correspond to simple repeats of repeated (CA) and (TG) sequences, and column 5 shows enrichments for all simple repeats. State Acet1 is most enriched with simple repeats and this enrichment is mostly driven by enrichments with repeated CA and TG dinucleotides. In each panel, the values in all columns except the first and second columns correspond to fold enrichment for different repeat contexts in the full-stack states. Values are colored on a column-specific color scale. The last row gives the percentage of the genome that each repeat class occupies.

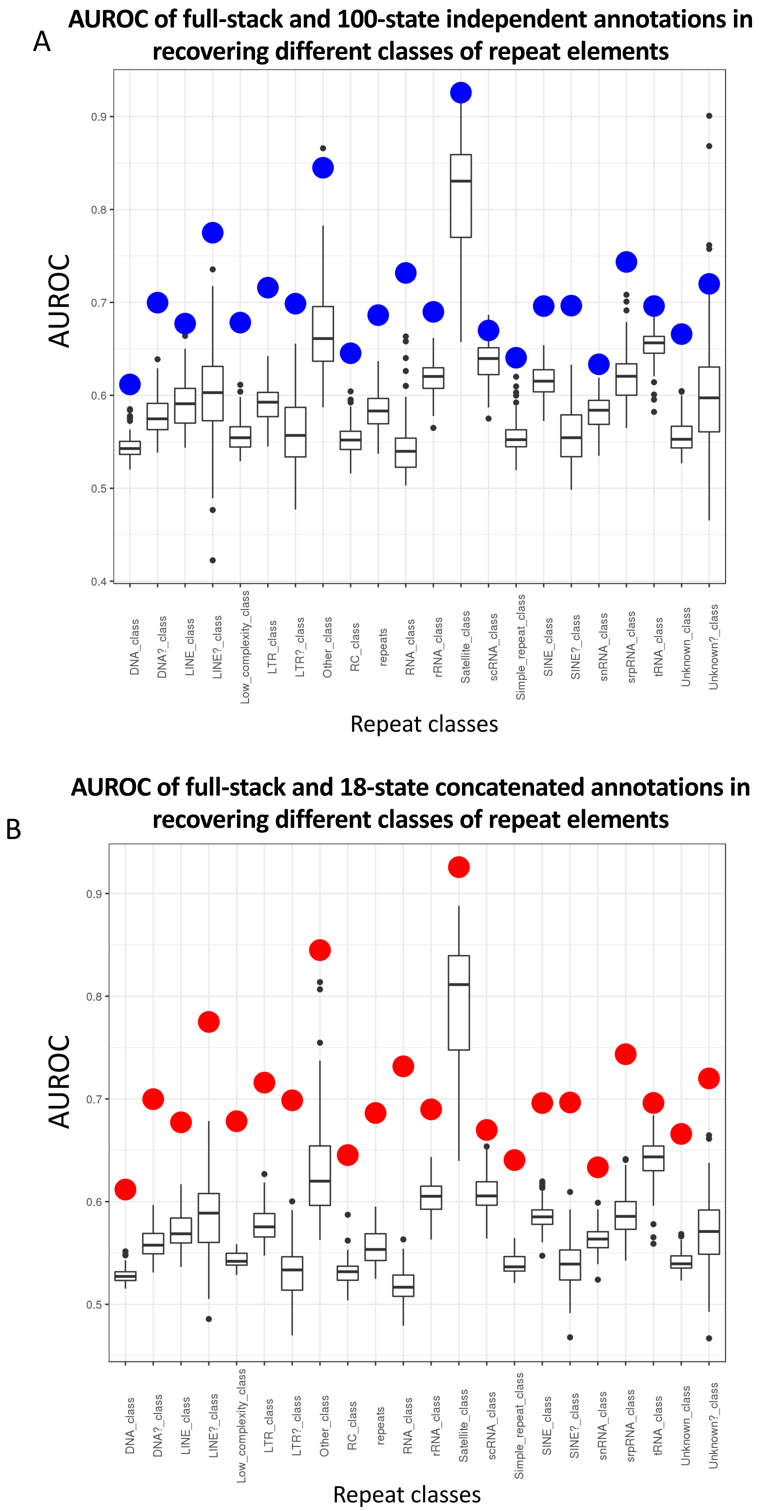

Figure S29: AUROC comparison of the full-stack, concatenated and independent chromatin state annotations at predicting different classes of repeat elements. Box-plots showing AUROC of the **(A)** 127 100-state annotations from independent models and **(B)** 98 18-state annotations from a concatenated model at predicting bases in different repeat classes labeled on the x-axis. The dots colored **(A)** blue and **(B)** red show the AUROC for the full-stack chromatin state annotations.

A

state	max_enrich	consHMM_state
GapArtf1	7.7	96-AM_SPrim
GapArtf2	8.1	96-AM_SPrim
GapArtf3	7.2	95-AM_SPrim
Quies1	1.8	30-AM_SMam
Quies2	1.8	76-AM_Prim
Quies3	1.7	80-AM_Prim
Quies4	4.6	86-AM_Prim
Quies5	1.8	72-AM_Prim
HET1	3.4	76-AM_Prim
HET2	3.7	82-AM_Prim
HET3	4.4	86-AM_Prim
HET4	2.8	93-AM_SPrim
HET5	5.7	100-artifact
HET6	5.3	93-AM_SPrim
HET7	4.3	93-AM_SPrim
HET8	2.6	82-AM_Prim
HET9	3.5	93-AM_SPrim
ReprPC1	4.6	1-AM_allVert
ReprPC2	2.5	5-AM_Mam
ReprPC3	1.6	100-artifact
ReprPC4	1.5	69-AM_Prim
ReprPC5	2.7	82-AM_Prim
ReprPC6	1.9	100-artifact
ReprPC7	4.6	28-AM_SMam
ReprPC8	2.6	100-artifact
ReprPC9	1.8	100-artifact
Acet1	9.0	82-AM_Prim
Acet2	2.5	100-artifact
Acet3	1.9	75-AM_Prim
Acet4	2.0	14-AM_Mam
Acet5	1.6	75-AM_Prim
Acet6	2.1	9-AM_Mam
Acet7	2.6	14-AM_Mam
Acet8	1.6	75-AM_Prim
EnhWk1	2.2	5-AM_Mam
EnhWk2	2.7	28-AM_SMam
EnhWk3	2.8	5-AM_Mam
EnhWk4	3.7	2-AM_nonMam
EnhWk5	1.4	75-AM_Prim
EnhWk6	1.6	13-AM_Mam
EnhWk7	1.5	77-AM_Prim
EnhWk8	2.7	2-AM_nonMam
EnhA1	4.1	5-AM_Mam
EnhA2	5.7	2-AM_nonMam
EnhA3	4.8	5-AM_Mam
EnhA4	3.7	5-AM_Mam
EnhA5	3.2	5-AM_Mam
EnhA6	3.1	2-AM_nonMam
EnhA7	2.0	28-AM_SMam
EnhA8	2.3	15-AM_Mam
EnhA9	2.8	14-AM_Mam
EnhA10	2.2	6-AM_Mam
EnhA11	1.7	77-AM_Prim
EnhA12	2.1	14-AM_Mam
EnhA13	1.6	9-AM_Mam
EnhA14	2.2	14-AM_Mam
EnhA15	1.5	6-AM_Mam
EnhA16	1.9	100-artifact
EnhA17	6.4	2-AM_nonMam
EnhA18	2.2	94-AM_SPrim
EnhA19	3.0	2-AM_nonMam
EnhA20	2.1	5-AM_Mam
TxEnh1	2.6	6-AM_Mam
TxEnh2	2.2	37-AM_SMam
TxEnh3	2.3	82-AM_Prim
TxEnh4	15.9	1-AM_allVert
TxEnh5	9.5	1-AM_allVert
TxEnh6	5.6	1-AM_allVert
TxEnh7	2.4	47-A_SMam
TxEnh8	5.6	1-AM_allVert
TxWk1	2.4	7-AM_Mam
TxWk2	6.7	1-AM_allVert
Tx1	1.9	44-AM_SMam
Tx2	2.0	77-AM_Prim
Tx3	2.1	75-AM_Prim
Tx4	2.9	1-AM_allVert
Tx5	4.0	77-AM_Prim
Tx6	5.9	7-AM_Mam
Tx7	8.4	1-AM_allVert
Tx8	2.8	3-AM_nonMam
TxEx1	12.1	1-AM_allVert
TxEx2	18.4	1-AM_allVert
TxEx3	10.1	1-AM_allVert
TxEx4	11.4	1-AM_allVert
znf1	3.0	79-AM_Prim
znf2	10.9	100-artifact
DNase1	3.1	28-AM_SMam
BivProm1	16.6	28-AM_SMam
BivProm2	11.3	28-AM_SMam
BivProm3	6.0	28-AM_SMam
BivProm4	7.8	2-AM_nonMam
PromF1	3.0	28-AM_SMam
PromF2	3.8	5-AM_Mam
PromF3	3.9	28-AM_SMam
PromF4	20.7	28-AM_SMam
PromF5	15.6	28-AM_SMam
PromF6	3.4	1-AM_allVert
PromF7	2.7	6-AM_Mam
TSS1	44.5	28-AM_SMam
TSS2	47.8	28-AM_SMam

- High align and match frequencies for a few primates
- High align and march for mammals, but missing notable subsets
- High align and match frequencies for primates
- Putative artifact
- High align and match frequency for all vertebrates
- High align and match for mammals
- High align and match for mammals and some non-mammals

B

ConsHMM_state	Characterization based on Supp. Data. File 1 from Arneson & Ernst, 2019
1-AM_allVert	**Most conserved**. High align and match frequency with all vertebrates' genomes. Most enriched for CDS, UTRs, exons of protein coding genes with preference for 1st and 2nd codon position, canonical splice-site.
2-AM_nonMam	**Conserved enhancers**. Most enriched for TES of protein coding genes, enhancer-group chromatin states in ct-spec annotations
3-AM_nonMam	Enriched for CDS and exons of protein coding genes with 3rd codon position preference; DHS in non-exons
5-AM_Mam	**Conserved enhancer and DNase** (top2 most enriched with enhancer-related and DNase states in ct-spec annotations)
14-AM_Mam	Most enriched state for TxEnh5' and TxEnhW chromatin states in ct-spec annotations
28-AM_SMam	Strongest signals of overlapping **TSS and promoter** states in ct-spec annotations, CpG islands, Low Complexity class and family Repeats, DHS in non-exons
82-AM_Prim	**Moderate conservation** (align and match with primates); **ReprPC** state in ct-spec annotations
75-AM_Prim	Most enriched state for **RNA, scRNA, snRNA and srpRNA class and family repeats**
76-AM_Prim	Most enriched state for **LTR class and ERVL-MaLR, PiggyBac, and TcMar-Mariner family repeats**
77-AM_Prim	Most enriched state for **SINE class, and Alu family repeats** and Tx5' and TxWk chromatin states
86-AM_Prim	Most enriched state for **LINE class, L1 family repeats**, and **Quies** chromatin state in ct-spec annotations
100-artifact	**Alignment artifacts**. Most enriched state for TSS, Exons, TES of pseudogenes, ZNF/Rpts chromatin state in ct-spec annotations. tRNA class and family repeats, protein-DNA complex assembly genes
93-AM_SPrim	Most enriched state for **HET** chromatin state in ct-spec annotations, **ERV1 and ERVK family repeats**
95-AM_SPrim	Most enriched state for **simple and rRNA class and family and acro and telo family repeats**
96-AM_SPrim	Most enriched state for assembly gaps, centr family repeats

C

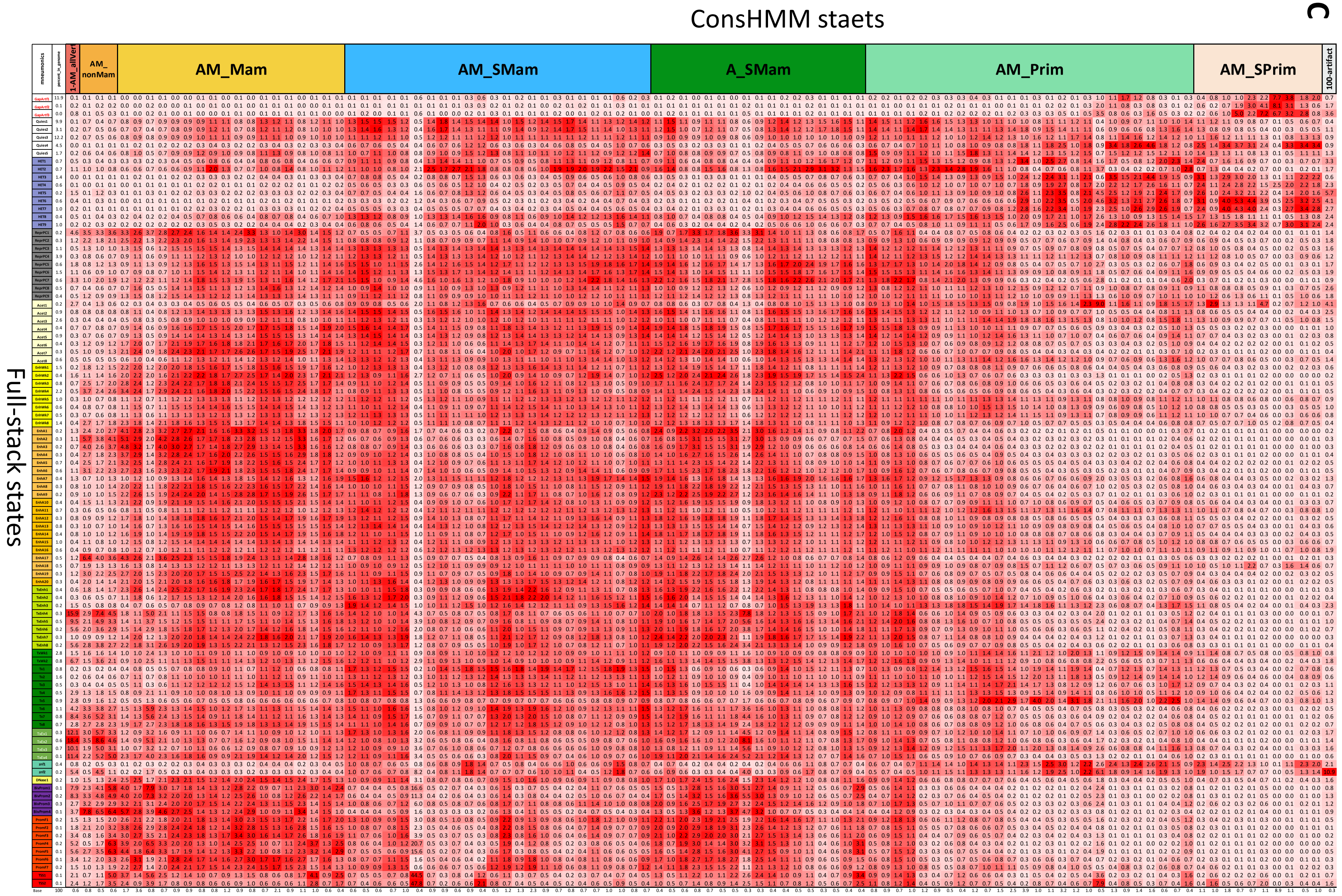
ConsHMM staets
Full-stack states
mneumonics
percent_in_genome
1-AM_allVert
AM_nonMam
AM_Mam
AM_SMam
A_SMam
AM_Prim
AM_SPrim
100-artifact

Figure S30: Full-stack states enrichments with conservation states. (A) The first column gives the label of the full-stack states. The second column shows the maximum fold enrichment for each full-stack state for any ConsHMM state defined to annotate nucleotides based on sequence conservation patterns (Arneson & Ernst, 2019) (**Methods**). The third column shows the ConsHMM state that had the highest fold-enrichment in each full-stack state. One notable ConsHMM state is state 1 (1-AM_allVert), representing regions with high probabilities of aligning and matching the human reference genome for all vertebrates and the most enriched for exons. Full-stack states in the transcription-exon group (TxEx) are all maximally enriched with ConsHMM state 1. Another notable ConsHMM state, state 28 (28-AM_SMam), was the ConsHMM most strongly enriched for overlapping annotated TSS. Consistent with this, this state is also the maximum-enriched ConsHMM state in many full-stack states in TSS and Promoter flanking groups. **(B)** Characterizations of notable ConsHMM states. **(C)** Enrichments of full-stack states for each ConsHMM state from a 100-state model based on a 100-way vertebrate alignment (Arneson & Ernst, 2019). Rows (vertical) correspond to different full-stack states. The header row gives the ConsHMM state labels, where ConsHMM states are placed in groups previously defined based on their patterns of sequence alignment with other vertebrates (Arneson & Ernst, 2019), colored as in **(A)**. The second column (horizontal) shows the percentage of the genome that each full-stack state falls into. Each of the remaining columns (horizontal) corresponds to one ConsHMM state. Values in the columns are colored on a column specific coloring scale. The last row (vertical) in the heatmap gives the percentage of the genome that is covered by each ConsHMM state. The corresponding excel file for this figure is provided in **Additional File 7.**

Enrichment of full-stack states major ZNF gene states

State	Genome %	ZNF* gene	ZNF* C2H2 gene	ZNF* not C2H2
GapArtf1	11.86	0.21	0.14	0.42
GapArtf2	0.05	1.44	1.68	0.79
GapArtf3	0.01	1.26	1.54	0.60
Quies1	9.88	0.40	0.03	1.51
Quies2	3.07	0.29	0.11	0.83
Quies3	12.23	0.55	0.41	0.99
Quies4	4.45	0.80	0.66	1.20
Quies5	1.69	0.20	0.22	0.13
HET1	0.71	0.33	0.11	1.00
HET2	0.69	0.66	0.80	0.17
HET3	1.36	1.77	1.83	1.58
HET4	0.56	6.03	7.02	3.68
HET5	0.25	13.97	16.70	7.34
HET6	0.58	2.58	2.93	1.43
HET7	1.02	2.04	2.15	1.70
HET8	0.43	0.87	0.81	0.98
HET9	1.00	1.44	1.56	1.00
ReprPC1	0.19	0.30	0.12	0.86
ReprPC2	0.32	0.28	0.17	0.59
ReprPC3	1.11	0.36	0.28	0.59
ReprPC4	3.93	0.29	0.23	0.46
ReprPC5	0.63	0.38	0.27	0.70
ReprPC6	1.51	0.32	0.25	0.52
ReprPC7	0.61	0.61	0.61	0.58
ReprPC8	0.48	0.37	0.31	0.56
ReprPC9	0.37	0.53	0.47	0.70
Acet1	0.18	1.09	1.00	1.42
Acet2	0.85	0.37	0.24	0.73
Acet3	2.65	0.40	0.28	0.74
Acet4	0.40	0.38	0.41	0.25
Acet5	0.86	0.32	0.30	0.36
Acet6	0.43	0.46	0.48	0.38
Acet7	0.28	0.39	0.45	0.19
Acet8	0.56	0.31	0.26	0.45
EnhWk1	1.54	0.59	0.35	1.30
EnhWk2	0.35	0.66	0.64	0.75
EnhWk3	0.83	0.72	0.62	1.02
EnhWk4	2.22	0.59	0.42	1.08
EnhWk5	0.99	0.60	0.49	0.91
EnhWk6	0.59	0.72	0.62	0.99
EnhWk7	0.48	0.50	0.58	0.21
EnhWk8	1.37	0.42	0.28	0.84
EnhA1	0.18	0.73	0.88	0.28
EnhA2	0.33	0.80	0.85	0.61
EnhA3	0.19	0.82	1.00	0.20
EnhA4	0.30	0.54	0.58	0.37
EnhA5	0.71	0.55	0.47	0.77
EnhA6	0.56	0.91	0.98	0.65
EnhA7	0.39	0.50	0.39	0.80
EnhA8	0.25	0.71	0.56	1.09
EnhA9	0.16	0.89	1.14	0.07
EnhA10	0.39	0.92	1.03	0.61
EnhA11	0.72	0.73	0.78	0.55
EnhA12	0.33	0.31	0.24	0.50
EnhA13	0.76	0.27	0.19	0.52
EnhA14	0.37	0.34	0.29	0.52
EnhA15	1.02	0.42	0.32	0.71
EnhA16	0.65	0.41	0.35	0.57
EnhA17	0.53	0.94	0.99	0.73
EnhA18	0.46	0.49	0.49	0.47
EnhA19	0.26	1.12	1.25	0.64
EnhA20	0.35	0.77	0.77	0.69
TxEnh1	0.39	1.14	1.40	0.27
TxEnh2	0.39	1.95	2.14	1.24
TxEnh3	0.25	3.15	3.76	1.05
TxEnh4	0.27	1.47	1.76	0.58
TxEnh5	0.50	1.42	1.69	0.61
TxEnh6	0.19	1.14	1.44	0.11
TxEnh7	0.27	1.28	1.59	0.24
TxEnh8	0.24	1.22	1.41	0.57
TxWk1	2.80	1.71	1.84	1.37
TxWk2	0.84	1.27	1.28	1.21
Tx1	0.82	2.24	2.25	2.11
Tx2	1.58	1.74	1.88	1.28
Tx3	0.51	1.64	2.03	0.32
Tx4	0.47	2.70	3.01	1.55
Tx5	0.94	2.17	2.51	1.03
Tx6	1.11	1.71	1.79	1.58
Tx7	0.82	1.91	2.13	1.23
Tx8	0.68	1.29	1.40	0.92
TxEx1	0.27	2.61	2.97	1.27
TxEx2	0.56	2.45	2.85	1.10
TxEx3	0.66	2.60	3.14	0.94
TxEx4	0.10	2.17	2.55	0.91
znf1	0.41	20.83	25.12	8.91
znf2	0.15	68.59	86.77	17.81
DNase1	0.20	0.80	0.77	0.86
BivProm1	0.15	0.87	0.66	1.44
BivProm2	0.16	0.50	0.34	0.95
BivProm3	0.29	0.82	0.80	0.83
BivProm4	0.13	1.28	1.13	1.78
PromF1	0.20	0.81	0.76	0.91
PromF2	0.14	1.23	1.34	0.85
PromF3	0.15	3.39	3.95	1.60
PromF4	0.19	4.78	5.61	2.20
PromF5	0.14	2.27	2.34	2.19
PromF6	0.13	4.34	5.14	2.05
PromF7	0.16	4.41	5.24	1.76
TSS1	0.12	3.75	4.31	1.98
TSS2	0.11	2.13	2.27	1.76
Base %	100	0.73	0.57	0.18

Figure S31: Full-stack states enrichments with different subsets of ZNF genes. The rows correspond to full-stack states. The first column presents the state labels, the second presents the percentage of the genome that each state occupies, and the remaining three columns enrichments for different subsets of zinc finger genes. The first of these is all genes with a ZNF symbol. The second is the subset of ZNF genes also annotated as C2H2 genes and the third those that are not C2H2 genes. The values correspond to the full-stack states' fold enrichment for the ZNF gene families. Values are colored on a column-specific color scale. The last row gives the percentage of the genome that each type of ZNF gene family occupies

Enrichment of full-stack states with structural variants

state	% Genome	deletion	duplication
GapArtf1	4.88	0.71	0.76
GapArtf2	0.05	1.29	1.58
GapArtf3	0.01	0.89	1.22
Quies1	10.7	1.63	1.21
Quies2	3.31	1.57	1.26
Quies3	13.2	1.04	0.94
Quies4	4.8	1.23	1.14
Quies5	1.82	0.32	1.19
HET1	0.76	1.58	1.3
HET2	0.75	1	1.45
HET3	1.47	1.16	1.03
HET4	0.61	1.54	1.47
HET5	0.27	1.23	1.22
HET6	0.63	1.36	1.37
HET7	1.1	1.18	1.13
HET8	0.47	0.96	1.08
HET9	1.07	1.35	1.46
ReprPC1	0.21	0.5	0.81
ReprPC2	0.35	0.63	0.86
ReprPC3	1.19	0.77	0.88
ReprPC4	4.24	0.86	0.92
ReprPC5	0.68	0.81	1.08
ReprPC6	1.63	0.81	0.97
ReprPC7	0.66	0.73	1.03
ReprPC8	0.51	0.16	1.22
ReprPC9	0.4	0.86	1.01
Acet1	0.2	1	1.19
Acet2	0.92	1	0.94
Acet3	2.86	1.06	0.96
Acet4	0.43	0.87	0.89
Acet5	0.93	0.99	0.92
Acet6	0.46	0.94	0.88
Acet7	0.31	0.82	0.88
Acet8	0.61	0.9	0.98
EnhWk1	1.66	1.04	0.93
EnhWk2	0.38	0.73	0.91
EnhWk3	0.89	0.85	0.88
EnhWk4	2.39	1.04	0.91
EnhWk5	1.07	1.01	0.94
EnhWk6	0.72	0.7	0.72
EnhWk7	0.52	0.95	0.96
EnhWk8	1.48	1.17	1.02
EnhA1	0.19	0.66	0.86
EnhA2	0.35	0.82	0.86
EnhA3	0.21	0.75	0.84
EnhA4	0.32	0.87	0.87
EnhA5	0.77	0.96	0.9
EnhA6	0.61	0.79	0.84
EnhA7	0.42	0.81	0.99
EnhA8	0.27	0.73	0.81
EnhA9	0.17	0.67	0.75
EnhA10	0.43	0.82	0.91
EnhA11	0.77	0.93	1.01
EnhA12	0.36	0.82	0.93
EnhA13	0.82	0.97	0.95
EnhA14	0.39	0.82	0.99
EnhA15	1.1	0.93	0.98
EnhA16	0.69	1.03	1
EnhA17	0.57	0.9	0.88
EnhA18	0.5	1.04	1.06
EnhA19	0.28	0.8	0.9
EnhA20	0.37	1.04	0.92
TxEnh1	0.42	0.7	0.85
TxEnh2	0.42	0.7	0.83
TxEnh3	0.27	0.75	0.91
TxEnh4	0.29	0.51	0.86
TxEnh5	0.54	0.62	0.94
TxEnh6	0.2	0.59	0.8
TxEnh7	0.29	0.66	0.86
TxEnh8	0.26	0.57	0.78
TxWk1	3.02	0.74	0.88
TxWk2	0.91	0.7	0.96
Tx1	0.89	0.77	0.87
Tx2	1.7	0.85	0.92
Tx3	0.55	0.82	0.96
Tx4	0.51	0.63	0.87
Tx5	1.02	0.66	0.94
Tx6	1.2	0.56	0.85
Tx7	0.88	0.52	0.84
Tx8	0.73	0.63	0.8
TxEx1	0.29	0.53	0.82
TxEx2	0.6	0.49	0.93
TxEx3	0.72	0.57	0.97
TxEx4	0.11	0.45	0.8
znf1	0.44	0.94	1.02
znf2	0.16	1.09	1.26
DNase1	0.22	0.81	0.98
BivProm1	0.16	0.53	0.9
BivProm2	0.17	0.52	0.88
BivProm3	0.31	0.66	1
BivProm4	0.14	0.54	0.84
PromF1	0.22	0.75	0.99
PromF2	0.15	0.6	0.86
PromF3	0.16	0.56	0.87
PromF4	0.21	0.51	0.88
PromF5	0.15	0.6	0.9
PromF6	0.14	0.52	0.88
PromF7	0.17	0.58	0.87
TSS1	0.13	0.53	0.9
TSS2	0.12	0.62	0.99
	100	29.1	31.6

Figure S32: Full-stack states enrichments with structural variants. The rows correspond to full-stack states. The first column presents the state labels, the second presents the percentage of the genome in hg38 that each state occupies, and the last two columns correspond to two different types of structural variants: deletions and duplications. The values correspond to the full-stack states' fold enrichment for the structural variant type. Values are colored on a column-specific color scale. The last row gives the percentage of the genome that each type of structural variants occupies.

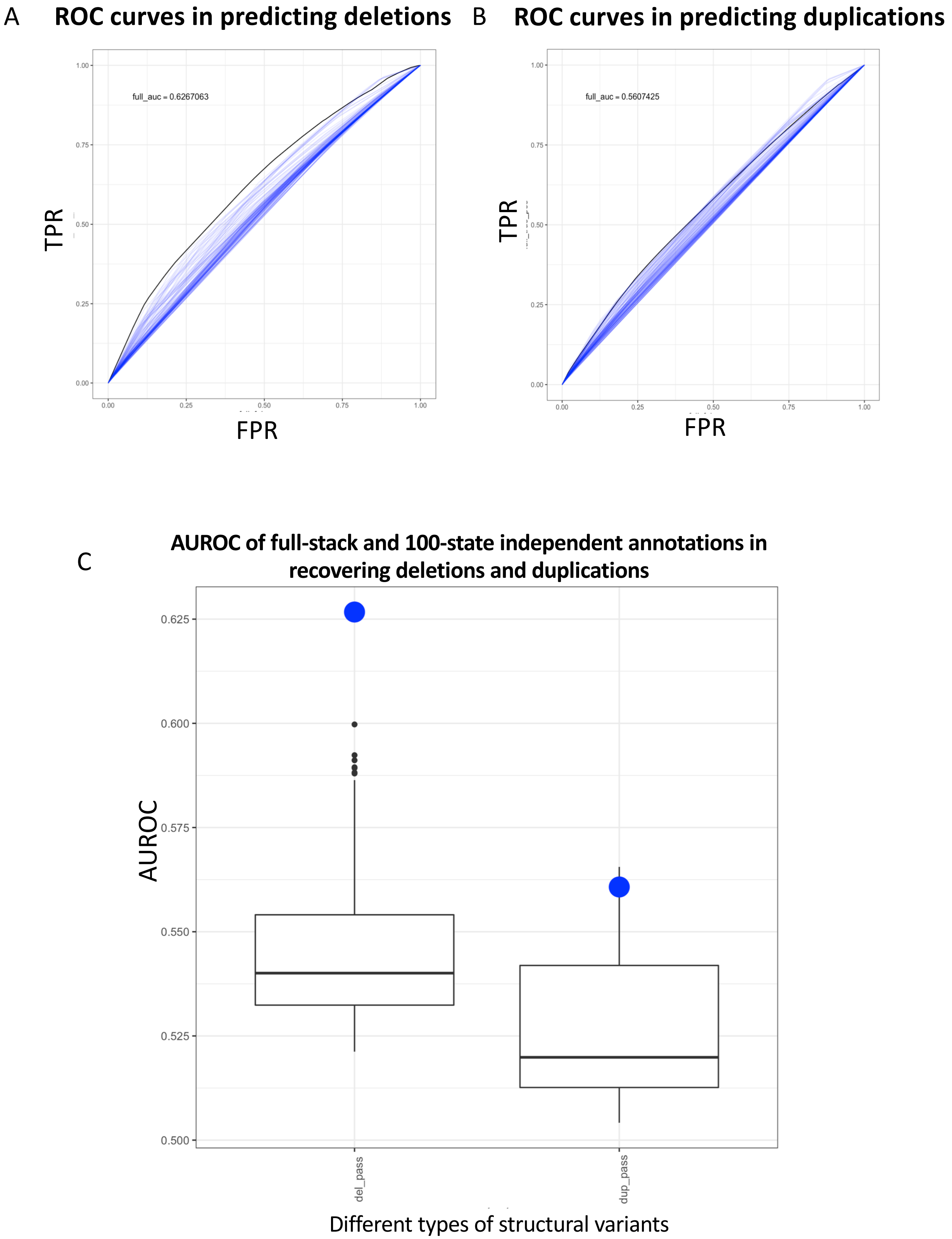

Figure S33: Comparison of full-stack model annotations and the 100-state independent model annotations in predicting structural variants of type deletions and duplications. **(A)** ROC curves for the full-stack model and the 127 100-state independent models' chromatin state annotations at predicting bases covered by deletions (**Methods**). The full-stack model's annotation ROC curve is in black and the 127 100-state independent models' annotation ROCs are shown in blue. **(B)** Similar plot as **(A),** but for duplications. **(C)** Comparison of the AUROC in predicting structural variants. The x-axis represents different types of structural variants. The box-plots show AUROC for 127 100-state independent models' in predicting deletions and duplications. The blue dots show the AUROC of the full-stack chromatin state annotation.

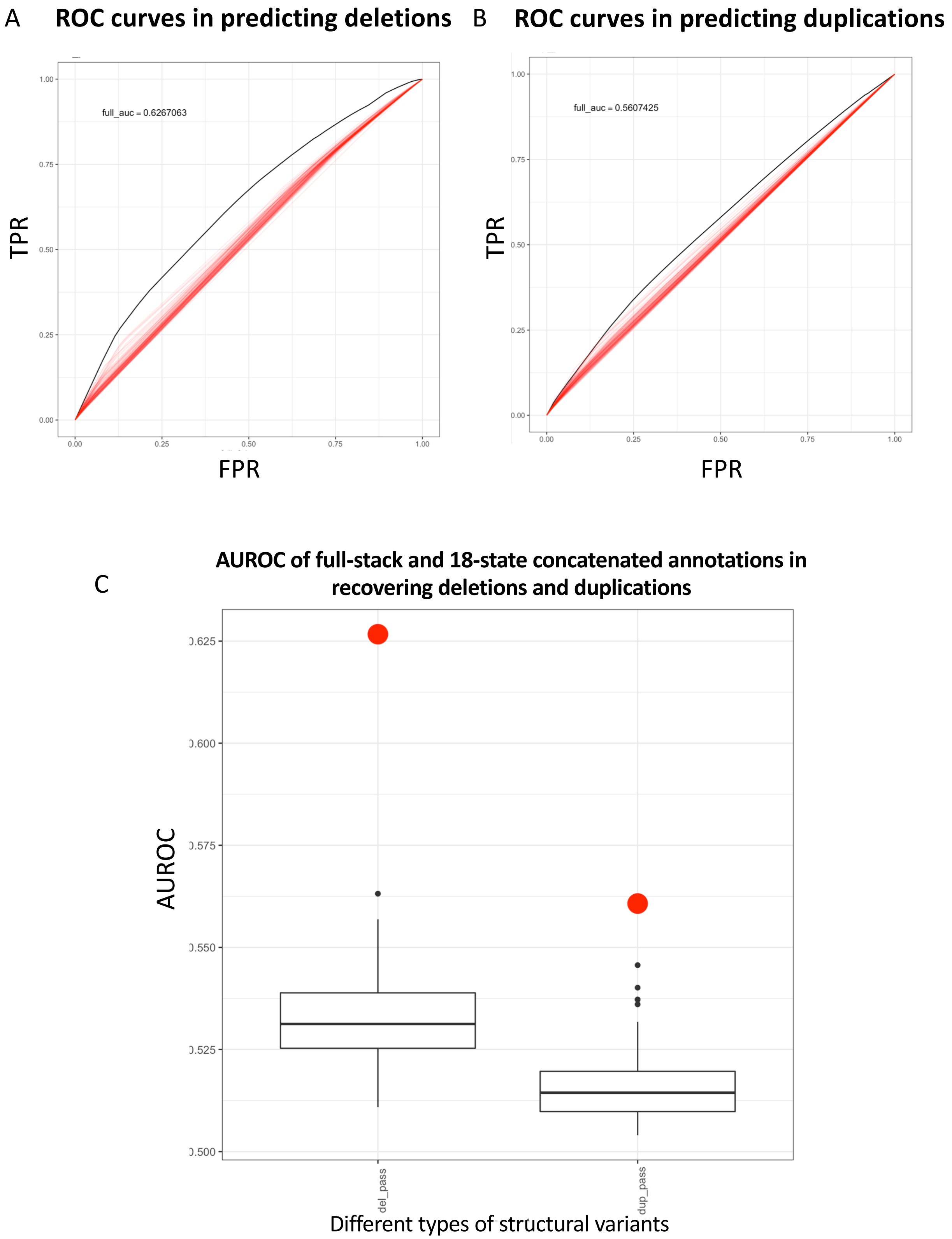

Figure S34: Comparison of full-stack model annotations and 18-state concatenated model annotations in predicting structural variants of type deletions and duplications. (**A**) ROC curves for the full-stack model and 98 concatenated models' chromatin state annotations at predicting bases covered by deletion (**Methods**). The full-stack model's annotation ROC curve is in black and the 98 18-state annotations from concatenated models ROCs are shown in red. (**B**) Similar plot as (A), but for duplications. **(C)** Comparison of the AUROC in predicting structural variants. The x-axis represents different types of structural variants. The box-plots show AUROC of 98 18-state concatenated models' in predicting deletions and duplications. The red dots show the AUROC of the full-stack chromatin state annotations in predicting bases in each type of structural variant.

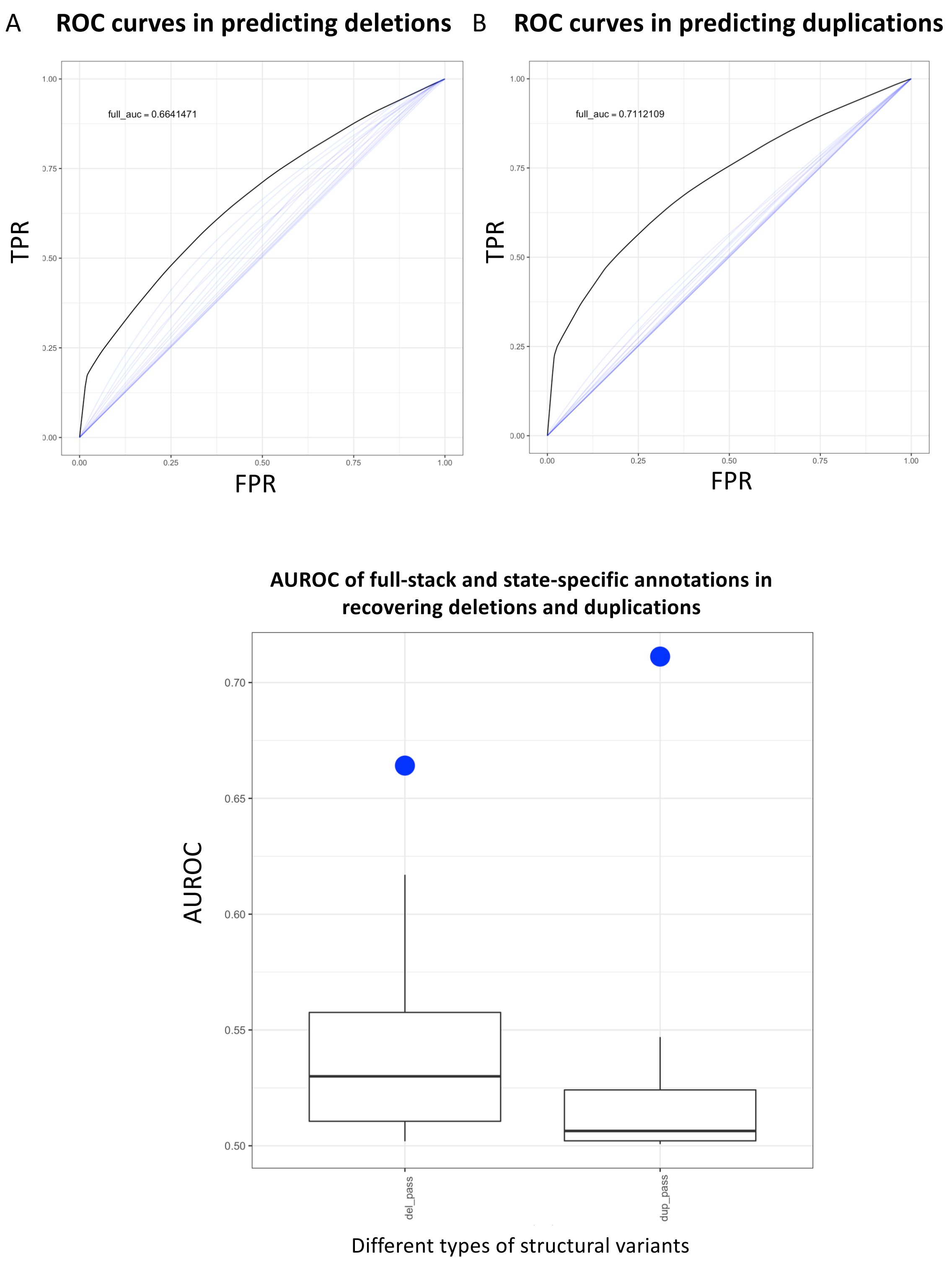

Figure S35: Comparison of full-stack states vs. state-specific annotations in predicting structural variants of types deletions and duplications. We followed the procedure outlined in (Abel et al., 2020) to compute the enrichments between annotations associated with one chromatin state and structural variants. In particular, we utilized 15-state chromatin state annotation for 127 reference epigenomes from Roadmap Epigenomics Consortium. Then, for each of the 15 states, we stratified genomic positions based on the number of cell types in which the state is present (ranging from 0 to 127), resulting in 15 state-specific models' annotations (**Methods**). **(A)** ROC curves for the full-stack model and 15 state-specific models' annotations at predicting bases covered by deletions (**Methods**) The full-stack model's ROC curve is in black, and state-specific models' ROCs are shown in blue. (**B**) Similar plot as (A), but for duplications. **(C)** Comparison of the AUROC in predicting structural variants. The x-axis represents different types of structural variants. The box-plots show AUROC of 15 state-specific models' annotation in predicting deletions and duplications. The blue dots show the AUROC of the full-stack chromatin state annotations in predicting respective types of structural variants.

A

Top 10% non-coding prioritized variants

state	% genome	FIRE	fitCons	FATHMM	GERP	LINSIGHT	PhastCons	phyloP	DANN	CDTS	CADD	REMM	Eigen	Eigen_PC	funSeq2
GapArtf2	0.05	0.2	0.3	3.1	0.1	0.2	0.3	0.1	0.2	0.1	0.2	0.3	0.4	2.2	0.7
GapArtf3	0.01	0.1	0.5	3.7	0.1	0.4	0.4	0.3	0.4	0.2	0.9	0.8	2.2	6.9	1.5
EnhA2	0.36	0.6	2.2	2.5	2.7	3.8	2	2	1	1.5	2.7	4.2	3.7	4.2	2.8
EnhA3	0.21	0.9	5.9	1.9	2.6	4.1	1.9	1.9	1.1	1.7	2.8	4.8	5.5	8.6	5.3
EnhA17	0.58	0.5	1.7	2.4	2.5	3.2	2	2	0.9	1.3	2.5	3	3	2.5	2.2
TxEnh4	0.25	5.8	6	1.3	1.4	2.4	0.8	1.1	1	2.4	1.3	3.4	2.3	4.6	4.9
Tx7	0.83	5.9	6.7	1.3	1.7	1.9	1.1	1.5	1	1.8	1.4	1.1	1.5	0.7	3.1
TxEx2	0.5	6.6	6.9	1.4	1.3	1.8	0.8	1.1	0.9	2.5	1.2	2	1.4	1.5	3.6
TxEx4	0.1	4.8	4.6	1.2	1.6	2.9	1	1.3	1.1	2.3	1.7	5.2	3.5	6.7	6.6
BivProm1	0.14	1.3	8.1	2.4	2.2	4.7	1.8	1.9	3.9	7.2	4.3	6.9	7	9.3	5
BivProm2	0.16	0.9	7	2.5	2.2	4	1.6	1.8	3	6.6	3.1	5.7	5.9	8.6	3.4
BivProm4	0.14	1.2	3.6	2.5	2.8	4.6	2.2	2.2	1.6	3.4	3.2	5.4	5.1	6.6	4.1
PromF2	0.15	3.7	6.1	1.5	1.9	3.9	1.3	1.5	1.7	4.1	2.6	5.9	6.8	9.2	6.5
PromF3	0.16	6.2	3.4	1	1.7	4.3	1.1	1.4	2.3	6.7	3	6.3	8.1	9.8	8.4
PromF4	0.19	6.7	0.9	1.6	2.3	5.7	1.8	1.8	4	7.3	4.4	7.5	8.9	9.7	8.8
PromF5	0.14	2.3	7.3	2	2.3	5.2	1.9	2	4	6.9	4.4	7.3	7.9	9.5	6.5
TSS1	0.13	5.1	3.1	2.1	2.6	5.9	2.5	2.3	4.9	5.3	4.9	7.7	7.9	9.4	7.4
TSS2	0.12	2.4	5.6	2.2	2.1	5	2.4	2.1	4.7	3.5	4.1	6.4	6.2	8.5	5

B

Top 5% non-coding prioritized variants

state	% genome	FIRE	fitCons	FATHMM	GERP	LINSIGHT	PhastCons	phyloP	DANN	CDTS	CADD	REMM	Eigen	Eigen_PC	funSeq2
GapArtf2	0.05	0	0.5	4.2	0.1	0.1	0.3	0.1	0.2	0.1	0.1	0.2	0.5	1.9	1
GapArtf3	0.01	0	1	5	0.1	0.2	0.5	0.2	0.4	0.3	0.4	0.7	2.8	9	2.5
EnhA2	0.36	0.5	3.1	3.4	3.5	4.9	3.3	2.5	0.7	1.4	3.9	5.1	4.4	3.5	3.7
EnhA3	0.21	0.6	6.3	2.6	3.2	4.9	3	2.3	0.8	1.7	3.7	5.3	6.4	12	7.8
EnhA17	0.58	0.4	2.8	3.3	3.4	4.1	3.1	2.5	0.6	1.2	3.5	3.7	3.7	1.5	2.6
TxEnh4	0.25	8.1	7.4	1.8	1.5	2.4	1.1	1.2	0.8	2.8	1.5	3.4	2.3	5.4	5.9
TxEx2	0.5	8.4	6.6	2	1.4	1.8	1	1.2	0.8	2.9	1.2	1.8	1.2	1.3	3.3
TxEx3	0.65	7.6	4.5	1.1	0.9	1.1	0.8	0.9	0.9	2.3	0.8	1.3	0.8	1.8	1.9
BivProm1	0.14	0.8	17	3.2	2.7	5.4	2.9	2.3	5.5	13	4.9	10	9.6	17	7.5
BivProm2	0.16	0.5	15	3.3	2.7	4.7	2.7	2.3	3.7	12	3.9	7.8	7.1	15	4.9
BivProm4	0.14	0.8	7	3.7	3.9	5.8	3.6	2.9	1.4	4.6	4.5	7.2	6.3	7.8	5.9
PromF2	0.15	4.2	9.7	2	2.2	4.1	2.1	1.8	1.5	5.9	3.2	7.8	9.8	16	10
PromF3	0.16	8	6.8	1.2	1.8	3.9	1.6	1.5	2.5	12	3	9.2	13	18	13
PromF4	0.19	9.2	1.9	2.2	2.8	6.1	2.9	2.3	5.6	14	4.9	12	16	19	14
PromF5	0.14	2	15	2.8	2.9	5.9	3.1	2.5	5.6	13	5.1	12	12	18	9.9
TSS1	0.13	5.7	6.4	2.8	3.2	7.2	4.3	3	7.7	10	6.4	13	13	18	12
TSS2	0.12	2.1	12	3.1	2.4	6.2	3.9	2.6	7.5	6.2	5.7	9.8	9.6	15	7.9

C

Top 1% non-coding prioritized variants

state	% genome	FIRE	fitCons	FATHMM	GERP	LINSIGHT	PhastCons	phyloP	DANN	CDTS	CADD	REMM	Eigen	Eigen_PC	funSeq2
ReprPC1	0.2	0	1.1	4.4	3.9	3.9	3.6	3.8	0.7	11	4.9	5	5.2	4.3	1.5
EnhA2	0.36	0.3	1.9	4.5	5.6	5.2	4.8	4.2	0.2	0.8	6.3	7.4	6.2	0.8	4.6
EnhA3	0.21	0.3	9.2	3.2	4.5	4.5	3.9	3.4	0.3	0.8	5.6	5.6	8	7.9	12
EnhA17	0.58	0.2	1	4.8	5.7	4.8	4.7	4.3	0.3	0.6	5.9	5.8	5.5	0.1	2.3
TxEnh4	0.25	15	17	2.9	2	2.2	1.5	1.8	0.7	2.8	2.1	2.8	2	4.1	7.8
TxEnh8	0.25	4.1	9	2	2.2	2.3	1.6	1.8	0.4	0.8	1.9	2.6	2.3	2.5	7.7
TxEx1	0.26	9.9	10	2.5	2.1	1.9	1.7	2	0.6	1.2	2	2.2	1.8	0.1	3.2
TxEx2	0.5	13	14	3.2	1.9	1.8	1.4	1.8	0.8	2.5	1.6	1.9	1.1	0.3	2.1
TxEx3	0.65	13	8.7	1.8	1	1.1	0.9	1.2	0.7	2.1	0.9	1.1	0.7	0.8	1.1
DNase1	0.22	0.3	3	10	2.2	3	2.4	2.5	2.2	2.3	2.8	3.2	4.3	10	7.1
BivProm1	0.14	0.2	1.1	4.5	2.8	4.1	4.1	3.8	8.8	52	8.7	13	11	32	14
BivProm2	0.16	0	1.3	4.8	3.5	4.2	4.2	4.1	4.8	41	7.3	8.9	8.6	16	6.9
BivProm4	0.14	0.5	1.7	5.4	6.6	5.8	5.7	5.4	0.7	7.8	8.4	9.7	9.8	6.5	8.6
PromF2	0.15	4.1	5.5	2.6	2.6	3.5	2.7	2.5	0.9	11	5	5.3	10	40	21
PromF3	0.16	11	0.8	1.2	1.4	2.6	2	1.8	2.3	35	4.2	6.7	16	64	33
PromF4	0.19	13	0.2	2.4	2.6	4.5	4	3.4	9.8	56	8.7	19	32	73	38
PromF5	0.14	0.8	1.1	3.7	3	4.2	4.4	3.9	8.9	48	9.1	17	16	52	21
TSS1	0.13	6	0.8	3	3.4	5.3	6.1	4.8	17	41	12	24	26	61	30
TSS2	0.12	1.5	2	4.2	2.5	3.7	5.3	4	19	23	9.6	14	16	40	16

State rank: 1, 2, 3, 4, 5

Fold Enrichment highlights: FE >= 1; FE < 1

Figure S36: Enrichment of selected full-stack states with prioritized variants, non-coding genome. Extended version of figure **Fig. 5C** showing fold enrichment of full-stack states for genomic bases prioritized in the **(A)** top 10% **(B)** top 5%, and **(C)** top 1% among non-coding bases by 14-different variant prioritization scores previously curated in (Arneson & Ernst, 2019) (Methods). Only states that were among the top five with greatest enrichments for at least one score are shown. Top enrichment values are colored red based on the rank of the state for each score as indicated in the color legend at the bottom. Depletions are shown in yellow.

Top 1% prioritized variants, non-coding

state	% NC genome	CADD	CDTS	DANN	Eigen_PC	Eigen	FATHMM	FIRE	GERP	LINSIGHT	PhastCons	REMM	fitCons	funSeq2	phyloP
GapArtf1	4.19	0.07	0.09	2.31	0.11	0.04	0.34	0.06	0.06	0.05	0.36	0.03	0.79	0.01	0.36
GapArtf2	0.05	0.05	0.19	0.22	1.6	0.55	2.62	0	0.04	0.05	0.29	0.09	0.36	4.06	0.17
GapArtf3	0.01	0.16	0.76	0.36	12.5	5.85	3.36	0	0.15	0.14	0.62	0.23	3.1	9.64	0.43
Quies1	10.8	0.6	0.06	0.74	0	0.39	0.65	0	0.69	0.6	0.67	0.28	0.02	0.01	0.65
Quies2	3.38	0.61	0.1	0.46	0	0.4	0.67	0	0.6	0.59	0.67	0.52	0.05	0.01	0.62
Quies3	13.4	0.51	0.22	1.03	0.03	0.39	0.6	0.56	0.66	0.62	0.67	0.27	0.24	0.02	0.7
Quies4	4.79	0.08	0.13	2.7	0	0.04	0.14	0.08	0.09	0.09	0.19	0.04	0.05	0	0.19
Quies5	1.86	0.4	2.77	1.77	0.01	0.03	0.06	0.04	0.55	0.18	0.84	0.31	0.02	0.01	0.8
HET1	0.78	0.28	0.3	0.63	0.01	0.19	0.49	0.01	0.28	0.3	0.56	0.33	0.17	0.02	0.6
HET2	0.76	0.61	0.63	0.28	0.07	0.44	0.92	0	0.62	0.69	0.76	0.73	0.13	0.02	0.8
HET3	1.5	0.06	0.12	2.72	0	0.03	0.09	0.05	0.06	0.08	0.12	0.05	0.07	0.02	0.13
HET4	0.62	0.03	0.07	1.9	0.01	0.02	0.58	0.05	0.03	0.05	0.15	0.03	0.15	0.12	0.21
HET5	0.27	0.07	0.43	1.2	0.14	0.04	0.42	0.02	0.08	0.11	0.38	0.11	0.69	0.43	0.52
HET6	0.63	0.02	0.3	1.2	0.06	0.01	0.33	0.12	0.03	0.04	0.41	0.04	0.24	0.03	0.48
HET7	1.11	0.05	0.31	1.69	0.01	0.03	0.21	0.15	0.05	0.07	0.3	0.06	0.22	0.01	0.34
HET8	0.48	0.14	0.35	0.88	0.11	0.09	0.32	0.12	0.13	0.23	0.35	0.19	0.32	0.27	0.41
HET9	1.08	0.11	0.11	1.52	0	0.06	0.4	0.21	0.11	0.11	0.41	0.09	0.09	0.04	0.41
ReprPC1	0.2	4.91	11.5	0.65	4.3	5.23	4.44	0.02	3.9	3.95	3.59	5	1.15	1.45	3.85
ReprPC2	0.36	2.33	1.41	0.33	0.48	2.06	2.28	0.14	2.31	2.25	2.13	2.07	0.6	0.4	2.13
ReprPC3	1.22	1.28	0.31	0.47	0.02	1.02	1.37	0.27	1.28	1.33	1.31	1.14	0.21	0.13	1.29
ReprPC4	4.33	0.79	0.17	0.76	0.01	0.59	0.9	0.25	0.84	0.86	0.9	0.55	0.1	0.02	0.87
ReprPC5	0.68	0.74	1.05	0.33	0.64	0.55	1.07	0.28	0.63	0.96	0.88	0.98	0.54	0.22	0.99
ReprPC6	1.65	0.56	0.48	0.38	0.18	0.4	0.83	0.43	0.53	0.74	0.75	0.64	0.3	0.06	0.82
ReprPC7	0.65	1.01	1.23	0.34	1.94	0.82	1.27	0.64	0.8	1.27	1.03	1.53	0.62	0.94	1.13
ReprPC8	0.52	0.52	4.8	1.12	0	0.01	0.02	0	0.55	0.33	1.03	0.8	0.01	0.05	1.05
ReprPC9	0.41	1.07	0.81	0.6	0.22	0.84	1.14	0.74	1.14	1.12	1.17	0.87	0.39	0.23	1.17
Acet1	0.2	0.2	1.26	0.61	1.37	0.15	0.62	0.13	0.2	0.3	0.59	0.36	1.02	0.82	0.7
Acet2	0.94	0.76	0.37	0.34	0.03	0.57	0.92	0.31	0.76	0.89	0.9	0.88	0.24	0.15	0.96
Acet3	2.91	0.37	0.28	0.59	0.01	0.25	0.48	0.5	0.4	0.48	0.51	0.35	0.16	0.03	0.54
Acet4	0.44	0.71	0.49	0.23	0.41	0.58	0.71	0.41	0.69	1.04	0.73	1.06	0.6	1.53	0.79
Acet5	0.95	0.72	0.21	0.36	0.02	0.53	0.68	0.3	0.76	0.87	0.75	0.72	0.17	0.18	0.77
Acet6	0.47	1.26	0.23	0.21	0.18	1.1	0.98	0.25	1.31	1.47	1.16	1.48	0.71	1.41	1.11
Acet7	0.31	1.66	0.61	0.2	5.21	2.12	0.95	0.33	1.34	2.01	1.3	2.42	2.52	7.82	1.24
Acet8	0.62	0.48	0.51	0.52	0.1	0.34	0.57	0.38	0.46	0.66	0.58	0.59	0.28	0.45	0.62
EnhWk1	1.7	1.7	0.22	0.41	0.01	1.29	1.45	0.19	1.87	1.73	1.7	1.27	0.19	0.19	1.54
EnhWk2	0.38	1.62	1.16	0.26	4.04	1.63	1.06	1.42	1.26	2.11	1.35	2.71	1.54	4.62	1.35
EnhWk3	0.91	2.73	0.48	0.22	0.18	2.18	2.12	0.51	2.6	2.66	2.38	2.93	0.38	1.05	2.14
EnhWk4	2.44	3.49	0.2	0.26	0.02	2.77	3.04	0.16	3.58	3.03	3.1	2.46	0.11	0.15	2.72
EnhWk5	1.09	0.89	0.47	0.62	0.05	0.71	0.84	0.6	1.03	0.97	0.97	0.69	0.27	0.16	0.95
EnhWk6	0.64	0.8	0.62	0.48	0.15	0.74	0.67	1	0.91	1.06	0.87	0.87	1.14	1.9	0.92
EnhWk7	0.53	0.79	0.36	0.56	0.14	0.76	0.69	0.68	0.84	0.94	0.84	0.93	1.55	0.61	0.83
EnhWk8	1.51	2.29	0.34	0.61	0	1.81	2.11	0.12	2.44	2.02	2.1	1.83	0.3	0.18	1.91
EnhA1	0.19	4.39	1.91	0.42	21	7.39	1.9	1.29	2.95	3.89	2.82	5.37	6.9	12.5	2.58
EnhA2	0.36	6.3	0.78	0.2	0.85	6.17	4.46	0.31	5.57	5.24	4.78	7.39	1.86	4.55	4.23
EnhA3	0.21	5.64	0.81	0.33	7.88	8	3.21	0.35	4.53	4.55	3.9	5.6	9.25	12.3	3.44
EnhA4	0.33	3.59	0.36	0.21	1.96	4.42	2.16	0.09	3.1	3.18	2.74	3.57	5.43	7.05	2.37
EnhA5	0.79	2.94	0.28	0.3	0.17	2.69	2.07	0.14	2.82	2.7	2.56	2.74	1.78	1.8	2.21
EnhA6	0.62	2.66	0.54	0.22	0.24	2.34	2.25	0.55	2.79	2.67	2.36	2.7	0.42	1.13	2.26
EnhA7	0.42	0.81	1.16	0.33	1.76	0.75	0.98	0.87	0.68	1.03	0.8	1.19	1.92	2.47	0.87
EnhA8	0.28	1.52	1.58	0.36	4.89	1.97	1.05	1.31	1.21	1.74	1.19	2.15	2.92	8.02	1.21
EnhA9	0.18	2.11	1.62	0.37	13	3.42	0.93	2.47	1.38	2.25	1.44	2.82	5.94	13.1	1.4
EnhA10	0.43	1.85	1.4	0.41	1.62	2.09	1.29	1.24	1.7	1.92	1.56	2.18	2.81	4.74	1.47
EnhA11	0.79	0.62	0.5	0.6	0.07	0.49	0.61	1	0.67	0.77	0.71	0.58	0.52	0.39	0.72
EnhA12	0.36	1.33	0.69	0.31	2.06	1.4	0.97	0.48	1.14	1.62	1.14	2.03	0.87	5.3	1.11
EnhA13	0.84	1.05	0.31	0.41	0.06	0.84	0.91	0.28	1.1	1.17	1.03	1.04	0.2	0.8	1
EnhA14	0.4	1.29	0.77	0.35	2.15	1.37	1.03	1.04	1.16	1.6	1.15	1.64	0.75	4.67	1.16
EnhA15	1.12	1.03	0.37	0.53	0.11	0.8	0.96	0.73	1.09	1.15	1.05	0.83	0.22	0.57	1.02
EnhA16	0.71	0.81	0.97	0.8	0.44	0.7	0.92	0.78	0.85	0.89	0.95	0.8	0.34	0.73	0.98
EnhA17	0.58	5.89	0.57	0.27	0.14	5.47	4.85	0.21	5.66	4.83	4.74	5.83	0.96	2.32	4.32
EnhA18	0.5	1.87	0.81	0.76	0.09	1.53	1.73	0.21	1.75	1.63	1.62	2.15	1.43	1.77	1.53
EnhA19	0.28	3.35	1.64	0.35	2.23	3.52	2.46	0.89	2.74	2.98	2.51	4.42	3.24	8.15	2.36
EnhA20	0.38	1.74	0.28	0.3	0.07	1.55	1.54	0.13	1.91	1.71	1.62	1.48	2.5	0.42	1.56
TxEnh1	0.43	1.59	0.34	0.24	0.08	1.65	0.92	0.34	1.94	2.03	1.58	1.89	1.12	2.4	1.59
TxEnh2	0.43	0.72	0.61	0.32	0.21	0.68	0.36	1.87	0.75	1.04	0.74	1.11	1.42	3.1	0.85
TxEnh3	0.27	0.44	0.83	0.53	0.72	0.37	0.55	3.18	0.43	0.61	0.63	0.71	2.14	2.59	0.72
TxEnh4	0.25	2.06	2.81	0.67	4.08	2	2.9	14.5	1.96	2.22	1.54	2.84	17.2	7.85	1.81
TxEnh5	0.49	1.25	2.04	0.57	2.35	1.21	1.91	5.89	1.33	1.6	1.15	1.82	7.23	2.82	1.37
TxEnh6	0.19	1.25	1.54	0.44	3.02	1.41	1.66	7.08	1.34	1.62	1.1	1.86	8.85	6.75	1.28
TxEnh7	0.3	1.27	0.96	0.23	3.97	1.49	0.65	1.38	1.03	1.71	1.06	2.18	2.3	5.29	1.07
TxEnh8	0.25	1.92	0.85	0.36	2.53	2.31	1.99	4.1	2.17	2.3	1.63	2.56	8.98	7.73	1.75
TxWk1	3.03	0.59	0.58	0.87	0.02	0.71	1.07	2.95	1.11	0.9	0.9	0.53	1.92	0.16	1.07
TxWk2	0.86	0.69	1.32	0.57	0.53	0.55	1.32	2.12	0.84	1.02	0.86	0.93	2.7	0.41	1.08
Tx1	0.91	0.28	0.46	0.81	0.01	0.23	0.23	0.92	0.34	0.45	0.42	0.34	0.42	0.66	0.5
Tx2	1.74	0.4	0.43	0.88	0.01	0.36	0.32	0.86	0.57	0.63	0.56	0.38	0.3	0.27	0.63
Tx3	0.56	0.42	0.53	0.4	0.24	0.38	0.33	0.94	0.47	0.73	0.53	0.65	0.48	1.1	0.59
Tx4	0.5	0.69	0.51	0.69	0.03	0.62	1	4.09	0.93	0.87	0.79	0.75	3.07	0.94	1
Tx5	1	0.37	1	0.76	0.12	0.33	0.8	9.56	0.5	0.52	0.53	0.46	3.32	0.29	0.64
Tx6	1.17	1.11	0.55	0.62	0	1.33	2.22	3.31	2.1	1.57	1.48	0.96	5.37	0.45	1.86
Tx7	0.83	1.4	0.64	0.57	0.01	1.43	2.68	7.44	2.17	1.76	1.51	1.41	7.92	1.07	1.94
Tx8	0.73	1.5	0.54	0.38	0.14	1.62	1.77	3	2.07	1.91	1.61	1.71	4.66	1.72	1.72
TxEx1	0.26	1.98	1.22	0.62	0.15	1.76	2.49	9.93	2.12	1.93	1.69	2.22	10.2	3.21	2.03
TxEx2	0.5	1.64	2.49	0.78	0.31	1.14	3.21	13.3	1.92	1.84	1.4	1.9	13.7	2.1	1.81
TxEx3	0.65	0.9	2.13	0.71	0.77	0.67	1.8	12.7	0.98	1.09	0.93	1.13	8.72	1.07	1.17
TxEx4	0.1	3.01	2.69	0.53	10.4	3.9	2.43	8.09	2.28	2.78	2.03	3.84	8.28	12.6	2.2
znf1	0.44	0.08	0.29	2.09	0.03	0.05	0.24	0.13	0.09	0.13	0.26	0.11	1.12	0.25	0.32
znf2	0.15	0.2	0.31	1.35	0.17	0.09	0.73	0.11	0.16	0.22	0.39	0.26	5.07	0.61	0.59
DNase1	0.22	2.78	2.32	2.17	10.4	4.3	10.1	0.34	2.23	2.95	2.41	3.17	3.03	7.12	2.45
BivProm1	0.14	8.74	52	8.77	31.7	11.3	4.48	0.16	2.8	4.06	4.14	12.7	1.06	13.9	3.81
BivProm2	0.16	7.25	41.1	4.76	16.1	8.61	4.81	0.04	3.45	4.2	4.22	8.94	1.25	6.89	4.14
BivProm3	0.3	4.01	5.6	1.24	6.14	4.06	3.44	0.47	2.83	3.24	3.14	5.01	1.26	3.33	3
BivProm4	0.14	8.44	7.77	0.72	6.48	9.81	5.41	0.49	6.65	5.8	5.66	9.73	1.66	8.63	5.39
PromF1	0.22	2.94	5.38	0.82	20.5	4.58	3.41	1.66	1.69	2.68	1.98	4.22	4.59	12.1	1.85
PromF2	0.15	5.02	10.8	0.92	39.6	10.5	2.57	4.13	2.6	3.48	2.75	5.25	5.45	20.9	2.52
PromF3	0.16	4.2	35.4	2.26	63.7	15.6	1.24	10.8	1.38	2.61	1.96	6.69	0.76	32.8	1.84
PromF4	0.19	8.7	55.7	9.76	72.8	31.5	2.4	13.1	2.61	4.48	4	19	0.21	37.9	3.39
PromF5	0.14	9.06	48.3	8.88	51.6	16.5	3.7	0.85	3.02	4.25	4.38	17.2	1.13	21.4	3.88
PromF6	0.14	2.68	9.81	0.52	19.8	4.8	0.91	7.92	1.53	2	1.57	2.92	0.56	16.2	1.67
PromF7	0.17	1.47	3.75	0.41	3.19	1.54	0.72	6.51	1.27	1.51	1.16	1.89	1.35	5.38	1.34
TSS1	0.13	12.1	41.2	17.4	61.4	25.7	3.01	6	3.43	5.3	6.09	23.5	0.83	30.3	4.8
TSS2	0.12	9.59	23.1	18.7	39.7	16	4.24	1.49	2.48	3.71	5.27	13.6	1.98	16	4.04
	100	0.99	0.99	0.99	0.99	0.99	0.99	1.00	1.00	0.99	1.71	1.01	1.01	0.99	0.99

Top 1% prioritized variants, whole-genome

state	% genome	CADD	CDTS	DANN	Eigen_PC	Eigen	FATHMM	FIRE	GERP	PhastCons	REMM	fitCons	phyloP
GapArtf1	12	0.07	0.03	0.31	0.04	0.01	0.1	0.02	0.01	0.15	0.01	0.17	0.13
GapArtf2	0.1	0.18	0.27	0.21	1.6	0.5	1.28	0	0.08	0.31	0.12	0.26	0.19
GapArtf3	0	0.62	0.96	0.6	12	5.25	2.22	0	0.43	0.8	0.28	0.62	0.71
Quies1	9.9	0.19	0.05	0.23	0	0.39	0.48	0	0.48	0.54	0.15	0.05	0.36
Quies2	3.1	0.28	0.09	0.19	0	0.41	0.55	0	0.47	0.57	0.42	0.16	0.45
Quies3	12	0.27	0.2	0.41	0.03	0.4	0.49	0.6	0.51	0.59	0.16	0.24	0.47
Quies4	4.5	0.05	0.11	0.73	0	0.04	0.11	0.08	0.06	0.18	0.03	0.07	0.14
Quies5	1.7	0.19	2.57	0.63	0.01	0.03	0.04	0.04	0.4	0.72	0.2	0.02	0.47
HET1	0.7	0.39	0.32	0.39	0.01	0.21	0.51	0.01	0.37	0.55	0.41	0.48	0.63
HET2	0.7	0.82	0.72	0.77	0.07	0.49	0.99	0	0.7	0.82	0.87	1.04	0.92
HET3	1.4	0.05	0.11	0.66	0	0.04	0.07	0.06	0.05	0.11	0.04	0.08	0.09
HET4	0.6	0.09	0.06	0.47	0.01	0.02	0.31	0.06	0.03	0.14	0.03	0.23	0.19
HET5	0.2	1.01	0.81	1.31	0.18	0.07	0.55	0.02	0.28	0.47	0.37	1.83	0.78
HET6	0.6	0.32	0.33	0.56	0.08	0.02	0.33	0.13	0.07	0.4	0.09	0.87	0.51
HET7	1	0.17	0.32	0.5	0.01	0.03	0.21	0.17	0.08	0.29	0.1	0.32	0.36
HET8	0.4	0.44	0.38	0.6	0.19	0.1	0.36	0.14	0.2	0.38	0.27	0.79	0.46
HET9	1	0.16	0.1	0.48	0.01	0.06	0.3	0.23	0.09	0.36	0.07	0.35	0.34
ReprPC1	0.2	4.61	12	2.45	4.95	5.45	4.49	0.02	3.69	3.58	5.07	2.86	4.12
ReprPC2	0.3	1.4	1.39	0.7	0.56	2.11	2.02	0.16	1.93	1.9	1.72	0.85	1.79
ReprPC3	1.1	0.72	0.28	0.45	0.02	1.03	1.17	0.3	1.08	1.15	0.93	0.54	1
ReprPC4	3.9	0.44	0.15	0.38	0.01	0.6	0.75	0.27	0.69	0.79	0.4	0.34	0.64
ReprPC5	0.6	1.62	1.25	1.67	0.77	0.66	1.4	0.3	0.95	1.15	1.49	2.21	1.42
ReprPC6	1.5	1.02	0.51	1.06	0.21	0.46	1	0.47	0.72	0.87	0.9	1.46	1.01
ReprPC7	0.6	2.82	1.52	2.98	2.21	1.07	2.13	0.71	1.49	1.65	2.6	3.96	2.14
ReprPC8	0.5	0.7	4.67	0.88	0	0.01	0.02	0	0.6	1.04	0.86	0.02	0.94
ReprPC9	0.4	0.61	0.78	0.43	0.26	0.87	1.03	0.8	0.93	1.05	0.7	0.39	0.91
Acet1	0.2	1.82	1.9	2.04	1.61	0.35	1.37	0.14	0.97	1.11	1.39	2.68	1.63
Acet2	0.9	0.77	0.37	0.63	0.04	0.61	0.95	0.33	0.83	0.9	0.97	0.82	0.96
Acet3	2.6	0.35	0.26	0.36	0.01	0.27	0.46	0.54	0.39	0.49	0.37	0.39	0.48
Acet4	0.4	0.75	0.45	0.62	0.47	0.62	0.77	0.45	0.75	0.77	1.1	0.8	0.78
Acet5	0.9	0.41	0.19	0.26	0.02	0.55	0.59	0.33	0.63	0.67	0.61	0.3	0.55
Acet6	0.4	0.6	0.2	0.3	0.22	1.13	0.83	0.27	1.03	1.02	1.13	0.39	0.77
Acet7	0.3	0.96	0.57	0.5	5.82	2.2	0.85	0.37	1.1	1.19	1.8	0.65	0.95
Acet8	0.6	0.48	0.49	0.47	0.12	0.36	0.57	0.41	0.48	0.58	0.62	0.52	0.59
EnhWk1	1.5	0.57	0.19	0.22	0.01	1.31	1.1	0.21	1.36	1.41	0.81	0.19	0.96
EnhWk2	0.4	1.7	1.26	1.49	4.43	1.8	1.35	1.56	1.4	1.51	2.83	1.99	1.53
EnhWk3	0.8	1.12	0.46	0.39	0.2	2.22	1.73	0.55	2.02	2.03	2.34	0.49	1.56
EnhWk4	2.2	1.14	0.18	0.19	0.02	2.8	2.34	0.18	2.67	2.54	1.65	0.2	1.85
EnhWk5	1	0.42	0.44	0.34	0.06	0.72	0.68	0.65	0.81	0.84	0.49	0.28	0.66
EnhWk6	0.6	0.54	0.58	0.45	0.18	0.77	0.6	1.07	0.8	0.81	0.71	0.47	0.7
EnhWk7	0.5	0.46	0.34	0.37	0.27	0.79	0.59	0.73	0.68	0.75	0.73	0.35	0.6
EnhWk8	1.4	0.83	0.3	0.28	0.01	1.83	1.65	0.13	1.85	1.74	1.26	0.2	1.31
EnhA1	0.2	2.67	1.99	1.13	22.3	7.57	1.75	1.46	2.49	2.57	3.68	1.42	2.17
EnhA2	0.3	2.49	0.72	0.42	1	6.22	3.52	0.35	4.29	3.96	5.58	0.52	3.11
EnhA3	0.2	2.47	0.74	0.44	8.92	8.12	2.47	0.38	3.46	3.26	3.59	0.43	2.44
EnhA4	0.3	1.45	0.33	0.33	2.33	4.48	1.68	0.1	2.32	2.3	2.32	0.36	1.6
EnhA5	0.7	1.06	0.25	0.28	0.2	2.72	1.59	0.16	2.09	2.13	1.85	0.29	1.46
EnhA6	0.6	1.3	0.55	0.63	0.27	2.41	1.95	0.6	2.36	2.11	2.32	0.77	1.85
EnhA7	0.4	1.52	1.29	1.58	2.03	0.86	1.24	0.95	0.95	1	1.51	2.03	1.15
EnhA8	0.3	1.45	1.61	1.16	5.57	2.14	1.07	1.42	1.17	1.22	2.02	1.39	1.12
EnhA9	0.2	1.62	1.67	1.04	14.1	3.61	0.97	2.73	1.27	1.43	2.19	1.3	1.25
EnhA10	0.4	0.86	1.31	0.41	1.86	2.13	1.05	1.34	1.32	1.35	1.57	0.41	1.02
EnhA11	0.7	0.4	0.47	0.39	0.08	0.51	0.53	1.07	0.55	0.64	0.47	0.41	0.52
EnhA12	0.3	1.07	0.68	0.79	2.37	1.48	0.95	0.53	1.06	1.11	1.79	1.02	0.98
EnhA13	0.8	0.49	0.28	0.28	0.08	0.86	0.75	0.3	0.85	0.89	0.76	0.3	0.66
EnhA14	0.4	1.06	0.74	0.84	2.38	1.43	1.07	1.14	1.1	1.12	1.52	1.08	1.04
EnhA15	1	0.56	0.34	0.4	0.13	0.82	0.85	0.79	0.88	0.93	0.65	0.45	0.74
EnhA16	0.6	0.5	0.92	0.49	0.5	0.73	0.79	0.84	0.69	0.85	0.62	0.43	0.74
EnhA17	0.5	2.15	0.52	0.29	0.18	5.51	3.8	0.23	4.4	3.89	4.37	0.31	3.19
EnhA18	0.5	0.98	0.78	0.48	0.11	1.56	1.48	0.24	1.45	1.41	1.8	0.45	1.24
EnhA19	0.3	1.89	1.65	0.86	2.58	3.63	2.15	1	2.35	2.26	3.7	1.05	2.01
EnhA20	0.3	0.72	0.24	0.27	0.09	1.57	1.21	0.15	1.44	1.37	0.92	0.29	1.04
TxEnh1	0.4	0.63	0.32	0.33	0.09	1.71	0.76	0.38	1.53	1.4	1.38	0.36	1.11
TxEnh2	0.4	0.44	0.55	0.33	0.24	0.73	0.37	2.01	0.66	0.72	0.88	0.37	0.59
TxEnh3	0.2	1.22	0.92	1.25	0.83	0.54	0.94	3.43	0.93	0.96	1.31	1.59	1.21
TxEnh4	0.3	11.6	4.65	11.8	4.16	3.37	7.86	14.1	7.68	5.51	9.96	15.5	8.5
TxEnh5	0.5	7.11	3.25	7.54	2.63	1.97	4.76	5.99	4.41	3.41	5.84	10.1	5.17
TxEnh6	0.2	4.83	2.02	5	3.31	1.95	3.16	7.3	3.19	2.48	4.12	6.77	3.49
TxEnh7	0.3	1.04	1.02	0.8	4.43	1.64	0.79	1.53	1.04	1.11	2	1.08	1.06
TxEnh8	0.2	4.42	1	4.13	2.76	2.81	3.5	4.28	3.94	2.8	4.13	5.27	3.66
TxWk1	2.8	1.2	0.55	1.3	0.03	0.79	1.28	3.14	1.4	1.14	0.65	1.43	1.34
TxWk2	0.8	4.88	2.02	5.3	0.65	0.97	3.35	2.19	3.02	2.43	3.53	6.85	3.7
Tx1	0.8	0.2	0.41	0.28	0.02	0.25	0.24	1.02	0.32	0.41	0.27	0.2	0.34
Tx2	1.6	0.21	0.39	0.3	0.01	0.38	0.29	0.95	0.47	0.51	0.27	0.18	0.42
Tx3	0.5	0.3	0.5	0.26	0.29	0.41	0.34	1.02	0.44	0.51	0.62	0.32	0.49
Tx4	0.5	2.27	0.54	2.33	0.03	0.86	1.73	4.31	2.01	1.49	1.66	2.84	2
Tx5	0.9	2.16	1.11	2.34	0.15	0.5	1.56	9.87	1.51	1.22	1.53	2.93	1.77
Tx6	1.1	3.33	0.54	3.29	0	1.58	2.98	3.48	3.38	2.41	1.47	3.8	3.12
Tx7	0.8	6.12	0.67	6.1	0.01	1.93	4.77	7.43	5.32	3.57	3.82	7.43	5.09
Tx8	0.7	2.36	0.52	2.15	0.17	1.82	2.24	3.16	2.71	2.04	2.22	2.7	2.36
TxEx1	0.3	9.04	2.18	8.81	0.19	2.68	6.21	9.54	7.07	4.74	7.48	11.3	7.09
TxEx2	0.6	13.1	3.66	13.5	0.36	2.32	8.73	12.4	9.42	6.2	9.82	17.4	9.87
TxEx3	0.7	7.38	3.3	7.88	0.89	1.36	4.67	12.5	4.51	3.48	5.29	10.3	5.46
TxEx4	0.1	9.49	4.54	8.66	10.4	5.16	6.3	7.94	5.69	4.73	7.72	11.6	6.7
znf1	0.4	0.65	0.36	0.98	0.05	0.09	0.42	0.16	0.35	0.42	0.34	1.05	0.57
znf2	0.2	5.7	1.02	7.38	0.18	0.2	1.26	0.12	0.84	1.1	1.69	11	2.16
DNase1	0.2	1.76	2.26	1.89	11.5	4.42	8.48	0.38	1.88	2.18	2.61	1	1.94
BivProm1	0.1	11.7	52.9	10.5	32.5	12.1	5.48	0.19	3.08	5.23	12.7	7.8	5.61
BivProm2	0.2	9.42	42.3	7.56	17.3	9.29	5.76	0.05	3.63	5.07	9.92	7.35	5.8
BivProm3	0.3	3.38	6.04	2.64	6.88	4.29	3.38	0.53	2.57	3.09	4.82	2.85	3.04
BivProm4	0.1	5.74	7.96	2.13	7.11	9.97	4.84	0.56	5.77	5.13	8.18	2.37	4.92
PromF1	0.2	2.58	5.63	1.8	21.8	4.84	3.19	1.91	1.61	1.99	3.81	2.17	1.81
PromF2	0.1	4.09	11.1	2.05	40.5	10.7	2.52	4.58	2.34	2.66	3.94	2.31	2.37
PromF3	0.2	5.61	35.9	4.12	62.6	16.1	2.2	11.5	1.91	2.63	6.38	3.15	2.78
PromF4	0.2	11.9	56.6	11.5	68.2	30.9	3.61	13.5	3.41	5.42	16.6	2.29	5.3
PromF5	0.1	10.3	49.1	9.24	51.5	16.9	4.36	1.01	2.98	5.05	14.7	5.6	4.91
PromF6	0.1	4.01	10.2	3.02	20.2	5.37	1.97	8.23	2.21	2.25	3.49	3.67	2.63
PromF7	0.2	1.77	3.73	1.44	3.38	1.72	1.05	6.9	1.45	1.41	1.97	1.69	1.49
TSS1	0.1	10.8	41	13.6	59.6	25.3	2.67	6.49	2.58	5.88	17.5	2.76	4.28
TSS2	0.1	7.96	22.6	14.1	39.7	15.9	3.71	1.59	1.8	5	9.9	3.88	3.61
	100	1.00	1.00	1.00	1.00	1.00	1.00	1.02	1.00	2.13	1.02	1.02	1.00

Figure S37: Enrichment of all full-stack states for top 1% bases prioritized by variant prioritization scores. Extended version of figure **Fig. 5C** showing the enrichment values of all full-stack states for genomic bases prioritized in the top 1% prioritized bases **(A)** in non-coding genome, and **(B)** genome-wide, by various variant prioritization scores. Coloring of enrichments is column specific. The second column in each heatmap, to the right of the state labels, shows the percentage of the background region (non-coding genome in **(A)** and whole genome in **(B)**) that each full-stack state covers. The last line in both heatmaps gives the actual percentage of the background region that is covered by each set of prioritized variants, which can differ from 1% exactly because of how ties of prioritization scores among bases were handled.

Top 5% prioritized variants, non-coding

state	% NC genome	CADD	CDTS	DANN	Eigen_PC	Eigen	FATHMM	FIRE	GERP	LINSIGHT	PhastCons	REMM	fitCons	funSeq2	phyloP
GapArtf1	4.19	0.32	0.13	1.57	0.11	0.12	0.48	0.18	0.12	0.13	0.57	0.08	0.48	0.03	0.4
GapArtf2	0.05	0.13	0.12	0.21	1.91	0.46	4.19	0.03	0.05	0.09	0.29	0.25	0.51	1.02	0.14
GapArtf3	0.01	0.39	0.34	0.42	9.02	2.83	5.02	0.03	0.09	0.19	0.51	0.72	1.04	2.53	0.23
Quies1	10.8	0.68	0.32	0.78	0.03	0.58	1	0.01	0.88	0.58	0.75	0.29	0.02	0.1	0.97
Quies2	3.38	0.59	0.36	0.54	0.04	0.49	0.82	0.01	0.68	0.54	0.6	0.41	0.08	0.12	0.75
Quies3	13.4	0.77	0.63	1.08	0.09	0.58	0.81	0.72	0.85	0.61	0.88	0.3	0.19	0.26	0.94
Quies4	4.79	0.5	0.33	1.8	0.02	0.1	0.3	0.2	0.18	0.13	0.55	0.06	0.06	0.04	0.35
Quies5	1.86	0.73	2.53	1.44	0.01	0.04	0.07	0.04	0.9	0.48	1.37	0.38	0.02	0.2	1.1
HET1	0.78	0.38	0.5	0.7	0.15	0.23	0.52	0.01	0.32	0.31	0.47	0.36	0.35	0.11	0.49
HET2	0.76	0.51	0.74	0.48	0.66	0.49	0.86	0.01	0.6	0.64	0.61	0.68	0.28	0.15	0.75
HET3	1.5	0.67	0.42	1.91	0.04	0.08	0.21	0.29	0.15	0.12	0.61	0.07	0.13	0.09	0.32
HET4	0.62	0.54	0.28	1.41	0.08	0.06	2.25	0.2	0.09	0.1	0.55	0.06	0.24	0.14	0.28
HET5	0.27	0.53	0.5	1.21	0.45	0.13	0.61	0.24	0.13	0.22	0.61	0.29	0.87	0.37	0.42
HET6	0.63	0.31	0.29	1.13	0.16	0.06	0.39	0.17	0.05	0.13	0.49	0.09	0.21	0.05	0.29
HET7	1.11	0.34	0.43	1.38	0.08	0.07	0.24	0.3	0.1	0.13	0.4	0.11	0.22	0.06	0.27
HET8	0.48	0.53	0.59	1	0.68	0.2	0.42	0.27	0.28	0.27	0.51	0.56	0.63	0.33	0.54
HET9	1.08	0.44	0.27	1.21	0.04	0.14	0.73	0.27	0.18	0.17	0.62	0.1	0.09	0.05	0.44
ReprPC1	0.2	2.55	5.34	1.13	7.87	3.79	2.76	0.34	2.37	3.33	2.15	3.69	5.48	1.91	1.96
ReprPC2	0.36	1.68	1.69	0.68	1.29	1.69	1.83	0.61	1.74	2.05	1.57	1.69	0.92	0.81	1.47
ReprPC3	1.22	1.17	0.92	0.71	0.22	0.97	1.26	0.55	1.17	1.15	1.16	0.97	0.32	0.36	1.16
ReprPC4	4.33	0.93	0.66	0.9	0.07	0.69	1.01	0.43	0.92	0.76	0.97	0.46	0.1	0.18	0.98
ReprPC5	0.68	0.7	1.31	0.64	1.98	0.71	0.96	0.42	0.75	0.88	0.73	1.27	0.7	0.46	0.9
ReprPC6	1.65	0.6	0.91	0.63	0.59	0.5	0.81	0.5	0.66	0.65	0.65	0.75	0.3	0.27	0.81
ReprPC7	0.65	0.86	1.5	0.71	3.99	1.12	1.06	0.68	0.92	1.26	0.83	1.85	1.01	1.08	1
ReprPC8	0.52	0.82	3.68	1.15	0.01	0.01	0.02	0.01	1.03	0.68	1.36	0.93	0.01	0.31	1.17
ReprPC9	0.41	1.07	1.14	0.8	0.44	0.89	1.12	0.9	1.13	1.09	1.09	0.85	0.55	0.48	1.08
Acet1	0.2	0.36	0.99	0.74	3.65	0.32	0.52	0.26	0.25	0.38	0.75	0.85	1.45	0.9	0.59
Acet2	0.94	0.73	0.85	0.54	0.36	0.59	0.89	0.39	0.82	0.75	0.74	1.11	0.57	0.42	0.88
Acet3	2.91	0.55	0.71	0.75	0.09	0.35	0.6	0.61	0.58	0.43	0.56	0.41	0.2	0.2	0.68
Acet4	0.44	0.75	1.03	0.5	1.56	0.74	0.78	0.53	0.91	0.92	0.69	1.95	1.03	1.51	0.87
Acet5	0.95	0.78	0.68	0.57	0.27	0.65	0.83	0.41	0.95	0.76	0.76	0.84	0.33	0.47	0.93
Acet6	0.47	1.23	0.79	0.49	1.44	1.26	1.19	0.38	1.46	1.41	1.1	1.93	0.98	1.49	1.19
Acet7	0.31	1.48	1.25	0.59	7.51	2.77	1.16	0.54	1.53	2.09	1.16	4.08	3.1	4.93	1.19
Acet8	0.62	0.58	0.92	0.7	0.53	0.45	0.65	0.49	0.64	0.59	0.56	0.91	0.51	0.53	0.69
EnhWk1	1.7	1.6	0.68	0.69	0.22	1.5	1.64	0.33	1.81	1.66	1.57	1.11	0.35	0.64	1.51
EnhWk2	0.38	1.42	1.68	0.75	5.21	2.11	1.11	1.34	1.45	2.15	1.14	3.35	2.12	3.63	1.2
EnhWk3	0.91	2.04	1.03	0.54	0.7	2.02	1.89	0.57	2.1	2.42	1.81	2.31	0.76	1.44	1.6
EnhWk4	2.44	2.46	0.71	0.55	0.14	2.44	2.57	0.25	2.6	2.6	2.29	1.66	0.21	0.69	1.97
EnhWk5	1.09	0.97	0.85	0.82	0.23	0.8	0.95	0.72	1.07	0.91	1	0.65	0.44	0.5	1.03
EnhWk6	0.64	0.99	1.14	0.77	1.45	0.89	0.77	1.16	1.09	1.09	0.95	1.23	1.4	1.99	1.02
EnhWk7	0.53	1.01	0.92	0.81	2.82	0.96	0.86	0.87	1.02	0.96	0.96	1.51	2.57	1.23	1
EnhWk8	1.51	1.74	0.76	0.72	0.17	1.61	1.85	0.22	1.89	1.75	1.68	1.32	0.81	0.55	1.53
EnhA1	0.19	2.94	2.32	1.09	13.8	7.27	1.88	1.53	2.48	4.09	2.13	6.79	6.4	8.17	1.83
EnhA2	0.36	3.88	1.42	0.68	3.52	4.41	3.38	0.47	3.52	4.88	3.26	5.13	3.11	3.74	2.54
EnhA3	0.21	3.74	1.66	0.8	12	6.45	2.58	0.63	3.25	4.88	2.95	5.34	6.33	7.79	2.34
EnhA4	0.33	2.73	1	0.62	8.45	3.96	2.19	0.27	2.6	3.48	2.29	3.57	4.43	4.57	1.92
EnhA5	0.79	2.39	0.87	0.68	2.52	2.54	2.12	0.34	2.37	2.77	2.16	2.36	2.47	1.91	1.85
EnhA6	0.62	1.95	1.15	0.56	0.83	1.97	1.77	0.68	2.11	2.35	1.75	2.03	0.67	1.62	1.62
EnhA7	0.42	0.8	1.49	0.64	4.15	1.01	0.9	0.96	0.82	1.02	0.73	2.28	2.53	2.11	0.89
EnhA8	0.28	1.38	1.83	0.73	8.16	2.51	1.15	1.37	1.37	1.99	1.11	3.84	3.31	5.3	1.14
EnhA9	0.18	1.8	2.12	0.86	11.6	4.47	1.04	2.34	1.6	2.57	1.28	5.55	4.78	8.59	1.28
EnhA10	0.43	1.7	1.7	0.76	5.19	2.2	1.35	1.36	1.64	2.11	1.44	2.97	3.63	3.68	1.36
EnhA11	0.79	0.84	1.02	0.85	0.59	0.65	0.78	1.13	0.85	0.78	0.84	0.77	0.88	0.68	0.9
EnhA12	0.36	1.29	1.19	0.67	5.56	1.79	1.14	0.63	1.29	1.69	1.07	3.19	2.33	3.33	1.1
EnhA13	0.84	1.12	0.79	0.67	0.97	1.02	1.1	0.47	1.26	1.13	1.07	1.25	0.56	0.88	1.14
EnhA14	0.4	1.23	1.34	0.68	3.79	1.6	1.12	1.19	1.29	1.63	1.06	2.17	1.52	3.35	1.1
EnhA15	1.12	1.08	0.88	0.77	0.48	0.92	1.06	0.87	1.19	1.09	1.05	0.76	0.37	0.86	1.09
EnhA16	0.71	1.01	1.22	0.94	1.63	0.89	0.97	0.9	0.99	0.95	1.03	1.21	0.7	1.04	1.05
EnhA17	0.58	3.53	1.19	0.62	1.5	3.67	3.32	0.38	3.36	4.09	3.12	3.66	2.83	2.57	2.51
EnhA18	0.5	1.35	1.1	0.85	0.84	1.18	1.36	0.35	1.29	1.39	1.23	1.85	3.61	1.39	1.11
EnhA19	0.28	2.24	1.85	0.81	5.47	2.84	1.94	0.94	2.02	2.85	1.82	4.23	5.68	5.19	1.59
EnhA20	0.38	1.56	0.77	0.63	0.92	1.58	1.61	0.29	1.82	1.7	1.51	1.49	3.7	1.02	1.51
TxEnh1	0.43	1.75	1.16	0.69	1.29	1.82	1.01	0.67	1.99	2.18	1.58	2.12	1.53	3.58	1.58
TxEnh2	0.43	1.16	1.4	0.78	1.76	1.08	0.48	1.85	1.19	1.34	1.06	2.66	2.19	4.36	1.22
TxEnh3	0.27	0.65	1.56	0.84	1.6	0.51	0.43	2.78	0.54	0.67	0.68	2.02	2.2	3.61	0.78
TxEnh4	0.25	1.48	2.85	0.83	5.35	2.26	1.83	8.07	1.54	2.38	1.14	3.44	7.4	5.95	1.25
TxEnh5	0.49	1.02	2.18	0.79	3.88	1.45	1.27	4.1	1.18	1.61	0.9	2.28	3.92	3.07	1.05
TxEnh6	0.19	1.18	2.08	0.75	4.8	1.77	1.21	4.8	1.3	1.8	0.95	3.02	4.72	5.27	1.1
TxEnh7	0.3	1.24	1.71	0.7	5.58	1.93	0.65	1.37	1.24	1.76	0.96	4.04	3.02	5.67	1.07
TxEnh8	0.25	1.68	1.76	0.71	4.96	2.45	1.53	3.87	1.82	2.54	1.34	3.36	4.56	5.94	1.39
TxWk1	3.03	0.91	1.19	1.08	0.13	0.74	0.87	3.29	1.06	0.91	1.02	0.49	1.25	0.94	1.09
TxWk2	0.86	0.7	1.64	0.75	1.3	0.69	0.87	1.99	0.87	0.96	0.72	1.14	1.72	1.12	0.89
Tx1	0.91	0.81	1.17	1.12	0.23	0.47	0.28	1.38	0.67	0.56	0.92	0.7	0.7	1.81	1.01
Tx2	1.74	0.81	1.02	1.09	0.14	0.57	0.38	1.32	0.84	0.66	0.9	0.47	0.39	1.09	0.96
Tx3	0.56	0.6	1.23	0.7	0.89	0.48	0.32	1.07	0.68	0.64	0.57	1.38	0.88	1.93	0.73
Tx4	0.5	0.96	1.33	0.99	0.29	0.7	0.75	4.16	0.97	0.97	0.98	0.9	2.21	2.23	1.14
Tx5	1	0.55	1.59	0.96	0.38	0.37	0.57	6.86	0.5	0.52	0.59	0.51	2.22	0.88	0.61
Tx6	1.17	1.42	1.42	1	0.17	1.3	1.66	4.61	1.81	1.67	1.49	0.79	2.65	2	1.71
Tx7	0.83	1.42	1.68	0.84	0.33	1.31	1.83	6.97	1.77	1.77	1.34	1.06	3.94	2.68	1.61
Tx8	0.73	1.51	1.43	0.75	1.11	1.52	1.41	3.25	1.74	1.94	1.38	1.67	2.7	2.81	1.45
TxEx1	0.26	1.33	1.87	0.86	0.98	1.21	1.45	6.9	1.37	1.68	1.21	2.02	5.95	4.35	1.36
TxEx2	0.5	1.24	2.87	0.76	1.26	1.22	1.96	8.42	1.44	1.81	1.04	1.83	6.57	3.32	1.22
TxEx3	0.65	0.81	2.29	0.85	1.76	0.81	1.13	7.64	0.86	1.11	0.76	1.27	4.54	1.9	0.86
TxEx4	0.1	2	2.67	0.96	7.73	3.9	1.68	5.77	1.78	2.93	1.47	6.15	7.51	9.25	1.48
znf1	0.44	0.67	0.71	1.71	0.22	0.12	0.24	1.23	0.18	0.21	0.76	0.26	1.17	0.69	0.45
znf2	0.15	0.62	0.83	1.33	0.55	0.23	0.69	1.18	0.26	0.4	0.75	0.5	3.38	1.19	0.62
DNase1	0.22	2.16	1.99	1.43	11.7	4.68	3.31	0.65	1.98	2.95	1.9	2.66	4.25	5.5	1.68
BivProm1	0.14	4.9	13.4	5.46	16.6	9.6	3.24	0.78	2.69	5.36	2.88	10.5	17.3	7.45	2.33
BivProm2	0.16	3.88	11.9	3.72	14.8	7.12	3.29	0.48	2.67	4.69	2.72	7.79	15	4.87	2.28
BivProm3	0.3	2.5	3.32	1.32	7.32	3.57	2.43	0.87	2.12	3.3	2.13	4.16	5.24	2.77	1.83
BivProm4	0.14	4.51	4.58	1.42	7.8	6.33	3.71	0.84	3.88	5.76	3.61	7.17	7	5.87	2.89
PromF1	0.22	2.13	3.47	1.26	12.9	5.55	1.98	1.94	1.67	3.01	1.59	5.37	8.38	6.76	1.44
PromF2	0.15	3.16	5.86	1.54	15.9	9.78	1.96	4.16	2.22	4.1	2.07	7.79	9.74	10.3	1.77
PromF3	0.16	2.96	11.6	2.51	18.5	13.5	1.24	8.02	1.75	3.88	1.59	9.18	6.83	13.5	1.49
PromF4	0.19	4.87	13.8	5.63	19.4	15.9	2.15	9.21	2.77	6.11	2.91	12.5	1.89	14.4	2.27
PromF5	0.14	5.13	12.8	5.58	17.6	12.4	2.77	1.95	2.85	5.86	3.1	11.7	15.5	9.86	2.45
PromF6	0.14	2.28	5.91	1.06	10.6	6.12	0.93	6.59	1.78	3.32	1.42	5.69	5.97	9.39	1.45
PromF7	0.17	1.69	3.33	0.88	3.18	2.22	0.82	5.37	1.63	2.44	1.28	4.11	4.18	5.01	1.41
TSS1	0.13	6.36	10.1	7.68	18.1	13.3	2.84	5.74	3.21	7.19	4.26	12.7	6.44	12	2.98
TSS2	0.12	5.67	6.24	7.48	14.9	9.56	3.11	2.07	2.44	6.21	3.94	9.83	11.8	7.89	2.64
	100	4.96	4.97	4.95	4.96	4.97	4.96	4.98	4.98	4.97	4.96	5.00	5.02	4.96	4.97

Top 5% prioritized variants, whole-genome

state	% genome	CADD	CDTS	DANN	Eigen_PC	Eigen	FATHMM	FIRE	GERP	PhastCons	REMM	fitCons	phyloP
GapArtf1	12	0.12	0.04	0.51	0.04	0.04	0.16	0.07	0.04	0.22	0.03	0.19	0.15
GapArtf2	0.1	0.15	0.14	0.23	1.79	0.42	3.13	0.04	0.06	0.35	0.25	0.46	0.15
GapArtf3	0	0.44	0.4	0.48	8.38	2.57	4	0.04	0.18	0.7	0.75	0.95	0.32
Quies1	9.9	0.62	0.33	0.71	0.03	0.57	0.95	0.01	0.84	0.7	0.28	0.03	0.93
Quies2	3.1	0.57	0.38	0.51	0.05	0.5	0.8	0.01	0.67	0.58	0.4	0.12	0.74
Quies3	12	0.72	0.65	1.02	0.11	0.59	0.8	0.77	0.83	0.83	0.3	0.24	0.92
Quies4	4.5	0.42	0.34	1.67	0.03	0.1	0.29	0.22	0.17	0.47	0.06	0.07	0.34
Quies5	1.7	0.67	2.58	1.36	0.01	0.04	0.07	0.04	0.88	1.25	0.38	0.02	1.07
HET1	0.7	0.41	0.53	0.71	0.18	0.27	0.55	0.01	0.36	0.49	0.39	0.44	0.52
HET2	0.7	0.63	0.81	0.58	0.74	0.57	0.94	0.01	0.69	0.7	0.75	0.5	0.82
HET3	1.4	0.56	0.44	1.81	0.04	0.09	0.2	0.32	0.14	0.49	0.07	0.15	0.31
HET4	0.6	0.47	0.3	1.36	0.09	0.07	2	0.23	0.09	0.46	0.06	0.28	0.28
HET5	0.2	0.73	0.65	1.39	0.53	0.23	0.71	0.3	0.26	0.65	0.43	1.22	0.52
HET6	0.6	0.37	0.31	1.15	0.21	0.09	0.41	0.19	0.09	0.49	0.14	0.4	0.33
HET7	1	0.33	0.45	1.33	0.1	0.08	0.25	0.33	0.11	0.38	0.13	0.28	0.29
HET8	0.4	0.57	0.64	1.03	0.75	0.25	0.46	0.3	0.32	0.52	0.57	0.79	0.57
HET9	1	0.42	0.28	1.18	0.05	0.14	0.69	0.29	0.19	0.57	0.11	0.16	0.45
ReprPC1	0.2	2.86	5.62	1.48	7.94	3.93	2.98	0.39	2.61	2.46	3.85	4.11	2.21
ReprPC2	0.3	1.7	1.79	0.74	1.36	1.74	1.87	0.67	1.77	1.62	1.7	0.91	1.51
ReprPC3	1.1	1.14	0.97	0.72	0.25	0.99	1.26	0.59	1.17	1.14	0.96	0.42	1.16
ReprPC4	3.9	0.88	0.69	0.86	0.09	0.71	1	0.47	0.91	0.94	0.47	0.17	0.98
ReprPC5	0.6	0.99	1.44	0.9	2.11	0.87	1.15	0.47	0.95	0.96	1.43	1.11	1.08
ReprPC6	1.5	0.76	0.99	0.77	0.67	0.61	0.92	0.54	0.77	0.78	0.85	0.6	0.91
ReprPC7	0.6	1.42	1.7	1.24	4.17	1.41	1.47	0.77	1.32	1.28	2.19	1.72	1.36
ReprPC8	0.5	0.88	3.81	1.21	0.02	0.01	0.02	0.01	1.07	1.35	0.97	0.02	1.22
ReprPC9	0.4	1.05	1.2	0.81	0.49	0.92	1.15	0.96	1.13	1.1	0.85	0.57	1.09
Acet1	0.2	0.75	1.22	1.1	3.84	0.57	0.83	0.29	0.56	1.04	1.15	1.84	0.87
Acet2	0.9	0.78	0.9	0.6	0.41	0.66	0.95	0.42	0.88	0.8	1.12	0.72	0.93
Acet3	2.6	0.54	0.75	0.73	0.12	0.38	0.61	0.65	0.59	0.56	0.42	0.27	0.69
Acet4	0.4	0.81	1.09	0.56	1.64	0.81	0.85	0.57	0.96	0.75	1.88	1.14	0.92
Acet5	0.9	0.75	0.72	0.55	0.3	0.67	0.82	0.45	0.94	0.74	0.81	0.38	0.92
Acet6	0.4	1.18	0.82	0.48	1.49	1.28	1.18	0.42	1.44	1.09	1.83	1.02	1.18
Acet7	0.3	1.47	1.31	0.61	7.61	2.79	1.19	0.59	1.54	1.19	3.83	3.04	1.21
Acet8	0.6	0.6	0.98	0.71	0.57	0.48	0.67	0.53	0.66	0.59	0.89	0.59	0.72
EnhWk1	1.5	1.5	0.71	0.64	0.24	1.5	1.6	0.36	1.76	1.51	1.08	0.38	1.47
EnhWk2	0.4	1.62	1.83	0.95	5.31	2.23	1.31	1.48	1.61	1.33	3.36	2.16	1.36
EnhWk3	0.8	1.98	1.09	0.54	0.75	2.04	1.89	0.61	2.08	1.8	2.24	0.82	1.6
EnhWk4	2.2	2.33	0.74	0.51	0.16	2.44	2.51	0.27	2.53	2.22	1.62	0.25	1.93
EnhWk5	1	0.92	0.89	0.79	0.25	0.81	0.94	0.76	1.05	0.97	0.64	0.47	1.02
EnhWk6	0.6	0.97	1.2	0.76	1.52	0.92	0.79	1.24	1.11	0.95	1.2	1.34	1.04
EnhWk7	0.5	0.96	0.96	0.78	2.94	0.99	0.87	0.94	1.02	0.94	1.44	2.39	1.01
EnhWk8	1.4	1.65	0.79	0.68	0.19	1.61	1.81	0.24	1.84	1.62	1.28	0.81	1.5
EnhA1	0.2	2.97	2.48	1.16	13.8	7.25	1.98	1.68	2.54	2.22	6.49	5.74	1.91
EnhA2	0.3	3.75	1.48	0.66	3.61	4.42	3.36	0.51	3.48	3.22	4.95	3.05	2.53
EnhA3	0.2	3.59	1.73	0.75	12.1	6.44	2.57	0.69	3.21	2.91	5.07	6.01	2.33
EnhA4	0.3	2.61	1.05	0.58	8.57	3.95	2.17	0.31	2.56	2.25	3.38	4.38	1.9
EnhA5	0.7	2.27	0.91	0.64	2.59	2.54	2.08	0.38	2.33	2.1	2.27	2.45	1.82
EnhA6	0.6	1.94	1.23	0.61	0.89	2.02	1.82	0.73	2.13	1.79	2.03	0.78	1.66
EnhA7	0.4	1.06	1.62	0.9	4.27	1.14	1.07	1.05	1	0.92	2.33	2.62	1.04
EnhA8	0.3	1.52	1.96	0.88	8.31	2.61	1.27	1.48	1.49	1.22	3.77	3.17	1.24
EnhA9	0.2	1.87	2.26	0.95	11.7	4.5	1.15	2.53	1.69	1.38	5.29	4.29	1.36
EnhA10	0.4	1.63	1.77	0.73	5.28	2.2	1.35	1.45	1.63	1.42	2.81	3.29	1.35
EnhA11	0.7	0.81	1.06	0.82	0.62	0.66	0.79	1.21	0.85	0.81	0.75	0.89	0.9
EnhA12	0.3	1.34	1.26	0.74	5.64	1.84	1.2	0.69	1.35	1.13	3.06	2.41	1.15
EnhA13	0.8	1.06	0.82	0.64	1.01	1.03	1.09	0.51	1.24	1.04	1.19	0.6	1.12
EnhA14	0.4	1.28	1.42	0.76	3.87	1.65	1.2	1.29	1.34	1.12	2.13	1.6	1.16
EnhA15	1	1.04	0.92	0.75	0.51	0.94	1.07	0.94	1.18	1.03	0.76	0.45	1.09
EnhA16	0.6	0.97	1.27	0.92	1.68	0.91	0.98	0.96	0.99	1.01	1.16	0.73	1.05
EnhA17	0.5	3.38	1.24	0.58	1.56	3.66	3.28	0.42	3.3	3.05	3.54	2.67	2.47
EnhA18	0.5	1.32	1.15	0.84	0.89	1.21	1.36	0.38	1.3	1.23	1.79	3.32	1.13
EnhA19	0.3	2.25	1.96	0.88	5.57	2.89	2.01	1.03	2.07	1.89	4.09	5.02	1.66
EnhA20	0.3	1.48	0.81	0.59	0.96	1.59	1.58	0.32	1.78	1.47	1.43	3.62	1.49
TxEnh1	0.4	1.67	1.24	0.66	1.36	1.86	1.04	0.74	1.98	1.57	2.04	1.44	1.59
TxEnh2	0.4	1.1	1.47	0.74	1.83	1.12	0.52	1.97	1.2	1.05	2.5	1.92	1.23
TxEnh3	0.2	0.84	1.72	1.01	1.74	0.67	0.61	3.01	0.73	0.87	2.09	2.04	0.95
TxEnh4	0.3	3.84	3.74	3.15	6.01	3.59	3.66	8.18	3.42	3.35	5.19	8.04	3.03
TxEnh5	0.5	2.52	2.75	2.25	4.38	2.29	2.43	4.29	2.34	2.23	3.42	5	2.13
TxEnh6	0.2	2.12	2.45	1.69	5.08	2.27	1.91	5.02	2	1.78	3.59	5.15	1.77
TxEnh7	0.3	1.31	1.84	0.77	5.69	2	0.76	1.51	1.32	1.06	3.88	2.5	1.15
TxEnh8	0.2	2.39	2.01	1.45	5.29	2.9	2.15	4.02	2.4	2.06	3.79	4.88	1.97
TxWk1	2.8	1.05	1.26	1.2	0.24	0.87	1.02	3.45	1.19	1.17	0.64	1.46	1.22
TxWk2	0.8	1.75	2.01	1.75	1.76	1.31	1.7	2.07	1.69	1.65	1.98	2.84	1.65
Tx1	0.8	0.73	1.23	1.05	0.26	0.49	0.3	1.52	0.67	0.87	0.67	0.67	1
Tx2	1.6	0.74	1.07	1.02	0.16	0.58	0.39	1.44	0.83	0.85	0.47	0.4	0.95
Tx3	0.5	0.58	1.29	0.67	0.93	0.5	0.35	1.17	0.69	0.58	1.3	0.81	0.74
Tx4	0.5	1.32	1.47	1.35	0.52	1.01	1.08	4.39	1.31	1.35	1.24	2.44	1.44
Tx5	0.9	0.97	1.75	1.33	0.59	0.65	0.9	7	0.86	0.98	0.88	2.42	0.94
Tx6	1.1	1.92	1.54	1.52	0.49	1.69	2.08	4.81	2.24	2.01	1.27	3.24	2.12
Tx7	0.8	2.51	1.89	1.95	0.97	2.12	2.7	7.07	2.67	2.45	2.05	4.87	2.46
Tx8	0.7	1.81	1.54	1.09	1.32	1.77	1.7	3.42	2.01	1.71	1.92	2.94	1.71
TxEx1	0.3	3.1	2.51	2.56	1.91	2.41	2.83	6.9	2.83	2.9	3.52	6.38	2.7
TxEx2	0.6	3.94	3.68	3.4	2.57	2.97	3.98	8.4	3.62	3.65	4.13	7.81	3.29
TxEx3	0.7	2.4	2.88	2.37	2.44	1.74	2.31	7.69	2.13	2.22	2.6	5.3	2.05
TxEx4	0.1	3.73	3.45	2.66	8.09	4.71	3.03	5.97	3.1	3.05	7.01	8.31	2.74
znf1	0.4	0.72	0.8	1.76	0.28	0.2	0.33	1.37	0.27	0.75	0.34	1.29	0.53
znf2	0.2	2.14	1.3	2.81	0.75	0.65	1.17	1.28	0.79	1.2	1.28	4.93	1.15
DNase1	0.2	2.17	2.07	1.48	11.8	4.66	3.36	0.72	2.02	1.95	2.63	4.11	1.73
BivProm1	0.1	5.92	13.5	6.41	15.9	9.44	3.92	0.86	3.45	3.83	10.8	13.3	3.1
BivProm2	0.2	4.8	12.1	4.61	14.4	7.21	3.88	0.54	3.33	3.54	8.16	10.8	2.96
BivProm3	0.3	2.79	3.55	1.65	7.39	3.68	2.63	0.95	2.32	2.39	4.27	4.26	2.05
BivProm4	0.1	4.63	4.82	1.67	7.9	6.37	3.86	0.93	4.03	3.8	7.11	4.97	3.06
PromF1	0.2	2.32	3.67	1.45	12.8	5.56	2.15	2.14	1.82	1.76	5.23	7.15	1.59
PromF2	0.1	3.31	6.12	1.74	15.7	9.7	2.15	4.47	2.38	2.25	7.53	7.44	1.93
PromF3	0.2	3.47	11.9	3.01	18.1	13.2	1.71	8.41	2.17	2.11	9.11	5.48	1.91
PromF4	0.2	5.99	14.1	6.63	18.3	15.1	2.91	9.4	3.59	4	12.7	3.05	3.12
PromF5	0.1	5.86	12.9	6.26	17	12	3.32	2.14	3.4	3.83	11.9	12.1	3.04
PromF6	0.1	2.72	6.28	1.55	10.7	6.27	1.36	6.96	2.14	1.89	5.75	4.4	1.82
PromF7	0.2	1.82	3.51	1.06	3.32	2.35	1.01	5.64	1.78	1.47	4.03	3.36	1.56
TSS1	0.1	6.81	10.1	8.06	17.3	12.9	3.03	6.09	3.47	4.71	12.8	5.57	3.32
TSS2	0.1	6.02	6.16	7.76	14.4	9.26	3.23	2.2	2.62	4.27	9.88	9.16	2.91
	100	5.00	5.00	5.00	5.00	4.98	5.00	5.01	5.02	5.00	5.03	5.03	5.00

Figure S38: Enrichment of all full-stack states for top 5% bases prioritized by variant prioritization scores. Analogous to Fig. S**37**, but for top 5% prioritized bases.

Top 10% prioritized variants, non-coding

state	% NC genome	CADD	CDTS	DANN	Eigen_PC	Eigen	FATHMM	FIRE	GERP	LINSIGHT	PhastCons	REMM	fitCons	funSeq2	phyloP
GapArtf1	4.19	0.53	0.14	1.21	0.14	0.14	0.48	0.3	0.17	0.17	1.26	0.1	0.24	0.12	0.45
GapArtf2	0.05	0.21	0.09	0.22	2.17	0.41	3.12	0.17	0.06	0.18	0.3	0.31	0.26	0.68	0.15
GapArtf3	0.01	0.91	0.23	0.35	6.86	2.23	3.72	0.13	0.08	0.37	0.43	0.75	0.5	1.47	0.25
Quies1	10.8	0.7	0.46	0.82	0.09	0.61	1.29	0.02	0.95	0.57	0.78	0.25	0.01	0.16	1.03
Quies2	3.38	0.59	0.48	0.61	0.14	0.48	1.06	0.02	0.76	0.51	0.59	0.44	0.04	0.16	0.8
Quies3	13.4	0.87	0.76	1.06	0.18	0.69	0.93	0.8	0.92	0.68	0.96	0.3	0.23	0.45	0.99
Quies4	4.79	0.78	0.42	1.37	0.06	0.17	0.4	0.28	0.25	0.16	1.34	0.07	0.06	0.11	0.44
Quies5	1.86	0.86	2.01	1.24	0.01	0.04	0.09	0.04	0.98	0.59	1.56	0.4	0.01	0.24	1.13
HET1	0.78	0.48	0.56	0.71	0.32	0.24	0.66	0.02	0.39	0.31	0.61	0.5	0.17	0.15	0.52
HET2	0.76	0.47	0.75	0.64	1.07	0.48	1.07	0.02	0.66	0.65	0.48	0.86	0.15	0.23	0.81
HET3	1.5	1.01	0.55	1.45	0.14	0.19	0.3	0.42	0.23	0.17	1.5	0.11	0.16	0.21	0.44
HET4	0.62	0.82	0.39	1.08	0.4	0.16	1.77	0.39	0.15	0.15	1.25	0.1	0.2	0.23	0.36
HET5	0.27	0.8	0.56	1.05	1.02	0.23	0.54	0.58	0.21	0.3	1.08	0.47	0.63	0.46	0.48
HET6	0.63	0.53	0.31	0.94	0.28	0.1	0.49	0.23	0.08	0.17	0.95	0.16	0.13	0.09	0.3
HET7	1.11	0.55	0.48	1.14	0.14	0.11	0.29	0.41	0.15	0.15	0.92	0.18	0.18	0.16	0.31
HET8	0.48	0.74	0.69	0.98	0.94	0.33	0.57	0.41	0.42	0.38	0.82	0.87	0.37	0.39	0.65
HET9	1.08	0.67	0.33	0.98	0.13	0.18	0.73	0.31	0.25	0.22	1.17	0.13	0.07	0.08	0.5
ReprPC1	0.2	1.94	3.64	1.33	5.65	3.58	2.31	0.64	1.92	2.91	1.33	3.29	2.58	1.5	1.58
ReprPC2	0.36	1.43	1.63	0.89	1.46	1.66	1.69	0.84	1.54	1.96	1.13	1.67	0.47	0.81	1.34
ReprPC3	1.22	1.12	1.07	0.84	0.5	0.99	1.35	0.7	1.18	1.14	1	1.07	0.19	0.43	1.16
ReprPC4	4.33	0.98	0.82	0.93	0.19	0.76	1.16	0.54	0.98	0.8	0.99	0.49	0.06	0.27	1.03
ReprPC5	0.68	0.7	1.28	0.84	2.15	0.82	1.13	0.51	0.87	0.94	0.58	1.58	0.36	0.51	0.94
ReprPC6	1.65	0.65	0.99	0.8	0.82	0.57	0.98	0.57	0.79	0.71	0.59	0.99	0.17	0.38	0.86
ReprPC7	0.65	0.78	1.41	0.95	3.72	1.28	1.06	0.73	0.99	1.31	0.58	2.13	0.54	1.08	0.99
ReprPC8	0.52	0.94	2.76	1.13	0.02	0.01	0.03	0.03	1.14	0.84	1.39	1.16	0.01	0.36	1.18
ReprPC9	0.41	1.04	1.16	0.89	0.57	0.96	1.18	0.93	1.12	1.16	0.96	0.92	0.32	0.57	1.07
Acet1	0.2	0.5	0.88	0.77	3.15	0.48	0.54	0.4	0.33	0.61	0.76	1.19	0.88	0.9	0.67
Acet2	0.94	0.75	0.96	0.69	0.68	0.65	1.04	0.46	0.93	0.78	0.66	1.4	0.31	0.51	0.89
Acet3	2.91	0.65	0.83	0.81	0.21	0.44	0.77	0.66	0.7	0.49	0.66	0.56	0.14	0.32	0.75
Acet4	0.44	0.76	1.12	0.71	1.91	0.93	0.9	0.62	1.03	1.02	0.57	2.4	0.75	1.33	0.91
Acet5	0.95	0.81	0.84	0.71	0.51	0.75	0.99	0.49	1.05	0.81	0.69	1.07	0.27	0.6	0.96
Acet6	0.47	1.13	0.96	0.69	2.19	1.47	1.23	0.53	1.44	1.42	0.86	2.35	0.98	1.36	1.18
Acet7	0.31	1.3	1.31	0.87	6.43	3	1.13	0.7	1.49	2.1	0.83	4.19	2.72	3.41	1.15
Acet8	0.62	0.65	0.98	0.82	0.76	0.54	0.8	0.55	0.76	0.65	0.59	1.2	0.34	0.55	0.74
EnhWk1	1.7	1.38	0.85	0.85	0.6	1.49	1.64	0.45	1.66	1.57	1.21	1.07	0.27	0.77	1.43
EnhWk2	0.38	1.21	1.62	1.03	4.41	2.22	1	1.33	1.43	2.11	0.77	3.35	1.61	2.87	1.14
EnhWk3	0.91	1.59	1.13	0.79	1.23	1.83	1.65	0.64	1.81	2.1	1.22	2.19	0.56	1.35	1.43
EnhWk4	2.44	1.84	0.88	0.78	0.53	2.1	2.18	0.31	2.12	2.17	1.52	1.34	0.16	0.83	1.71
EnhWk5	1.09	0.97	0.93	0.9	0.46	0.86	1.01	0.78	1.08	0.95	0.92	0.7	0.4	0.69	1.04
EnhWk6	0.64	1.03	1.22	0.89	1.71	1.1	0.78	1.31	1.14	1.23	0.86	1.51	1.46	1.91	1.05
EnhWk7	0.53	1.04	1.04	0.9	2.96	1.24	0.94	1.02	1.07	1.07	0.92	1.84	2.09	1.32	1.03
EnhWk8	1.51	1.42	0.89	0.84	0.52	1.47	1.68	0.3	1.63	1.52	1.32	1.19	0.45	0.66	1.39
EnhA1	0.19	2.24	2.11	1.37	8.95	5.85	1.45	1.59	2.09	3.55	1.35	5.5	5.53	5.56	1.56
EnhA2	0.36	2.71	1.46	1.03	4.25	3.7	2.53	0.63	2.66	3.78	2	4.22	2.16	2.84	2.04
EnhA3	0.21	2.77	1.68	1.15	8.57	5.5	1.89	0.94	2.55	4.06	1.89	4.84	5.91	5.27	1.95
EnhA4	0.33	2.14	1.17	0.91	7.47	3.95	1.86	0.5	2.18	3.04	1.55	3.64	4.18	3.24	1.7
EnhA5	0.79	1.9	1.03	0.91	3.47	2.48	1.87	0.53	2.02	2.43	1.52	2.32	1.85	1.67	1.66
EnhA6	0.62	1.54	1.26	0.81	1.25	1.82	1.43	0.77	1.82	2.07	1.18	1.92	0.59	1.62	1.44
EnhA7	0.42	0.81	1.43	0.83	3.64	1.28	0.94	1.04	0.94	1.21	0.61	2.58	1.76	1.83	0.92
EnhA8	0.28	1.27	1.71	0.93	5.88	2.82	1.04	1.53	1.35	2.17	0.84	3.79	2.86	3.9	1.11
EnhA9	0.18	1.59	2	1.11	7.83	4.28	0.87	2.23	1.55	2.68	0.89	4.99	4.4	6	1.21
EnhA10	0.43	1.51	1.62	0.96	4.76	2.42	1.22	1.49	1.54	2.13	1.09	3.18	2.92	2.82	1.29
EnhA11	0.79	0.91	1.09	0.93	0.92	0.78	0.9	1.2	0.93	0.9	0.84	0.96	0.7	0.8	0.95
EnhA12	0.36	1.19	1.25	0.89	4.91	2.12	1.15	0.78	1.3	1.74	0.85	3.43	1.38	2.42	1.08
EnhA13	0.84	1.08	0.92	0.81	1.45	1.16	1.21	0.61	1.28	1.18	0.94	1.5	0.37	0.83	1.14
EnhA14	0.4	1.14	1.38	0.88	3.57	1.78	1.07	1.29	1.3	1.72	0.85	2.38	1.05	2.5	1.08
EnhA15	1.12	1.05	1	0.87	0.84	1	1.1	0.97	1.19	1.12	0.94	0.83	0.31	0.9	1.09
EnhA16	0.71	1.06	1.22	0.97	2.33	1.09	1	1.01	1.04	1.08	1.01	1.48	0.42	1.04	1.07
EnhA17	0.58	2.5	1.3	0.91	2.51	3.03	2.43	0.53	2.54	3.18	1.98	3.03	1.71	2.2	2.01
EnhA18	0.5	1.15	1.1	0.92	1.29	1.12	1.28	0.44	1.16	1.23	1.09	1.95	2.05	1.23	1.02
EnhA19	0.28	1.73	1.71	1.06	4.89	2.71	1.56	1.03	1.72	2.47	1.25	3.89	3.88	3.8	1.38
EnhA20	0.38	1.37	0.92	0.81	1.95	1.71	1.56	0.45	1.67	1.63	1.15	1.64	3.15	1.03	1.44
TxEnh1	0.43	1.59	1.34	0.92	2.25	2.04	0.77	0.99	1.85	2.21	1.18	2.39	1.89	3.24	1.5
TxEnh2	0.43	1.26	1.5	0.95	2.58	1.44	0.4	1.95	1.33	1.63	0.93	3.13	2.13	4.12	1.24
TxEnh3	0.27	0.78	1.56	0.95	1.88	0.69	0.32	2.66	0.68	0.9	0.68	2.43	3.4	3.71	0.83
TxEnh4	0.25	1.27	2.43	1.01	4.61	2.32	1.31	5.85	1.39	2.4	0.78	3.42	6.02	4.88	1.14
TxEnh5	0.49	0.95	1.94	0.97	3.35	1.59	0.93	3.36	1.16	1.68	0.66	2.49	3.99	2.9	1.01
TxEnh6	0.19	1.14	1.94	0.95	4.09	2.02	0.9	3.94	1.28	1.96	0.72	3.15	4.68	4.26	1.07
TxEnh7	0.3	1.13	1.71	0.97	4.93	2.19	0.5	1.45	1.3	1.87	0.7	4.04	2.87	4.64	1.06
TxEnh8	0.25	1.49	1.78	0.95	4.87	2.66	1.1	3.6	1.65	2.5	0.95	3.57	5.22	4.62	1.3
TxWk1	3.03	1.02	1.27	1.1	0.29	0.87	0.66	3.12	1.06	1	1.01	0.52	2.66	1.48	1.09
TxWk2	0.86	0.75	1.55	0.89	1.42	0.84	0.67	1.9	0.95	1.07	0.63	1.41	1.74	1.4	0.9
Tx1	0.91	1.04	1.3	1.12	0.64	0.71	0.25	1.71	0.88	0.82	1.03	1.03	2.18	2.57	1.09
Tx2	1.74	0.96	1.14	1.09	0.35	0.74	0.32	1.49	0.95	0.82	0.97	0.6	1.35	1.68	1.01
Tx3	0.56	0.7	1.31	0.87	1.19	0.64	0.25	1.16	0.83	0.77	0.57	1.8	1.51	2.25	0.8
Tx4	0.5	1.1	1.44	1.05	0.67	0.9	0.55	3.97	1.05	1.15	0.98	1.16	5.18	2.83	1.14
Tx5	1	0.67	1.54	1	0.48	0.46	0.43	5.24	0.55	0.6	0.68	0.65	4.82	1.71	0.68
Tx6	1.17	1.47	1.56	1.11	0.47	1.51	1.19	4.55	1.7	1.8	1.23	0.77	5.31	2.48	1.59
Tx7	0.83	1.43	1.77	0.98	0.71	1.51	1.28	5.94	1.66	1.87	1.06	1.14	6.66	3.07	1.49
Tx8	0.73	1.4	1.54	0.94	1.66	1.67	1.02	3.19	1.61	1.93	1.05	1.84	4.05	2.82	1.36
TxEx1	0.26	1.23	1.8	0.98	1.48	1.25	0.99	5.76	1.26	1.66	0.93	2.16	6.09	4.42	1.23
TxEx2	0.5	1.17	2.48	0.9	1.53	1.37	1.37	6.57	1.32	1.82	0.77	1.96	6.91	3.61	1.13
TxEx3	0.65	0.83	2.01	0.96	1.76	0.93	0.82	5.65	0.85	1.18	0.66	1.46	5.79	2.49	0.86
TxEx4	0.1	1.68	2.29	1.13	6.74	3.53	1.21	4.83	1.59	2.89	0.98	5.19	4.58	6.58	1.31
znf1	0.44	1.01	0.85	1.39	0.49	0.27	0.2	1.97	0.28	0.31	1.39	0.41	2.89	1.18	0.56
znf2	0.15	0.92	0.97	1.2	0.89	0.36	0.55	2.97	0.36	0.57	1.09	0.7	5.32	1.5	0.72
DNase1	0.22	1.73	1.72	1.31	9.02	4.49	2.09	0.98	1.71	2.83	1.29	2.58	2.22	4.04	1.45
BivProm1	0.14	4.33	7.17	3.91	9.27	6.97	2.38	1.34	2.24	4.71	1.76	6.88	8.09	5.05	1.87
BivProm2	0.16	3.1	6.63	2.98	8.6	5.88	2.53	0.89	2.16	4.05	1.64	5.69	7.01	3.38	1.79
BivProm3	0.3	1.89	2.54	1.41	5.4	3.28	1.92	0.97	1.78	2.88	1.34	3.68	2.58	2.17	1.53
BivProm4	0.14	3.22	3.37	1.61	6.59	5.09	2.53	1.17	2.82	4.62	2.18	5.37	3.6	4.07	2.23
PromF1	0.22	1.75	2.66	1.39	8.17	4.79	1.52	1.95	1.54	2.98	1.07	4.73	4.77	4.55	1.3
PromF2	0.15	2.6	4.06	1.66	9.24	6.81	1.48	3.72	1.91	3.85	1.34	5.89	6.07	6.55	1.53
PromF3	0.16	2.97	6.67	2.29	9.85	8.13	1.03	6.18	1.67	4.32	1.05	6.33	3.35	8.35	1.35
PromF4	0.19	4.42	7.32	3.99	9.74	8.93	1.63	6.73	2.29	5.69	1.76	7.52	0.89	8.75	1.82
PromF5	0.14	4.39	6.9	4	9.53	7.9	1.99	2.35	2.34	5.15	1.89	7.31	7.3	6.45	1.96
PromF6	0.14	2.21	4.13	1.25	8.46	4.98	0.74	5.64	1.7	3.74	1.01	5.21	2.91	6.32	1.37
PromF7	0.17	1.71	2.73	1.07	4.24	2.48	0.66	4.61	1.62	2.84	1.01	4.42	2.32	4.08	1.37
TSS1	0.13	4.91	5.35	4.9	9.36	7.85	2.14	5.06	2.56	5.89	2.54	7.7	3.06	7.37	2.27
TSS2	0.12	4.14	3.47	4.65	8.47	6.17	2.23	2.42	2.09	5.03	2.43	6.42	5.64	4.97	2.07
	100	9.92	9.93	9.91	9.91	9.93	9.93	9.92	10.0	9.93	9.93	9.96	10.7	9.91	9.93

Top 10% prioritized variants, whole-genome

state	% genome	CADD	CDTS	DANN	Eigen_PC	Eigen	FATHMM	FIRE	GERP	PhastCons	REMM	fitCons	phyloP
GapArtf1	12	0.21	0.05	0.41	0.05	0.05	0.17	0.11	0.06	0.48	0.04	0.09	0.18
GapArtf2	0.1	0.23	0.1	0.24	2.1	0.4	2.8	0.17	0.06	0.33	0.32	0.26	0.16
GapArtf3	0	0.9	0.27	0.39	6.46	2.08	3.31	0.16	0.13	0.54	0.77	0.52	0.32
Quies1	9.9	0.7	0.49	0.82	0.1	0.63	1.33	0.03	0.97	0.79	0.26	0.02	1.05
Quies2	3.1	0.6	0.52	0.63	0.16	0.51	1.11	0.02	0.79	0.61	0.45	0.06	0.83
Quies3	12	0.88	0.81	1.08	0.2	0.73	0.97	0.86	0.95	0.98	0.32	0.25	1.02
Quies4	4.5	0.77	0.44	1.34	0.07	0.18	0.41	0.3	0.25	1.35	0.07	0.07	0.45
Quies5	1.7	0.87	2.08	1.26	0.01	0.05	0.09	0.05	1.01	1.57	0.42	0.01	1.16
HET1	0.7	0.52	0.61	0.75	0.36	0.28	0.72	0.03	0.43	0.64	0.54	0.22	0.56
HET2	0.7	0.55	0.82	0.72	1.15	0.55	1.16	0.03	0.74	0.54	0.94	0.26	0.88
HET3	1.4	1	0.6	1.46	0.15	0.2	0.31	0.45	0.24	1.51	0.12	0.16	0.46
HET4	0.6	0.82	0.42	1.1	0.44	0.17	1.77	0.44	0.16	1.26	0.11	0.22	0.38
HET5	0.2	0.94	0.67	1.19	1.13	0.32	0.65	0.7	0.3	1.14	0.58	0.79	0.56
HET6	0.6	0.59	0.34	1	0.33	0.13	0.53	0.26	0.11	0.99	0.2	0.22	0.35
HET7	1	0.57	0.51	1.15	0.16	0.13	0.31	0.45	0.17	0.95	0.2	0.22	0.34
HET8	0.4	0.8	0.75	1.04	1.02	0.39	0.63	0.46	0.47	0.87	0.93	0.46	0.7
HET9	1	0.69	0.35	1.01	0.15	0.2	0.75	0.33	0.26	1.2	0.14	0.1	0.54
ReprPC1	0.2	2.15	3.82	1.56	5.74	3.74	2.48	0.72	2.11	1.52	3.46	2.76	1.75
ReprPC2	0.3	1.5	1.74	0.97	1.56	1.75	1.77	0.91	1.62	1.19	1.75	0.55	1.41
ReprPC3	1.1	1.15	1.15	0.88	0.55	1.05	1.41	0.75	1.23	1.03	1.11	0.24	1.2
ReprPC4	3.9	0.99	0.88	0.96	0.22	0.8	1.22	0.59	1.02	1.01	0.51	0.1	1.07
ReprPC5	0.6	0.88	1.4	1.01	2.27	0.97	1.28	0.57	1.02	0.72	1.73	0.58	1.07
ReprPC6	1.5	0.76	1.07	0.9	0.91	0.66	1.09	0.62	0.89	0.68	1.09	0.32	0.95
ReprPC7	0.6	1.1	1.57	1.25	3.88	1.51	1.32	0.84	1.25	0.84	2.38	0.92	1.22
ReprPC8	0.5	1.01	2.87	1.2	0.02	0.01	0.03	0.03	1.21	1.45	1.23	0.01	1.25
ReprPC9	0.4	1.09	1.24	0.95	0.64	1.02	1.25	0.99	1.17	1.02	0.97	0.36	1.12
Acet1	0.2	0.73	1.04	0.99	3.31	0.67	0.72	0.45	0.52	0.93	1.4	1.12	0.85
Acet2	0.9	0.8	1.03	0.75	0.75	0.72	1.12	0.5	1	0.71	1.47	0.39	0.96
Acet3	2.6	0.68	0.89	0.84	0.24	0.47	0.82	0.7	0.74	0.68	0.59	0.18	0.79
Acet4	0.4	0.82	1.2	0.78	2	1.01	0.97	0.69	1.11	0.62	2.48	0.82	0.98
Acet5	0.9	0.82	0.9	0.73	0.55	0.79	1.04	0.53	1.09	0.71	1.11	0.3	1
Acet6	0.4	1.15	1.02	0.72	2.29	1.54	1.29	0.59	1.49	0.89	2.4	1.01	1.22
Acet7	0.3	1.34	1.4	0.92	6.56	3.11	1.19	0.78	1.56	0.87	4.26	2.75	1.21
Acet8	0.6	0.69	1.05	0.86	0.81	0.58	0.86	0.6	0.81	0.63	1.25	0.39	0.79
EnhWk1	1.5	1.38	0.9	0.87	0.65	1.54	1.68	0.49	1.7	1.22	1.09	0.29	1.47
EnhWk2	0.4	1.35	1.75	1.18	4.53	2.35	1.14	1.47	1.57	0.9	3.48	1.77	1.26
EnhWk3	0.8	1.61	1.21	0.83	1.31	1.9	1.71	0.7	1.87	1.25	2.24	0.6	1.48
EnhWk4	2.2	1.84	0.94	0.79	0.59	2.16	2.23	0.35	2.16	1.53	1.36	0.18	1.74
EnhWk5	1	0.98	1	0.92	0.5	0.9	1.05	0.84	1.12	0.95	0.73	0.43	1.08
EnhWk6	0.6	1.06	1.3	0.94	1.78	1.17	0.83	1.41	1.2	0.9	1.57	1.49	1.1
EnhWk7	0.5	1.07	1.12	0.94	3.06	1.32	0.99	1.09	1.13	0.94	1.9	2.11	1.08
EnhWk8	1.4	1.42	0.95	0.86	0.57	1.51	1.72	0.33	1.67	1.33	1.21	0.47	1.42
EnhA1	0.2	2.33	2.25	1.48	9	5.95	1.56	1.75	2.19	1.44	5.56	5.55	1.66
EnhA2	0.3	2.73	1.55	1.08	4.39	3.8	2.59	0.69	2.73	2.03	4.27	2.19	2.09
EnhA3	0.2	2.79	1.79	1.19	8.68	5.63	1.94	1.06	2.63	1.92	4.91	5.91	2.01
EnhA4	0.3	2.16	1.25	0.95	7.59	4.06	1.92	0.57	2.24	1.58	3.7	4.18	1.75
EnhA5	0.7	1.91	1.1	0.94	3.59	2.56	1.92	0.59	2.07	1.53	2.37	1.86	1.7
EnhA6	0.6	1.6	1.36	0.88	1.34	1.9	1.5	0.83	1.9	1.23	1.99	0.66	1.51
EnhA7	0.4	0.98	1.55	1	3.76	1.43	1.09	1.15	1.08	0.73	2.72	1.92	1.05
EnhA8	0.3	1.4	1.84	1.06	6.02	2.98	1.18	1.67	1.48	0.93	3.93	2.96	1.22
EnhA9	0.2	1.68	2.14	1.21	7.91	4.41	0.96	2.43	1.66	0.98	5.08	4.45	1.3
EnhA10	0.4	1.54	1.7	0.99	4.87	2.51	1.27	1.61	1.6	1.12	3.24	2.93	1.34
EnhA11	0.7	0.93	1.16	0.96	0.97	0.82	0.94	1.29	0.98	0.86	1	0.73	0.99
EnhA12	0.3	1.26	1.34	0.97	5.02	2.22	1.23	0.87	1.39	0.91	3.51	1.46	1.15
EnhA13	0.8	1.09	0.98	0.83	1.52	1.22	1.26	0.66	1.32	0.95	1.54	0.4	1.18
EnhA14	0.4	1.21	1.48	0.97	3.67	1.88	1.16	1.4	1.38	0.91	2.46	1.14	1.16
EnhA15	1	1.07	1.07	0.91	0.89	1.05	1.15	1.04	1.24	0.97	0.87	0.35	1.13
EnhA16	0.6	1.08	1.29	1.01	2.41	1.14	1.04	1.09	1.08	1.04	1.53	0.46	1.11
EnhA17	0.5	2.51	1.39	0.94	2.62	3.1	2.47	0.59	2.59	1.99	3.07	1.72	2.05
EnhA18	0.5	1.18	1.17	0.96	1.37	1.17	1.33	0.48	1.21	1.12	2	2.06	1.07
EnhA19	0.3	1.8	1.82	1.14	5	2.81	1.64	1.14	1.81	1.32	3.97	3.91	1.46
EnhA20	0.3	1.38	0.98	0.84	2.04	1.77	1.61	0.5	1.72	1.16	1.68	3.14	1.48
TxEnh1	0.4	1.63	1.45	0.96	2.37	2.15	0.81	1.11	1.93	1.22	2.46	1.92	1.57
TxEnh2	0.4	1.29	1.6	0.99	2.69	1.53	0.44	2.12	1.4	0.97	3.21	2.17	1.31
TxEnh3	0.2	0.92	1.71	1.09	2.02	0.82	0.43	2.89	0.82	0.81	2.58	3.51	0.96
TxEnh4	0.3	2.51	2.97	2.18	5.11	3.12	2.28	5.99	2.38	1.93	4.2	6.47	2.09
TxEnh5	0.5	1.76	2.31	1.72	3.74	2.14	1.58	3.54	1.8	1.37	3.08	4.5	1.61
TxEnh6	0.2	1.66	2.2	1.45	4.32	2.37	1.3	4.13	1.69	1.17	3.5	4.95	1.46
TxEnh7	0.3	1.21	1.85	1.06	5.07	2.31	0.58	1.6	1.39	0.78	4.14	2.94	1.15
TxEnh8	0.2	1.9	2	1.36	5.12	2.97	1.45	3.76	2	1.35	3.86	5.4	1.63
TxWk1	2.8	1.13	1.36	1.21	0.39	0.99	0.76	3.26	1.17	1.12	0.63	2.73	1.2
TxWk2	0.8	1.33	1.81	1.42	1.79	1.26	1.13	2.01	1.41	1.13	1.88	2.26	1.33
Tx1	0.8	1.06	1.39	1.15	0.69	0.76	0.28	1.89	0.92	1.06	1.07	2.21	1.14
Tx2	1.6	0.98	1.22	1.12	0.38	0.79	0.34	1.62	0.99	1	0.63	1.36	1.06
Tx3	0.5	0.73	1.41	0.9	1.24	0.69	0.27	1.26	0.87	0.6	1.86	1.54	0.84
Tx4	0.5	1.34	1.59	1.27	0.87	1.12	0.75	4.19	1.27	1.21	1.39	5.31	1.35
Tx5	0.9	0.92	1.68	1.22	0.65	0.66	0.63	5.33	0.77	0.9	0.88	4.9	0.88
Tx6	1.1	1.77	1.69	1.4	0.74	1.78	1.44	4.72	1.97	1.53	1.06	5.41	1.85
Tx7	0.8	2.02	1.98	1.55	1.21	2.01	1.76	6.04	2.15	1.65	1.68	6.79	1.96
Tx8	0.7	1.61	1.67	1.14	1.86	1.87	1.2	3.35	1.8	1.25	2.04	4.17	1.54
TxEx1	0.3	2.17	2.24	1.84	2.19	2	1.73	5.84	2.04	1.81	2.9	6.41	1.95
TxEx2	0.6	2.59	3.01	2.2	2.51	2.44	2.44	6.63	2.45	2.12	3.08	7.25	2.22
TxEx3	0.7	1.69	2.39	1.73	2.29	1.54	1.47	5.73	1.55	1.43	2.16	6.07	1.51
TxEx4	0.1	2.58	2.77	2.01	6.97	4.02	1.94	5.08	2.31	1.81	5.62	5.11	2
znf1	0.4	1.09	0.95	1.47	0.56	0.34	0.26	2.18	0.35	1.47	0.49	2.97	0.64
znf2	0.2	1.75	1.32	1.97	1.23	0.71	0.88	3.32	0.74	1.34	1.21	5.79	1.04
DNase1	0.2	1.8	1.8	1.39	9.04	4.59	2.16	1.09	1.8	1.36	2.67	2.28	1.53
BivProm1	0.1	4.8	7.2	4.44	9.06	6.93	2.78	1.48	2.71	2.29	7.11	8.09	2.32
BivProm2	0.2	3.58	6.71	3.48	8.5	5.96	2.87	1.01	2.58	2.1	5.94	7.1	2.19
BivProm3	0.3	2.1	2.7	1.63	5.49	3.41	2.08	1.09	1.95	1.51	3.85	2.75	1.7
BivProm4	0.1	3.36	3.54	1.81	6.69	5.2	2.66	1.31	2.98	2.33	5.48	3.72	2.37
PromF1	0.2	1.9	2.81	1.55	8.18	4.88	1.66	2.15	1.68	1.19	4.83	4.82	1.42
PromF2	0.1	2.74	4.23	1.84	9.21	6.86	1.62	3.98	2.06	1.47	5.96	6.08	1.66
PromF3	0.2	3.26	6.81	2.62	9.73	8.1	1.31	6.4	1.95	1.35	6.46	3.56	1.61
PromF4	0.2	4.91	7.4	4.55	9.42	8.62	2.08	6.84	2.8	2.36	7.72	1.62	2.32
PromF5	0.1	4.76	6.95	4.42	9.33	7.79	2.33	2.58	2.72	2.31	7.48	7.34	2.32
PromF6	0.1	2.48	4.36	1.56	8.54	5.14	0.99	5.89	1.96	1.28	5.39	3.13	1.61
PromF7	0.2	1.84	2.9	1.22	4.43	2.63	0.79	4.84	1.76	1.15	4.55	2.43	1.5
TSS1	0.1	5.17	5.33	5.19	9.1	7.67	2.31	5.32	2.81	2.83	7.82	3.28	2.51
TSS2	0.1	4.38	3.42	4.88	8.24	6.06	2.35	2.6	2.3	2.67	6.56	5.67	2.28
	100	10.00	10.00	10.00	9.95	9.95	10.00	10.03	10.02	10.00	10.03	10.88	10.00

Figure S39: Enrichment of all full-stack states for top 10% bases prioritized by variant prioritization scores. Analogous to **Fig. S37** and **Fig. S38**, but for top 10% prioritized bases.

A

Top 10% whole-genome prioritized variants

state	genome %	FIRE	fitCons	FATHMM	GERP	phyloP	PhastCons	DANN	CDTS	CADD	REMM	Eigen	Eigen_PC
GapArtf2	0.05	0.17	0.26	2.8	0.06	0.16	0.33	0.24	0.1	0.23	0.32	0.4	2.1
GapArtf3	0.01	0.16	0.52	3.31	0.13	0.32	0.54	0.39	0.27	0.9	0.77	2.08	6.46
EnhA2	0.33	0.69	2.19	2.59	2.73	2.09	2.03	1.08	1.55	2.73	4.27	3.8	4.39
TxEnh4	0.27	5.99	6.47	2.28	2.38	2.09	1.93	2.18	2.97	2.51	4.2	3.12	5.11
Tx7	0.82	6.04	6.79	1.76	2.15	1.96	1.65	1.55	1.98	2.02	1.68	2.01	1.21
TxEx2	0.56	6.63	7.25	2.44	2.45	2.22	2.12	2.2	3.01	2.59	3.08	2.44	2.51
BivProm1	0.15	1.48	8.09	2.78	2.71	2.32	2.29	4.44	7.2	4.8	7.11	6.93	9.06
BivProm2	0.16	1.01	7.1	2.87	2.58	2.19	2.1	3.48	6.71	3.58	5.94	5.96	8.5
BivProm4	0.13	1.31	3.72	2.66	2.98	2.37	2.33	1.81	3.54	3.36	5.48	5.2	6.69
PromF2	0.14	3.98	6.08	1.62	2.06	1.66	1.47	1.84	4.23	2.74	5.96	6.86	9.21
PromF3	0.15	6.4	3.56	1.31	1.95	1.61	1.35	2.62	6.81	3.26	6.46	8.1	9.73
PromF4	0.19	6.84	1.62	2.08	2.8	2.32	2.36	4.55	7.4	4.91	7.72	8.62	9.42
PromF5	0.14	2.58	7.34	2.33	2.72	2.32	2.31	4.42	6.95	4.76	7.48	7.79	9.33
TSS1	0.12	5.32	3.28	2.31	2.81	2.51	2.83	5.19	5.33	5.17	7.82	7.67	9.1
TSS2	0.11	2.6	5.67	2.35	2.3	2.28	2.67	4.88	3.42	4.38	6.56	6.06	8.24

B

Top 5% whole-genome prioritized variants

state	genome %	FIRE	fitCons	FATHMM	GERP	phyloP	PhastCons	DANN	CDTS	CADD	REMM	Eigen	Eigen_PC
GapArtf3	0.01	0.04	0.95	4	0.18	0.32	0.7	0.48	0.4	0.44	0.75	2.57	8.38
EnhA2	0.33	0.51	3.05	3.36	3.48	2.53	3.22	0.66	1.48	3.75	4.95	4.42	3.61
TxEnh4	0.27	8.18	8.04	3.66	3.42	3.03	3.35	3.15	3.74	3.84	5.19	3.59	6.01
TxEx2	0.56	8.4	7.81	3.98	3.62	3.29	3.65	3.4	3.68	3.94	4.13	2.97	2.57
TxEx3	0.66	7.69	5.3	2.31	2.13	2.05	2.22	2.37	2.88	2.4	2.6	1.74	2.44
TxEx4	0.1	5.97	8.31	3.03	3.1	2.74	3.05	2.66	3.45	3.73	7.01	4.71	8.09
BivProm1	0.15	0.86	13.3	3.92	3.45	3.1	3.83	6.41	13.5	5.92	10.8	9.44	15.9
BivProm2	0.16	0.54	10.8	3.88	3.33	2.96	3.54	4.61	12.1	4.8	8.16	7.21	14.4
BivProm4	0.13	0.93	4.97	3.86	4.03	3.06	3.8	1.67	4.82	4.63	7.11	6.37	7.9
PromF2	0.14	4.47	7.44	2.15	2.38	1.93	2.25	1.74	6.12	3.31	7.53	9.7	15.7
PromF3	0.15	8.41	5.48	1.71	2.17	1.91	2.11	3.01	11.9	3.47	9.11	13.2	18.1
PromF4	0.19	9.4	3.05	2.91	3.59	3.12	4	6.63	14.1	5.99	12.7	15.1	18.3
PromF5	0.14	2.14	12.1	3.32	3.4	3.04	3.83	6.26	12.9	5.86	11.9	12	17
TSS1	0.12	6.09	5.57	3.03	3.47	3.32	4.71	8.06	10.1	6.81	12.8	12.9	17.3
TSS2	0.11	2.2	9.16	3.23	2.62	2.91	4.27	7.76	6.16	6.02	9.88	9.26	14.4

C

Top 1% whole-genome prioritized variants

state	genome %	FIRE	fitCons	FATHMM	GERP	phyloP	PhastCons	DANN	CDTS	CADD	REMM	Eigen	Eigen_PC
TxEnh4	0.27	14.1	15.5	7.86	7.68	8.5	5.51	11.8	4.65	11.6	9.96	3.37	4.16
TxEx1	0.27	9.54	11.3	6.21	7.07	7.09	4.74	8.81	2.18	9.04	7.48	2.68	0.19
TxEx2	0.56	12.4	17.4	8.73	9.42	9.87	6.2	13.5	3.66	13.1	9.82	2.32	0.36
TxEx3	0.66	12.5	10.3	4.67	4.51	5.46	3.48	7.88	3.3	7.38	5.29	1.36	0.89
TxEx4	0.1	7.94	11.6	6.3	5.69	6.7	4.73	8.66	4.54	9.49	7.72	5.16	10.4
znf2	0.15	0.12	11	1.26	0.84	2.16	1.1	7.38	1.02	5.7	1.69	0.2	0.18
DNase1	0.2	0.38	1	8.48	1.88	1.94	2.18	1.89	2.26	1.76	2.61	4.42	11.5
BivProm1	0.15	0.19	7.8	5.48	3.08	5.61	5.23	10.5	52.9	11.7	12.7	12.1	32.5
BivProm2	0.16	0.05	7.35	5.76	3.63	5.8	5.07	7.56	42.3	9.42	9.92	9.29	17.3
BivProm4	0.13	0.56	2.37	4.84	5.77	4.92	5.13	2.13	7.96	5.74	8.18	9.97	7.11
PromF2	0.14	4.58	2.31	2.52	2.34	2.37	2.66	2.05	11.1	4.09	3.94	10.7	40.5
PromF3	0.15	11.5	3.15	2.2	1.91	2.78	2.63	4.12	35.9	5.61	6.38	16.1	62.6
PromF4	0.19	13.5	2.29	3.61	3.41	5.3	5.42	11.5	56.6	11.9	16.6	30.9	68.2
PromF5	0.14	1.01	5.6	4.36	2.98	4.91	5.05	9.24	49.1	10.3	14.7	16.9	51.5
TSS1	0.12	6.49	2.76	2.67	2.58	4.28	5.88	13.6	41	10.8	17.5	25.3	59.6
TSS2	0.11	1.59	3.88	3.71	1.8	3.61	5	14.1	22.6	7.96	9.9	15.9	39.7

State rank	
	1
	2
	3
	4
	5

Fold Enrichment highlights	
	FE >= 1
	FE < 1

Figure S40: Enrichment of selected full-stack states with prioritized variants, whole genome. A similar figure to **Fig. S36**, except showing the top enriched states for prioritized variants from the whole genome not restricted to non-coding regions. Fold enrichment of full-stack states for genomic bases prioritized in the **(A)** top 10% **(B)** top 5%, and **(C)** top 1% bases by 12-different variant prioritization scores (**Methods**). Only states that were among the five with greatest enrichment for at least one score are shown. Top enrichment values are colored red based on the rank of the state for the score as indicated in the color legend at the bottom. Depletions are shown in yellow.

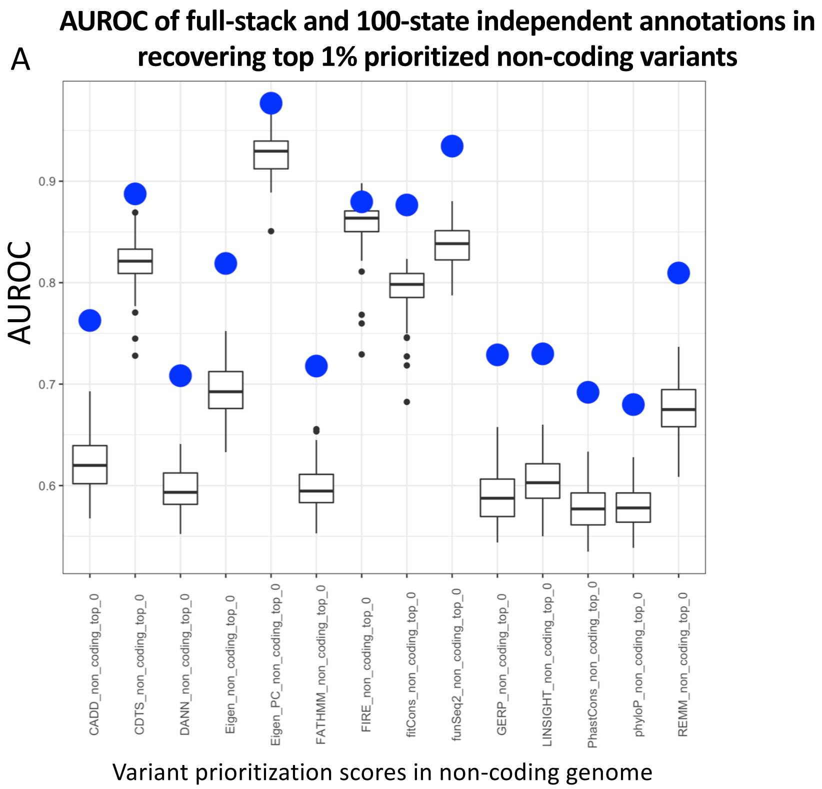

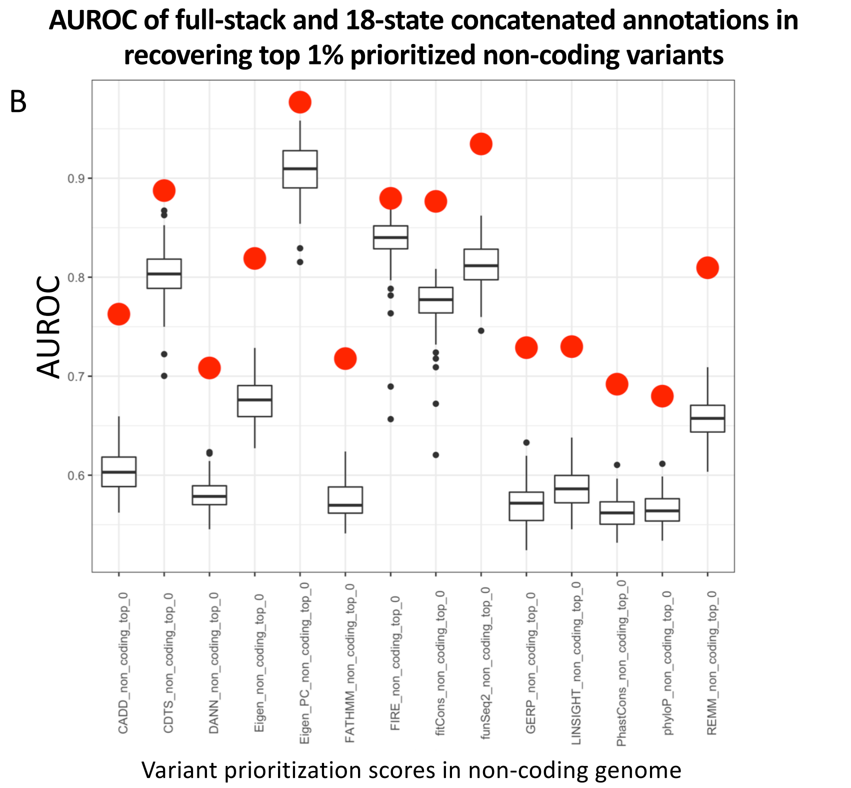

Figure S41: Comparison of the full-stack model annotations against the concatenated and independent model annotations at predicting top 1% non-coding bases prioritized by various variant prioritization scores. The box-plots show AUROC of the **(A)** 127 100-state annotations from independent models and **(B)** 98 18-state annotations from concatenated models at predicting locations of the top 1% non-coding prioritized variants. In both panels, the x-axis represents different groups of top 1% non-coding bases prioritized variants previously curated in (Arneson & Ernst, 2019), based on 14-different variant-prioritization scores (**Methods**). The **(A)** blue and **(B)** red dots show the AUROC for the full-stack chromatin state annotations.

A

Enrichment of full-stack states with GNOMAD variants

state	% Genome	maf_0_0.0001	maf_0.0001_0.0005	maf_0.0005_0.001	maf_0.001_0.005	maf_0.005_0.01	maf_0.01_0.05	maf_0.05_0.1	maf_0.1_0.2	maf_0.2_0.3	maf_0.3_0.4	maf_0.4_0.5	ucsc_snp151_common
GapArtf1	11.9	0.25	0.35	0.34	0.35	0.34	0.34	0.33	0.32	0.32	0.31	0.3	0.29
GapArtf2	0.05	0.74	1.3	1.22	1.21	1.17	1.3	1.41	1.61	1.43	1.11	1.27	2.49
GapArtf3	0.01	0.59	1.27	1.39	1.29	1.28	1.38	1.73	1.81	1.27	1	0.92	2.64
Quies1	9.88	1.12	1.1	1.12	1.14	1.15	1.17	1.19	1.19	1.22	1.23	1.24	1.18
Quies2	3.07	1.19	1.11	1.11	1.12	1.1	1.11	1.11	1.13	1.16	1.17	1.2	1.17
Quies3	12.2	1.08	1.17	1.22	1.23	1.28	1.31	1.37	1.34	1.32	1.3	1.3	1.21
Quies4	4.45	1.09	1.15	1.16	1.16	1.16	1.16	1.17	1.18	1.16	1.21	1.19	1.18
Quies5	1.69	0.69	0.93	0.9	0.87	0.87	0.85	0.82	0.76	0.74	0.73	0.7	0.78
HET1	0.71	1.26	1.19	1.18	1.18	1.15	1.16	1.12	1.16	1.16	1.21	1.19	1.24
HET2	0.69	1.29	1.29	1.25	1.26	1.25	1.28	1.26	1.3	1.32	1.35	1.31	1.39
HET3	1.36	1.07	1	1	0.99	0.97	0.96	0.94	0.96	0.98	1.01	1	1.03
HET4	0.56	1.17	1.13	1.11	1.08	1.12	1.07	0.94	0.92	0.91	0.94	1.1	1.21
HET5	0.25	1.09	1.07	1.05	1.03	1.03	1.02	1.01	1	1.08	1.01	1.02	1.3
HET6	0.58	1.28	1.35	1.31	1.29	1.28	1.28	1.21	1.22	1.22	1.24	1.21	1.39
HET7	1.02	1.23	1.36	1.33	1.34	1.34	1.33	1.34	1.36	1.34	1.38	1.35	1.37
HET8	0.43	1.11	1.07	1.06	1.06	1.04	1.05	1.04	1.13	1.14	1.13	1.08	1.17
HET9	1	1.11	1.12	1.1	1.07	1.09	1.06	1.05	1.04	0.99	1.03	1.1	1.2
ReprPC1	0.19	1.15	1.03	1.01	1	0.96	0.93	0.97	0.93	0.95	0.94	0.87	0.98
ReprPC2	0.32	1.06	0.99	0.99	0.99	0.99	0.99	1.04	1.08	1.05	0.97	1	1.03
ReprPC3	1.11	1.05	0.95	0.94	0.94	0.93	0.95	0.96	1	1.02	1	1	1.01
ReprPC4	3.93	1.07	1.02	1.03	1.04	1.06	1.08	1.12	1.14	1.16	1.17	1.18	1.11
ReprPC5	0.63	1.19	1.15	1.11	1.11	1.08	1.09	1.07	1.12	1.16	1.14	1.11	1.19
ReprPC6	1.51	1.17	1.13	1.11	1.11	1.09	1.12	1.13	1.17	1.19	1.2	1.19	1.2
ReprPC7	0.61	1.26	1.24	1.19	1.17	1.11	1.12	1.07	1.12	1.09	1.12	1.12	1.21
ReprPC8	0.48	0.68	0.86	0.83	0.8	0.75	0.72	0.65	0.58	0.57	0.58	0.55	0.66
ReprPC9	0.37	1.05	1.03	1.04	1.05	1.09	1.08	1.11	1.13	1.17	1.12	1.1	1.1
Acet1	0.18	1.38	1.86	1.97	1.97	1.96	1.99	1.99	1.98	1.92	1.88	1.85	2.09
Acet2	0.85	1.15	1.07	1.05	1.06	1.03	1.04	1.03	1.05	1.07	1.04	1.07	1.1
Acet3	2.65	1.13	1.1	1.1	1.11	1.11	1.13	1.16	1.17	1.16	1.15	1.16	1.15
Acet4	0.4	1.15	1.08	1.06	1.06	1.03	1.04	1.02	1.05	1.08	1.05	1.08	1.1
Acet5	0.86	1.1	1.04	1.04	1.05	1.03	1.06	1.04	1.1	1.09	1.11	1.09	1.09
Acet6	0.43	1.07	0.99	0.99	0.98	0.97	1	0.97	0.99	0.98	1.01	1	1.02
Acet7	0.28	1.14	1.05	1.03	1.02	0.99	0.98	0.94	0.97	0.99	1.06	1.02	1.05
Acet8	0.56	1.17	1.14	1.11	1.13	1.11	1.13	1.12	1.15	1.17	1.18	1.2	1.18
EnhWk1	1.54	1.04	0.98	0.98	1	1	1	1	1.03	1.02	1.03	1.04	1.01
EnhWk2	0.35	1.2	1.15	1.12	1.08	1.02	1.03	0.93	0.96	1	0.96	0.99	1.07
EnhWk3	0.83	1.1	1.03	1.01	1.01	0.99	1	0.99	1	1	0.99	1.06	1.02
EnhWk4	2.22	1.07	1	1	1	0.99	1	1	1.01	1	0.99	1	1
EnhWk5	0.99	1.07	1.07	1.09	1.1	1.13	1.14	1.16	1.16	1.17	1.19	1.17	1.12
EnhWk6	0.59	1.05	1.03	1.03	1.03	1.04	1.05	1.03	1.06	1.07	1.04	1.03	1.04
EnhWk7	0.48	1.07	1.04	1.06	1.04	1.08	1.07	1.12	1.13	1.2	1.16	1.16	1.11
EnhWk8	1.37	1.07	1	1	1	1	0.99	0.97	1	1.03	1.04	1.04	1.04
EnhA1	0.18	1.18	1.09	1.05	1.02	0.97	0.92	0.84	0.86	0.87	0.83	0.84	0.95
EnhA2	0.33	1.1	1.01	0.98	0.98	0.94	0.92	0.89	0.88	0.94	0.86	0.86	0.93
EnhA3	0.19	1.05	0.94	0.91	0.91	0.89	0.87	0.83	0.81	0.84	0.86	0.82	0.86
EnhA4	0.3	1.04	0.94	0.92	0.93	0.91	0.9	0.87	0.89	0.89	0.9	0.85	0.92
EnhA5	0.71	1.03	0.94	0.95	0.94	0.94	0.92	0.93	0.94	0.92	0.94	0.94	0.94
EnhA6	0.56	1.09	1	0.99	0.98	0.95	0.93	0.9	0.93	0.96	0.94	0.9	0.95
EnhA7	0.39	1.16	1.11	1.07	1.07	1.06	1.06	1.04	1.1	1.09	1.1	1.11	1.16
EnhA8	0.25	1.12	1.06	1.04	1.02	1.03	1.02	1	1.01	1.06	1.02	0.99	1.07
EnhA9	0.16	1.14	1.05	1.02	0.98	0.94	0.91	0.87	0.89	0.87	0.78	0.79	0.93
EnhA10	0.39	1.05	1	1.01	0.99	0.99	0.99	0.98	1	0.99	0.99	0.96	0.98
EnhA11	0.72	1.07	1.07	1.09	1.1	1.13	1.14	1.19	1.2	1.18	1.22	1.17	1.13
EnhA12	0.33	1.12	1.05	1.03	1.01	1	1	0.97	0.98	1.04	1	1.03	1.05
EnhA13	0.76	1.06	1	1.01	1	1	1.02	1.02	1.05	1.07	1.06	1.07	1.04
EnhA14	0.37	1.11	1.04	1.02	1.01	1	1	0.97	1.01	1.02	1.04	1.07	1.05
EnhA15	1.02	1.07	1.03	1.04	1.05	1.06	1.07	1.08	1.11	1.11	1.15	1.16	1.08
EnhA16	0.65	1.07	1.06	1.08	1.08	1.1	1.1	1.12	1.13	1.15	1.14	1.13	1.11
EnhA17	0.53	1.06	0.95	0.94	0.93	0.91	0.9	0.86	0.88	0.89	0.86	0.87	0.91
EnhA18	0.46	1.16	1.11	1.09	1.09	1.08	1.08	1.07	1.09	1.1	1.09	1.11	1.15
EnhA19	0.26	1.14	1.05	1.03	1.02	0.96	0.97	0.92	0.94	0.94	0.92	0.93	1.01
EnhA20	0.35	1.04	0.96	0.97	0.97	0.97	0.97	0.95	0.97	1	1.02	1.04	1
TxEnh1	0.39	1.03	0.93	0.92	0.91	0.89	0.86	0.81	0.81	0.77	0.8	0.81	0.86
TxEnh2	0.39	1.09	0.98	0.97	0.95	0.93	0.86	0.82	0.79	0.76	0.71	0.73	0.83
TxEnh3	0.25	1.2	1.11	1.08	1.04	1.01	0.93	0.83	0.84	0.8	0.77	0.76	0.92
TxEnh4	0.27	1.22	1.13	1.05	0.99	0.9	0.85	0.75	0.72	0.66	0.71	0.72	0.86
TxEnh5	0.5	1.22	1.17	1.11	1.06	1	0.97	0.89	0.88	0.87	0.9	0.94	1.01
TxEnh6	0.19	1.14	1.05	1	0.96	0.92	0.89	0.9	0.85	0.8	0.8	0.8	0.92
TxEnh7	0.27	1.19	1.11	1.06	1.03	0.98	0.93	0.85	0.81	0.87	0.87	0.86	0.95
TxEnh8	0.24	1.1	0.98	0.93	0.92	0.86	0.81	0.75	0.78	0.75	0.77	0.75	0.86
TxWk1	2.8	1.06	1.08	1.09	1.09	1.11	1.09	1.08	1.04	1.03	1.02	1.01	1.04
TxWk2	0.84	1.16	1.12	1.09	1.07	1.04	1.02	0.98	0.99	0.98	0.95	0.96	1.06
Tx1	0.82	1.09	1.04	1.03	1.02	1.01	0.96	0.91	0.88	0.84	0.84	0.8	0.88
Tx2	1.58	1.08	1.09	1.1	1.1	1.12	1.1	1.09	1.04	1.03	1.02	1.01	1.01
Tx3	0.51	1.14	1.09	1.06	1.06	1.01	1.01	0.96	0.93	0.96	0.96	0.95	1.02
Tx4	0.47	1.08	1	0.98	0.95	0.94	0.87	0.85	0.8	0.78	0.75	0.72	0.86
Tx5	0.94	1.15	1.19	1.17	1.15	1.16	1.11	1.09	1.06	1.03	1.04	1.02	1.05
Tx6	1.11	1	0.9	0.88	0.88	0.86	0.8	0.77	0.73	0.73	0.71	0.74	0.81
Tx7	0.82	1.03	0.9	0.87	0.84	0.81	0.75	0.68	0.67	0.68	0.63	0.64	0.75
Tx8	0.68	1.04	0.95	0.94	0.91	0.91	0.88	0.82	0.82	0.83	0.82	0.82	0.88
TxEx1	0.27	1.14	1	0.96	0.9	0.85	0.79	0.72	0.67	0.65	0.61	0.66	0.8
TxEx2	0.56	1.13	1	0.94	0.88	0.82	0.76	0.67	0.66	0.63	0.6	0.62	0.76
TxEx3	0.66	1.21	1.17	1.1	1.05	1.01	0.95	0.89	0.85	0.87	0.84	0.83	0.95
TxEx4	0.1	1.22	1.07	1.02	0.93	0.88	0.8	0.73	0.72	0.69	0.64	0.68	0.84
znf1	0.41	1.1	1.08	1.05	1.05	1.01	0.98	0.94	0.91	0.92	0.96	0.93	1
znf2	0.15	1.1	1.06	0.99	1.01	0.97	0.9	0.84	0.94	0.82	0.95	0.93	0.97
DNase1	0.2	1.16	1.15	1.15	1.14	1.13	1.15	1.14	1.14	1.14	1.12	1.11	1.16
BivProm1	0.15	1.29	1.11	1.05	1.03	0.97	0.95	0.9	0.9	0.82	0.81	0.87	0.99
BivProm2	0.16	1.25	1.09	1.06	1.03	0.97	0.94	0.95	0.89	0.87	0.85	0.83	1
BivProm3	0.29	1.2	1.15	1.1	1.09	1.05	1.05	1.02	1.04	1	0.98	0.97	1.1
BivProm4	0.13	1.09	0.99	0.97	0.96	0.94	0.93	0.9	0.9	0.88	0.84	0.84	0.9
PromF1	0.2	1.21	1.13	1.08	1.06	1.04	1	0.97	0.96	0.96	0.94	0.97	1.05
PromF2	0.14	1.17	1.04	1.01	0.95	0.91	0.89	0.81	0.8	0.81	0.78	0.8	0.89
PromF3	0.15	1.22	1.02	0.97	0.94	0.89	0.84	0.77	0.77	0.68	0.71	0.69	0.86
PromF4	0.19	1.53	1.29	1.23	1.16	1.05	0.95	0.86	0.8	0.79	0.74	0.8	0.98
PromF5	0.14	1.3	1.12	1.08	1.06	0.96	0.95	0.92	0.85	0.83	0.85	0.82	0.98
PromF6	0.13	1.13	0.97	0.95	0.92	0.87	0.84	0.83	0.78	0.77	0.71	0.76	0.86
PromF7	0.16	1.08	0.96	0.96	0.94	0.93	0.89	0.88	0.83	0.84	0.84	0.82	0.89
TSS1	0.12	1.68	1.65	1.52	1.43	1.33	1.22	1.16	1.07	1.02	1.02	1.1	1.28
TSS2	0.11	1.5	1.73	1.63	1.55	1.46	1.38	1.35	1.29	1.18	1.15	1.2	1.35
	100	4.95	0.96	0.24	0.41	0.14	0.22	0.07	0.07	0.04	0.03	0.02	0.52

B

CG Sites enrichments

State	% Genome	CGSites
GapArtf1	12	0.4
GapArtf2	0.1	1.9
GapArtf3	0	2.5
Quies1	9.9	0.6
Quies2	3.1	0.9
Quies3	12	1
Quies4	4.5	0.7
Quies5	1.7	0.8
HET1	0.7	1.2
HET2	0.7	2
HET3	1.4	0.5
HET4	0.6	0.5
HET5	0.2	1.1
HET6	0.6	1.5
HET7	1	1.5
HET8	0.4	1
HET9	1	0.7
ReprPC1	0.2	2.4
ReprPC2	0.3	1.1
ReprPC3	1.1	0.8
ReprPC4	3.9	0.8
ReprPC5	0.6	1.8
ReprPC6	1.5	1.5
ReprPC7	0.6	2.3
ReprPC8	0.5	0.9
ReprPC9	0.4	1
Acet1	0.2	2.1
Acet2	0.9	1.2
Acet3	2.6	1.2
Acet4	0.4	1.4
Acet5	0.9	0.9
Acet6	0.4	0.8
Acet7	0.3	1.4
Acet8	0.6	1.5
EnhWk1	1.5	0.6
EnhWk2	0.4	2
EnhWk3	0.8	1.1
EnhWk4	2.2	0.7
EnhWk5	1	0.9
EnhWk6	0.6	1
EnhWk7	0.5	1
EnhWk8	1.4	0.7
EnhA1	0.2	1.9
EnhA2	0.3	1.2
EnhA3	0.2	1
EnhA4	0.3	0.8
EnhA5	0.7	0.6
EnhA6	0.6	1.1
EnhA7	0.4	1.7
EnhA8	0.3	1.5
EnhA9	0.2	1.6
EnhA10	0.4	1.1
EnhA11	0.7	1.1
EnhA12	0.3	1.4
EnhA13	0.8	0.8
EnhA14	0.4	1.4
EnhA15	1	0.9
EnhA16	0.6	1.1
EnhA17	0.5	0.9
EnhA18	0.5	1.3
EnhA19	0.3	1.6
EnhA20	0.3	0.6
TxEnh1	0.4	0.7
TxEnh2	0.4	0.9
TxEnh3	0.2	1.7
TxEnh4	0.3	2.2
TxEnh5	0.5	2.2
TxEnh6	0.2	1.6
TxEnh7	0.3	1.7
TxEnh8	0.2	1.3
TxWk1	2.8	1.1
TxWk2	0.8	1.8
Tx1	0.8	0.9
Tx2	1.6	1
Tx3	0.5	1.5
Tx4	0.5	1
Tx5	0.9	1.8
Tx6	1.1	0.6
Tx7	0.8	0.7
Tx8	0.7	0.9
TxEx1	0.3	1.3
TxEx2	0.6	1.7
TxEx3	0.7	2.2
TxEx4	0.1	1.8
znf1	0.4	1
znf2	0.2	1.2
DNase1	0.2	1.9
BivProm1	0.1	7.3
BivProm2	0.2	5.8
BivProm3	0.3	2.6
BivProm4	0.1	1.9
PromF1	0.2	2.4
PromF2	0.1	2.4
PromF3	0.2	4.2
PromF4	0.2	8.6
PromF5	0.1	7
PromF6	0.1	1.9
PromF7	0.2	1.3
TSS1	0.1	10
TSS2	0.1	9.7
	100	1.8

Figure S42: Full-stack states enrichments with variants from GNOMAD stratified by minor allele frequencies, common variants (A) and CpG dinucleotides (B). In each subpanel, each row corresponds to a full-stack state. The first column gives the state labels, the second gives the percent of the genome that each state covers. The heatmap colors are on a column specific coloring scale. The last row shows the percentage of the genome that each group of variants occupy. **(A)** The last column shows the enrichment of full-stack states with common variants from UCSC Genome Browser' snp151 track (Methods). Other columns show fold enrichments of full-stack states for GNOMAD variants with the specified ranges of MAF, which are ordered in increasing MAF (Karczewski et al., 2020). **(B)** The last column shows fold enrichment of full-stack states with CpG dinucleotides**.** The three states showing highest enrichment if variants of lowest MAF (0 < MAF <= 0.0001) (TSS1-2, PromF4) are also the states most enriched states with CpG dinucleotide sites, likely reflecting the higher mutation rates associated with CpG dinucleotide sites (Karczewski et al., 2020). We note the distinction between this panel, which shows the enrichments of states with CpG dinucleotide sites, and Fig. S28B, which highlights the relative higher enrichments of TSS-associated states with regions that are rich with G, C or both nucleotides and hence with low-complexity repeat class.

	background: genome		background: common snps		background: genome		
state	% background	gwas_catalog	% background	gwas_catalog bg: ucsc_snp151_common	autosomal	chrX	chrY
GapArtf1	11.861	0.254	3.449	0.873	0.871	1.142	6.888
GapArtf2	0.050	0.267	0.125	0.107	0.911	0.716	6.047
GapArtf3	0.012	0.279	0.031	0.106	0.923	0.473	6.106
Quies1	9.883	0.737	11.671	0.624	1.048	0.349	0.355
Quies2	3.070	0.793	3.586	0.679	1.054	0.323	0.131
Quies3	12.226	1.098	14.755	0.910	1.057	0.240	0.235
Quies4	4.452	0.803	5.259	0.679	0.930	2.184	1.317
Quies5	1.692	0.180	1.323	0.230	0.087	18.310	0.044
HET1	0.706	0.738	0.874	0.597	1.053	0.389	0.028
HET2	0.692	1.021	0.964	0.733	1.058	0.301	0.004
HET3	1.359	0.950	1.397	0.925	1.005	1.273	0.035
HET4	0.563	0.949	0.683	0.784	1.024	0.808	0.319
HET5	0.249	0.940	0.325	0.721	1.053	0.251	0.384
HET6	0.581	0.723	0.808	0.520	1.006	1.196	0.174
HET7	1.023	0.952	1.405	0.693	0.980	1.533	0.581
HET8	0.435	1.228	0.508	1.050	1.044	0.541	0.047
HET9	0.998	0.637	1.195	0.531	0.929	2.391	0.831
ReprPC1	0.191	1.540	0.188	1.565	1.056	0.339	0.000
ReprPC2	0.325	1.602	0.334	1.560	1.051	0.429	0.000
ReprPC3	1.107	1.536	1.124	1.513	1.062	0.239	0.003
ReprPC4	3.935	1.262	4.385	1.132	1.069	0.108	0.008
ReprPC5	0.628	1.547	0.746	1.302	1.065	0.176	0.001
ReprPC6	1.513	1.356	1.818	1.129	1.073	0.032	0.003
ReprPC7	0.614	1.573	0.739	1.305	1.065	0.178	0.000
ReprPC8	0.477	0.173	0.314	0.263	0.046	19.086	0.011
ReprPC9	0.375	1.025	0.412	0.933	1.018	1.016	0.094
Acet1	0.184	1.154	0.386	0.552	1.047	0.494	0.024
Acet2	0.855	1.209	0.936	1.104	1.069	0.096	0.018
Acet3	2.649	1.113	3.035	0.971	1.064	0.123	0.201
Acet4	0.403	1.348	0.443	1.224	1.064	0.186	0.001
Acet5	0.860	1.214	0.934	1.117	1.059	0.293	0.000
Acet6	0.428	1.210	0.435	1.190	1.057	0.322	0.002
Acet7	0.285	1.479	0.298	1.415	1.064	0.190	0.000
Acet8	0.562	1.386	0.661	1.179	1.051	0.388	0.113
EnhWk1	1.540	1.046	1.561	1.033	1.044	0.529	0.115
EnhWk2	0.352	1.535	0.377	1.435	1.066	0.165	0.000
EnhWk3	0.830	1.289	0.844	1.267	1.051	0.426	0.012
EnhWk4	2.216	1.038	2.209	1.042	1.057	0.313	0.033
EnhWk5	0.994	1.171	1.114	1.045	1.024	0.815	0.329
EnhWk6	0.588	1.472	0.612	1.414	1.030	0.766	0.140
EnhWk7	0.484	1.509	0.540	1.354	1.047	0.496	0.034
EnhWk8	1.369	0.945	1.419	0.912	1.010	1.129	0.165
EnhA1	0.179	1.605	0.170	1.689	1.071	0.068	0.000
EnhA2	0.328	1.315	0.307	1.407	1.061	0.258	0.000
EnhA3	0.194	1.478	0.166	1.725	1.059	0.293	0.000
EnhA4	0.301	1.245	0.275	1.361	1.052	0.424	0.002
EnhA5	0.714	1.158	0.671	1.233	1.042	0.578	0.056
EnhA6	0.563	1.111	0.537	1.164	1.051	0.429	0.001
EnhA7	0.393	1.647	0.454	1.425	1.045	0.541	0.021
EnhA8	0.255	1.747	0.272	1.633	1.041	0.611	0.040
EnhA9	0.162	1.568	0.151	1.678	1.057	0.333	0.002
EnhA10	0.395	1.486	0.389	1.509	1.044	0.542	0.044
EnhA11	0.715	1.217	0.807	1.078	1.041	0.604	0.033
EnhA12	0.332	1.376	0.347	1.316	1.051	0.444	0.004
EnhA13	0.764	1.149	0.795	1.105	1.037	0.689	0.041
EnhA14	0.366	1.474	0.385	1.401	1.056	0.344	0.019
EnhA15	1.017	1.214	1.097	1.126	1.040	0.599	0.105
EnhA16	0.645	1.137	0.715	1.026	1.024	0.905	0.075
EnhA17	0.526	1.215	0.477	1.338	1.045	0.533	0.017
EnhA18	0.457	1.114	0.526	0.969	1.013	1.120	0.060
EnhA19	0.257	1.372	0.260	1.354	1.054	0.383	0.001
EnhA20	0.346	1.052	0.346	1.054	1.026	0.807	0.243
TxEnh1	0.391	1.265	0.335	1.476	1.060	0.268	0.012
TxEnh2	0.391	1.384	0.325	1.666	1.056	0.339	0.023
TxEnh3	0.250	1.603	0.230	1.741	1.063	0.214	0.005
TxEnh4	0.267	1.858	0.229	2.164	1.069	0.102	0.000
TxEnh5	0.498	1.484	0.502	1.472	1.061	0.245	0.012
TxEnh6	0.189	1.531	0.174	1.661	1.058	0.293	0.012
TxEnh7	0.270	1.526	0.257	1.606	1.070	0.077	0.000
TxEnh8	0.243	1.603	0.210	1.856	1.066	0.151	0.000
TxWk1	2.800	1.152	2.899	1.113	1.029	0.766	0.215
TxWk2	0.842	1.336	0.893	1.260	1.039	0.649	0.028
Tx1	0.824	1.279	0.728	1.447	1.049	0.448	0.060
Tx2	1.580	1.208	1.602	1.192	1.035	0.673	0.144
Tx3	0.508	1.297	0.517	1.275	1.058	0.300	0.019
Tx4	0.470	1.326	0.402	1.548	1.054	0.349	0.098
Tx5	0.942	1.393	0.989	1.326	1.044	0.538	0.097
Tx6	1.109	1.183	0.899	1.459	1.038	0.647	0.067
Tx7	0.819	1.209	0.617	1.606	1.061	0.255	0.000
Tx8	0.681	1.302	0.599	1.480	1.055	0.343	0.041
TxEx1	0.265	1.600	0.212	2.005	1.064	0.190	0.000
TxEx2	0.556	1.630	0.424	2.137	1.066	0.152	0.000
TxEx3	0.663	1.606	0.633	1.683	1.052	0.410	0.002
TxEx4	0.098	1.952	0.083	2.314	1.065	0.181	0.000
znf1	0.406	1.247	0.407	1.242	1.039	0.623	0.072
znf2	0.152	1.087	0.147	1.119	1.063	0.212	0.000
DNase1	0.201	1.371	0.232	1.187	1.032	0.768	0.037
BivProm1	0.145	1.760	0.144	1.776	1.054	0.388	0.002
BivProm2	0.159	1.536	0.159	1.539	1.051	0.434	0.002
BivProm3	0.286	1.483	0.315	1.343	1.045	0.525	0.040
BivProm4	0.130	1.550	0.117	1.718	1.032	0.777	0.021
PromF1	0.201	1.775	0.212	1.684	1.057	0.333	0.002
PromF2	0.138	2.049	0.123	2.301	1.058	0.313	0.002
PromF3	0.153	1.847	0.132	2.137	1.063	0.220	0.000
PromF4	0.190	1.453	0.187	1.477	1.046	0.536	0.000
PromF5	0.135	1.667	0.133	1.694	1.043	0.589	0.002
PromF6	0.129	1.331	0.111	1.551	1.043	0.581	0.000
PromF7	0.160	1.545	0.143	1.732	1.038	0.665	0.029
TSS1	0.123	1.686	0.158	1.315	1.011	1.162	0.033
TSS2	0.114	1.567	0.154	1.160	0.991	1.375	0.450
	100	0.0029	100	0.5556	93.07	5.02	1.92

Figure S43: Full-stack states enrichments with GWAS catalog variants (Welter et al., 2014) and sex chromosomes. Each row corresponds to a full-stack state. The first column gives the state labels and the second column shows the percentage in the genome that each full-stack state occupies. The third column shows the fold enrichments of full-stack states with GWAS catalog variants against the whole-genome. The fourth column shows the percentage of the background context (UCSC snp151 common variants) that each full-stack state occupies. The fifth column shows fold enrichments with GWAS catalog variants against the background of common variants (**Methods**). The sixth, seventh and eight columns report the autosomal, chrX, and chrY fold enrichments, respectively. Columns are colored on a column specific coloring scale. The last row reports the percent of the background context (whole genome and set of common variants) that each annotation category covers.

Enrichment of full-stack states with fine-mapped variants

state	% background	caviar	finemap
GapArtf1	3.4	0.3	0.3
GapArtf2	0.1	0.1	0.1
GapArtf3	0	0.2	0.2
Quies1	12	0.5	0.5
Quies2	3.6	0.6	0.6
Quies3	15	0.8	0.8
Quies4	5.3	0.5	0.5
Quies5	1.3	0.1	0.1
HET1	0.9	0.5	0.5
HET2	1	0.5	0.5
HET3	1.4	0.7	0.7
HET4	0.7	0.7	0.7
HET5	0.3	0.6	0.6
HET6	0.8	0.5	0.5
HET7	1.4	0.6	0.6
HET8	0.5	0.8	0.8
HET9	1.2	0.4	0.4
ReprPC1	0.2	2.3	2.3
ReprPC2	0.3	1.7	1.7
ReprPC3	1.1	1.3	1.3
ReprPC4	4.4	0.9	0.9
ReprPC5	0.7	1.2	1.2
ReprPC6	1.8	1	1
ReprPC7	0.7	1.4	1.4
ReprPC8	0.3	0.1	0.1
ReprPC9	0.4	1.1	1.1
Acet1	0.4	0.5	0.5
Acet2	0.9	1.1	1.1
Acet3	3	1	1
Acet4	0.4	1.5	1.5
Acet5	0.9	1.1	1.1
Acet6	0.4	1.2	1.2
Acet7	0.3	1.9	1.9
Acet8	0.7	1.1	1.1
EnhWk1	1.6	1.1	1.1
EnhWk2	0.4	2.2	2.2
EnhWk3	0.8	1.5	1.5
EnhWk4	2.2	1.1	1.1
EnhWk5	1.1	1.2	1.2
EnhWk6	0.6	1.6	1.6
EnhWk7	0.5	1.3	1.3
EnhWk8	1.4	0.9	0.9
EnhA1	0.2	2.9	2.9
EnhA2	0.3	2	2
EnhA3	0.2	2.6	2.6
EnhA4	0.3	1.6	1.6
EnhA5	0.7	1.3	1.3
EnhA6	0.5	1.4	1.4
EnhA7	0.5	1.6	1.6
EnhA8	0.3	2.3	2.3
EnhA9	0.2	2.7	2.7
EnhA10	0.4	1.8	1.8
EnhA11	0.8	1.3	1.3
EnhA12	0.3	1.3	1.3
EnhA13	0.8	0.9	0.9
EnhA14	0.4	1.5	1.5
EnhA15	1.1	1.1	1.1
EnhA16	0.7	1.2	1.2
EnhA17	0.5	1.5	1.5
EnhA18	0.5	0.9	0.9
EnhA19	0.3	1.6	1.6
EnhA20	0.3	1.3	1.3
TxEnh1	0.3	2	2
TxEnh2	0.3	2.2	2.2
TxEnh3	0.2	2.1	2.1
TxEnh4	0.2	2.9	2.9
TxEnh5	0.5	2	2
TxEnh6	0.2	2.6	2.6
TxEnh7	0.3	2.5	2.5
TxEnh8	0.2	2.4	2.4
TxWk1	2.9	1.2	1.2
TxWk2	0.9	1.5	1.5
Tx1	0.7	1.8	1.8
Tx2	1.6	1.3	1.3
Tx3	0.5	1.6	1.6
Tx4	0.4	1.8	1.8
Tx5	1	1.5	1.5
Tx6	0.9	1.5	1.5
Tx7	0.6	1.9	1.9
Tx8	0.6	1.8	1.8
TxEx1	0.2	2	2
TxEx2	0.4	2.5	2.5
TxEx3	0.6	2	2
TxEx4	0.1	3.4	3.4
znf1	0.4	1	1
znf2	0.1	1.1	1.1
DNase1	0.2	1.2	1.2
BivProm1	0.1	2.6	2.6
BivProm2	0.2	2.5	2.5
BivProm3	0.3	1.6	1.6
BivProm4	0.1	2.9	2.9
PromF1	0.2	2.5	2.5
PromF2	0.1	3.1	3
PromF3	0.1	3	3
PromF4	0.2	3.1	3.1
PromF5	0.1	2.9	2.9
PromF6	0.1	2.3	2.3
PromF7	0.1	2.1	2.1
TSS1	0.2	2.4	2.4
TSS2	0.2	1.5	1.6
	100	0.3	0.3

Figure S44: Full-stack states enrichment values for fine-mapped variants at phenotype associated loci. At phenotype associated loci, causal variants were fine-mapped by two methods, CAVIAR and Finemap (Benner et al., 2016; Tate et al., 2019). A set of lead fine-mapped variants in 1MB loci across the genome were identified (**Methods**). The figure shows the full-stack states' enrichment values for these fine-mapped variants calculated against a background of common variants. The rows correspond to full-stack states. The first column gives the state labels, the second column the percent of the genome that each state covers, followed by columns with the fold enrichment for fine-mapped variants by CAVIAR and Finemap. The heatmap colors are on a column specific coloring scale. The last row shows the percentage of the background set of variants that the sets of lead fine-mapped variants occupy.

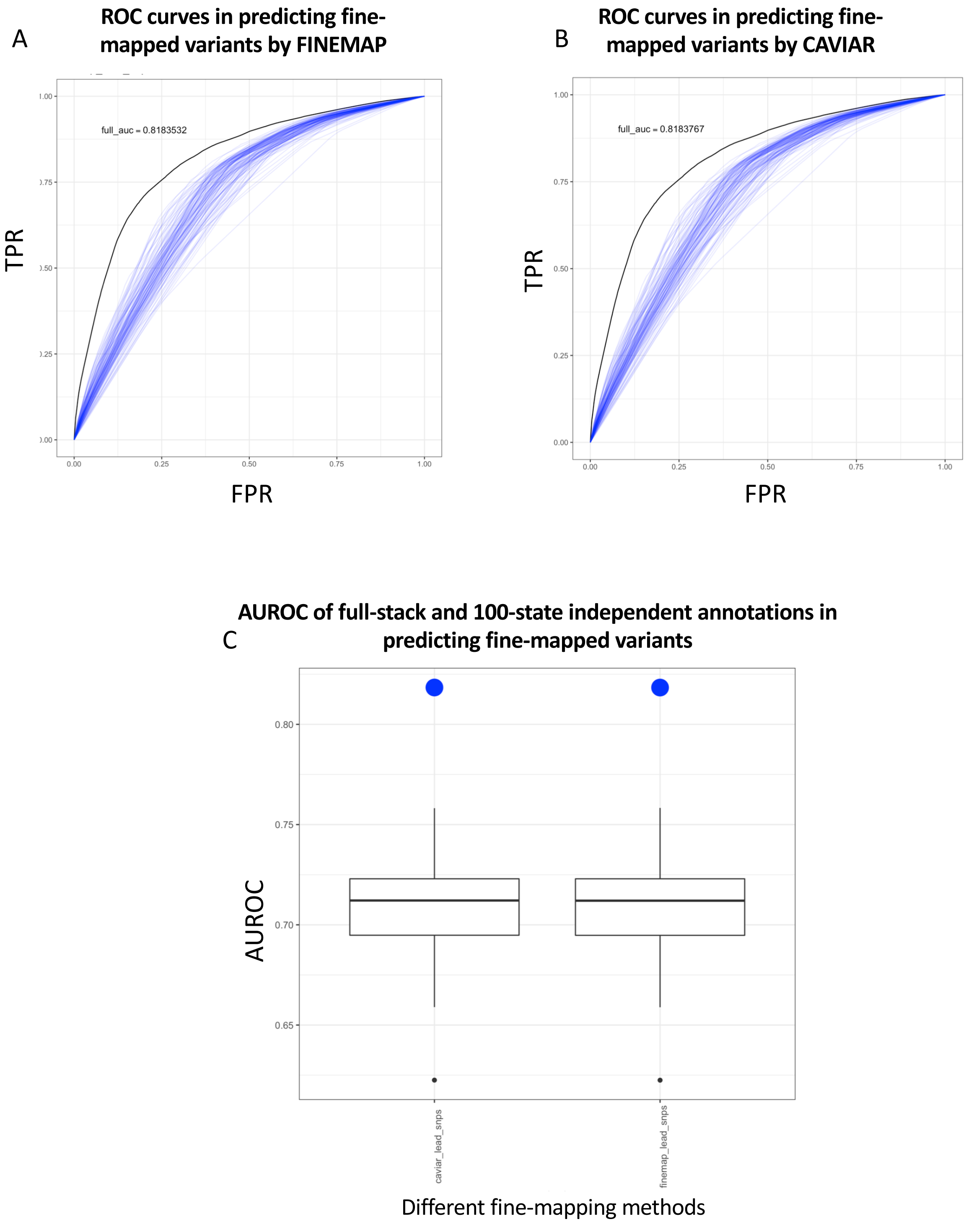

Figure S45: Comparison of full-stack model annotations and the 100-state annotations from independent models in predicting fine-mapped variants. (A) ROC curves for the full-stack model and the 127 100-state independent models' chromatin state annotations at predicting variants that show highest probabilities of being causal according to fine-mapping method FINEMAP (Benner et al., 2016) against the background of common variants (**Methods**). The full-stack annotation model's ROC curve is in black and the 127 100-state annotations from independent models' ROCs are shown in blue. **(B)** Similar plot as (A), but for variants evaluated by fine-mapping method CAVIAR (Chen et al., 2015). **(C)** Comparison of the AUROC in predicting fine-mapped variants from a background of common variants. The x-axis represents two different fine-mapping methods used to evaluate variants' potential for causing diseases. The box-plots show AUROC of 127 100 annotations from independent models in predicting these variants. The blue dots show the AUROC of the full-stack chromatin state annotations.

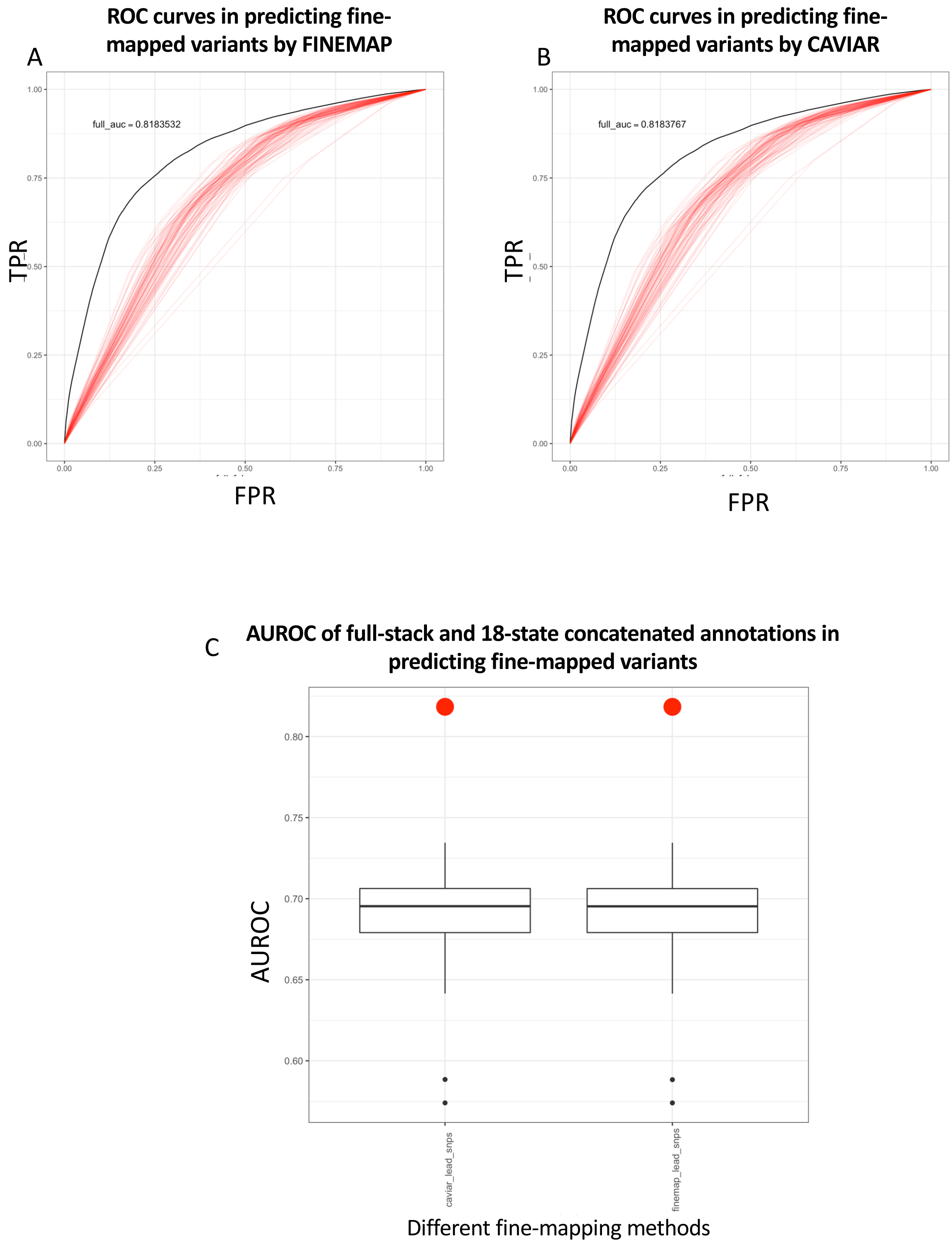

Figure S46: Comparison of full-stack model annotations and the 18-state annotations from a concatenated model in predicting fine-mapped variants. (A) ROC curves for the full-stack model and the 98 18-state annotations from concatenated models' chromatin state annotations at predicting variants that show highest probabilities of being causal according to fine-mapping method FINEMAP (Benner et al., 2016) against the background of common variants (**Methods**). The full-stack model annotation's ROC curve is in black and the 98 18-state annotations from a concatenated model ROCs are shown in red. **(B)** Similar plot as (A), but for variants evaluated by fine-mapping method CAVIAR (Chen et al., 2015). **(C)** Comparison of the AUROC in predicting fine-mapped variants from a background of common variants. The x-axis represents two different fine-mapping methods used to evaluate variants' potential for causing diseases. The box-plots show AUROC of 98 18-state annotations from concatenated models in predicting these variants. The red dots show the AUROC of the full-stack chromatin state annotations.

Enrichment of full-stack states with COSMIC database's cancer associated variants

state	% background	breast	haematopoietic lymphoid_tissu	liver	pancreas
GapArtf1	4.51	0.7	0.6	0.54	0.55
GapArtf2	0.05	0.76	4.88	2.07	4.09
GapArtf3	0.01	1.35	5.58	5.38	4.22
Quies1	10.8	1.21	1.69	1.54	1.57
Quies2	3.36	1.26	1.41	1.49	1.75
Quies3	13.4	0.97	1.23	0.95	0.94
Quies4	4.86	1.18	1.25	1.09	1.23
Quies5	1.85	1.34	0.96	0.79	0.98
HET1	0.77	1.25	1.38	1.53	1.95
HET2	0.75	1.29	0.92	1.2	1.68
HET3	1.49	1.11	0.89	1.01	1.06
HET4	0.61	0.87	1.14	0.89	1.47
HET5	0.27	1.04	0.97	1.23	1.32
HET6	0.63	1.37	1.29	1.31	1.89
HET7	1.12	1.19	1.17	1.03	1.37
HET8	0.47	1.15	0.85	0.95	0.98
HET9	1.08	1.44	1.34	1.18	1.56
ReprPC1	0.2	0.97	0.72	0.95	0.92
ReprPC2	0.35	1.06	0.76	0.89	0.84
ReprPC3	1.21	0.96	0.65	0.84	0.81
ReprPC4	4.3	1.02	0.88	0.92	0.9
ReprPC5	0.67	0.91	0.63	0.9	0.94
ReprPC6	1.63	0.92	0.77	0.88	0.94
ReprPC7	0.64	0.8	0.6	0.88	0.93
ReprPC8	0.52	1.24	0.75	0.64	0.77
ReprPC9	0.41	0.93	1.05	0.96	0.97
Acet1	0.2	0.83	2.67	1.23	1.37
Acet2	0.93	1	0.88	0.96	1.03
Acet3	2.9	1.04	0.98	0.93	1.01
Acet4	0.44	0.85	0.65	0.81	0.85
Acet5	0.94	0.95	0.92	0.93	0.93
Acet6	0.47	0.9	0.84	0.86	0.83
Acet7	0.31	0.77	0.65	0.77	0.77
Acet8	0.61	0.93	0.75	0.87	1
EnhWk1	1.69	1.03	0.99	1	0.93
EnhWk2	0.38	0.77	0.56	0.83	0.71
EnhWk3	0.91	0.86	0.77	0.86	0.83
EnhWk4	2.43	1.07	1.07	1.1	1.04
EnhWk5	1.09	1	0.93	0.96	0.9
EnhWk6	0.64	0.98	0.72	0.82	0.7
EnhWk7	0.53	1	0.88	0.83	0.79
EnhWk8	1.5	1.06	1.1	1.16	1.12
EnhA1	0.19	0.84	0.51	0.82	0.6
EnhA2	0.36	1.07	0.72	0.87	0.77
EnhA3	0.21	1.18	0.52	0.82	0.61
EnhA4	0.33	1	0.76	0.87	0.69
EnhA5	0.78	1.02	0.85	0.92	0.82
EnhA6	0.61	1	0.75	0.9	0.91
EnhA7	0.42	0.84	0.78	0.83	0.8
EnhA8	0.27	0.85	0.84	0.79	0.69
EnhA9	0.18	1.02	0.49	0.75	0.63
EnhA10	0.43	0.86	0.64	0.76	0.64
EnhA11	0.78	0.93	0.79	0.82	0.79
EnhA12	0.36	0.89	0.65	0.9	0.77
EnhA13	0.84	0.98	0.95	0.96	0.85
EnhA14	0.4	0.91	0.65	0.8	0.61
EnhA15	1.11	0.91	0.91	0.87	0.74
EnhA16	0.7	1.06	0.94	0.92	0.88
EnhA17	0.58	0.95	0.81	0.95	0.83
EnhA18	0.5	1.04	0.88	1.02	1.1
EnhA19	0.28	0.93	0.65	0.8	0.77
EnhA20	0.38	0.93	1.03	1.03	0.88
TxEnh1	0.43	0.83	0.62	0.78	0.59
TxEnh2	0.43	0.73	0.43	0.73	0.55
TxEnh3	0.27	0.76	0.46	0.73	0.6
TxEnh4	0.25	0.68	0.37	0.96	0.63
TxEnh5	0.49	0.82	0.48	0.83	0.67
TxEnh6	0.19	0.74	0.38	0.8	0.6
TxEnh7	0.29	0.69	0.48	0.76	0.59
TxEnh8	0.25	0.83	0.41	0.84	0.59
TxWk1	3.03	0.84	0.76	0.82	0.68
TxWk2	0.85	0.85	0.6	0.85	0.8
Tx1	0.9	0.76	0.62	0.7	0.55
Tx2	1.73	0.82	0.85	0.77	0.68
Tx3	0.56	0.75	0.62	0.73	0.67
Tx4	0.5	0.77	0.44	0.78	0.54
Tx5	1	0.76	0.58	0.72	0.66
Tx6	1.17	0.75	0.46	0.91	0.54
Tx7	0.83	0.71	0.37	1	0.49
Tx8	0.73	0.81	0.48	0.85	0.58
TxEx1	0.26	0.7	0.33	0.87	0.54
TxEx2	0.5	0.66	0.35	0.95	0.57
TxEx3	0.65	0.75	0.44	0.82	0.62
TxEx4	0.09	0.94	0.33	0.95	0.56
znf1	0.44	0.85	0.53	0.73	0.7
znf2	0.15	0.77	0.42	0.83	0.72
DNase1	0.22	1	0.72	1.09	0.78
BivProm1	0.14	0.78	0.63	1.01	0.8
BivProm2	0.16	0.79	0.68	1.06	1.05
BivProm3	0.3	0.87	0.72	0.91	0.83
BivProm4	0.14	0.92	0.8	0.89	0.78
PromF1	0.22	0.91	0.48	0.82	0.66
PromF2	0.15	0.89	0.53	0.81	0.55
PromF3	0.16	0.77	0.51	0.83	0.58
PromF4	0.18	0.79	0.63	0.97	0.58
PromF5	0.14	0.83	0.62	0.94	0.67
PromF6	0.14	0.75	0.46	0.83	0.51
PromF7	0.17	0.69	0.54	0.78	0.57
TSS1	0.12	0.95	0.57	0.94	0.66
TSS2	0.12	0.81	0.81	0.88	0.68
	100	0.01	0.02	0.05	0.02

Figure S47: Full-stack states enrichments with cancer-associated somatic mutations in the non-coding genome. Each row corresponds to a full-stack state. The first column gives the state labels and the second column shows the percentage in the background genome context that each full-stack state occupies. For this analysis, the background context is the non-coding genome (**Methods**). The following columns correspond to one of four cancer types with the most number of mutations in the COSMIC database (Tate et al., 2019). These columns give the enrichments of full-stack states for mutations that appear at least once in the database for the cancer types. The heatmap colors are on a column specific coloring scale. The last row shows the percentage of the genome that mutations associated with each cancer type occupy.

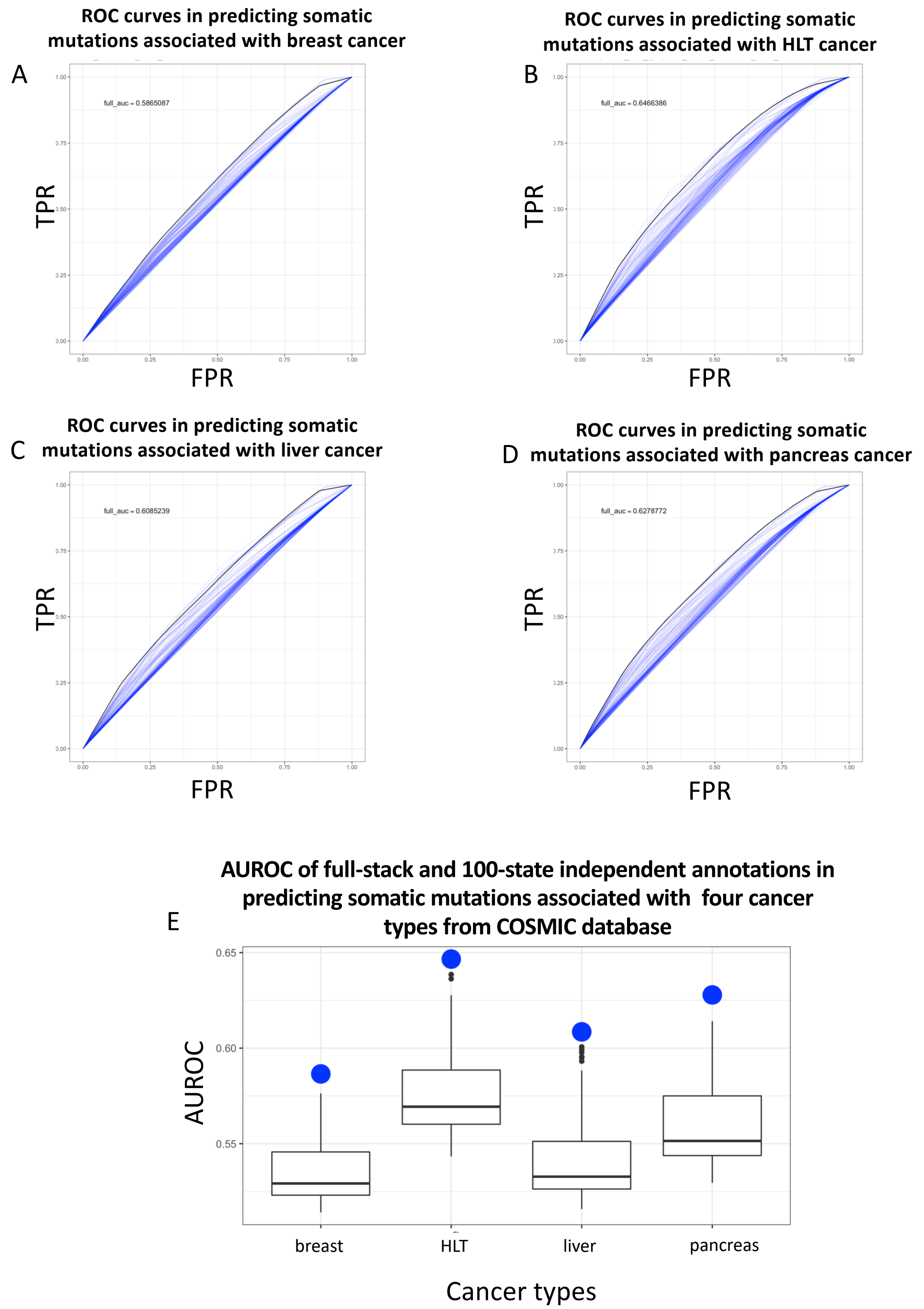

Figure S48: Comparison of full-stack model annotation and the 100-state independent annotations in predicting somatic mutations associated with four cancer types from COSMIC database (Tate et al., 2019). **(A)** ROC curves for the full-stack model's annotations and the 127 100-state annotations from independent models at predicting somatic mutations associated with breast cancer against the background of non-coding genome (**Methods**). The full-stack model annotation's ROC curve is in black and the 127 100-state independent model annotations' ROCs are shown in blue. **(B-D)** Similar plot as (A), but for mutations associated with **(B)** haematopoietic and lymphoid tissue (HLT) cancer, **(C)** liver cancer and **(D)** pancreas cancer, respectively. **(E)** Comparison of the AUROC in predicting cancer-associated somatic mutations from a background of non-coding genome. The x-axis represents four different cancer types that we considered in this analysis. The box-plots show AUROC of 127 100-state independent models' in predicting these mutations. The blue dots show the AUROC of the full-stack chromatin state annotations.

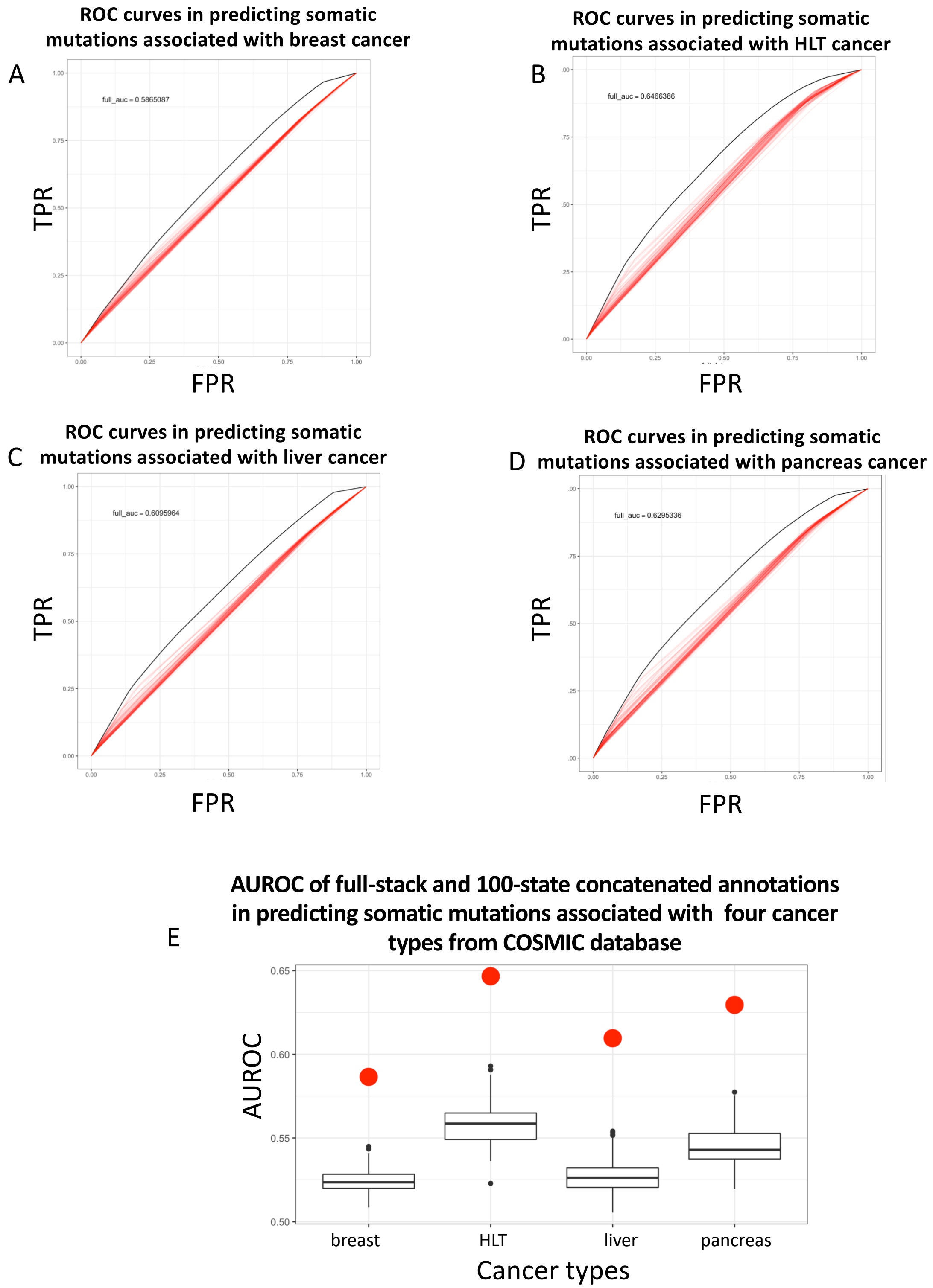

Figure S49: Comparison of full-stack model annotation and the 18-state concatenated annotations in predicting somatic mutations associated with four cancer types from COSMIC database (Tate et al., 2019). (A) ROC curves for the full-stack model and the 98 18-state concatenatedmodels' chromatin state annotations at predicting somatic mutations associated with breast cancer against the background of non-coding genome (**Methods**). The full-stack model's ROC curve is in black and the 98 18-state annotations from a concatenated model ROCs are shown in blue. **(B-D)** Similar plot as (A), but for mutations associated with haematopoietic and lymphoid tissue (HLT) cancer, liver cancer and pancreas cancer, respectively. **(C)** Comparison of the AUROC in predicting cancer-associated somatic mutations from a background of non-coding genome. The x-axis represents four different cancer types that we considered in this analysis. The box-plots show AUROC of 98 18-state annotations from a concatenated model in predicting these mutations. The blue dots show the AUROC of the full-stack chromatin state annotations.

state	percent_in_genome	unmapped_hg19_to_hg38	percent_unmapped_in_state
GapArtf1	11.86	8.40	99.59
GapArtf2	0.05	0.14	0.01
GapArtf3	0.01	0.35	0.00
Quies1	9.88	0.00	0.02
Quies2	3.07	0.00	0.00
Quies3	12.23	0.01	0.09
Quies4	4.45	0.01	0.05
Quies5	1.69	0.01	0.02
HET1	0.71	0.00	0.00
HET2	0.69	0.01	0.00
HET3	1.36	0.00	0.00
HET4	0.56	0.01	0.01
HET5	0.25	0.01	0.00
HET6	0.58	0.01	0.01
HET7	1.02	0.01	0.01
HET8	0.43	0.01	0.00
HET9	1.00	0.02	0.02
ReprPC1	0.19	0.01	0.00
ReprPC2	0.32	0.01	0.00
ReprPC3	1.11	0.00	0.00
ReprPC4	3.93	0.00	0.01
ReprPC5	0.63	0.00	0.00
ReprPC6	1.51	0.00	0.01
ReprPC7	0.61	0.00	0.00
ReprPC8	0.48	0.01	0.00
ReprPC9	0.37	0.01	0.00
Acet1	0.18	0.02	0.00
Acet2	0.85	0.00	0.00
Acet3	2.65	0.00	0.01
Acet4	0.40	0.00	0.00
Acet5	0.86	0.00	0.00
Acet6	0.43	0.00	0.00
Acet7	0.28	0.00	0.00
Acet8	0.56	0.00	0.00
EnhWk1	1.54	0.00	0.00
EnhWk2	0.35	0.00	0.00
EnhWk3	0.83	0.00	0.00
EnhWk4	2.22	0.00	0.00
EnhWk5	0.99	0.01	0.01
EnhWk6	0.59	0.01	0.01
EnhWk7	0.48	0.00	0.00
EnhWk8	1.37	0.00	0.00
EnhA1	0.18	0.00	0.00
EnhA2	0.33	0.00	0.00
EnhA3	0.19	0.00	0.00
EnhA4	0.30	0.00	0.00
EnhA5	0.71	0.00	0.00
EnhA6	0.56	0.00	0.00
EnhA7	0.39	0.00	0.00
EnhA8	0.25	0.01	0.00
EnhA9	0.16	0.00	0.00
EnhA10	0.39	0.00	0.00
EnhA11	0.72	0.01	0.00
EnhA12	0.33	0.00	0.00
EnhA13	0.76	0.00	0.00
EnhA14	0.37	0.00	0.00
EnhA15	1.02	0.00	0.00
EnhA16	0.65	0.01	0.01
EnhA17	0.53	0.00	0.00
EnhA18	0.46	0.00	0.00
EnhA19	0.26	0.00	0.00
EnhA20	0.35	0.00	0.00
TxEnh1	0.39	0.00	0.00
TxEnh2	0.39	0.00	0.00
TxEnh3	0.25	0.00	0.00
TxEnh4	0.27	0.00	0.00
TxEnh5	0.50	0.00	0.00
TxEnh6	0.19	0.00	0.00
TxEnh7	0.27	0.00	0.00
TxEnh8	0.24	0.00	0.00
TxWk1	2.80	0.00	0.01
TxWk2	0.84	0.00	0.00
Tx1	0.82	0.00	0.00
Tx2	1.58	0.00	0.00
Tx3	0.51	0.00	0.00
Tx4	0.47	0.00	0.00
Tx5	0.94	0.00	0.00
Tx6	1.11	0.00	0.00
Tx7	0.82	0.00	0.00
Tx8	0.68	0.00	0.00
TxEx1	0.27	0.00	0.00
TxEx2	0.56	0.00	0.00
TxEx3	0.66	0.00	0.00
TxEx4	0.10	0.00	0.00
znf1	0.41	0.00	0.00
znf2	0.15	0.00	0.00
DNase1	0.20	0.01	0.00
BivProm1	0.15	0.01	0.00
BivProm2	0.16	0.01	0.00
BivProm3	0.29	0.01	0.00
BivProm4	0.13	0.01	0.00
PromF1	0.20	0.00	0.00
PromF2	0.14	0.00	0.00
PromF3	0.15	0.00	0.00
PromF4	0.19	0.00	0.00
PromF5	0.14	0.00	0.00
PromF6	0.13	0.00	0.00
PromF7	0.16	0.00	0.00
TSS1	0.12	0.01	0.00
TSS2	0.11	0.03	0.00
**percent in genome**	100	7.665	100

Enrichment of full-stack states with bases in hg19 that were unmapped to hg38, and analysis of unmapped bases with assembly gaps (all analyses in chr1-22,X,Y)

B

# bp in hg38 not mapped to a state	218,892,096
# bp in hg38 not mapped to a state AND overlapping hg38 assembly gaps	134,483,364
Fraction of hg38 bases that are unannotated to a state after liftOver from hg19 AND overlap hg38 assembly gaps	0.61
# bp in hg19 unmapped to hg38	237,275,800
# bp in hg19 unmapped to hg38 AND overlapping assembly gaps	234,342,292
Fraction of unmapped_to_hg38 bp in hg19 that also overlap hg19 assembly gaps	0.99
# bp in hg38 that are not assembly gaps	2,937,659,104
# bp in hg38 that are not assembly gaps AND annotated as a state after liftOver	2,853,200,372
Fraction of hg38 bases that are non-assembly gaps AND annotated to a state after liftOver	0.97

Figure S50: Full-stack states enrichments of bases that were not lifted over from hg19 to hg38. (A) The heatmap shows enrichment values for the full-stack states (rows) of genomic bases that were unmapped when lifting the state annotation from hg19 to hg38 (Methods). The first column shows the state label and the second column shows the percentage of the genome that each state covers. The third column shows enrichment values, colored such that highest enrichment values are colored red and lowest ones are colored white. The fourth column shows the percentage of the unmapped regions (from hg19 to hg38) in each state. **(B)** Table showing details of numbers of bases involved in liftOver procedure, highlighting the overlap between the unmapped and unannotated regions with assembly gaps. As part of the liftOver procedure, bases in hg38 that are mapped to from multiple bases in hg19 are left unannotated to any state in hg38 (Methods). All results are reported in chromosomes 1-22, X, Y.

References

Abel, H. J., Larson, D. E., Regier, A. A., Chiang, C., Das, I., Kanchi, K. L., Layer, R. M., Neale, B. M., Salerno, W. J., & Reeves, C. (2020). Mapping and characterization of structural variation in 17,795 human genomes. *Nature*, *583*(7814), 83–89.

Arneson, A., & Ernst, J. (2019). Systematic discovery of conservation states for single-nucleotide annotation of the human genome. *Communications Biology*, *2*(1), 1–14.

Benner, C., Spencer, C. C., Havulinna, A. S., Salomaa, V., Ripatti, S., & Pirinen, M. (2016). FINEMAP: Efficient variable selection using summary data from genome-wide association studies. *Bioinformatics*, *32*(10), 1493–1501.

Chen, W., Larrabee, B. R., Ovsyannikova, I. G., Kennedy, R. B., Haralambieva, I. H., Poland, G. A., & Schaid, D. J. (2015). Fine mapping causal variants with an approximate Bayesian method using marginal test statistics. *Genetics*, *200*(3), 719–736.

Ernst, J., & Kellis, M. (2015). Large-scale imputation of epigenomic datasets for systematic annotation of diverse human tissues. *Nature Biotechnology*, *33*(4), 364–376.

Hoffman, M. M., Ernst, J., Wilder, S. P., Kundaje, A., Harris, R. S., Libbrecht, M., Giardine, B., Ellenbogen, P. M., Bilmes, J. A., & Birney, E. (2013). Integrative annotation of chromatin elements from ENCODE data. *Nucleic Acids Research*, *41*(2), 827–841.

Jones, P. A., & Takai, D. (2001). The role of DNA methylation in mammalian epigenetics. *Science*, *293*(5532), 1068–1070.

Karczewski, K. J., Francioli, L. C., Tiao, G., Cummings, B. B., Alföldi, J., Wang, Q., Collins, R. L., Laricchia, K. M., Ganna, A., & Birnbaum, D. P. (2020). The mutational constraint spectrum quantified from variation in 141,456 humans. *Nature*, *581*(7809), 434–443.

Kundaje, A., Meuleman, W., Ernst, J., Bilenky, M., Yen, A., Heravi-Moussavi, A., Kheradpour, P., Zhang, Z., Wang, J., & Ziller, M. J. (2015). Integrative analysis of 111 reference human epigenomes. *Nature*, *518*(7539), 317–330.

Lister, R., Pelizzola, M., Dowen, R. H., Hawkins, R. D., Hon, G., Tonti-Filippini, J., Nery, J. R., Lee, L., Ye, Z., & Ngo, Q.-M. (2009). Human DNA methylomes at base resolution show widespread epigenomic differences. *Nature*, *462*(7271), 315–322.

McLean, C. Y., Bristor, D., Hiller, M., Clarke, S. L., Schaar, B. T., Lowe, C. B., Wenger, A. M., & Bejerano, G. (2010). GREAT improves functional interpretation of cis-regulatory regions. *Nature Biotechnology*, *28*(5), 495–501.

Tate, J. G., Bamford, S., Jubb, H. C., Sondka, Z., Beare, D. M., Bindal, N., Boutselakis, H., Cole, C. G., Creatore, C., & Dawson, E. (2019). COSMIC: the catalogue of somatic mutations in cancer. *Nucleic Acids Research*, *47*(D1), D941–D947.

Weber, M., Hellmann, I., Stadler, M. B., Ramos, L., Pääbo, S., Rebhan, M., & Schübeler, D. (2007). Distribution, silencing potential and evolutionary impact of promoter DNA methylation in the human genome. *Nature Genetics*, *39*(4), 457–466.

Welter, D., MacArthur, J., Morales, J., Burdett, T., Hall, P., Junkins, H., Klemm, A., Flicek, P., Manolio, T., & Hindorff, L. (2014). The NHGRI GWAS Catalog, a curated resource of SNP-trait associations. *Nucleic Acids Research*, *42*(D1), D1001–D1006.
