# Supplementary material for: Universal annotation of the human genome through integration of over a thousand epigenomic datasets
Source: Genome Biol. 2022 Jan 6;23:9. doi: 10.1186/s13059-021-02572-z (PMC8734071; doi:10.1186/s13059-021-02572-z)
Supplement: Supplementary file 2 — Additional file 2. GO terms associated with each full-stack state. Each figure shows the 5 GO Biological Process and 5 GO Molecular Function terms most significantly enriched in each full-stack state. State Quies3 did not have a list of enriched GO terms because there was no output from GREAT for regions associated with state. Therefore, there are 99 figures corresponding to 99 full-stack states with output from GREAT. [file 13059_2021_2572_MOESM2_ESM.pdf]

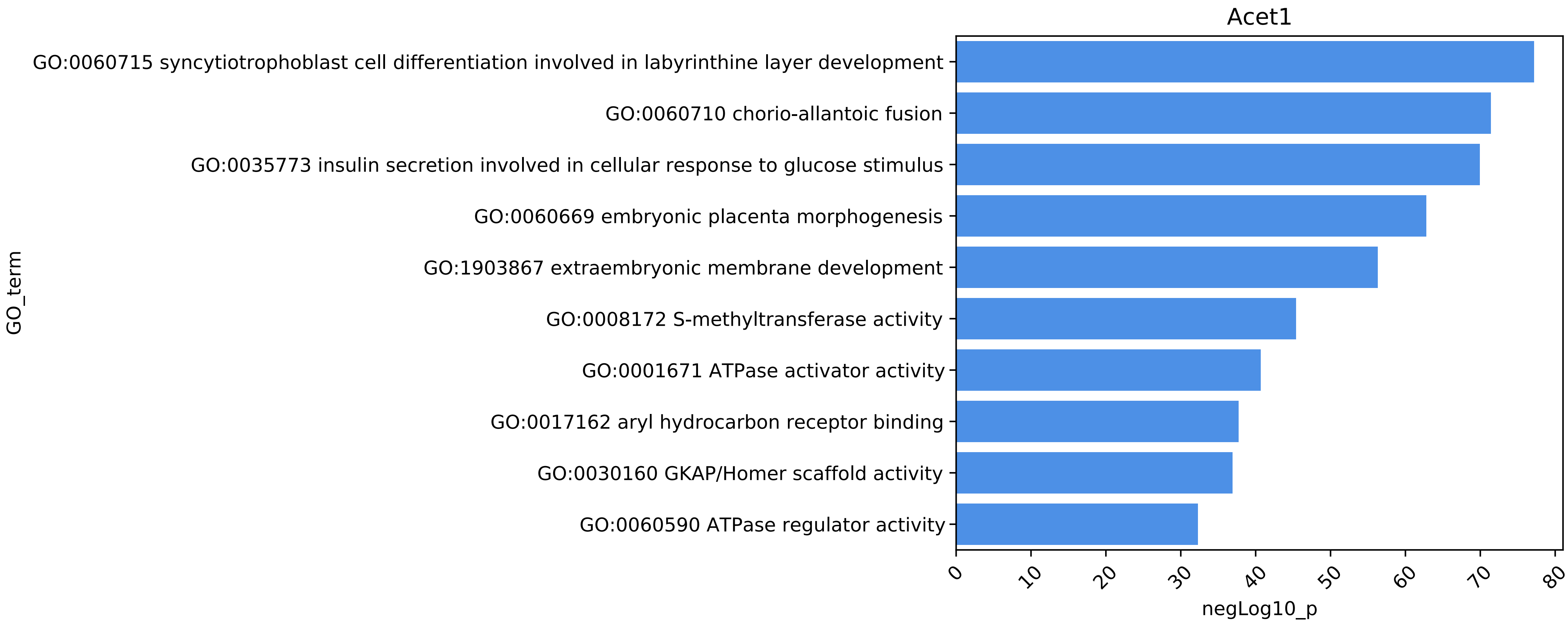

Acet1
GO:0060715 syncytiotrophoblast cell differentiation involved in labyrinthine layer development
GO:0060710 chorio-allantoic fusion
GO:0035773 insulin secretion involved in cellular response to glucose stimulus
GO:0060669 embryonic placenta morphogenesis
GO:1903867 extraembryonic membrane development
GO:0008172 S-methyltransferase activity
GO:0001671 ATPase activator activity
GO:0017162 aryl hydrocarbon receptor binding
GO:0030160 GKAP/Homer scaffold activity
GO:0060590 ATPase regulator activity
GO_term
0
10
20
30
40
50
60
70
80
negLog10_p

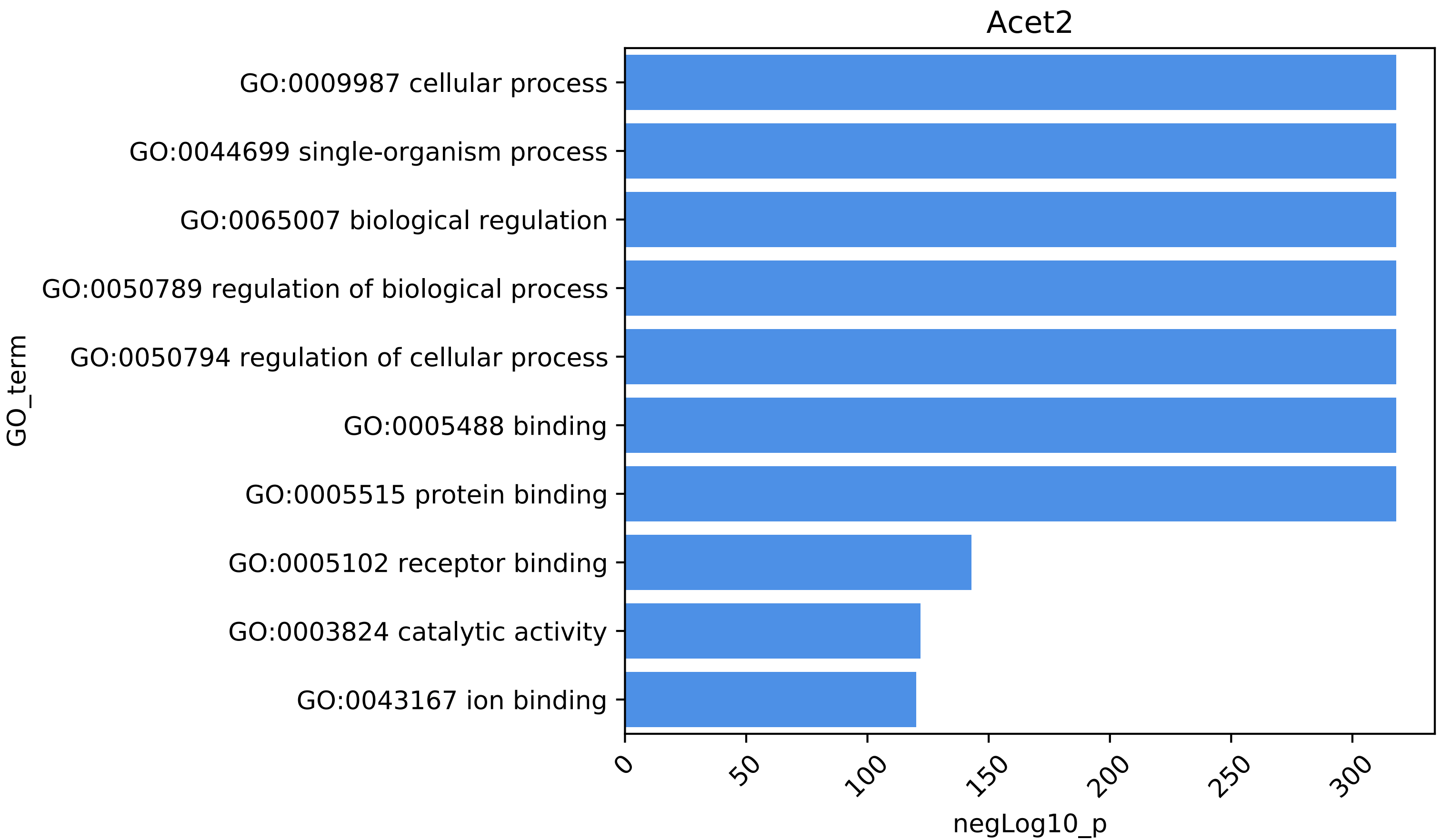

Acet2
GO:0009987 cellular process
GO:0044699 single-organism process
GO:0065007 biological regulation
GO:0050789 regulation of biological process
GO:0050794 regulation of cellular process
GO:0005488 binding
GO:0005515 protein binding
GO:0005102 receptor binding
GO:0003824 catalytic activity
GO:0043167 ion binding
GO_term
0
50
100
150
200
250
300
negLog10_p

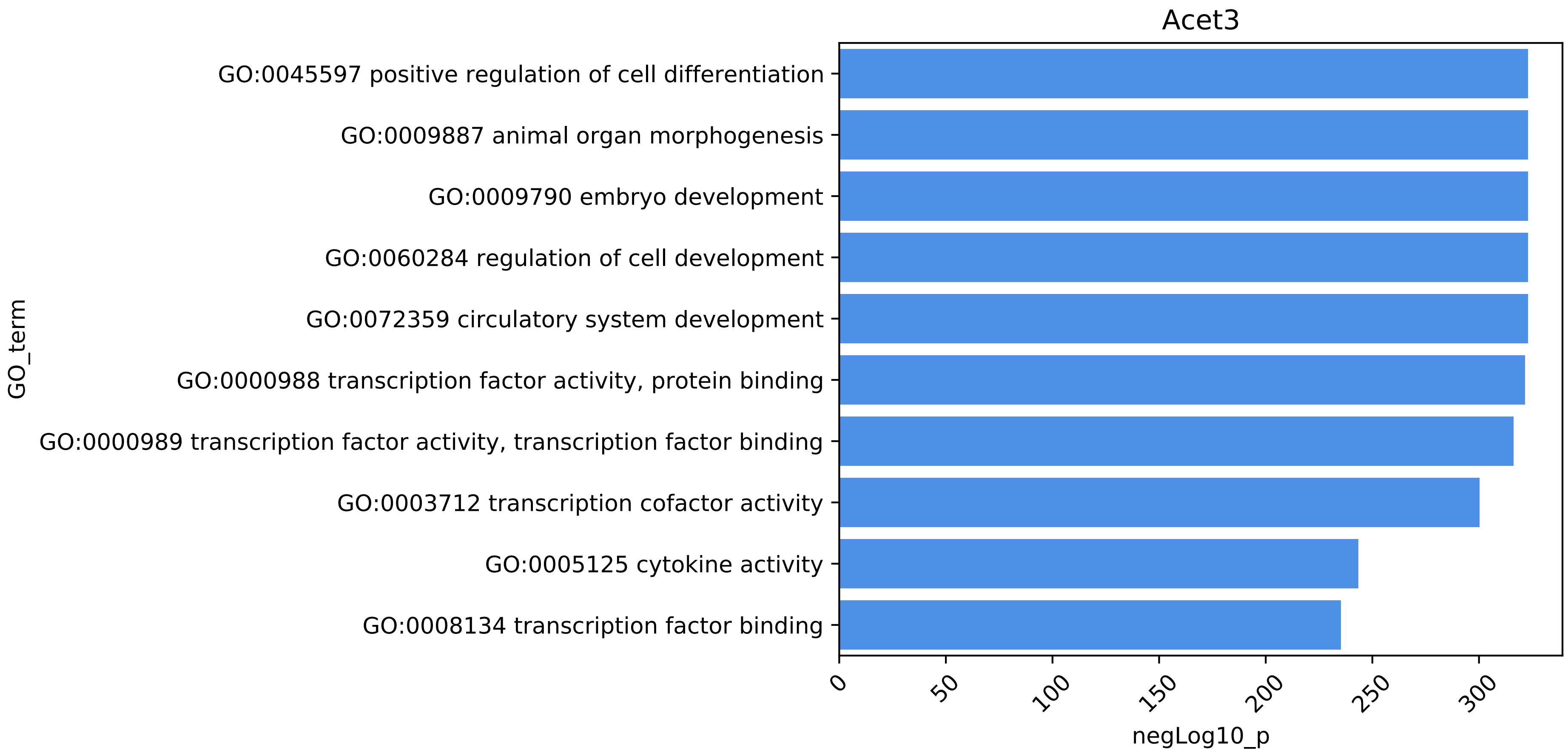
Acet3
GO_term
GO:0045597 positive regulation of cell differentiation
GO:0009887 animal organ morphogenesis
GO:0009790 embryo development
GO:0060284 regulation of cell development
GO:0072359 circulatory system development
GO:0000988 transcription factor activity, protein binding
GO:0000989 transcription factor activity, transcription factor binding
GO:0003712 transcription cofactor activity
GO:0005125 cytokine activity
GO:0008134 transcription factor binding
0
50
100
150
200
250
300
negLog10_p

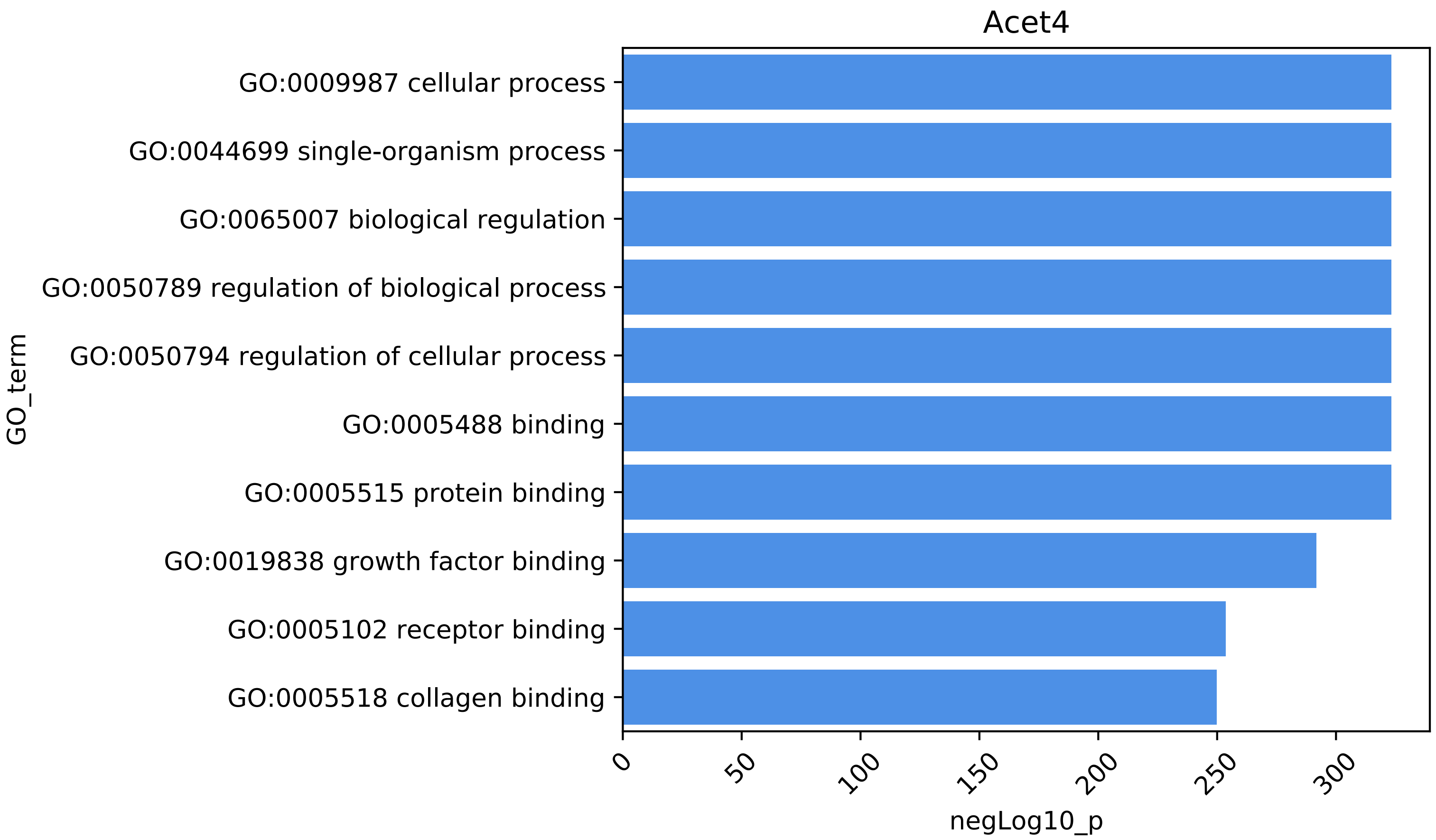
Acet4
GO:0009987 cellular process
GO:0044699 single-organism process
GO:0065007 biological regulation
GO:0050789 regulation of biological process
GO:0050794 regulation of cellular process
GO:0005488 binding
GO:0005515 protein binding
GO:0019838 growth factor binding
GO:0005102 receptor binding
GO:0005518 collagen binding
GO_term
0
50
100
150
200
250
300
negLog10_p

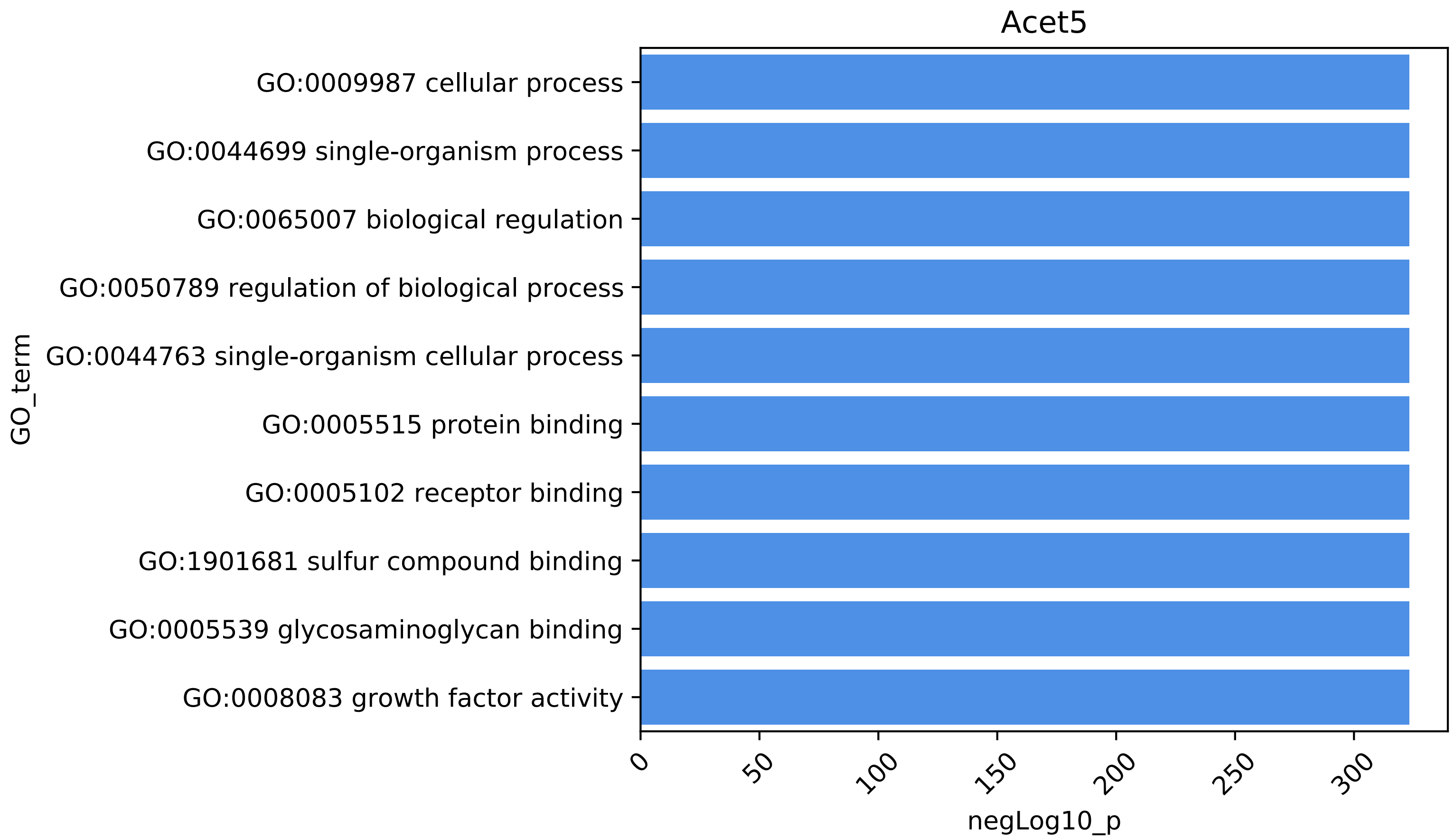

Acet5
GO_term
GO:0009987 cellular process
GO:0044699 single-organism process
GO:0065007 biological regulation
GO:0050789 regulation of biological process
GO:0044763 single-organism cellular process
GO:0005515 protein binding
GO:0005102 receptor binding
GO:1901681 sulfur compound binding
GO:0005539 glycosaminoglycan binding
GO:0008083 growth factor activity
0
50
100
150
200
250
300
negLog10_p

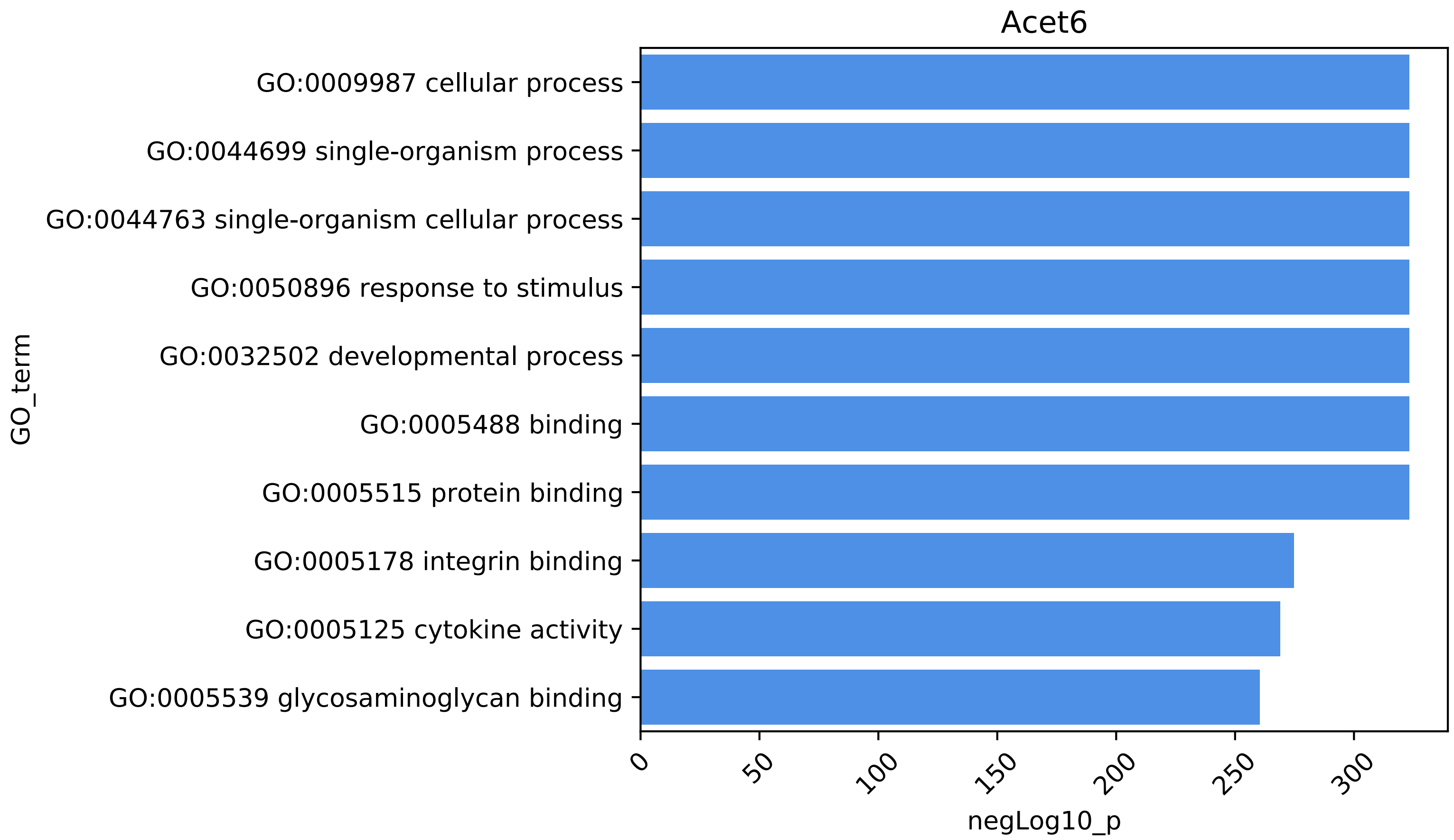
Acet6
GO:0009987 cellular process
GO:0044699 single-organism process
GO:0044763 single-organism cellular process
GO:0050896 response to stimulus
GO:0032502 developmental process
GO:0005488 binding
GO:0005515 protein binding
GO:0005178 integrin binding
GO:0005125 cytokine activity
GO:0005539 glycosaminoglycan binding
GO_term
0
50
100
150
200
250
300
negLog10_p

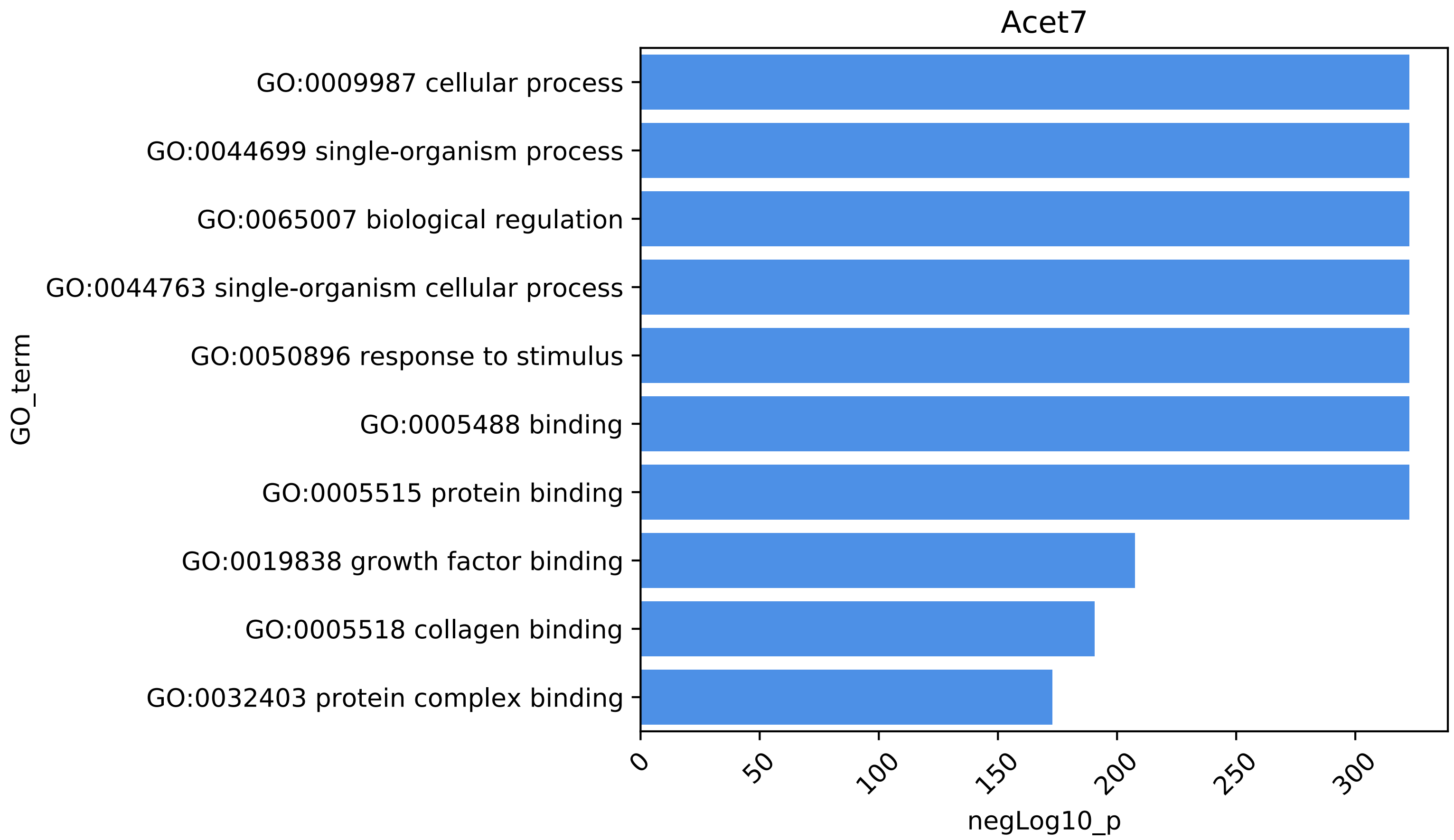

Acet7
GO:0009987 cellular process
GO:0044699 single-organism process
GO:0065007 biological regulation
GO:0044763 single-organism cellular process
GO:0050896 response to stimulus
GO:0005488 binding
GO:0005515 protein binding
GO:0019838 growth factor binding
GO:0005518 collagen binding
GO:0032403 protein complex binding
GO_term
0
50
100
150
200
250
300
negLog10_p

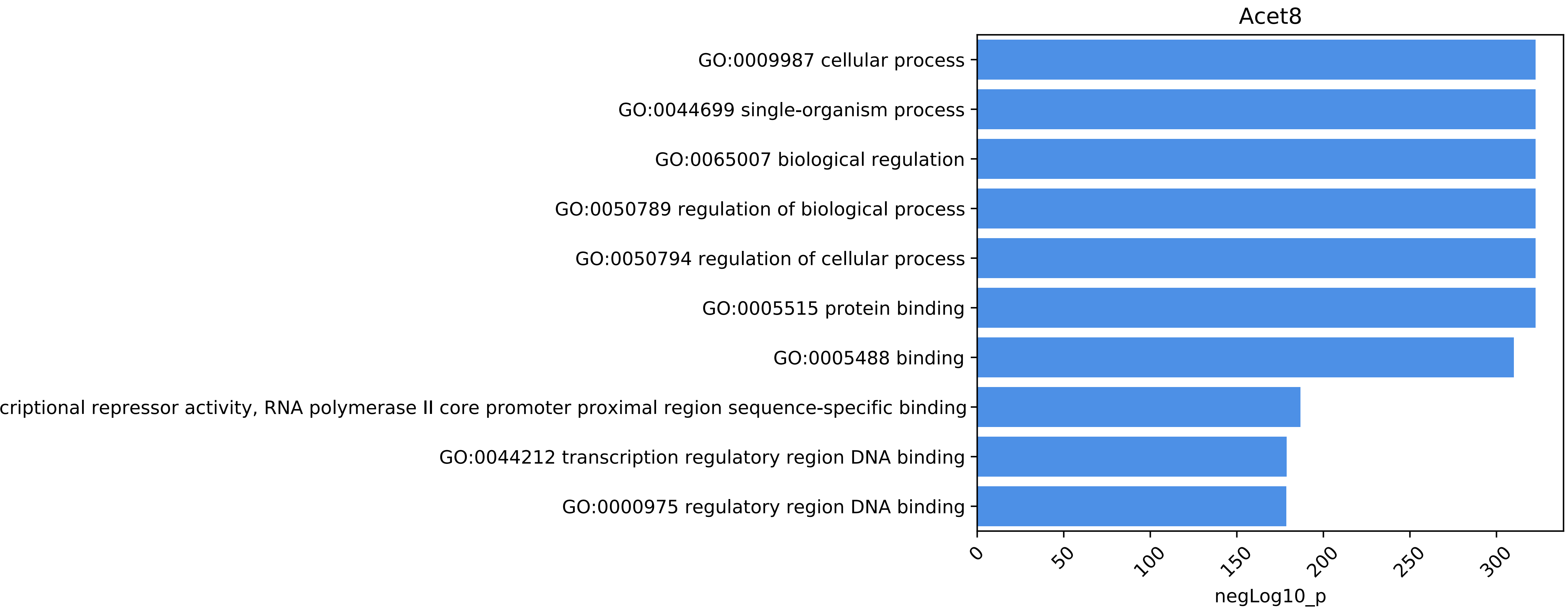
Acet8
GO:0009987 cellular process
GO:0044699 single-organism process
GO:0065007 biological regulation
GO:0050789 regulation of biological process
GO:0050794 regulation of cellular process
GO:0005515 protein binding
GO:0005488 binding
criptional repressor activity, RNA polymerase II core promoter proximal region sequence-specific binding
GO:0044212 transcription regulatory region DNA binding
GO:0000975 regulatory region DNA binding
0
50
100
150
200
250
300
negLog10_p

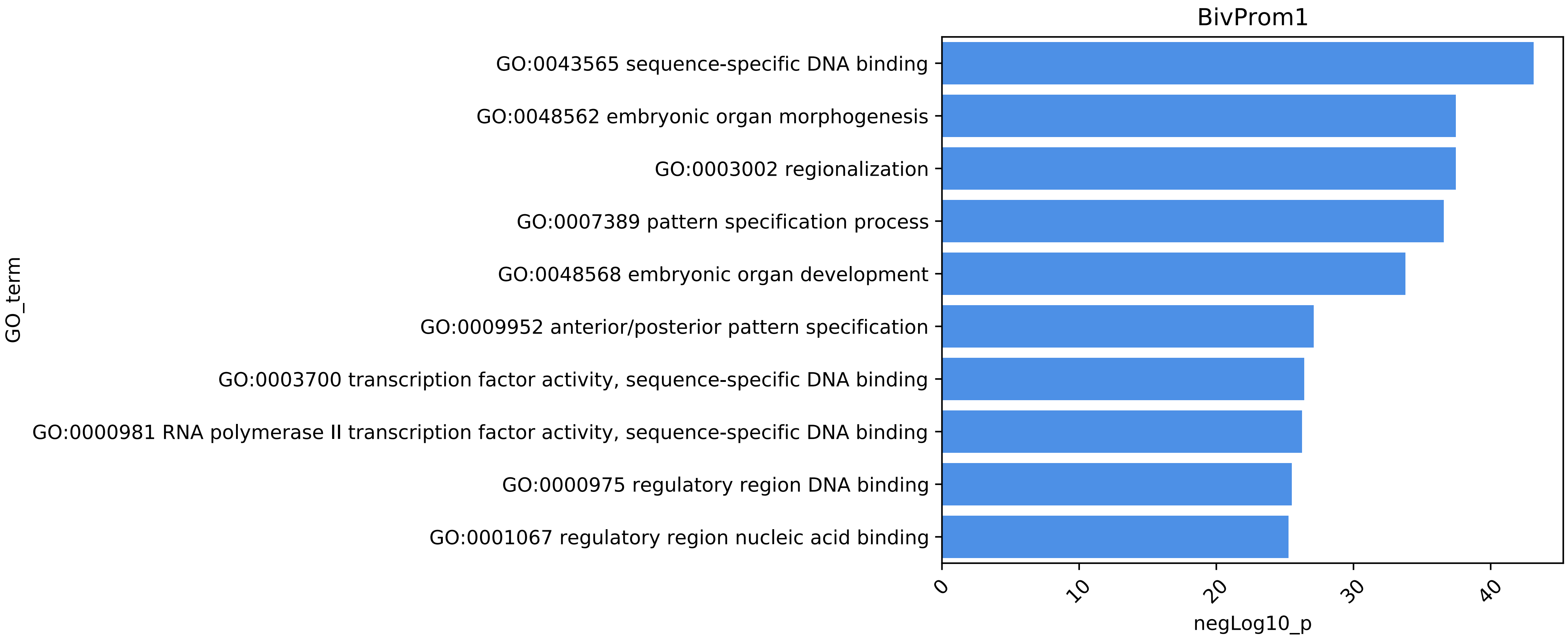
BivProm1
GO:0043565 sequence-specific DNA binding
GO:0048562 embryonic organ morphogenesis
GO:0003002 regionalization
GO:0007389 pattern specification process
GO:0048568 embryonic organ development
GO:0009952 anterior/posterior pattern specification
GO:0003700 transcription factor activity, sequence-specific DNA binding
GO:0000981 RNA polymerase II transcription factor activity, sequence-specific DNA binding
GO:0000975 regulatory region DNA binding
GO:0001067 regulatory region nucleic acid binding
GO_term
0
10
20
30
40
negLog10_p

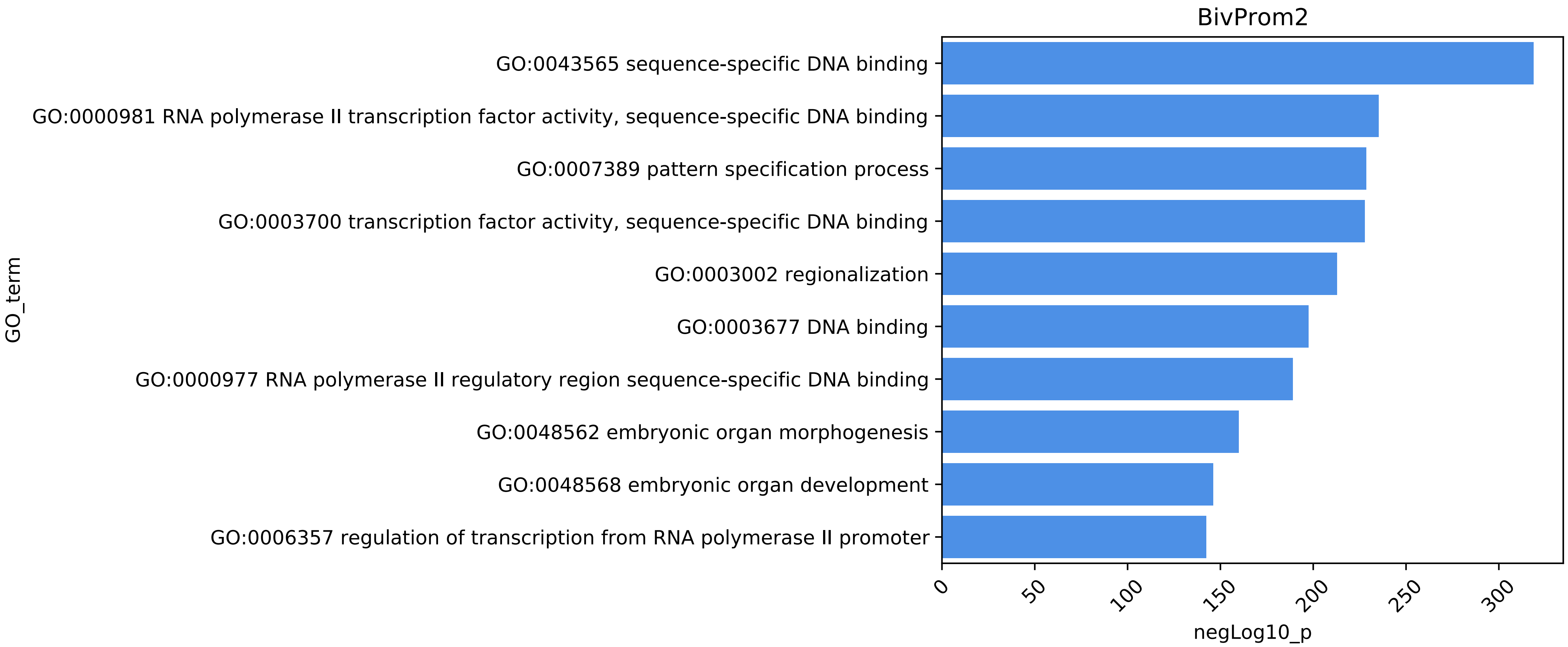
BivProm2
GO:0043565 sequence-specific DNA binding
GO:0000981 RNA polymerase II transcription factor activity, sequence-specific DNA binding
GO:0007389 pattern specification process
GO:0003700 transcription factor activity, sequence-specific DNA binding
GO:0003002 regionalization
GO:0003677 DNA binding
GO:0000977 RNA polymerase II regulatory region sequence-specific DNA binding
GO:0048562 embryonic organ morphogenesis
GO:0048568 embryonic organ development
GO:0006357 regulation of transcription from RNA polymerase II promoter
GO_term
0
50
100
150
200
250
300
negLog10_p

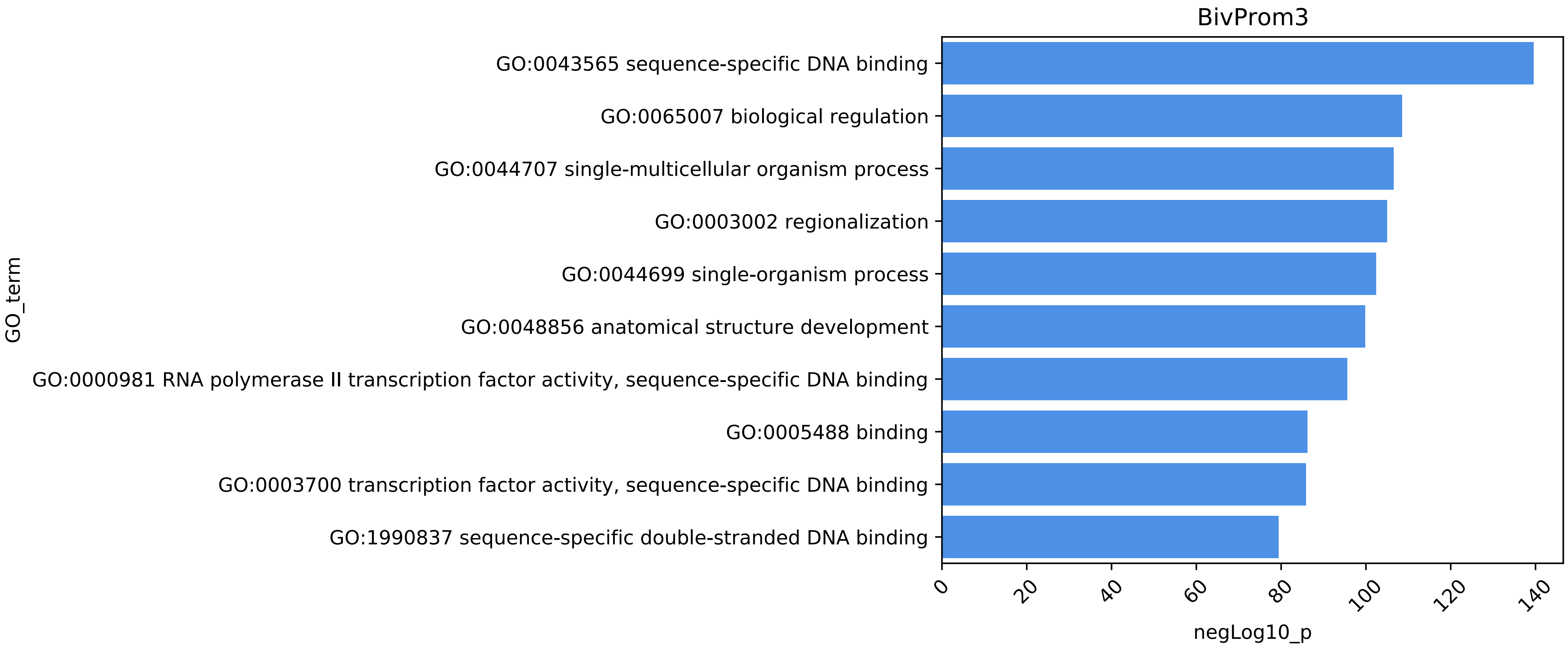

BivProm3
GO_term
GO:0043565 sequence-specific DNA binding
GO:0065007 biological regulation
GO:0044707 single-multicellular organism process
GO:0003002 regionalization
GO:0044699 single-organism process
GO:0048856 anatomical structure development
GO:0000981 RNA polymerase II transcription factor activity, sequence-specific DNA binding
GO:0005488 binding
GO:0003700 transcription factor activity, sequence-specific DNA binding
GO:1990837 sequence-specific double-stranded DNA binding
0
20
40
60
80
100
120
140
negLog10_p

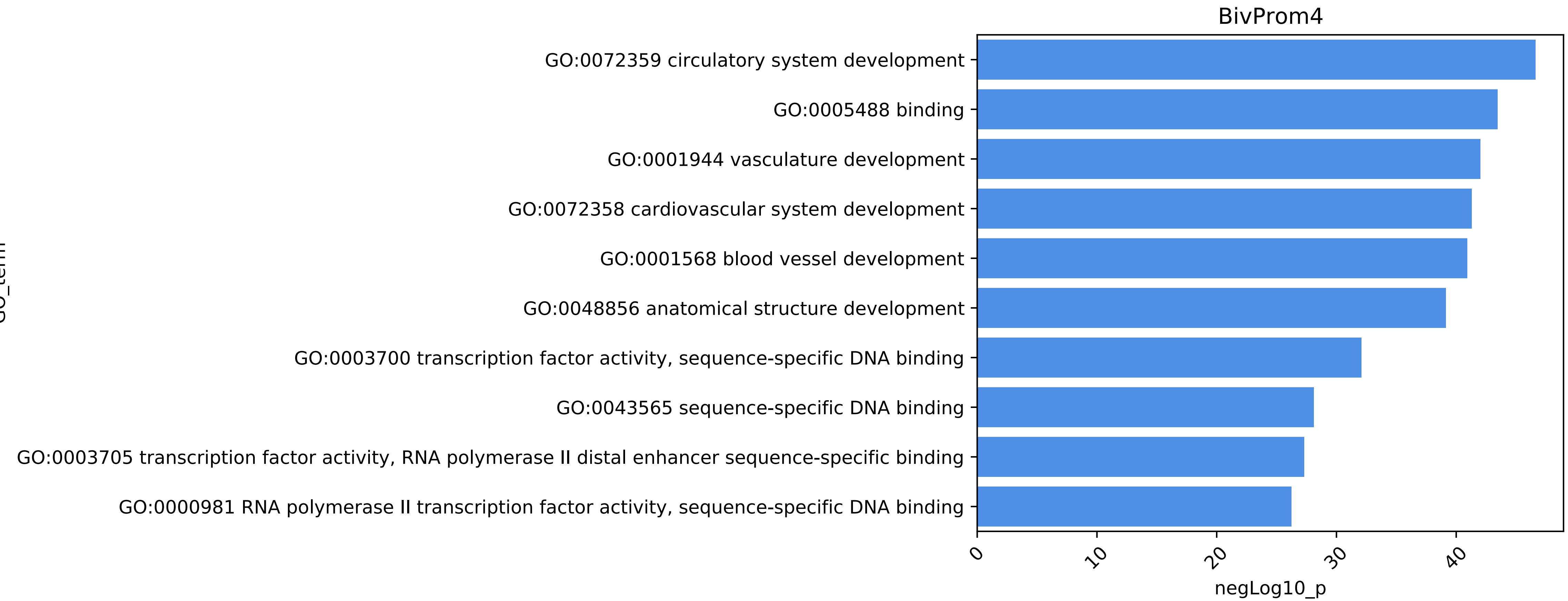
BivProm4
GO:0072359 circulatory system development
GO:0005488 binding
GO:0001944 vasculature development
GO:0072358 cardiovascular system development
GO:0001568 blood vessel development
GO:0048856 anatomical structure development
GO:0003700 transcription factor activity, sequence-specific DNA binding
GO:0043565 sequence-specific DNA binding
GO:0003705 transcription factor activity, RNA polymerase II distal enhancer sequence-specific binding
GO:0000981 RNA polymerase II transcription factor activity, sequence-specific DNA binding
0
10
20
30
40
negLog10_p

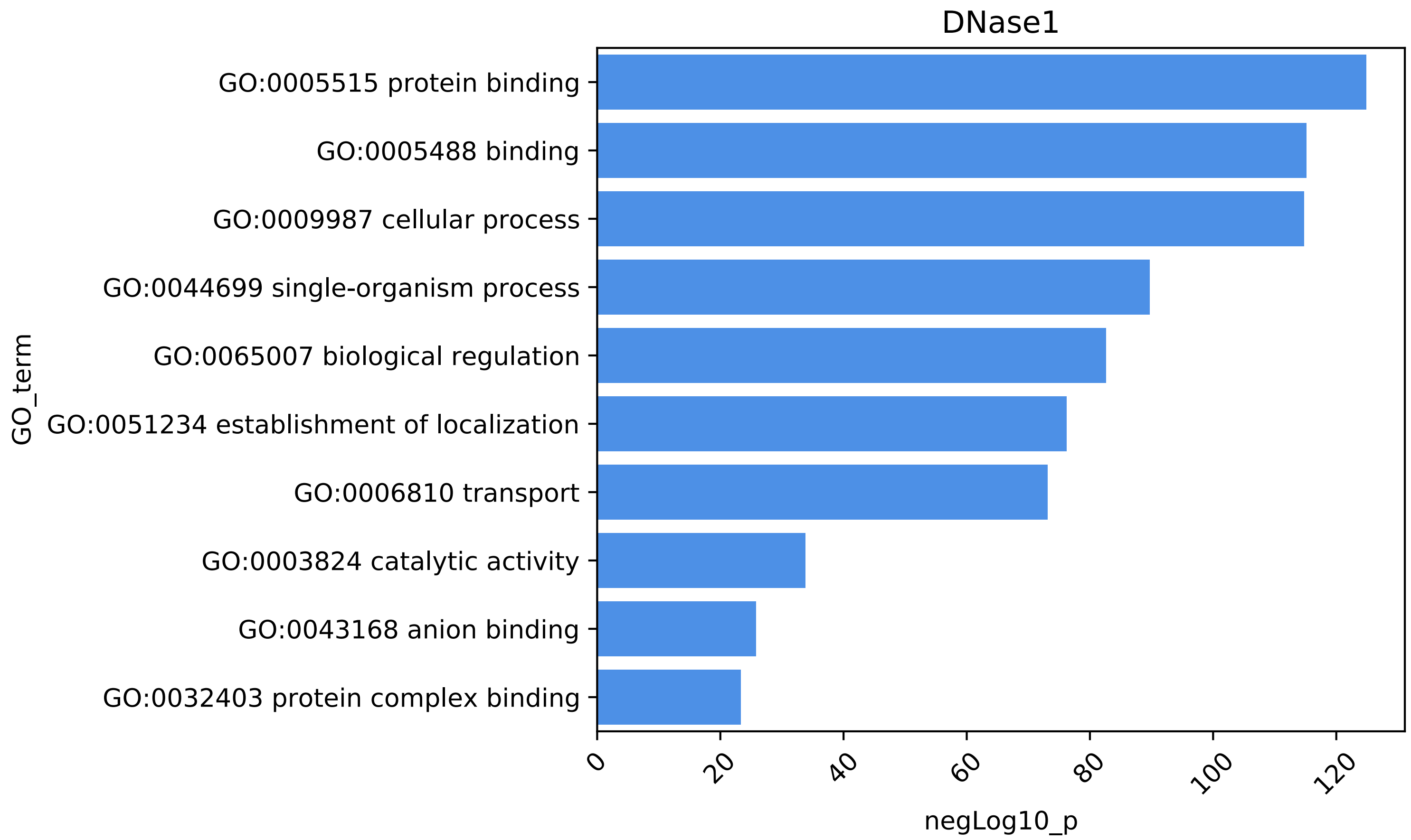

DNase1
GO:0005515 protein binding
GO:0005488 binding
GO:0009987 cellular process
GO:0044699 single-organism process
GO:0065007 biological regulation
GO:0051234 establishment of localization
GO:0006810 transport
GO:0003824 catalytic activity
GO:0043168 anion binding
GO:0032403 protein complex binding
GO_term
0
20
40
60
80
100
120
negLog10_p

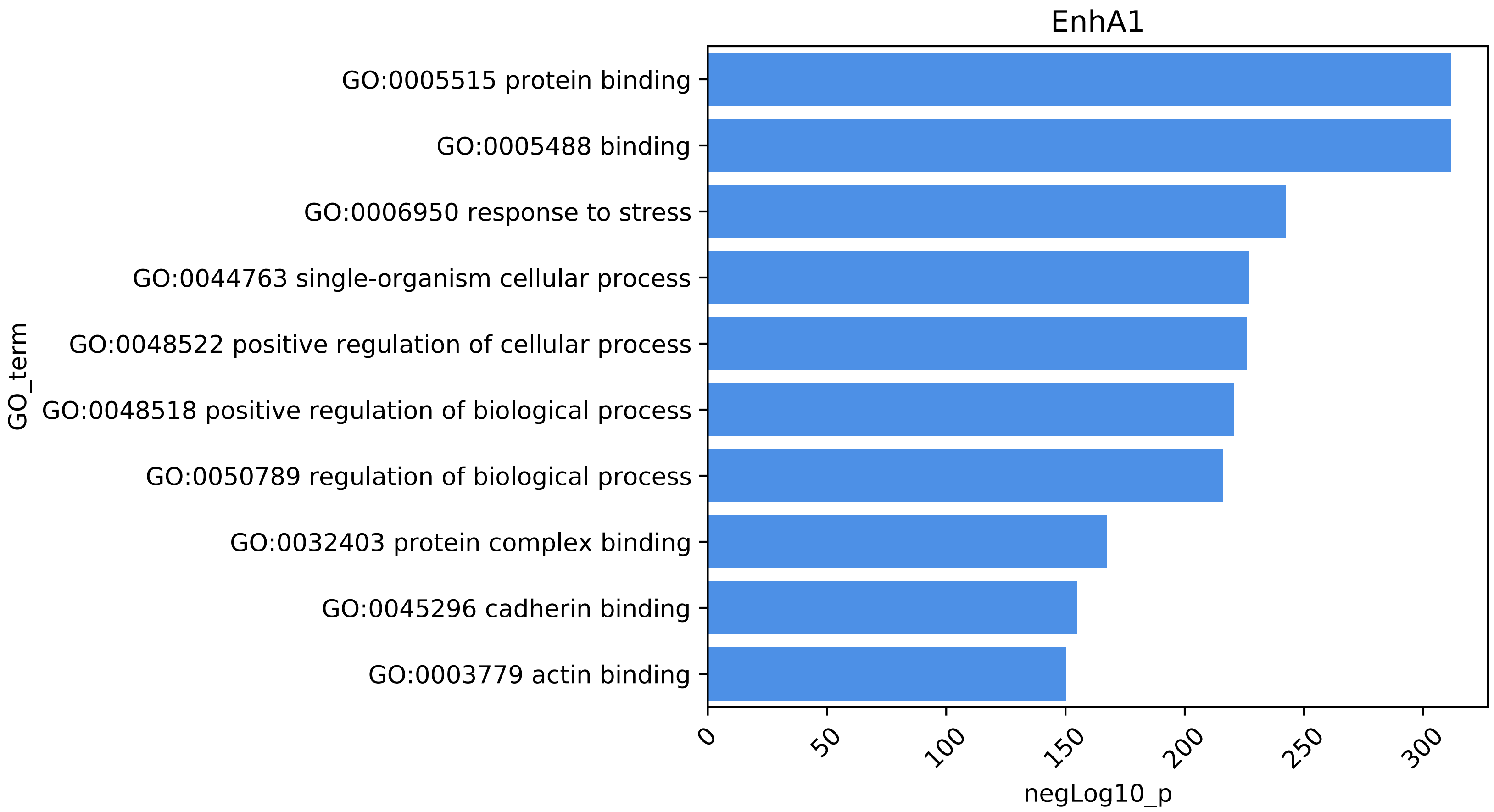
EnhA1
GO_term
GO:0005515 protein binding
GO:0005488 binding
GO:0006950 response to stress
GO:0044763 single-organism cellular process
GO:0048522 positive regulation of cellular process
GO:0048518 positive regulation of biological process
GO:0050789 regulation of biological process
GO:0032403 protein complex binding
GO:0045296 cadherin binding
GO:0003779 actin binding
0
50
100
150
200
250
300
negLog10_p

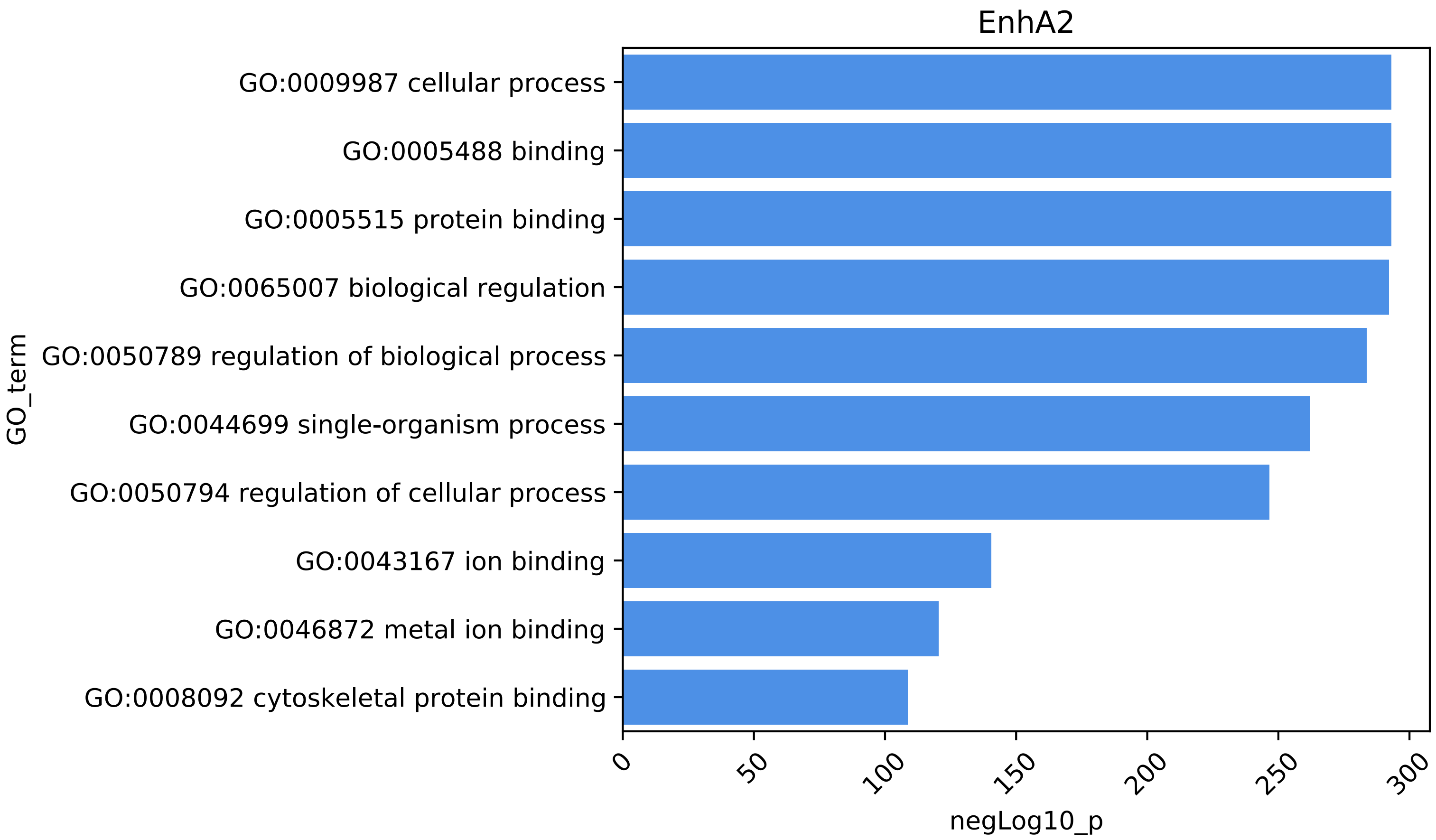
EnhA2
GO:0009987 cellular process
GO:0005488 binding
GO:0005515 protein binding
GO:0065007 biological regulation
GO:0050789 regulation of biological process
GO:0044699 single-organism process
GO:0050794 regulation of cellular process
GO:0043167 ion binding
GO:0046872 metal ion binding
GO:0008092 cytoskeletal protein binding
GO_term
0
50
100
150
200
250
300
negLog10_p

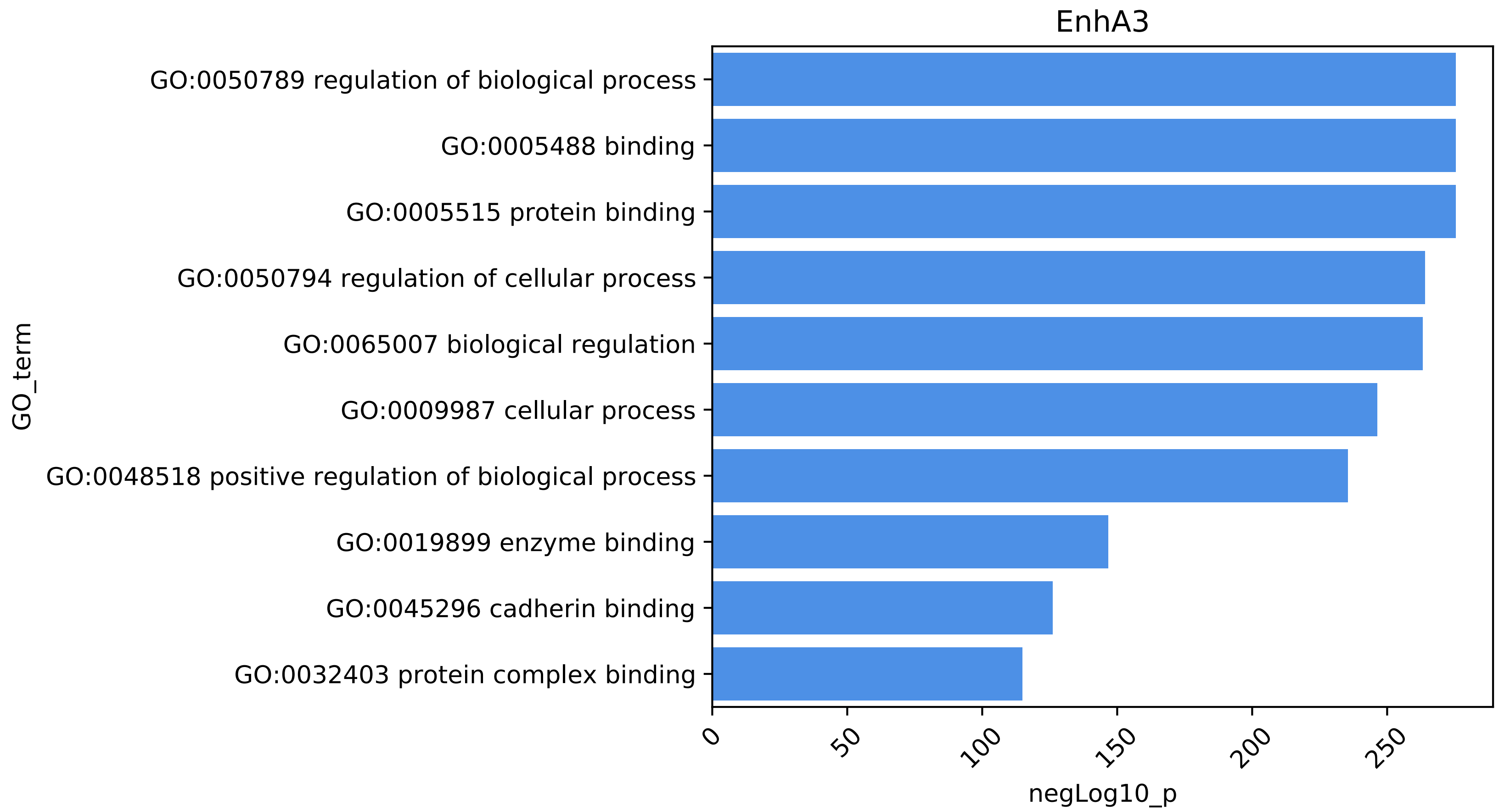
EnhA3
GO:0050789 regulation of biological process
GO:0005488 binding
GO:0005515 protein binding
GO:0050794 regulation of cellular process
GO:0065007 biological regulation
GO:0009987 cellular process
GO:0048518 positive regulation of biological process
GO:0019899 enzyme binding
GO:0045296 cadherin binding
GO:0032403 protein complex binding
GO_term
0
50
100
150
200
250
negLog10_p

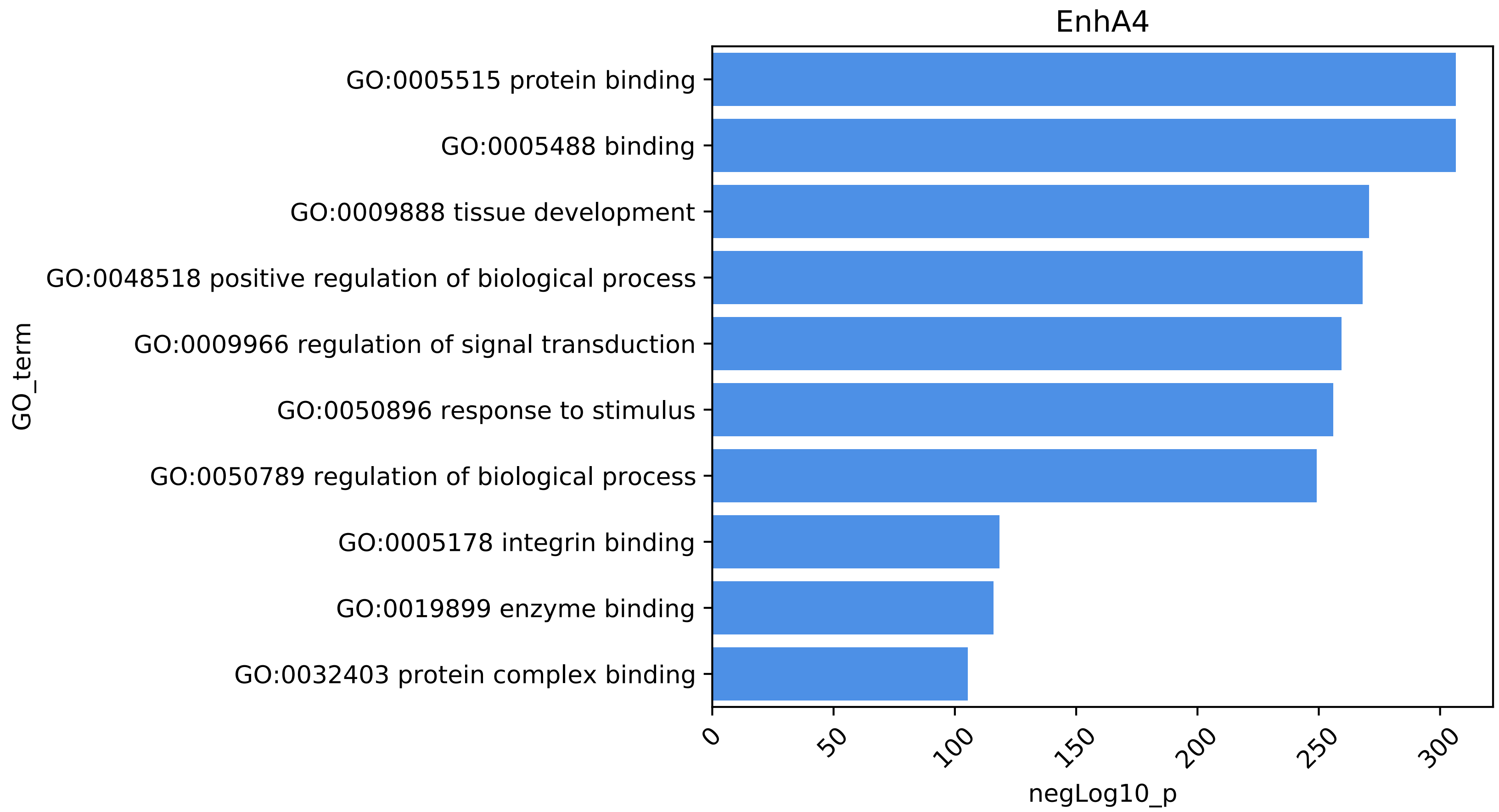

EnhA4
GO_term
GO:0005515 protein binding
GO:0005488 binding
GO:0009888 tissue development
GO:0048518 positive regulation of biological process
GO:0009966 regulation of signal transduction
GO:0050896 response to stimulus
GO:0050789 regulation of biological process
GO:0005178 integrin binding
GO:0019899 enzyme binding
GO:0032403 protein complex binding
0
50
100
150
200
250
300
negLog10_p

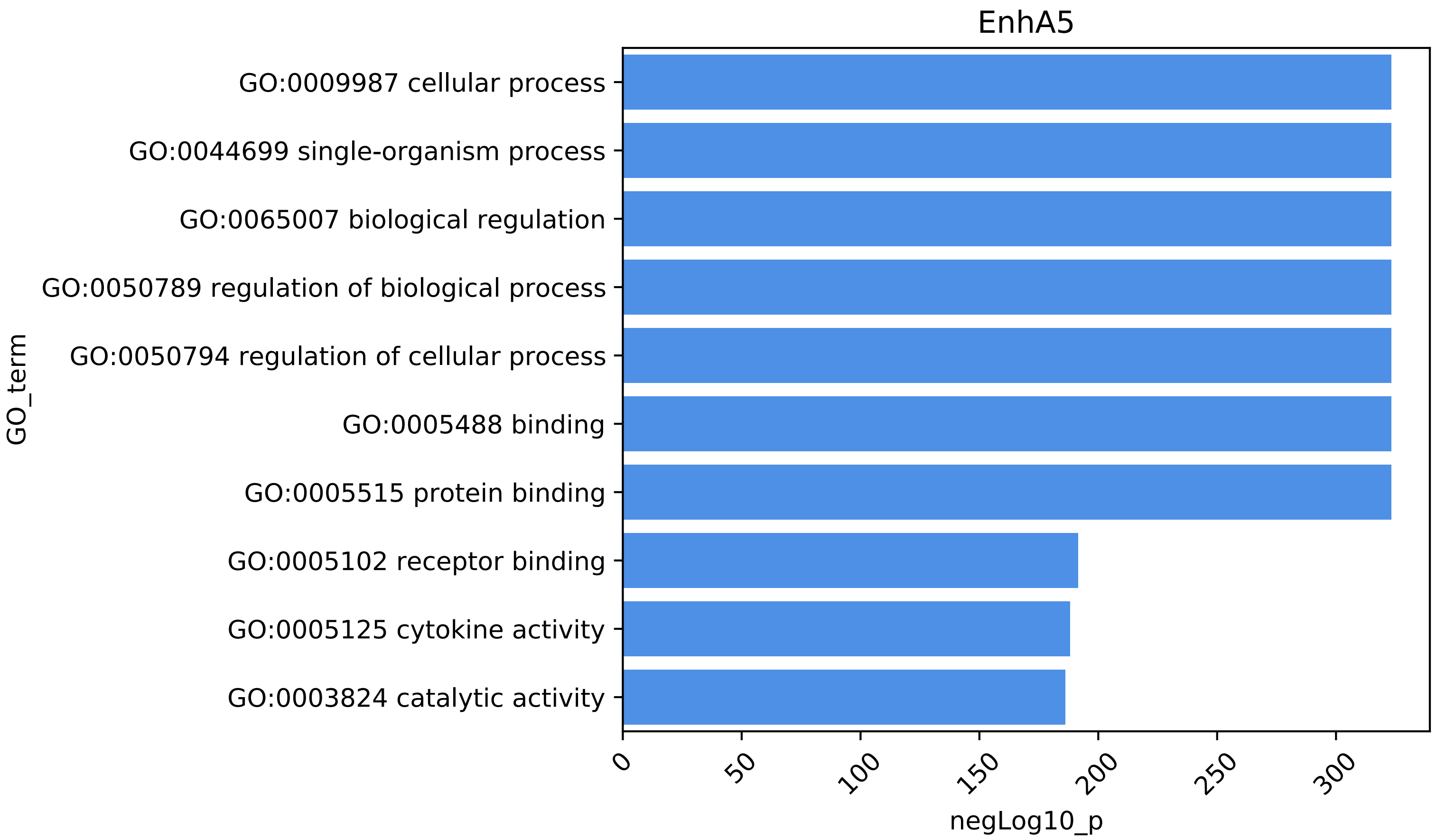
EnhA5
GO_term
GO:0009987 cellular process
GO:0044699 single-organism process
GO:0065007 biological regulation
GO:0050789 regulation of biological process
GO:0050794 regulation of cellular process
GO:0005488 binding
GO:0005515 protein binding
GO:0005102 receptor binding
GO:0005125 cytokine activity
GO:0003824 catalytic activity
0
50
100
150
200
250
300
negLog10_p

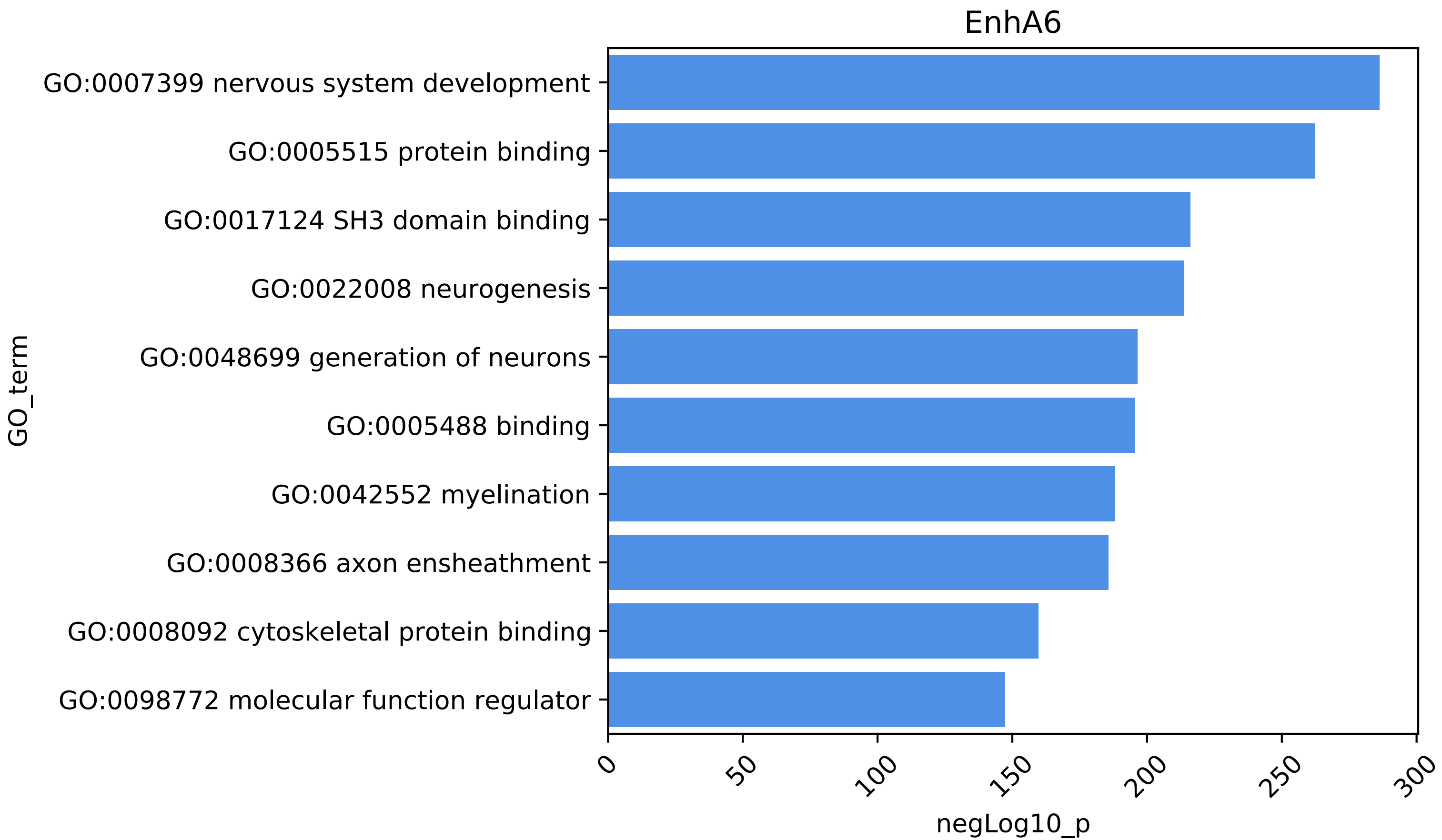
EnhA6
GO:0007399 nervous system development
GO:0005515 protein binding
GO:0017124 SH3 domain binding
GO:0022008 neurogenesis
GO:0048699 generation of neurons
GO:0005488 binding
GO:0042552 myelination
GO:0008366 axon ensheathment
GO:0008092 cytoskeletal protein binding
GO:0098772 molecular function regulator
GO_term
0
50
100
150
200
250
300
negLog10_p

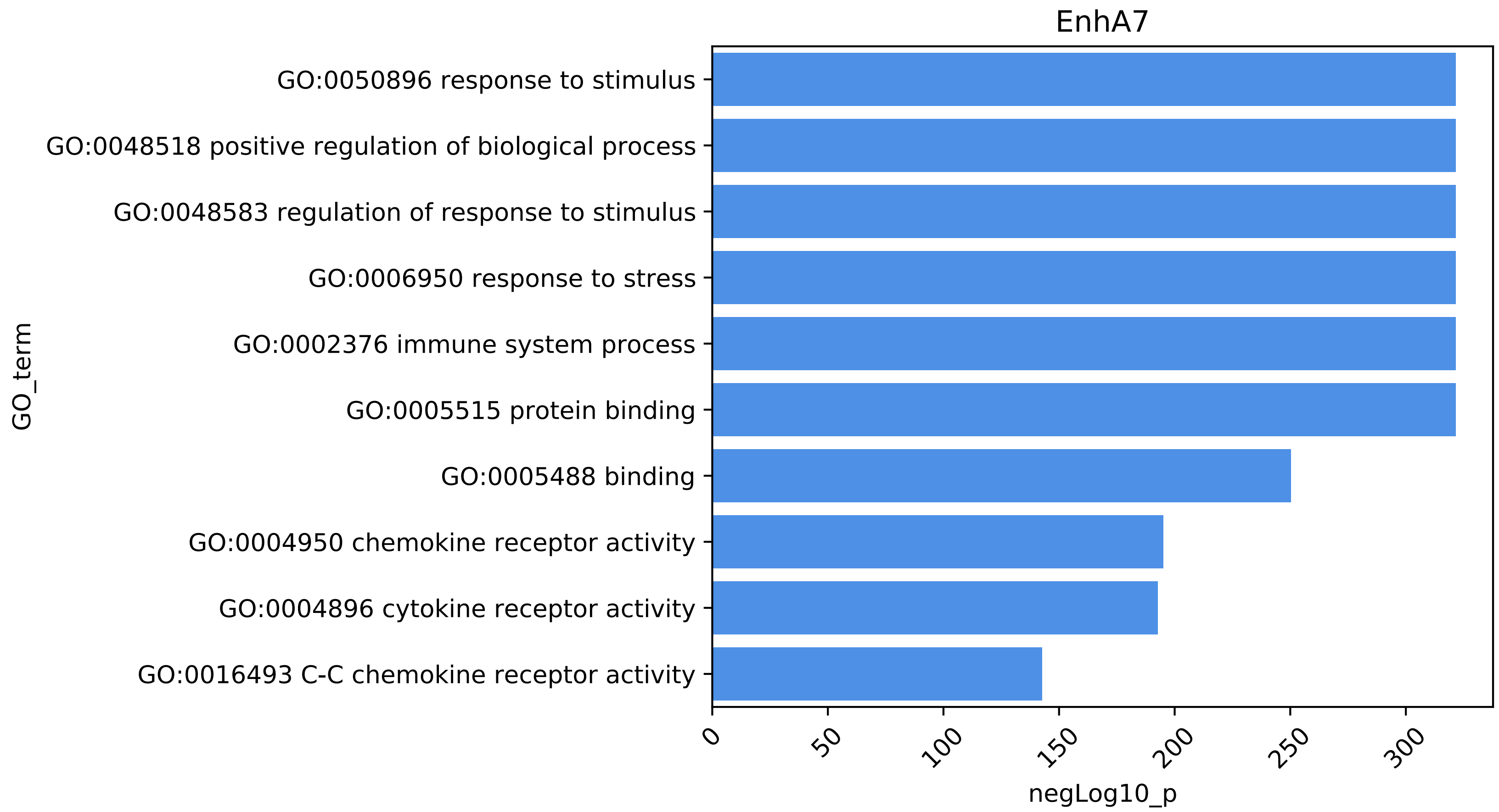
EnhA7
GO:0050896 response to stimulus
GO:0048518 positive regulation of biological process
GO:0048583 regulation of response to stimulus
GO:0006950 response to stress
GO:0002376 immune system process
GO:0005515 protein binding
GO:0005488 binding
GO:0004950 chemokine receptor activity
GO:0004896 cytokine receptor activity
GO:0016493 C-C chemokine receptor activity
GO_term
0
50
100
150
200
250
300
negLog10_p

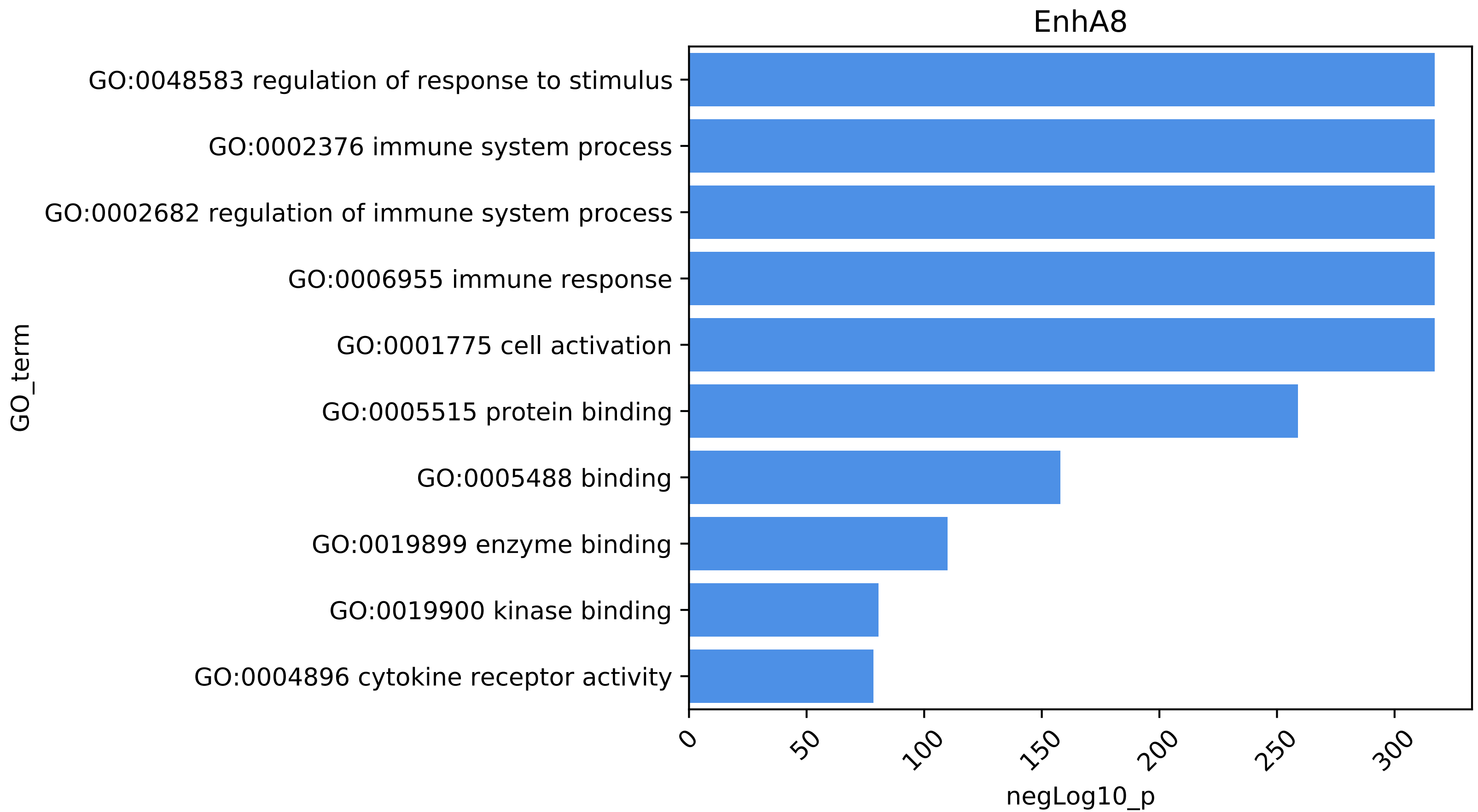
EnhA8
GO:0048583 regulation of response to stimulus
GO:0002376 immune system process
GO:0002682 regulation of immune system process
GO:0006955 immune response
GO:0001775 cell activation
GO:0005515 protein binding
GO:0005488 binding
GO:0019899 enzyme binding
GO:0019900 kinase binding
GO:0004896 cytokine receptor activity
GO_term
0
50
100
150
200
250
300
negLog10_p

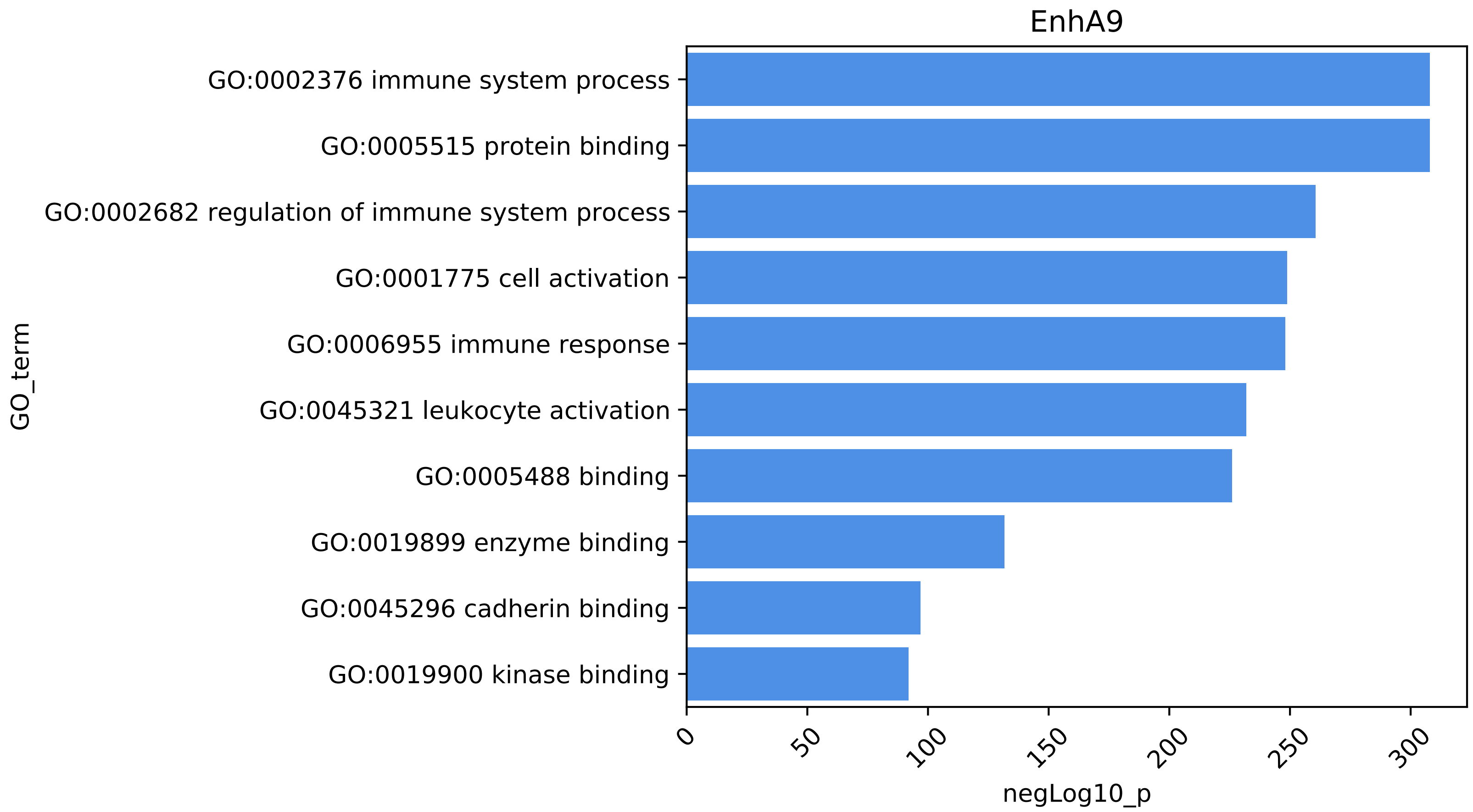
EnhA9
GO:0002376 immune system process
GO:0005515 protein binding
GO:0002682 regulation of immune system process
GO:0001775 cell activation
GO:0006955 immune response
GO:0045321 leukocyte activation
GO:0005488 binding
GO:0019899 enzyme binding
GO:0045296 cadherin binding
GO:0019900 kinase binding
GO_term
0
50
100
150
200
250
300
negLog10_p

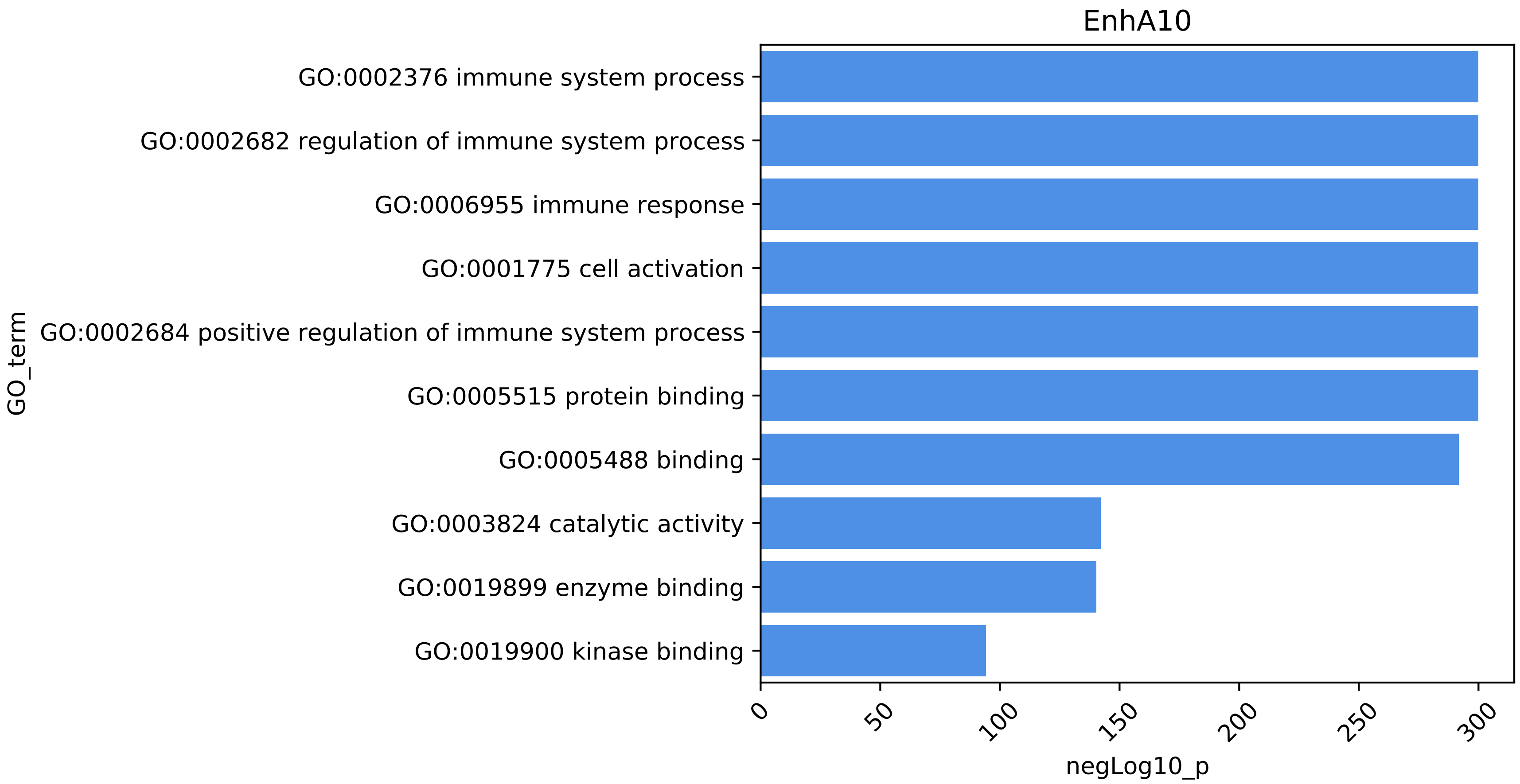
EnhA10
GO_term
GO:0002376 immune system process
GO:0002682 regulation of immune system process
GO:0006955 immune response
GO:0001775 cell activation
GO:0002684 positive regulation of immune system process
GO:0005515 protein binding
GO:0005488 binding
GO:0003824 catalytic activity
GO:0019899 enzyme binding
GO:0019900 kinase binding
0
50
100
150
200
250
300
negLog10_p

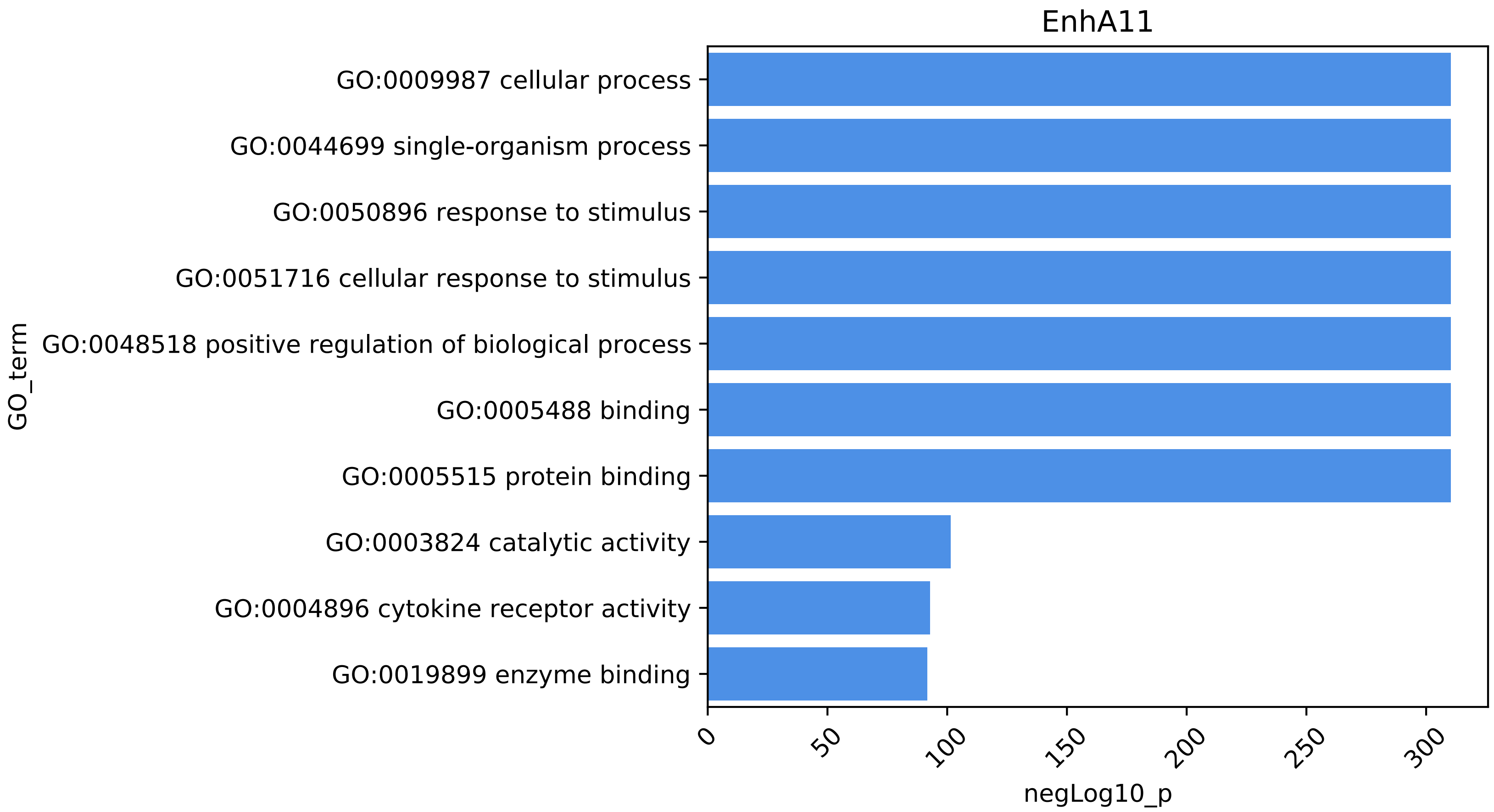

EnhA11
GO:0009987 cellular process
GO:0044699 single-organism process
GO:0050896 response to stimulus
GO:0051716 cellular response to stimulus
GO:0048518 positive regulation of biological process
GO:0005488 binding
GO:0005515 protein binding
GO:0003824 catalytic activity
GO:0004896 cytokine receptor activity
GO:0019899 enzyme binding
GO_term
0
50
100
150
200
250
300
negLog10_p

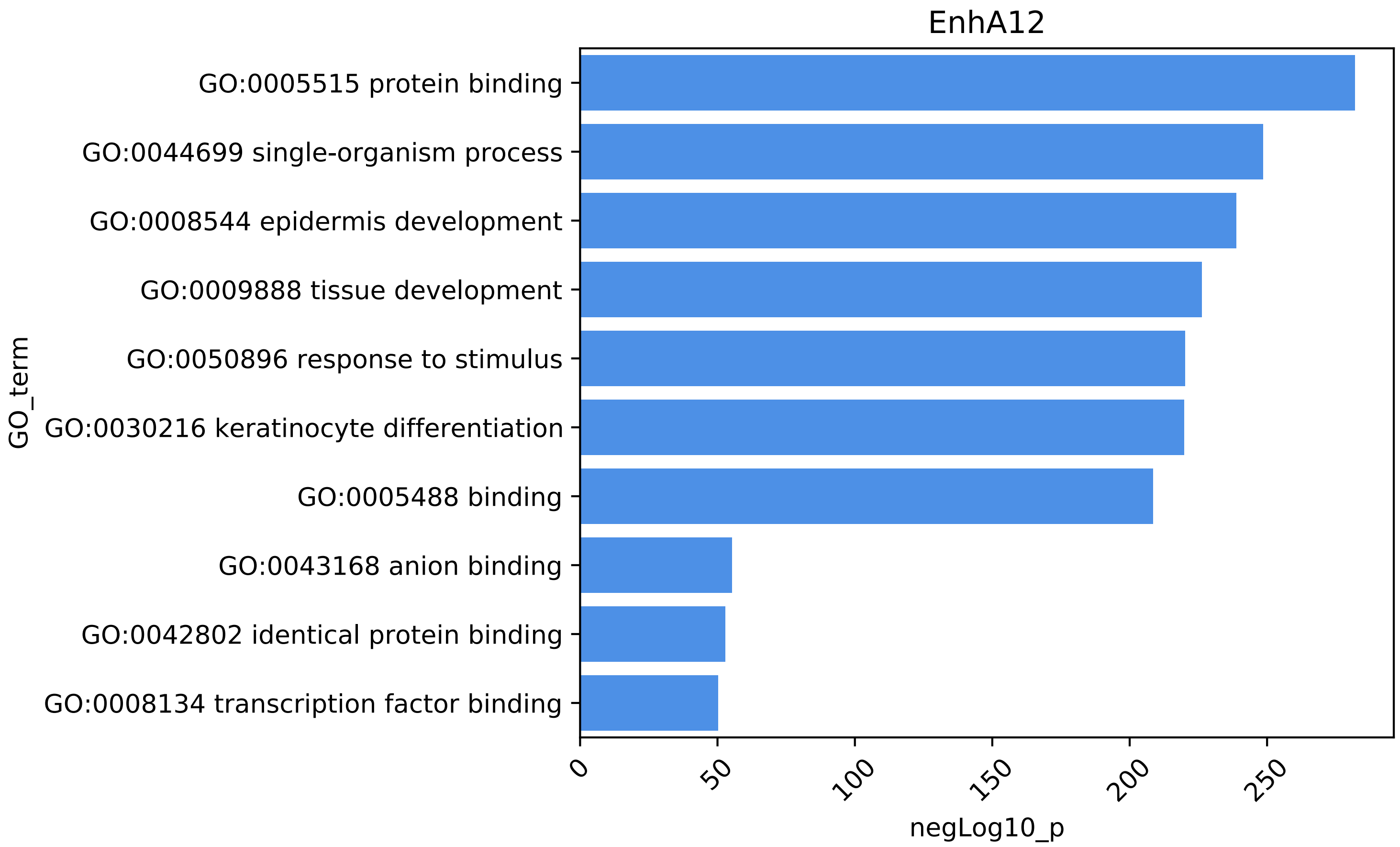
EnhA12
GO:0005515 protein binding
GO:0044699 single-organism process
GO:0008544 epidermis development
GO:0009888 tissue development
GO:0050896 response to stimulus
GO:0030216 keratinocyte differentiation
GO:0005488 binding
GO:0043168 anion binding
GO:0042802 identical protein binding
GO:0008134 transcription factor binding
GO_term
0
50
100
150
200
250
negLog10_p

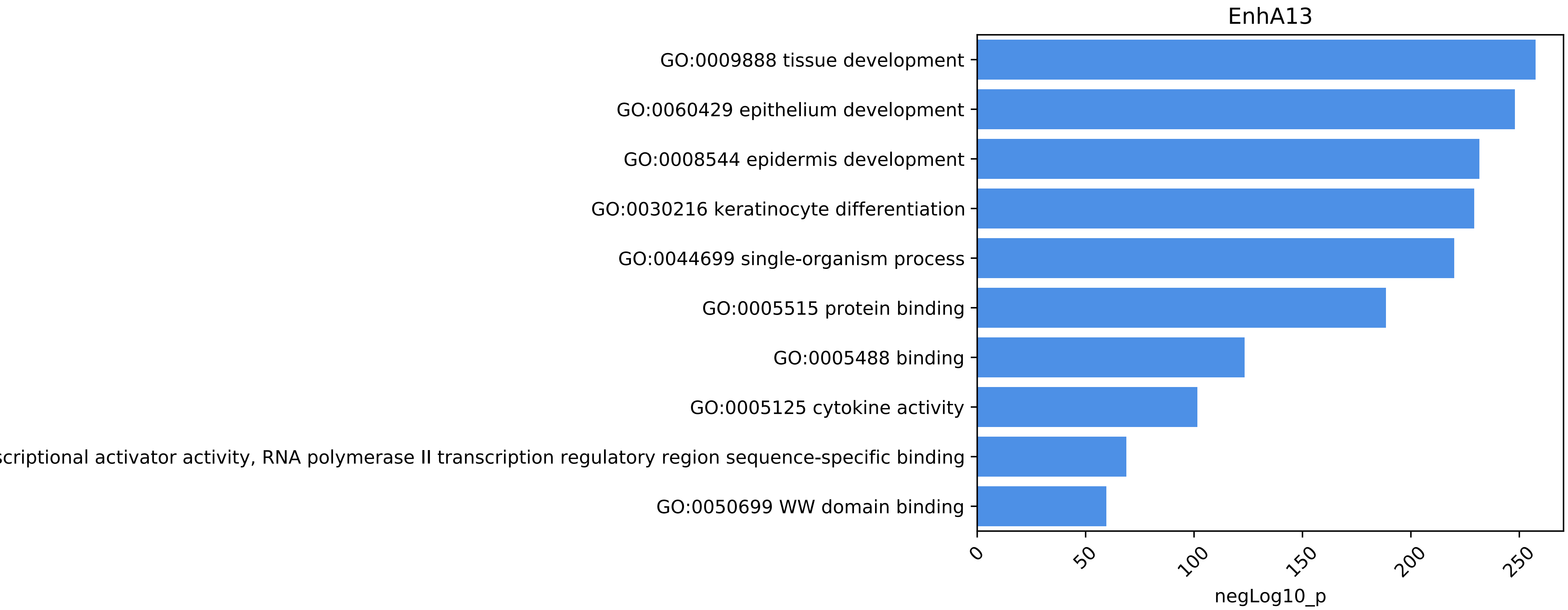
EnhA13
GO:0009888 tissue development
GO:0060429 epithelium development
GO:0008544 epidermis development
GO:0030216 keratinocyte differentiation
GO:0044699 single-organism process
GO:0005515 protein binding
GO:0005488 binding
GO:0005125 cytokine activity
scriptional activator activity, RNA polymerase II transcription regulatory region sequence-specific binding
GO:0050699 WW domain binding
0
50
100
150
200
250
negLog10_p

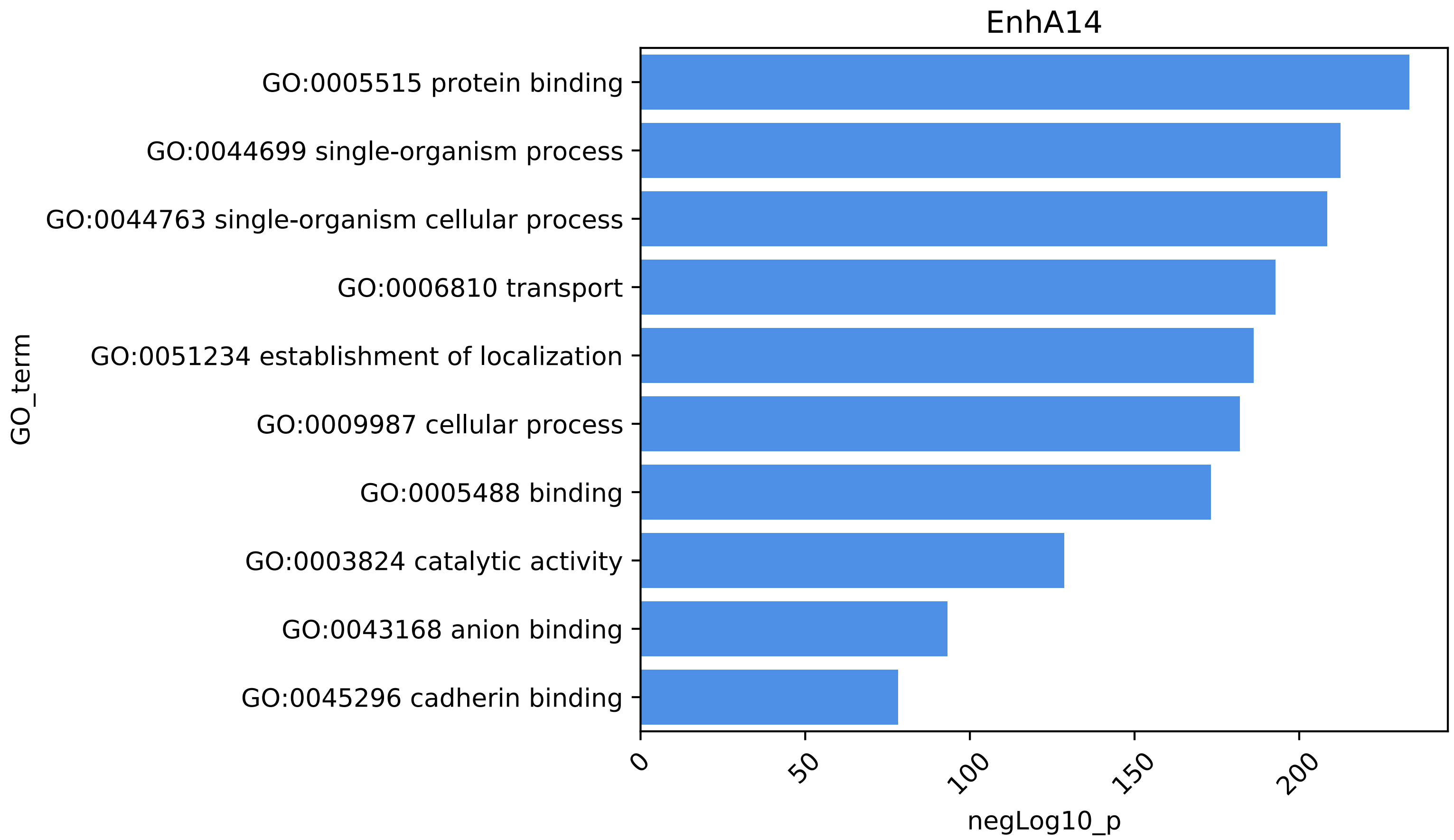

EnhA14
GO:0005515 protein binding
GO:0044699 single-organism process
GO:0044763 single-organism cellular process
GO:0006810 transport
GO:0051234 establishment of localization
GO:0009987 cellular process
GO:0005488 binding
GO:0003824 catalytic activity
GO:0043168 anion binding
GO:0045296 cadherin binding
GO_term
0
50
100
150
200
negLog10_p

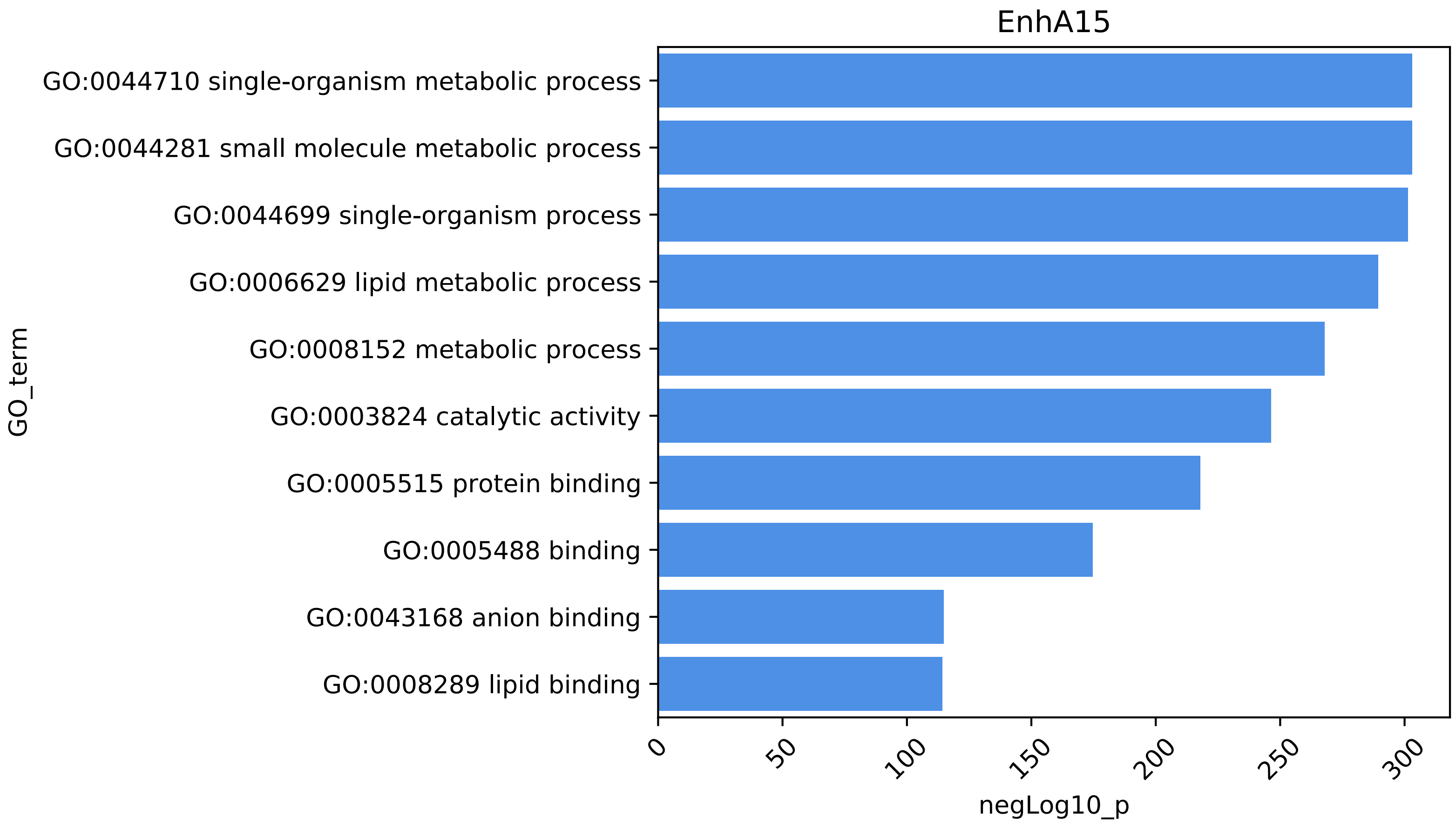
EnhA15
GO_term
GO:0044710 single-organism metabolic process
GO:0044281 small molecule metabolic process
GO:0044699 single-organism process
GO:0006629 lipid metabolic process
GO:0008152 metabolic process
GO:0003824 catalytic activity
GO:0005515 protein binding
GO:0005488 binding
GO:0043168 anion binding
GO:0008289 lipid binding
0
50
100
150
200
250
300
negLog10_p

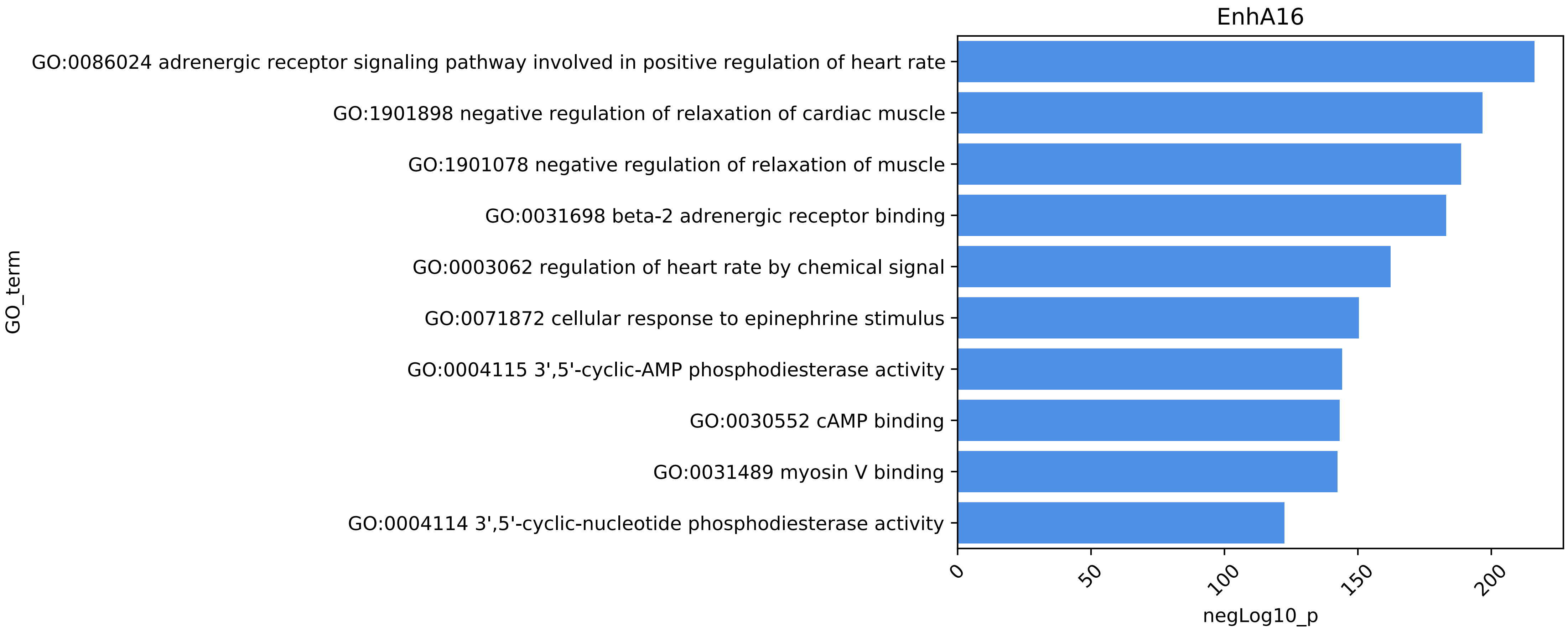
EnhA16
GO_term
GO:0086024 adrenergic receptor signaling pathway involved in positive regulation of heart rate
GO:1901898 negative regulation of relaxation of cardiac muscle
GO:1901078 negative regulation of relaxation of muscle
GO:0031698 beta-2 adrenergic receptor binding
GO:0003062 regulation of heart rate by chemical signal
GO:0071872 cellular response to epinephrine stimulus
GO:0004115 3',5'-cyclic-AMP phosphodiesterase activity
GO:0030552 cAMP binding
GO:0031489 myosin V binding
GO:0004114 3',5'-cyclic-nucleotide phosphodiesterase activity
0
50
100
150
200
negLog10_p

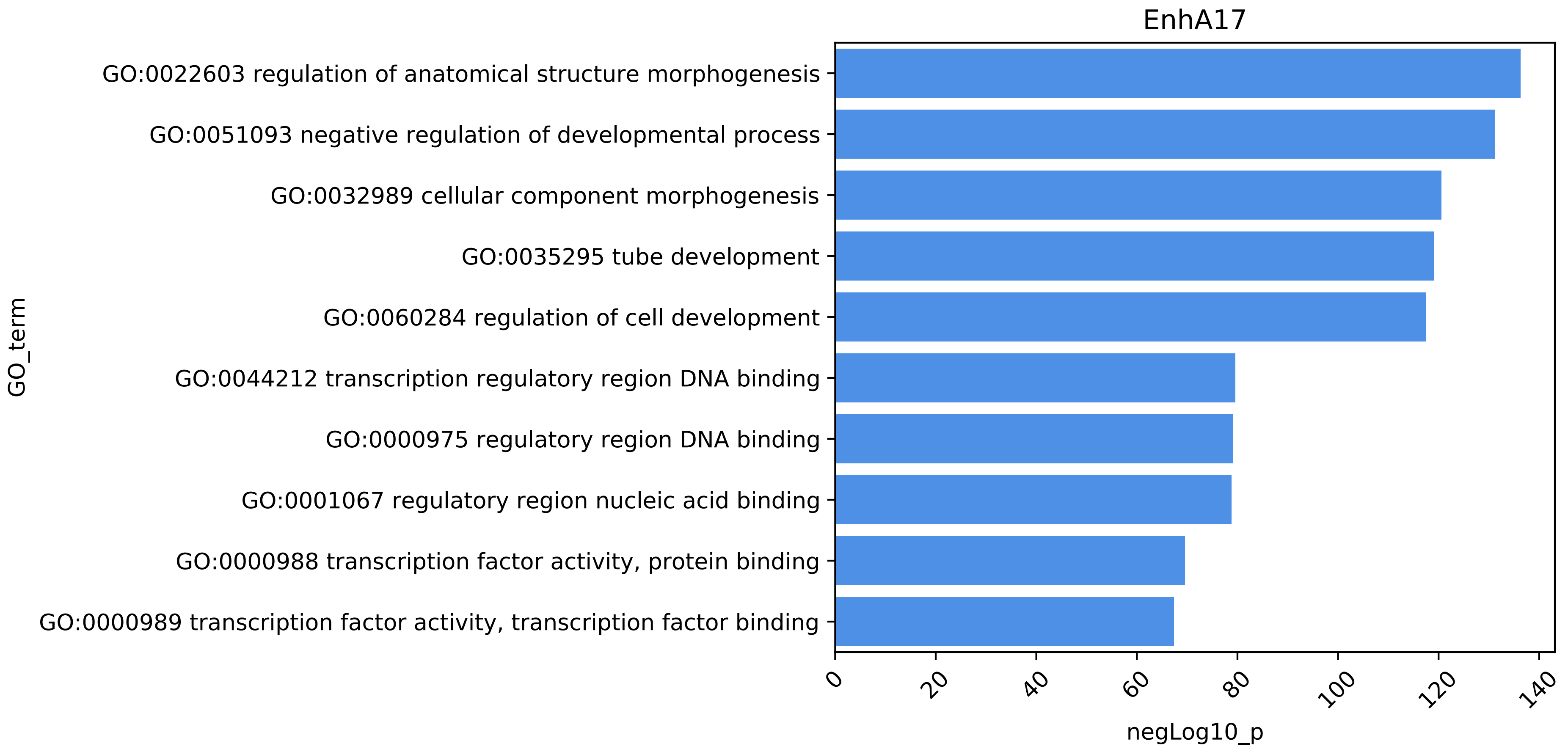

EnhA17
GO:0022603 regulation of anatomical structure morphogenesis
GO:0051093 negative regulation of developmental process
GO:0032989 cellular component morphogenesis
GO:0035295 tube development
GO:0060284 regulation of cell development
GO:0044212 transcription regulatory region DNA binding
GO:0000975 regulatory region DNA binding
GO:0001067 regulatory region nucleic acid binding
GO:0000988 transcription factor activity, protein binding
GO:0000989 transcription factor activity, transcription factor binding
GO_term
0
20
40
60
80
100
120
140
negLog10_p

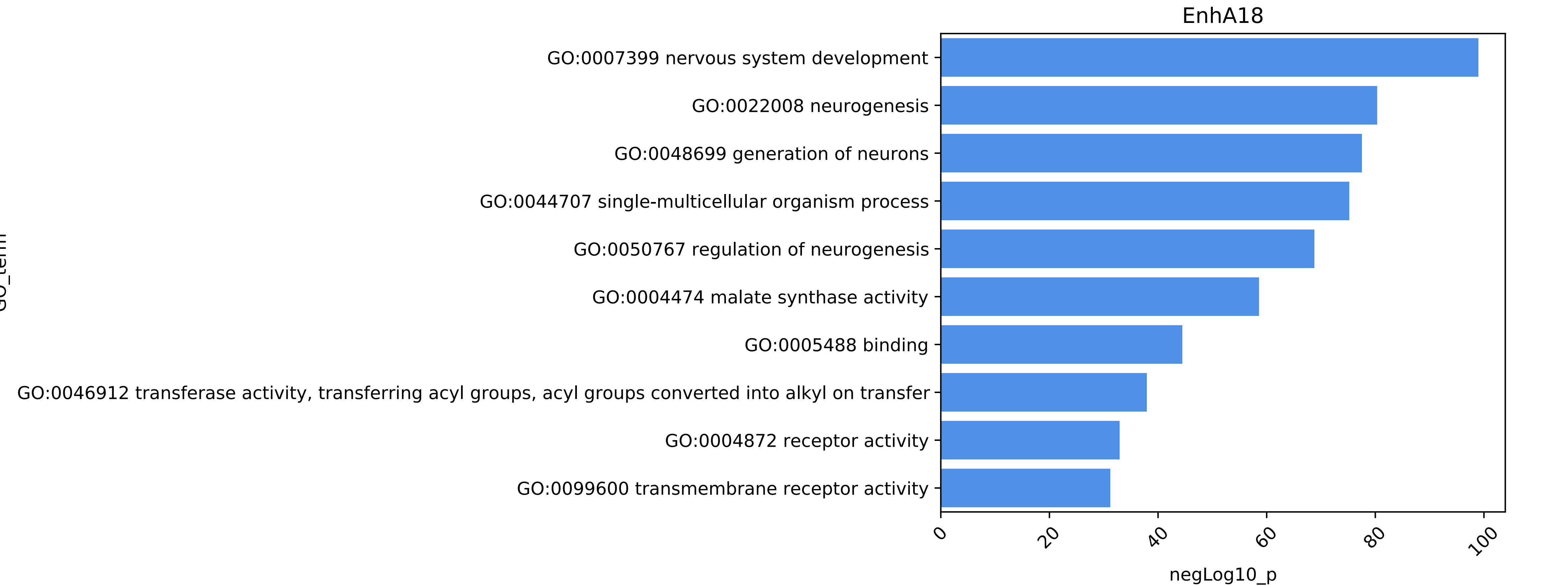
EnhA18
GO:0007399 nervous system development
GO:0022008 neurogenesis
GO:0048699 generation of neurons
GO:0044707 single-multicellular organism process
GO:0050767 regulation of neurogenesis
GO:0004474 malate synthase activity
GO:0005488 binding
GO:0046912 transferase activity, transferring acyl groups, acyl groups converted into alkyl on transfer
GO:0004872 receptor activity
GO:0099600 transmembrane receptor activity
GO_term
0
20
40
60
80
100
negLog10_p

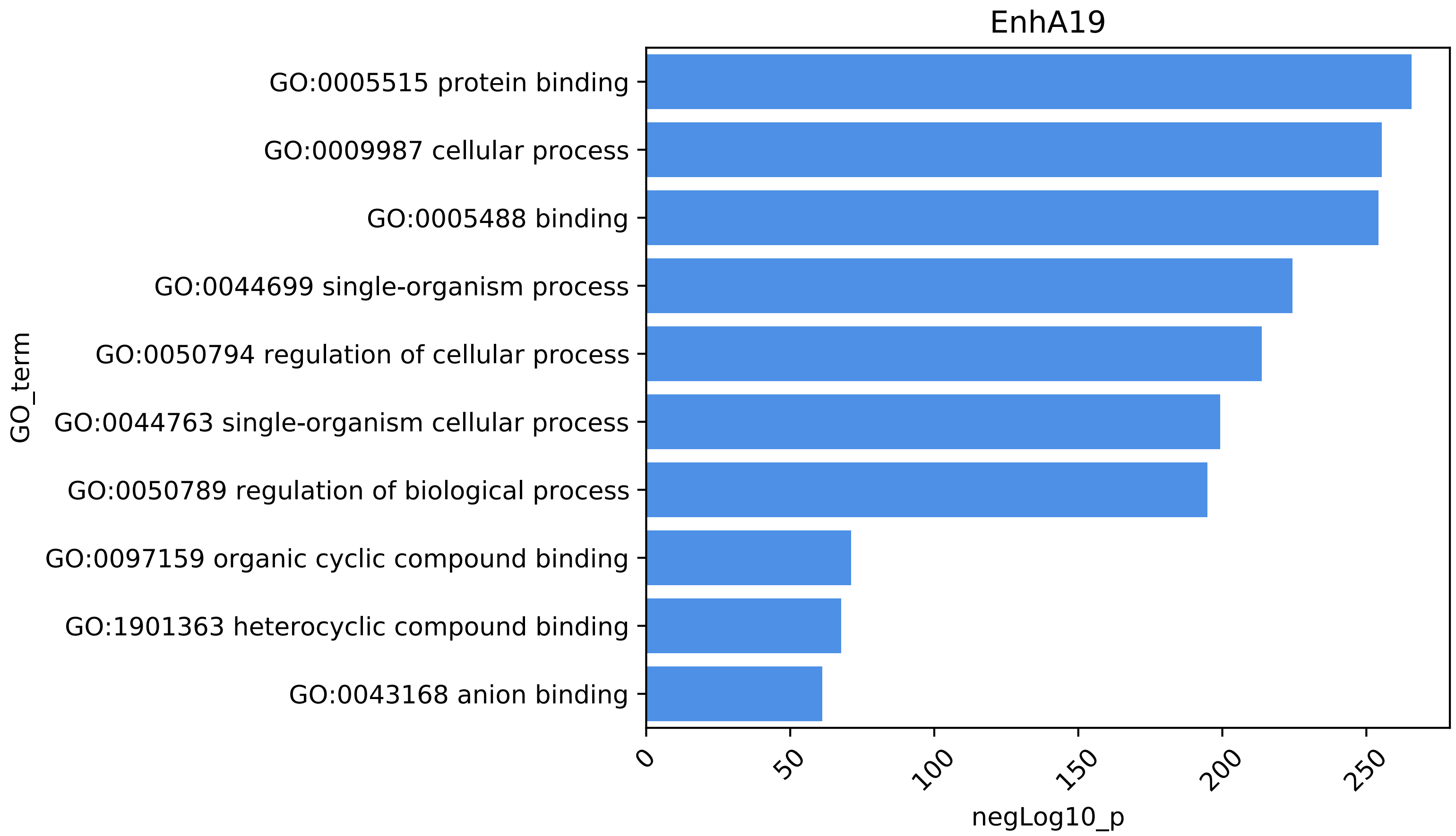
EnhA19
GO:0005515 protein binding
GO:0009987 cellular process
GO:0005488 binding
GO:0044699 single-organism process
GO:0050794 regulation of cellular process
GO:0044763 single-organism cellular process
GO:0050789 regulation of biological process
GO:0097159 organic cyclic compound binding
GO:1901363 heterocyclic compound binding
GO:0043168 anion binding
GO_term
0
50
100
150
200
250
negLog10_p

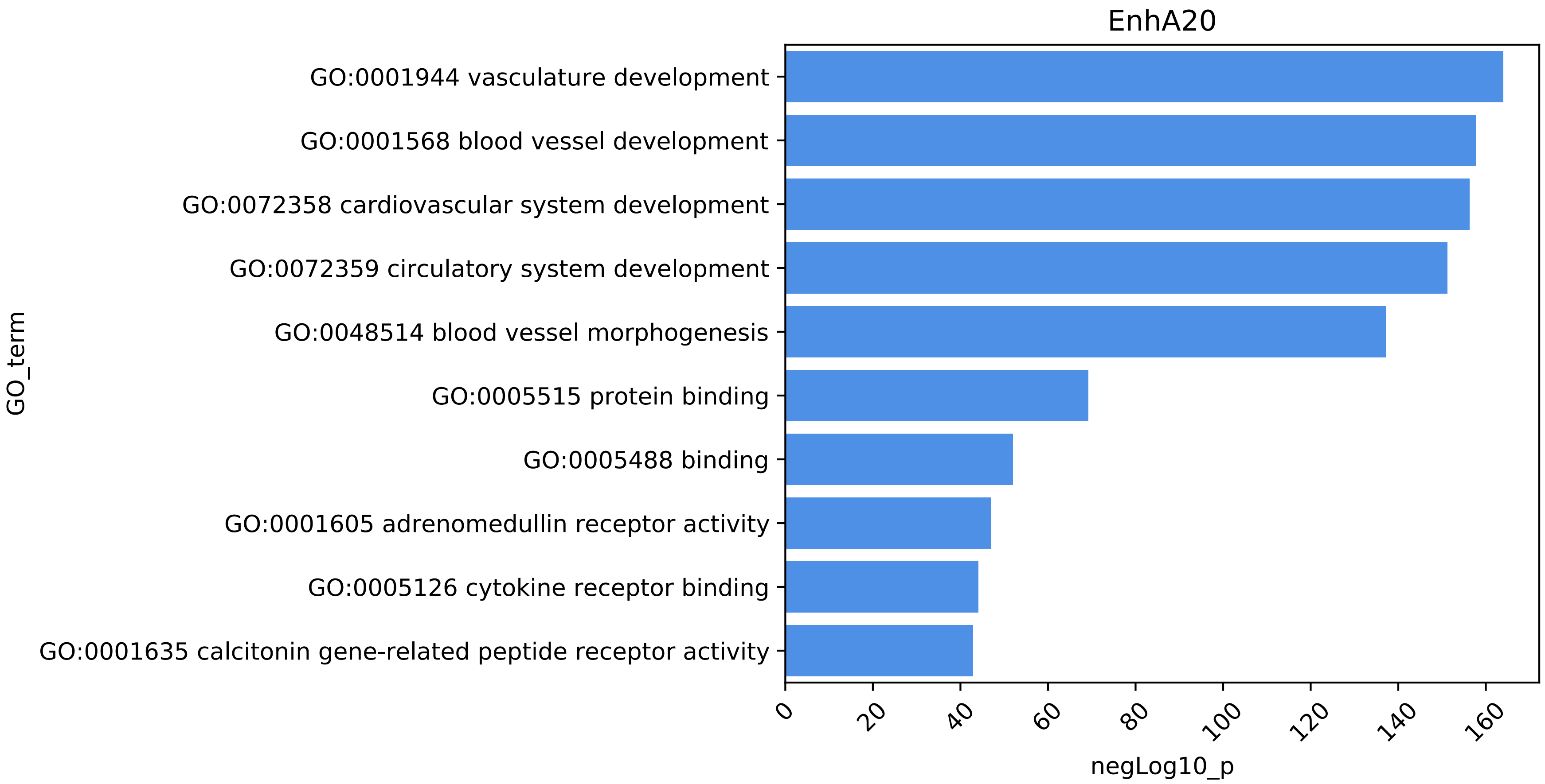
EnhA20
GO_term
GO:0001944 vasculature development
GO:0001568 blood vessel development
GO:0072358 cardiovascular system development
GO:0072359 circulatory system development
GO:0048514 blood vessel morphogenesis
GO:0005515 protein binding
GO:0005488 binding
GO:0001605 adrenomedullin receptor activity
GO:0005126 cytokine receptor binding
GO:0001635 calcitonin gene-related peptide receptor activity
0
20
40
60
80
100
120
140
160
negLog10_p

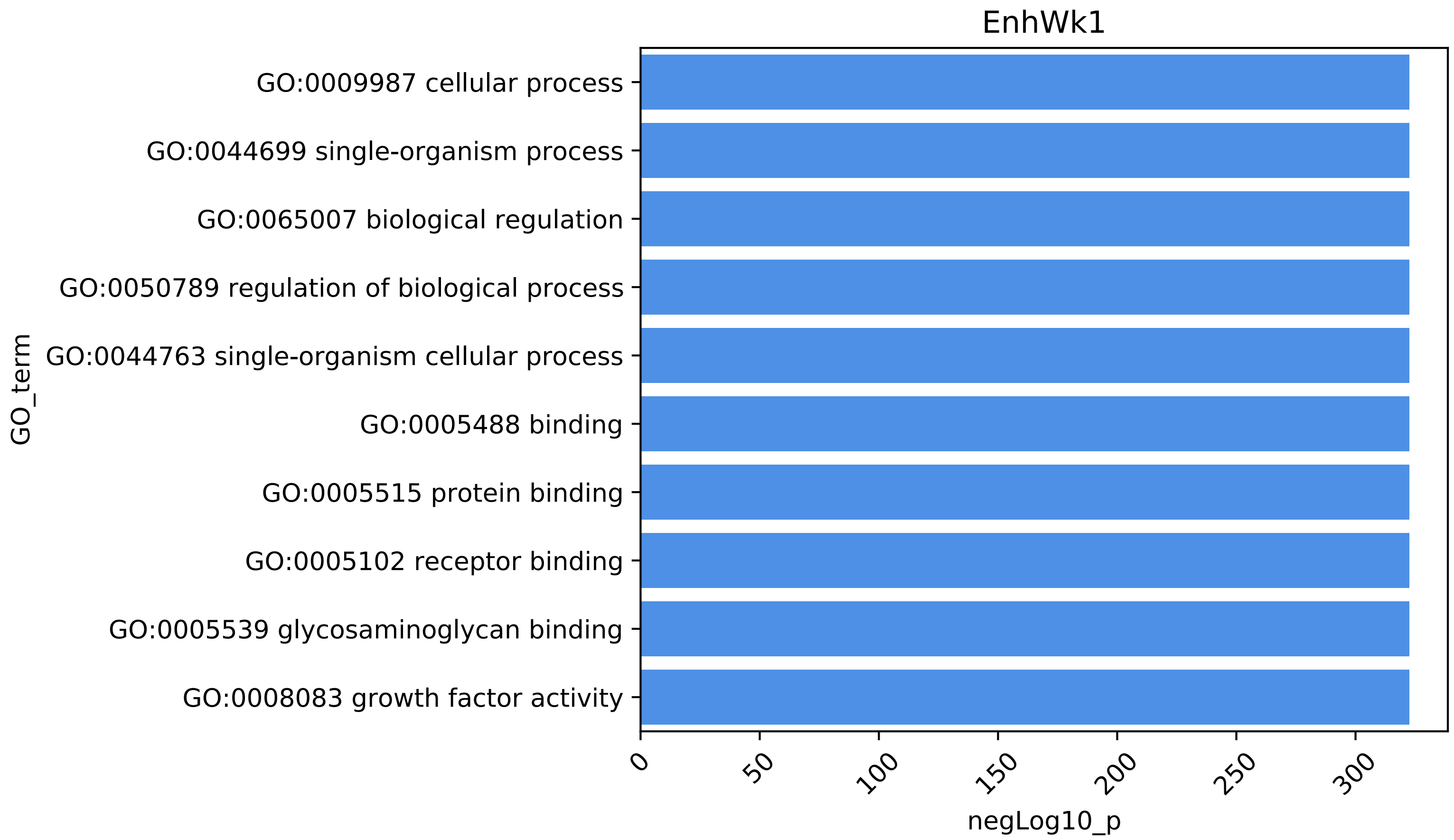

EnhWk1
GO_term
GO:0009987 cellular process
GO:0044699 single-organism process
GO:0065007 biological regulation
GO:0050789 regulation of biological process
GO:0044763 single-organism cellular process
GO:0005488 binding
GO:0005515 protein binding
GO:0005102 receptor binding
GO:0005539 glycosaminoglycan binding
GO:0008083 growth factor activity
0
50
100
150
200
250
300
negLog10_p

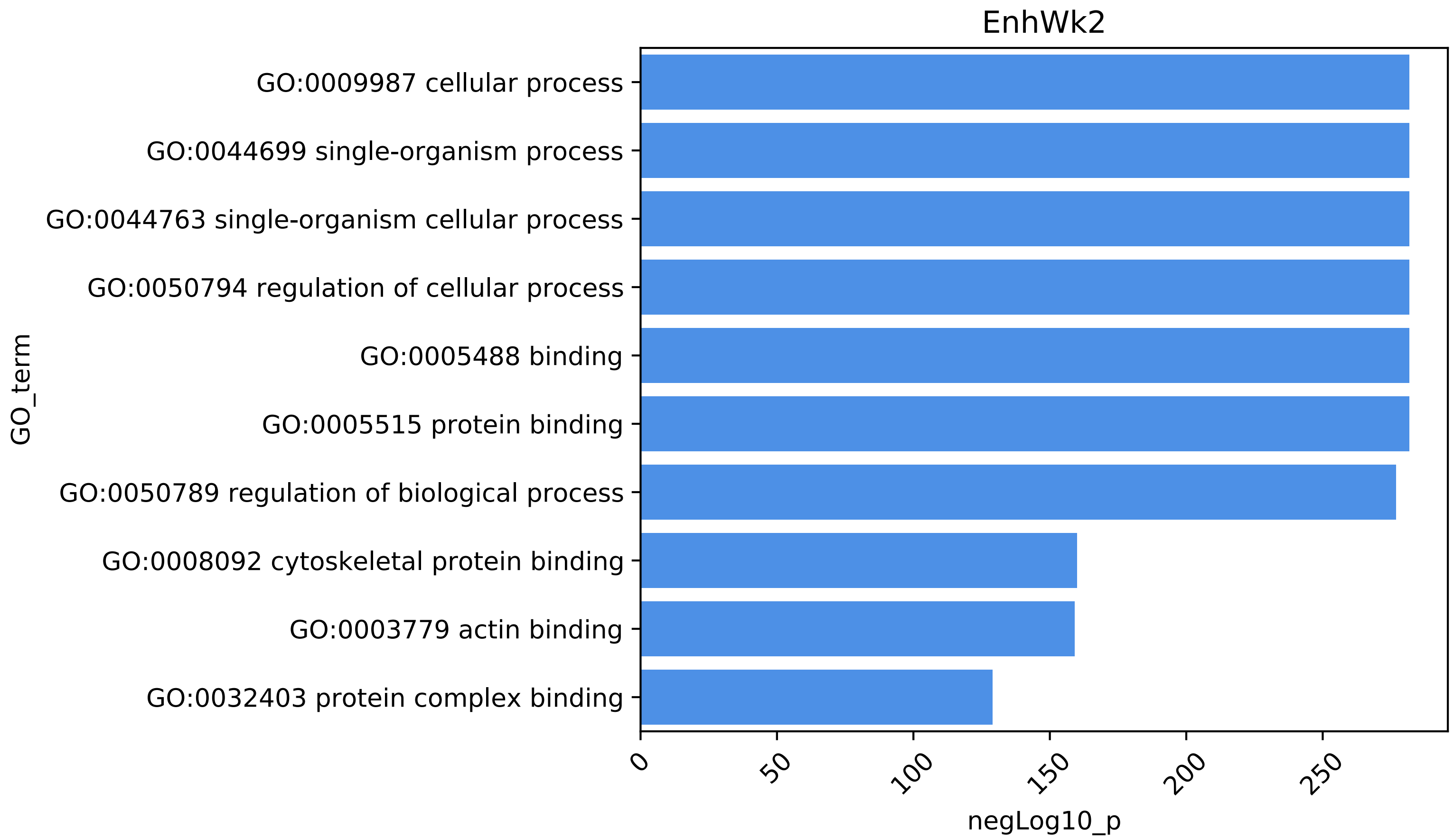
EnhWk2
GO:0009987 cellular process
GO:0044699 single-organism process
GO:0044763 single-organism cellular process
GO:0050794 regulation of cellular process
GO:0005488 binding
GO:0005515 protein binding
GO:0050789 regulation of biological process
GO:0008092 cytoskeletal protein binding
GO:0003779 actin binding
GO:0032403 protein complex binding
GO_term
0
50
100
150
200
250
negLog10_p

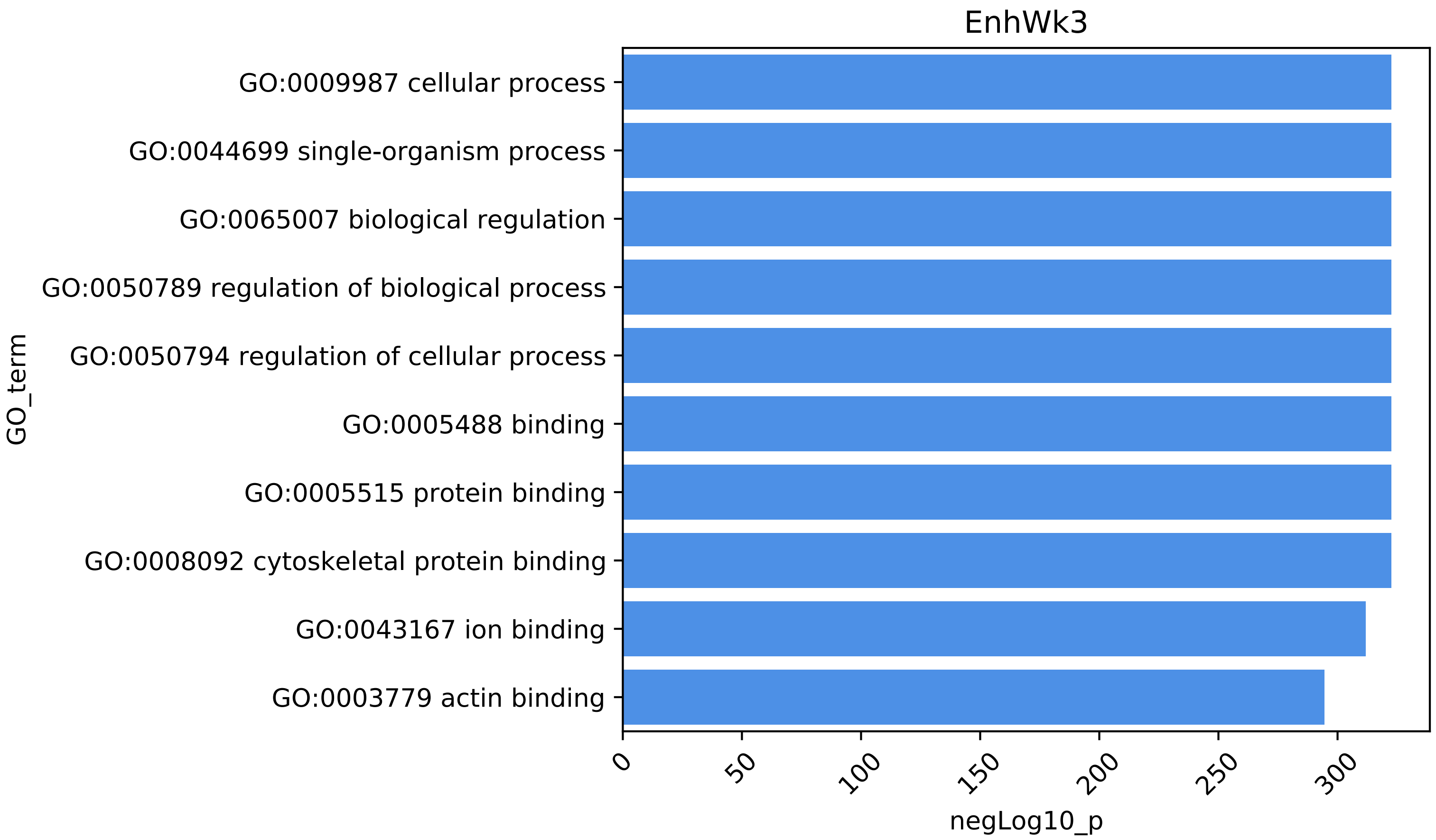
EnhWk3
GO_term
GO:0009987 cellular process
GO:0044699 single-organism process
GO:0065007 biological regulation
GO:0050789 regulation of biological process
GO:0050794 regulation of cellular process
GO:0005488 binding
GO:0005515 protein binding
GO:0008092 cytoskeletal protein binding
GO:0043167 ion binding
GO:0003779 actin binding
0
50
100
150
200
250
300
negLog10_p

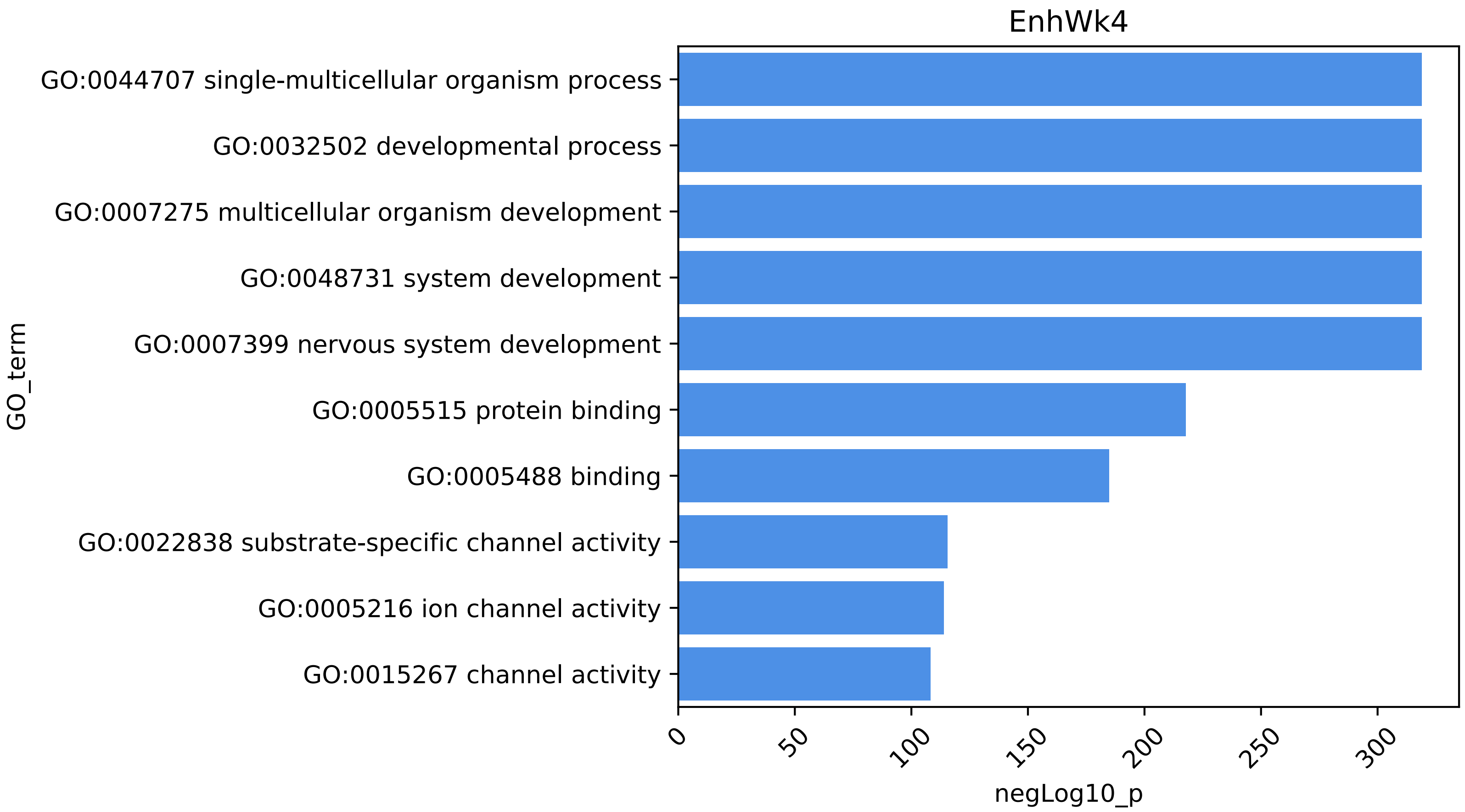

EnhWk4
GO_term
GO:0044707 single-multicellular organism process
GO:0032502 developmental process
GO:0007275 multicellular organism development
GO:0048731 system development
GO:0007399 nervous system development
GO:0005515 protein binding
GO:0005488 binding
GO:0022838 substrate-specific channel activity
GO:0005216 ion channel activity
GO:0015267 channel activity
0
50
100
150
200
250
300
negLog10_p

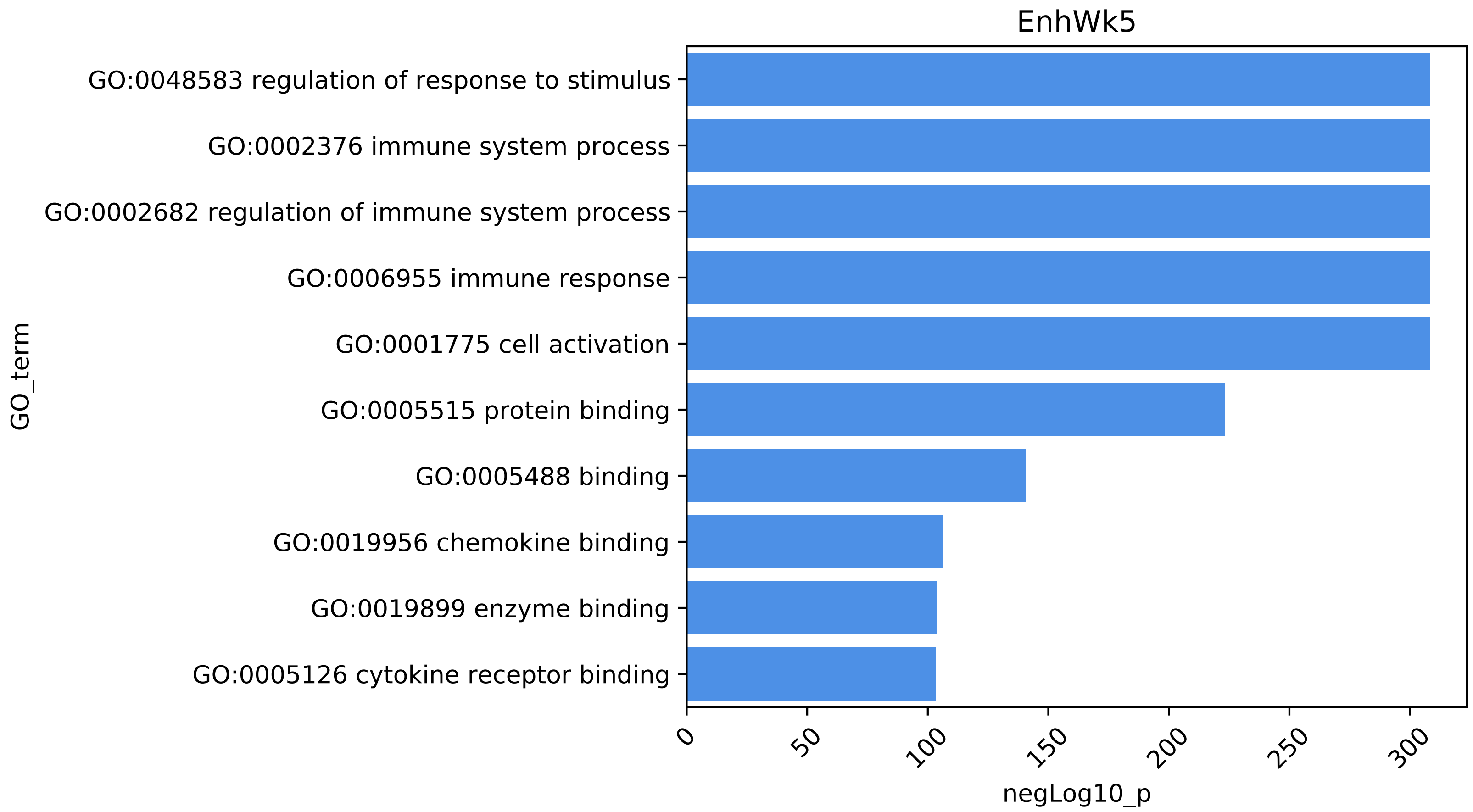

EnhWk5
GO:0048583 regulation of response to stimulus
GO:0002376 immune system process
GO:0002682 regulation of immune system process
GO:0006955 immune response
GO:0001775 cell activation
GO:0005515 protein binding
GO:0005488 binding
GO:0019956 chemokine binding
GO:0019899 enzyme binding
GO:0005126 cytokine receptor binding
GO_term
0
50
100
150
200
250
300
negLog10_p

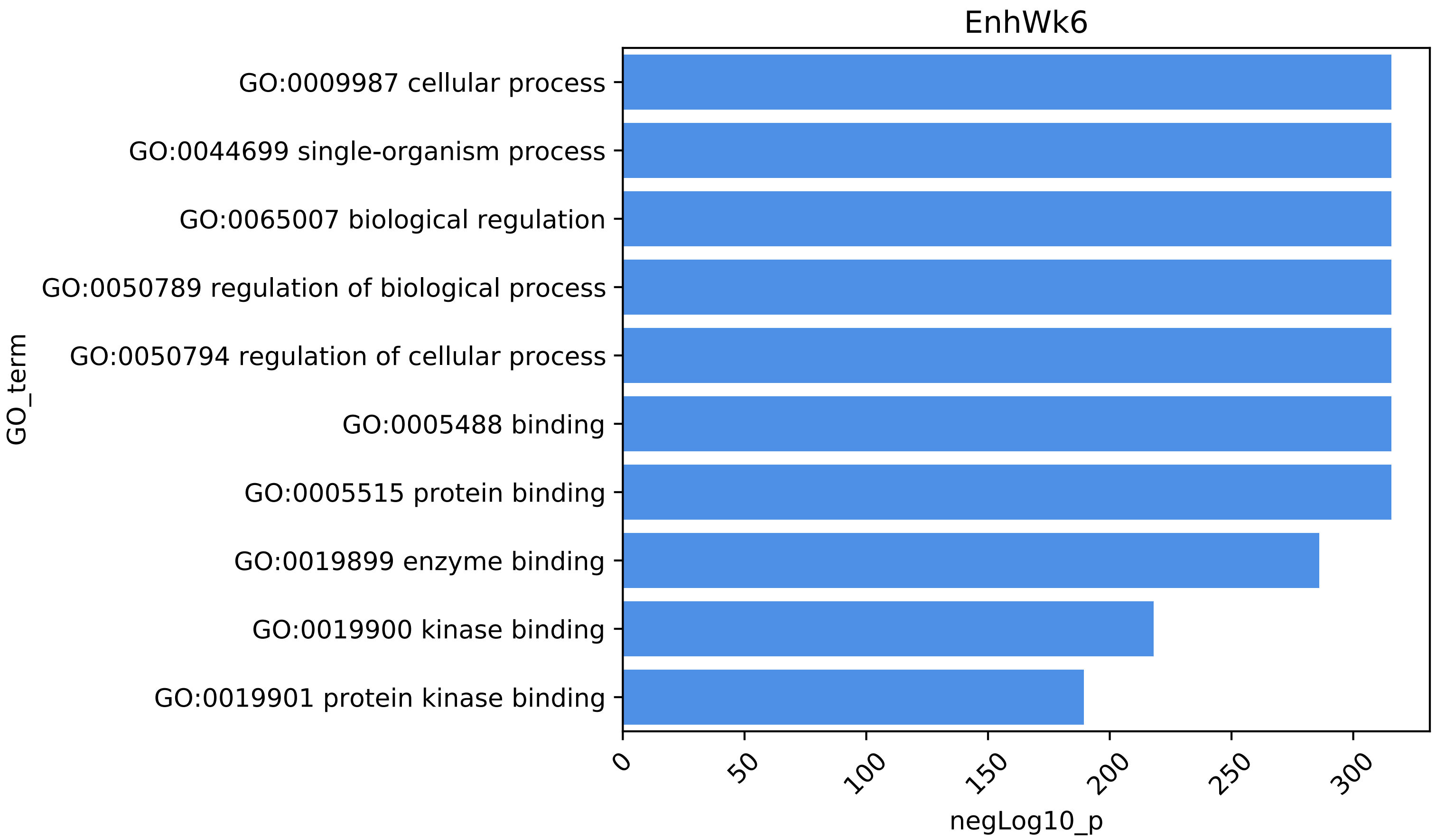

EnhWk6
GO:0009987 cellular process
GO:0044699 single-organism process
GO:0065007 biological regulation
GO:0050789 regulation of biological process
GO:0050794 regulation of cellular process
GO:0005488 binding
GO:0005515 protein binding
GO:0019899 enzyme binding
GO:0019900 kinase binding
GO:0019901 protein kinase binding
GO_term
0
50
100
150
200
250
300
negLog10_p

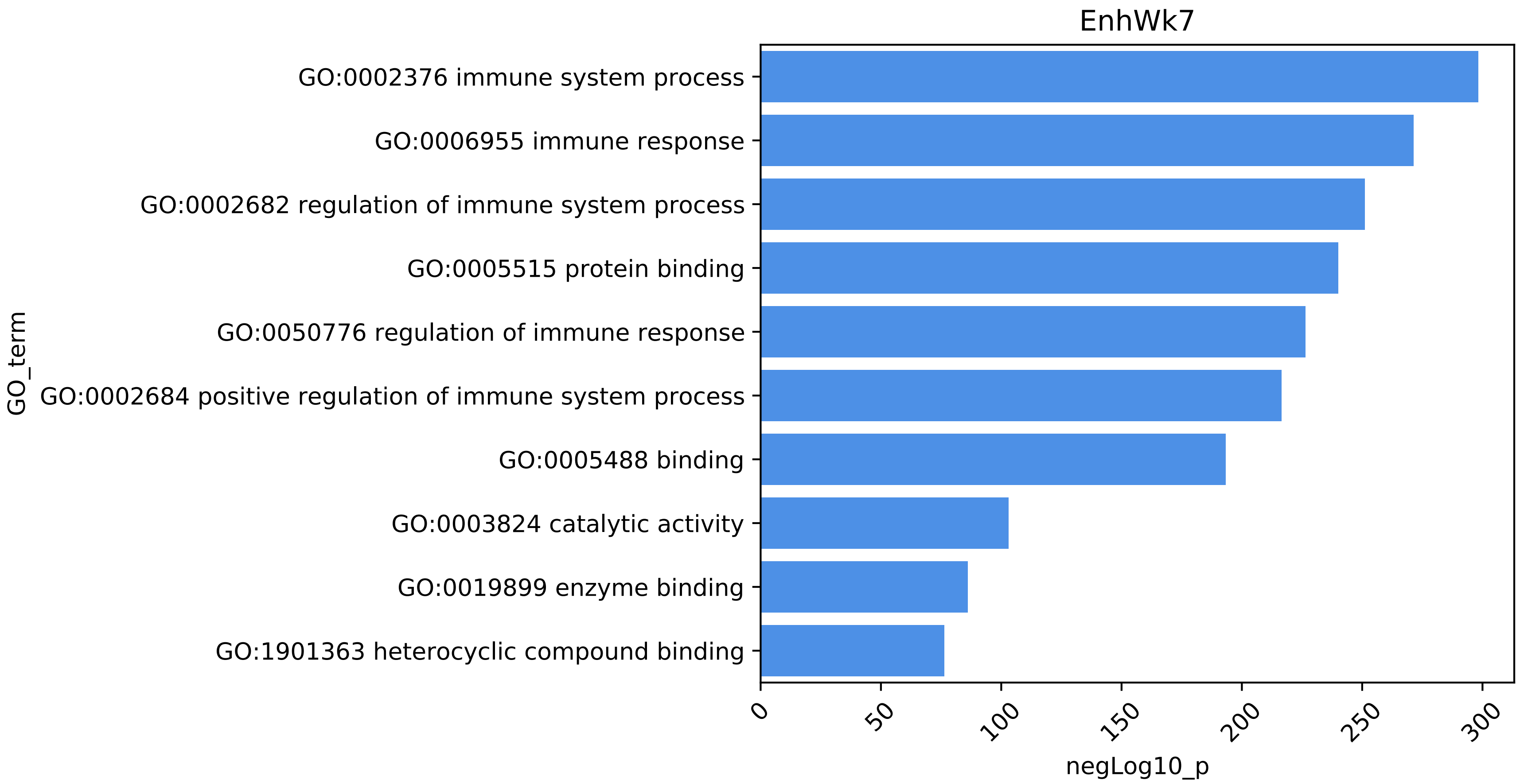

EnhWk7
GO:0002376 immune system process
GO:0006955 immune response
GO:0002682 regulation of immune system process
GO:0005515 protein binding
GO:0050776 regulation of immune response
GO:0002684 positive regulation of immune system process
GO:0005488 binding
GO:0003824 catalytic activity
GO:0019899 enzyme binding
GO:1901363 heterocyclic compound binding
GO_term
0
50
100
150
200
250
300
negLog10_p

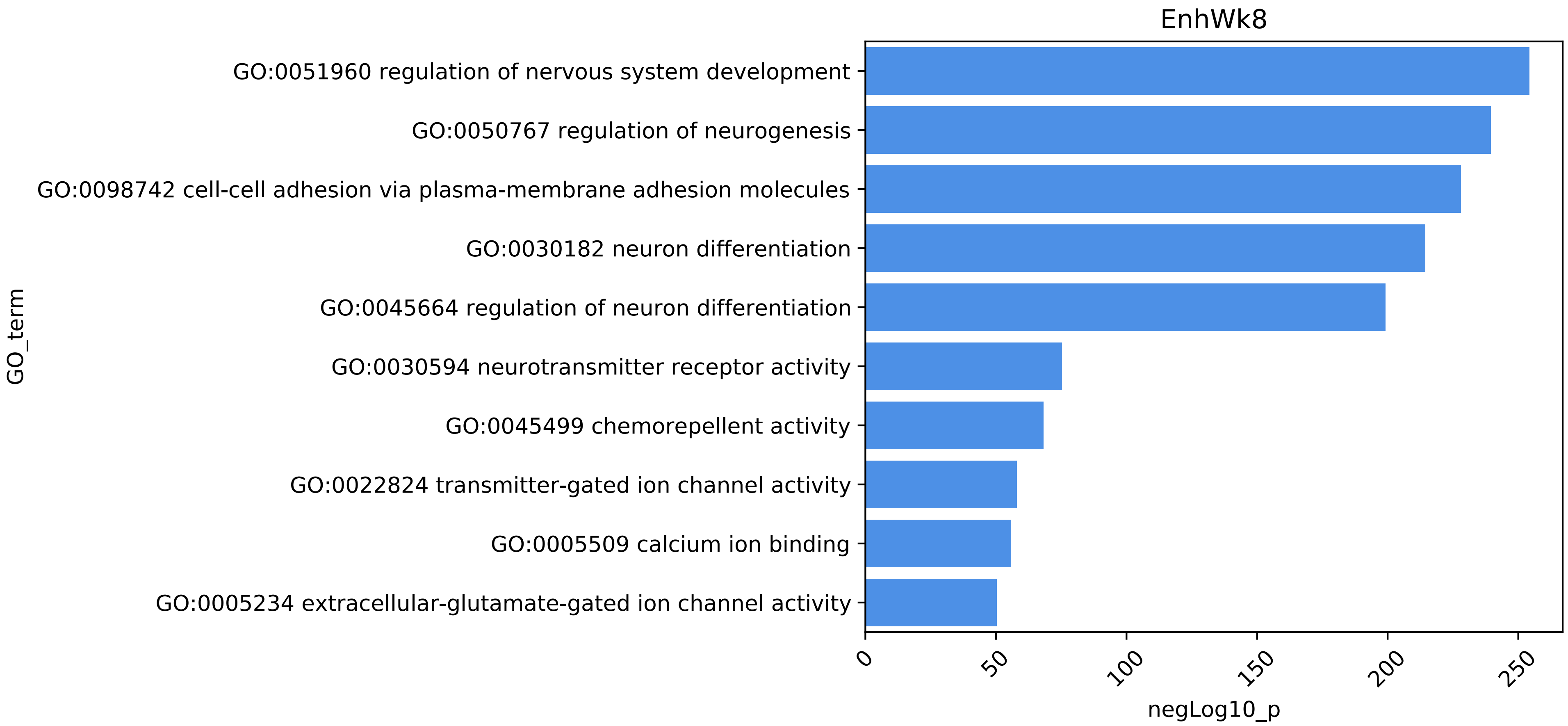
EnhWk8
GO:0051960 regulation of nervous system development
GO:0050767 regulation of neurogenesis
GO:0098742 cell-cell adhesion via plasma-membrane adhesion molecules
GO:0030182 neuron differentiation
GO:0045664 regulation of neuron differentiation
GO:0030594 neurotransmitter receptor activity
GO:0045499 chemorepellent activity
GO:0022824 transmitter-gated ion channel activity
GO:0005509 calcium ion binding
GO:0005234 extracellular-glutamate-gated ion channel activity
GO_term
0
50
100
150
200
250
negLog10_p

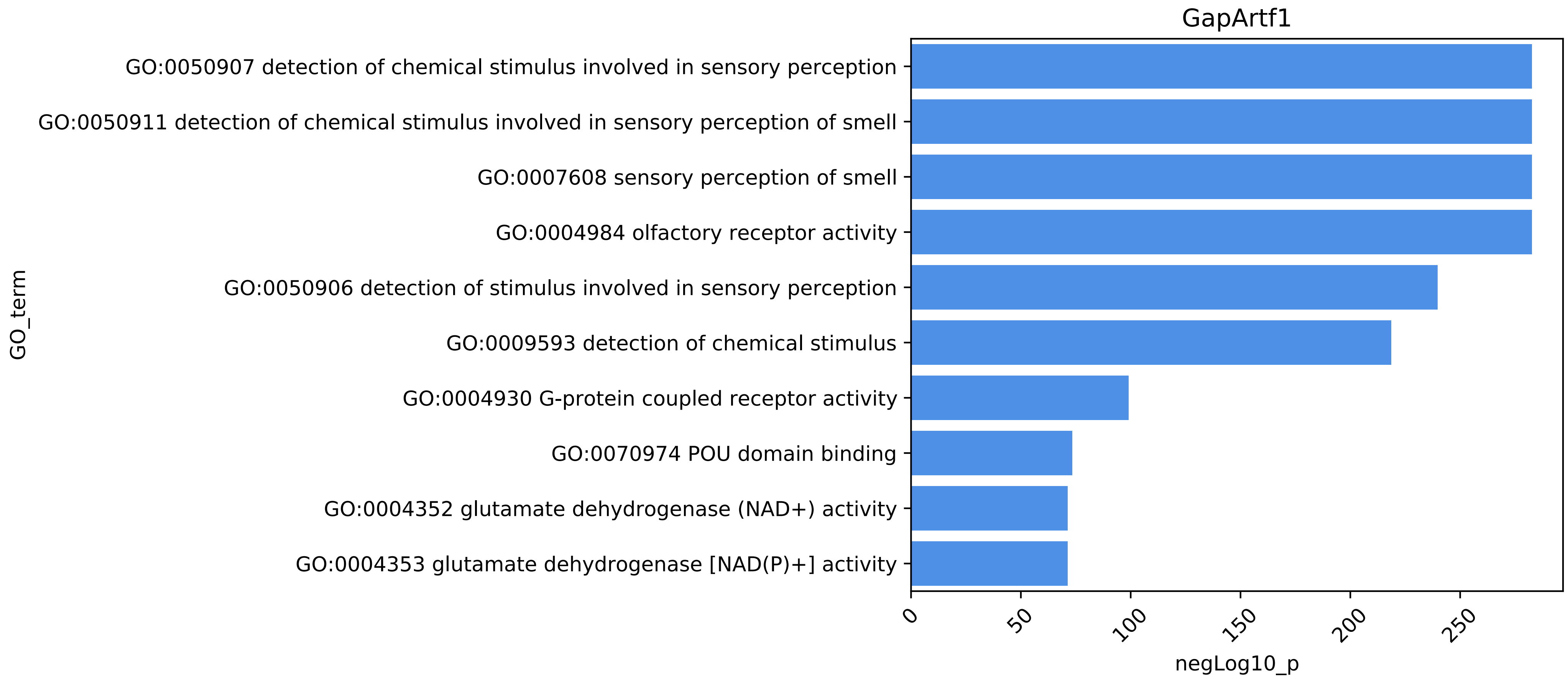
GapArtf1
GO_term
GO:0050907 detection of chemical stimulus involved in sensory perception
GO:0050911 detection of chemical stimulus involved in sensory perception of smell
GO:0007608 sensory perception of smell
GO:0004984 olfactory receptor activity
GO:0050906 detection of stimulus involved in sensory perception
GO:0009593 detection of chemical stimulus
GO:0004930 G-protein coupled receptor activity
GO:0070974 POU domain binding
GO:0004352 glutamate dehydrogenase (NAD+) activity
GO:0004353 glutamate dehydrogenase [NAD(P)+] activity
0
50
100
150
200
250
negLog10_p

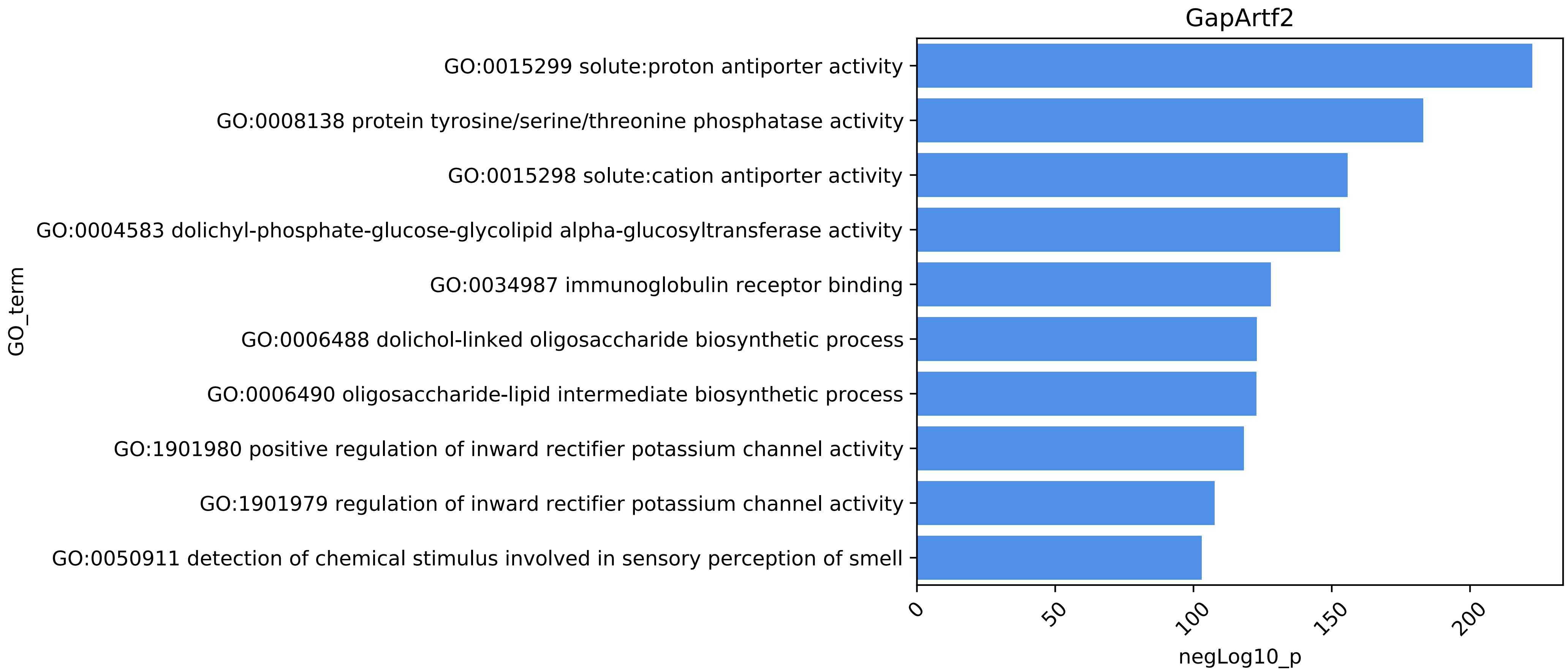
GapArtf2
GO:0015299 solute:proton antiporter activity
GO:0008138 protein tyrosine/serine/threonine phosphatase activity
GO:0015298 solute:cation antiporter activity
GO:0004583 dolichyl-phosphate-glucose-glycolipid alpha-glucosyltransferase activity
GO:0034987 immunoglobulin receptor binding
GO:0006488 dolichol-linked oligosaccharide biosynthetic process
GO:0006490 oligosaccharide-lipid intermediate biosynthetic process
GO:1901980 positive regulation of inward rectifier potassium channel activity
GO:1901979 regulation of inward rectifier potassium channel activity
GO:0050911 detection of chemical stimulus involved in sensory perception of smell
GO_term
0
50
100
150
200
negLog10_p

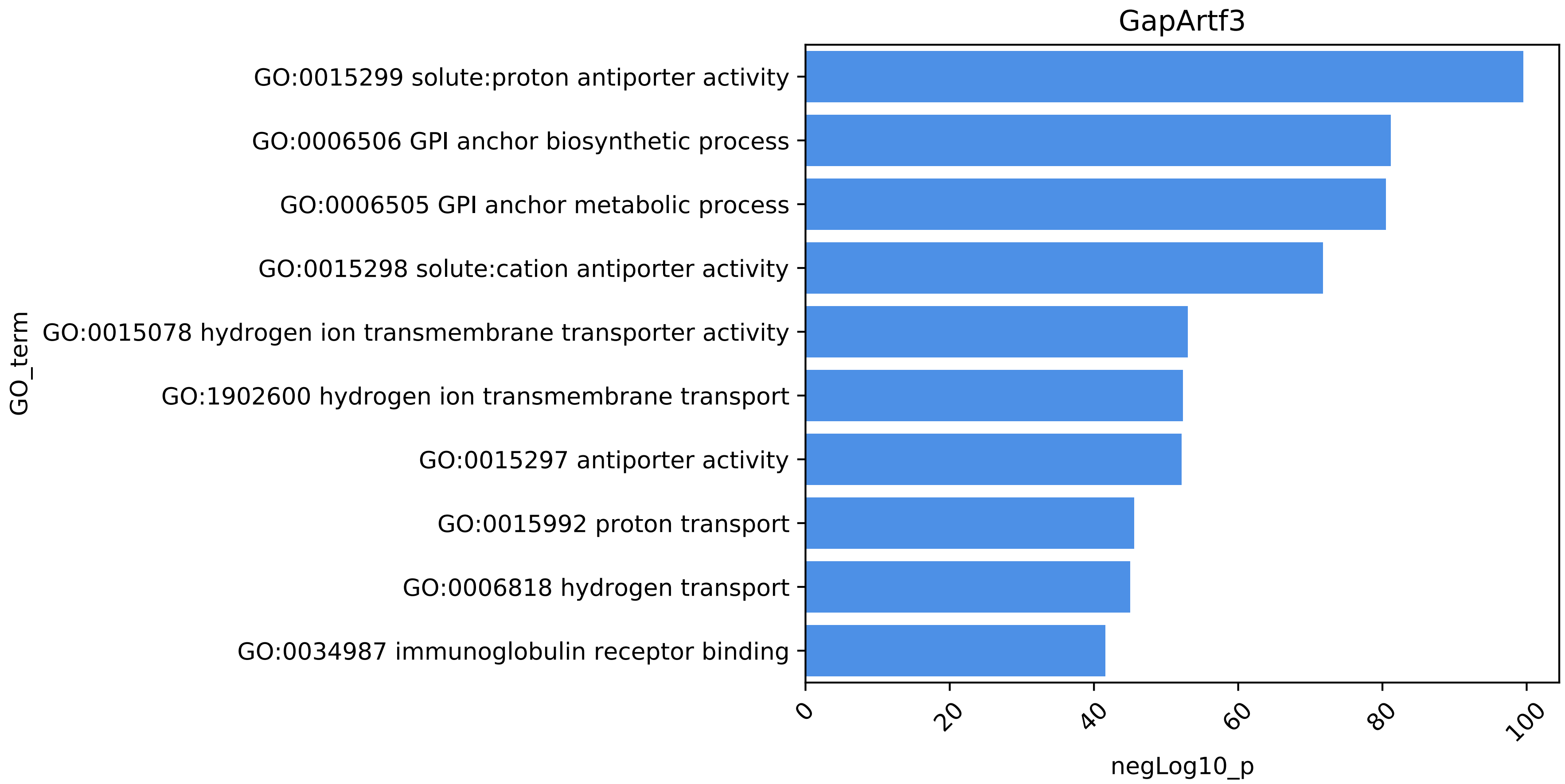
GapArtf3
GO:0015299 solute:proton antiporter activity
GO:0006506 GPI anchor biosynthetic process
GO:0006505 GPI anchor metabolic process
GO:0015298 solute:cation antiporter activity
GO:0015078 hydrogen ion transmembrane transporter activity
GO:1902600 hydrogen ion transmembrane transport
GO:0015297 antiporter activity
GO:0015992 proton transport
GO:0006818 hydrogen transport
GO:0034987 immunoglobulin receptor binding
GO_term
0
20
40
60
80
100
negLog10_p

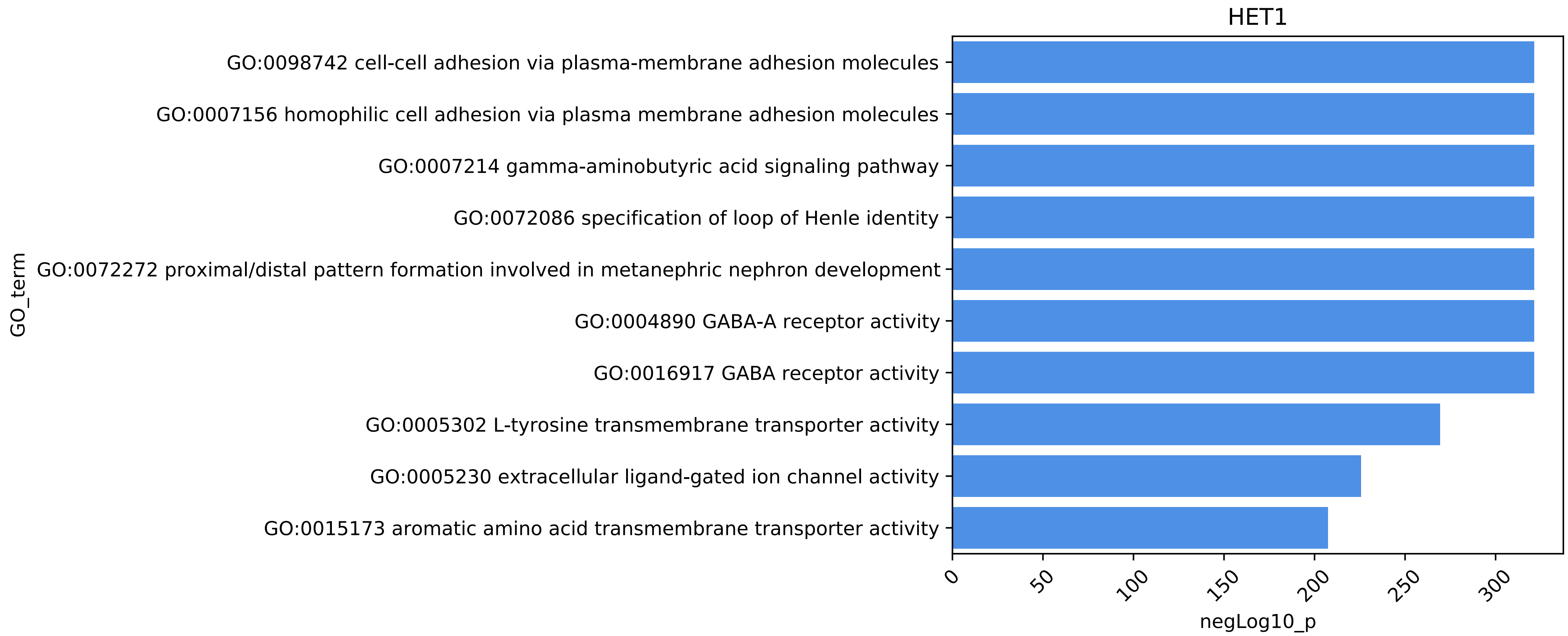
HET1
GO:0098742 cell-cell adhesion via plasma-membrane adhesion molecules
GO:0007156 homophilic cell adhesion via plasma membrane adhesion molecules
GO:0007214 gamma-aminobutyric acid signaling pathway
GO:0072086 specification of loop of Henle identity
GO:0072272 proximal/distal pattern formation involved in metanephric nephron development
GO:0004890 GABA-A receptor activity
GO:0016917 GABA receptor activity
GO:0005302 L-tyrosine transmembrane transporter activity
GO:0005230 extracellular ligand-gated ion channel activity
GO:0015173 aromatic amino acid transmembrane transporter activity
GO_term
0
50
100
150
200
250
300
negLog10_p

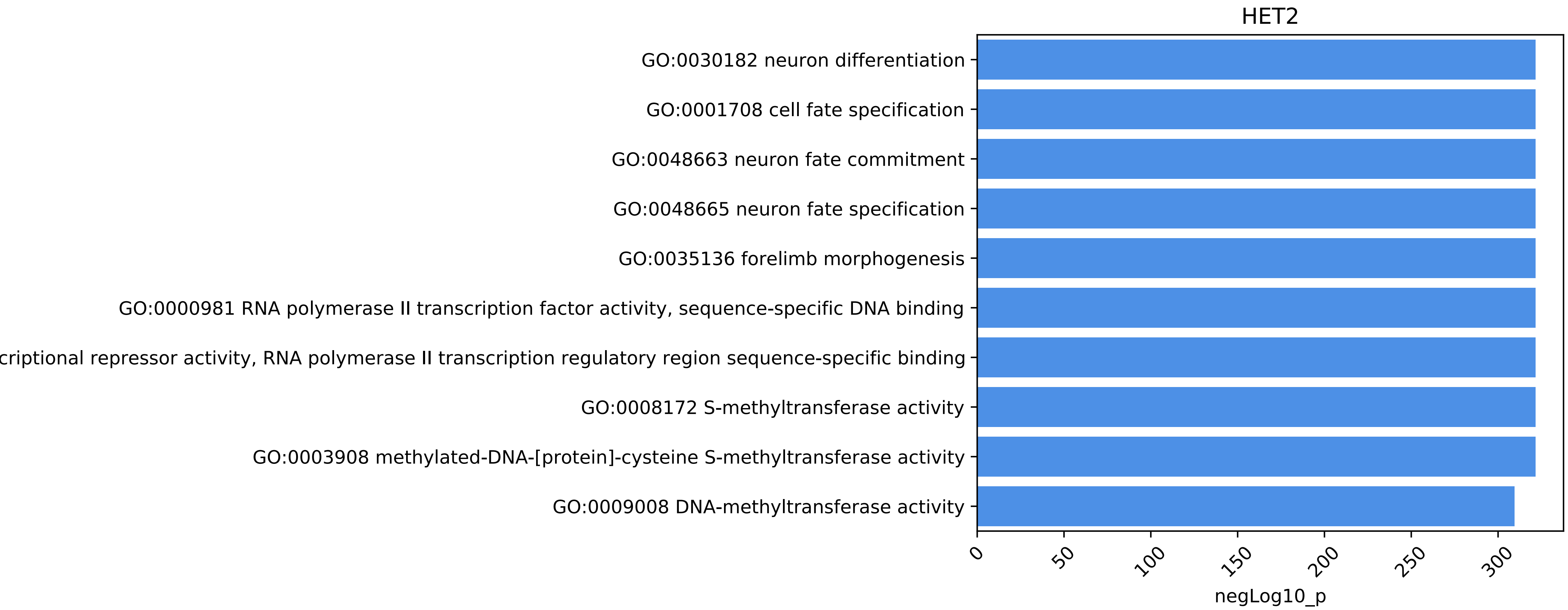
HET2
GO:0030182 neuron differentiation
GO:0001708 cell fate specification
GO:0048663 neuron fate commitment
GO:0048665 neuron fate specification
GO:0035136 forelimb morphogenesis
GO:0000981 RNA polymerase II transcription factor activity, sequence-specific DNA binding
criptional repressor activity, RNA polymerase II transcription regulatory region sequence-specific binding
GO:0008172 S-methyltransferase activity
GO:0003908 methylated-DNA-[protein]-cysteine S-methyltransferase activity
GO:0009008 DNA-methyltransferase activity
0
50
100
150
200
250
300
negLog10_p

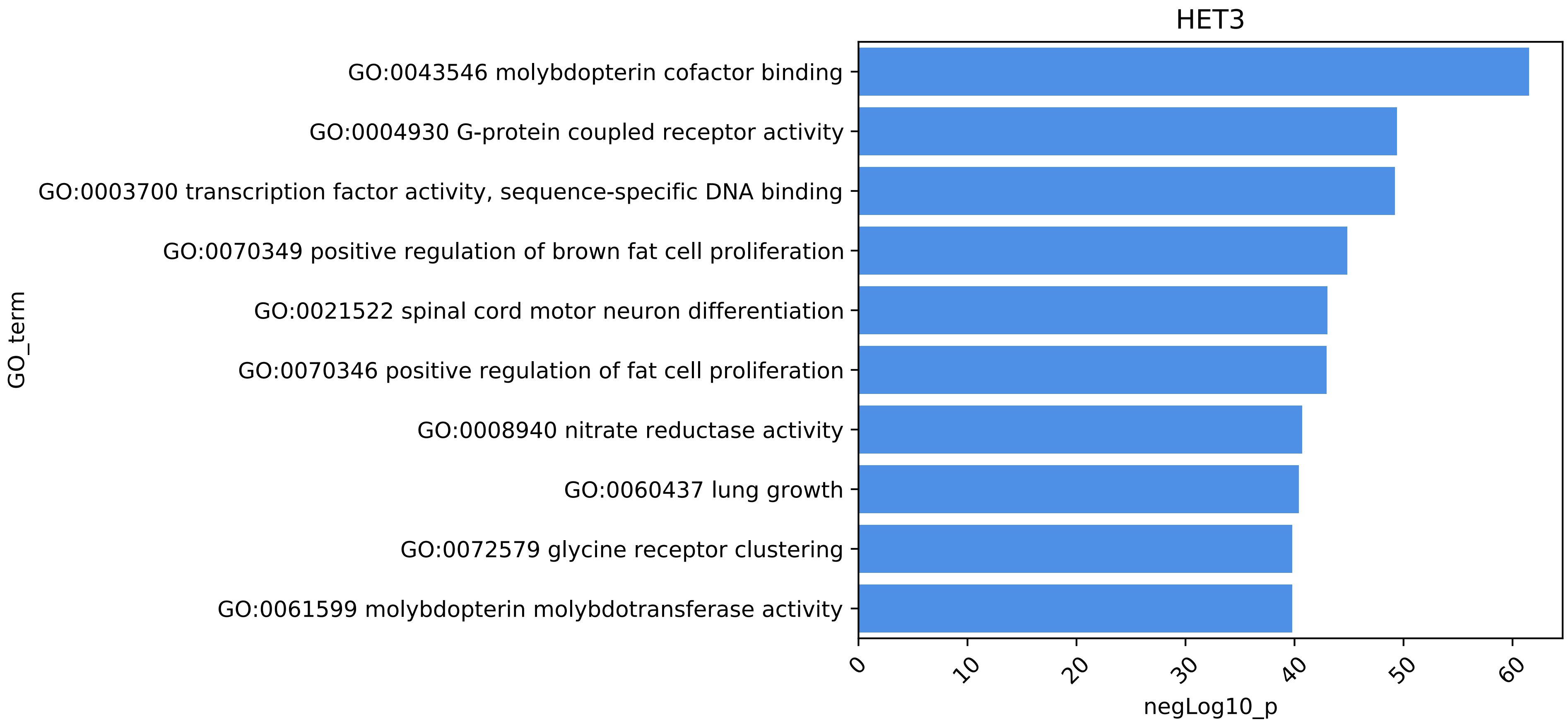
HET3
GO:0043546 molybdopterin cofactor binding
GO:0004930 G-protein coupled receptor activity
GO:0003700 transcription factor activity, sequence-specific DNA binding
GO:0070349 positive regulation of brown fat cell proliferation
GO:0021522 spinal cord motor neuron differentiation
GO:0070346 positive regulation of fat cell proliferation
GO:0008940 nitrate reductase activity
GO:0060437 lung growth
GO:0072579 glycine receptor clustering
GO:0061599 molybdopterin molybdotransferase activity
GO_term
0
10
20
30
40
50
60
negLog10_p

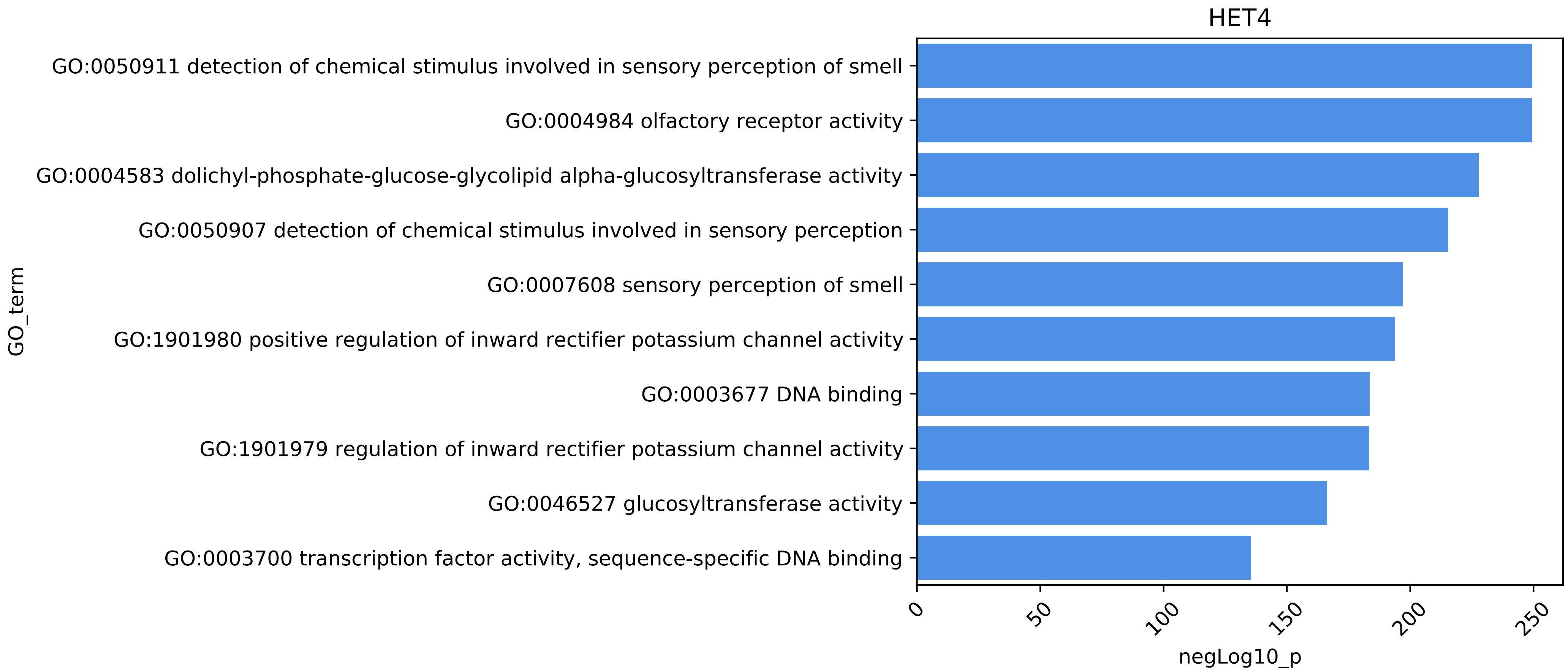
HET4
GO:0050911 detection of chemical stimulus involved in sensory perception of smell
GO:0004984 olfactory receptor activity
GO:0004583 dolichyl-phosphate-glucose-glycolipid alpha-glucosyltransferase activity
GO:0050907 detection of chemical stimulus involved in sensory perception
GO:0007608 sensory perception of smell
GO:1901980 positive regulation of inward rectifier potassium channel activity
GO:0003677 DNA binding
GO:1901979 regulation of inward rectifier potassium channel activity
GO:0046527 glucosyltransferase activity
GO:0003700 transcription factor activity, sequence-specific DNA binding
GO_term
0
50
100
150
200
250
negLog10_p

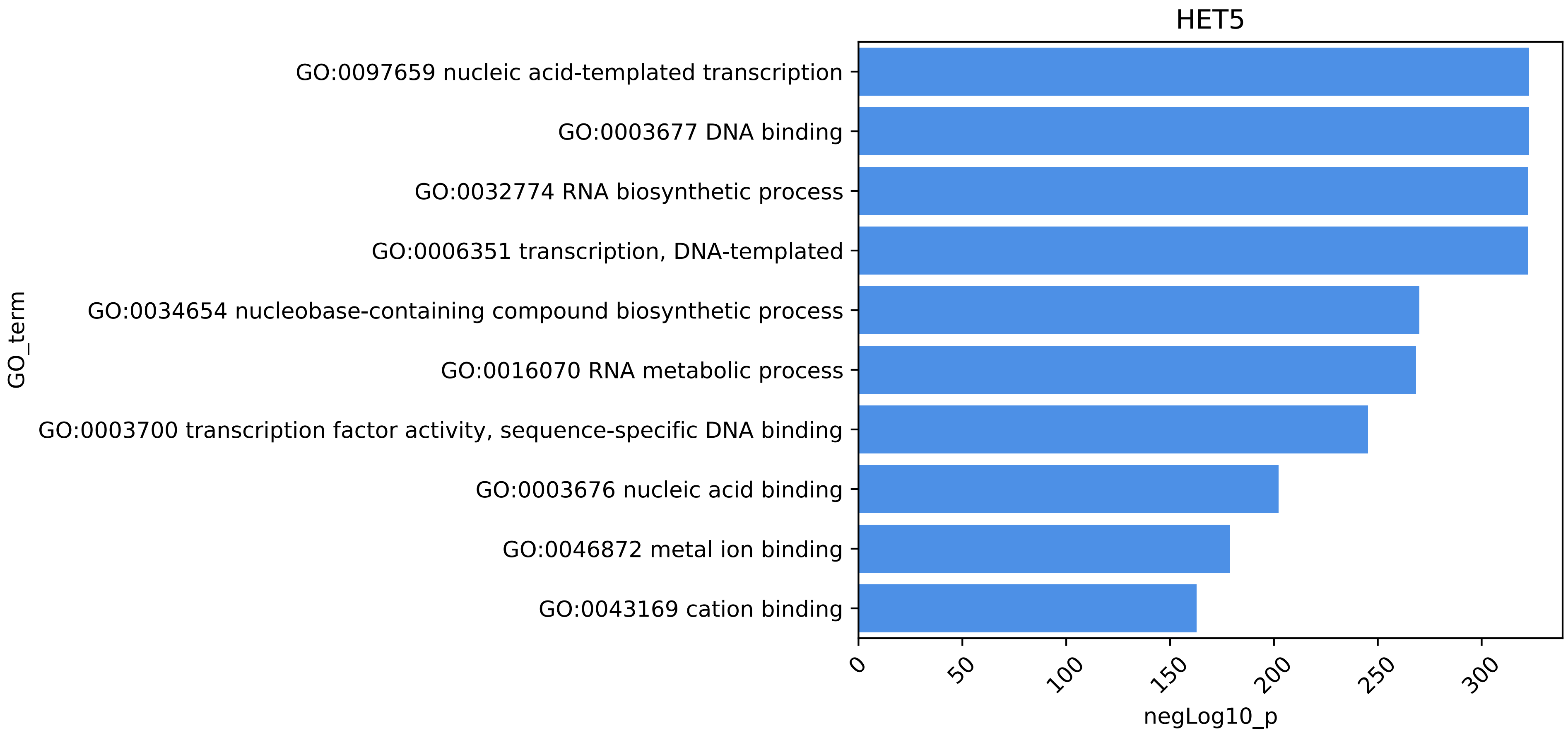
HET5
GO:0097659 nucleic acid-templated transcription
GO:0003677 DNA binding
GO:0032774 RNA biosynthetic process
GO:0006351 transcription, DNA-templated
GO:0034654 nucleobase-containing compound biosynthetic process
GO:0016070 RNA metabolic process
GO:0003700 transcription factor activity, sequence-specific DNA binding
GO:0003676 nucleic acid binding
GO:0046872 metal ion binding
GO:0043169 cation binding
GO_term
0
50
100
150
200
250
300
negLog10_p

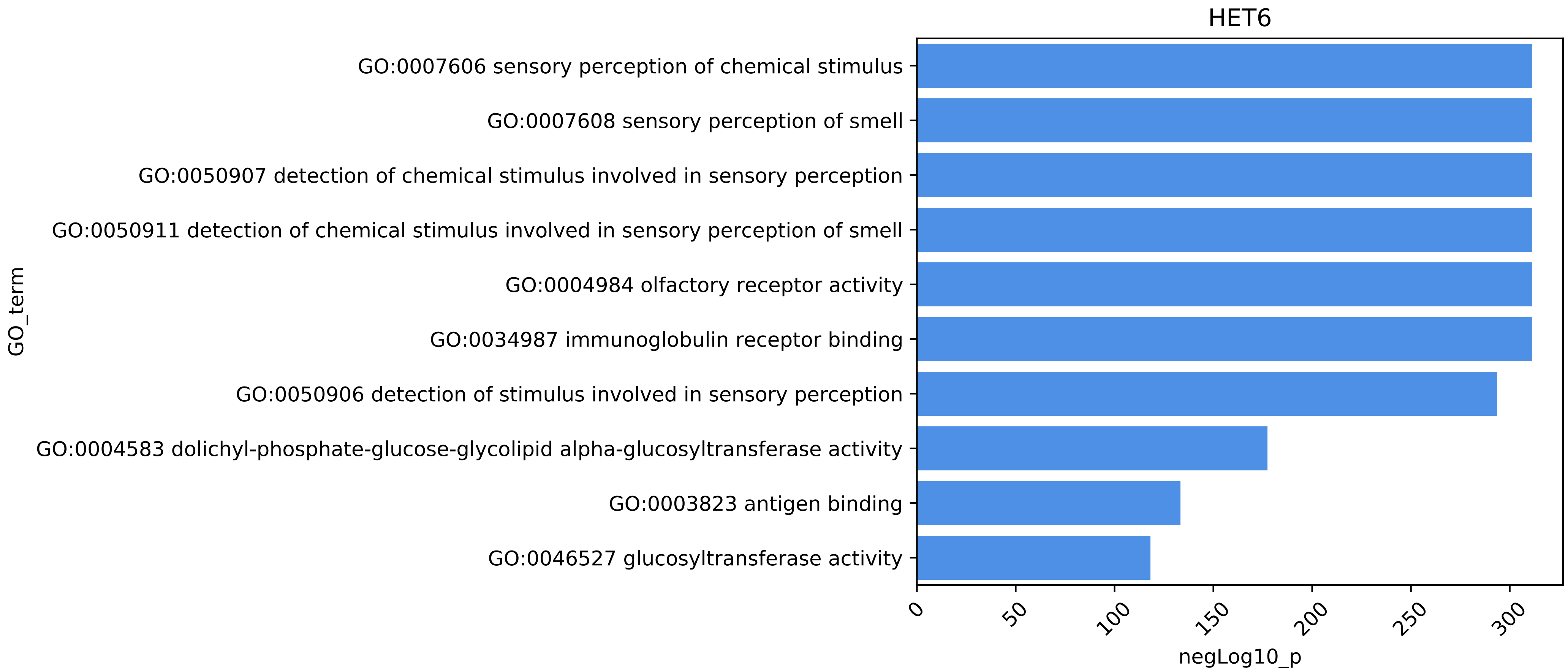

HET6
GO:0007606 sensory perception of chemical stimulus
GO:0007608 sensory perception of smell
GO:0050907 detection of chemical stimulus involved in sensory perception
GO:0050911 detection of chemical stimulus involved in sensory perception of smell
GO:0004984 olfactory receptor activity
GO:0034987 immunoglobulin receptor binding
GO:0050906 detection of stimulus involved in sensory perception
GO:0004583 dolichyl-phosphate-glucose-glycolipid alpha-glucosyltransferase activity
GO:0003823 antigen binding
GO:0046527 glucosyltransferase activity
GO_term
0
50
100
150
200
250
300
negLog10_p

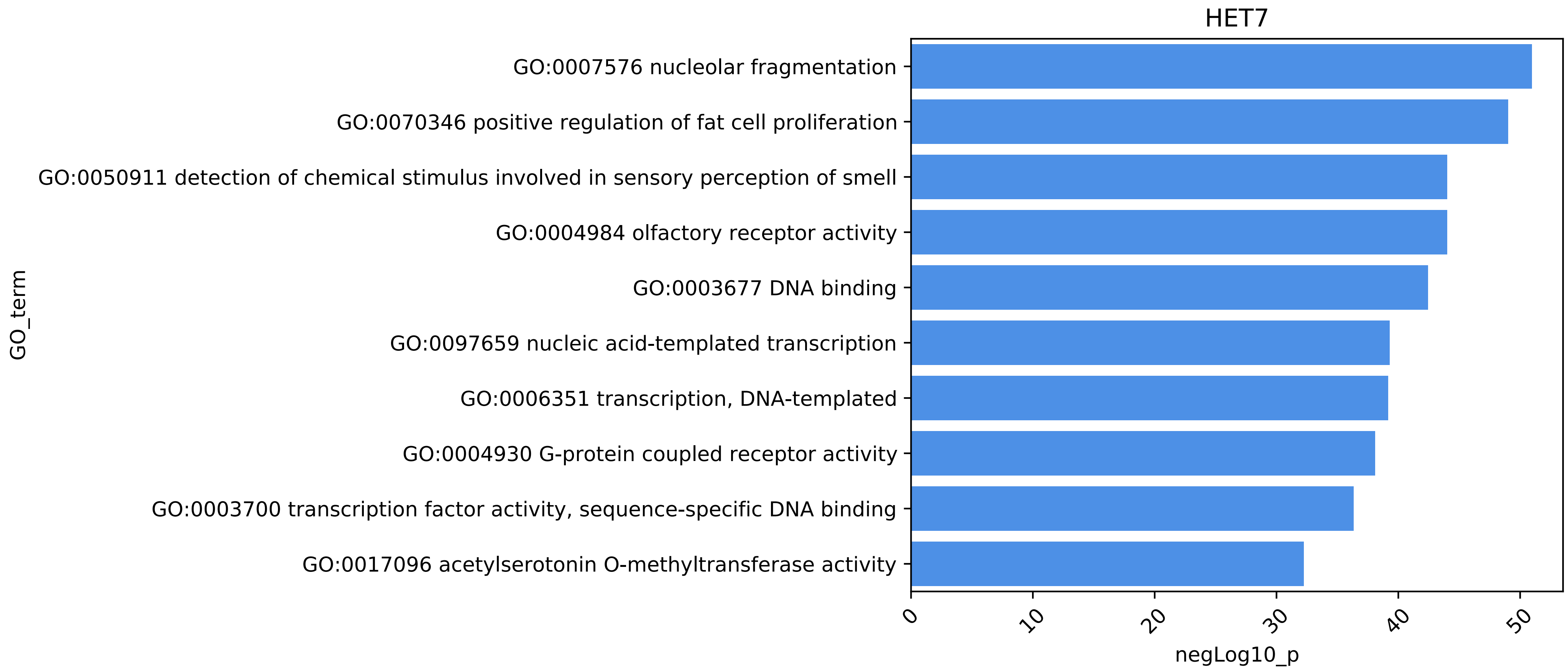
HET7
GO:0007576 nucleolar fragmentation
GO:0070346 positive regulation of fat cell proliferation
GO:0050911 detection of chemical stimulus involved in sensory perception of smell
GO:0004984 olfactory receptor activity
GO:0003677 DNA binding
GO:0097659 nucleic acid-templated transcription
GO:0006351 transcription, DNA-templated
GO:0004930 G-protein coupled receptor activity
GO:0003700 transcription factor activity, sequence-specific DNA binding
GO:0017096 acetylserotonin O-methyltransferase activity
GO_term
0
10
20
30
40
50
negLog10_p

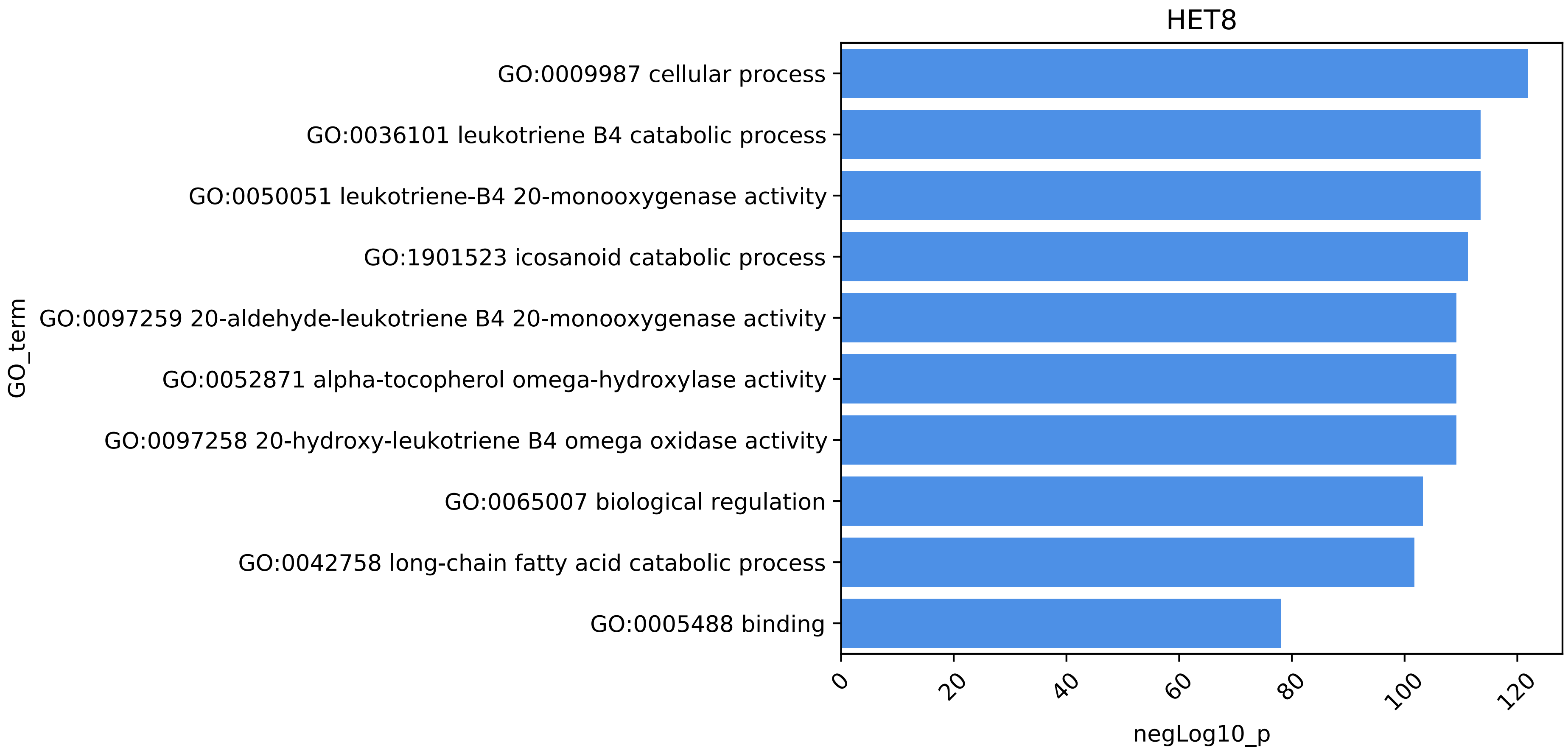
HET8
GO_term
GO:0009987 cellular process
GO:0036101 leukotriene B4 catabolic process
GO:0050051 leukotriene-B4 20-monooxygenase activity
GO:1901523 icosanoid catabolic process
GO:0097259 20-aldehyde-leukotriene B4 20-monooxygenase activity
GO:0052871 alpha-tocopherol omega-hydroxylase activity
GO:0097258 20-hydroxy-leukotriene B4 omega oxidase activity
GO:0065007 biological regulation
GO:0042758 long-chain fatty acid catabolic process
GO:0005488 binding
0
20
40
60
80
100
120
negLog10_p

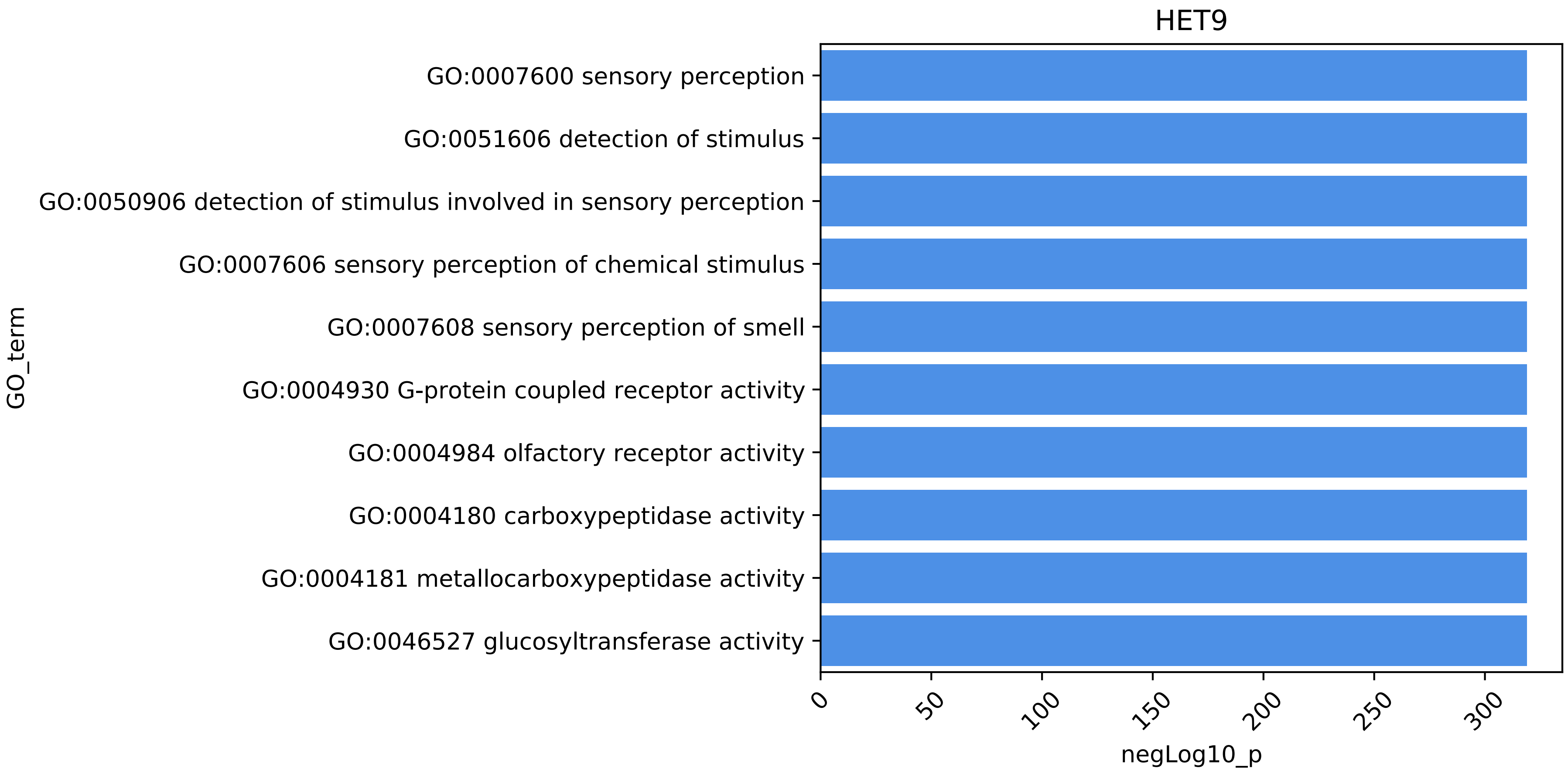

HET9
GO:0007600 sensory perception
GO:0051606 detection of stimulus
GO:0050906 detection of stimulus involved in sensory perception
GO:0007606 sensory perception of chemical stimulus
GO:0007608 sensory perception of smell
GO:0004930 G-protein coupled receptor activity
GO:0004984 olfactory receptor activity
GO:0004180 carboxypeptidase activity
GO:0004181 metallocarboxypeptidase activity
GO:0046527 glucosyltransferase activity
GO_term
0
50
100
150
200
250
300
negLog10_p

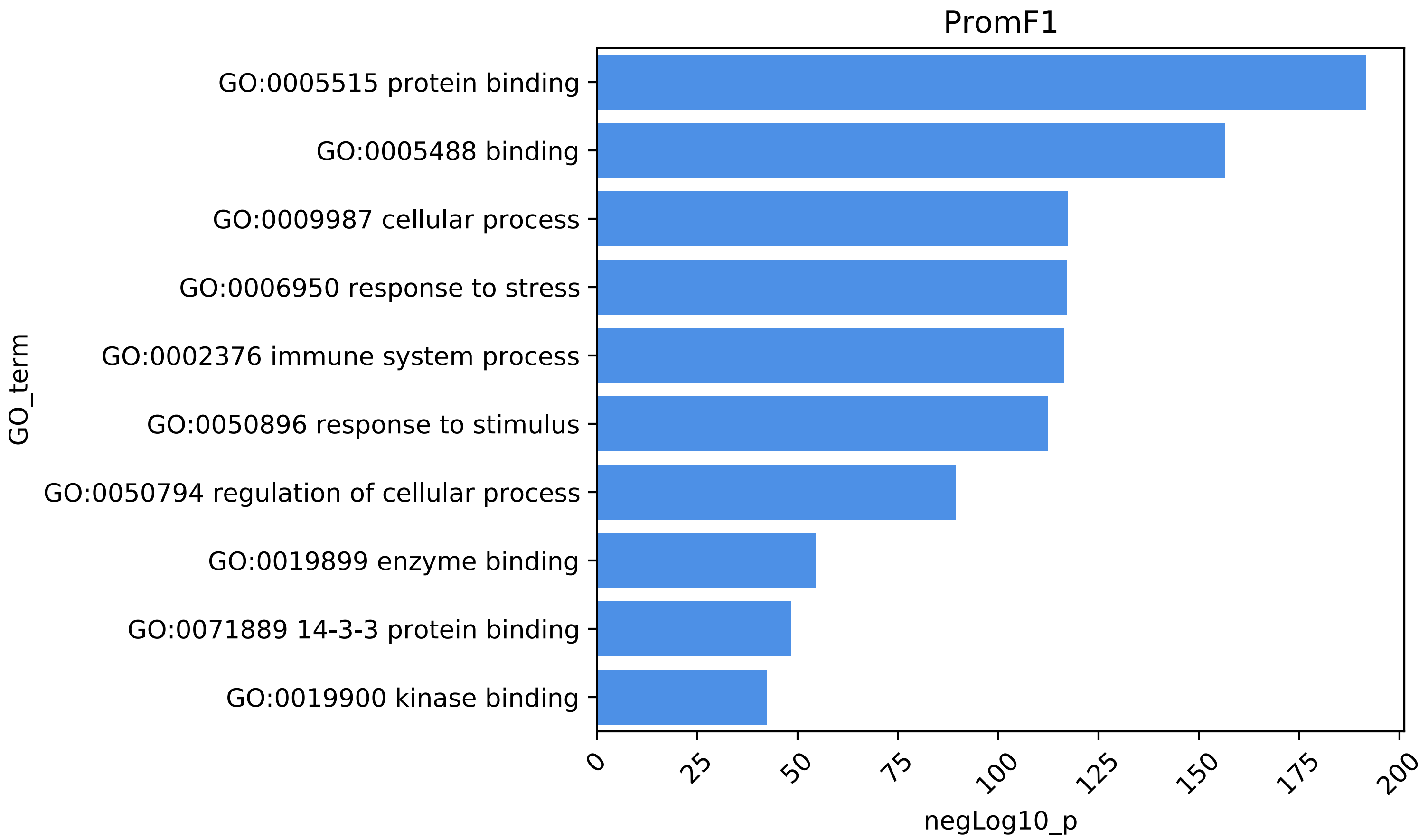
PromF1
GO:0005515 protein binding
GO:0005488 binding
GO:0009987 cellular process
GO:0006950 response to stress
GO:0002376 immune system process
GO:0050896 response to stimulus
GO:0050794 regulation of cellular process
GO:0019899 enzyme binding
GO:0071889 14-3-3 protein binding
GO:0019900 kinase binding
GO_term
0
25
50
75
100
125
150
175
200
negLog10_p

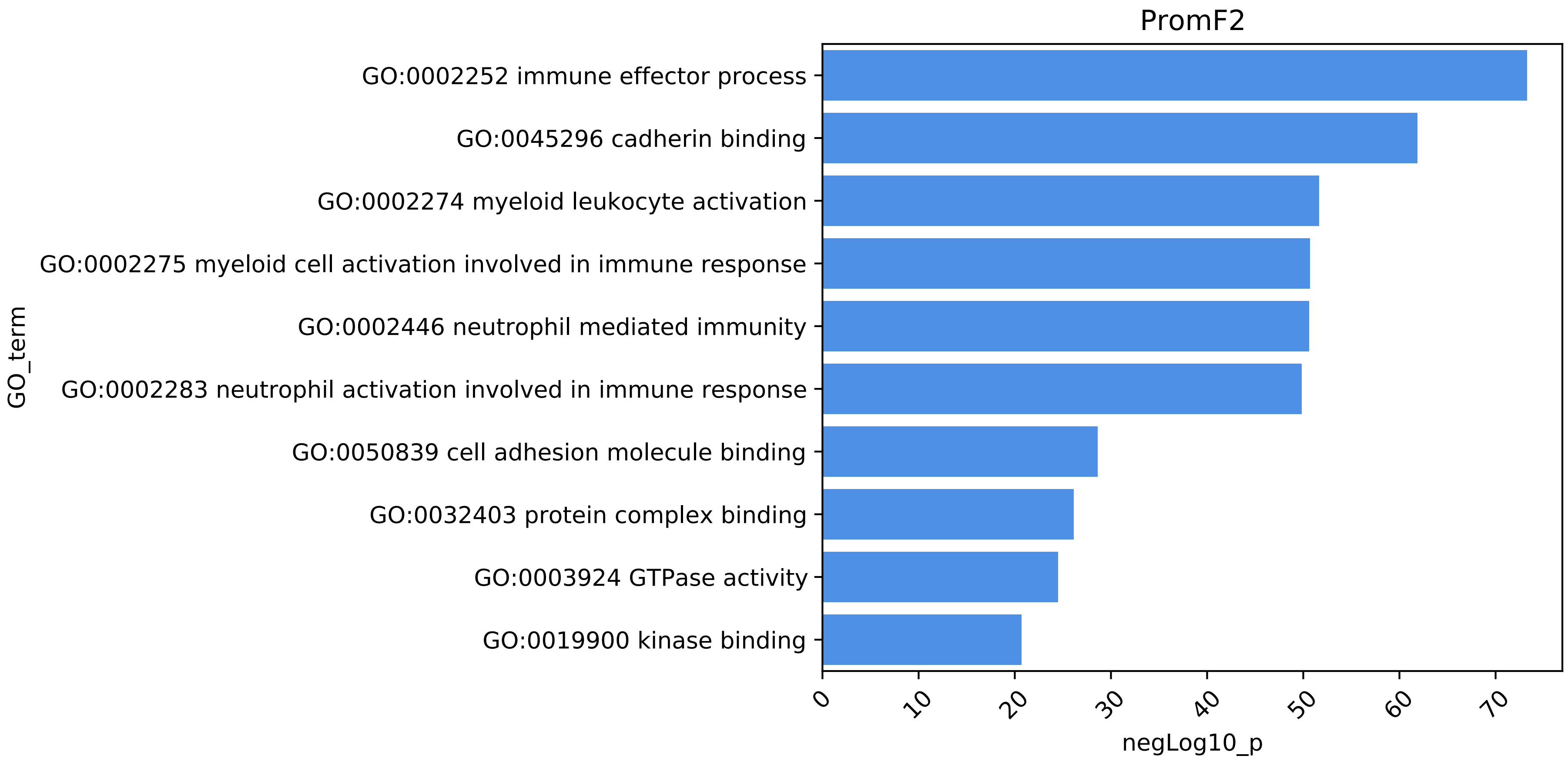
PromF2
GO_term
GO:0002252 immune effector process
GO:0045296 cadherin binding
GO:0002274 myeloid leukocyte activation
GO:0002275 myeloid cell activation involved in immune response
GO:0002446 neutrophil mediated immunity
GO:0002283 neutrophil activation involved in immune response
GO:0050839 cell adhesion molecule binding
GO:0032403 protein complex binding
GO:0003924 GTPase activity
GO:0019900 kinase binding
0
10
20
30
40
50
60
70
negLog10_p

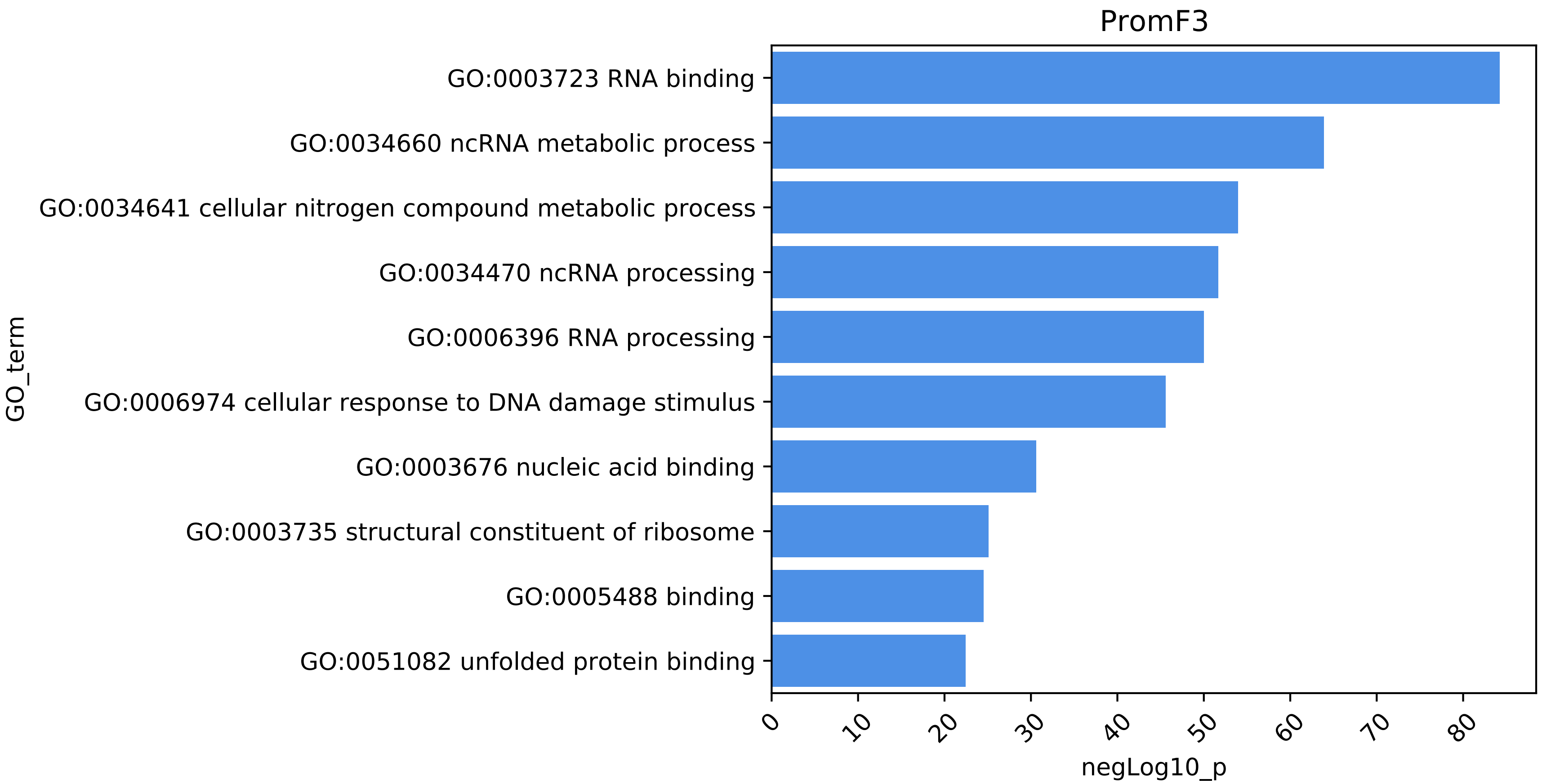

PromF3
GO:0003723 RNA binding
GO:0034660 ncRNA metabolic process
GO:0034641 cellular nitrogen compound metabolic process
GO:0034470 ncRNA processing
GO:0006396 RNA processing
GO:0006974 cellular response to DNA damage stimulus
GO:0003676 nucleic acid binding
GO:0003735 structural constituent of ribosome
GO:0005488 binding
GO:0051082 unfolded protein binding
GO_term
0
10
20
30
40
50
60
70
80
negLog10_p

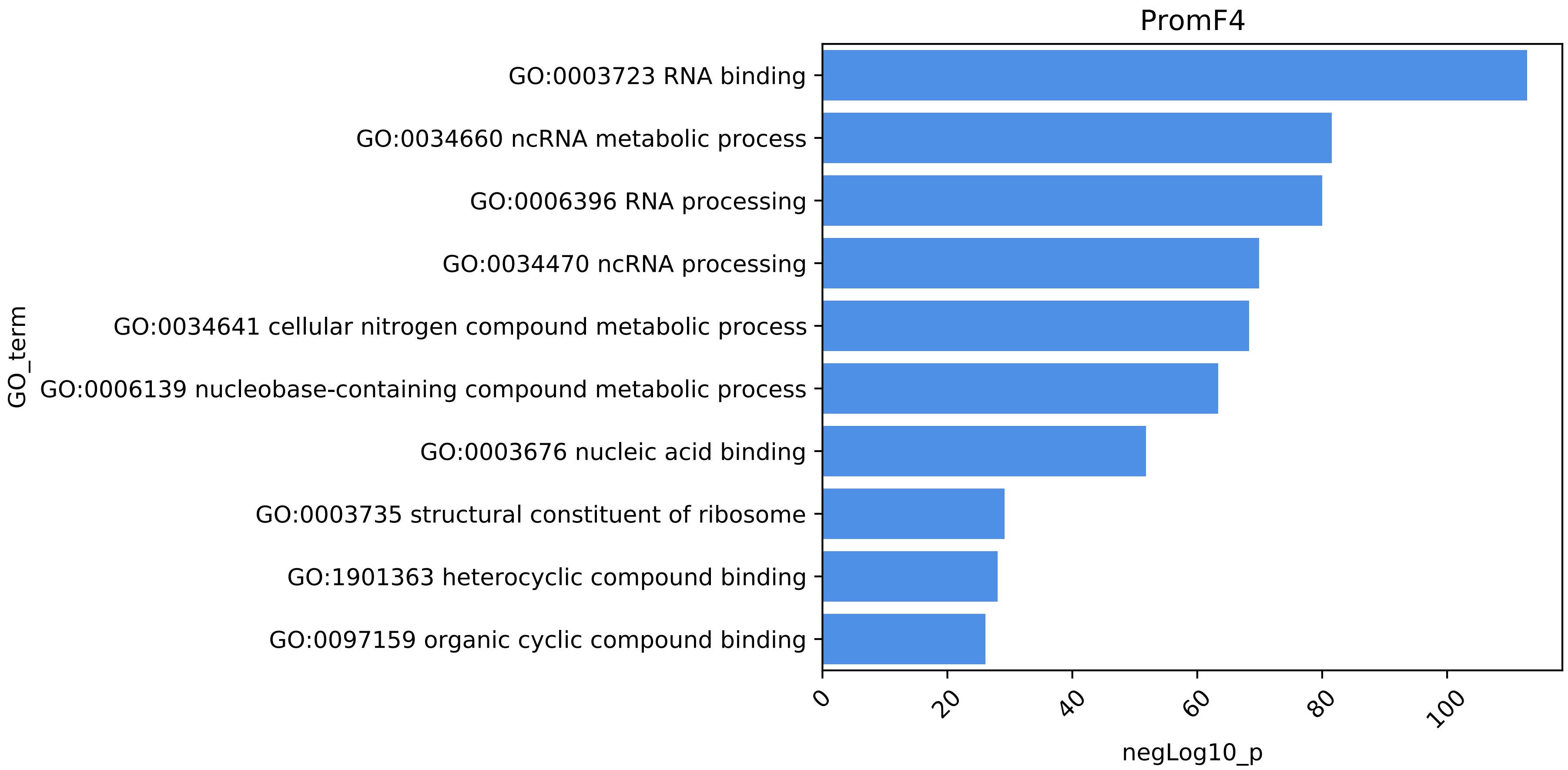
PromF4
GO:0003723 RNA binding
GO:0034660 ncRNA metabolic process
GO:0006396 RNA processing
GO:0034470 ncRNA processing
GO:0034641 cellular nitrogen compound metabolic process
GO:0006139 nucleobase-containing compound metabolic process
GO:0003676 nucleic acid binding
GO:0003735 structural constituent of ribosome
GO:1901363 heterocyclic compound binding
GO:0097159 organic cyclic compound binding
GO_term
0
20
40
60
80
100
negLog10_p

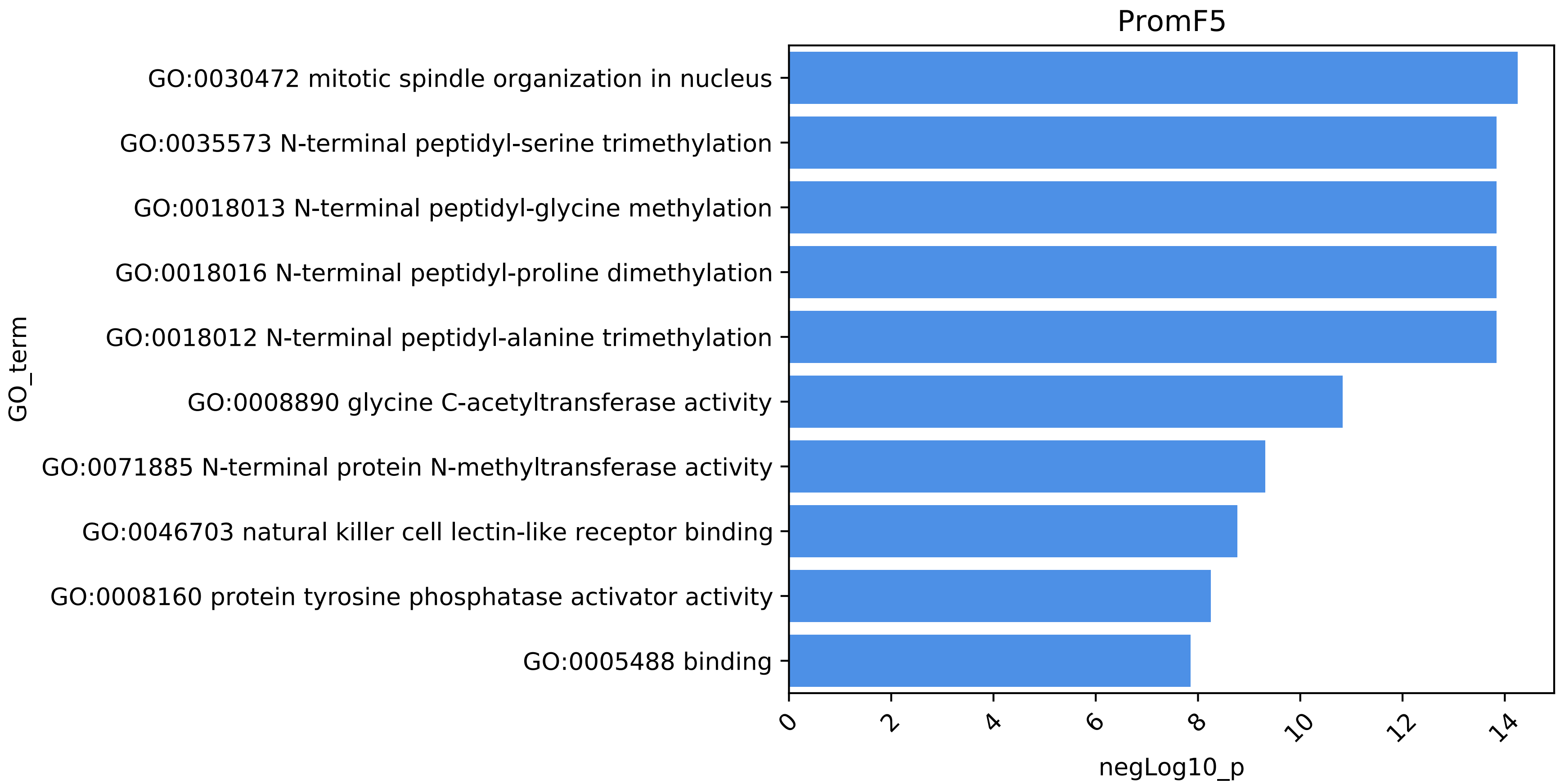
PromF5
GO:0030472 mitotic spindle organization in nucleus
GO:0035573 N-terminal peptidyl-serine trimethylation
GO:0018013 N-terminal peptidyl-glycine methylation
GO:0018016 N-terminal peptidyl-proline dimethylation
GO:0018012 N-terminal peptidyl-alanine trimethylation
GO:0008890 glycine C-acetyltransferase activity
GO:0071885 N-terminal protein N-methyltransferase activity
GO:0046703 natural killer cell lectin-like receptor binding
GO:0008160 protein tyrosine phosphatase activator activity
GO:0005488 binding
GO_term
0
2
4
6
8
10
12
14
negLog10_p

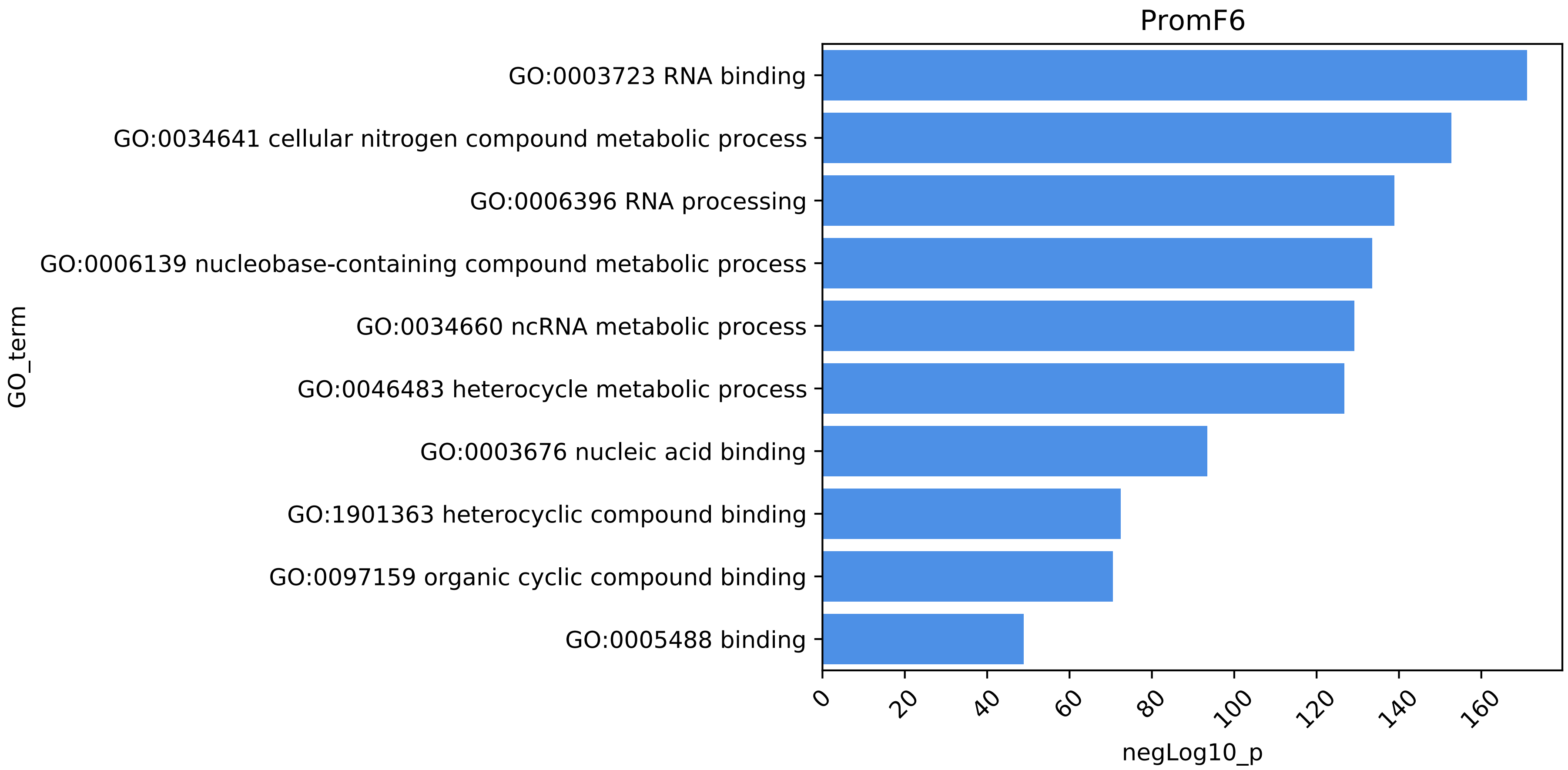
PromF6
GO:0003723 RNA binding
GO:0034641 cellular nitrogen compound metabolic process
GO:0006396 RNA processing
GO:0006139 nucleobase-containing compound metabolic process
GO:0034660 ncRNA metabolic process
GO:0046483 heterocycle metabolic process
GO:0003676 nucleic acid binding
GO:1901363 heterocyclic compound binding
GO:0097159 organic cyclic compound binding
GO:0005488 binding
GO_term
0
20
40
60
80
100
120
140
160
negLog10_p

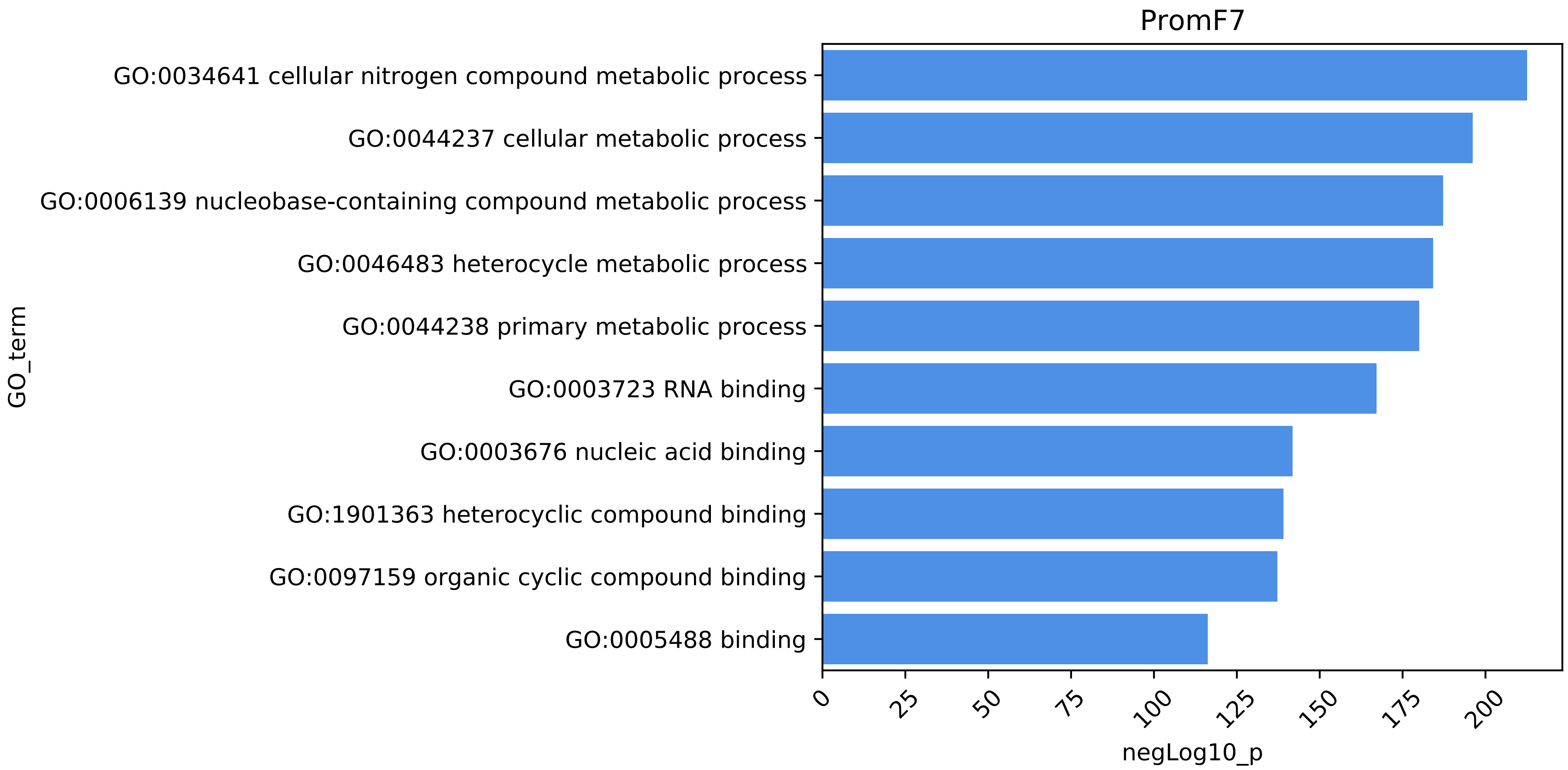
PromF7
GO:0034641 cellular nitrogen compound metabolic process
GO:0044237 cellular metabolic process
GO:0006139 nucleobase-containing compound metabolic process
GO:0046483 heterocycle metabolic process
GO:0044238 primary metabolic process
GO:0003723 RNA binding
GO:0003676 nucleic acid binding
GO:1901363 heterocyclic compound binding
GO:0097159 organic cyclic compound binding
GO:0005488 binding
GO_term
0
25
50
75
100
125
150
175
200
negLog10_p

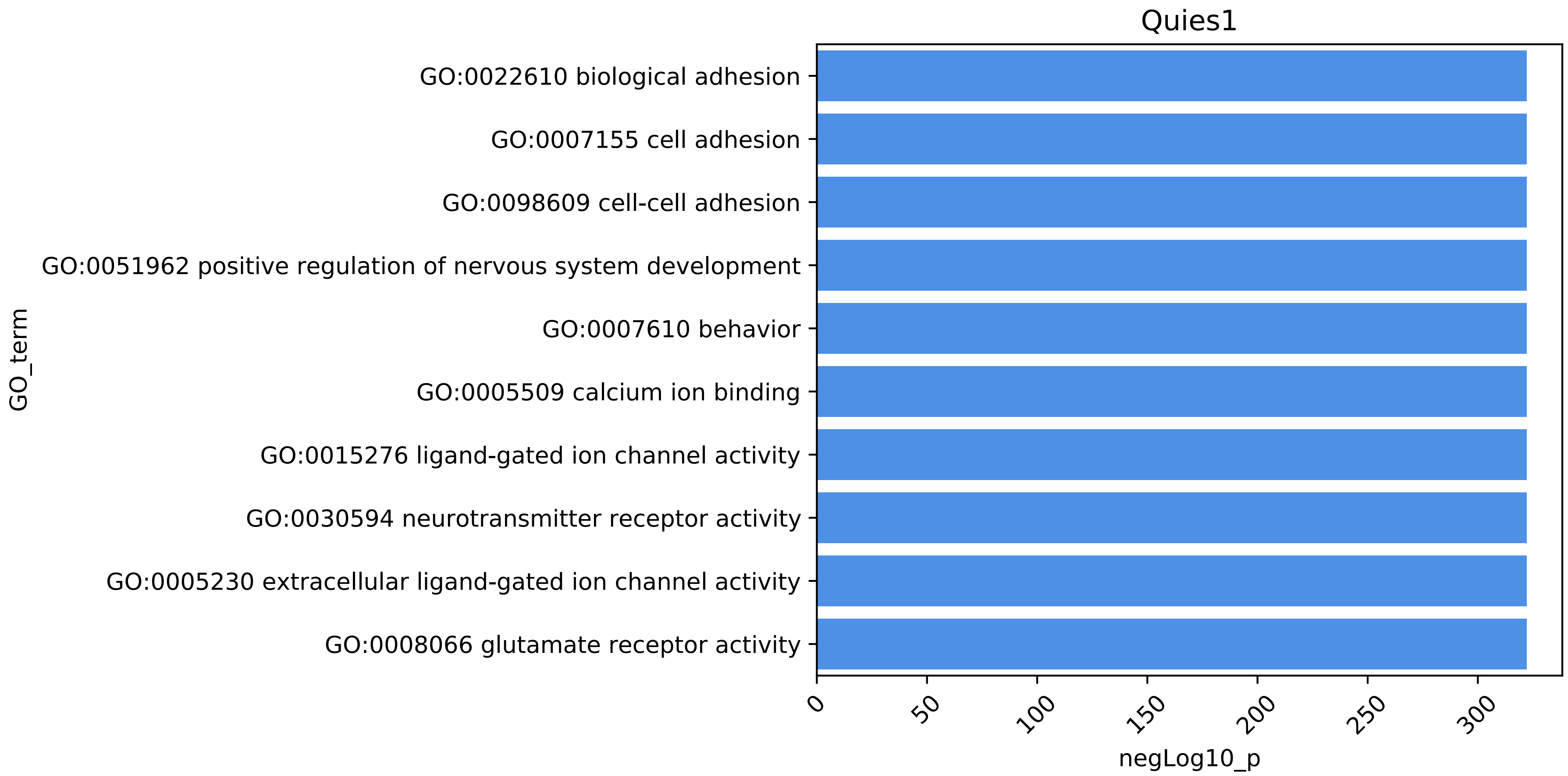
Quies1
GO:0022610 biological adhesion
GO:0007155 cell adhesion
GO:0098609 cell-cell adhesion
GO:0051962 positive regulation of nervous system development
GO:0007610 behavior
GO:0005509 calcium ion binding
GO:0015276 ligand-gated ion channel activity
GO:0030594 neurotransmitter receptor activity
GO:0005230 extracellular ligand-gated ion channel activity
GO:0008066 glutamate receptor activity
GO_term
0
50
100
150
200
250
300
negLog10_p

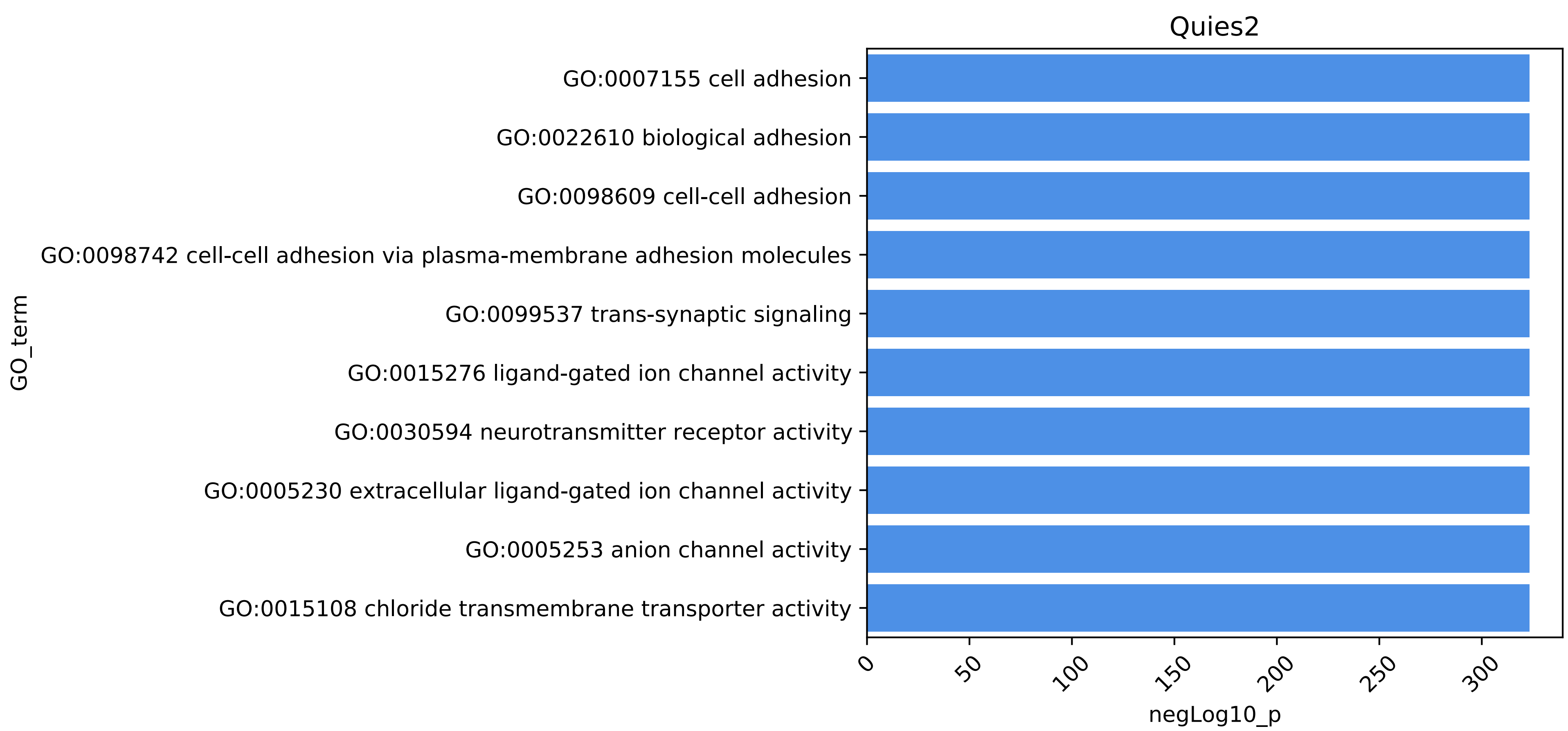

Quies2
GO:0007155 cell adhesion
GO:0022610 biological adhesion
GO:0098609 cell-cell adhesion
GO:0098742 cell-cell adhesion via plasma-membrane adhesion molecules
GO:0099537 trans-synaptic signaling
GO:0015276 ligand-gated ion channel activity
GO:0030594 neurotransmitter receptor activity
GO:0005230 extracellular ligand-gated ion channel activity
GO:0005253 anion channel activity
GO:0015108 chloride transmembrane transporter activity
GO_term
0
50
100
150
200
250
300
negLog10_p

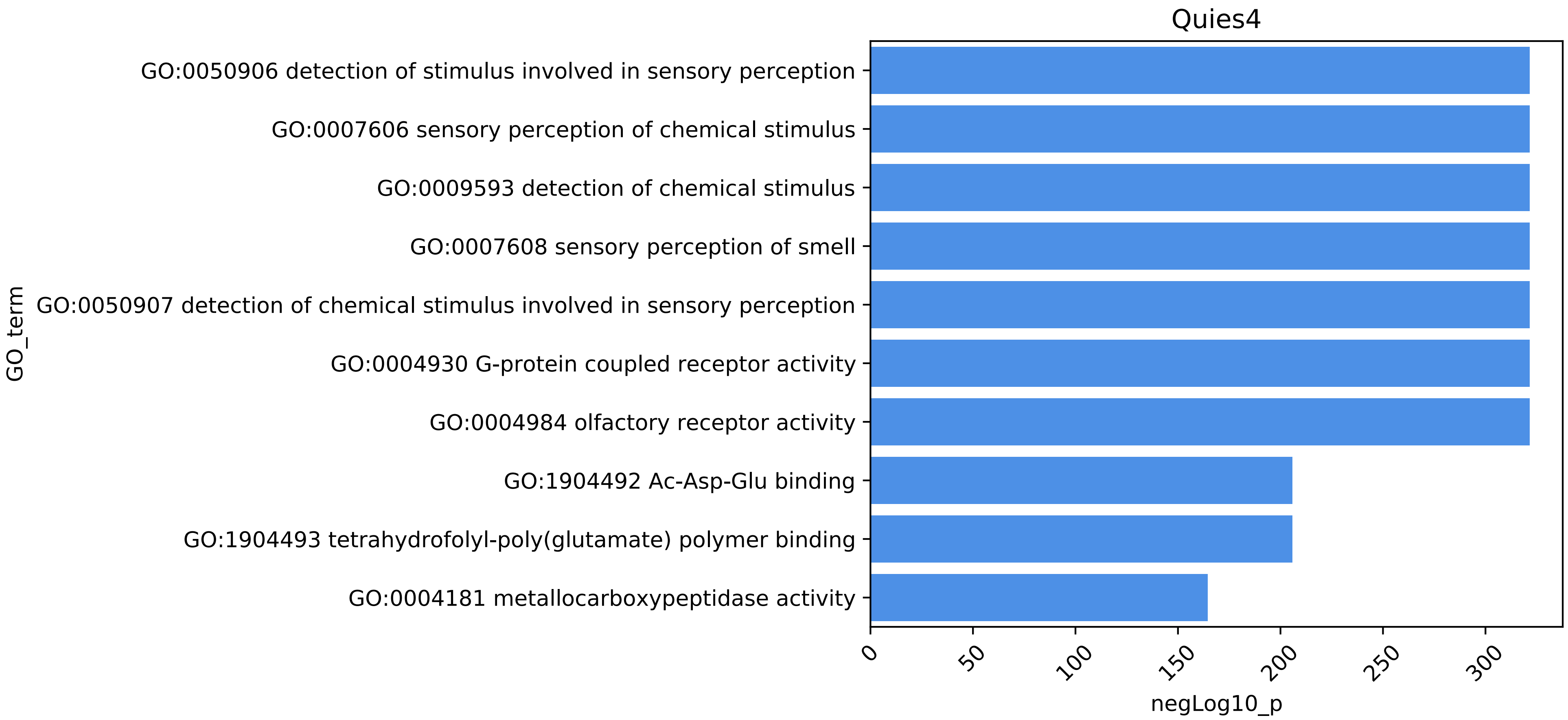
Quies4
GO:0050906 detection of stimulus involved in sensory perception
GO:0007606 sensory perception of chemical stimulus
GO:0009593 detection of chemical stimulus
GO:0007608 sensory perception of smell
GO:0050907 detection of chemical stimulus involved in sensory perception
GO:0004930 G-protein coupled receptor activity
GO:0004984 olfactory receptor activity
GO:1904492 Ac-Asp-Glu binding
GO:1904493 tetrahydrofolyl-poly(glutamate) polymer binding
GO:0004181 metallocarboxypeptidase activity
GO_term
0
50
100
150
200
250
300
negLog10_p

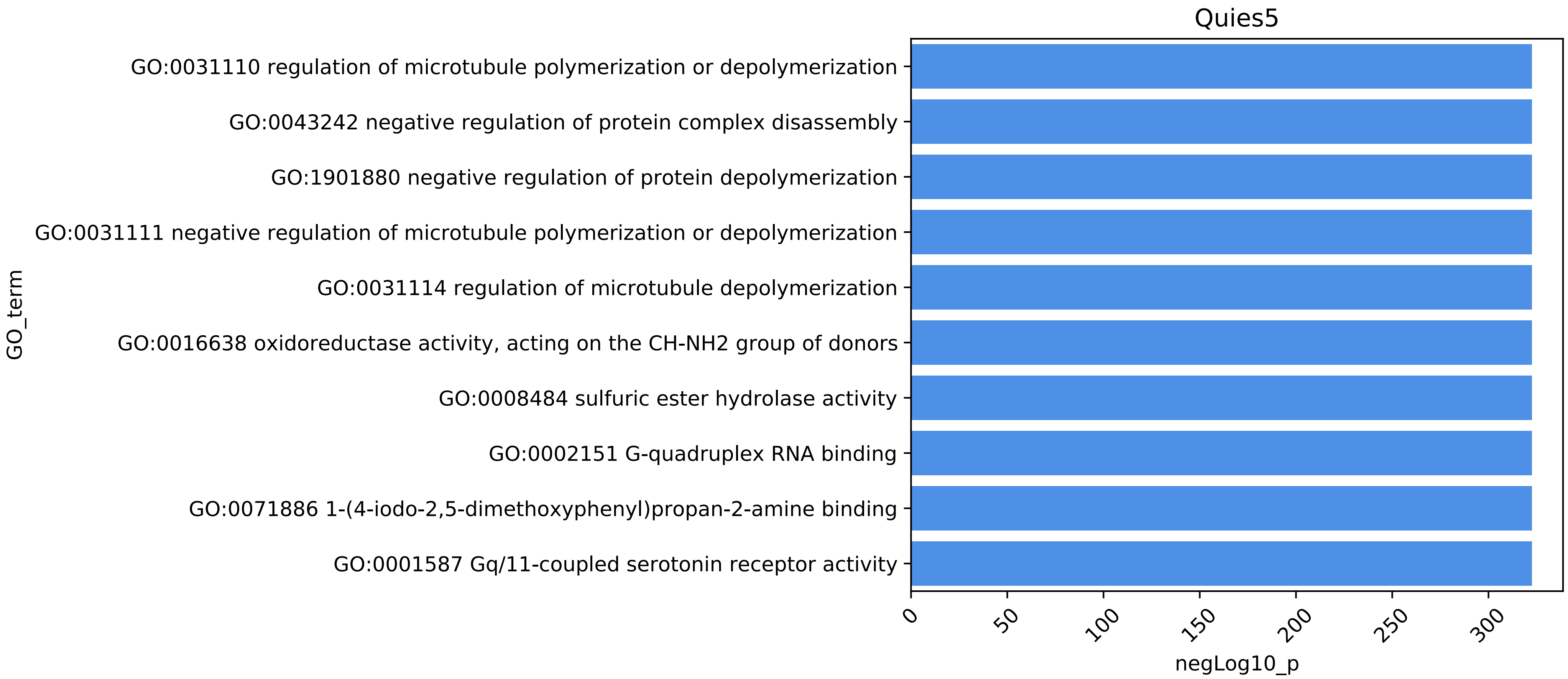
Quies5
GO:0031110 regulation of microtubule polymerization or depolymerization
GO:0043242 negative regulation of protein complex disassembly
GO:1901880 negative regulation of protein depolymerization
GO:0031111 negative regulation of microtubule polymerization or depolymerization
GO:0031114 regulation of microtubule depolymerization
GO:0016638 oxidoreductase activity, acting on the CH-NH2 group of donors
GO:0008484 sulfuric ester hydrolase activity
GO:0002151 G-quadruplex RNA binding
GO:0071886 1-(4-iodo-2,5-dimethoxyphenyl)propan-2-amine binding
GO:0001587 Gq/11-coupled serotonin receptor activity
GO_term
0
50
100
150
200
250
300
negLog10_p

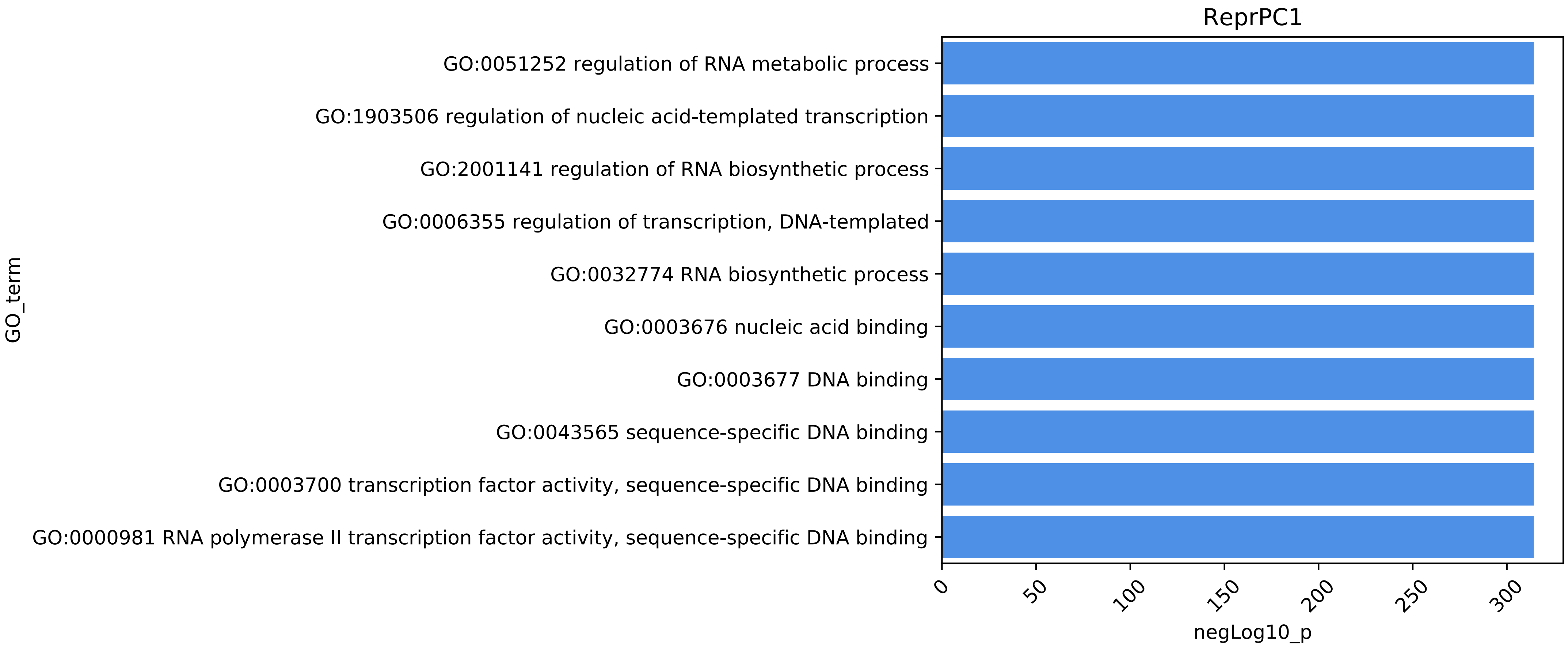
ReprPC1
GO:0051252 regulation of RNA metabolic process
GO:1903506 regulation of nucleic acid-templated transcription
GO:2001141 regulation of RNA biosynthetic process
GO:0006355 regulation of transcription, DNA-templated
GO:0032774 RNA biosynthetic process
GO:0003676 nucleic acid binding
GO:0003677 DNA binding
GO:0043565 sequence-specific DNA binding
GO:0003700 transcription factor activity, sequence-specific DNA binding
GO:0000981 RNA polymerase II transcription factor activity, sequence-specific DNA binding
GO_term
0
50
100
150
200
250
300
negLog10_p

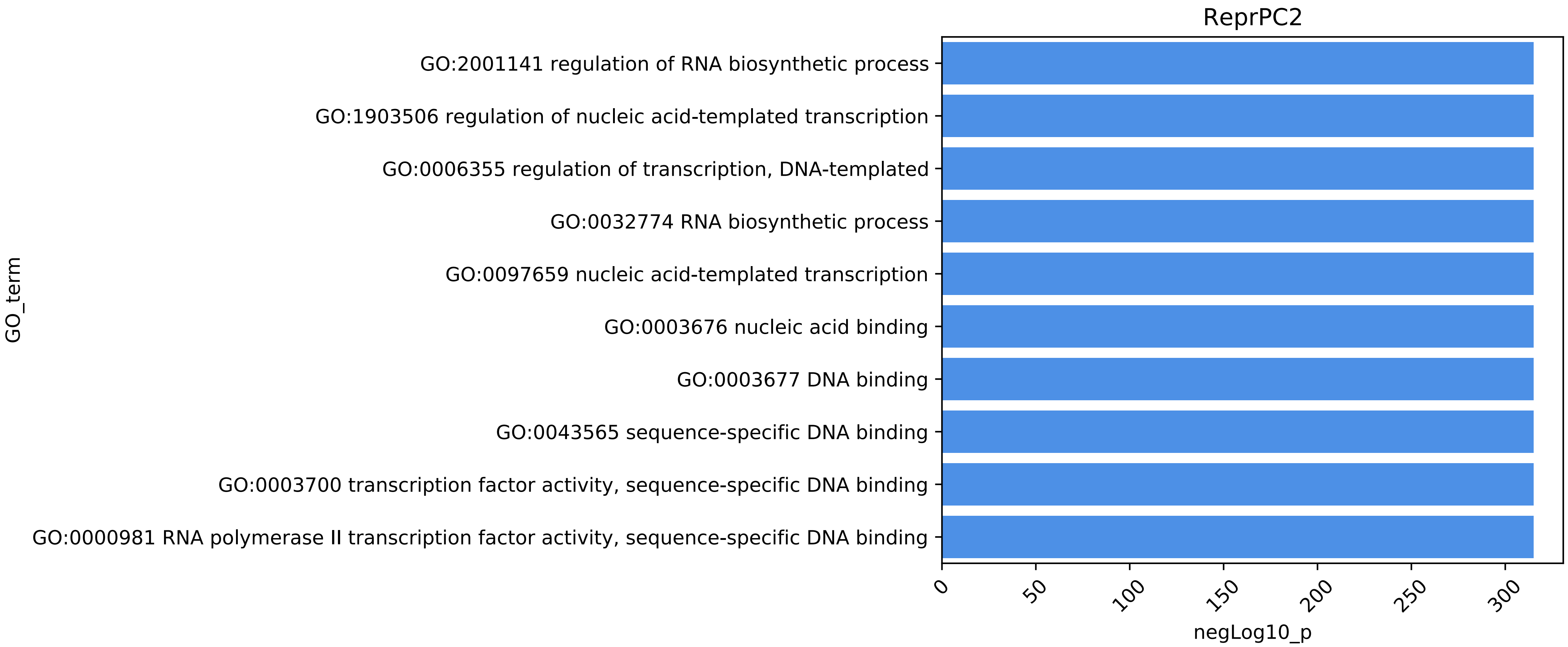
ReprPC2
GO_term
GO:2001141 regulation of RNA biosynthetic process
GO:1903506 regulation of nucleic acid-templated transcription
GO:0006355 regulation of transcription, DNA-templated
GO:0032774 RNA biosynthetic process
GO:0097659 nucleic acid-templated transcription
GO:0003676 nucleic acid binding
GO:0003677 DNA binding
GO:0043565 sequence-specific DNA binding
GO:0003700 transcription factor activity, sequence-specific DNA binding
GO:0000981 RNA polymerase II transcription factor activity, sequence-specific DNA binding
0
50
100
150
200
250
300
negLog10_p

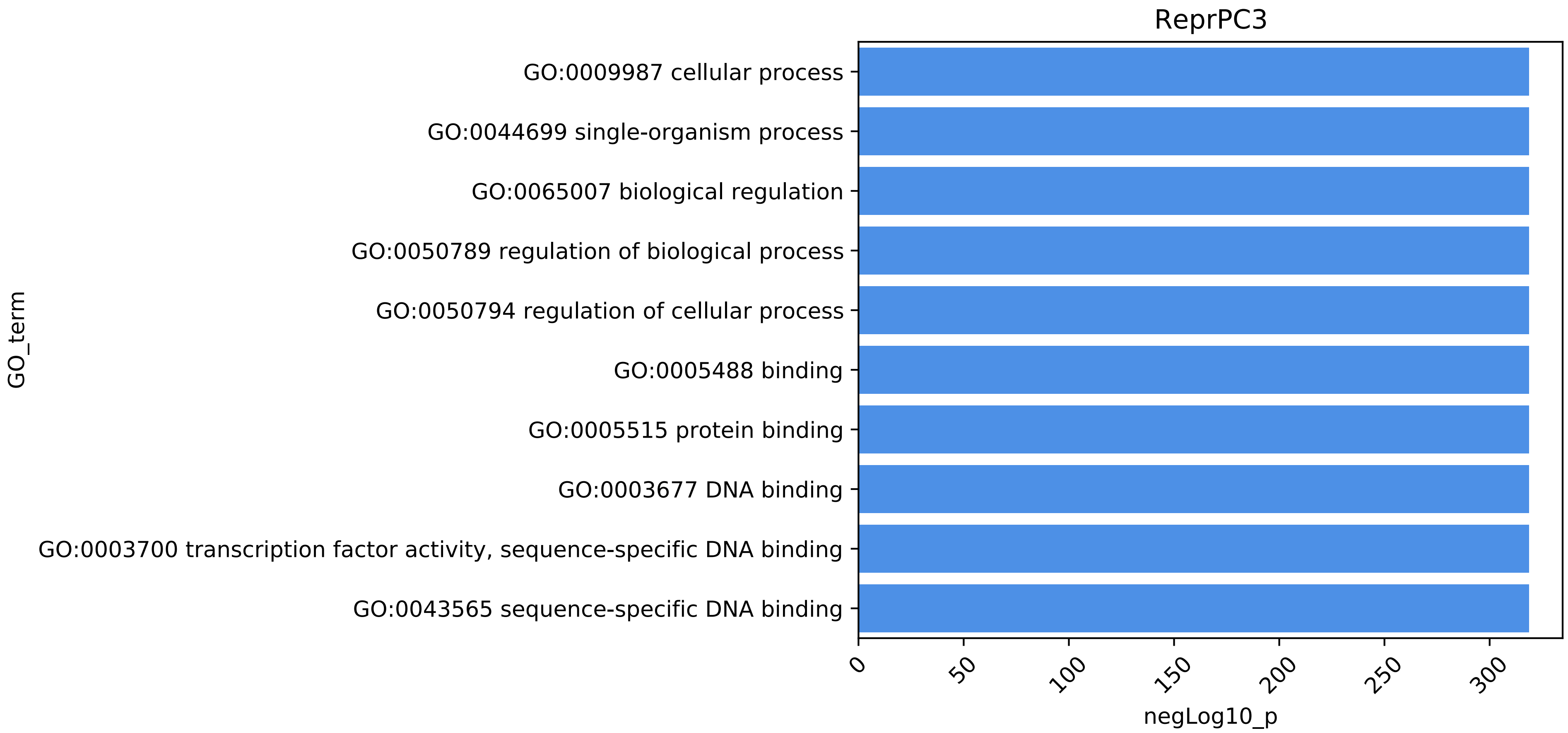
ReprPC3
GO_term
GO:0009987 cellular process
GO:0044699 single-organism process
GO:0065007 biological regulation
GO:0050789 regulation of biological process
GO:0050794 regulation of cellular process
GO:0005488 binding
GO:0005515 protein binding
GO:0003677 DNA binding
GO:0003700 transcription factor activity, sequence-specific DNA binding
GO:0043565 sequence-specific DNA binding
0
50
100
150
200
250
300
negLog10_p

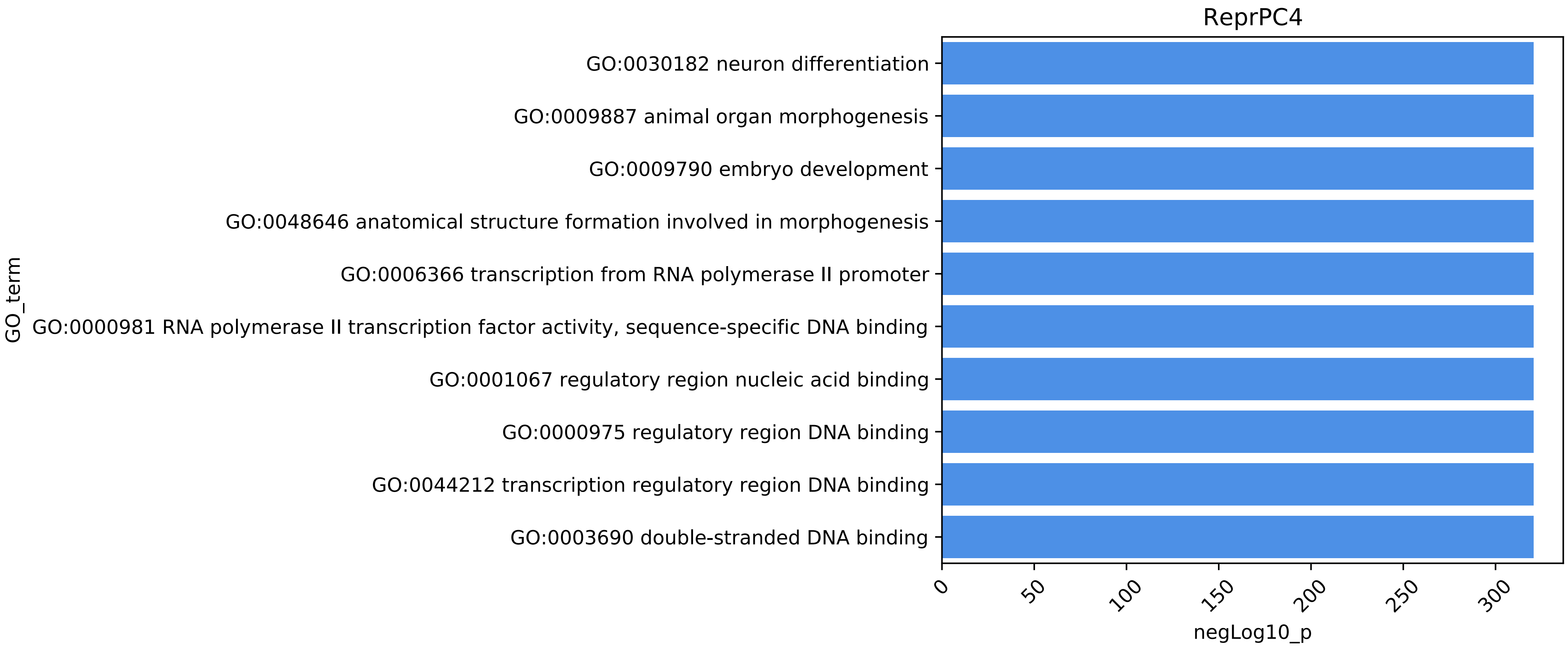
ReprPC4
GO:0030182 neuron differentiation
GO:0009887 animal organ morphogenesis
GO:0009790 embryo development
GO:0048646 anatomical structure formation involved in morphogenesis
GO:0006366 transcription from RNA polymerase II promoter
GO:0000981 RNA polymerase II transcription factor activity, sequence-specific DNA binding
GO:0001067 regulatory region nucleic acid binding
GO:0000975 regulatory region DNA binding
GO:0044212 transcription regulatory region DNA binding
GO:0003690 double-stranded DNA binding
GO_term
0
50
100
150
200
250
300
negLog10_p

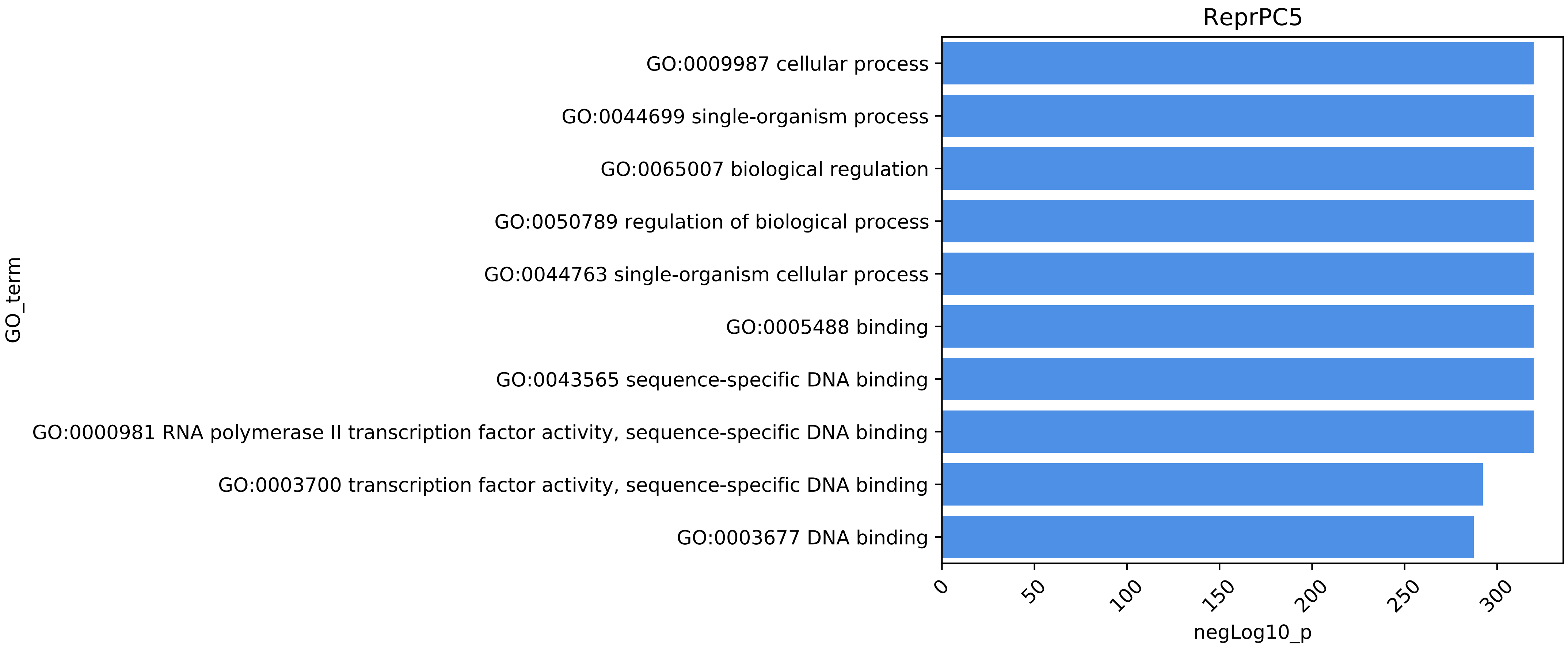
ReprPC5
GO:0009987 cellular process
GO:0044699 single-organism process
GO:0065007 biological regulation
GO:0050789 regulation of biological process
GO:0044763 single-organism cellular process
GO:0005488 binding
GO:0043565 sequence-specific DNA binding
GO:0000981 RNA polymerase II transcription factor activity, sequence-specific DNA binding
GO:0003700 transcription factor activity, sequence-specific DNA binding
GO:0003677 DNA binding
GO_term
0
50
100
150
200
250
300
negLog10_p

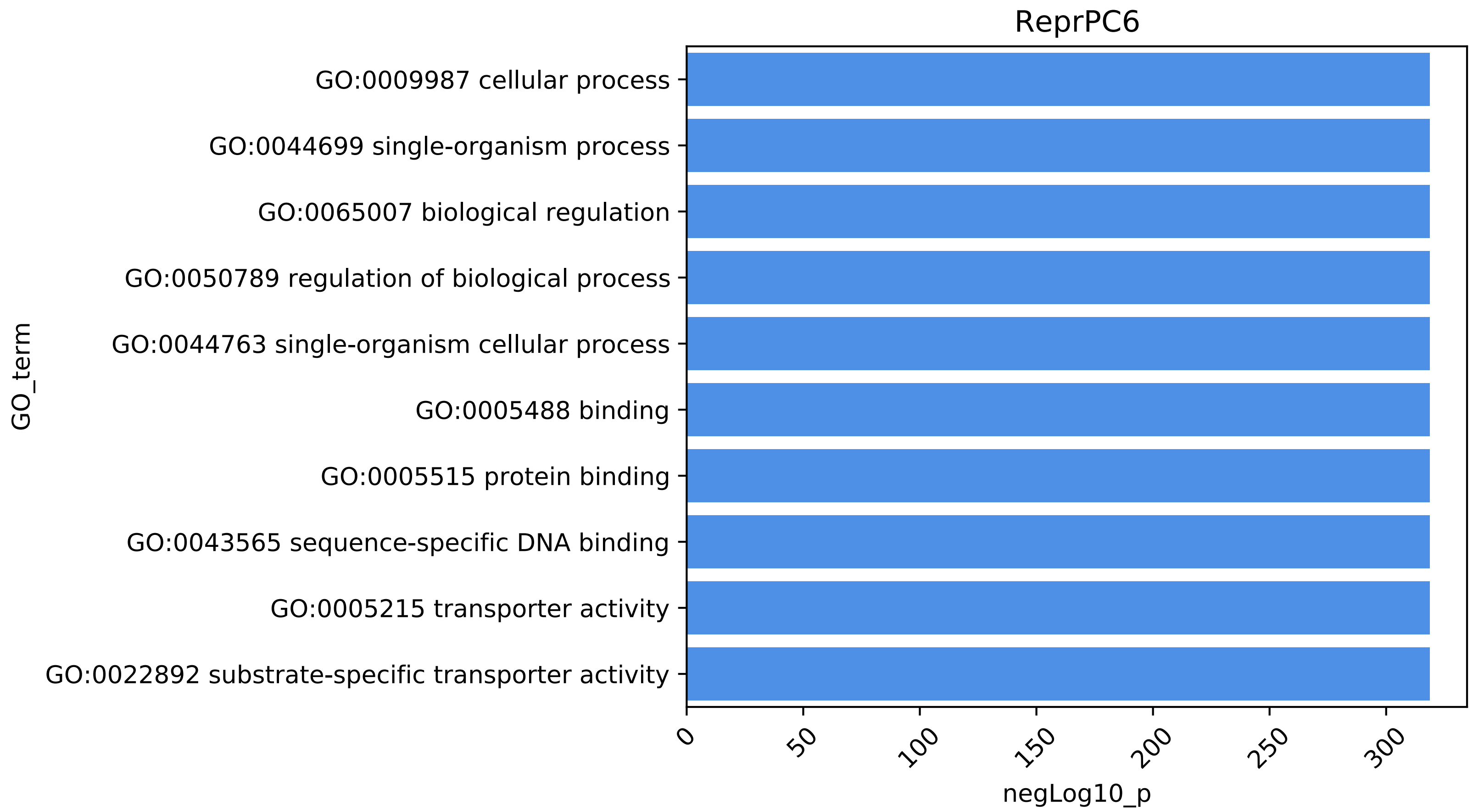

ReprPC6
GO_term
GO:0009987 cellular process
GO:0044699 single-organism process
GO:0065007 biological regulation
GO:0050789 regulation of biological process
GO:0044763 single-organism cellular process
GO:0005488 binding
GO:0005515 protein binding
GO:0043565 sequence-specific DNA binding
GO:0005215 transporter activity
GO:0022892 substrate-specific transporter activity
0
50
100
150
200
250
300
negLog10_p

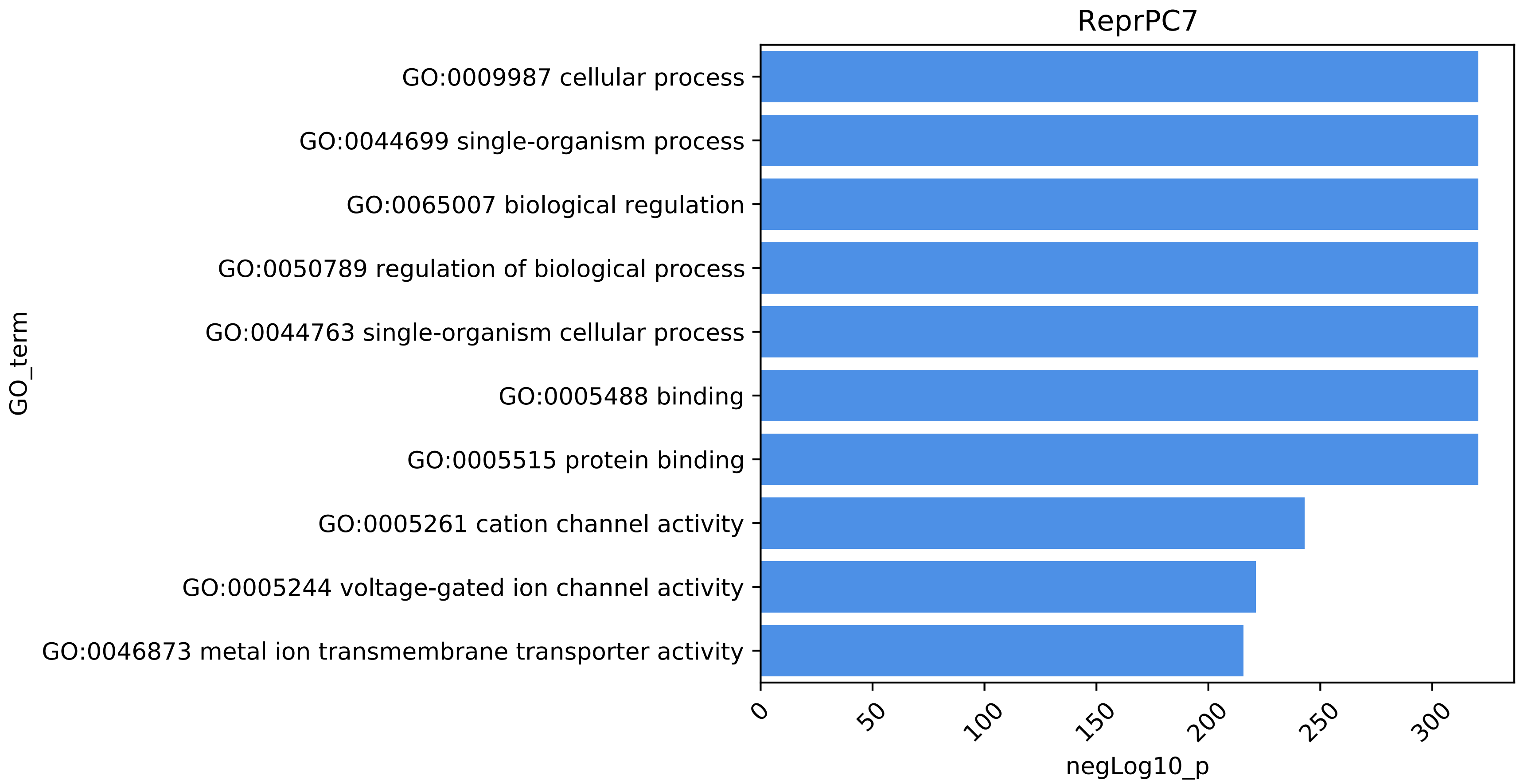

ReprPC7
GO:0009987 cellular process
GO:0044699 single-organism process
GO:0065007 biological regulation
GO:0050789 regulation of biological process
GO:0044763 single-organism cellular process
GO:0005488 binding
GO:0005515 protein binding
GO:0005261 cation channel activity
GO:0005244 voltage-gated ion channel activity
GO:0046873 metal ion transmembrane transporter activity
GO_term
0
50
100
150
200
250
300
negLog10_p

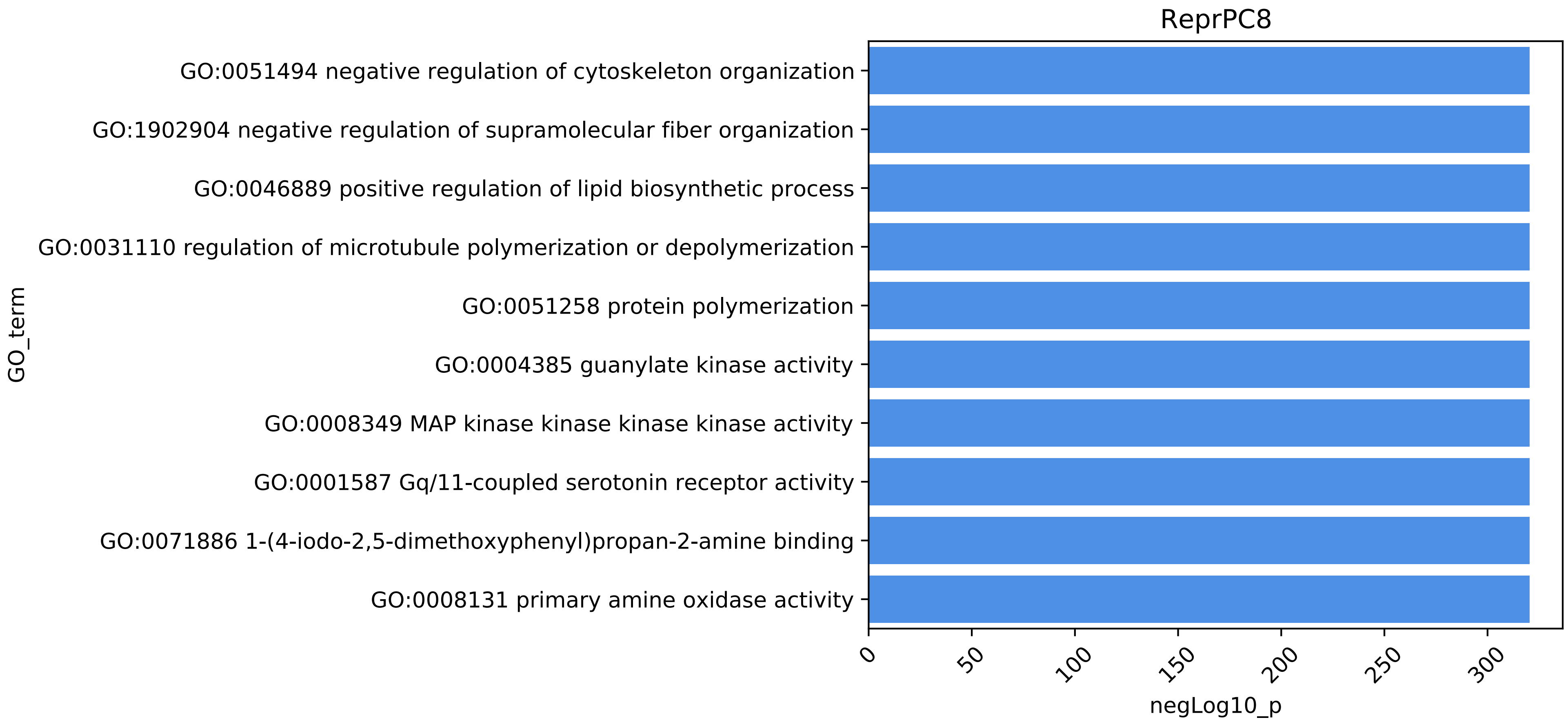
ReprPC8
GO_term
GO:0051494 negative regulation of cytoskeleton organization
GO:1902904 negative regulation of supramolecular fiber organization
GO:0046889 positive regulation of lipid biosynthetic process
GO:0031110 regulation of microtubule polymerization or depolymerization
GO:0051258 protein polymerization
GO:0004385 guanylate kinase activity
GO:0008349 MAP kinase kinase kinase kinase activity
GO:0001587 Gq/11-coupled serotonin receptor activity
GO:0071886 1-(4-iodo-2,5-dimethoxyphenyl)propan-2-amine binding
GO:0008131 primary amine oxidase activity
0
50
100
150
200
250
300
negLog10_p

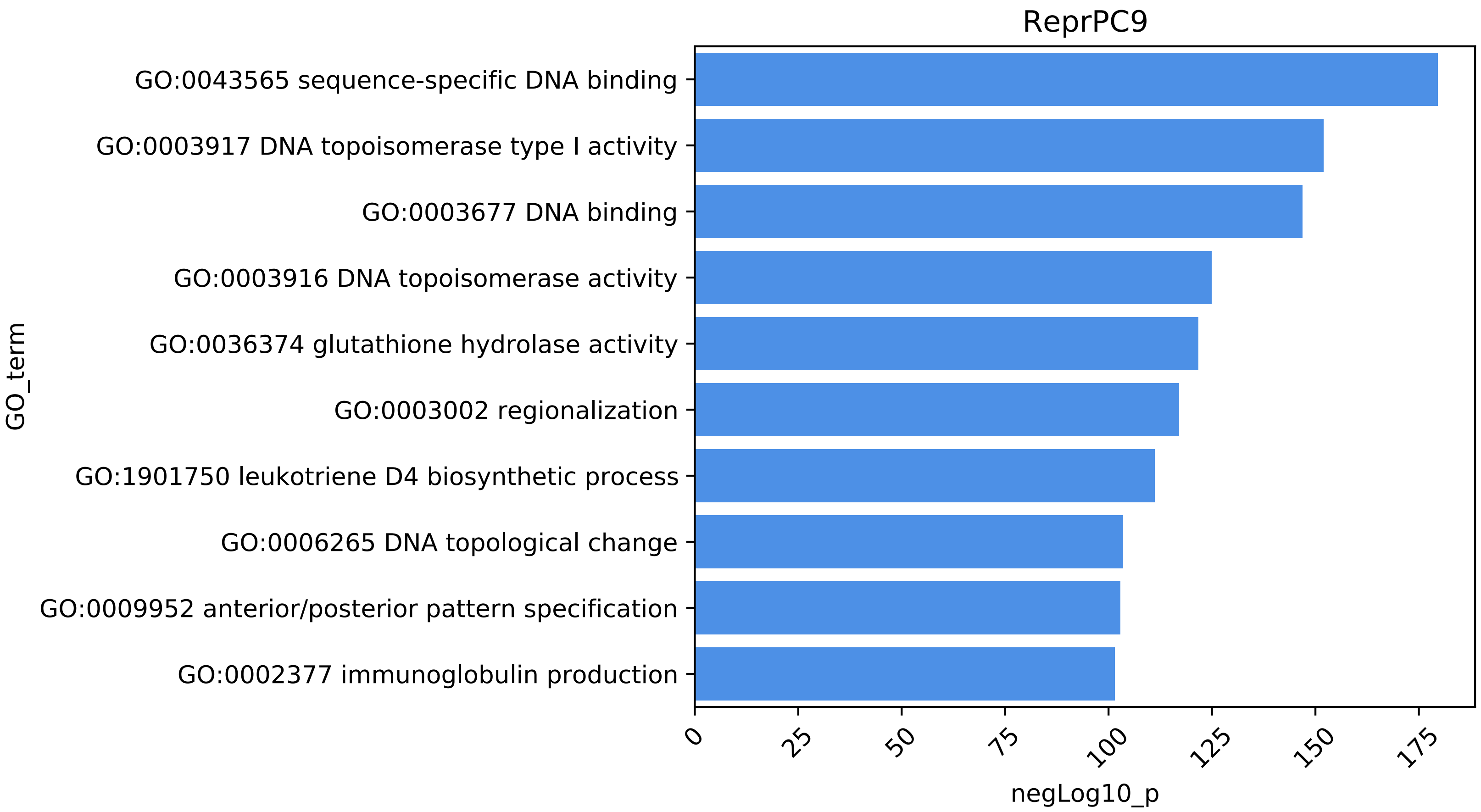
ReprPC9
GO:0043565 sequence-specific DNA binding
GO:0003917 DNA topoisomerase type I activity
GO:0003677 DNA binding
GO:0003916 DNA topoisomerase activity
GO:0036374 glutathione hydrolase activity
GO:0003002 regionalization
GO:1901750 leukotriene D4 biosynthetic process
GO:0006265 DNA topological change
GO:0009952 anterior/posterior pattern specification
GO:0002377 immunoglobulin production
GO_term
0
25
50
75
100
125
150
175
negLog10_p

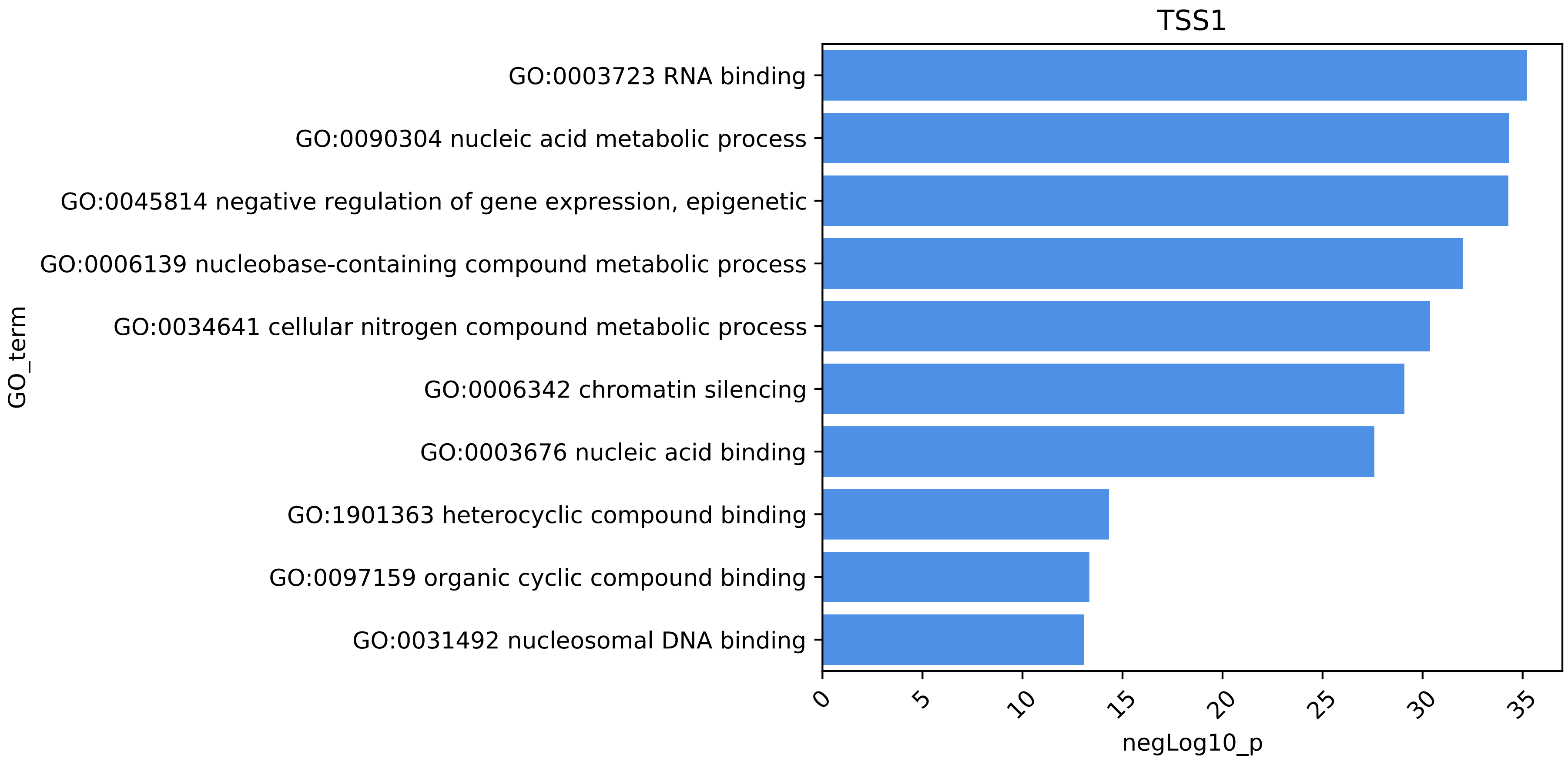
TSS1
GO:0003723 RNA binding
GO:0090304 nucleic acid metabolic process
GO:0045814 negative regulation of gene expression, epigenetic
GO:0006139 nucleobase-containing compound metabolic process
GO:0034641 cellular nitrogen compound metabolic process
GO:0006342 chromatin silencing
GO:0003676 nucleic acid binding
GO:1901363 heterocyclic compound binding
GO:0097159 organic cyclic compound binding
GO:0031492 nucleosomal DNA binding
GO_term
0
5
10
15
20
25
30
35
negLog10_p

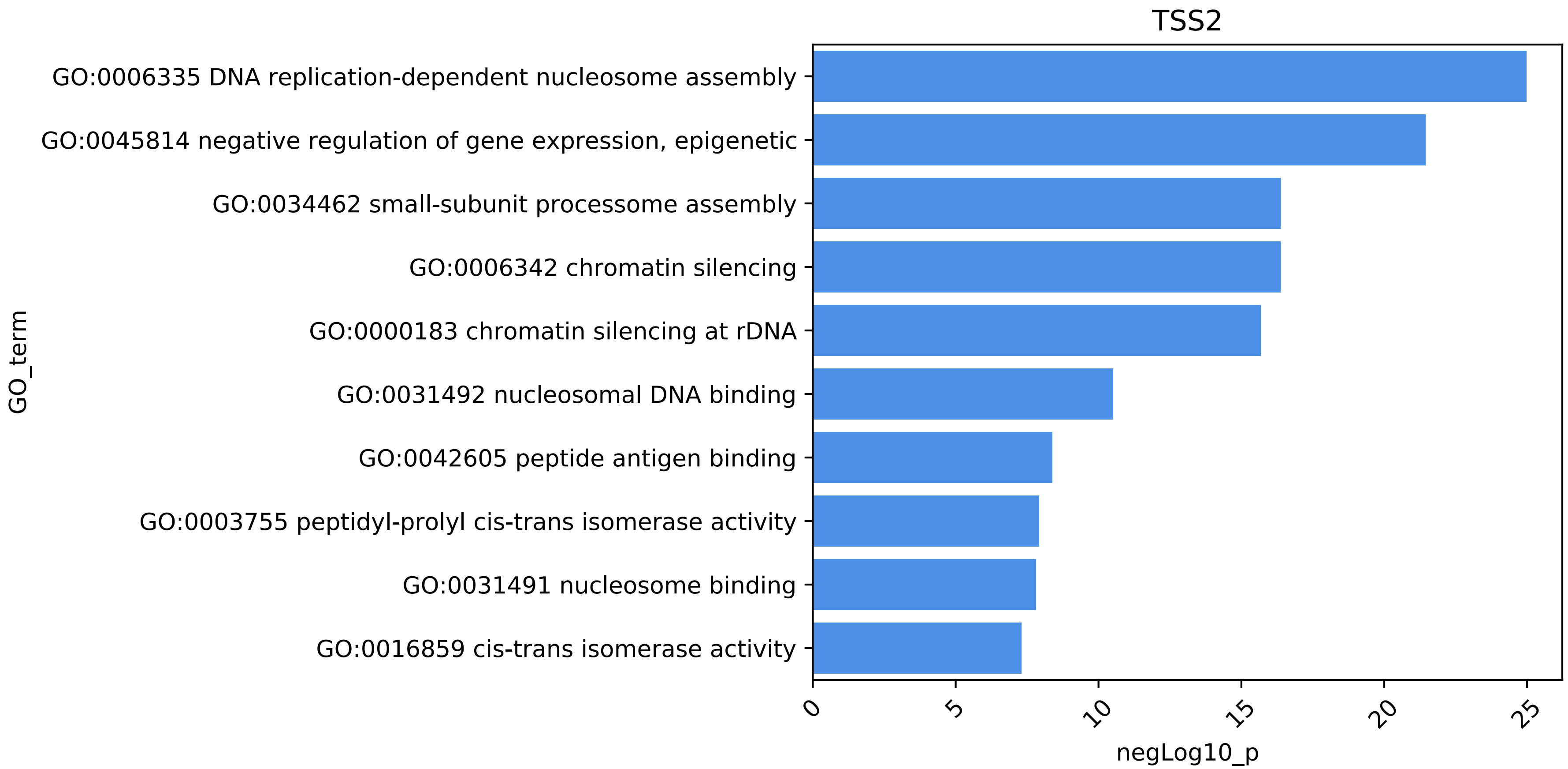

TSS2
GO:0006335 DNA replication-dependent nucleosome assembly
GO:0045814 negative regulation of gene expression, epigenetic
GO:0034462 small-subunit processome assembly
GO:0006342 chromatin silencing
GO:0000183 chromatin silencing at rDNA
GO:0031492 nucleosomal DNA binding
GO:0042605 peptide antigen binding
GO:0003755 peptidyl-prolyl cis-trans isomerase activity
GO:0031491 nucleosome binding
GO:0016859 cis-trans isomerase activity
GO_term
0
5
10
15
20
25
negLog10_p

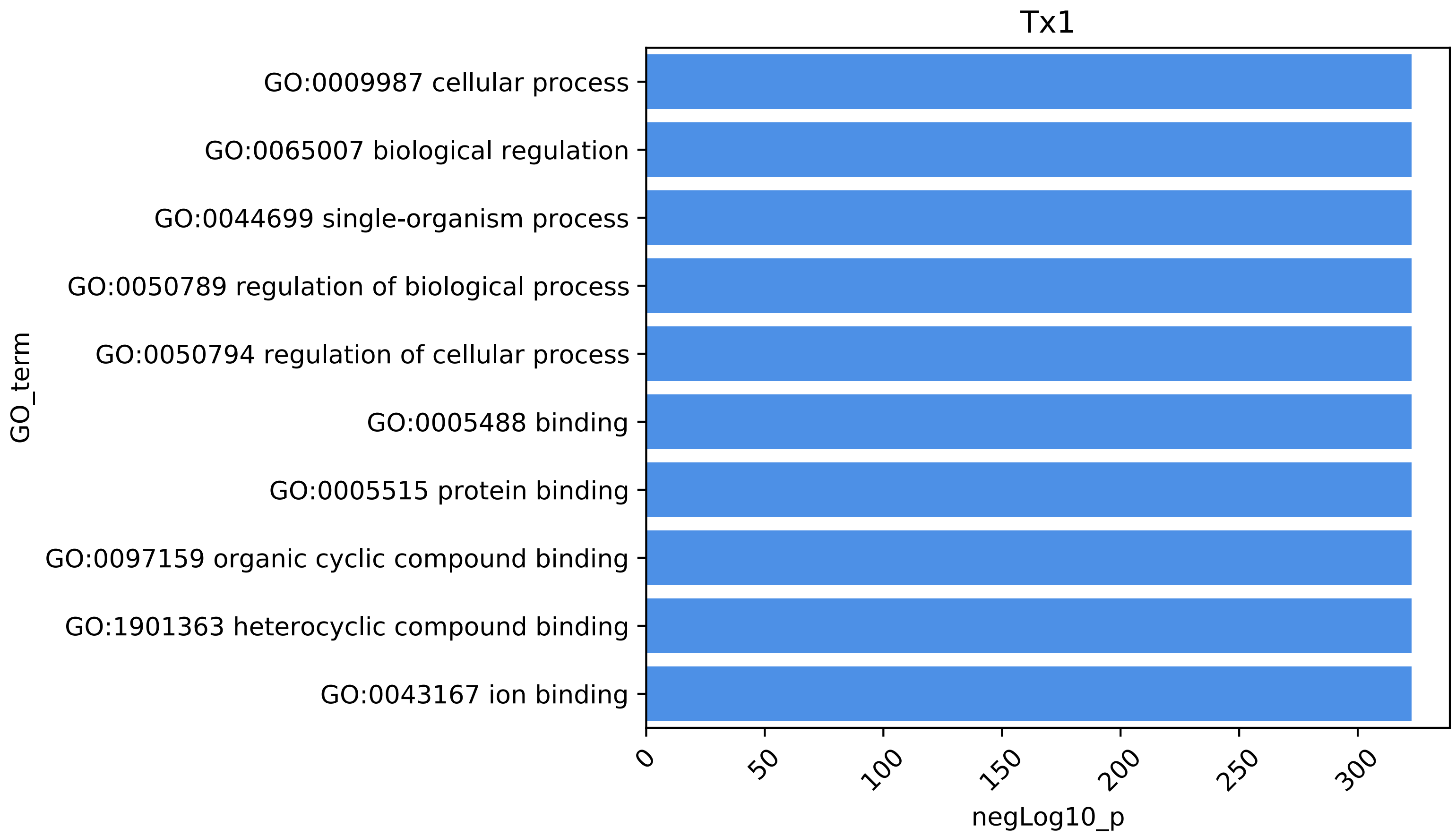
Tx1
GO:0009987 cellular process
GO:0065007 biological regulation
GO:0044699 single-organism process
GO:0050789 regulation of biological process
GO:0050794 regulation of cellular process
GO:0005488 binding
GO:0005515 protein binding
GO:0097159 organic cyclic compound binding
GO:1901363 heterocyclic compound binding
GO:0043167 ion binding
GO_term
0
50
100
150
200
250
300
negLog10_p

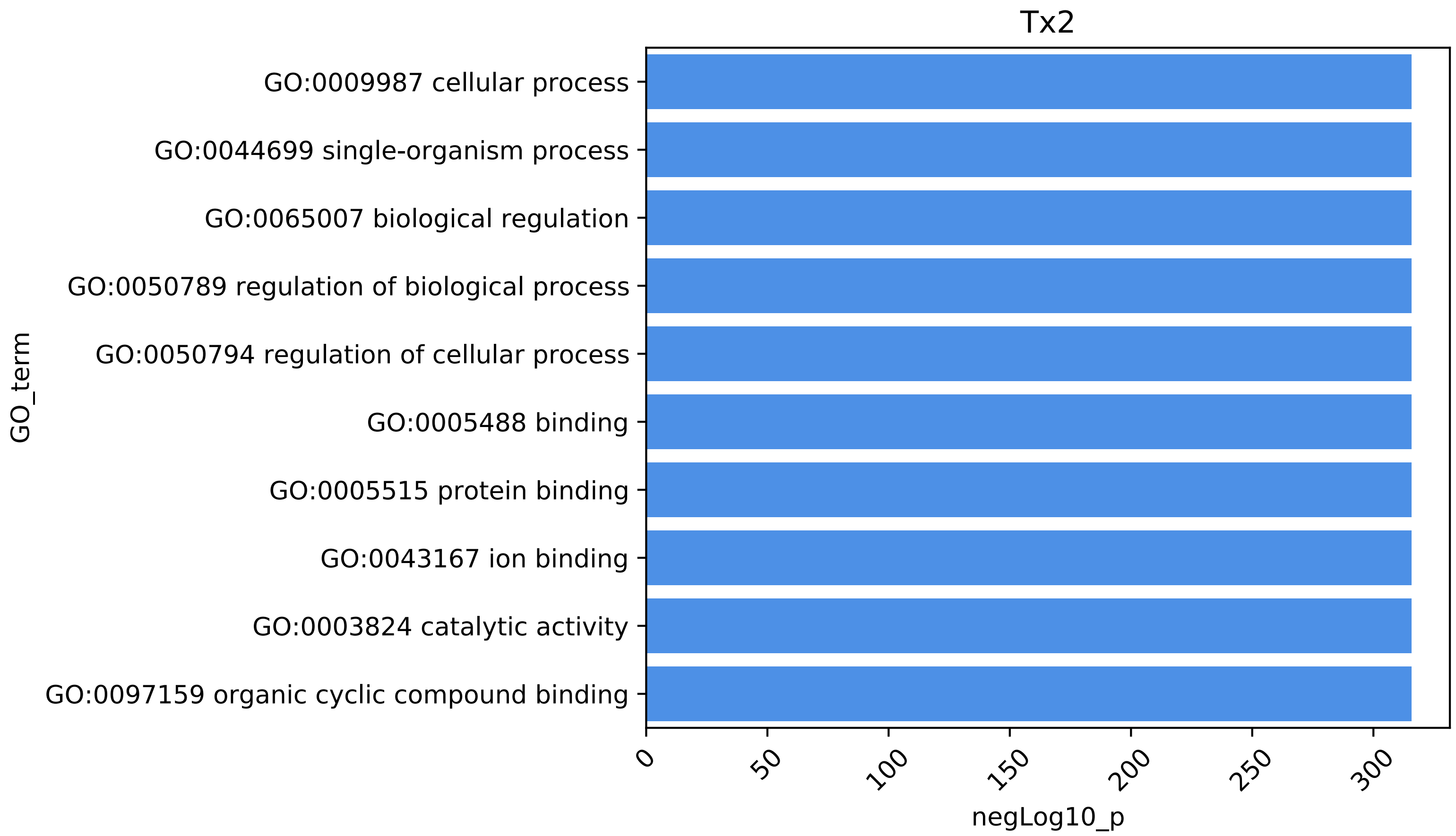
Tx2
GO:0009987 cellular process
GO:0044699 single-organism process
GO:0065007 biological regulation
GO:0050789 regulation of biological process
GO:0050794 regulation of cellular process
GO:0005488 binding
GO:0005515 protein binding
GO:0043167 ion binding
GO:0003824 catalytic activity
GO:0097159 organic cyclic compound binding
GO_term
0
50
100
150
200
250
300
negLog10_p

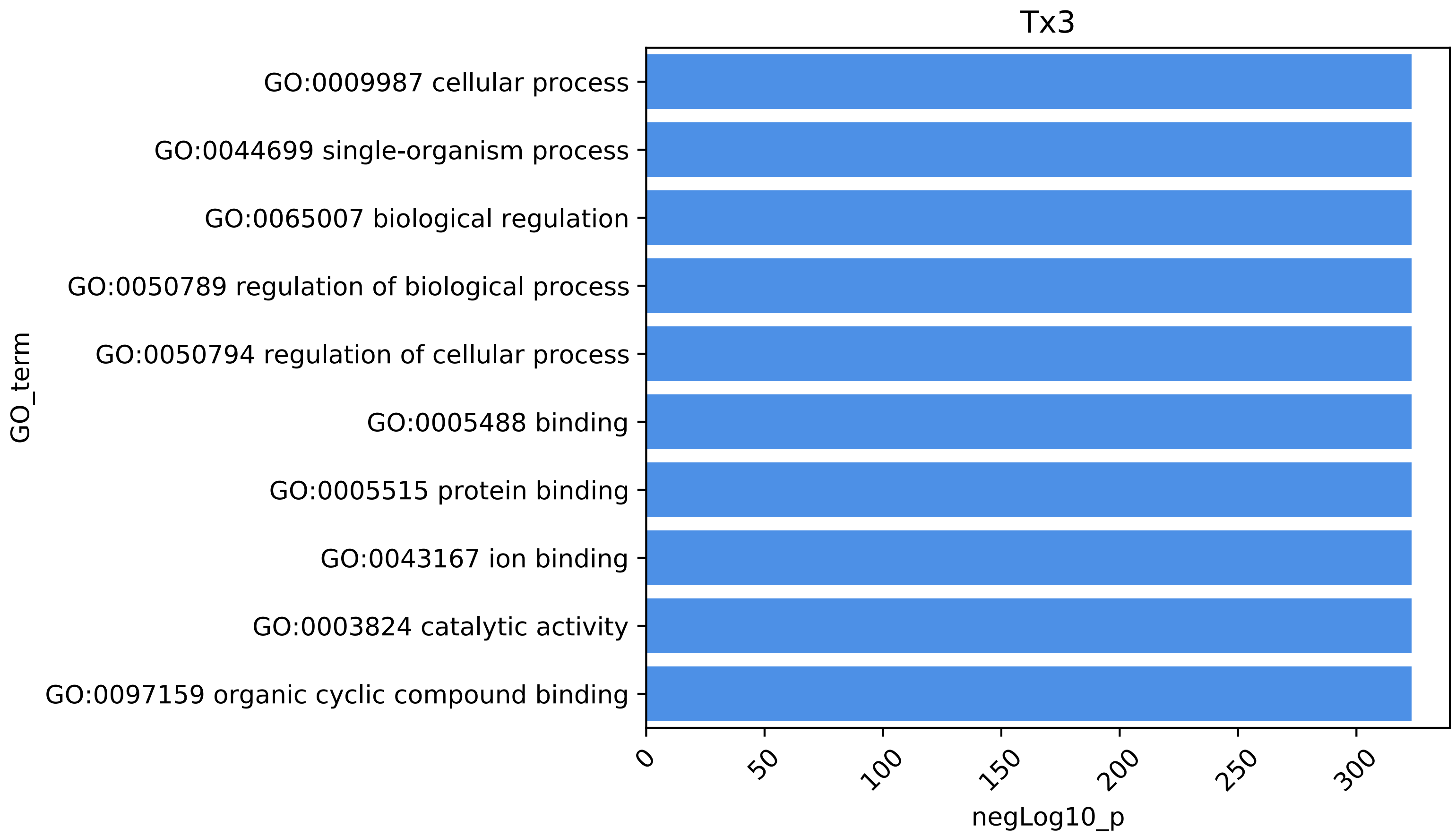

Tx3
GO_term
GO:0009987 cellular process
GO:0044699 single-organism process
GO:0065007 biological regulation
GO:0050789 regulation of biological process
GO:0050794 regulation of cellular process
GO:0005488 binding
GO:0005515 protein binding
GO:0043167 ion binding
GO:0003824 catalytic activity
GO:0097159 organic cyclic compound binding
0
50
100
150
200
250
300
negLog10_p

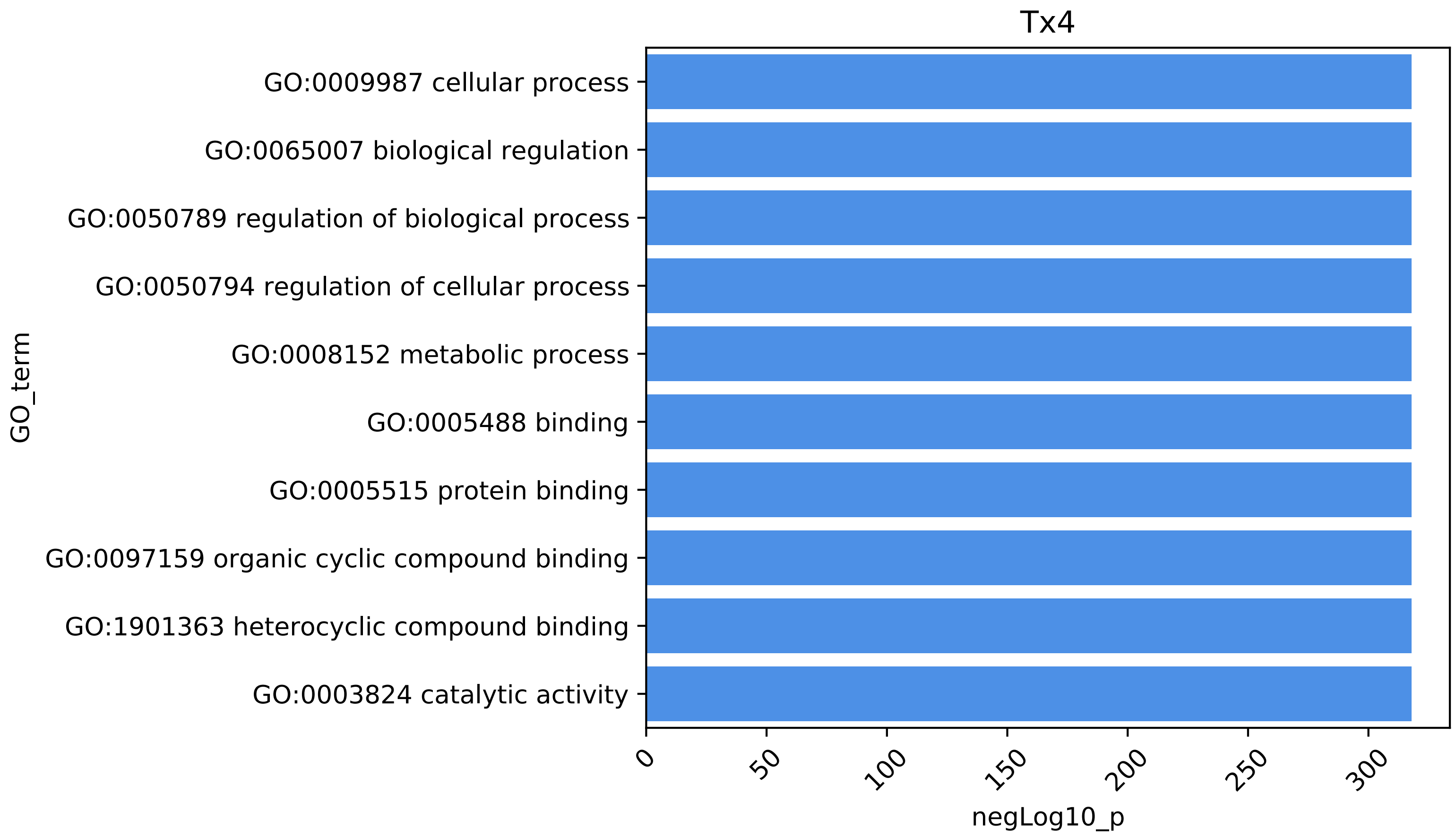
Tx4
GO:0009987 cellular process
GO:0065007 biological regulation
GO:0050789 regulation of biological process
GO:0050794 regulation of cellular process
GO:0008152 metabolic process
GO:0005488 binding
GO:0005515 protein binding
GO:0097159 organic cyclic compound binding
GO:1901363 heterocyclic compound binding
GO:0003824 catalytic activity
GO_term
0
50
100
150
200
250
300
negLog10_p

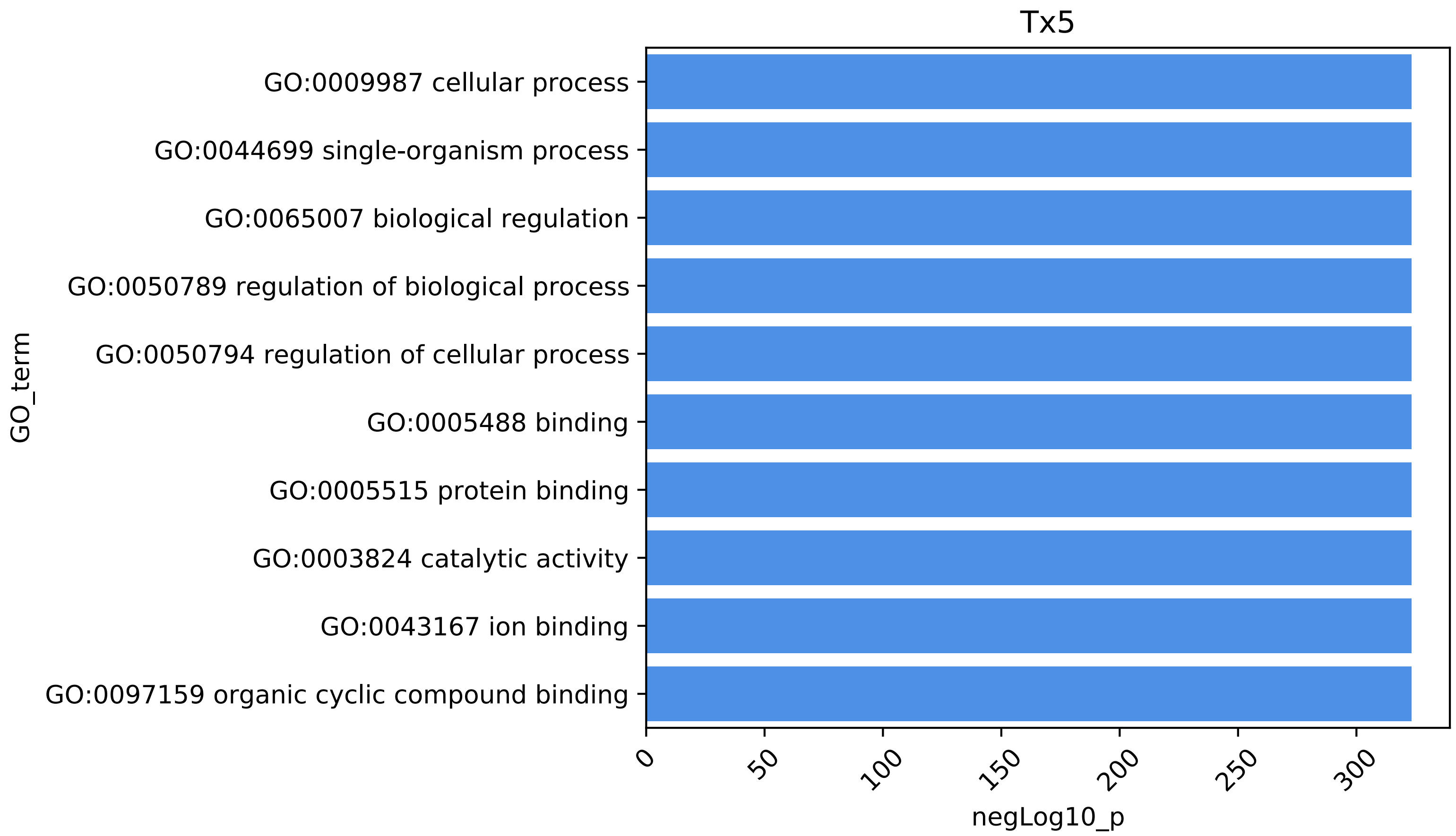

Tx5
GO:0009987 cellular process
GO:0044699 single-organism process
GO:0065007 biological regulation
GO:0050789 regulation of biological process
GO:0050794 regulation of cellular process
GO:0005488 binding
GO:0005515 protein binding
GO:0003824 catalytic activity
GO:0043167 ion binding
GO:0097159 organic cyclic compound binding
GO_term
0
50
100
150
200
250
300
negLog10_p

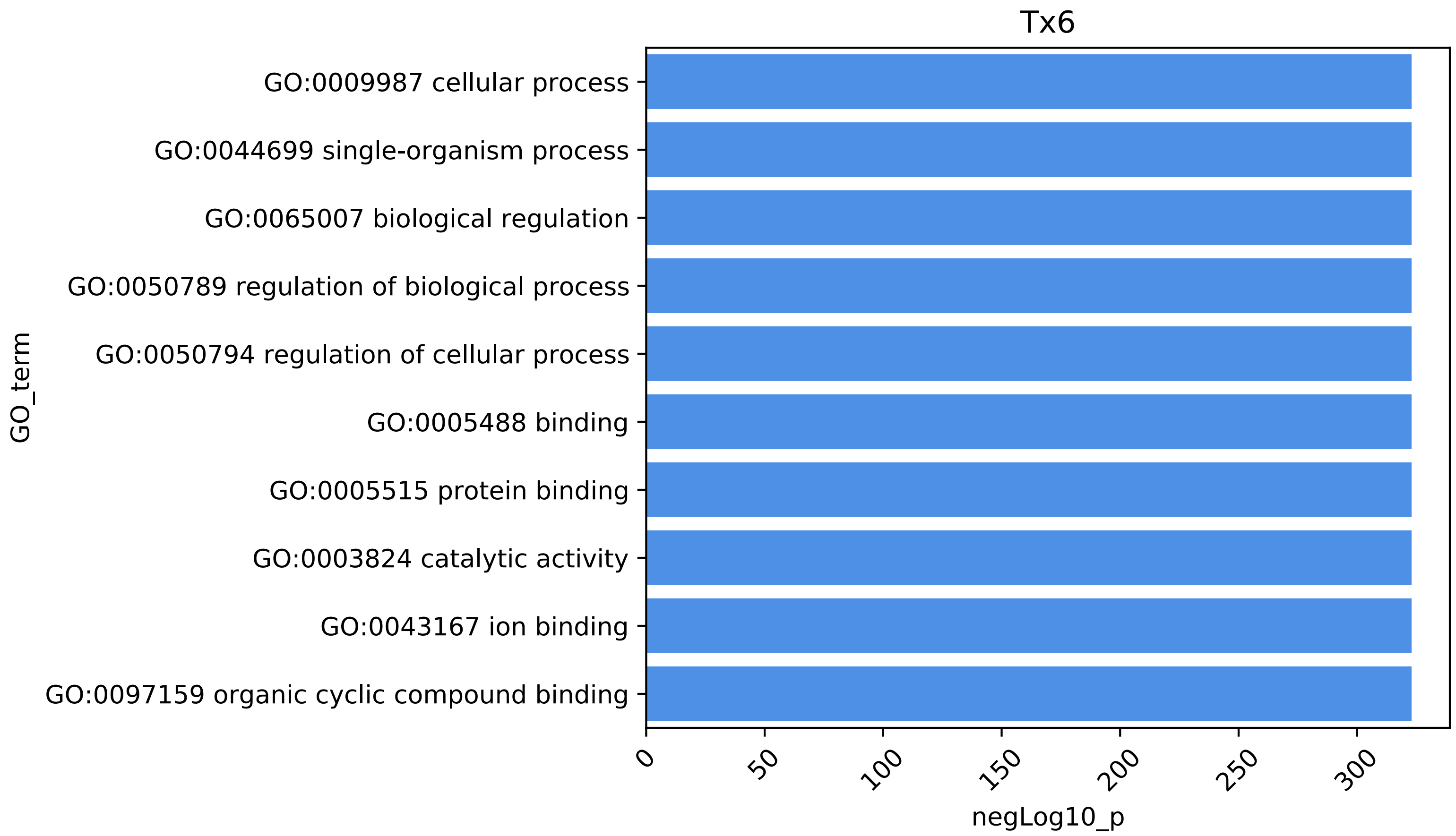

Tx6
GO:0009987 cellular process
GO:0044699 single-organism process
GO:0065007 biological regulation
GO:0050789 regulation of biological process
GO:0050794 regulation of cellular process
GO:0005488 binding
GO:0005515 protein binding
GO:0003824 catalytic activity
GO:0043167 ion binding
GO:0097159 organic cyclic compound binding
GO_term
0
50
100
150
200
250
300
negLog10_p

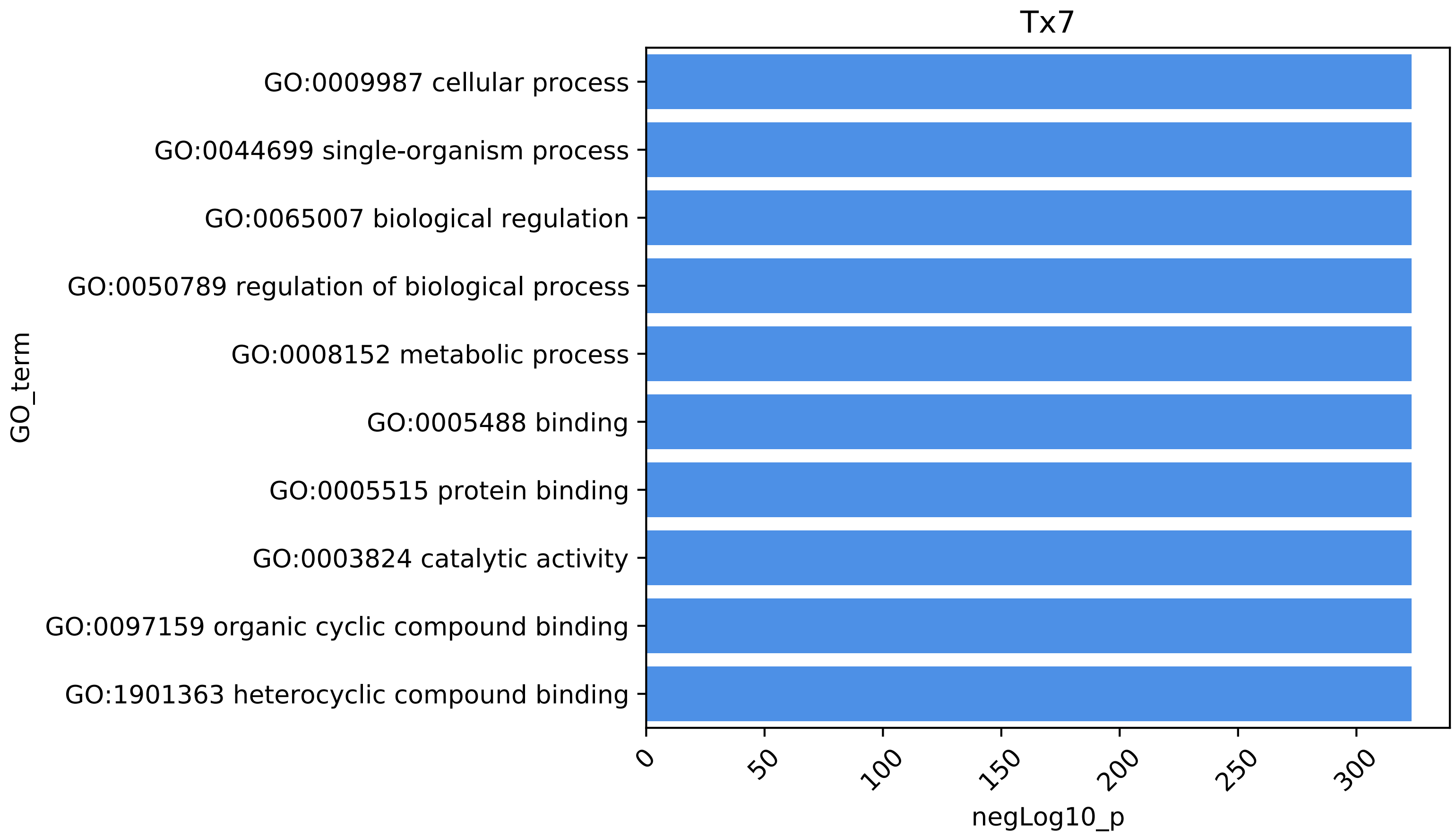
Tx7
GO:0009987 cellular process
GO:0044699 single-organism process
GO:0065007 biological regulation
GO:0050789 regulation of biological process
GO:0008152 metabolic process
GO:0005488 binding
GO:0005515 protein binding
GO:0003824 catalytic activity
GO:0097159 organic cyclic compound binding
GO:1901363 heterocyclic compound binding
GO_term
0
50
100
150
200
250
300
negLog10_p

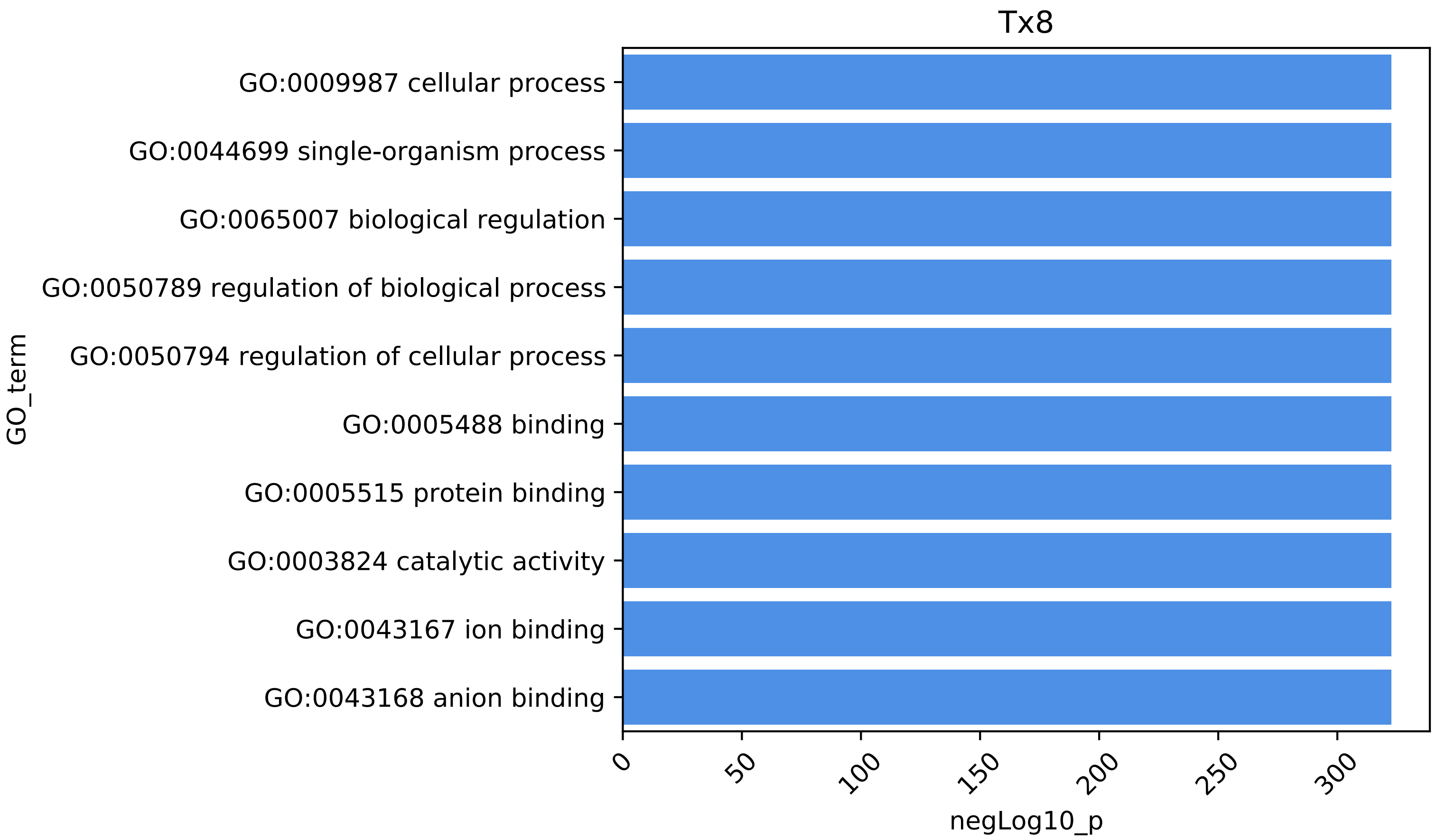
Tx8
GO:0009987 cellular process
GO:0044699 single-organism process
GO:0065007 biological regulation
GO:0050789 regulation of biological process
GO:0050794 regulation of cellular process
GO:0005488 binding
GO:0005515 protein binding
GO:0003824 catalytic activity
GO:0043167 ion binding
GO:0043168 anion binding
GO_term
0
50
100
150
200
250
300
negLog10_p

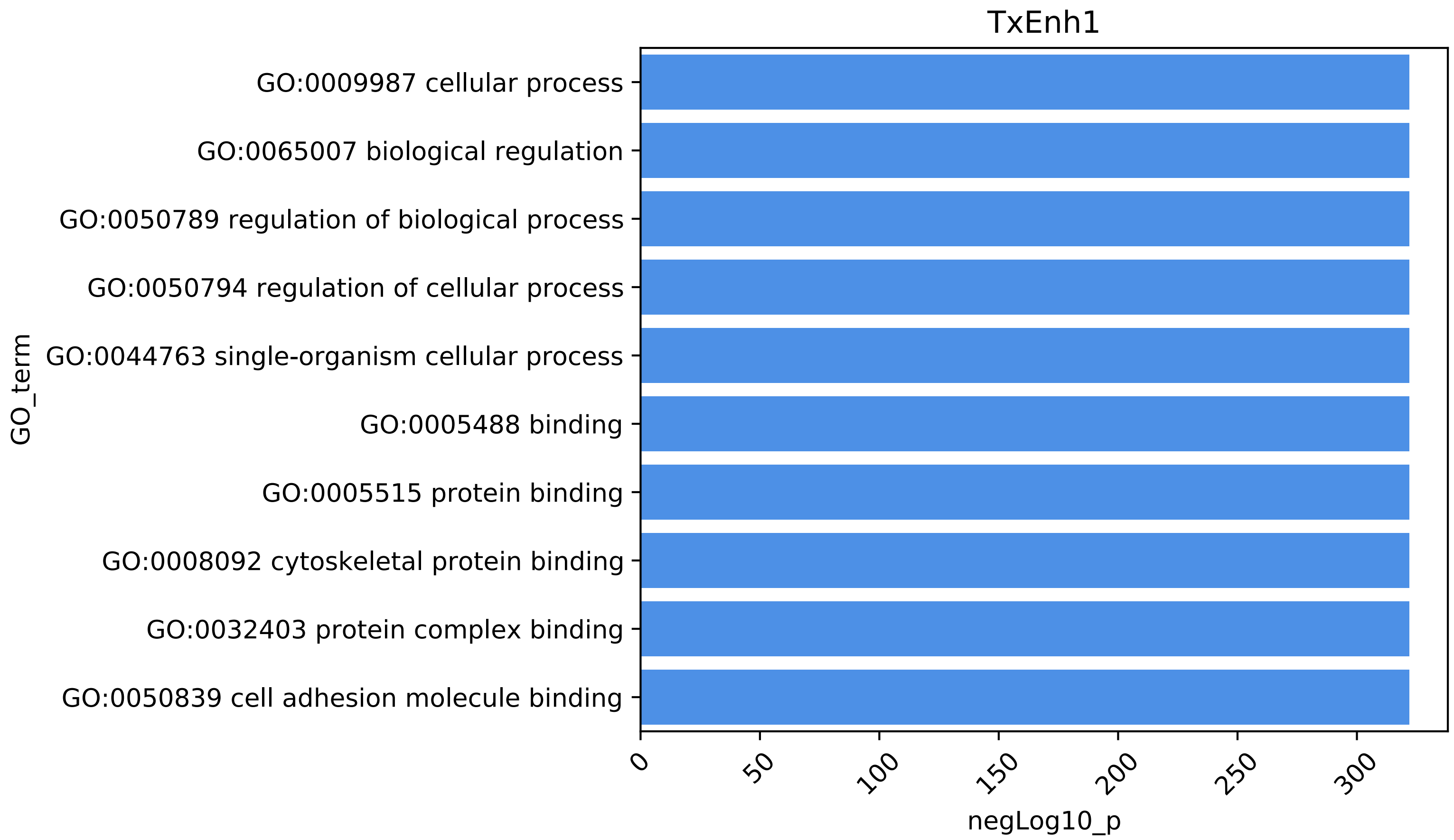

TxEnh1
GO:0009987 cellular process
GO:0065007 biological regulation
GO:0050789 regulation of biological process
GO:0050794 regulation of cellular process
GO:0044763 single-organism cellular process
GO:0005488 binding
GO:0005515 protein binding
GO:0008092 cytoskeletal protein binding
GO:0032403 protein complex binding
GO:0050839 cell adhesion molecule binding
GO_term
0
50
100
150
200
250
300
negLog10_p

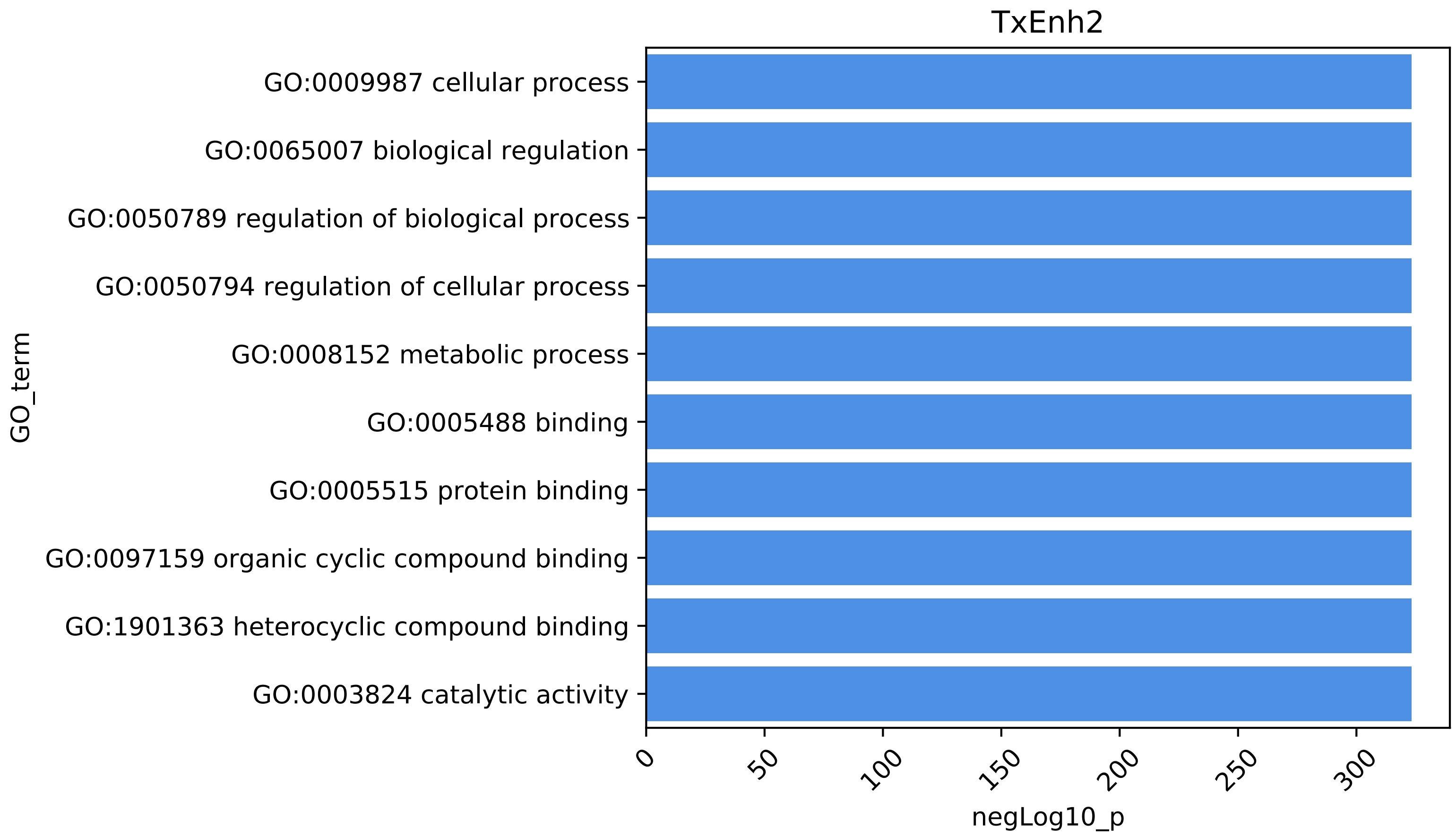
TxEnh2
GO_term
GO:0009987 cellular process
GO:0065007 biological regulation
GO:0050789 regulation of biological process
GO:0050794 regulation of cellular process
GO:0008152 metabolic process
GO:0005488 binding
GO:0005515 protein binding
GO:0097159 organic cyclic compound binding
GO:1901363 heterocyclic compound binding
GO:0003824 catalytic activity
0
50
100
150
200
250
300
negLog10_p

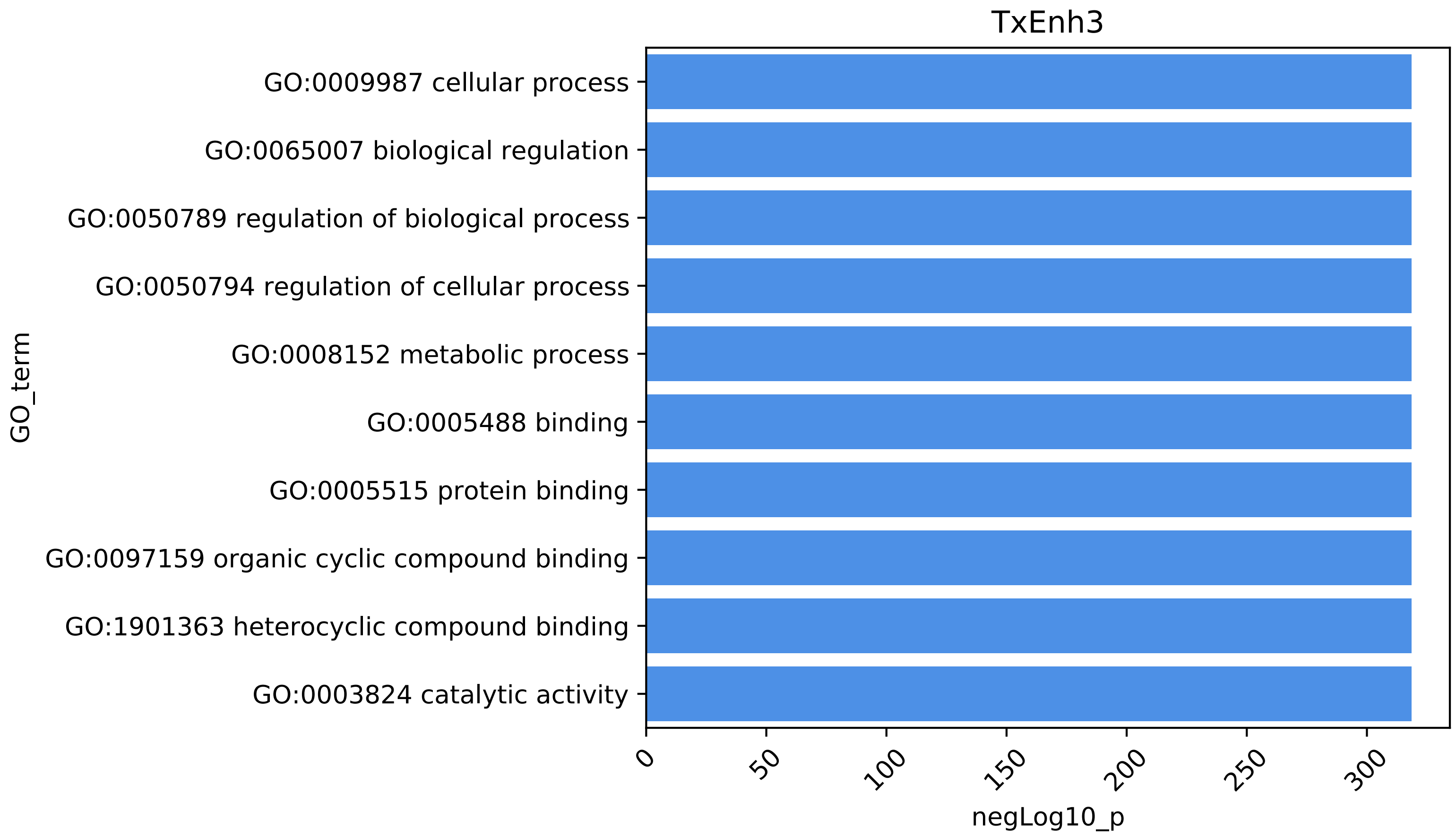
TxEnh3
GO_term
GO:0009987 cellular process
GO:0065007 biological regulation
GO:0050789 regulation of biological process
GO:0050794 regulation of cellular process
GO:0008152 metabolic process
GO:0005488 binding
GO:0005515 protein binding
GO:0097159 organic cyclic compound binding
GO:1901363 heterocyclic compound binding
GO:0003824 catalytic activity
0
50
100
150
200
250
300
negLog10_p

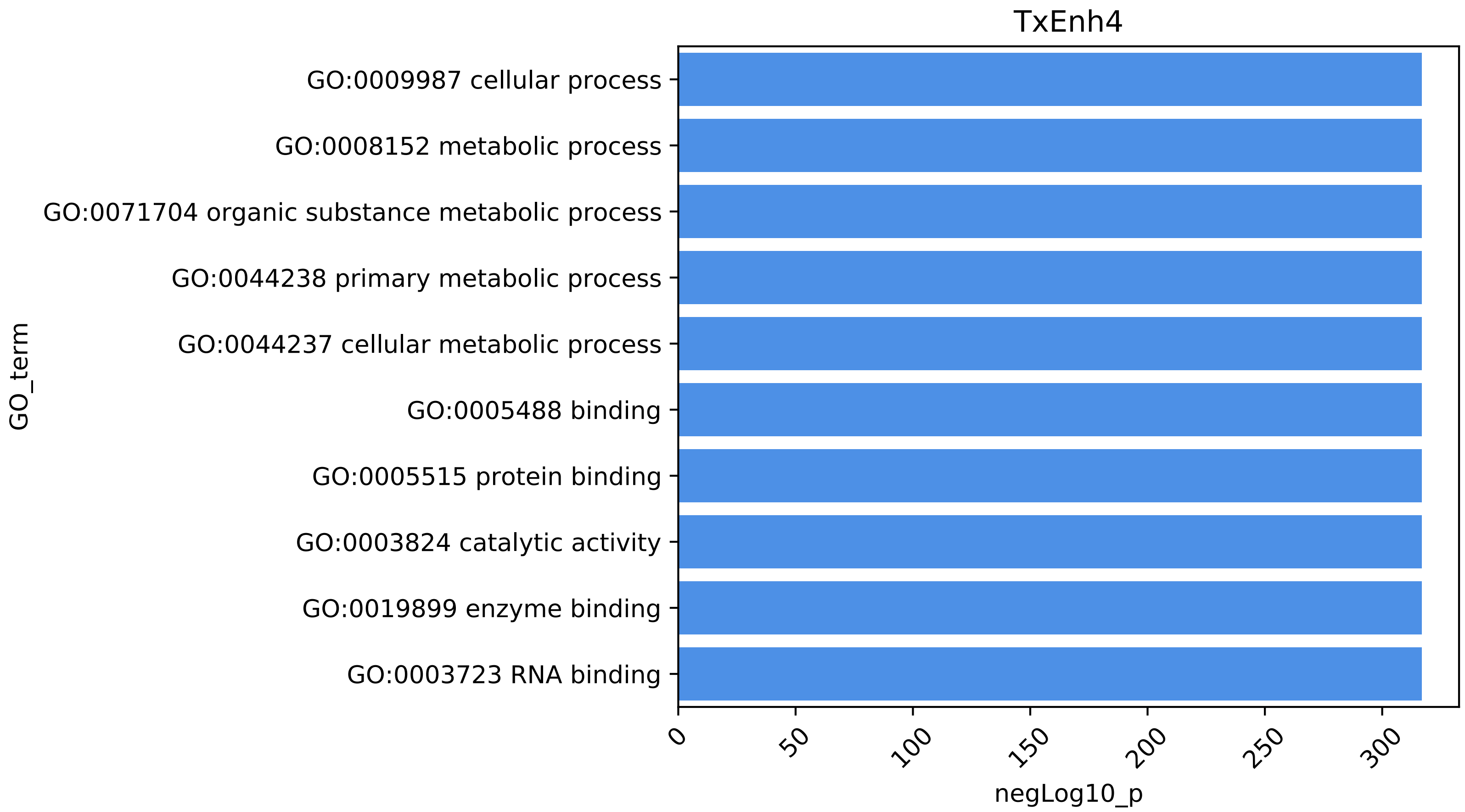
TxEnh4
GO_term
GO:0009987 cellular process
GO:0008152 metabolic process
GO:0071704 organic substance metabolic process
GO:0044238 primary metabolic process
GO:0044237 cellular metabolic process
GO:0005488 binding
GO:0005515 protein binding
GO:0003824 catalytic activity
GO:0019899 enzyme binding
GO:0003723 RNA binding
0
50
100
150
200
250
300
negLog10_p

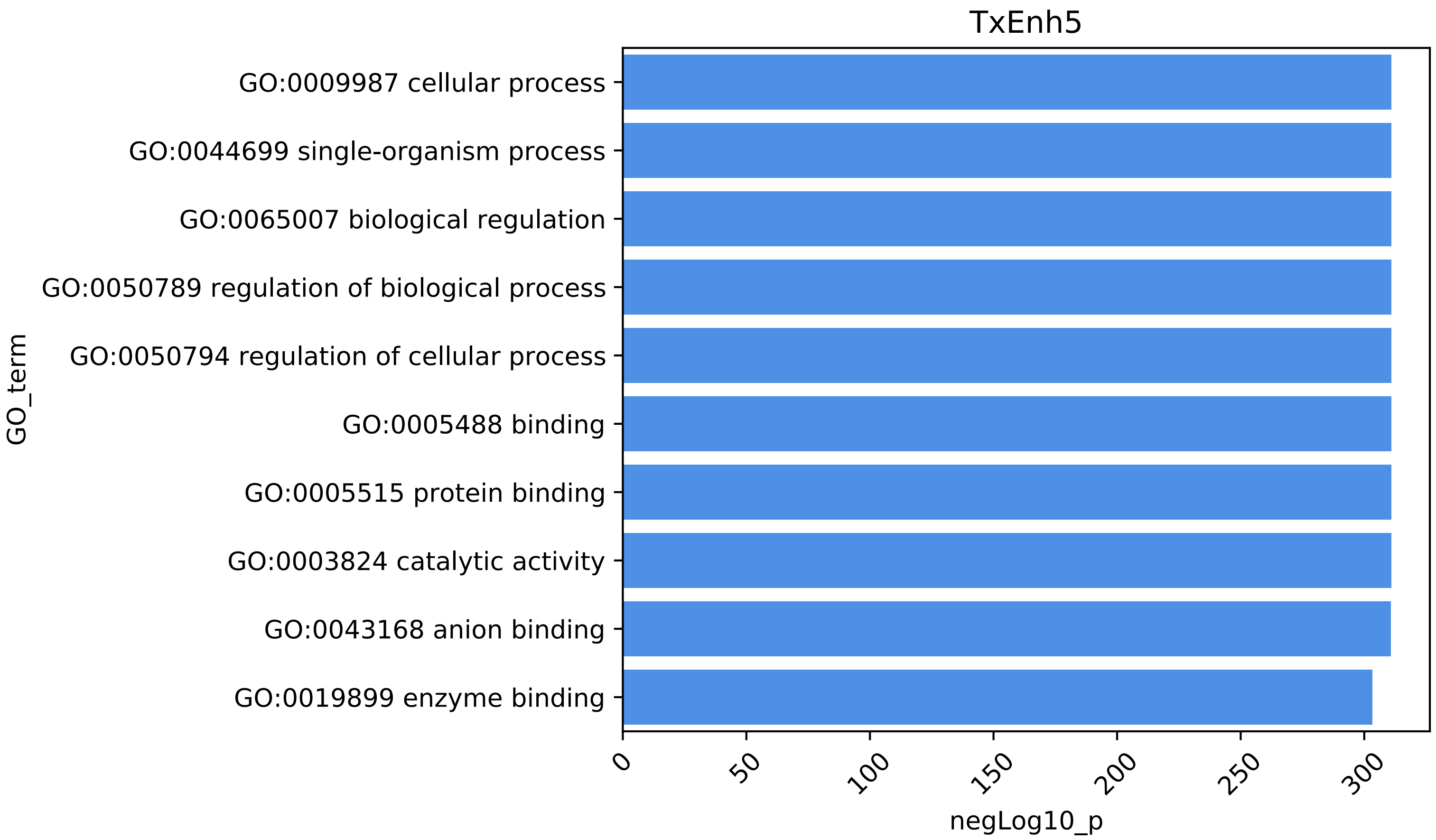
TxEnh5
GO:0009987 cellular process
GO:0044699 single-organism process
GO:0065007 biological regulation
GO:0050789 regulation of biological process
GO:0050794 regulation of cellular process
GO:0005488 binding
GO:0005515 protein binding
GO:0003824 catalytic activity
GO:0043168 anion binding
GO:0019899 enzyme binding
GO_term
0
50
100
150
200
250
300
negLog10_p

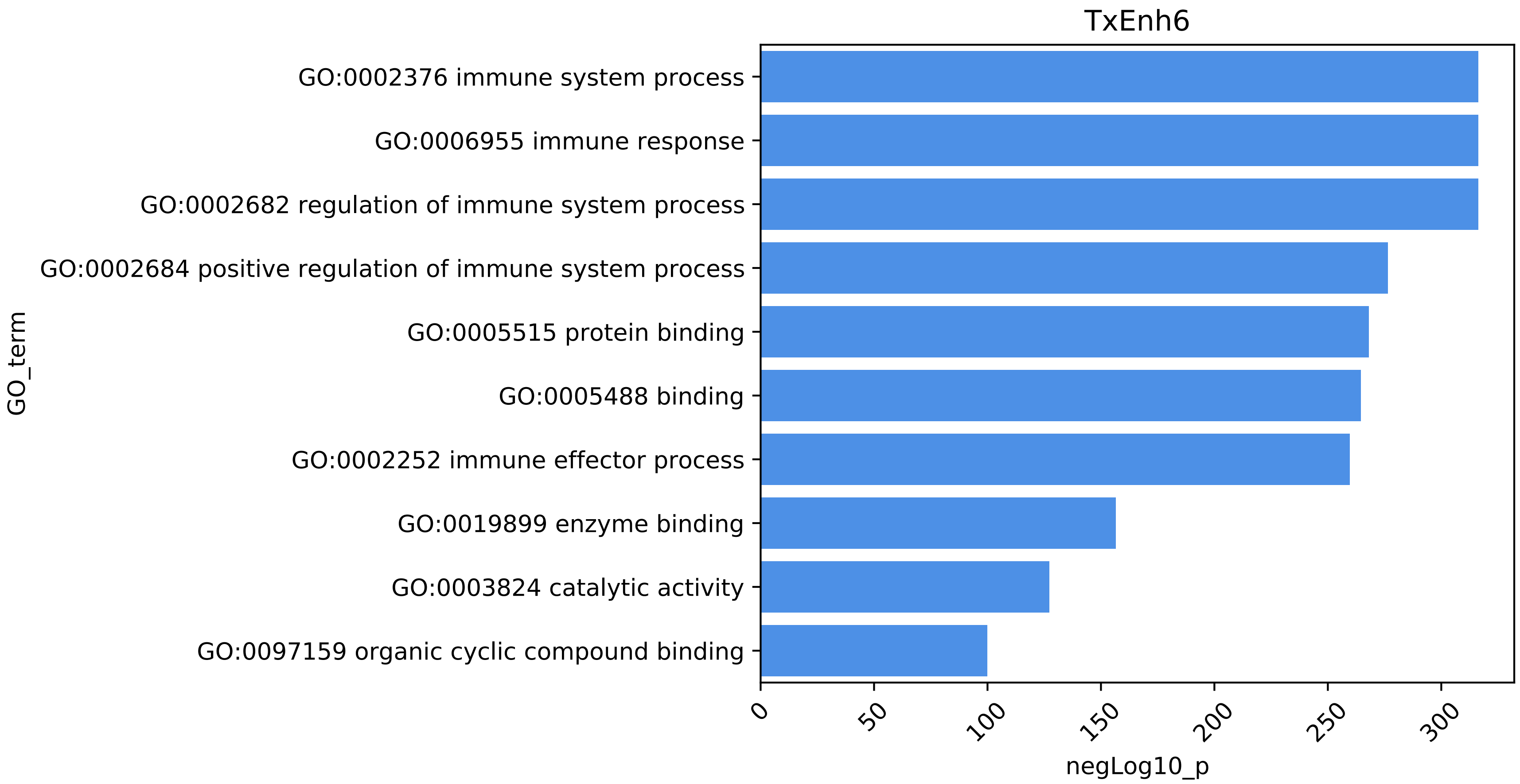
TxEnh6
GO:0002376 immune system process
GO:0006955 immune response
GO:0002682 regulation of immune system process
GO:0002684 positive regulation of immune system process
GO:0005515 protein binding
GO:0005488 binding
GO:0002252 immune effector process
GO:0019899 enzyme binding
GO:0003824 catalytic activity
GO:0097159 organic cyclic compound binding
GO_term
0
50
100
150
200
250
300
negLog10_p

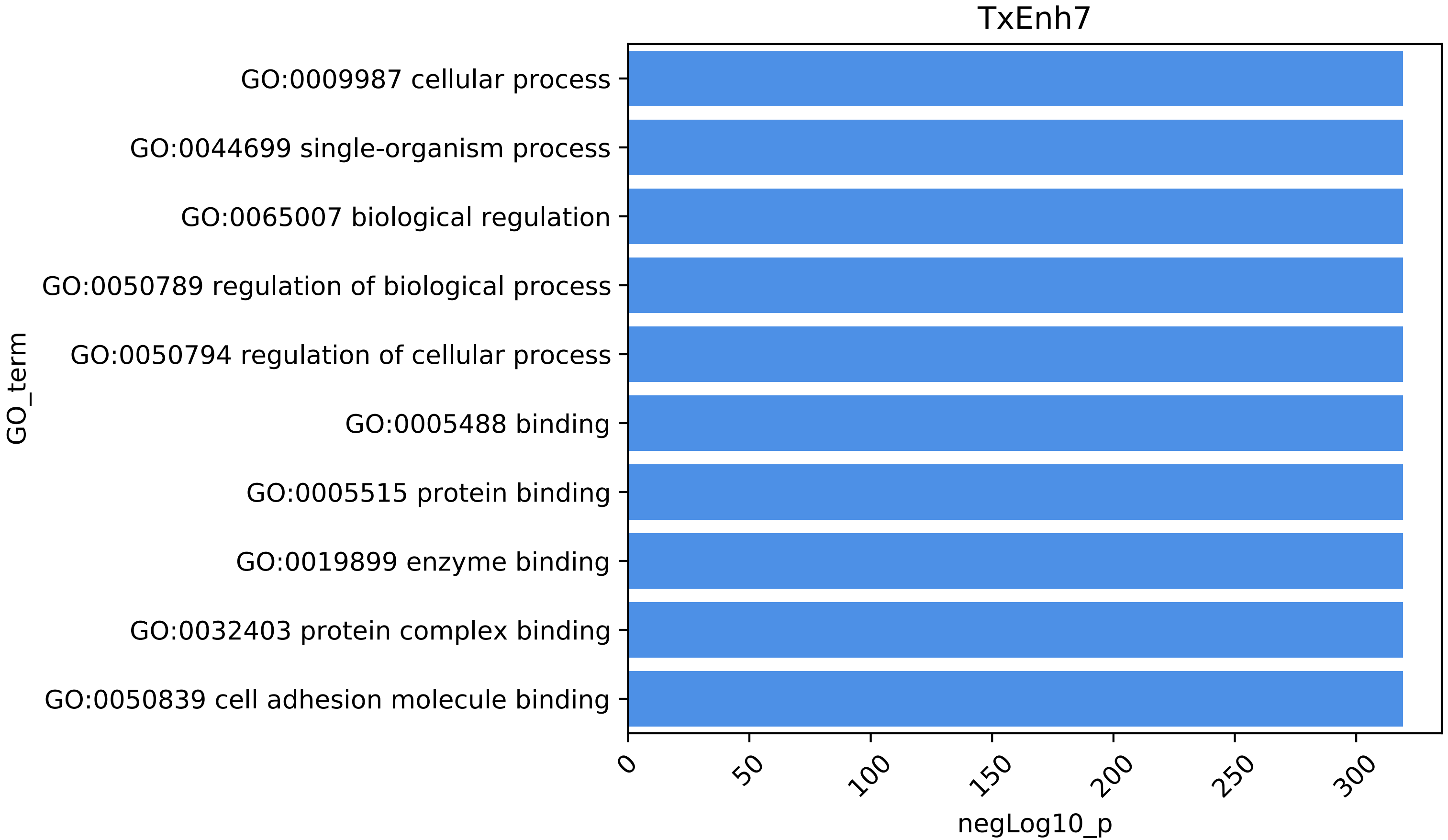
TxEnh7
GO_term
GO:0009987 cellular process
GO:0044699 single-organism process
GO:0065007 biological regulation
GO:0050789 regulation of biological process
GO:0050794 regulation of cellular process
GO:0005488 binding
GO:0005515 protein binding
GO:0019899 enzyme binding
GO:0032403 protein complex binding
GO:0050839 cell adhesion molecule binding
0
50
100
150
200
250
300
negLog10_p

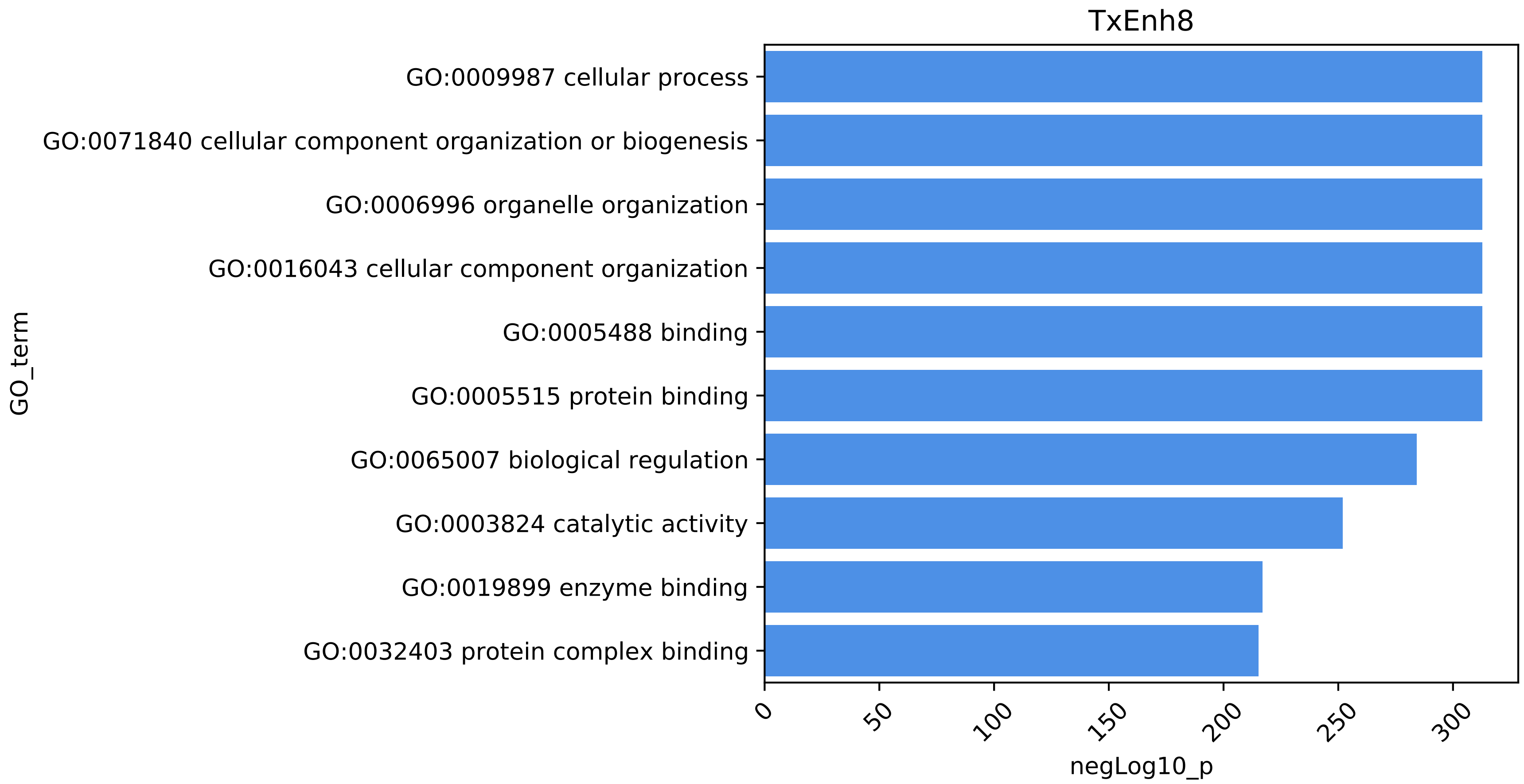

TxEnh8
GO:0009987 cellular process
GO:0071840 cellular component organization or biogenesis
GO:0006996 organelle organization
GO:0016043 cellular component organization
GO:0005488 binding
GO:0005515 protein binding
GO:0065007 biological regulation
GO:0003824 catalytic activity
GO:0019899 enzyme binding
GO:0032403 protein complex binding
GO_term
0
50
100
150
200
250
300
negLog10_p

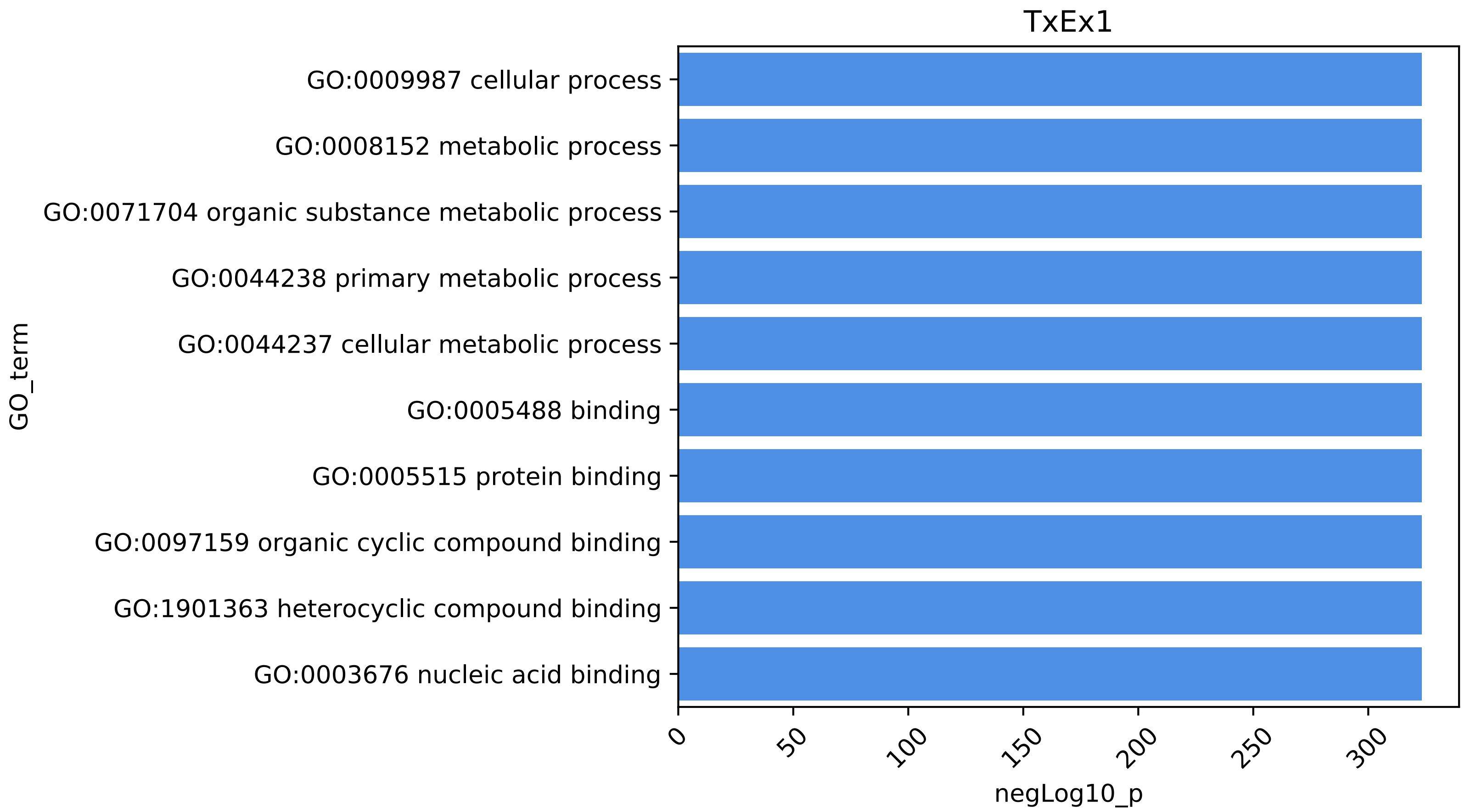

TxEx1
GO_term
GO:0009987 cellular process
GO:0008152 metabolic process
GO:0071704 organic substance metabolic process
GO:0044238 primary metabolic process
GO:0044237 cellular metabolic process
GO:0005488 binding
GO:0005515 protein binding
GO:0097159 organic cyclic compound binding
GO:1901363 heterocyclic compound binding
GO:0003676 nucleic acid binding
0
50
100
150
200
250
300
negLog10_p

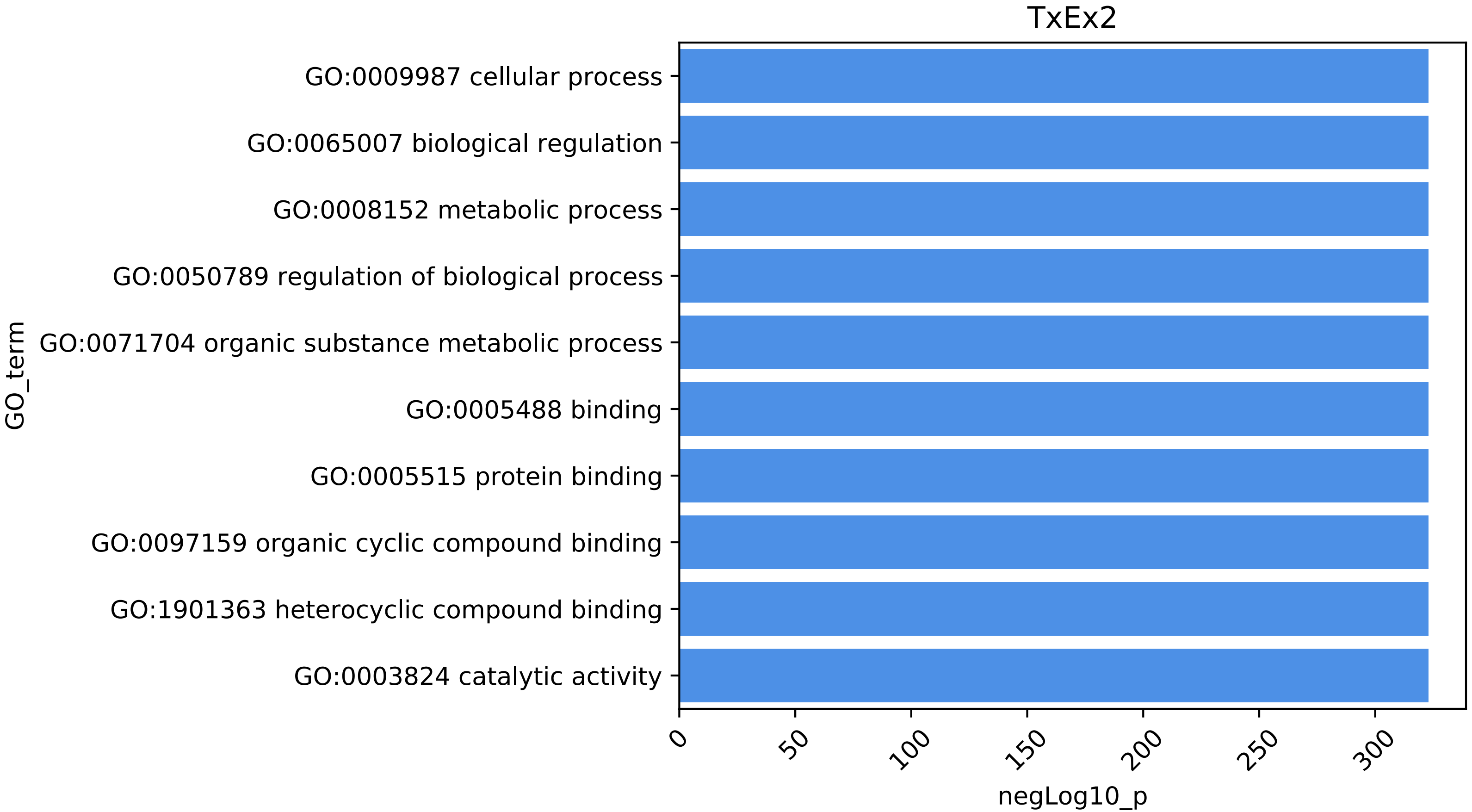

TxEx2
GO:0009987 cellular process
GO:0065007 biological regulation
GO:0008152 metabolic process
GO:0050789 regulation of biological process
GO:0071704 organic substance metabolic process
GO:0005488 binding
GO:0005515 protein binding
GO:0097159 organic cyclic compound binding
GO:1901363 heterocyclic compound binding
GO:0003824 catalytic activity
GO_term
0
50
100
150
200
250
300
negLog10_p

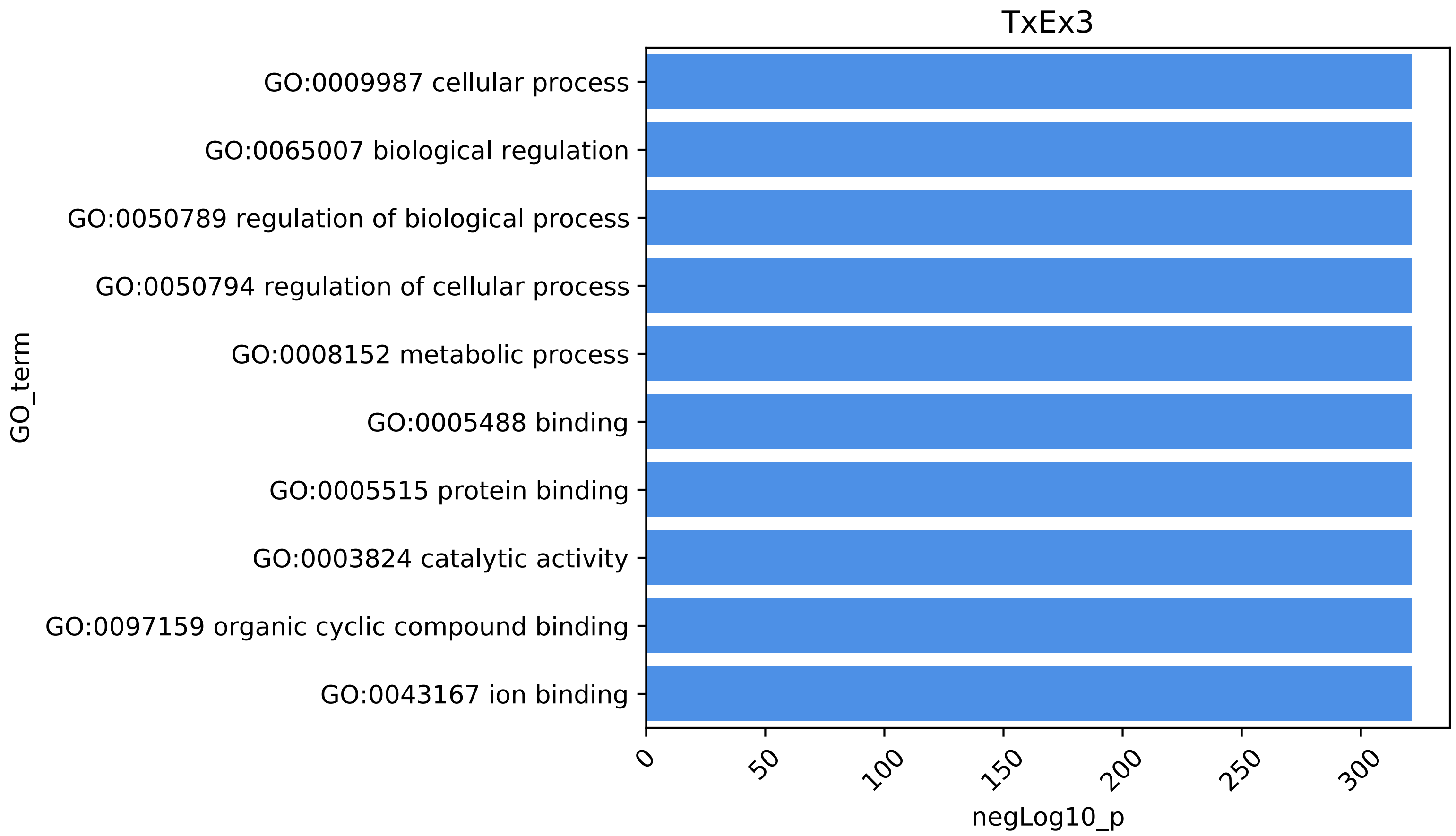
TxEx3
GO:0009987 cellular process
GO:0065007 biological regulation
GO:0050789 regulation of biological process
GO:0050794 regulation of cellular process
GO:0008152 metabolic process
GO:0005488 binding
GO:0005515 protein binding
GO:0003824 catalytic activity
GO:0097159 organic cyclic compound binding
GO:0043167 ion binding
GO_term
0
50
100
150
200
250
300
negLog10_p

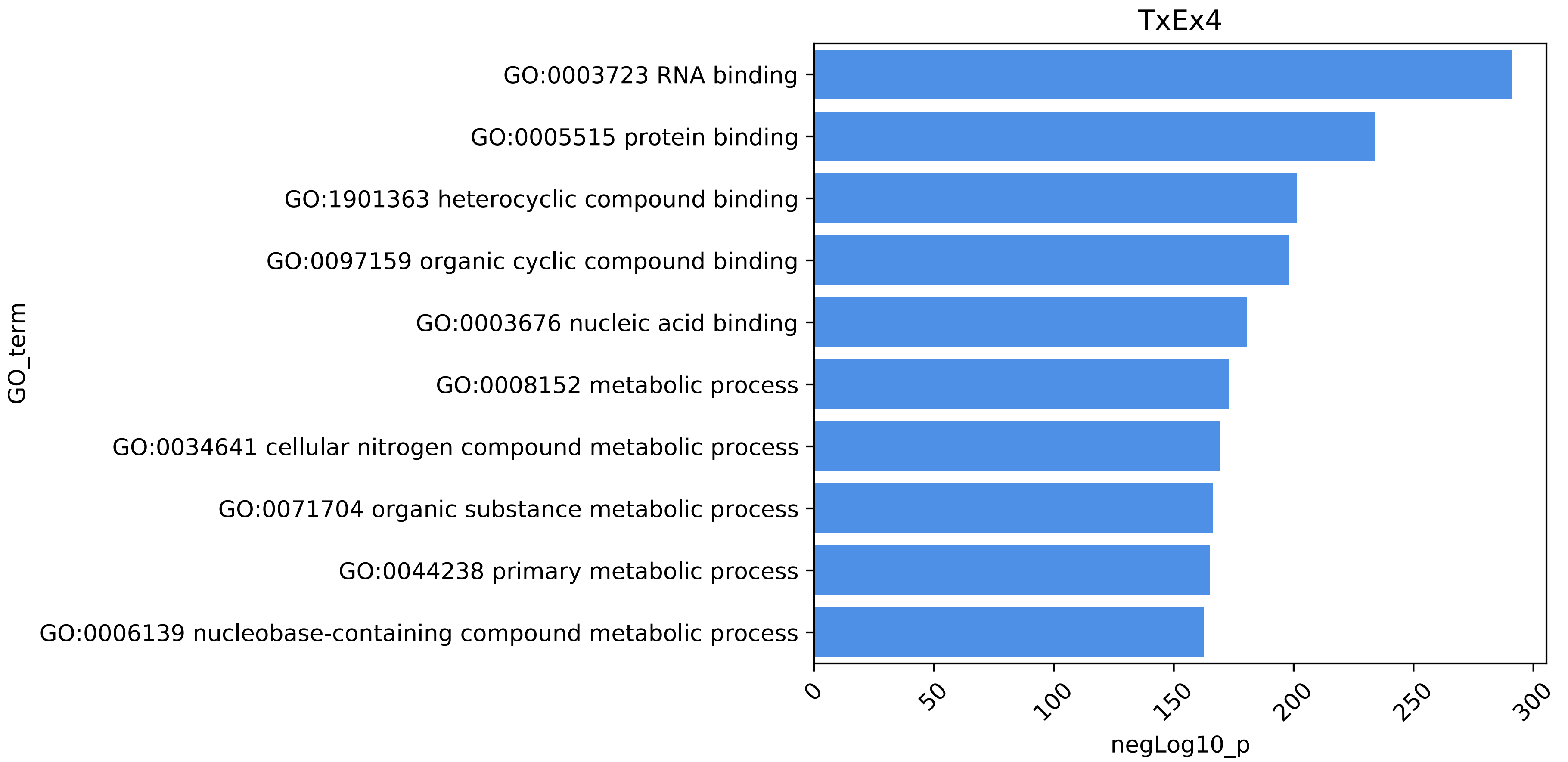
TxEx4
GO:0003723 RNA binding
GO:0005515 protein binding
GO:1901363 heterocyclic compound binding
GO:0097159 organic cyclic compound binding
GO:0003676 nucleic acid binding
GO:0008152 metabolic process
GO:0034641 cellular nitrogen compound metabolic process
GO:0071704 organic substance metabolic process
GO:0044238 primary metabolic process
GO:0006139 nucleobase-containing compound metabolic process
GO_term
0
50
100
150
200
250
300
negLog10_p

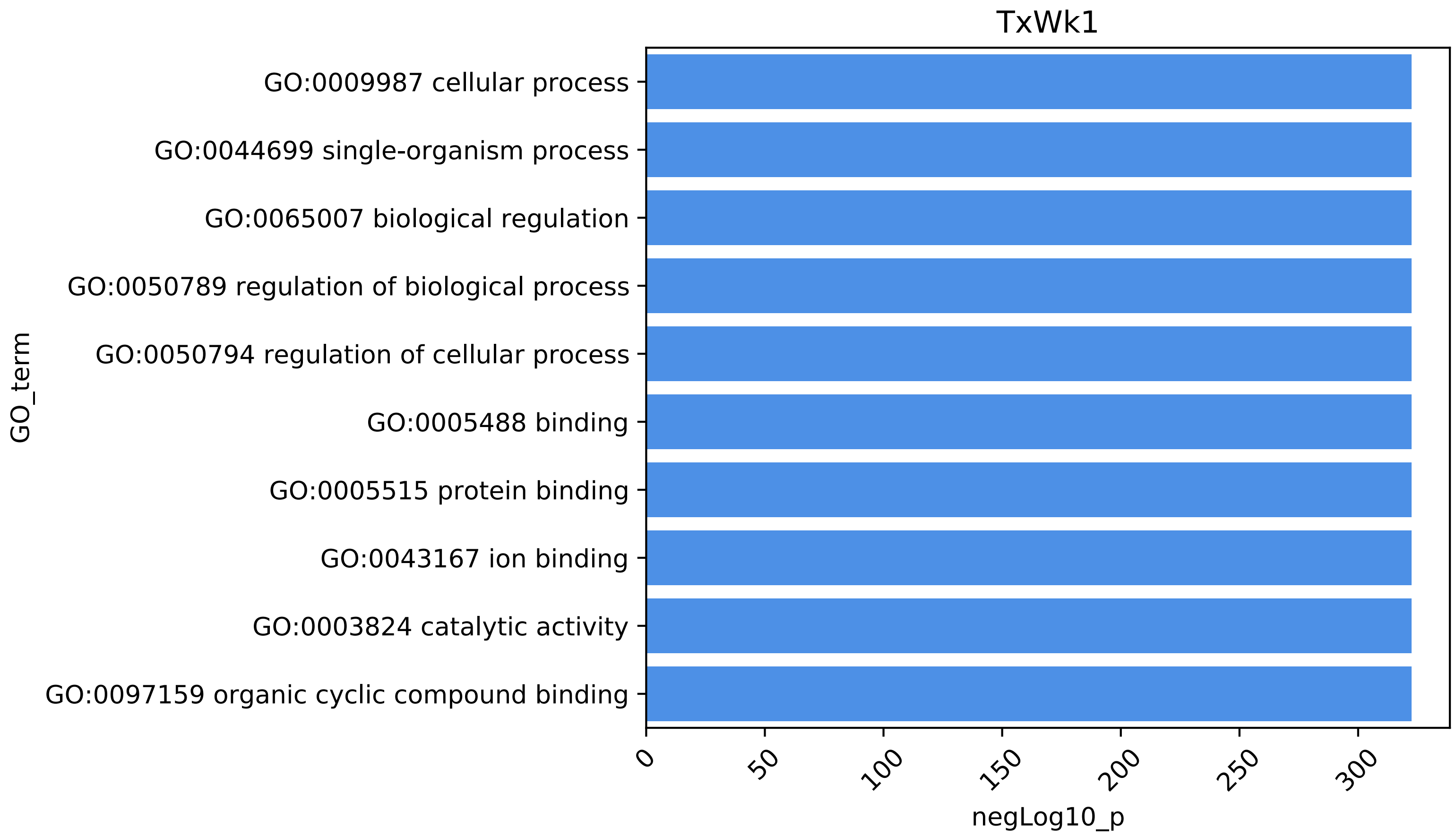
TxWk1
GO:0009987 cellular process
GO:0044699 single-organism process
GO:0065007 biological regulation
GO:0050789 regulation of biological process
GO:0050794 regulation of cellular process
GO:0005488 binding
GO:0005515 protein binding
GO:0043167 ion binding
GO:0003824 catalytic activity
GO:0097159 organic cyclic compound binding
GO_term
0
50
100
150
200
250
300
negLog10_p

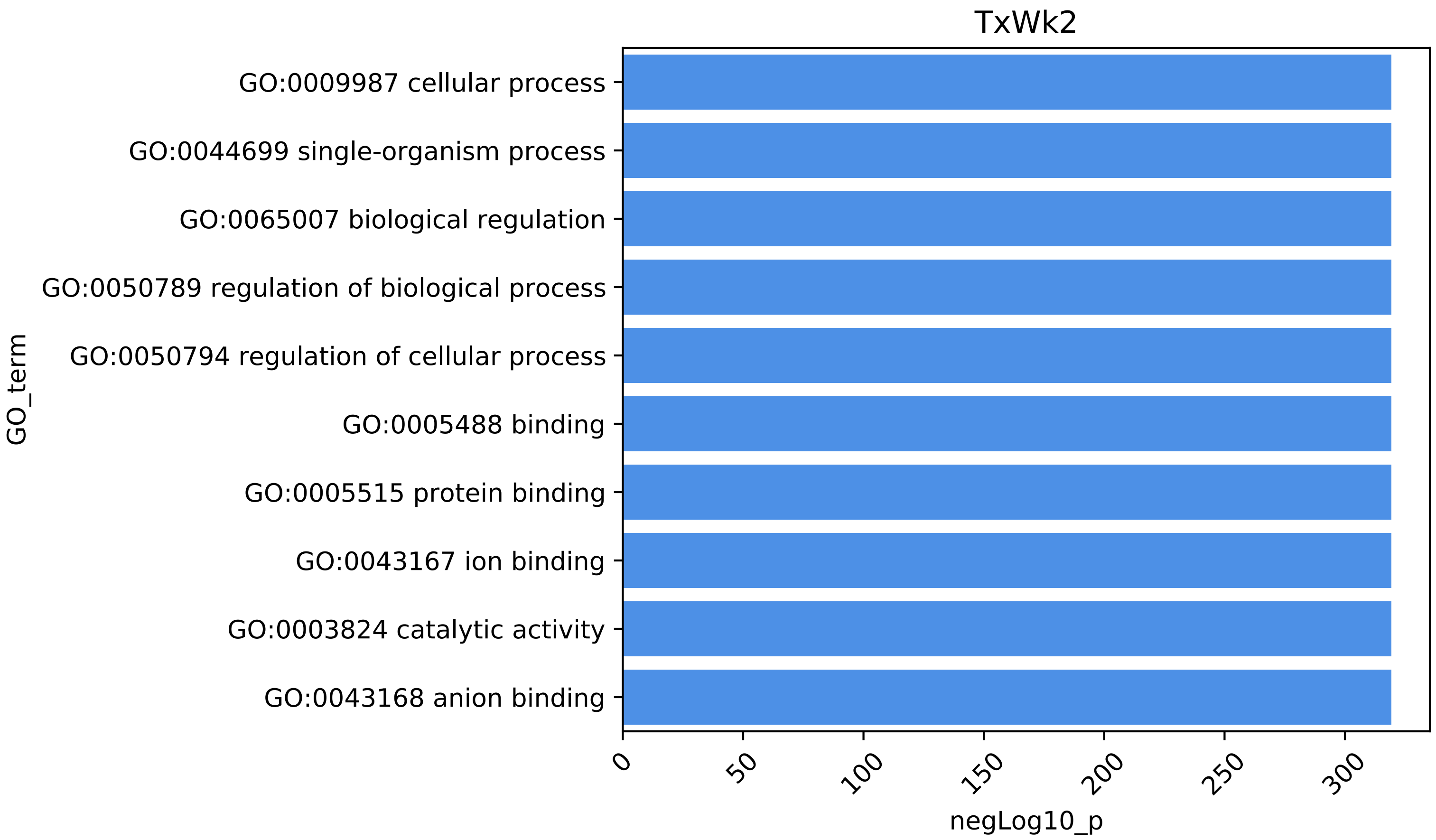
TxWk2
GO:0009987 cellular process
GO:0044699 single-organism process
GO:0065007 biological regulation
GO:0050789 regulation of biological process
GO:0050794 regulation of cellular process
GO:0005488 binding
GO:0005515 protein binding
GO:0043167 ion binding
GO:0003824 catalytic activity
GO:0043168 anion binding
GO_term
0
50
100
150
200
250
300
negLog10_p

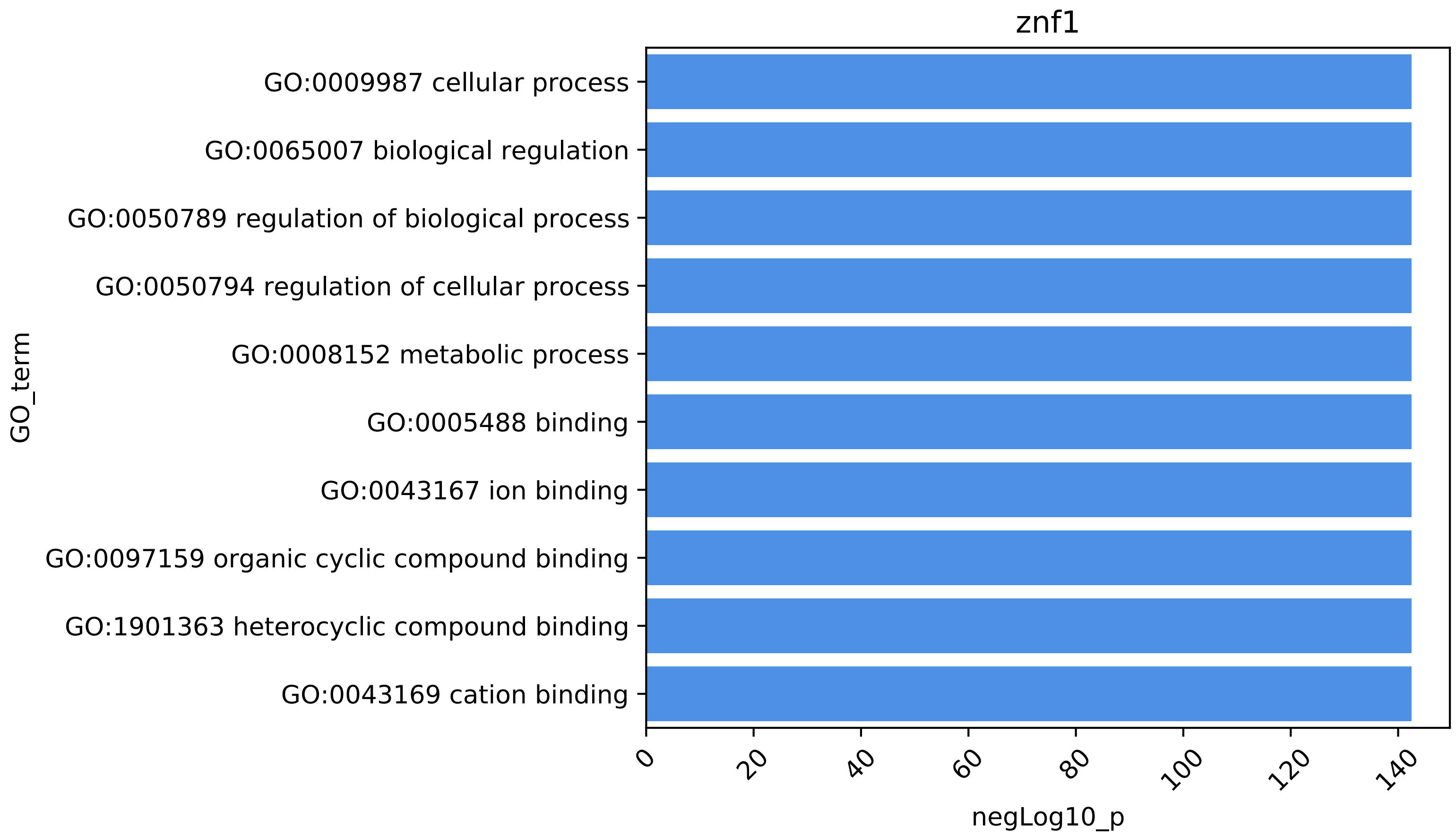
znf1
GO_term
GO:0009987 cellular process
GO:0065007 biological regulation
GO:0050789 regulation of biological process
GO:0050794 regulation of cellular process
GO:0008152 metabolic process
GO:0005488 binding
GO:0043167 ion binding
GO:0097159 organic cyclic compound binding
GO:1901363 heterocyclic compound binding
GO:0043169 cation binding
0
20
40
60
80
100
120
140
negLog10_p

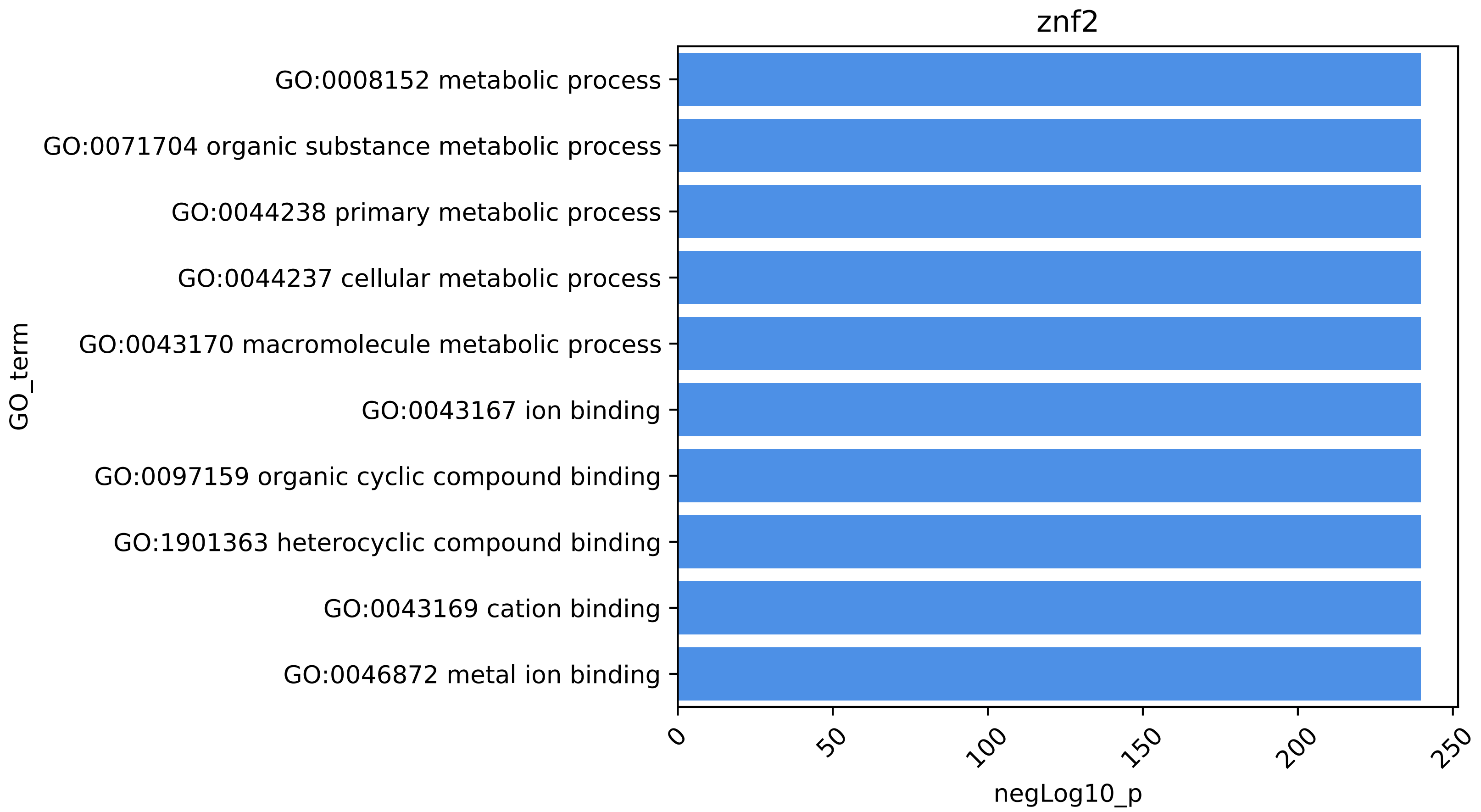
znf2
GO_term
GO:0008152 metabolic process
GO:0071704 organic substance metabolic process
GO:0044238 primary metabolic process
GO:0044237 cellular metabolic process
GO:0043170 macromolecule metabolic process
GO:0043167 ion binding
GO:0097159 organic cyclic compound binding
GO:1901363 heterocyclic compound binding
GO:0043169 cation binding
GO:0046872 metal ion binding
0
50
100
150
200
250
negLog10_p
