# Supplementary material for: Universal annotation of the human genome through integration of over a thousand epigenomic datasets
Source: Genome Biol. 2022 Jan 6;23:9. doi: 10.1186/s13059-021-02572-z (PMC8734071; doi:10.1186/s13059-021-02572-z)
Supplement: Supplementary file 10 — Additional file 10. Peer review history. [file 13059_2021_2572_MOESM10_ESM.docx]

**Review history**

**First round of review**

**Reviewer 1**

Manuscript review
Title: Annotating the human genome by integrating over a thousand epigenomic datasets
Authors: Vu & Ernst
Journal: Genome Biology

----------------------------------------------------------------------------------------------

The authors have constructed a large resource of chromatin state calls, using their previously published multivariate Hidden Markov Model (HMM) approach.
Here, they apply this modeling approach to learn chromatin states across 1000+ datasets simultaneous, in a so called "stacked" manner.
Although the general methodology of stacked modeling was proposed several years ago by Ernst & Kellis, the resulting dataset of chromatin state calls is new to this work.
My main comments are listed below, followed by a few minor issues and suggestions.

1. Stacked model learning
The use of stacked models on datasets of this size was previously not possible due to technical scalability issues.
Here, the authors have aimed to resolve these by learning chromatin state models through iterative sampling of 300 randomly chosen genomic regions of size 1 Mb.
Is this sampling approach also applicable to the learning of concatenation-based approaches?
And if so, does that require keeping track of sampled regions across input samples, to make sure the same sampled 200bp bins are used across (concatenated) biosamples?

2. Terminology of non-stacked models
At several points I was confused about the terminology used with respect to "cell-type-specific" models, assuming these referred to ChromHMM models learnt using data from a single biosample only.
Indeed, starting from the beginning of the manuscript, it seems that models trained on a single biosample are sometimes conflated with concatenation-based models.
For instance, it is hard to distinguish multi-biosample models from the ones described by the authors on line 832 and onwards, if both are referred to as "cell-type-specific".
This is particularly confusing when it comes to the interpretation of results.
For most analyses it the authors use (Roadmap) concatenation-based multi-biosample models, and for some they use models truely trained on individual biosamples.
Therefore I think a different terminology is in order, and I suggest the use of "cell-type-agnostic" (across multiple biosamples, in a concatenated manner) versus "cell-type-specific" (using a single biosample only).

3. Benefits of stacked models
The authors argue that post-hoc clustering of concatenated state models (as done by Ernst et al. 2011 and Kundaje et al. 2015) does not provide a "systematic and global view of the data",
without specifying exactly what this really means.
Throughout the manuscript the authors allude to the potential benefits of stacked models over models based on either concatenation of multiple cell types or individual cell types.
However, in my opinion, it remains unclear precisely what the benefits are and in which scenarios stacked models are preferred.

The manuscript contains several sections outlining enrichment levels of full-stack model states with a variety of other genomic and genetic annotations.
Although informative as a resource, these sections have not convinced me that non-stacked models are not / less well able to meaningfully pinpoint such annotations.
With stacked models, each genomic position is assigned a single chromatin state label, across all input biosamples.
However, they also add complexity (due to the increased number of states as compared to (concatenated) "cell-type-agnostic" models, and increased number of datasets as compared to "cell-type-specific" models).
It is not very clear how this increase in complexity is justified by a potential increase in Biological interpretation of states.

Regarding section "Full-stack states are more predictive of external annotations than cell-type-specific models":
ChromImpute and other related approaches have taught us that there is a lot of value in considering shared information across chromatin marks and cell types.
As such, it does not come as a big surprise that the full-stack model is better able to predict the occurrence of certain external annotations.
It does suggest that stacked models offer an added value similar to that of data imputation, in the sense that shared patterns are re-inforced and random noise is suppressed.
I wonder whether this could be the reason why for instance LMNB1 domains are better predicted using the full stack model than the 18-state concatenated model,
yet there are individual cell-type-specific models that perform better than the full-stack model (i.e. Fig 4b).
An important detail here is that these LMNB1 domains were profiled in Tig3 human lung fibroblasts, which are not (exactly) present in the set of 98 or 127 Roadmap samples.
What samples from these cell-type-specific models outperform the full-stack model? Fibroblast-related ones?

4. Stacked vs. concatenated models for characterizing dynamic state behavior
This pertains to section "Stacked Model Differentiates Cell-Type-Specific from Constitutive Activity":
The authors convincingly show that different full-stack states show up selectively for certain (groups of) cell types.
However, it is not clear to me exactly how a stacked model approach yields more meaningful information about activity specificity than a post-hoc analysis of concatenated model results.
In a way, a concatenated approach offers more flexibility to select for different levels of specificity (e.g. "x out of n samples are in state i"),
rather than being confined to a fixed number of full-stack model states.

5. Choice of number of chromatin states
This is a commonly recurring question in genome segmentation and clustering approaches, and I realize there is no definitive answer.
That said, I found the justification for the use of 100 states not particularly strong.
The authors tried out several numbers of states, and conclude that 100 is "best", although their reasoning would just as well support e.g. 80 states.
What does the number of states mean in terms of downstream results? Does it even matter in practice?
What guidance can they give end users regarding this matter? It would be good to discuss this more.

Also, since each chromatin mark is expected to play a role (or at least, occur) roughly equally often in each cell type (with some exceptions, e.g. PSCs),
I wonder whether the distribution of chromatin marks over full-stack states can be used as a guideline to choose a more optimal number of states.
For instance, one would expect that each general "group" of cell types (e.g. "Brain") has some set of group-specific enhancers, which would ideally be captured by their own state (at least one).
If enhancer states only capture a few such cell type groups, perhaps the number of states is too low, whereas if they capture similar patterns "spread out" across groups, the number of states might be too high.
I imagine this is also a function of the input datasets, e.g. if 90% of input datasets are "Brain", that group will likely be favored over others?

6. The role of data imputation
Given the excellent work by Dr. Ernst on data imputation (i.e. ChromImpute), its role cannot be ignored.
One of the "features" of full-stack models mentioned is essentially that the "data matrix" does not need to be complete for all marks and all cell types.
This, combined with the earlier demonstrated often superior quality of imputed data, begs the question: why not use imputed data to learn full-stack models?
Would this add much or, since both imputation and the current full stack models are based on the same "observed" data, are the stacked models sufficient?

I think rather than going into great depth and detail on the various genomic annotation enrichment analyses (mentioned above),
it might be more insightful to do a head-on comparison of concatenated vs. stacked models based on complete (possibly imputed) datasets, using the exact same input data.

7. Extendability to new datasets and establishing standard annotations
Concatenated models offer an attractive opportunity to add additional biosamples at a later time, by re-using an existing model and calling states on unseen (but identically processed) data.
Because of the nature of data stacking, what possibilities do stacked models offer in this area?
This is an important point for establishing a set of "common" chromatin states in the community that can be used as a reference point for additional datasets.
What are the recommendations the authors can give the community in terms of choosing an appropriate chromatin state modeling approach?
Given the availability of increasing numbers of datasets (10s to 100s of thousands within the next few years), are stacked models the preferred approach?

In conclusion:
The authors have demonstrated, for the first time, the use of a stacked chromatin state modeling approach on 1000+ genome-wide datasets.
Although interesting in principle, it remains unclear what the actual practical benefits are in terms of Biological interpretation.

Minor points:
- In lifting over 200bp bin coordinates from hg19 to hg38, the authors find that 1,186,379 200bp segments were not lifted over.
That number seems very high and I'm curious to hear what liftOver parameters were used, i.e. requiring perfect mapping of 200bp coordinates, done in a reciprocal manner between the two assemblies?
- Supplementary Figure 8: labels are shown upside down. Legend: reference to EnhA7 state should be EnhA8.
- Supplementary Figure 12: since there is still significant amounts of DNase signal in the 'Closed' state, this should be worded differently.

**Reviewer 2**

The authors provide a full-stack ChromHMM annotation across 127 human samples, which gives a unified view of ~1,000 epigenomic data sets. With their detailed characterization of 100 chromatin states, which are clustered into 16 state groups, some state groups show signals across all cell types while other state groups such as enhancers show cell type specificity. While the full-stack model agrees with previously published cell-type-specific chromatin states, it also provides more detailed annotations on repeat elements, structural variation, sequence conservation, and genetic variation.

Overall, this full-stack chromatin state annotation has advantages over the cell-type-specific annotations in pan-tissue-type applications that do not require detailed chromatin state annotation in a particular cell type. These full-stack models are difficult to build and annotate, and the authors did a good job validating the quality of the model using the extensive infrastructure built up with their earlier work for the Epigenomic Roadmap project. Therefore, I suggest that the paper be accepted for publication after responding to comments below.

Major comments:
It is the underlying assumption of ChromHMM that each genomic bin can only be assigned to one chromatin state. When we consider the concatenated model, for the genomic bins that are in one state (say, TSS, the gene is expressed) in one sample but in another state in another sample (say, ReprPC, the gene is repressed), and in a third state in yet another sample (say, Bivalent TSS, the gene is poised for expression), what would this genomic bin be assigned to in the full-stack model? Can the authors perform a detailed analysis on this subset of genomic bins so that the reader can get a better understanding of the full-stack model?
In Fig1, the authors showed two loci of full-stack modeling annotations. It is indeed an advantage of the full-stack model to provide a big-picture view of the epigenomes, as illustrated by these two loci. However, there are many genomic regions with frequent state transitions as shown by the concatenated approach; can the authors provide two examples of such regions and illustrate their state assignment in the full-stack model?
Are different TSS states (TSS1-2, BivProm1-4) in the full-stack model associated with different classes of genes? Can the author perform GO enrichment for each subset of genes?
The paper provides many numbers for enrichment, but very often, it is unclear whether the enrichment has any biological significance. For example:
Line 130-131: Acetylation is an informative histone modification. For those regions assigned as acetylation groups, do you have any biological explanation? What's their role in gene regulation?
Line 138-139: "A DNase group consists of one state (DNase1) with strong emission of only DNase in all profiled cell types." What's the biological function of the DNase group since other active states are also expected to have high DNase signals?
In Fig 3A, Why are the TSS enrichments in PromF4 and PromF5 so high (62.2 and 31.9, respectively), even higher than BivProm1-4 (enrichment 8-28)? Would these two PromF states actually be a weak TSS state instead of flanking? Or are they actually located in gene-dense regions with many TSSs?
Line 228-229: "Acet1, a state associated with various acetylation marks and H3K9me3 had a 23-fold enrichment for simple repeats (Supp. Fig. 20)." What is the biological implication of this enrichment?
Line 231-232: "States in the transcription start site group, TSS1-2 were most strongly enriched with tRNA and low complexity repeat class 232 (~10-60 fold) (Supp. Fig. 20)." What does this mean biologically?
Line 233-237: "We also saw specific states associated with the largest repeat classes of the genome, SINEs, LINEs, and LTRs. SINE repeats were most enriched in state Tx5 (3.7 fold) (Fig. 5C), which had high emission of H3K36me3 (Fig. 2A-B, Supp. Fig. 4-5). LINEs and LTRs repeats were most enriched for states in the H3K9me3-associated heterochromatin group with LINE most enriched in HET3 (3.4 fold), while LTRs were most enriched in HET5 (4.7 fold)" What's the biological explanation for these specific states enriched with specific repeats? Are there any published analyses that support these enrichments?
Line 271-278: "This is consistent with previous analysis using cell-type-specific annotations showing that ConsHMM state 100 was enriched in a ZNF gene-associated chromatin state [32]. Interestingly though, another full-stack state (ZNF2) that was even more strongly enriched for ZNF genes (68.6 folds), had 0.4 fold enrichment for ConsHMM state 100, and instead was most enriched with ConsHMM state 1 (Fig. 3A- B, Supp. Fig. 22). Therefore, the full-stack annotation helped distinguish two ZNF-gene associated states that are associated with distinct conservation states." Indeed ZNF1 and ZNF2 differ in their enrichments with ConsHMM state 100, but what does this result mean biologically? Do ZNF1 and ZNF2 belong to different classes of ZNF proteins with different biology?
Line 288-290: "Consistent with those previous results, using the full-stack model, we observed that of the 13 states that were in the top 10 maximally enriched states with either deletions or duplications (1.18 fold or greater), seven were in the heterochromatin group (HET1-2,4-7,9) and one was in the ZNF gene state (ZNF2)". Why are these states enriched in deletions or duplications? Any biological explanation? Or do you mean these regions have annotation artifacts?
Line 329-331: "Some other scores, however, showed relatively weak enrichments or even depletions for these promoter- and TSS- associated states." Why do these other scores have lower enrichment for promoters?
Line 335-344: "Enhancer states EnhA2-3,17 were among the states in the top five most enriched for FATHMM, GERP++, LINSIGHT, PhastCons, and PhyloP prioritized non-coding variants. These states also showed consistent enrichments with variants prioritized by Eigen, funSeq2, CADD, and REMM, though those scores showed even stronger relative enrichments for promoter states. In contrast, FIRE, DANN and CDTS were depleted for prioritized variants in all these enhancer states, and Eigen_PC showed both enrichments and depletions (Fig. 6C). FIRE and fitCons showed strong enrichment for exon states (TxEx1-3), which are associated with coding regions, even though coding bases were excluded in this analysis (Fig. 6C). FATHMM had the greatest relative enrichment for the primary DNase state associated with CTCF cell type-specific chromatin states (DNase1) (~10 fold), and was the only score for which this state was among the top five most enriched states (Fig. 6C, Supp. Fig. 28)." What do these different enrichment biases mean? Do these means promoter/enhancer/exon are with different conservation patterns biologically or some technically score bias?
Line 370-371: "TSS and promoters associated states, PromF4 and TSS1- 2, were maximally enriched for variants in the lowest range of MAF (0 < MAF <= 0.0001), 1.5-1.7 fold." Why would these highly functional states be enriched in rare variants? Shouldn't these states be depleted in rare variants? As shown in Fig. 3A, these states are highly conserved evolutionarily and, as a corollary, one would think they should be depleted of rare variants.
Line 424-445 and FigS39: why are the H3K9me3 states and GapArtf states so enriched in cancer-associated variants, much higher than enhancer/promoter states? Any biological reasons? One would think that states that are of regulatory functions (e.g., enhancers and promoters) should be enriched in cancer-associated variants.
The comparison of full-stack and concatenated models on predicting external genomic annotations is like attacking a strawman. For example, in predicting annotated exons (Fig. 4A), the full-stack annotation would obviously do better than annotations in individual samples since only a subset of genes is expressed in a particular sample. But what does this prove? It is hardly a useful application to predict exons using chromatin states since the former is better annotated than the latter. I suggest the authors tone down the expectation in this section and keep Figure 4 in Supplementary Materials.
The authors performed all the segmentation in hg19 and then lifted-over chromatin annotations in each 200-bp bin to GRCh38. Currently, almost all annotations are done on GRCh38. In order for the full-stack annotations to be a more useful resource, can the authors also run the whole pipeline in GRCh38 from reads alignment to segmentation? Please also provide a comparison between the state annotations in hg19 and GRCh38 to show whether the full-stack modeling is sensitive to a change in the version of the reference genome.

Minor changes:
Line 57-60: "Variants of this approach allow information from other cell types to influence the state annotations in one cell type at a position, but still produce cell-type-specific state annotations [26,27]. These models that produce cell-type-specific annotations are natural for cell-type-specific analyses." These sentences are confusing. What do you mean by "other cell types to influence one cell type ... still produce cell type-specific annotations"? What is "natural" about the analyses?
Line 80-83: The authors compared the number of states (100) in the full-stack model to the number of states theoretically possible (M^K or even M*K) in a concatenated model, where M = 15 or 18 and K = 127. This comparison indicates that the total number of states in the full-stack model (100) is very small. Does that mean that most of the states in the concatenated models are not occupied? Can the authors show this using the enhancer states as an example? How many genomic bins belong to one of the enhancer states in the concatenated model and which states do these genomic bins take on in the full-stack model?
The authors described the improvement on scalability for ChromHMM which makes full-stacked modeling possible. How does computing time grow with the number of input tracks? How does computing time grow with genome size?
The assembly gap states seem to be new for the full-stack modeling. Why were such states not identified previous by concatenated modeling?
Line 148-149: "States in the transcription groups (TxEnh1-8, TxWk1-2, Tx1- 8, TxEx1-4) were all at least 2.1 fold enriched for annotated genes, which covered 88.8-97.5% of the states." What do you mean by "which"? By annotated genes, I assume you mean exon plus intron positions. Which genome annotation are you using? In total, what fraction of the genome does "annotated genes" cover?
How is the color scale in Fig 3A defined? It seems to differ across the columns; some small enrichments (like 2.2 in RefSeqGene column) are in bright red while other even higher enrichments (like 62.2 TSS enrichment in PromF4) are in light red.
Line 223: the cited supplementary figure seems wrong. Should it be Fig. S20 & S21?

**Authors’ response to reviewers**

Reviewer reports:

Reviewer #1:

Manuscript review

Title: Annotating the human genome by integrating over a thousand epigenomic datasets Authors: Vu & Ernst

Journal: Genome Biology

*The authors have constructed a large resource of chromatin state calls, using their previously published multivariate Hidden Markov Model (HMM) approach.*

*Here, they apply this modeling approach to learn chromatin states across 1000+ datasets simultaneous, in a so-called "stacked" manner.*

*Although the general methodology of stacked modeling was proposed several years ago by Ernst & Kellis, the resulting dataset of chromatin state calls is new to this work.*

*My main comments are listed below, followed by a few minor issues and suggestions.*

We thank the reviewer for the summary. Yes, while the stacked modeling approach had been previously proposed, a large-scale application of the approach had not been previously demonstrated.

We also thank the reviewer for the comments and suggestions, which we have used to strengthen and clarify the manuscript. We provide our response to each individual comment below.

1. *Stacked model learning*

*The use of stacked models on datasets of this size was previously not possible due to technical scalability issues.*

*Here, the authors have aimed to resolve these by learning chromatin state models through iterative sampling of 300 randomly chosen genomic regions of size 1 Mb.*

*Is this sampling approach also applicable to the learning of concatenation-based approaches?*

We thank the reviewer for this question. Yes, this sampling approach is also applicable for learning chromatin state maps in the concatenation-based approach. We have found less of a reason to use it for the concatenated approach, since typically the number of inputs and states will be fewer in concatenated models, and the concatenated approach can more easily parallelize over a greater number of cores without hitting memory limits. We also note that in addition to improving the time and memory scalability of ChromHMM, we had also improved the numerical scalability for cases with a large number of inputs as we considered here (using the ‘-many’ flag option we added in ChromHMM).

*And if so, does that require keeping track of sampled regions across input samples, to make sure the same sampled 200bp bins are used across (concatenated) biosamples?*

We thank the reviewer for this question. In applying the concatenated approach to multiple biosamples, there is no constraint that the same 200bp bins are used across biosamples in each training iteration. However, the output model would be used to annotate every 200bp in the genome in every biosample. We believe that allowing different positions to be sampled from different biosamples would be a more effective

training strategy for the concatenated approach because in each iteration, the model would be expected to use a more representative subset of the entire genome from multiple input biosamples.

In response to these comments we edited the first paragraph in the section ‘Methods/ **Training full-stack model and generating genome-wide state annotations**’ to now state:

“We note that this sampling procedure can also be applied to learn concatenated models, in which case there would be no requirement that the same segments are sampled in each cell type. However, sampling can be unnecessary for typical instances of learning concatenated chromatin state models, given that it usually involves fewer different inputs to the models, fewer number of states, and in training these models, ChromHMM is able to tolerate more parallel cores without reaching memory limits.”

We have added to the supplementary information a discussion of the asymptotic growth of time and memory (also in response to a comment from Reviewer 2).

2. *Terminology of non-stacked models*

*At several points I was confused about the terminology used with respect to "cell-type-specific" models, assuming these referred to ChromHMM models learnt using data from a single biosample only.*

*Indeed, starting from the beginning of the manuscript, it seems that models trained on a single biosample are sometimes conflated with concatenation-based models.*

*For instance, it is hard to distinguish multi-biosample models from the ones described by the authors on line 832 and onwards, if both are referred to as "cell-type-specific".*

*This is particularly confusing when it comes to the interpretation of results.*

*For most analyses it the authors use (Roadmap) concatenation-based multi-biosample models, and for some they use models truely trained on individual biosamples.*

*Therefore I think a different terminology is in order, and I suggest the use of "cell-type-agnostic" (across multiple biosamples, in a concatenated manner) versus "cell-type-specific" (using a single biosample only).*

We thank the reviewer for raising this point and we apologize for the confusion in terminology. We now use the terminology ‘per-cell-type chromatin state annotations’ or ‘per-cell-type annotations’ to refer to chromatin state ***annotations*** that are on a per-cell-type basis, which includes those from models learned both by the concatenated and independent approaches. Where it is relevant to distinguish whether the ***models*** that produced the per-cell-type annotations were generated based on a concatenated or independent approach, we use the ‘concatenated’ or ‘independent’ terminology along with ‘per-cell-type’. Our concern with adopting the suggested ‘cell-type agnostic’ and ‘cell-type-specific’ terminology is that it could also lead to confusion, since the ***annotations*** produced by the concatenated model are not agnostic to the cell type.

The sentences where we introduce our terminology in the introduction now read:

“Previously, large collections of per-cell-type chromatin state annotations have been generated using either

(1) independent models that learn a different set of states in each cell or tissue type or (2) a single model that is learned across all cells and tissues, resulting in a common set of states across cell types, yet generating per-cell-type annotations (in some cases per-tissue annotations are generated, but we will use the terms cell- type and tissue interchangeably for ease of presentation). This latter approach is referred to as a ‘concatenated’ approach (**Supp. Fig. 1**) [1,2]”

We have made additional edits throughout the manuscript to reflect this updated terminology, and updated the Methods section as follows.

###### “Per-cell-type annotations used to compare against full-stack annotations

In evaluating how predictive the full-stack model is at annotating external genomic elements, we compared the full-stack model to two sets of per-cell-type chromatin state annotations in terms of their ability to predict external annotations. One set of per-cell type annotations was the 18-state ChromHMM from Roadmap Epigenomic Project [3] , which was based on a model trained using the concatenated approach and observed data of six chromatin marks (H3K4me1, H3K4me3, H3K9me3, H3K27ac, H3K27me3 and H3K36me3) in 98 cell types. In this model, we have a common set of state definitions across cell types, but unique state annotations for each cell type. The second set of per-cell type annotations were based on models learned independently in each of the 127 cell types. In learning these models, we partitioned the 1032 datasets used to learn the full-stack model into 127 subsets based on their associated cell type. For each of the 127 cell types, we applied ChromHMM to learn a 100-state per-cell-type ChromHMM model using only the observed data in the corresponding cell type. The number of states is similar to that in the full-stack model, to control for this variable in the evaluation. This process generates 127 models, each used to generate per-cell-type annotations in one cell type. The independent model learning approach of ChromHMM differs from the concatenated approach because the model parameters (state emission, transition, and initial probabilities) are different for different cell types, while state parameters in concatenated model are shared across cell-types. However, these two approaches both produce chromatin state annotations on a per-cell type basis. We learned these independent models with the same ChromHMM parameters as described above for the full-stack model, with the exception of using the ‘-init random’ flag to randomly initialize models’ parameters. Even when we specified the number of states to ChromHMM as 100, however, we note that due to the large number of states relative to the input tracks, for some of these models, fewer than 100 distinct states ended up being assigned to positions in the genome.”

3. *Benefits of stacked models*

*The authors argue that post-hoc clustering of concatenated state models (as done by Ernst et al. 2011 and Kundaje et al. 2015) does not provide a "systematic and global view of the data",*

*without specifying exactly what this really means.*

We thank the reviewer for pointing out that this was unclear. By “systematic and global view of the data” we mean an unbiased view on all the input data at once, allowing one to view how different data sets relate to each other. The post-hoc clustering is presenting a view on a subset of the data or dimension of it that was manually decided to focus on (as, for example, shown in Fig. 2 in [4], clustering of one promoter state and one enhancer state). We have revised the text to state:

“Previously, subsets of the genome assigned to individual chromatin states from ‘concatenated’ per-cell- type annotations were post-hoc clustered to analyze chromatin dynamics across cell and tissue types. However, such an approach does not provide a view of the dynamics of all the data at once, which the stacked modeling provides [3,4].”

*Throughout the manuscript the authors allude to the potential benefits of stacked models over models based on either concatenation of multiple cell types or individual cell types.*

*However, in my opinion, it remains unclear precisely what the benefits are and in which scenarios stacked models are preferred.*

*The manuscript contains several sections outlining enrichment levels of full-stack model states with a variety of other genomic and genetic annotations.*

*Although informative as a resource, these sections have not convinced me that non-stacked models are not / less well able to meaningfully pinpoint such annotations.*

*With stacked models, each genomic position is assigned a single chromatin state label, across all input biosamples.*

*However, they also add complexity (due to the increased number of states as compared to (concatenated) "cell-type-agnostic" models, and increased number of datasets as compared to "cell-type-specific" models).*

*It is not very clear how this increase in complexity is justified by a potential increase in Biological interpretation of states.*

We thank the reviewer for the feedback and apologize that the benefits of the stacked modeling were not clearer. Our purpose with the extensive set of quantitative comparisons was to demonstrate in an objective way that the stacked annotations can pin-point the genomic locations of a variety of external genomic annotations of interest better than non-stacked models. These external annotations include gene annotations, evolutionarily conserved bases, repeat classes, fine-mapped variants and structural variants, among others. We expected these results to stem primarily from the fact that the stacked model uses data from multiple cell and tissue types for annotation, and that in many cases, there is not a single cell or tissue type where all the genomic targets of interest will overlap with distinct epigenetic marks.

The stacked modeling also has the advantage of being expressive of combinations of signals of the same or different assays across cell types, hence allowing the model to directly differentiate constitutive from cell type dependent activity. As one specific example, the stacked model identified a state associated with H3K9me3 specifically in embryonic and iPSCs (Het9), and limited signal of any other mark in all cell types. As H3K9me3 is usually a marker of constitutive repressive activity, the presence of a H3K9me3- associated state in a per-cell-type annotation is insufficient to identify this cell-type-specific activity. Furthermore, the stacked model directly highlights how these regions lack signal for marks other than H3K9me3 across cell and tissue types. We have better highlighted this example in our revised version through **Supp. Fig. 26** (also shown below) and in the last paragraph of the Results section ‘*Stacked Model Differentiates Cell-Type-Specific from Constitutive Activity’.*

Additionally, in the stacked modeling, having a single annotation of the genome based on data from multiple cell types can also facilitate biological interpretation. For example, when analyzing the relationship between chromatin state and evolutionarily conserved bases, if one conducts the analysis in one cell type using its per-cell type annotation, many bases would lack an informative chromatin state annotation in that cell type (e.g. be assigned to the quiescent state). A subset of those bases would have informative chromatin state annotations in other cell types. To make statements across cell types using per-cell-type annotations would require a post-hoc method for reasoning about a potentially exponential number of chromatin state combinations across cell types. Such post-hoc approaches generally either oversimplify the data or can be more difficult to interpret. The stacked modeling avoids this by directly providing a single annotation of the genome based on all the data.

We have revised our Introduction and Discussion to make the points clearer. In the introduction we now state:

“Such a universal annotation from stacked modeling provides potential complementary benefits to existing per-cell-type chromatin state annotations. First, since the model can learn patterns from signals from the same assay across cell types, a stacked model may help differentiate regions with constitutive chromatin activities from those with cell-type-specific activities. Previously, subsets of the genome assigned to individual chromatin states from ‘concatenated’ per-cell-type annotations were post-hoc clustered to

analyze chromatin dynamics across cell and tissue types [3,4]. However, such an approach does not provide a view of the dynamics of all the data at once, which the stacked modeling provides. Second, the stacked modeling approach bypasses the need to pick a specific cell or tissue type when analyzing a single partitioning and annotation of the genome. Focusing on a single cell or tissue type may not be desirable for many analyses involving other data that are not inherently cell-type-specific, such as those involving conserved DNA sequence or genetic variants. For example, when studying the relationship between chromatin states and evolutionarily conserved sequences, if one uses per-cell-type chromatin state annotations from one cell type, many bases will lack an informative chromatin state assignment (e.g. many bases are in a quiescent state), while subsets of those bases will have a more informative annotation in other cell types. Third, if one tries to analyze per-cell-type annotations across cell types, one would need a post- hoc method to reason about an exponentially large number of possible combinations of chromatin states across cell types (if each of 𝐾 cell types has 𝑀 states, there are 𝑀^𝑘^ possible combinations of states for a genomic position) many of which would likely lack biologically meaningful distinctions. In contrast, for the stacked model, there will be a single annotation per position out of a possibly much smaller fixed number of states (compared to 𝑀^𝐾^). These states are directly informative of cross-cell type activity, though the state definitions can be more complex. Finally, annotations by the stacked modeling leverages a larger set of data for annotation, and thus has the potential to be able to identify genomic elements with greater sensitivity and specificity. ”

and in our discussion, which reads:

“The full-stack modeling has advantages to commonly used per-cell-type chromatin state annotations in several respects. First, the full-stack model learns patterns of signals of the same or different assays across cell types, hence can provide a unified view of all the data and directly uncover states that correspond to constitutive or cell-type-specific activities. For example, a state from the model, HET9, was associated with only the mark H3K9me3 specifically in ESCs and iPSCs even though this mark is typically associated with constitutive repression. Second, the full-stack annotation consistently showed better recovery of various genomic features compared to per-cell-type annotations. This improvement is expected since full-stack models can leverage information from multiple cell types for genome annotations. Third, in cases where it is not desirable to focus on only one specific cell or tissue for analysis, the full-stack modeling can bypass the need to pick one such cell or tissue type or to consider a large number of different per-cell-type chromatin state annotations simultaneously. Such cases may arise when studying other genomic information that is not inherently cell-type-specific such as genome variation and sequence conservation.”

We recognize that these benefits do come at the tradeoff of increased complexity of state definition. We previously discussed these tradeoffs in the introduction and discussion sections of our initial submission and have expanded this discussion section in the revised manuscript. We emphasize that we believe that the stacked modeling approach is complementary to non-stacked approaches, and which modeling approach is more appropriate will depend on the specific application or analysis. In particular, we expect per-cell- type annotations based on concatenated or independent models to be preferred if one is interested in a particular cell/tissue type such as when analyzing other data collected in the same cell or tissue type. Additionally, per-cell type annotations can also be preferred even if one is considering multiple cell or tissue types, but the primary interest is in making statements about activity in individual cell or tissue types. We expect the stacked modeling to often be preferred when one is interested in the joint analysis of multiple cell or tissue types. This could be, for example, when one is interested in distinguishing constitutive activity to activity specific to certain groups of cell or tissue type.

Our updated paragraph of the discussion on trade-offs now reads:

“Despite these advantages, there are trade-offs in using the stacked modeling approach, and we emphasize that the stacked modeling approach should be considered a complement to and not a replacement of the per-cell-type annotations. Compared to typical concatenated models, the full-stack model has increased model complexity because of the increased number of parameters from the larger number of states and input features, which can make interpreting some model states relatively more difficult. Additionally, if one is interested in a specific cell type, then corresponding per-cell-type annotations can have advantages in that all the annotations are directly informative about the chromatin state in the cell type of interest. An additional trade-off is that with the stacked model, it is not possible to incorporate additional data without relearning a model, while for a concatenated-model one can annotate a new cell based on an existing model, provided that the set of marks in the new cell type are the same as the existing model. We also note that post-hoc per-cell-type state annotations can also be used for cross-cell type analyses, particularly on a per- state basis by analyzing the frequency of a specific state across cell types. While per-state analyses using per-cell-type concatenated annotations can be relatively straightforward and informative, they give only partial and potentially oversimplified views of all the data, ignoring distinctions among different per-cell- type states. Whether to use per-cell-type annotations or full-stack annotations will depend on the specific application. Per-cell-type annotations may be preferable when one is interested in studying a specific cell type, while full-stack annotations can be preferred in joint analyses of multiple cell types.”

To concurrently address reviewer 2’s comments about the quantitative comparisons with the per-cell type annotations, we have removed the section ‘Results/ Full-stack states are more predictive of external annotations than cell-type-specific models’ to put less emphasis on the comparison. Instead, we added a revised paragraph towards the end of section ‘Results/Major groups of full-stack states’, which is presented after the next comment.

We have also added two examples of loci where the full-stack state annotations capture dynamic cross- cell type activity, assigned to states HET9 and PromF5, as in Supplementary Figures 26-27, respectively. These figures are shown below and referenced from the main text with the statement:

“Additionally, state HET9 showed strong association as heterochromatin in ESC/iPSC groups, while being mostly quiescent in other cell types based on per-cell-type annotations (**Fig. 2C, Supp. Fig. 26, Supplementary Data File 1,3**). State PromF5 is associated with putative bivalent promoter chromatin states in some blood and ESC-related cell types, but with flanking promoter states in most other cell types (**Supp. Fig. 27, Supplementary Data 1,3**).”

#### Full- stack states

**blood**

**Brain**

**Digestive**

**Epithelial**

**ESC**

**Heart**

**iPSC**

**M esench**

**M uscle**

**M yosat**

**Ne urosph**

**Skin**

**Thymus**

**Demonstration of the state transitions for full-stack state HET9**

**Full-stack state colors**

**Assembly Gaps &**

**artifacts**

**quiescent**

**HET**

**polycomb**

**repressed**

**acetylations**

**weak**

**enhancers**

**enhancers**

**transcribed &**

**enhancer**

**weak**

**transcription**

**transcription**

**exon &**

**transcription**

**ZNF genes**

**DNase**

**bivalent**

**promoter**

**flanking**

**promoter**

**TSS**

**Per-cell-type state colors**

### **Supplementary Figure 26: Illustration of full-stack heterochromatin state HET9.** The figure captures the per-cell-type-concatenated chromatin state maps for various reference epigenomes, and the corresponding full-stack chromatin state maps at region chr17:21772099- 21791900. The first 66 tracks show chromatin state annotations from a 25-state concatenated model for 66 reference epigenomes (equivalently, in this paper, cell types) (Ernst & Kellis, 2015). The individual reference epigenomes IDs and their tissue groups are labeled on the left. The chromatin state colors are explained on the right. The last track, shown in full mode to display all state labels on the right, corresponds to the full-stack chromatin state map at this region. State HET9 is characterized, based on our analysis, as an ESC-group-related heterochromatin state (**Fig.**

**TssA**

**Prom U**

**Prom D 1**

**Prom D 2**

**Tx5p**

**Tx**

**Tx3p**

**TxWk**

**Tx Reg**

**Tx Enh5p**

**Tx Enh3p**

**Tx EnhW**

**EnhA1**

**EnhA2**

**EnhAF**

**EnhW 1**

**EnhW 2**

**EnhAc**

**DNase**

**ZNF/Rpts**

**Het**

**Prom P**

**Prom Biv**

**ReprPC**

**Quies**

**2C, Supp. Fig. 8-9, Supplementary Data 1,3**). Detailed characterizations of all full-stack states are in Supplementary Data.

#### Demonstration of the state transitions for full-stack state PromF5

**Full-stack state colors**

**blood**

**Brain**

**Digestive**

**Epithelial**

**ESC**

**Heart**

**iPSC**

**Me sench**

**M uscle**

**M yosat**

**Neurosph**

**Skin**

**Thymus**

**Assembly Gaps &**

**artifacts**

**quiescent**

**HET**

**polycomb**

**repressed**

**acetylations**

**weak**

**enhancers**

**enhancers**

**transcribed &**

**enhancer**

**weak**

**transcription**

**transcription**

**exon &**

**transcription**

**ZNF genes**

**DNase**

**bivalent**

**promoter**

**flanking**

**promoter**

**TSS**

**Per-cell-type state colors**

**TssA**

**Prom U**

**Prom D 1**

**Prom D 2**

**Tx 5p**

**Tx**

**Tx 3p**

**Tx Wk**

**TxReg**

**Tx Enh5p**

**Tx Enh3p**

**Tx EnhW**

**EnhA1**

**EnhA2**

**EnhAF**

**EnhW 1**

**EnhW 2**

**EnhAc**

**DNase**

**ZNF/Rpts**

**Het**

**Prom P**

**Prom Biv**

**ReprPC**

**Quies**

**Full-stack**

### **Supplementary Figure 27: Illustration of full stack flanking promoter state PromF5.** The figure captures the per-cell-type-concatenated chromatin state maps for various reference epigenomes, and the corresponding full-stack chromatin state maps at region chr10:33621649-33627350. The first 66 tracks show chromatin state annotations from a 25-state concatenated model for 66 reference epigenomes (equivalently, in this paper, cell types) (Ernst & Kellis, 2015). The individual reference epigenomes IDs and their tissue groups are labeled on the left. The chromatin

state coloring is labeled on the right. The last track, shown in full mode to display all state labels on the left, corresponds to the full-stack chromatin state map at this region. State PromF5 is characterized, based on our various analyses as state frequently found at flanking promoter regions with some upstream bias, and sometimes, this state overlaps with regions of bivalent promoters in Blood-related and ESC-related groups (Blood & T cells, HSC & B cells, ESC, iPSC and ES-deriv) (**Supp. Fig. 8-9, Supplementary Data 1,3**). Detailed characterization of all full-stack states are in **Supplementary Data 1-3**.

*Regarding section "Full-stack states are more predictive of external annotations than cell-type-specific models":*

*ChromImpute and other related approaches have taught us that there is a lot of value in considering shared information across chromatin marks and cell types.*

*As such, it does not come as a big surprise that the full-stack model is better able to predict the occurrence of certain external annotations.*

*It does suggest that stacked models offer an added value similar to that of data imputation, in the sense that shared patterns are re-inforced and random noise is suppressed.*

*I wonder whether this could be the reason why for instance LMNB1 domains are better predicted using the full stack model than the 18-state concatenated model,*

*yet there are individual cell-type-specific models that perform better than the full-stack model (i.e. Fig 4b).*

*An important detail here is that these LMNB1 domains were profiled in Tig3 human lung fibroblasts, which are not (exactly) present in the set of 98 or 127 Roadmap samples.*

*What samples from these cell-type-specific models outperform the full-stack model? Fibroblast-related ones?*

We thank the reviewer for the comments and questions. As the reviewer suggests, one reason that the full- stack model can outperform per-cell-type models is that the additional data can remove noise and reinforce shared signal. An additional, and likely the primary reason is that the stacked model has coverage of more cell types, so it can better capture regions of interest that are not constitutively active and for which no per- cell-type annotation can capture all. Per-cell-type state annotations would be expected to have relatively better performance when the regions of interest are specific to that cell type or a closely related one, as the modeling power is entirely devoted to annotations specific to that cell type. Consistent with this, a number of the samples that outperformed the stacked model for LMNB1 in Tig3 human lung fibroblasts are other fibroblast samples. Specifically, the outperforming samples are H1 Derived Mesenchymal Stem Cells (E006), IMR90 fetal lung fibroblasts Cell Line (E017), Mesenchymal Stem Cell Derived Chondrocyte Cultured Cells (E049), Foreskin Fibroblast Primary Cells skin01 (E055), Foreskin Fibroblast Primary Cells skin01 (E056), and Osteoblast Primary Cells (E129). We now provide **Supplementary Data File 4** to summarize results of all comparison between full-stack annotation and per-cell type annotations.

In the revised manuscript we now address this point in the last paragraph of the section ‘Major groups of full-stack states’:

“We also confirmed that the full-stack annotations were generally more predictive of the positions of a variety of external genome annotations considered in **Fig. 3A** than two sets of per-cell-type annotations, a previous 18-state per-cell-type chromatin state annotation based on concatenated models from 127 cell types [3], and 100-state per-cell-type annotations learned independently in each cell type (**Methods**). As

expected, since the full-stack model uses more data representing more cell types, the full-stack annotations had greater predictive performance in most cases (**Supp. Fig. 19-22, Supplementary Data 4)**. One of the exceptions to this was lambin B1 associated domains from Tig3 human lung fibroblasts [5], where six per- cell type annotations were more predictive, three of which are annotations for fibroblasts. We note that these evaluations were done under the assumption that a chromatin state annotation that is more predictive of well-established external genomic elements will also be more informative of less well-established classes of elements. These results suggest that the full-stack annotations will, in most cases, have greater information than any single per-cell-type annotations about localization patterns of some target genomic elements, with likely exceptions when the target of interest is specific to a certain cell type. In such cases, the corresponding per-cell type chromatin state annotation may show better recovery.”

4. *Stacked vs. concatenated models for characterizing dynamic state behavior*

*This pertains to section "Stacked Model Differentiates Cell-Type-Specific from Constitutive Activity": The authors convincingly show that different full-stack states show up selectively for certain (groups of) cell types.*

*However, it is not clear to me exactly how a stacked model approach yields more meaningful information about activity specificity than a post-hoc analysis of concatenated model results.*

*In a way, a concatenated approach offers more flexibility to select for different levels of specificity (e.g. "x out of n samples are in state i"), rather than being confined to a fixed number of full-stack model states.*

We thank the reviewer for pointing out that this was not clear. While the analysis approach suggested by the reviewer may be a reasonable choice for some specific analyses, we feel it can be too oversimplified for others.

Knowing the groups of cell types that have shared activity can be valuable information to preserve in some cases, which would be lost when simply counting the number of cell types assigned to a given state. As the reviewer acknowledges, the stacked modeling is able to effectively capture this information. Additionally, simply counting the number of cell types or biosamples that are annotated as a state runs a greater risk of being confounded by imbalances in the representation (number of available biosamples) of different classes of cell and tissue types.

The approach suggested by the reviewer would also ignore potentially important distinctions between the states being assigned at a position in multiple cell types/biosamples. For example, it is common for a chromatin state model to have multiple different enhancer associated states. In such cases, if one is analyzing one of the enhancer states, a location that is assigned to that enhancer state in half the cell types and a quiescent state in the other half would be treated the same as a location assigned to that enhancer state in half the cell types and other enhancer states in the remaining cell types.

Additionally, conducting analyses on a per-state basis will only directly give incomplete views of the data, similarly to conducting analyses on a per-cell type basis. For example, if one is analyzing the relationship between chromatin states and evolutionarily conserved bases, some conserved bases might not appear in a specific chromatin state of interest in any cell type, but in another state of interest. One would then need a post-hoc way to reason about the frequency across cell types of each state at a position.

In our discussion we have added the following text to summarize these points:

“We also note that post-hoc per-cell-type state annotations can also be used for cross-cell type analyses, particularly on a per-state basis by analyzing the frequency of a specific state across cell types. While per- state analyses using per-cell-type concatenated annotations can be relatively straightforward and informative, they give only partial and potentially oversimplified views of all the data, ignoring distinctions among different per-cell-type states.”

We have also created additional supplementary figures (now in **Supp. Fig. 8-9, 26-27**) better highlighting the different patterns of cross-cell type activity captured by the chromatin states (see response to previous point and response to reviewer 2).

5. *Choice of number of chromatin states*

*This is a commonly recurring question in genome segmentation and clustering approaches, and I realize there is no definitive answer.*

*That said, I found the justification for the use of 100 states not particularly strong.*

*The authors tried out several numbers of states, and conclude that 100 is "best", although their reasoning would just as well support e.g. 80 states.*

*What does the number of states mean in terms of downstream results? Does it even matter in practice? What guidance can they give end users regarding this matter? It would be good to discuss this more.*

*Also, since each chromatin mark is expected to play a role (or at least, occur) roughly equally often in each cell type (with some exceptions, e.g. PSCs),*

*I wonder whether the distribution of chromatin marks over full-stack states can be used as a guideline to choose a more optimal number of states.*

*For instance, one would expect that each general "group" of cell types (e.g. "Brain") has some set of group-specific enhancers, which would ideally be captured by their own state (at least one).*

*If enhancer states only capture a few such cell type groups, perhaps the number of states is too low, whereas if they capture similar patterns "spread out" across groups, the number of states might be too high.*

*I imagine this is also a function of the input datasets, e.g. if 90% of input datasets are "Brain", that group will likely be favored over others?*

We thank the reviewer for these questions and suggestions.

In the revised manuscript, we now learned models with 10 to 120 states in increments of 5 states. We applied the ‘CompareModels’ command of ChromHMM using the 100-state model as the reference model. This reports, for each other model (whose number of states is not 100), the maximum correlation in terms of the emission parameters of any state in the model with a state of the reference (100-state) model. The CompareModels analysis results (shown in **Supp. Fig.3C** and below) revealed states in the 100-state model that were less well-represented in models with 55 to 95 states based on emission parameter correlation. For example, EnhA20, corresponding to HUVEC enhancers, had a maximum correlation in the range 0.31 to

0.71 for models with 95 or fewer states, but 1.00 for those with greater than 100 states. There is a phase transition related to the ‘Brain’ group for models with at least 55 states, where the correlation with EnhA6, the primary brain specific enhancer state in 100-state full-stack model, goes from <=0.87 for models with

<= 50 states to >=0.98 for models with >=55 states.

We did another analysis where for each of these models, we obtained emission probabilities of marks associated with H3K4me1, an enhancer mark available in all the cell and tissue types. For each state in each model, there are 127 input features from different cell types associated with H3K4me1. These inputs features can be grouped by their associated tissue group, for example, feature ‘E003-H3K4me1’ is associated with tissue group ESC based on metadata from [Roadmap Epigenomics Consortium](https://docs.google.com/spreadsheets/d/1yikGx4MsO9Ei36b64yOy9Vb6oPC5IBGlFbYEt-N6gOM/edit#gid%3D15). Then, for each of the 19-tissue groups from Roadmap, we computed the maximum correlations (among all states in each model) between emission parameters of H3K4me1 and a vector of size 127 that is ‘1’ for samples in the group and ‘0’ outside the group. Results of this analysis are shown in **Supp. Fig. 3D** and below. Similar to the CompareModels analysis mentioned above, the correlations based on H3K4me1 in the Brain group also saw a similar transition with 55 states from a correlation <=0.63 with fewer than 55 states to correlations 0.78-0.81 with 55 or more states.

The allocation of states representing a set of enhancers would be expected to depend, to some extent, on how well-represented that data is in the input, but also how distinct those inputs are. Even with 100-states, we were able to identify some states that were highly specific to a small number of inputs such as the Huvec-specific enhancer state EnhA20.

We also conducted an additional analysis analyzing the negative log-likelihood, AIC, and BIC as a function of the different number of states evaluated at 300 genomic regions of length 1-Mbp. All of these measures decrease with the number of states, but by diminishing amounts with the increase of the number of states (now shown in **Supp. Fig. 3B** and below). This is consistent with our previous AUROC analysis of predictions of external annotations for which we saw diminishing returns as we continued to increase the number of states.

These analyses support that biologically meaningful distinctions continue to be made at 100 states. They also suggest that while additional biological distinctions may be expected with more states, the distinctions would likely be fewer and more subtle. As our focus is to provide a characterization of each state as a resource for future applications, we felt limiting the model size to 100 states keeps this at a manageable number to interpret and use in other applications. We also note that we grouped these 100 states into 16 groups, and these groupings can be useful for some purposes such as viewing in a genome browser.

As the reviewer notes, the question of choosing the number of states in a model is a general question. The best number can depend on the desired biological resolution. It is also easier to establish lower bounds than upper bounds, since with lower bounds one can point to clear biological distinctions of interest that cannot be resolved with a sufficient number of states. The [2] paper has discussion of this and the CompareModels command in ChromHMM.

##### A B

**AIC and BIC of models with different numbers of states**

num_state

values

D	**Max correlations of emission parameters associated with H3K4me1 with indicators of cell groups**

Number of states

Tissue groups

|  | 10 | 15 | 20 | 25 | 30 | 35 | 40 | 45 | 50 | 55 | 60 | 65 | 70 | 75 | 80 | 85 | 90 | 95 | 100 | 105 | 110 | 115 | 120 |
| --- | --- | --- | --- | --- | --- | --- | --- | --- | --- | --- | --- | --- | --- | --- | --- | --- | --- | --- | --- | --- | --- | --- | --- |
| Adipose | 0.17 | 0.18 | 0.18 | 0.18 | 0.18 | 0.17 | 0.17 | 0.17 | 0.17 | 0.18 | 0.19 | 0.18 | 0.18 | 0.18 | 0.19 | 0.18 | 0.18 | 0.18 | 0.19 | 0.21 | 0.19 | 0.19 | 0.19 |
| Blood & T-cell | 0.67 | 0.67 | 0.68 | 0.70 | 0.69 | 0.71 | 0.71 | 0.76 | 0.84 | 0.84 | 0.86 | 0.86 | 0.86 | 0.86 | 0.86 | 0.86 | 0.85 | 0.86 | 0.86 | 0.84 | 0.85 | 0.85 | 0.86 |
| Brain | 0.01 | 0.06 | 0.11 | 0.10 | 0.56 | 0.58 | 0.57 | 0.61 | 0.63 | 0.80 | 0.80 | 0.80 | 0.80 | 0.80 | 0.80 | 0.77 | 0.78 | 0.81 | 0.81 | 0.79 | 0.79 | 0.80 | 0.80 |
| Digestive | -0.05 | -0.02 | 0.07 | 0.06 | 0.23 | 0.33 | 0.45 | 0.55 | 0.54 | 0.50 | 0.61 | 0.54 | 0.55 | 0.63 | 0.64 | 0.65 | 0.64 | 0.65 | 0.64 | 0.64 | 0.64 | 0.65 | 0.64 |
| ENCODE2012 | 0.39 | 0.44 | 0.43 | 0.45 | 0.45 | 0.45 | 0.46 | 0.44 | 0.45 | 0.44 | 0.47 | 0.49 | 0.48 | 0.48 | 0.48 | 0.50 | 0.52 | 0.50 | 0.52 | 0.49 | 0.49 | 0.52 | 0.53 |
| Epithelial | 0.34 | 0.34 | 0.35 | 0.36 | 0.38 | 0.38 | 0.38 | 0.45 | 0.45 | 0.45 | 0.46 | 0.45 | 0.45 | 0.48 | 0.47 | 0.48 | 0.48 | 0.47 | 0.48 | 0.48 | 0.49 | 0.49 | 0.49 |
| ESC | 0.38 | 0.40 | 0.62 | 0.62 | 0.67 | 0.67 | 0.65 | 0.67 | 0.68 | 0.69 | 0.68 | 0.69 | 0.68 | 0.68 | 0.68 | 0.68 | 0.70 | 0.70 | 0.72 | 0.70 | 0.71 | 0.72 | 0.71 |
| ES-deriv | 0.17 | 0.32 | 0.34 | 0.29 | 0.29 | 0.34 | 0.41 | 0.45 | 0.42 | 0.38 | 0.42 | 0.46 | 0.41 | 0.43 | 0.42 | 0.40 | 0.43 | 0.43 | 0.45 | 0.43 | 0.43 | 0.48 | 0.45 |
| Heart | 0.13 | 0.12 | 0.22 | 0.20 | 0.20 | 0.24 | 0.24 | 0.25 | 0.27 | 0.28 | 0.32 | 0.35 | 0.36 | 0.34 | 0.35 | 0.37 | 0.33 | 0.40 | 0.39 | 0.29 | 0.41 | 0.39 | 0.42 |
| HSC & B-cell | 0.52 | 0.57 | 0.55 | 0.57 | 0.58 | 0.61 | 0.60 | 0.74 | 0.78 | 0.75 | 0.80 | 0.80 | 0.80 | 0.79 | 0.80 | 0.79 | 0.71 | 0.80 | 0.65 | 0.66 | 0.81 | 0.79 | 0.76 |
| IMR90 | 0.19 | 0.19 | 0.20 | 0.47 | 0.48 | 0.46 | 0.50 | 0.52 | 0.51 | 0.55 | 0.52 | 0.57 | 0.57 | 0.58 | 0.56 | 0.56 | 0.60 | 0.56 | 0.63 | 0.56 | 0.67 | 0.60 | 0.62 |
| iPSC | 0.37 | 0.32 | 0.44 | 0.45 | 0.48 | 0.48 | 0.48 | 0.48 | 0.49 | 0.48 | 0.48 | 0.48 | 0.48 | 0.48 | 0.48 | 0.50 | 0.51 | 0.52 | 0.51 | 0.51 | 0.51 | 0.51 | 0.52 |
| Mesench | 0.45 | 0.48 | 0.49 | 0.48 | 0.49 | 0.58 | 0.53 | 0.58 | 0.68 | 0.62 | 0.69 | 0.68 | 0.71 | 0.74 | 0.72 | 0.65 | 0.69 | 0.62 | 0.76 | 0.75 | 0.73 | 0.77 | 0.68 |
| Muscle | 0.28 | 0.28 | 0.41 | 0.37 | 0.35 | 0.40 | 0.43 | 0.41 | 0.40 | 0.53 | 0.50 | 0.55 | 0.53 | 0.55 | 0.50 | 0.54 | 0.52 | 0.63 | 0.53 | 0.54 | 0.53 | 0.63 | 0.60 |
| Myosat | 0.24 | 0.28 | 0.28 | 0.27 | 0.27 | 0.33 | 0.32 | 0.33 | 0.32 | 0.35 | 0.33 | 0.34 | 0.34 | 0.33 | 0.36 | 0.37 | 0.36 | 0.38 | 0.36 | 0.34 | 0.33 | 0.36 | 0.36 |
| Neurosph | 0.12 | 0.16 | 0.15 | 0.17 | 0.25 | 0.28 | 0.28 | 0.28 | 0.29 | 0.27 | 0.26 | 0.27 | 0.36 | 0.32 | 0.31 | 0.32 | 0.29 | 0.47 | 0.40 | 0.37 | 0.34 | 0.38 | 0.46 |
| Other | 0.15 | 0.17 | 0.17 | 0.18 | 0.19 | 0.23 | 0.23 | 0.23 | 0.24 | 0.29 | 0.28 | 0.29 | 0.29 | 0.37 | 0.35 | 0.36 | 0.31 | 0.29 | 0.32 | 0.42 | 0.45 | 0.32 | 0.38 |
| Sm. Muscle | 0.05 | 0.07 | 0.06 | 0.07 | 0.11 | 0.10 | 0.11 | 0.13 | 0.11 | 0.21 | 0.21 | 0.20 | 0.20 | 0.20 | 0.21 | 0.22 | 0.22 | 0.23 | 0.23 | 0.22 | 0.23 | 0.24 | 0.23 |
| Thymus | 0.06 | 0.13 | 0.13 | 0.13 | 0.13 | 0.16 | 0.16 | 0.17 | 0.16 | 0.17 | 0.17 | 0.17 | 0.17 | 0.17 | 0.16 | 0.18 | 0.19 | 0.18 | 0.20 | 0.21 | 0.21 | 0.22 | 0.22 |

C	**Maximum correlations of emission parameters of full-stack states with any state in other models**

Number of states

0.98	0.97	0.78

0.96	0.91	0.93	0.99	0.99

0.93	0.93	0.37	0.93	0.67	0.94	0.92

0.86	1.00	1.00	1.00	1.00

1.00	1.00	1.00	1.00	1.00

0.98	0.99	0.99	0.99	0.99	1.00	0.99	0.99	0.99	1.00	1.00

0.92	0.92	0.91	0.90	0.90	0.92	1.00

0.87	1.00	0.99	0.99	0.99	0.99	0.99

0.98	0.87	0.94	0.96	0.96 0.99	1.00

0.95	0.98	0.98

0.94	0.93	0.98	0.96	0.90

0.95	0.88	0.91

0.40	0.86	0.91	0.94	0.99	0.98	0.99	0.99	0.99	0.94	0.99	0.99	0.99	0.98	0.98	0.97	1.00

0.90	0.92	0.94	0.96	0.98	0.97	0.95	0.97	0.97	0.98	0.99	1.00	0.99	0.99	1.00	1.00	0.99	1.00

0.82	0.79	0.87	0.81	0.82	0.80	0.82	0.81	0.81	0.85	0.87	0.85	0.86	0.86	0.84	0.99	0.99	0.98

0.84	0.86	0.91	0.91	0.89	0.89	0.89	0.87	0.88	0.87	0.94	0.92	0.91	0.95

0.85	0.93	0.91	0.95	0.94	0.94	0.97	0.94	0.96	0.94	0.94	0.97	0.97	0.97	0.93

0.85	0.84	0.86	0.93	0.97	0.96	0.97	0.96	0.99	0.98	0.98	0.98	0.98	1.00

0.95	0.97	0.99	0.95	0.99	0.99	1.00	0.99	0.98	1.00	1.00

0.33	0.30	0.36	0.49	0.86	0.88	0.94	0.88	0.87	0.87	0.89

0.91	0.92	0.92	0.89	0.91	0.90	0.91	0.91	0.91	0.99	0.91

0.94	0.96	0.98	0.94	0.98	0.97	0.97	0.97	0.96	0.97	0.97

1.00	0.99	0.99

0.98	0.95	0.97

0.99	0.99	0.99	0.99	0.99	0.96	0.94	0.94	0.94	0.99	0.98	0.97	0.99	0.99	1.00

0.69	0.70	0.70	0.69	0.79	0.78	0.78	0.79	0.79	0.79	0.78	0.78	0.74	0.78	0.94	0.77	0.78

0.77	0.92	0.86	0.80	0.80

0.97	0.97	0.97	0.97	0.98	0.98	0.98	0.98	1.00	1.00	1.00	1.00	0.99	1.00	0.98	1.00	1.00	1.00

0.73	0.74

0.94	0.94	0.96	0.99	0.99	0.99	1.00	0.99	1.00	1.00	1.00	1.00	1.00	1.00	1.00	1.00

0.52	0.54	0.56	0.98	0.99	1.00	1.00	1.00	1.00	1.00	1.00	1.00	1.00	1.00	1.00

0.76	0.81	0.91	0.87	0.98	0.99	0.95	0.97	0.97	0.95

0.92	0.96	0.95	0.96	0.96	0.96	0.96	0.98	0.98	0.98	0.99	0.98	0.98	0.98	0.98	1.00	1.00	1.00

0.96	0.92	0.96	0.95	0.96	0.95	0.96	0.95	0.96	0.96	0.96	0.99	1.00

1.00	1.00	1.00	1.00

1.00	1.00	0.99	1.00

1.00	0.98	1.00	1.00

1.00

0.95	0.99	0.99

0.83	0.80	0.80	0.82	0.81

**TSS2**

**TSS1**

0.64

**Prom F7**

1.00

1.00

1.00

1.00

0.96	0.96	0.97	1.00	1.00	1.00	1.00

0.95	0.96	0.95	1.00	0.99	1.00	1.00

**Prom F6**

1.00	1.00	1.00	1.00

1.00

0.98	0.97	1.00	1.00	1.00	1.00	1.00

**Prom F5**

1.00	1.00	1.00	1.00	1.00	1.00	1.00

0.91	0.98	0.96	0.98	0.99	1.00	1.00	1.00	1.00	1.00	1.00

0.95	0.91	0.89	0.93	0.94	0.91	0.97	0.93	0.91	0.92	0.92

0.85	0.88	0.81	0.97	0.97 0.92	0.95	0.95	0.93	0.95	0.97

**Prom F4**

**Prom F3**

1.00	0.98	0.99	0.95

1.00	1.00	1.00	0.99

1.00	1.00	1.00	1.00

1.00

1.00

1.00

0.85	0.88	0.88	0.87	0.89	0.89	0.90	0.88	0.89	0.96	0.97	0.96	0.99	0.99	0.99	1.00	1.00	1.00

0.98	0.97	0.98	0.96	0.95	0.98	0.97	0.99	0.99	0.99	0.99	1.00	1.00	0.99	1.00	1.00	1.00	1.00

**Prom F2**

0.92

0.86	0.86	0.86	0.83	0.85	0.84

0.72

0.77

0.81

**Prom F1**

0.87	0.79	0.92	0.92	0.94	0.72	0.73	0.89

0.83	0.83	0.82	0.85	0.90	0.92	0.91	0.96

0.73

0.65	0.72	0.89

0.77	0.75	0.73	0.73	0.72

0.62

**BivProm4**

0.96

**BivProm3**

0.86

**BivProm2**

0.79	0.97	0.94	0.94	0.99	0.95	0.99	0.99	0.99	0.98	0.93	0.91	1.00	1.00	0.99	0.99	1.00

0.93	0.87	0.94	0.96	0.95 0.93	0.93	0.98	0.95	0.99	0.95	0.97	0.99	0.99	0.97	0.97	1.00

0.92	0.91	0.90	0.90	0.90	0.86	0.90	0.90	0.91	0.96	0.96	0.90	0.92	0.93	0.89	0.87	0.87

0.64

**BivProm1**

1.00	0.99	1.00	1.00

1.00	1.00	1.00	1.00

1.00	1.00	0.99	1.00

0.97	0.96	0.92	0.97

0.97	0.96	0.96	0.94

1.00	0.87	0.97	0.88

1.00

1.00

1.00

1.00

1.00

1.00

0.34	0.34	0.34

**DNase1**

1.00	1.00	0.99	0.99

1.00

0.72

**znf2**

1.00	1.00	0.98	0.98

1.00

1.00	1.00	1.00	1.00	1.00	1.00

0.86	0.86	0.86	0.91	0.90	0.89	1.00	0.88	0.87	0.85

0.75

0.71

0.63

**znf1**

0.94

0.87

0.98	0.99

1.00

0.99	0.97	0.99

0.93	0.96	0.94

0.90	0.89

0.82	0.93	0.83	0.80	0.82	0.80	0.80	0.84

TxEx4

1.00	0.99	1.00	1.00

1.00

TxEx3

TxEx2

TxEx1

0.96	0.95	0.97	0.98

0.96	0.98	1.00	0.98

1.00	1.00	1.00	1.00

1.00

1.00

1.00

0.90	0.90	0.89	0.89	0.90 0.90	0.91	0.91	0.91	0.96	0.93	0.95	0.96	0.96	0.93	0.99	0.94	0.99

0.95	0.95	0.95	0.95	0.94	0.94	0.94	0.93	0.94	0.94	0.94	0.94	0.98	0.95	0.96	0.96	0.94	0.97

0.99	0.99	0.99	0.99	1.00	1.00	1.00	1.00	1.00	1.00	1.00	1.00	1.00	1.00	1.00	1.00	1.00	1.00

**Tx8**

**Tx7**

**Tx6**

0.99	0.99	0.99	0.99

1.00	1.00	1.00	0.99

1.00	1.00	1.00	1.00

1.00

1.00

1.00

0.94	0.94	0.94	0.91	0.93	0.93	0.93	0.92	0.97	0.96	0.97	0.97	0.99	0.97	1.00	1.00	1.00	1.00

0.92	0.92	0.91	0.95	0.95	0.95	0.94	0.94	0.96	0.97	0.97	0.99	0.99	0.99	1.00	1.00	0.99	1.00

0.95	0.95	0.97	0.99	0.99	0.99	0.99	0.99	1.00	1.00	1.00	1.00	1.00	0.99	1.00	1.00	0.99	1.00

**Tx5**

0.99	0.99	0.99	0.99

1.00

0.99	0.97	0.99	0.99	0.99	0.99	0.99	0.99	0.99	0.99

0.89	0.85

**Tx4**

0.77

0.79

**Tx3**

0.84

**Tx2**

0.97	0.98	0.99	0.99	0.99	0.99	0.98	0.99	0.98	0.97	0.98	1.00	0.99	0.99	1.00	1.00	1.00

0.95	0.94	0.96	0.98	0.98	0.98	0.99	0.98	0.99	0.99	0.99	1.00	1.00	0.97	1.00	1.00	1.00

0.86	0.83	0.83	0.86	0.86	0.85	0.86	0.87	0.86	0.97	0.98	0.97	0.96	0.96	1.00	0.99	0.99

0.90

**Tx1**

0.88	0.88	0.88	0.87	0.88	0.95	0.98	0.98	0.97	0.99	0.91	0.99	1.00	0.99	1.00

0.71	0.71	0.71

**TxWk2**

0.96	0.97	0.93	0.96	0.99	0.99	0.97	0.98	0.97	1.00	0.97	0.99	1.00	0.92	0.99	1.00	0.99	1.00

**TxWk1**

0.71	0.84	0.84	0.84	0.79	0.84	0.91	0.93	0.92	0.97	0.92	0.93	1.00	0.93	0.99

0.66	0.67	0.67

**TxEnh8**

0.97	0.92	0.96	0.93

0.99	0.93	0.97	0.97

0.99	0.99	1.00	0.94

0.99	0.98	1.00	0.96

1.00	1.00	0.99	1.00

1.00	1.00	1.00	1.00

0.98	0.99	0.99	0.99

1.00

1.00

1.00

1.00

1.00

1.00

1.00

0.84	0.89	0.89	0.87	0.83	0.82	0.83	0.99	0.97	0.92	0.94	0.91	0.89	0.88	0.88	0.98	0.94	0.97

**TxEnh7**

1.00	1.00	1.00

0.79

1.00

1.00

**TxEnh6**

0.86	0.86	0.86

**TxEnh5**

0.98	0.97	0.99	0.98

1.00	0.99	1.00	0.98

1.00

1.00

0.96	0.97	0.97	0.97	0.96	0.96	0.96	0.97	0.96	0.97	0.99	0.98	0.97	0.99	0.98	0.98	0.98	0.98

**TxEnh4**

0.88	0.86	0.87

0.82

**TxEnh3**

0.98	0.99	0.98	0.99

0.97	0.89	0.89	0.99

0.87

0.95	0.88	0.90

**TxEnh2**

0.84

0.79	0.79	0.78

0.70

**TxEnh1**

1.00	1.00	1.00	1.00

0.37	0.39	0.38	0.40	0.40	0.41	0.45	0.41	0.44	0.48	0.46	0.40	0.71	0.53

0.82	0.83	0.81	0.78	0.82	0.81	0.84	0.82	0.85	0.85	0.88	0.96	0.90	0.99

0.95	0.95	0.95	0.93	0.99	0.96	0.95	0.98	0.97	0.95	0.99	0.96	0.94	1.00

0.86	0.86	0.86	0.90	0.85	0.91	0.87	0.89	0.89	0.90	0.90	0.87	0.90	0.89

0.31	0.34	0.35	0.36

**EnhA20**

0.92

1.00

1.00

1.00

1.00

1.00

0.96	0.95	0.90	1.00	0.97	0.93	0.91	0.92	0.94	0.93	0.96

0.80	0.96	0.98

0.46	0.80	0.86	0.79

**EnhA19**

0.97	0.98	1.00	0.98

1.00

0.54

**EnhA18**

0.95

1.00	1.00	1.00

0.64

**EnhA17**

0.86	0.86	0.85	0.86

1.00

1.00

0.25

0.43

0.39

**EnhA16**

0.96	0.96	0.95	0.94

1.00

0.46	0.40	0.47	0.73	0.82

0.43	0.44	0.45	0.42	0.44

0.93	0.93	0.90	0.92	0.91

0.88	0.88	0.94	0.94	0.90

0.38

0.39

0.30

**EnhA15**

0.92

0.81

0.58	0.58

**EnhA14**

0.62

0.68

0.58	0.62

0.43	0.42	0.46

0.44	0.44	0.51

**EnhA13**

0.65

**EnhA12**

0.83	0.80	0.84	0.85	0.85 0.86	0.90	0.88	0.91	1.00	0.91

0.92	0.92	0.90	0.90	0.89	0.89	1.00	1.00	1.00	1.00	1.00

0.97	0.98	0.99	0.99	1.00	1.00	1.00	1.00	1.00	1.00	1.00

0.96	0.94	0.91	0.99	0.95	0.96	1.00	1.00	1.00	1.00	1.00

0.63

0.65

0.54	0.53	0.63 0.67	0.67	0.72

0.25

**EnhA11**

0.97	0.89	0.92	0.94

1.00	0.99	0.99	0.95

1.00	1.00	1.00	0.99

1.00	1.00	1.00	1.00

1.00	1.00	1.00	0.99

1.00

1.00

1.00

1.00

1.00

0.98	0.93	0.97

0.86

0.91	0.92

0.81	0.83	0.89	0.83	0.84	0.85

0.89	0.82

0.83	0.83

0.50	0.50

0.58	0.59

0.74

**EnhA10**

0.91	0.88	0.88	0.95	0.96	0.92	0.91	0.91	0.95	0.95	0.97	0.99	0.97	1.00

0.80

0.81

0.84	0.80	0.88

**EnhA9**

0.99	1.00	1.00	1.00

0.97	0.99	0.99	0.98

1.00

1.00

0.94	0.94	0.94	0.90	0.97	0.98	0.95	0.93	0.97	0.97	0.98	0.98	0.99	0.96	0.99	0.99	1.00

0.45

**EnhA8**

0.78	0.97	0.98

0.99

1.00

0.73

0.81	0.81	0.80	0.82	0.84	0.83	0.79	0.77	0.75	0.76	0.77	0.78	0.79	0.76	0.73	0.83

0.54

**EnhA7**

0.27	0.35	0.37	0.37	0.81 0.82	0.81	0.85	0.87	1.00	1.00	1.00	1.00	1.00	1.00

**EnhA6**

0.77

**EnhA5**

0.96	0.95	0.95	0.99

0.96	0.99	0.96	0.98

0.99	0.99	1.00	1.00

1.00

1.00

1.00

0.98	1.00	0.98

0.98	0.99	0.99

0.98	0.99	0.99

**EnhA4**

0.91

0.97	0.95	0.97

1.00

0.99

0.92	0.90

**EnhA3**

0.98	1.00	0.98	0.99

1.00

**EnhA2**

**EnhA1**

0.34

**EnhWk8**

0.27	0.46	0.46	0.46	0.47 0.52	0.52	0.57	0.57	0.58	0.58 0.59	0.59	0.60	0.59	0.60	1.00	0.59

**EnhWk7**

0.97	0.99	0.99	1.00	1.00	1.00	1.00	0.99	1.00	1.00	0.99

0.87	0.87

0.91	0.91	0.93	0.94

0.54

**EnhWk6**

0.99	1.00	1.00	0.76

1.00	1.00	1.00	1.00

1.00	1.00	1.00	1.00

1.00	1.00	1.00	0.99

1.00	0.96	1.00	0.98

1.00

1.00

1.00

1.00

1.00

0.74

0.74	0.74	0.75	0.78	0.79	0.79	0.76	0.73	0.74	0.73	0.73	0.73	0.74	0.74	0.74	1.00

**EnhWk5**

0.53	0.55	0.68	0.71	0.72	0.70	0.70

0.50

0.38

0.40

**EnhWk4**

1.00	1.00	0.98	0.98

0.99	0.98	0.98	0.93

0.65	0.77	0.75	0.76	0.72	0.70

0.58

0.57

0.55

**EnhWk3**

0.86

**EnhWk2**

0.68

0.67

**EnhWk1**

0.92	0.89	0.94	0.96	0.94	1.00	1.00	1.00	1.00

0.86	0.92	0.90	0.97	0.99	0.95	0.93	0.88	0.91

0.84	0.93	0.95	0.97	0.94	0.99	0.89	0.90	0.94

0.99	0.99	0.99	0.99	0.99	0.99	1.00	1.00	0.93

0.66	0.74	0.58	0.97	0.91	0.88	0.71	0.64	0.89

0.63	0.64	0.73	0.88	0.82	0.87	0.87

0.74	0.72	0.74	0.82	0.82	0.86	0.91

0.85	0.88	0.85	0.93	0.96	0.92	0.90

0.61

0.74

0.76

0.39

**Acet8**

**Acet7**

0.98	1.00	0.98	1.00

1.00	0.92	0.99	0.98

1.00	1.00	1.00	1.00

0.99	0.99	0.95	0.97

1.00

1.00

1.00

1.00

1.00

1.00

1.00

0.91	0.90	0.90	0.92	0.90	0.89	0.90	0.96	0.93	0.95	0.92	0.93	0.95	0.89	0.96	0.88

0.94	0.92	0.94	0.94	0.97 0.97	0.97 0.96	0.95	0.93	0.92	0.91	0.96	0.94	0.99	0.99

0.85	0.90

0.94	0.92

**Acet6**

1.00	1.00	1.00	1.00

1.00

0.96	0.97	0.96	0.95	0.97	0.96	0.99	0.98	1.00	1.00	1.00	1.00	1.00	1.00	1.00

0.59	0.59	0.63

**Acet5**

0.99	1.00	0.99	0.98

1.00

0.98

0.93	0.91	0.90	0.94	0.91	0.93	0.87	0.88	0.91

0.65	0.67

0.74

**Acet4**

0.58

**Acet3**

0.90	0.88	0.91	0.92	0.92	0.93	0.98	0.97	0.95

0.97	0.92	0.96	0.98	0.97	0.95	0.97	0.96	0.97

0.69

0.61

0.60

**Acet2**

0.96	0.99	0.95	0.93	0.95	0.90

0.88	0.83	0.89

0.70	0.84	0.77	0.70	0.79	0.76	0.78

0.79	0.84	0.74	0.79	0.81	0.77	0.80

0.72	0.77	0.71	0.81	0.81	0.79	0.84

0.50

**Acet1**

0.50

0.46

**ReprPC9**

1.00	1.00	1.00	1.00

1.00	1.00	1.00	1.00

0.98	0.98	0.99	0.97

0.99	1.00	1.00	1.00

0.99	0.99	0.98	0.98

0.49	0.50	0.51	0.51	0.51	0.52	0.98	0.98	0.51	0.98	0.68	0.98	0.99	0.79	0.98	1.00	1.00

0.57	0.59	0.56	0.55	0.67	0.61	0.69	0.66	0.68	0.64	0.82	0.82	0.72	0.72	0.95	0.94	1.00

0.42

**ReprPC8**

0.89

0.90	0.73	0.86

0.73	0.75

**ReprPC7**

0.78

**ReprPC6**

0.89

**ReprPC5**

0.66

**ReprPC4**

0.87	0.95	0.95	0.96

0.83	0.92	0.95	0.95

0.96	0.89	0.96	0.94

0.91	0.81	0.87	0.81

0.75

**ReprPC3**

0.81

**ReprPC2**

1.00	1.00	1.00	1.00

1.00	1.00	1.00	1.00

1.00	0.99	0.98	0.99

1.00	0.99	1.00	1.00

0.98	1.00	1.00	1.00

0.99	0.97	0.97	0.95

1.00

1.00

1.00

1.00

1.00

1.00

1.00

1.00

1.00

1.00

1.00

1.00

0.96	0.99	0.98	0.96	0.97	0.97	1.00	1.00	0.99	0.99	0.98	0.98	1.00

0.99	0.95	0.99	0.99	0.99	0.98	0.99	0.98	0.98	0.99	0.98	0.98	1.00

0.95	0.94	0.96	0.96	0.97	0.97	0.98	0.96	0.97	0.98	0.98	0.97	0.99

0.95	0.96	0.96	0.93	0.95	0.94	0.97	0.99	0.98	0.99	0.99	1.00	1.00

0.93	0.91	0.95	0.94	0.96	0.96	0.95	0.96	0.94	0.96	0.99	0.99	1.00

0.96	0.92	0.96	0.95	0.98	0.97	0.98	0.92	0.98	0.98	0.94	0.93	0.95

0.92	0.95	0.91	0.92	0.91	0.88	0.88	0.92	0.89	0.89	0.95	0.96	0.93

0.97	0.97	0.97	0.96

0.91	0.94	0.90	0.91

0.88

**ReprPC1**

0.87	0.83	0.85	0.88	0.87	0.88	0.88

0.72	0.75	0.73	0.77

**HET9**

**HET8**

1.00	0.99	1.00	1.00

0.95	0.94	0.95	0.97

0.99	0.98	0.97	1.00

1.00

1.00

1.00

0.86	0.84	0.85	0.84	0.82	0.83	0.87	0.95	0.95	0.93	0.94

0.87	0.83	0.88	0.87	0.86 0.87	0.85	0.88	0.87	0.85	0.89

**HET7**

0.95	0.91	0.95	0.94	0.93	0.94	0.92

**HET6**

**HET5**

0.89	0.88	0.91	0.91	0.94 0.97	0.96 0.99	1.00 0.99	0.99	0.98	1.00	1.00	1.00	1.00	1.00	1.00

0.96	0.98	0.96	0.96	0.97	0.99	0.98	0.99	0.99	0.99	1.00	0.99	1.00	0.99	0.98	0.98	1.00	1.00

**HET4**

0.94

0.81	0.80	0.82	0.83

**HET3**

0.78

0.81	0.79	0.79	0.88	0.88	0.81	0.90	0.99

0.90	0.91	0.91	0.95	0.95 0.96	0.96 0.99

0.57

0.43	0.47

**HET2**

0.95	0.96	1.00	0.97

1.00	1.00	0.99	1.00

0.99	0.98	1.00	1.00

1.00	1.00	1.00	1.00

1.00	1.00	1.00	1.00

0.98	0.97	1.00	1.00

1.00

1.00

1.00

1.00

1.00

1.00

0.95	0.97	0.97	0.94	0.94	0.98	0.94	0.99	0.98	0.97	1.00	0.99	1.00	1.00

0.67

0.69

0.72	0.74

0.65

**HET1**

0.93	0.99	1.00

0.79

0.35	0.35	0.37

0.30	0.32	0.34	0.35

**Quies5**

0.95	0.90	0.90	1.00

0.98	1.00	1.00	0.99

0.80	0.82	0.81	0.85

**Quies4**

0.84

0.68	0.77	0.58	0.78

0.60

0.59	0.55	0.59	0.60

**Quies3**

**Quies2**

0.95	0.96	1.00	0.98

0.96	0.97	0.99	0.97

1.00

1.00

1.00

1.00

1.00

0.94	0.96	0.95	0.96	1.00	1.00	1.00	1.00	1.00

0.94	0.99	0.93	0.93	1.00	1.00	1.00	1.00	1.00

0.90	0.84	0.84	0.96	0.88	0.86	0.95	0.98	0.96

0.98	0.98	1.00	1.00	0.99	0.99	0.99	1.00	1.00

0.80	0.77	0.83	0.83	0.95

0.96	0.84	0.91	0.99	0.99

0.78	0.79	0.81	0.87

0.75	0.70	0.79	0.74

**Quies1**

0.43	0.45	0.43	0.43	0.44	0.48

**GapArtf3**

1.00	1.00	1.00	1.00

1.00	1.00	1.00	1.00

1.00

1.00

0.94	0.93	0.93	0.93	0.92	0.88	0.89	0.86	0.86	0.85	1.00	1.00

0.89	0.92	0.93	0.93	0.94	0.97	0.96	0.98	0.98	0.98	1.00	1.00

0.69	0.70	0.69	0.70	0.71	0.73

**GapArtf2**

0.97	0.95

0.87	0.90

1.00

0.98	0.99	1.00	1.00	1.00

0.50	0.52	0.50	0.51	0.56	0.53	0.54	0.56	0.57	0.61	0.59	0.60	0.60

**GapArtf1**

120

115

110

105

100

95

90

85

80

75

70

65

60

55

50

45

40

35

30

25

20

15

10

0.96

**Supplementary Figure 3: Evaluation of full-stack model’s number of states. (A)** AUCs of full-stack models with varying number of states in recovering external genomic annotations. The figure shows the AUC of full-stack models with 20, 40, 60, 80, 100, and 120 states at predicting the genomic locations of multiple different external genomic annotations (CpG Islands, lamina associated domains (laminB1lads), Exon, Gene body, TES, TSS, and TSS2kb regions) (**Methods**). As the number of chromatin states increases, the AUC increases, but the level of the AUC increases diminishes. **(B)** The estimated AIC-BIC curves for models with the number of states ranging from 10 to 120 (5 states apart). We calculated the AIC and BIC based on ChromHMM’s output reporting the log-likelihood of observed data for 300 1-Mb regions. Neg2_log_llh: -2 * negative log likelihood of observed data. (**C**) Maximum correlations of emission

parameters between each state in the 100-state model and any state for each other model. This is output from ChromHMM’s CompareModels command. Rows correspond to the states of the 100-state model. Columns correspond to models with varying numbers of states. Values are the maximum correlation of any state from the model in the column (with varying number of states) with the state from the 100-state in the row. The 100-state model is boxed. (**D**) Maximum correlations of emission parameters associated with H3K4me1 (an enhancer mark available in all cell types) and the binary vector indicating whether the cell type associated with an emission parameter is in a tissue group (1) or not (0). The rows correspond to different tissue groups from Roadmap Epigenomics Consortium (Roadmap Epigenomics Consortium et al, Nature 2015). The columns correspond to different models with varying numbers of states. The values show the maximum correlations mentioned above across all states within a model. The 100-state model is boxed.

We updated the results section to now read:

“We focused our analysis on a model with 100 states (**Methods**). The number of states is larger than typically used for models that generate per-cell-type annotations, which reflects the greater information available when defining states based on data from many cell types. This number of states was large enough to be able to capture some relatively cell-type-specific regulatory activity, while being small enough to give distinct biological interpretations to each state (**Supp. Fig. 3**). We denote the model’s output chromatin state annotation the ‘full-stack’ genome annotation.”

We updated the section ‘Methods/Choice of number of states’ which now reads:

###### “Choice of number of states

We trained full-stack models with 10-120 states, in 5 state increments, using the data and procedure outlined above. For each of these models, we calculated an estimated Akaike Information Criterion (AIC)

[6] and Bayesian Information Criterion (BIC) [7] value based on a subset of the genome (**Supp. Fig. 3**). AIC and BIC are calculated based on the log likelihood for 300 random 1Mb regions outputted by ChromHMM from the last training iteration. In general, both the AIC and BIC decrease as the numbers of states increase, but with diminishing improvements. We also applied the CompareModels command of ChromHMM [2] with the 100-state model as a reference model, which reports, for each state of the 100- state model, the maximum correlations of emission parameters between the state in the 100-state model and any state for each other models (**Supp. Fig. 3C**). We conducted a similar analysis with the emission parameters of H3K4me1, hence for each state in each model, we obtained emission probabilities of H3K4me1 in 127 cell types. For this analysis, for each of the 19 tissue groups previously defined [3], we calculated the correlation of each state’s H3K4me1 emission parameters with a binary vector indicating if the cell type in each parameter is in the tissue group (1) or not (0). We then report, for each tissue group, the maximum correlations among all states in each model (**Supp. Fig. 3D**). These analyses showed, for instance, that a state corresponding to Brain-specific enhancers in the 100-state model, EnhA6, was well captured in models with 55-states or more (correlation of >=0.98 with states in models with >=55 states and correlation >=0.78 for H3K4me1-emissions with the Brain binary vector). A state characterized as enhancers specific to Huvec cells in the 100-state model, EnhA20, was well captured in models with 100 or more states (correlation >=1.00 based on all marks’ emission parameters).

Additionally, for models with 20, 40, 60, 80, 100 and 120 states, we also produced genome annotations and then quantitatively compared the chromatin state annotations from models in terms of their power to predict locations of various other genomic annotations not used in the model training: Exon, Gene Body, TSS, TSS2kb, CpG Islands, TES, laminB1lads elements (listed in section *External Annotation Sources* section). Specifically, we evaluated the predictive power using the AUROCs that are calculated as described in a subsection below. Across different genomic contexts, as the number of full-stack states increased, the AUROC increased, but with diminishing improvements as the number of states increased (**Supp. Fig. 3A**).

To balance the additional information available in models with an increased number of states, while keeping the number of states manageable for interpretation and downstream analysis, we choose to focus on a model with 100 states. We note that this choice is greater than previously used for concatenated models with per-cell-type annotations [3,4,8], which reflects the additional information available for genome annotation based on the large number of datasets spanning many cell types that we are using.”

6. *The role of data imputation*

*Given the excellent work by Dr. Ernst on data imputation (i.e. ChromImpute), its role cannot be ignored. One of the "features" of full-stack models mentioned is essentially that the "data matrix" does not need to be complete for all marks and all cell types.*

*This, combined with the earlier demonstrated often superior quality of imputed data, begs the question: why not use imputed data to learn full-stack models?*

*Would this add much or, since both imputation and the current full stack models are based on the same "observed" data, are the stacked models sufficient?*

We thank the reviewer for the questions and the compliment on the prior work on data imputation.

As our end objective is a genome annotation based on all the data, we do not expect an obvious benefit from using imputed data here. A primary reason for that, as the reviewer notes, is that imputed data is based on the same data as the observed data. This contrasts with when we use imputed data for per cell type chromatin state annotations where information from observed data in other closely related cell types is being leveraged through imputed data.

Another reason why we would not currently advocate for the use of imputed data in this setting of stacked modeling is a technical concern related to the possibility of having artificially highly correlated imputed data across cell types. For marks for which it is difficult to get an informative imputation, which is more likely if the mark has been mapped in a limited number of cell types, the imputation may be close to that mark’s average signals across available cell types. This would lead to extremely correlated predictions for a mark across many cell types, likely far more than expected from the corresponding experimental data. Given the assumption of ChromHMM that the input marks’ signals are modeled by independent Bernoulli random variables conditioned on the state, the stacked modeling would potentially devote many states to modeling these heavily correlated tracks corresponding to relatively uninformative sets of imputed tracks.

Additionally, as the focus of this paper is a large-scale application of stacked modeling and observed data is still more commonly used in the field, we think that trying to apply stacked modeling to imputed data here would add complexity that distracts from the main focus of the manuscript. However, applying stacked modeling in conjunction with imputed data could be a possible direction for future work.

In response to this comment we have added the following text to the discussion

“Previously, concatenated models were applied to observed and imputed data [9], however, we chose not to use imputed data as input to the full-stack model since imputed data would still be based on the same observed input data used in stacked-modeling.”

*I think rather than going into great depth and detail on the various genomic annotation enrichment analyses (mentioned above), it might be more insightful to do a head-on comparison of concatenated vs. stacked models based on complete (possibly imputed) datasets, using the exact same input data.*

We thank the reviewer for the feedback. Reviewer 2 had suggested we reduce emphasis on concatenated vs. stacked model comparisons given that the results are not surprising, since the stacked modeling leverages a larger amount of input data from multiple cell types compared to per-cell-type concatenated model. We agree with Reviewer 2’s advice and feel it would be better to reduce emphasis on the concatenated vs. stacked model performance comparisons instead of expanding it with an additional one. Also, as discussed in comment #3, our point is that full-stack annotations have a complementary role to per-cell type annotations, and should not replace them.

In the revised manuscript, we have placed greater emphasis on the discussion of the biological implications of specific enrichment values (see response to reviewer 2). We also note that a goal of our paper is to introduce an annotation of the genome that is well-characterized by us and can later be a resource for other scientists for their own downstream analysis. Including many enrichment analyses allows gaining better insights into the biological implications of each state and to identify distinctions between states from the same group. These enrichments thus enhance the utility of the full-stack annotations. We have tried to keep most of the more detailed enrichment and characterization in our extensive supplement (**Supplementary Data Files 1-5**)**.**

7. *Extendability to new datasets and establishing standard annotations*

*Concatenated models offer an attractive opportunity to add additional biosamples at a later time, by re- using an existing model and calling states on unseen (but identically processed) data.*

*Because of the nature of data stacking, what possibilities do stacked models offer in this area?*

*This is an important point for establishing a set of "common" chromatin states in the community that can be used as a reference point for additional datasets.*

*What are the recommendations the authors can give the community in terms of choosing an appropriate chromatin state modeling approach?*

*Given the availability of increasing numbers of datasets (10s to 100s of thousands within the next few years), are stacked models the preferred approach?*

We thank the reviewer for the questions. As the current stacked model is already based on data and annotations from over 1000 experiments, our expectation is that a limited number of additional datasets would have limited influence on the overall model and annotations. The availability of large amounts of additional data can make occasional relearning a stacked model more worthwhile, and we have plans to do that in the future.

We note that while one can reuse the concatenated model to annotate new biosamples in cases where data of the marks used in the existing concatenated model are available in new biosamples, one cannot incorporate additional marks that were not part of the original model. This, in practice, either leads to data on additional chromatin marks never being incorporated into annotations based on the existing concatenated model, or necessitates relearning a concatenated model.

We also want to emphasize that we see stacked modeling as complementary to concatenated modeling with each having their own strengths. As discussed in our response to comment #3 above, the preferred approach should largely depend on the nature of the specific application. We expect the concatenated approach to be preferred in applications where the primary interest is annotations based on each biosample individually.

The stacked models would more likely be preferred for applications involving the joint analysis of multiple cell or tissue types.

In response to this comment, in the expanded paragraph in the Discussion section discussing trade-offs to using the stacked models we now state:

“An additional trade-off is that with the stacked model, it is not possible to incorporate additional data without relearning a model, while for a concatenated-model one can annotate a new cell based on an existing model, provided that the set of marks in the new cell type are the same as the existing model.”

Also in that paragraph we try to succinctly summarize when one approach might be preferred over another:

“Whether to use per-cell-type annotations or full-stack annotations will depend on the specific application. Per-cell-type annotations may be preferable when one is interested in studying a specific cell type, while full-stack annotations can be preferred in joint analyses of multiple cell types.”

The full paragraph is provided above in response to point #3.

Also in the last paragraph of the discussion, when discussing future work we now state:

“Given the increasing availability of epigenomic datasets [10], future work could also learn new stack- models incorporating such data. The state characterizations (**Supplementary Data File 1-5**) and analyses introduced through this work will be useful in interpreting biological implications of new models’ states.”

*In conclusion:*

*The authors have demonstrated, for the first time, the use of a stacked chromatin state modeling approach on 1000+ genome-wide datasets.*

*Although interesting in principle, it remains unclear what the actual practical benefits are in terms of biological interpretation.*

We thank the reviewer for recognizing the novelty of our large-scale application of the stacked modeling approach, and for acknowledging that the work is interesting at least in principle. We have revised the manuscript to include additional biological characterizations of the full-stack states, and interpretation of enrichment values including those with respect to genetic variation, repeat elements, gene ontology, sex chromosomes, polycomb repressed elements, and DNA methylation sites (please see response to reviewer 2). We have also added a figure highlighting the example of the HET9 state, a heterochromatin state that is associated with only the mark H3K9me3 and only in ESC & iPSC cells (HET9) despite H3K9me3 generally being associated with constitutive repression (discussed above in response to comment #3 above). This state was automatically detected de novo based on the stacked modeling, but such regions are generally not isolated with per-cell type approaches.

We also want to again emphasize that we see stacked-annotations as complementary to per-cell-type annotations and the preferred annotation approach will depend on the specific application including whether the analysis of interests is about activity across cell or tissue types or in individual ones. We hope that the clarifications and changes we have described above and in response to Reviewer 2 better highlight benefits of the annotations we provide.

Additionally, as this paper is providing a resource of the full-stack annotations and our detailed characterizations of the states (**Supplementary Data Files 1-3**), we expect the impact and benefits of this work will continue to increase as they are applied in other papers addressing a variety of scientific questions. We and our collaborators have already found these annotations useful for a variety of ongoing projects, partially reflected in available pre-prints. For example, Li et al, 2021 [11] apply the full stack state annotations to analyze the chromatin state context of CG sites that are associated with mammalian lifespan based on DNA methylation data representing over 100+ species and multiple tissue types. Horvath et al., 2021 [12] use these annotations to investigate the correlation between DNA methylation at conserved loci and gene expression across tissue types in horse. Arneson et al., 2021 [13] used full stack annotations to characterize the coverage of an array designed to measure DNA methylation at conserved loci across mammals.

In our revised discussion, we now highlight how the full-stack annotations are beginning to be used in other work with this sentence:

“We expect many applications of the full-stack annotations that we generated here and they have already begun to be applied in other work [11–13], which we expect to further elucidate the biological significance of different states.”

We have also changed the title of the manuscript “Universal annotation of the human genome through integration of over a thousand epigenomic datasets” to better differentiate the annotations produced here from per-cell type annotations.

*Minor points:*

*- In lifting over 200bp bin coordinates from hg19 to hg38, the authors find that 1,186,379 200bp segments were not lifted over.*

*That number seems very high and I'm curious to hear what liftOver parameters were used, i.e. requiring perfect mapping of 200bp coordinates, done in a reciprocal manner between the two assemblies?*

We thank the reviewer for the question. We used liftOver with default parameters for this procedure to map 200bp bins in hg19 to hg38, which is based on a minimum ratio of 0.95 of base mapping. We also excluded from annotating bases in hg38 if more than one bases in hg19 mapped to it.

The reason the reported number of unmapped bases seemed high is it included assembly gaps. The vast majority of unmapped bases from hg19 are in annotated assembly gaps (98.7%). These unmapped bases are also almost entirely in the GapArtf1 state in hg19 (99.6%). We have now also analyzed the annotation coverage of hg38 and report that 92.9% of bases on chr1-22, X, or Y are annotated to a state with that number increasing to 97.1% when excluding assembly gaps.

In response to this comment we have revised the methods section ‘Lifting chromatin state annotations to hg38’ which now reads:

“The full stack chromatin state annotations were learned directly in hg19, as this was the assembly for which uniformly processed data from the Roadmap Epigenomics integrative analysis was available. Learning full-stack annotation directly in hg19 allowed direct comparison with existing per-cell-type annotations inferred. We also generated a version of full-stack annotation in hg38 by lifting over the original annotation from hg19 to hg38. To do this, we first wrote the hg19 chromatin state annotation into .bed format such that each line corresponds to a 200bp interval. We then used the liftOver tool [14] with default parameters to generate the annotation in hg38. We did not annotate bases in hg38, if multiple bases in hg19 mapped to it. In total, there are 1,186,379 200-bp segments that were not mapped from hg19 to hg38, of

which 98.7% fall into an assembly gap and 99.6% fall into the full-stack state primarily associated assembly gaps (GapArtf1) (**Supp. Fig. 50**). In hg38 on chr1-22, X, and Y, 92.9% of bases are annotated to a state, and that number increases to 97.1% when excluding assembly gaps. We verified that we saw highly similar state fold enrichments for similar annotations between hg19 and hg38 (**Supp. Fig. 18**). The sources of external annotations from hg38 are outlined in section “External annotation sources” below.”

We have also added **Supplementary Figure 50** showing the state enrichments of the unmapped annotations and providing additional information about them with respect to annotated assembly gaps:

### A

| **state** | **pe rce nt_in_ge nom e** | **unm appe d_hg1 9 _to_hg38** |  | **pe rce nt_unm appe d_in_stat** |
| --- | --- | --- | --- | --- |
| GapArtf1 | 11.86 | 8.40 |  | 99.59 |
| GapArtf2 | 0.05 | 0.14 |  | 0.01 |
| GapArtf3 | 0.01 | 0.35 |  | 0.00 |
| Quies1 | 9.88 | 0.00 |  | 0.02 |
| Quies2 | 3.07 | 0.00 |  | 0.00 |
| Quies3 | 12.23 | 0.01 |  | 0.09 |
| Quies4 | 4.45 | 0.01 |  | 0.05 |
| Quies5 | 1.69 | 0.01 |  | 0.02 |
| HET1 | 0.71 | 0.00 |  | 0.00 |
| HET2 | 0.69 | 0.01 |  | 0.00 |
| HET3 | 1.36 | 0.00 |  | 0.00 |
| HET4 | 0.56 | 0.01 |  | 0.01 |
| HET5 | 0.25 | 0.01 |  | 0.00 |
| HET6 | 0.58 | 0.01 |  | 0.01 |
| HET7 | 1.02 | 0.01 |  | 0.01 |
| HET8 | 0.43 | 0.01 |  | 0.00 |
| HET9 | 1.00 | 0.02 |  | 0.02 |
| ReprPC1 | 0.19 | 0.01 |  | 0.00 |
| ReprPC2 | 0.32 | 0.01 |  | 0.00 |
| ReprPC3 | 1.11 | 0.00 |  | 0.00 |
| ReprPC4 | 3.93 | 0.00 |  | 0.01 |
| ReprPC5 | 0.63 | 0.00 |  | 0.00 |
| ReprPC6 | 1.51 | 0.00 |  | 0.01 |
| ReprPC7 | 0.61 | 0.00 |  | 0.00 |
| ReprPC8 | 0.48 | 0.01 |  | 0.00 |
| ReprPC9 | 0.37 | 0.01 |  | 0.00 |
| Acet1 | 0.18 | 0.02 |  | 0.00 |
| Acet2 | 0.85 | 0.00 |  | 0.00 |
| Acet3 | 2.65 | 0.00 |  | 0.01 |
| Acet4 | 0.40 | 0.00 |  | 0.00 |
| Acet5 | 0.86 | 0.00 |  | 0.00 |
| Acet6 | 0.43 | 0.00 |  | 0.00 |
| Acet7 | 0.28 | 0.00 |  | 0.00 |
| Acet8 | 0.56 | 0.00 |  | 0.00 |
| EnhWk1 | 1.54 | 0.00 |  | 0.00 |
| EnhWk2 | 0.35 | 0.00 |  | 0.00 |
| EnhWk3 | 0.83 | 0.00 |  | 0.00 |
| EnhWk4 | 2.22 | 0.00 |  | 0.00 |
| EnhWk5 | 0.99 | 0.01 |  | 0.01 |
| EnhWk6 | 0.59 | 0.01 |  | 0.01 |
| EnhWk7 | 0.48 | 0.00 |  | 0.00 |
| EnhWk8 | 1.37 | 0.00 |  | 0.00 |
| EnhA1 | 0.18 | 0.00 |  | 0.00 |
| EnhA2 | 0.33 | 0.00 |  | 0.00 |
| EnhA3 | 0.19 | 0.00 |  | 0.00 |
| EnhA4 | 0.30 | 0.00 |  | 0.00 |
| EnhA5 | 0.71 | 0.00 |  | 0.00 |
| EnhA6 | 0.56 | 0.00 |  | 0.00 |
| EnhA7 | 0.39 | 0.00 |  | 0.00 |
| EnhA8 | 0.25 | 0.01 |  | 0.00 |
| EnhA9 | 0.16 | 0.00 |  | 0.00 |
| EnhA10 | 0.39 | 0.00 |  | 0.00 |
| EnhA11 | 0.72 | 0.01 |  | 0.00 |
| EnhA12 | 0.33 | 0.00 |  | 0.00 |
| EnhA13 | 0.76 | 0.00 |  | 0.00 |
| EnhA14 | 0.37 | 0.00 |  | 0.00 |
| EnhA15 | 1.02 | 0.00 |  | 0.00 |
| EnhA16 | 0.65 | 0.01 |  | 0.01 |
| EnhA17 | 0.53 | 0.00 |  | 0.00 |
| EnhA18 | 0.46 | 0.00 |  | 0.00 |
| EnhA19 | 0.26 | 0.00 |  | 0.00 |
| EnhA20 | 0.35 | 0.00 |  | 0.00 |
| TxEnh1 | 0.39 | 0.00 |  | 0.00 |
| TxEnh2 | 0.39 | 0.00 |  | 0.00 |
| TxEnh3 | 0.25 | 0.00 |  | 0.00 |
| TxEnh4 | 0.27 | 0.00 |  | 0.00 |
| TxEnh5 | 0.50 | 0.00 |  | 0.00 |
| TxEnh6 | 0.19 | 0.00 |  | 0.00 |
| TxEnh7 | 0.27 | 0.00 |  | 0.00 |
| TxEnh8 | 0.24 | 0.00 |  | 0.00 |
| TxWk1 | 2.80 | 0.00 |  | 0.01 |
| TxWk2 | 0.84 | 0.00 |  | 0.00 |
| Tx1 | 0.82 | 0.00 |  | 0.00 |
| Tx2 | 1.58 | 0.00 |  | 0.00 |
| Tx3 | 0.51 | 0.00 |  | 0.00 |
| Tx4 | 0.47 | 0.00 |  | 0.00 |
| Tx5 | 0.94 | 0.00 |  | 0.00 |
| Tx6 | 1.11 | 0.00 |  | 0.00 |
| Tx7 | 0.82 | 0.00 |  | 0.00 |
| Tx8 | 0.68 | 0.00 |  | 0.00 |
| TxEx1 | 0.27 | 0.00 |  | 0.00 |
| TxEx2 | 0.56 | 0.00 |  | 0.00 |
| TxEx3 | 0.66 | 0.00 |  | 0.00 |
| TxEx4 | 0.10 | 0.00 |  | 0.00 |
| znf1 | 0.41 | 0.00 |  | 0.00 |
| znf2 | 0.15 | 0.00 |  | 0.00 |
| DNase1 | 0.20 | 0.01 |  | 0.00 |
| BivProm1 | 0.15 | 0.01 |  | 0.00 |
| BivProm2 | 0.16 | 0.01 |  | 0.00 |
| BivProm3 | 0.29 | 0.01 |  | 0.00 |
| BivProm4 | 0.13 | 0.01 |  | 0.00 |
| PromF1 | 0.20 | 0.00 |  | 0.00 |
| PromF2 | 0.14 | 0.00 |  | 0.00 |
| PromF3 | 0.15 | 0.00 |  | 0.00 |
| PromF4 | 0.19 | 0.00 |  | 0.00 |
| PromF5 | 0.14 | 0.00 |  | 0.00 |
| PromF6 | 0.13 | 0.00 |  | 0.00 |
| PromF7 | 0.16 | 0.00 |  | 0.00 |
| TSS1 | 0.12 | 0.01 |  | 0.00 |
| TSS2 | 0.11 | 0.03 |  | 0.00 |

**pe rce nt in** 100	7.665	100

**ge nom e**

**Enrichment of full-stack states with bases in hg19 that were unmapped to hg38, and analysis of unmapped bases with assembly gaps (all analyses in chr1-22,X,Y)**

### B

| # bp in hg38 not mapped to a state | 218,892,096 |
| --- | --- |
| # bp in hg38 not mapped to a state AND overlapping hg38  assembly gaps | 134,483,364 |
| Fraction of hg38 bases that are unannotated to a state after  liftOver from hg19 AND overlap hg38 assembly gaps | 0.61 |
| # bp in hg19 unmapped to hg38 | 237,275,800 |
| # bp in hg19 unmapped to hg38 AND overlapping assembly gaps | 234,342,292 |
| Fraction of unmapped_to_hg38 bp in hg19 that also overlap  hg19 assembly gaps | 0.99 |
| # bp in hg38 that are not assembly gaps | 2,937,659,104 |
| # bp in hg38 that are not assembly gaps AND annotated as a  state after liftOver | 2,853,200,372 |
| Fraction of hg38 bases that are non-assembly gaps AND  annotated to a state after liftOver | 0.97 |

**Supplementary Figure 50: Full-stack states enrichments of bases that were not lifted over from hg19 to hg38. (A)** The heatmap shows enrichment values for the full-stack states (rows) of genomic bases that were unmapped when lifting the state annotation from hg19 to hg38 (Methods). The first column shows the state label and the second column shows the percentage of the genome that each state covers. The third column shows enrichment values, colored such that highest enrichment values are colored red and lowest

ones are colored white. The fourth column shows the percentage of the unmapped regions (from hg19 to hg38) in each state. **(B)** Table showing details of numbers of bases involved in liftOver procedure, highlighting the overlap between the unmapped and unannotated regions with assembly gaps. As part of the liftOver procedure, bases in hg38 that are mapped to from multiple bases in hg19 are left unannotated to any state in hg38 (Methods). All results are reported in chromosomes 1-22, X, Y.

*- Supplementary Figure 8: labels are shown upside down. Legend: reference to EnhA7 state should be EnhA8.*

We thank the reviewer for pointing these out. We have updated our figure labels and the legend to this figure.

The updated figure and legend appear below:

**Sm. Muscle**

**HSC & B-cell**

**Epithelial**

**ESC**

**iPSC**

**Heart**

**Muscle**

**Mesench**

##### 25-state

**A d ip o se**

**IM R 90**

**M yo sat**

**Thym us**

**Neu ro sph**

**Blood & T-cell**

**Digestive**

**Other**

**Brain**

**ES-deriv**

**TssA**

**Prom U**

**Prom D 1**

**Prom D 2**

**Tx5p**

**Tx**

**Tx3p**

**Tx Wk**

**Tx Reg**

**TxEnh5p**

**TxEnh3p**

**TxEnhW**

**EnhA1**

**EnhA2**

**EnhAF**

**EnhW 1**

**EnhW 2**

**EnhAc**

**DNase**

**ZNF/Rpts**

**Het**

**Prom P**

**Prom Biv**

**ReprPC**

**Quies**

**Supplementary Figure 8: Full-stack states maximum-enrichments with annotated chromatin states in 127 reference epigenomes.** Each row corresponds to one of 127 reference epigenomes from the Roadmap Epigenomics Consortium (**Methods**). Each column corresponds to a state of the full-stack model. Each color entry corresponds to a reference epigenome- full-stack state combination. The color corresponds to the chromatin state from the 25-state model annotating the respective reference epigenome that is most enriched with the respective full-stack state. The figure highlights how some full-stack states are maximally enriched with the same per-cell-type chromatin states across all the reference epigenomes; for example,

**ENCODE2012**

**GapA rtf1 GapA rtf2 GapA rtf3 Q uies1**

**Q uies2 Q uies3 Q uies4 Q uies5 HET1**

**HET2 HET3 HET4 HET5 HET6 HET7 HET8 HET9**

**ReprPC1 ReprPC2 ReprPC3 ReprPC4 ReprPC5 ReprPC6 ReprPC7 ReprPC8 ReprPC9 Ac et1**

**Ac et2 Ac et3 Ac et4 Ac et5 Ac et6 Ac et7 Ac et8**

**EnhWk1**

**EnhWk2 EnhWk3 EnhWk4 EnhWk5 EnhWk6 EnhWk7 EnhWk8 EnhA 1**

**EnhA 2**

**EnhA 3**

**EnhA 4**

**EnhA 5**

**EnhA 6**

**EnhA 7**

**EnhA 8**

**EnhA 9**

**EnhA1 0**

**EnhA1 1**

**EnhA1 2**

**EnhA1 3**

**EnhA1 4**

**EnhA1 5**

**EnhA1 6**

**EnhA1 7**

**EnhA1 8**

**EnhA1 9**

**EnhA2 0 TxEnh1 TxEnh2 TxEnh3 TxEnh4 TxEnh5 TxEnh6 TxEnh7 TxEnh8 TxW k1 TxW k2 Tx1**

**Tx2**

**Tx3 Tx4 Tx5 Tx6 Tx7 Tx8** TxEx1

TxEx2

TxEx3 TxEx4

**znf1 znf2**

**DNase 1 BivProm 1 BivProm 2 BivProm 3 BivProm 4**

**PromF1**

**PromF2**

**PromF3**

**PromF4**

**PromF5**

**PromF6**

**PromF7 TSS1 TSS2**

states ZNF1 and ZNF2 are maximally enriched with ZNF Gene state in all 127 reference epigenomes’ 25- state per-cell-type annotation. At the same time, other full-stack states are enriched for distinct per-cell- type states, for example state EnhA8-- characterized as a blood enhancer state based on emission probabilities-- is most enriched with activate/flanking enhancer in cell types of the groups Blood&Tcell, HSC&B-cell, while being most enriched with poised promoter and weak enhancer states in other cell types. Detailed description of each full-stack state enrichment patterns with per-cell-type states can be found in **Supplementary Data 3**.

- *Supplementary Figure 12: since there are still significant amounts of DNase signal in the 'Closed' state, this should be worded differently.*

We thank the reviewer for the suggestion. We have reworded this to eliminate the word ‘closed’. Now, the figure looks as follows:

CTCF + open chromatin elements enrichments	CTCF + *limited* open chromatin elements enrichments

**HET9**

**ReprPC1 ReprPC2 ReprPC3 ReprPC4**

1.0

0.2

0.3

1.1

3.9

0.5	0.4	0.6	0.7

1.1

1.4

1.2

0.5

0.2

0.7

1.4

2.2

1.6

1.3

1.0

1.2

1.3

0.6	0.6	0.6

0.5	0.7

0.3	1.3

0.8	1.7

1.0	1.5

0.6	0.7

**Enh A 8**

**Enh A 9 EnhA 10**

**EnhA 11**

**EnhA 12**

**EnhA 13**

**EnhA 14**

**EnhA 15**

**EnhA 16**

0.3	0.7	2.1

0.2	0.4	1.7

0.4	2.6	3.0

0.7	1.1	1.2

0.3	3.4	3.1

0.8	1.2	1.2

0.4	2.7	2.6

1.0	1.2	1.1

2.4

1.7

2.8

1.2

2.4

1.1

2.5

1.2

2.1	2.4	1.8

1.2	1.2	1.0

2.8	3.7	2.9

1.2	1.4	1.4

2.9	3.0	2.8

1.2

1.2

0.8

1.2

2.9

1.3

0.6 10.0	7.0	7.4	8.9	9.8

1.2

2.2

1.4

7.1

**BivProm 3**	0.3	5.9	1.5	4.9	3.3	4.3	3.5

**B iv Prom 4**	0.1	3.4	1.0	2.4	2.1	1.4	2.8

**Prom F1**	0.2	7.4	2.5	6.4	3.3	7.5	3.8

**state**

**Genome %**

**Gm 12878 _Ctcf**

**H1 hesc_Ctcf**

**Helas3 _Ctcf**

**Hepg2 _Ctcf**

**Huvec_Ctcf**

**K562 _Ctcf**

| **state** | **Genome %** | **Gm 12878** | **H 1 hesc** | **Helas3** | **Hepg2** | **Huvec** | **K562** |
| --- | --- | --- | --- | --- | --- | --- | --- |
| **GapArtf1** | 11.9 | 0.0 | 0.0 | 0.0 | 0.0 | 0.0 | 0.0 |
| **GapArtf2** | 0.1 | 5.5 | 3.1 | 2.7 | 4.4 | 3.9 | 4.0 |
| **GapArtf3** | 0.0 | 2.7 | 1.5 | 1.5 | 3.8 | 0.8 | 1.4 |
| **Q uies1** | 9.9 | 0.0 | 0.0 | 0.0 | 0.0 | 0.0 | 0.0 |
| **Quies2** | 3.1 | 0.0 | 0.1 | 0.1 | 0.0 | 0.0 | 0.1 |
| **Quies3** | 12.2 | 0.0 | 0.0 | 0.1 | 0.0 | 0.0 | 0.1 |
| **Quies4** | 4.5 | 0.0 | 0.0 | 0.0 | 0.0 | 0.0 | 0.0 |
| **Quies5** | 1.7 | 0.0 | 0.0 | 0.0 | 0.0 | 0.0 | 0.0 |
| **HET1** | 0.7 | 0.1 | 1.6 | 0.4 | 0.4 | 0.1 | 0.3 |
| **HET2** | 0.7 | 0.1 | 2.0 | 0.2 | 0.5 | 0.0 | 0.4 |
| **HET3** | 1.4 | 0.0 | 0.0 | 0.0 | 0.0 | 0.0 | 0.0 |
| **HET4** | 0.6 | 0.2 | 0.1 | 0.1 | 0.2 | 0.2 | 0.3 |
| **HET5** | 0.2 | 1.0 | 2.1 | 1.1 | 1.9 | 1.0 | 2.0 |
| **HET6** | 0.6 | 0.1 | 0.6 | 0.2 | 0.5 | 0.1 | 0.4 |
| **HET7** | 1.0 | 0.0 | 0.3 | 0.1 | 0.2 | 0.0 | 0.2 |
| **HET8** | 0.4 | 1.7 | 1.9 | 1.6 | 1.9 | 1.2 | 1.9 |
| **HET9** | 1.0 | 0.1 | 0.1 | 0.1 | 0.1 | 0.0 | 0.1 |
| **ReprPC 1** | 0.2 | 0.5 | 1.0 | 2.3 | 1.2 | 0.3 | 1.9 |
| **ReprPC 2** | 0.3 | 0.3 | 0.6 | 0.7 | 0.5 | 0.3 | 0.6 |
| **ReprPC 3** | 1.1 | 0.1 | 0.4 | 0.2 | 0.2 | 0.1 | 0.3 |
| **ReprPC 4** | 3.9 | 0.0 | 0.1 | 0.0 | 0.0 | 0.0 | 0.0 |
| **ReprPC 5** | 0.6 | 0.3 | 3.2 | 1.0 | 0.8 | 0.2 | 0.9 |
| **ReprPC 6** | 1.5 | 0.1 | 0.9 | 0.3 | 0.4 | 0.1 | 0.4 |
| **ReprPC 7** | 0.6 | 1.0 | 6.5 | 2.9 | 3.4 | 1.1 | 3.1 |
| **ReprPC 8** | 0.5 | 0.2 | 0.3 | 0.3 | 0.1 | 0.0 | 0.3 |
| **ReprPC 9** | 0.4 | 0.1 | 0.3 | 0.3 | 0.3 | 0.3 | 0.3 |
| **Acet1** | 0.2 | 2.0 | 7.0 | 2.8 | 4.1 | 1.6 | 3.1 |
| **Acet2** | 0.9 | 0.4 | 1.9 | 0.7 | 0.9 | 0.3 | 0.9 |
| **Acet3** | 2.6 | 0.0 | 0.2 | 0.1 | 0.1 | 0.1 | 0.2 |
| **Acet4** | 0.4 | 0.6 | 2.4 | 2.0 | 1.3 | 1.0 | 1.4 |
| **Acet5** | 0.9 | 0.1 | 0.2 | 0.3 | 0.2 | 0.3 | 0.2 |
| **Acet6** | 0.4 | 0.2 | 0.5 | 0.8 | 0.3 | 0.7 | 0.3 |
| **Acet7** | 0.3 | 1.6 | 3.6 | 3.2 | 2.5 | 1.7 | 1.8 |
| **Acet8** | 0.6 | 0.2 | 1.3 | 0.6 | 0.6 | 0.2 | 0.6 |
| **EnhWk1** | 1.5 | 0.1 | 0.0 | 0.3 | 0.1 | 0.5 | 0.2 |
| **EnhWk2** | 0.4 | 1.9 | 5.6 | 4.3 | 4.2 | 2.7 | 3.4 |
| **EnhWk3** | 0.8 | 0.2 | 0.8 | 0.6 | 0.6 | 0.6 | 0.5 |
| **EnhWk4** | 2.2 | 0.1 | 0.1 | 0.1 | 0.1 | 0.3 | 0.1 |
| **EnhWk5** | 1.0 | 0.3 | 0.2 | 0.2 | 0.3 | 0.3 | 0.3 |
| **EnhWk6** | 0.6 | 1.0 | 0.5 | 0.6 | 0.5 | 0.7 | 0.8 |
| **EnhWk7** | 0.5 | 0.9 | 0.3 | 0.5 | 0.4 | 0.4 | 0.6 |
| **EnhWk8** | 1.4 | 0.1 | 1.2 | 0.1 | 0.3 | 0.2 | 0.2 |
| **EnhA 1** | 0.2 | 6.8 | 5.3 | 6.0 | 5.9 | 4.4 | 5.0 |
| **EnhA 2** | 0.3 | 1.6 | 3.0 | 3.1 | 2.6 | 3.9 | 2.6 |
| **EnhA 3** | 0.2 | 16.5 | 9.9 | 10.7 | 12.3 | 10.0 | 14.0 |
| **EnhA 4** | 0.3 | 1.4 | 1.4 | 2.9 | 1.8 | 1.9 | 1.7 |
| **EnhA 5** | 0.7 | 0.6 | 0.4 | 2.0 | 0.8 | 2.3 | 0.8 |
| **EnhA 6** | 0.6 | 0.5 | 1.1 | 0.8 | 0.9 | 0.6 | 0.9 |
| **EnhA 7** | 0.4 | 3.0 | 4.8 | 2.7 | 3.3 | 1.5 | 3.6 |
| **EnhA 8** | 0.3 | 1.4 | 3.1 | 2.3 | 2.0 | 1.7 | 2.3 |
| **EnhA 9** | 0.2 | 2.5 | 5.7 | 5.7 | 5.3 | 4.5 | 3.1 |
| **EnhA 10** | 0.4 | 3.4 | 1.9 | 3.1 | 2.3 | 3.4 | 3.0 |
| **EnhA 11** | 0.7 | 0.4 | 0.3 | 0.4 | 0.5 | 0.3 | 0.5 |
| **EnhA 12** | 0.3 | 1.4 | 3.8 | 3.9 | 2.4 | 1.3 | 2.0 |
| **EnhA 13** | 0.8 | 0.2 | 0.3 | 0.6 | 0.2 | 0.2 | 0.2 |
| **EnhA 14** | 0.4 | 1.0 | 2.4 | 2.3 | 1.7 | 1.0 | 1.8 |
| **EnhA 15** | 1.0 | 0.1 | 0.2 | 0.3 | 0.4 | 0.1 | 0.3 |
| **EnhA 16** | 0.6 | 5.2 | 5.7 | 5.7 | 5.8 | 6.2 | 6.9 |
| **EnhA 17** | 0.5 | 1.5 | 3.4 | 1.7 | 1.9 | 2.2 | 1.8 |
| **EnhA 18** | 0.5 | 0.4 | 2.9 | 0.7 | 1.1 | 0.3 | 0.9 |
| **EnhA 19** | 0.3 | 3.1 | 3.9 | 5.2 | 4.4 | 3.4 | 4.1 |
| **EnhA 20** | 0.3 | 0.1 | 0.1 | 0.3 | 0.2 | 0.6 | 0.2 |
| **TxEnh1** | 0.4 | 0.4 | 0.3 | 0.7 | 0.5 | 0.9 | 0.5 |
| **TxEnh2** | 0.4 | 1.0 | 1.0 | 1.3 | 1.3 | 1.6 | 1.4 |
| **TxEnh3** | 0.2 | 1.1 | 2.7 | 1.7 | 1.6 | 1.0 | 0.9 |
| **TxEnh4** | 0.3 | 3.8 | 7.9 | 7.4 | 6.2 | 5.3 | 2.8 |
| **TxEnh5** | 0.5 | 2.0 | 6.9 | 4.2 | 5.3 | 3.2 | 3.7 |
| **TxEnh6** | 0.2 | 2.9 | 4.5 | 4.2 | 4.1 | 3.6 | 2.5 |
| **TxEnh7** | 0.3 | 3.1 | 4.9 | 5.2 | 4.5 | 4.1 | 3.0 |
| **TxEnh8** | 0.2 | 4.1 | 4.3 | 5.2 | 4.6 | 4.3 | 3.5 |
| **TxWk1** | 2.8 | 0.1 | 0.1 | 0.1 | 0.1 | 0.1 | 0.1 |
| **TxWk2** | 0.8 | 0.3 | 2.2 | 0.9 | 1.3 | 0.5 | 1.1 |
| **Tx1** | 0.8 | 0.2 | 0.2 | 0.3 | 0.2 | 0.3 | 0.3 |
| **Tx2** | 1.6 | 0.1 | 0.1 | 0.1 | 0.1 | 0.2 | 0.1 |
| **Tx3** | 0.5 | 0.5 | 1.6 | 1.0 | 1.0 | 0.5 | 0.9 |
| **Tx4** | 0.5 | 0.3 | 0.2 | 0.3 | 0.3 | 0.4 | 0.3 |
| **Tx5** | 0.9 | 0.1 | 0.5 | 0.4 | 0.3 | 0.2 | 0.3 |
| **Tx6** | 1.1 | 0.1 | 0.0 | 0.1 | 0.1 | 0.1 | 0.1 |
| **Tx7** | 0.8 | 0.2 | 0.1 | 0.3 | 0.2 | 0.3 | 0.2 |
| **Tx8** | 0.7 | 1.6 | 1.1 | 1.6 | 1.7 | 2.1 | 1.5 |
| TxEx1 | 0.3 | 0.3 | 0.6 | 0.4 | 0.2 | 0.3 | 0.2 |
| TxEx2 | 0.6 | 0.2 | 1.2 | 0.8 | 0.6 | 0.3 | 0.2 |
| TxEx3 | 0.7 | 0.2 | 2.3 | 1.3 | 1.3 | 0.6 | 0.9 |
| TxEx4 | 0.1 | 2.4 | 3.3 | 3.4 | 2.4 | 2.4 | 1.1 |
| **znf1** | 0.4 | 0.2 | 0.5 | 0.3 | 0.4 | 0.3 | 0.3 |
| **znf2** | 0.2 | 0.3 | 1.2 | 0.7 | 0.7 | 0.6 | 0.9 |
| **DNase 1** | 0.2 | 260.3 | 130.8 | 187.1 | 202.5 | 239.9 | 214.8 |
| **BivProm 1** | 0.1 | 6.2 | 0.7 | 4.7 | 4.3 | 3.3 | 5.0 |
| **BivProm 2** | 0.2 | 3.5 | 1.0 | 3.9 | 4.4 | 2.6 | 4.4 |
| **BivProm 3** | 0.3 | 4.8 | 7.7 | 6.6 | 6.6 | 5.1 | 6.6 |
| **BivProm 4** | 0.1 | 2.9 | 2.5 | 2.7 | 3.1 | 2.4 | 3.4 |
| **Prom F1** | 0.2 | 44.4 | 23.9 | 31.9 | 27.5 42.3 | | 28.1 |
| **Prom F2** | 0.1 | 8.0 | 6.2 | 6.5 | 4.8 | 6.5 | 5.1 |
| **Prom F3** | 0.2 | 0.1 | 0.2 | 0.3 | 0.4 | 0.0 | 0.3 |
| **Prom F4** | 0.2 | 0.0 | 0.0 | 0.0 | 0.1 | 0.0 | 0.1 |
| **Prom F5** | 0.1 | 2.8 | 0.3 | 2.9 | 2.2 | 0.3 | 2.9 |
| **Prom F6** | 0.1 | 0.0 | 0.1 | 0.0 | 0.1 | 0.0 | 0.1 |
| **Prom F7** | 0.2 | 0.2 | 0.6 | 0.2 | 0.2 | 0.3 | 0.2 |
| **TSS1** | 0.1 | 0.3 | 0.8 | 0.6 | 0.5 | 0.0 | 0.9 |
| **TSS2** | 0.1 | 16.3 | 8.8 | 8.9 | 10.9 | 8.2 | 12.2 |
| Base | 100 | 0.2 | 0.4 | 0.3 | 0.2 | 0.3 | 0.2 |

| **GapArtf1** | 11.9 | 0.0 | 0.0 | 0.0 | 0.0 | 0.0 | 0.0 |
| --- | --- | --- | --- | --- | --- | --- | --- |
| **GapArtf2** | 0.1 | 0.7 | 0.6 | 0.6 | 0.9 | 0.6 | 0.4 |
| **GapArtf3** | 0.0 | 0.3 | 0.0 | 0.4 | 0.3 | 0.0 | 0.1 |
| **Q uies1** | 9.9 | 0.2 | 0.2 | 0.2 | 0.2 | 0.2 | 0.1 |
| **Q uies2** | 3.1 | 0.5 | 1.0 | 0.5 | 0.7 | 0.3 | 0.5 |
| **Q uies3** | 12.2 | 0.5 | 0.4 | 0.5 | 0.5 | 0.8 | 0.6 |
| **Q uies4** | 4.5 | 0.2 | 0.2 | 0.2 | 0.2 | 0.2 | 0.2 |
| **Q uies5** | 1.7 | 0.4 | 0.3 | 0.4 | 0.3 | 0.3 | 0.4 |
| **HET1** | 0.7 | 2.0 | 4.0 | 1.9 | 2.8 | 1.1 | 1.6 |
| **HET2** | 0.7 | 1.3 | 2.9 | 1.3 | 2.0 | 0.5 | 1.9 |
| **HET3** | 1.4 | 0.3 | 0.3 | 0.3 | 0.4 | 0.4 | 0.4 |
| **HET4** | 0.6 | 0.6 | 0.5 | 0.6 | 0.7 | 0.7 | 0.7 |
| **HET5** | 0.2 | 2.3 | 2.5 | 2.4 | 2.8 | 2.6 | 2.8 |
| **HET6** | 0.6 | 1.2 | 1.8 | 1.4 | 2.0 | 1.0 | 1.7 |
| **HET7** | 1.0 | 0.8 | 1.4 | 1.0 | 1.4 | 0.8 | 1.2 |
| **HET8** | 0.4 | 2.5 | 2.5 | 2.6 | 2.9 | 2.5 | 2.9 |

| **ReprPC5** | 0.6 | 2.2 | 2.9 | 2.4 | 2.0 | 1.8 | 1.9 |
| --- | --- | --- | --- | --- | --- | --- | --- |
| **ReprPC6** | 1.5 | 1.4 | 2.4 | 1.6 | 1.7 | 1.3 | 1.8 |
| **ReprPC7** | 0.6 | 3.9 | 3.4 | 3.7 | 2.9 | 3.8 | 2.7 |
| **ReprPC8** | 0.5 | 1.6 | 1.6 | 1.6 | 1.4 | 1.3 | 1.7 |
| **ReprPC9** | 0.4 | 1.1 | 0.5 | 1.1 | 1.0 | 1.2 | 1.1 |
| **Acet1** | 0.2 | 3.5 | 4.1 | 3.3 | 3.8 | 3.5 | 2.8 |
| **Acet2** | 0.9 | 2.9 | 3.7 | 2.8 | 3.3 | 2.6 | 2.8 |
| **Acet3** | 2.6 | 0.9 | 1.2 | 1.0 | 1.1 | 1.0 | 1.1 |
| **Acet4** | 0.4 | 3.2 | 3.5 | 2.9 | 3.0 | 3.1 | 2.8 |
| **Acet5** | 0.9 | 1.3 | 1.4 | 1.4 | 1.4 | 1.5 | 1.4 |
| **Acet6** | 0.4 | 1.5 | 1.4 | 1.4 | 1.5 | 1.4 | 1.5 |
| **Acet7** | 0.3 | 2.8 | 2.5 | 1.7 | 2.0 | 1.7 | 2.4 |
| **Acet8** | 0.6 | 1.8 | 2.5 | 1.9 | 2.0 | 1.9 | 2.1 |
| **EnhWk1** | 1.5 | 1.3 | 1.0 | 1.2 | 1.2 | 1.7 | 1.2 |
| **EnhWk2** | 0.4 | 3.6 | 2.8 | 3.3 | 2.4 | 3.7 | 2.6 |
| **EnhWk3** | 0.8 | 1.9 | 2.2 | 1.9 | 1.9 | 2.0 | 2.1 |
| **EnhWk4** | 2.2 | 1.4 | 1.2 | 1.1 | 1.3 | 1.5 | 1.1 |
| **EnhWk5** | 1.0 | 1.5 | 1.2 | 1.2 | 1.3 | 1.7 | 1.5 |
| **EnhWk6** | 0.6 | 1.3 | 1.5 | 1.7 | 1.6 | 2.0 | 1.8 |
| **EnhWk7** | 0.5 | 0.5 | 1.3 | 1.4 | 1.6 | 1.8 | 1.7 |
| **EnhWk8** | 1.4 | 2.0 | 1.9 | 1.7 | 2.1 | 1.9 | 1.7 |
| **Enh A 1** | 0.2 | 3.0 | 1.0 | 1.5 | 1.2 | 1.2 | 1.8 |
| **Enh A 2** | 0.3 | 4.6 | 3.4 | 3.6 | 3.5 | 3.6 | 4.1 |
| **Enh A 3** | 0.2 | 3.3 | 3.6 | 1.9 | 3.0 | 1.0 | 3.3 |
| **Enh A 4** | 0.3 | 2.7 | 2.0 | 1.1 | 2.2 | 1.1 | 2.3 |
| **Enh A 5** | 0.7 | 2.7 | 2.1 | 1.9 | 2.5 | 2.1 | 2.2 |

| **EnhA 17** | 0.5 | 5.0 | 2.6 | 4.2 | 4.6 | 4.8 | 4.4 |
| --- | --- | --- | --- | --- | --- | --- | --- |
| **EnhA 18** | 0.5 | 2.9 | 1.1 | 2.6 | 3.0 | 2.6 | 2.8 |
| **EnhA 19** | 0.3 | 5.2 | 0.7 | 4.3 | 3.8 | 5.3 | 3.8 |
| **EnhA 20** | 0.3 | 1.2 | 0.9 | 1.0 | 1.0 | 0.2 | 1.1 |
| **TxEnh1** | 0.4 | 1.4 | 0.9 | 1.2 | 1.0 | 1.0 | 1.1 |
| **TxEnh2** | 0.4 | 0.6 | 0.9 | 1.1 | 0.7 | 1.0 | 0.7 |
| **TxEnh3** | 0.2 | 0.4 | 1.1 | 0.9 | 0.6 | 0.8 | 0.5 |
| **TxEnh 4** | 0.3 | 0.4 | 1.0 | 1.4 | 0.2 | 0.1 | 0.6 |
| **TxEnh5** | 0.5 | 2.1 | 2.2 | 2.9 | 1.9 | 2.1 | 2.2 |
| **TxEnh 6** | 0.2 | 0.6 | 1.8 | 2.1 | 1.2 | 1.2 | 1.2 |
| **TxEnh7** | 0.3 | 1.6 | 1.3 | 1.9 | 1.2 | 1.8 | 1.3 |
| **TxEnh8** | 0.2 | 0.9 | 1.5 | 1.5 | 0.7 | 0.8 | 1.3 |
| **TxW k1** | 2.8 | 0.5 | 0.5 | 0.6 | 0.5 | 0.7 | 0.6 |
| **TxW k2** | 0.8 | 1.6 | 2.1 | 1.9 | 1.7 | 1.7 | 1.8 |
| **Tx1** | 0.8 | 0.5 | 0.5 | 0.6 | 0.4 | 0.7 | 0.5 |
| **Tx2** | 1.6 | 0.5 | 0.5 | 0.6 | 0.5 | 0.8 | 0.6 |
| **Tx3** | 0.5 | 1.3 | 1.6 | 1.5 | 1.3 | 1.5 | 1.3 |
| **Tx4** | 0.5 | 0.3 | 0.5 | 0.5 | 0.2 | 0.4 | 0.3 |
| **Tx5** | 0.9 | 0.3 | 0.7 | 0.7 | 0.4 | 0.4 | 0.6 |
| **Tx6** | 1.1 | 0.3 | 0.3 | 0.4 | 0.3 | 0.6 | 0.3 |
| **Tx7** | 0.8 | 0.2 | 0.3 | 0.3 | 0.1 | 0.2 | 0.2 |
| **Tx8** | 0.7 | 1.6 | 1.8 | 1.9 | 1.5 | 1.9 | 1.8 |
| TxEx1 | 0.3 | 0.1 | 0.2 | 0.2 | 0.0 | 0.0 | 0.1 |
| TxEx2 | 0.6 | 0.0 | 0.3 | 0.4 | 0.0 | 0.0 | 0.1 |
| TxEx3 | 0.7 | 0.4 | 1.0 | 1.0 | 0.4 | 0.2 | 0.6 |
| TxEx4 | 0.1 | 0.1 | 0.3 | 0.3 | 0.2 | 0.1 | 0.2 |
| **znf1** | 0.4 | 0.5 | 0.8 | 0.9 | 0.8 | 0.8 | 0.8 |
| **znf2** | 0.2 | 0.4 | 0.5 | 0.7 | 0.4 | 0.4 | 0.6 |
| **DNase 1** | 0.2 | 31.7 | 32.8 | 26.5 | 37.6 | 18.6 | 28.5 |
| **BivProm 1** | 0.1 | 1.4 | 0.0 | 3.3 | 0.9 | 0.4 | 1.2 |
| **BivProm 2** | 0.2 | 1.7 | 0.1 | 2.7 | 1.3 | 0.8 | 1.4 |

| **Prom F2** | 0.1 | 0.7 | 0.6 | 1.4 | 0.6 | 0.3 | 0.9 |
| --- | --- | --- | --- | --- | --- | --- | --- |
| **Prom F3** | 0.2 | 0.0 | 0.0 | 0.1 | 0.0 | 0.0 | 0.0 |
| **Prom F4** | 0.2 | 0.0 | 0.0 | 0.0 | 0.0 | 0.0 | 0.0 |
| **Prom F5** | 0.1 | 0.6 | 0.0 | 1.7 | 0.4 | 0.1 | 1.0 |
| **Prom F6** | 0.1 | 0.0 | 0.0 | 0.0 | 0.0 | 0.0 | 0.0 |
| **Prom F7** | 0.2 | 0.1 | 0.2 | 0.1 | 0.1 | 0.2 | 0.3 |
| **TSS1** | 0.1 | 0.0 | 0.0 | 0.3 | 0.1 | 0.0 | 0.1 |
| **TSS2** | 0.1 | 1.8 | 1.0 | 2.7 | 1.3 | 0.7 | 1.6 |
|  | 100 | 0.6 | 0.7 | 0.8 | 0.6 | 0.6 | 0.7 |

| **Enh A 6** | 0.6 | 2.7 | 2.3 2.3 2.3 | 2.7 | 2.5 |
| --- | --- | --- | --- | --- | --- |
| **Enh A 7** | 0.4 | 3.3 | 3.6 4.1 3.7 | 4.3 | 3.2 |

Reviewer #2: *The authors provide a full-stack ChromHMM annotation across 127 human samples, which gives a unified view of ~1,000 epigenomic data sets. With their detailed characterization of 100 chromatin states, which are clustered into 16 state groups, some state groups show signals across all cell types while other state groups such as enhancers show cell type specificity. While the full-stack model agrees with previously published cell-type-specific chromatin states, it also provides more detailed annotations on repeat elements, structural variation, sequence conservation, and genetic variation.*

*Overall, this full-stack chromatin state annotation has advantages over the cell-type-specific annotations in pan-tissue-type applications that do not require detailed chromatin state annotation in a particular cell type. These full-stack models are difficult to build and annotate, and the authors did a good job validating the quality of the model using the extensive infrastructure built up with their earlier work for the Epigenomic Roadmap project. Therefore, I suggest that the paper be accepted for publication after responding to comments below.*

We thank the reviewer for the summary and the positive comments. We appreciate the reviewer for recognizing the effort involved in building and annotating these models, and that the model we presented can be useful in pan-tissue-type analyses. We also thank the reviewer for the comments below, which has led to a strengthened paper. We discuss below our response to each of those comments.

*Major comments:*

*It is the underlying assumption of ChromHMM that each genomic bin can only be assigned to one chromatin state. When we consider the concatenated model, for the genomic bins that are in one state (say, TSS, the gene is expressed) in one sample but in another state in another sample (say, ReprPC, the gene is repressed), and in a third state in yet another sample (say, Bivalent TSS, the gene is poised for expression), what would this genomic bin be assigned to in the full-stack model? Can the authors perform a detailed analysis on this subset of genomic bins so that the reader can get a better understanding of the full-stack model?*

We thank the reviewer for the suggestions. We agree it is informative to understand how the states of the full stack model correspond to the potential wide variety of states across individual cell types. Our original submission had one supplementary figure along these lines, and we have conducted new analyses to further address this point. In the original submission, we analyzed the enrichments of full-stack states with per- cell type chromatin state annotations from a 25-state concatenated model for 127 cell types [9]. For each cell type, we show the state in the 25-state model that is most enriched with each full-stack state. In the revised manuscript, this is now shown in **Supp. Fig. 8**, where each column corresponds to each reference epigenome, each row corresponds to a full-stack state, and the cells’ color shows the state in the 25-state model that is most enriched in the corresponding cell-type – full-stack state combination. This shows that in the same full-stack state, different per-cell-type concatenated states can be maximally enriched among the different reference epigenomes.

We had also included a version of this figure in our **Supplementary Data File 3** (sheet ‘25_state_most_enriched’). We highlight that on the excel sheet we had included a column with our comments on patterns of enrichment between each full-stack state and the per-cell-type chromatin state annotations. The description of a number of full-stack states reflects that each full-stack state can be

associated with different per-cell-type states, along the lines of the reviewer’s comment. This sheet is one example of detailed characterization of the full-stack states we provide, as our intention is to enhance the resource we have created and facilitate the community to use this annotation for future analyses.

We show a screenshot of a corner of this supplementary excel here:

To further address the reviewer’s comment, in the revised manuscript we added an analysis where we calculated the estimated frequencies that each full-stack state overlaps with each state in the 25-state concatenated model, for each group of cell types. The grouping of cell types/biosamples were based on the metadata provided by Roadmap Epigenomics (<https://egg2.wustl.edu/roadmap/web_portal/meta.html>). In the **Supp. Fig. 9** shown below, each row corresponds to a combination of a per-cell-type state annotation and a cell group. Each column corresponds to a full-stack state. A cell shows the estimated frequency that at a genomic position annotated as the corresponding full-stack state (column), a biosample from the corresponding cell group will be annotated as the corresponding per-cell-type state (row). In the supplementary table accompanying this figure (**Supp. Data. File 3**), the rows and columns are flipped. This analysis serves to further address the reviewer’s comment as it indicates quantitatively how frequently each full-stack state overlaps with different per-cell type chromatin state annotations from different cell groups. This is now presented in **Supp. Fig. 9 and Supp. Data. File 3** (sheet ‘prob_in_25-state’)**:**

Cell groups (1st column)

25-state

**Full-stack states**

**Supplementary Figure 9: Estimated probabilities of per-cell-type chromatin states overlapping with full-stack states.** The figure shows estimated probabilities of per-cell-type chromatin state assignments overlapping with full-stack state annotations conditioned on the cell group of the per-cell-type annotations. This figure is also provided as an excel file in **Supp. Data File 3** where

**Per-cell-type 25-state in different cell groups**

**T s sA**

**P ro m U**

**P ro m D 1**

**P rom D 2**

**T x5 p**

**T x**

**T x3 p**

**T xW k**

**T xR eg**

**T xE n h5 p**

**T xE nh 3p**

**T xE n hW**

**E n h A 1**

**En h A 2**

**E n h A F**

**En h W 1**

**E n hW 2**

**E nh A c**

**D N as e**

**ZN F/R p ts**

**H et**

**P ro m P**

**P ro m Biv**

**R ep rP C**

**Q uies**

**Gap Ar tf1**

**Gap Ar tf2 Gap Ar tf3 Q uies1**

**Q uies2 Q uies3 Q uies4 Q uies5 HET1**

**HET2 HET3 HET4 HET5 HET6 HET7 HET8 HET9**

**Re prP C1 Re prP C2 Re prP C3 Re prP C4 Re prP C5 Re prP C6 Re prP C7 Re prP C8 Re prP C9 Acet 1**

**Acet 2 Acet 3 Acet 4 Acet 5 Acet 6 Acet 7 Acet 8**

**EnhW k1 EnhW k2 EnhW k3 EnhW k4 EnhW k5 EnhW k6 EnhW k7 EnhW k8 EnhA 1**

**EnhA 2**

**EnhA 3**

**EnhA 4**

**EnhA 5**

**EnhA 6**

**EnhA 7**

**EnhA 8**

**EnhA 9**

**EnhA 10**

**EnhA 11**

**EnhA 12**

**EnhA 13**

**EnhA 14**

**EnhA 15**

**EnhA 16**

**EnhA 17**

**EnhA 18**

**EnhA 19**

**EnhA 20**

**TxEnh1**

**TxEnh2**

**TxEnh3**

**TxEnh4**

**TxEnh5**

**TxEnh6**

**TxEnh7**

**TxEnh8 TxW k1 TxW k2 Tx1**

**Tx2 Tx3 Tx4 Tx5 Tx6 Tx7 Tx8 TxEx1**

**TxEx2**

**TxEx3**

**TxEx4 znf1 znf2**

**DN ase1 BivProm 1 BivProm 2 BivProm 3 BivProm 4 Prom F1 Prom F2 Prom F3 Prom F4 Prom F5 Prom F6 Prom F7 TSS1**

**TSS2**

**m in_prop**

**0 .00**

**m ax_prop	0 .72**

**0.00**

**0.67**

**0 .00**

**0 .79**

**0.00**

**0.47**

**0 .00**

**0 .81**

**0.00**

**0.73**

**0 .00**

**0 .95**

**0.00**

**0.67**

**0 .00**

**0 .50**

**0.00**

**0.36**

**0.00**

**0.31**

**0.00**

**0.49**

**0 .00**

**0 .41**

**0.00**

**0.32**

**0.00**

**0.31**

**0.00**

**0.26**

**0 .00**

**0 .42**

**0.00**

**0.22**

**0 .0 0**

**0 .5 7**

**0.00**

**0.69**

**0 .00**

**0 .81**

**0.00**

**0.26**

**0 .0 0**

**0 .7 2**

**0.00**

**0.95**

**0 .0 0**

**1 .0 0**

**Sta te**

| **Other** | **ENCODE2012** | **ES-deriv** | **Digestive** | **ESC** | **iPSC** | **Mesench** | **Heart** | **IMR90** | **Sm. Muscle** | **Muscle** | **Myosat** | **Epithelial** | **Adipose** | **Thymus** | **Brain** | **Neurosph** | **HSC & B-cell** | **Blood & T-cell** |
| --- | --- | --- | --- | --- | --- | --- | --- | --- | --- | --- | --- | --- | --- | --- | --- | --- | --- | --- |

| **Quies** | **ReprPC** | **Prom Biv** | **Prom P** | **H et** | **ZNF /Rpts** | **D N ase** | **EnhAc** | **EnhW 2** | **EnhW 1** | **EnhAF** | **EnhA2** | **EnhA1** | **Tx EnhW** | **Tx Enh3p** | **Tx Enh5p** | **Tx Reg** | **Tx W k** | **Tx 3p** | **Tx** | **Tx 5p** | **Prom D 2** | **Prom D 1** | **Prom U** | **TssA** |
| --- | --- | --- | --- | --- | --- | --- | --- | --- | --- | --- | --- | --- | --- | --- | --- | --- | --- | --- | --- | --- | --- | --- | --- | --- |

### it is accompanied with detailed comments about each full-stack state. The figure is based on a 25-state per-cell type chromatin state model (Ernst & Kellis, 2015), and 19-previously defined tissue groups for the 127 reference epigenomes (Kundaje et al., 2015). Each row corresponds to a combination of per-cell type state (among 25 states) and tissue group, as denoted in the first two columns and legend. Rows corresponding to the same per-cell-type state are grouped together. The first two columns show the colors of tissue groups and per-cell-type state, respectively, as indicated in legends on the right, and matching with the colors in **Supp. Fig. 8**, except we changed per-cell-type quiescent 25-state from white to blue for better visibility. The 100 following columns correspond to 100 full-stack states. Values in the heatmap correspond to the estimated probability a genomic position annotated as a full-stack state (column) is also annotated as a per-cell-type state in a cell type from the corresponding tissue group (row) (**Methods**). The last two columns show the minimum and maximum probabilities observed for each per-cell type state for any combination of tissue group and full-stack state. The heatmap colors correspond to the 25-state’s colors and are scaled such that the maximum probability values in each block are colored darkest (as seen in the right most column). The figure complements **Supp. Fig. 8** in providing information on how each full-stack state can correspond to different 25-per-cell type states in different groups of cells, hence stratifying full-stack states’ characteristics in more details. For example, full-stack state ReprPC8 shows high probabilities of overlapping ReprPC state in ESC-related cell groups (ESC, iPSC, and ES-derived), and quiescent state in other cell groups.

We also show a screenshot of the excel **Supplementary Data File 3** that accompanies this figure with detailed comments about full-stack states based on our observations from the results of this analysis. Researchers can gain insights into specific full-stack states of interest in their future applications through studying results in this **Supplementary Data File 3**.

We updated the section ‘Methods/ Enrichment and estimated probabilities of overlap with cell-type-specific ChromHMM annotations’ to report this analysis procedure, which now reads

###### “Enrichment and estimated probabilities of overlap with per-cell-type chromatin state annotations

We obtained per-cell type chromatin state annotations based on a 25-state ChromHMM model learned using concatenated approach for 127 reference epigenomes, which we will refer to as cell types for ease of presentation, from the Roadmap Epigenomics project [3,9]. This model was trained based on observed and imputed data for 12-marks. We hereafter refer to this model as the CT-25-state model. As per the design of the concatenated approach of ChromHMM, CT-25-state model generates per-cell-type chromatin state annotations for each of the 127 cell types, and the 25 states’ characteristics are shared across 127 cell types. For each of these 127 cell types, we calculated overlap enrichments between the 100 full- stack states and the CT-25-states, resulting in 127 tables of size 100-by-25. We summarized this information by reporting, for each of the 100 full-stack states, and 127 cell types, the per-cell-type state in CT-25-state model that is maximally enriched, resulting in a 100-by-127 table (**Supp. Fig. 8**). We also provided detailed comments about the patterns of maximum-enriched-states observed across 127 cell types for each full-stack

state in the **Supplementary Data File 3** to serve as a resource for future applications. We also reported, for each of the 100 full-stack states and each per-cell-type-25-state, the maximum and median values of fold enrichments across 127 cell types (**Supplementary Data File 3**).

In addition, we also estimated for each combination of (1) CT-25 state, (2) cell type group and (3) full-stack state, the probability that a genomic position being annotated as the corresponding full-stack state will overlap with the corresponding per-cell type state in a cell type from the corresponding cell group. The 19 groups of cell types were previously defined by the Roadmap Epigenomics Consortium [3]. To compute the target estimate probabilities, for each full-stack state, we sampled 100 genomic bins (each of length 200bp) that are assigned to that full-stack state. Second, in each of the 127 cell types, we report the frequency that the sampled regions of each full-stack state overlapped with each CT-25-state. We repeated such a process 21 times. We then calculated the average frequencies of overlap between each full-stack state and each CT-25-state, across 21 random samplings and across the cell types in each group (Example: Blood, ESC). This results in a table of size 100 full-stack states by 475 combinations of CT-25 states and 19 cell groups, with each cell showing the values of estimated probabilities (**Supp. Fig. 9**). These values, along with detailed comments about patterns of these overlap probabilities for each full-stack state, are available in **Supplementary Data File 3**.”

We revised a sentence ‘Results/ Major groups of full-stack states’ to better highlight the utility of the extensive state characterization available in the supplement, which now reads:

“A detailed characterization of all states in terms of associated chromatin marks, genomic elements and different associated per-cell-type chromatin states across cell groups can be found in **Supplementary Data File 1-3. W**e expect it will serve as a resource for future applications using the full-stack annotations.”

*In Fig1, the authors showed two loci of full-stack modeling annotations. It is indeed an advantage of the full-stack model to provide a big-picture view of the epigenomes, as illustrated by these two loci.*

*However, there are many genomic regions with frequent state transitions as shown by the concatenated approach; can the authors provide two examples of such regions and illustrate their state assignment in the full-stack model?*

We thank the reviewer for the suggestions. We note that we had a supplementary figure (now **Supplementary Fig. 24**) illustrating the per-cell type chromatin state annotations at these loci across cell types with frequent state transitions. We now directly reference this supplementary figure from the **Fig. 1** legend. We also note that we had a supplementary figure illustrating three different cell-type-specific enhancer associated loci (now **Supplementary Fig. 25**). In our revised manuscript, we added supplementary figures for two additional loci (**Supplementary Figs. 26-27**). One figure illustrates a region that is in a per-cell-type quiescent state in a number of cell types, but frequently transitions to a heterochromatin state in embryonic and iPSC cells. This region is largely assigned to the HET9 full-stack state. Another locus is largely assigned to full-stack state PromF5, and transitions between promoter flanking and bivalent promoter states across different cell types.

The sentence we added to the **Fig. 1** legend is:

“Per-cell-type concatenated model annotations for these loci from these and additional reference epigenomes can be found in **Supp. Fig. 24**.”

The four supplementary figures illustrating seven different loci are shown below, with the last two supplementary figures new to this revised manuscript.

**chr5:153,820,000– 153,830,000**

Full-stack	Full-stack

**blood**

**Brain**

**Digestive**

**ESC**

**ES-deriv**

**iPSC**

**Heart**

**Mu scle**

**Skin**

**Sm Mu scle**

**blood**

**Brain**

**Digestive**

**ESC**

**ES-deriv**

**iPSC**

**Heart**

**Mu scle**

**Skin**

**Sm Mu scle**

Full-stack state colors

| **Assembly Gaps**  **& artifacts** | **quiescent** | **HET** | **polycomb repressed** | **acetylations** | **weak**  **enhancers** | **enhancers** | **transcribed & enhancer** | **weak**  **transcription** | **transcription** | **exon &**  **transcription** | **ZNF genes** | **DNase** | **bivalent promoter** | **flanking promoter** | **TSS** |
| --- | --- | --- | --- | --- | --- | --- | --- | --- | --- | --- | --- | --- | --- | --- | --- |

Per-cell-type state colors

| **Quiescent** | **Het** | **Wk ReprPC** | **ReprPC** | **Bivalent Enh** | **W k Enh** | **Active Enh** | **Genic Enh** | **Transcription** | **ZNF/Rpts** | **Poised TSS** | **Flk TSS** | **Active TSS** |
| --- | --- | --- | --- | --- | --- | --- | --- | --- | --- | --- | --- | --- |

**chr5:156,015,000 – 156,020,000**

### **Supplementary Figure 24: Illustration of the full-stack annotations.** Two loci representing regions that are in transcribed and active promoter states across cell types (left), and in an enhancer state specifically in brain (right). The loci correspond to those presented in **Fig. 1**. The top track shows the full-stack state annotations. The following tracks show per-cell-type- concatenated annotations from 18-state models based on imputed data (Kundaje et al., 2015). The cell types are ordered based on their associated cell groups. A color legend for the states is shown along the bottom.

##### A

**Full-stack state colors**

**blood**

**Brain**

**Digestive**

**ESC**

**ES-deriv**

**iPSC**

**Heart**

**M u scle**

**Skin**

**Sm M uscle**

**Assem bly Gaps &**

**artifacts**

**quiescent**

**HET**

**polycomb**

**repressed**

**acetylations**

**weak**

**enhancers**

**enhancers**

**transcribed &**

**enhancer**

**weak**

**transcription**

**transcription**

**exon &**

**transcription**

**ZNF genes**

**DNase**

**bivalent**

**prom oter**

**flanking**

**prom oter**

**TSS**

##### B

**blood**

**Brain**

**Digestive**

**ESC**

**ES-deriv**

**iPSC**

**Heart**

**Muscle**

**S kin**

**Sm Muscle**

**Per-cell-type state colors**

**Quiescent**

**Het**

**Wk ReprPC**

**ReprPC**

**Bivalent Enh**

**W k Enh**

**Active Enh**

**Genic Enh**

**Transcription**

**ZNF/Rpts**

**Poised TSS**

**Flk TSS**

**Active TSS**

C

**blood**

**Brain**

**Digestive**

**ESC**

**ES-deriv**

**iPSC**

**Heart**

**M uscle**

**Skin**

**Sm M uscle**

### **Supplementary Figure 25: Illustration of full-stack cell-type-specific enhancer states. (A-C)** The first track in each panel demonstrates the full-stack state annotation. Each of the following tracks show chromatin state annotations from a 18-state per-cell-type-concatenated model (Kundaje et al., 2015). The individual reference epigenomes IDs and their tissue groups are labeled on left. The chromatin state coloring is labeled on right. **(A)** A genomic region (chr5:157340200- 157342000) annotated to an active enhancer state in digestive cells in the full-stack model (EnhA14). **(B)** A genomic region (chr5:156679900-156686500) annotated to blood enhancer

states in the full-stack model (EnhWk6 and EnhA8). **(C)** A genomic region (chr5:156227000- 156231000) annotated as an ESC/iPSC-specific enhancer state in the full-stack model (state EnhA18).

#### Full- stack states

**blood**

**Brain**

**Digestive**

**Epithelial**

**ESC**

**Heart**

**iPSC**

**M esench**

**M uscle**

**M yosat**

**Ne urosph**

**Skin**

**Thymus**

**Demonstration of the state transitions for full-stack state HET9**

**Full-stack state colors**

**Assembly Gaps &**

**artifacts**

**quiescent**

**HET**

**polycomb**

**repressed**

**acetylations**

**weak**

**enhancers**

**enhancers**

**transcribed &**

**enhancer**

**weak**

**transcription**

**transcription**

**exon &**

**transcription**

**ZNF genes**

**DNase**

**bivalent**

**promoter**

**flanking**

**promoter**

**TSS**

**Per-cell-type state colors**

**Supplementary Figure 26: Illustration of full-stack heterochromatin state HET9.** The figure captures the per-cell-type-concatenated chromatin state maps for various reference epigenomes, and the corresponding full-stack chromatin state maps at region chr17:21772099-

**TssA**

**Prom U**

**Prom D 1**

**Prom D 2**

**Tx5p**

**Tx**

**Tx3p**

**TxWk**

**Tx Reg**

**Tx Enh5p**

**Tx Enh3p**

**Tx EnhW**

**EnhA1**

**EnhA2**

**EnhAF**

**EnhW 1**

**EnhW 2**

**EnhAc**

**DNase**

**ZNF/Rpts**

**Het**

**Prom P**

**Prom Biv**

**ReprPC**

**Quies**

### 21791900. The first 66 tracks show chromatin state annotations from a 25-state concatenated model for 66 reference epigenomes (equivalently, in this paper, cell types) (Ernst & Kellis, 2015). The individual reference epigenomes IDs and their tissue groups are labeled on the left. The chromatin state colors are explained on the right. The last track, shown in full mode to display all state labels on the right, corresponds to the full-stack chromatin state map at this region. State HET9 is characterized, based on our analysis, as an ESC-group-related heterochromatin state (**Fig. 2C, Supp. Fig. 8-9, Supplementary Data 1,3**). Detailed characterizations of all full-stack states are in **Supplementary Data 1-3**.

#### Demonstration of the state transitions for full-stack state PromF5

**Full-stack state colors**

**blood**

**Brain**

**Digestive**

**Epithelial**

**ESC**

**Heart**

**iPSC**

**Me sench**

**M uscle**

**M yosat**

**Neurosph**

**Skin**

**Thymus**

**Assembly Gaps &**

**artifacts**

**quiescent**

**HET**

**polycomb**

**repressed**

**acetylations**

**weak**

**enhancers**

**enhancers**

**transcribed &**

**enhancer**

**weak**

**transcription**

**transcription**

**exon &**

**transcription**

**ZNF genes**

**DNase**

**bivalent**

**promoter**

**flanking**

**promoter**

**TSS**

**TssA**

**Prom U**

**Prom D 1**

**Prom D 2**

**Tx 5p**

**Tx**

**Tx 3p**

**Tx Wk**

**TxReg**

**Tx Enh5p**

**Tx Enh3p**

**Tx EnhW**

**EnhA1**

**EnhA2**

**EnhAF**

**EnhW 1**

**EnhW 2**

**EnhAc**

**DNase**

**ZNF/Rpts**

**Het**

**Prom P**

**Prom Biv**

**ReprPC**

**Quies**

**Full-stack**

**Per-cell-type state colors**

### **Supplementary Figure 27: Illustration of full-stack flanking promoter state PromF5.** The figure captures the per-cell-type-concatenated chromatin state maps for various reference epigenomes, and the corresponding full-stack chromatin state maps at region chr10:33621649- 33627350. The first 66 tracks show chromatin state annotations from a 25-state concatenated model for 66 reference epigenomes (equivalently, in this paper, cell types) (Ernst & Kellis, 2015). The individual reference epigenomes IDs and their tissue groups are labeled on the left. The chromatin state coloring is labeled on the right. The last track, shown in full mode to display all state labels on the left, corresponds to the full-stack chromatin state map at this region. State PromF5 is characterized, based on our various analyses as state frequently found at flanking promoter regions with some upstream bias, and sometimes, this state overlaps with regions of bivalent promoters in Blood-related and ESC-related groups (Blood & T cells, HSC & B cells, ESC, iPSC and ES-deriv) (**Supp. Fig. 8-9, Supplementary Data 1,3**). Detailed characterizations of all full- stack states are in **Supplementary Data 1-3**.

We updated the main paper section “Results/ *Stacked Model Differentiates Cell-Type-Specific from Constitutive Activity*” in response to this comment, including stating:

“While the major groups of states outlined above can correspond to states from models producing per-cell- type annotations [3,4], the full-stack states provide additional information. For example, the states can differentiate cell-type-specific from constitutive activities. This cell type specificity in the full-stack states is reflected in the emission parameters of cell types from different tissue groups **(Fig. 2B,C, Supp. Data. File 1)** and the overlap of per-cell-type chromatin state annotations from different cell types [9] **(Supp. Figure 8-9, Supp. Data 3)**.”

and

“Additionally, state HET9 showed strong association as heterochromatin in ESC/iPSC groups, while being mostly quiescent in other cell types (**Fig. 2C, Supp. Fig. 26, Supplementary Data File 1,3**). State PromF5 is associated with putative bivalent promoter chromatin states in some blood and ESC-related cell types, but with flanking promoter states in most other cell types (**Supp. Fig. 27, Supplementary Data 1,3**).

*Are different TSS states (TSS1-2, BivProm1-4) in the full-stack model associated with different classes of genes? Can the author perform GO enrichment for each subset of genes?*

We thank the reviewer for this suggestion. We added a supplementary figure to address this question, as shown below. We provide the list of top GO terms, associated FDR-corrected p-values for all full-stack states in **Supplementary Data File 1**.

**Top gene ontology enrichment terms for bivalent promoter states**

**Top gene ontology enrichment terms for TSS states**

**Top gene ontology enrichment terms for flanking promoter states**

**Supplementary Figure 13: Top GO terms for states in promoter-associated states.** In each subpanel, each column corresponds to a full-stack state, each row corresponds to a GO term, and each cells show the negative log of FDR-corrected p values of enrichments for states and GO terms. Only terms that are a top enriched GO terms with at least one full-stack states are shown.

### For each full-stack state, only the associated top GO term’s negative log q-values are shown and colored according to the color scales on the right. The most enriched GO-terms for all full-stack states are provided in **Supplementary Data File 1**.

We added two sentences in section ‘Results/Major groups of states’ on this analysis and refer readers to our supplementary data files for detailed characterization of states with GO terms.

“States in promoter-associated groups (TSS, PromF, BivProm), along with those in other groups, show various enriched gene-ontology (GO) terms based on genes overlapping or proximal to each state (**Supp. Fig. 13, Supp. Data File 1, Methods**). For example, among biological process terms, BivProm1 is most enriched for ‘embryonic organ morphogenesis’ genes, while TSS1 is most enriched for ‘nucleic acid metabolic process’, consistent with the bivalent [15] and the constitutively active nature of the two states, respectively.”

We added the Methods section/ “Gene ontology enrichments with full-stack states” to describe this analysis, which now reads:

We calculated the gene ontology enrichments of genes being in proximity to each full-stack state annotation using GREAT [16]. For each full-stack state, we reported the top-GO terms with lowest FDR-corrected p- values. If multiple GO terms showed the same minimum p-values, we reported all of them. A full list of GO-terms associated with full-stack states are available in **Supplementary Data File 1**.

*The paper provides many numbers for enrichment, but very often, it is unclear whether the enrichment has any biological significance. For example:*

We thank the reviewer for the feedback. We have revised the manuscript to place greater emphasis on the biological significance of the enrichments with various genomic contexts. We present our response to reviewer’s particular comments below each comment.

Additionally, after addressing each of reviewer’s questions about the biological significance of our enrichment results, we present a supplementary figure that we extended include sex chromosome enrichments and three additional supplementary figures related to our analysis of full-stack states’ average DNA methylation levels and the states’ enrichment with different families of polycomb-repressed protein complexes (subunits BMI1, RING1 and RNF2 in PRC1, and EZH2, SUZ12, EED in PRC2). We have found that these analyses are helpful in supporting the biological significance of distinctions between a number of related states, including in some of our own ongoing applications of the full-stack annotations. We expect the results from these analyses will also be helpful for other researchers as well.

More generally, we expect the biological significance of states will continue to be elucidated as they are used by other researchers for various applications. To make that point we now cite three pre-prints now available by us and/or our collaborators making use of the state annotations [11–13].

“We expect many applications of the full-stack annotations and they have already begun to be applied in other work [11–13], which we expect to further elucidate the biological significance of different states.”

*Line 130-131: Acetylation is an informative histone modification. For those regions assigned as acetylation groups, do you have any biological explanation? What's their role in gene regulation?*

We thank the reviewer for the questions. These states are primarily associated with the large number of different acetylations whose mapping was limited to IMR90 or six ESC derived cells. Two acetylation marks H3K27ac and H3K9ac, associated with active enhancers and promoters, respectively, were mapped in a diverse set of cell or tissue types. Some of the positions in these states may correspond to regulatory elements that are relatively specific to IMR90 or the six ESC-derived cell types. However, a number of positions in these states also correspond to positions marked by less commonly mapped acetylations such as H4K91ac, H2BK12ac or H3K18ac (**Supplementary Data 1**), without strong signal for H3K27ac, H3K9ac, or H3K4me1/2/3 (**Fig. 2A**).

Genes proximal to these states have lower average expression than a number of enhancer and promoter states and the sequence overlapping the states is less conserved, suggesting an overall less significant role in gene regulation than those enhancer and promoter states (**Fig. 3A,C**). On the other hand, genes proximal to these states have an overall higher expression than states from the ReprPC and HET groups, thus not suggesting a clear repressive role for these states either and possibly suggesting a weaker activating role. We think that further experiments to investigate the effects of different acetylation marks may be required to fully understand their role in gene regulation.

Based on this comment we have now revised the Results section ‘Major groups of full-stack states’ to now state

“There is an acetylations group marked primarily by high emission of various acetylation marks profiled only in IMR90 or ESC derived cells, while having weaker signals for enhancer or promoter associated marks such as H3K4me1/2/3, H3K27ac, and H3K9ac (Acet1-8).”

and also state in the following paragraph:

“States in the Acet group had a lower average expression of proximal genes compared to states in the enhancer and promoter groups (**Fig.3C**) while higher compared to ReprPC and HET groups.”

*Line 138-139: "A DNase group consists of one state (DNase1) with strong emission of only DNase in all profiled cell types." What's the biological function of the DNase group since other active states are also expected to have high DNase signals?*

We thank the reviewer for the question. The DNase1 state is distinguished from other active states that have DNase signals in that it has a limited presence of histone modifications. This state corresponds to locations of CTCF binding with limited histone modifications previously described as candidate insulator regions in per cell type concatenated chromatin state annotations [17]. CTCF elements have been associated with a variety of roles including acting as an enhancer-blocking agent and being a boundary element of topologically associated domains (TADs) [18–20]. A recent study showed that cell-type-stable TAD boundaries are even more enriched for CTCF-binding [21]. The DNase1 can thus inform us of regions typically associated with “stable” (shared among multiple cell types) CTCF binding regions and their implicated biological functions.

We have updated out text on this result in the section ‘Results/ *Major groups of full-stack states*’ to now state:

“The DNase-specific state DNase1, showed distinct enrichment for CTCF-specific per-cell-type chromatin states defined in six cell types compared to other full-stack states [17] (**Fig. 3F, Supp. Fig. 14**). These CTCF-specific states have previously been suggested to be a candidate insulators and may have other roles that CTCF has been implicated in such as demarcating TAD boundaries [18–21]. The DNase1 state may correspond to similar roles, particularly where the CTCF-binding is relatively stable across cell types.”

*In Fig 3A, Why are the TSS enrichments in PromF4 and PromF5 so high (62.2 and 31.9, respectively), even higher than BivProm1-4 (enrichment 8-28)? Would these two PromF states actually be a weak TSS state instead of flanking? Or are they actually located in gene-dense regions with many TSSs?*

We thank the reviewer for these questions. States assigned to the bivalent promoter states (BivProm) had a balanced presence of H3K27me3 and H3K4me3, while states given the PromF and TSS labels had more limited levels of H3K27me3 relative to H3K4me3. The ‘Prom’ portion of the BivProm state labels is meant to imply enrichment for a broad promoter region, such as +/-2kb TSS, without differentiating between enrichment right at the TSS and flanking-TSS regions. BivProm states show 7.0-19.3 fold enrichment with

+/-2kb TSS regions, while PromF states show 22.4-28 fold enrichment. States given the ‘PromF’ label enrichment did not peak right at the transcription start site and instead at varying positions flanking it (as shown in **Fig. 3E** and **Supp. Fig. 12**). The enrichment of states given the TSS label peaked right at the TSS and also had a substantially higher fold enrichment than any other state.

While we could see an argument for giving states PromF4-5 a ‘TSS’ state label due to their high enrichment with TSS, which likely cannot simply be explained by being located in gene-dense regions with many TSS, we think the PromF label is more appropriate since these states showed even higher enrichments in the flanking regions of TSS (**Supp. Fig. 12**). Additionally, in the interest of keeping the number of state groups manageable, our preference is not to subdivide the PromF group, and rather convey details about individual states through our descriptions of them.

In terms of a ‘weak TSS’ label specifically, while this label may convey some association with a TSS, we feel such a label would not convey the even stronger enrichments for regions surrounding the TSS, and ‘weak’ could be misinterpreted in terms of correlation with gene expression.

We modified one sentence in the section ‘Results/Major groups of full-stack states’ to now state

“The states in the transcription start site group (TSS1-2) had enrichment values that peaked at the TSS (>= 100 fold enrichment) (**Fig. 3A, E**).”

We also updated the **Supplementary Data 1** to clarify this point.

*Line 228-229: "Acet1, a state associated with various acetylation marks and H3K9me3 had a 23-fold enrichment for simple repeats (Supp. Fig. 20)." What is the biological implication of this enrichment?*

We thank the reviewer for the question. This means that there is a combinatorial pattern of the 1032 data sets with signals where simple repeats are present in the genome. This enrichment appears to be in large part due to (CA)n and (TG)n repeats, which have 72 and 76 fold enrichment for this state respectively and comprise 74% of all simple repeats in the state. We now report their enrichments separately. Given that these repeats are known to be highly polymorphic within humans [22], we suspect the signal is related to

technical issues due to this variability from the reference genome. In general, states that might capture technical artifacts are still valuable, since it implies a greater portion of the signal captured in the remaining states would correspond to biological signals.

In response to this comment we have revised the manuscript to now state:

“For example, Acet1, a state associated with various acetylation marks and H3K9me3 had a 23-fold enrichment for simple repeats largely driven by (CA)n and (TG)n repeats which were 72 and 76 fold enriched and comprised 74% of all simple repeats in this state (**Supp. Fig. 28**). As (CA)n and (TG)n repeats are known to be highly polymorphic in humans [22], this suggests the possibility that signal detected in these regions may in part be due to technical issues related to deviations from the reference genome.”

The updated supplementary figure is presented below our response to the next question.

*Line 231-232: "States in the transcription start site group, TSS1-2 were most strongly enriched with tRNA and low complexity repeat class (~10-60 fold) (Supp. Fig. 20)." What does this mean biologically?*

We thank the reviewer for the question. The high enrichment of these transcription start site states for the tRNA repeat class is consistent with tRNAs being short genes [23] and thus the entire gene is near its promoter. The high enrichment for low complexity regions is consistent with the extreme GC content associated with these states. We now report the fold enrichments for G rich, C rich, and GC rich low complexity repeats individually. Notably the TSS1 and TSS2 had 195-303 fold enrichment for GC rich low complexity repeats. We also directly show these states are GC rich by separately computing the enrichment for 5bp windows that had 1, 2, 3, 4, or 5 G or C nucleotides as available from the UCSC genome browser [24]. Windows in which all five nucleotides were a G or C had a 19-20 fold enrichment in the TSS1-2 state.

We have the revised the text to now state:

“States in the transcription start site group, TSS1-2, were most strongly enriched with low complexity repeat class (10.5-18 fold) and most notably GC rich repeats (195-303 fold), consistent with these states being most enriched for windows of high GC content (**Supp. Fig. 28**). Moreover, the TSS1-2 states are also most enriched with tRNA class (50-61 fold) (**Supp. Fig. 28**), consistent with tRNAs being short genes [23].”

The updated **Supp. Fig. 28** as follows:

# A

**En richm ents w ith different classes o f repeats**

**state**

**Genome %**

**DNA**

**LINE?**

**L INE**

**LTR?**

**LTR**

**Other**

**SINE?**

**SINE**

**Satellite**

**Unknown?**

**Unknown**

**sc RNA**

**C-rich**

**Lo w-comp lexity rep eat en rich ed in states with h igh GC co n ten t**

**Genome %**

**GC_content_1 per5 bp**

**GC_content_2 per5 bp**

**GC_content_3 per5 bp**

**GC_content_4 per5 bp**

**GC_content_5 per5 bp**

**Low_complexity repeats**

B

**Simp le rep eats en rich ed in Acet1**

**Genome %**

**Simple_repeat**

C

**GapArtf1**

11.9 0.2	0.2

**DNA?**

0.0

0.9

0.0	0.3

2.4

0.2

**RC**

0.1

**RNA**

0.3

0.2	1.3	0.1	0.1	0.3	0.2

**rRNA**

0.4	0.4

**snRNA**

**srpRNA**

0.6

**GapArtf1**	11.9	0.43	0.4	0.36	0.32	0.32	0.37	0.45	0.13	0.4

**GapArtf2**	0.05	1.07	1.57	0.88	0.56	0.51	0.98	0.27	1.25	0.5

**GapArtf3**	0.01	0.83	1.43	1.09	1.03	1.58	7.47	4.68	5.35	3.7

**Quies 1**	9.88	1.44	1.09	0.75	0.51	0.36	0.28	0.28	0.01	1.9

**Quies 2**	3.07	1.02	1.18	1.17	1.01	0.72	0.55	0.59	0.01	0.5

**Quies 3**	12.2	1.25	1.03	0.9	0.85	0.81	0.99	0.87	0.05	1.9

**Quies 4**	4.45	1.31	1.13	0.87	0.67	0.55	0.35	0.5	0.04	0.8

**Quies 5**	1.69	1.26	1.11	0.92	0.75	0.59	0.58	0.63	0.08	1.0

**HET1**	0.71	0.62	1.1	1.55	1.67	1.32	0.49	0.64	0.06	0.2

**HET2**	0.69	0.49	0.96	1.63	2.24	2.46	2.13	2.51	0.27	0.6

**HET3**	1.36	1.35	1.22	0.87	0.52	0.25	0.18	0.17	0	0.3

**HET4**	0.56	1.29	1.27	0.91	0.57	0.29	0.05	0.05	0	0.3

**HET5**	0.25	0.71	1.1	1.47	1.58	1.3	1.4	0.15	0.02	0.2

**HET6**	0.58	0.56	0.99	1.58	2.03	2.06	2.49	1.36	0.14	0.4

**HET7**	1.02	0.68	1.02	1.45	1.78	1.88	1.69	1.76	0.14	0.6

**HET8**	0.43	0.74	1.13	1.43	1.45	1.14	0.79	0.66	0.16	0.4

**HET9**	1	1.18	1.17	1.02	0.82	0.55	0.26	0.29	0.02	0.4

**ReprPC1**	0.19	0.6	0.95	1.48	2.18	3.2	3.3	3.83	2.18	1.3

**ReprPC2**	0.32	0.94	1.11	1.23	1.26	1.2	1.81	2.84	0.9	1.4

**ReprPC3**	1.11	1.03	1.19	1.16	0.98	0.65	0.68	0.74	0.02	0.7

**ReprPC4**	3.93	1.18	1.13	1.01	0.87	0.64	0.74	0.76	0.03	0.9

**ReprPC5**	0.63	0.45	0.94	1.67	2.32	2.45	2.33	2.07	0.28	0.7

**ReprPC6**	1.51	0.61	1.02	1.53	1.92	1.93	1.99	1.68	0.16	0.6

**ReprPC7**	0.61	0.32	0.81	1.73	2.91	3.66	2.57	1.96	0.31	0.5

**ReprPC8**	0.48	0.85	1.15	1.33	1.28	0.97	2.49	2.05	0.02	1.2

**ReprPC9**	0.37	1.06	1.11	1.12	1.06	0.95	1.52	1.74	0.57	1.3

**Acet1**	0.18	0.4	1.03	1.77	2.01	1.87	3.71	4.24	0.14	0.8

**Acet2**	0.85	0.65	1.13	1.53	1.57	1.12	1.4	1.3	0.01	0.4

**Acet3**	2.65	0.86	1.1	1.3	1.35	1.08	1.31	1.14	0.05	0.6

**Acet4**	0.4	0.59	1.06	1.56	1.83	1.53	1.51	1.52	0.02	0.5

**Acet5**	0.86	0.98	1.15	1.2	1.1	0.76	1.12	1.12	0	0.9

**Acet6**	0.43	1.01	1.17	1.18	1.04	0.72	0.62	0.54	0	0.7

**Acet7**	0.28	0.61	1.04	1.53	1.88	1.76	1.25	1.34	0.04	0.4

**Acet8**	0.56	0.65	1.03	1.5	1.84	1.74	1.32	1.14	0.08	0.4

**EnhW k 1**	1.54	1.31	1.13	0.88	0.65	0.44	0.41	0.47	0.01	1.1

**EnhW k 2**	0.35	0.43	0.89	1.65	2.55	3.02	1.39	1.58	0.22	0.4

**EnhW k 3**	0.83	0.88	1.1	1.29	1.35	1.2	1.1	1.11	0.23	0.6

**EnhW k 4**	2.22	1.24	1.14	0.95	0.73	0.53	0.69	0.62	0.05	0.9

**EnhW k 5**	0.99	1.13	1.09	1.04	0.98	0.84	1.36	1.26	0.05	1.3

**EnhW k 6**	0.59	1.06	1.1	1.11	1.08	0.91	1.09	0.72	0.04	0.9

**EnhW k 7**	0.48	1.08	1.1	1.09	1.05	0.87	1.05	1.33	0.16	1.0

**EnhW k 8**	1.37	1.25	1.14	0.95	0.75	0.58	0.8	0.83	0.01	0.9

**EnhA1**	0.18	0.51	0.94	1.59	2.32	2.69	1.61	1.51	0.1	0.5

**EnhA2**	0.33	0.91	1.11	1.26	1.31	1.22	1.04	1.04	0.15	0.6

**EnhA3**	0.19	1.06	1.17	1.12	0.98	0.81	0.31	0.9	0.03	0.6

**EnhA4**	0.3	1.14	1.19	1.05	0.83	0.57	0.6	0.31	0	0.6

**EnhA5**	0.71	1.25	1.16	0.94	0.69	0.46	0.32	0.37	0.06	0.8

**EnhA6**	0.56	0.94	1.11	1.24	1.27	1.14	1.14	0.99	0.09	0.6

**EnhA7**	0.39	0.54	0.99	1.58	2.1	2.22	2.05	2.67	0.21	0.7

**EnhA8**	0.25	0.77	1.04	1.37	1.66	1.81	1.96	1.15	0.31	0.7

**EnhA9**	0.16	0.63	1	1.48	1.99	2.19	1.34	1.4	0.08	0.4

**EnhA10**	0.39	1.01	1.11	1.15	1.14	1.1	1.4	1.31	0.57	0.9

**EnhA11**	0.72	1.03	1.08	1.14	1.15	1	0.71	1.25	0.04	1.0

**EnhA12**	0.33	0.67	1.06	1.48	1.76	1.66	0.84	1.02	0.06	0.4

**EnhA13**	0.76	1.13	1.14	1.06	0.9	0.66	0.52	0.38	0.03	0.8

**EnhA14**	0.37	0.75	1.05	1.4	1.67	1.66	1.35	1.4	0.18	0.5

**EnhA15**	1.02	1.09	1.1	1.09	1.03	0.84	0.83	0.78	0.05	0.8

**EnhA16**	0.65	1.13	1.1	1.05	0.99	1.08	4.14	4.43	1.32	1.5

**EnhA17**	0.53	1.11	1.16	1.08	0.93	0.76	1.16	1.07	0.33	0.7

**EnhA18**	0.46	0.84	1.11	1.33	1.41	1.27	1.63	1.44	0.3	0.6

**EnhA19**	0.26	0.66	1.03	1.49	1.86	1.89	2.67	2.24	0.18	0.7

**EnhA20**	0.35	1.32	1.13	0.87	0.64	0.44	0.36	0.5	0	1.2

**Tx Enh1**	0.39	1.26	1.15	0.94	0.7	0.49	0.23	0.63	0.05	0.7

**Tx Enh2**	0.39	1.11	1.14	1.07	0.96	0.77	0.33	0.31	0.03	0.4

**Tx Enh3**	0.25	0.66	1.03	1.49	1.81	1.71	0.35	0.3	0.09	0.2

**Tx Enh4**	0.27	0.46	0.94	1.65	2.34	2.7	0.8	1	0.21	0.3

**Tx Enh5**	0.5	0.46	0.91	1.63	2.42	2.98	1.4	1.36	0.55	0.4

**Tx Enh6**	0.19	0.69	1.03	1.45	1.81	1.94	1.14	1.2	0.29	0.4

**Tx Enh7**	0.27	0.55	0.99	1.58	2.11	2.22	0.59	1.06	0.1	0.2

**Tx Enh8**	0.24	0.82	1.11	1.34	1.43	1.29	0.45	0.32	0.04	0.3

**Tx W k 1**	2.8	1.16	1.04	1	1	0.92	0.55	0.57	0.11	1.3

**Tx W k 2**	0.84	0.58	1	1.54	2.03	2.26	1.97	1.57	0.51	0.5

**Tx 1**	0.82	1.22	1.09	0.96	0.86	0.72	0.27	0.4	0.05	0.7

**Tx 2**	1.58	1.18	1.05	0.98	0.94	0.83	0.64	0.51	0.03	1.2

**Tx 3**	0.51	0.68	1.05	1.47	1.71	1.51	1.25	1.28	0.07	0.4

**Tx 4**	0.47	1.15	1.13	1.04	0.92	0.7	0.28	0.26	0.04	0.4

**Tx 5**	0.94	0.73	0.96	1.4	1.8	1.79	0.84	0.77	0.27	0.6

**Tx 6**	1.11	1.45	1.13	0.75	0.46	0.28	0.07	0.08	0.01	1.0

**Tx 7**	0.82	1.28	1.19	0.93	0.64	0.4	0.13	0.17	0	0.4

**Tx 8**	0.68	1.11	1.15	1.07	0.92	0.71	0.33	0.54	0.06	0.5

**Tx Ex 1**	0.27	0.89	1.14	1.28	1.28	1.09	0.42	0.26	0.04	0.2

**Tx Ex 2**	0.56	0.68	1.06	1.47	1.74	1.72	0.29	0.37	0.05	0.2

**Tx Ex 3**	0.66	0.55	0.93	1.55	2.21	2.63	0.99	1.09	0.42	0.3

**Tx Ex 4**	0.1	0.61	1.01	1.52	1.97	2.11	0.47	0.34	0.27	0.2

**znf1**	0.41	1.05	1.13	1.13	1.07	0.87	0.44	0.47	0.09	0.3

**znf2**	0.15	0.88	1.13	1.31	1.28	1.04	0.23	0.36	0.17	0.1

**DNase1**	0.2	0.87	1.02	1.22	1.54	2.86	12	11.7	10.6	2.6

**Biv Prom 1**	0.15	0.32	0.64	1.43	3.47	11.1	6.52	7.8	38.7	2.8

**Biv Prom 2**	0.16	0.39	0.72	1.46	3.13	8.58	8.16	8.38	25.9	2.7

**Biv Prom 3**	0.29	0.53	0.9	1.52	2.4	4.03	7.66	8.17	14.5	2.1

**Biv Prom 4**	0.13	0.93	1.05	1.2	1.44	2.06	2.65	3.77	3.16	1.6

**Prom F 1**	0.2	0.46	0.88	1.6	2.54	3.43	3.11	2.91	0.89	0.7

**Prom F 2**	0.14	0.59	0.94	1.48	2.22	3.12	2.15	2.27	0.96	0.6

**Prom F 3**	0.15	0.57	0.89	1.44	2.44	4.88	2.53	1.65	4.2	0.7

**Prom F 4**	0.19	0.32	0.62	1.38	3.5	12.8	7.04	6.35	48.7	2.6

**Prom F 5**	0.14	0.37	0.67	1.4	3.33	10.5	9.72	8.16	38.4	3.0

**Prom F 6**	0.13	1	1.09	1.15	1.24	1.58	0.6	0.66	0.5	0.5

**Prom F 7**	0.16	1.15	1.09	1.01	0.97	1.05	0.52	0.26	0.05	0.7

**TSS 1**	0.12	0.3	0.52	1.2	3.71	18.9	29.2	29.6	195	10.5

**TSS 2**	0.11	0.35	0.53	1.13	3.49	20.3	59.6	68.6	303	18.4

100	21.9	29.9	23	8.49	0.89 0.03 0.03 0.02	0.54

**(CA)n	(TG)n**

**GapArtf1**	11.9 0.24	0.23	0.6

**GapArtf2**	0.05 0.48	0.38	3.4

**GapArtf3**	0.01 2.54	1.79	6.5

**Quies1**	9.88 0.79	0.8	1.5

**Quies2**	3.07 1.17	1.15	0.7

**Quies3**	12.2	0.7	0.68	1.8

**Quies4**	4.45 0.53	0.56	0.9

**Quies5**	1.69 0.87	0.85	1.2

**HET1**	0.71 2.39	2.22	1.1

**HET2**	0.69 2.08	1.83	1.0

**HET3**	1.36 0.46	0.42	0.4

**HET4**	0.56 0.31	0.31	0.4

**HET5**	0.25 0.78	1.02	0.7

**HET6**	0.58 0.96	0.93	0.8

**HET7**	1.02 0.59	0.73	0.9

**HET8**	0.43 0.91	0.96	0.9

**HET9**	1	0.67	0.61	0.7

**ReprPC1**	0.19 2.77	2.77	1.3

**ReprPC2**	0.32 1.26	1.12	1.1

**ReprPC3**	1.11 0.72	0.71	0.7

**ReprPC4**	3.93 0.68	0.69	0.8

**ReprPC5**	0.63 2.04	2.12	1.0

**ReprPC6**	1.51 0.83	1.05	0.8

**ReprPC7**	0.61 1.41	1.59	0.7

**ReprPC8**	0.48 1.54	1.35	1.2

**ReprPC9**	0.37 0.92	0.81	1.1

**Acet1**	0.18 72.2	75.6	23.2

**Acet2**	0.85 4.26	4.44	1.7

**Acet3**	2.65 1.56	1.47	1.0

**Acet4**	0.4 2.98	3.57	1.1

**Acet5**	0.86 1.12	1.1	0.8

**Acet6**	0.43 1.24	0.9	0.6

**Acet7**	0.28 2.36	2.15	0.8

**Acet8**	0.56 1.09	0.96	0.5

**EnhW k1**	1.54	0.77	0.78	0.8

**EnhW k2**	0.35	1.64	1.65	0.7

**EnhW k3**	0.83	1.12	1	0.6

**EnhW k4**	2.22	1.07	1.06	0.8

**EnhW k5**	0.99	0.85	0.85	1.0

**EnhW k6**	0.59	0.82	0.88	0.8

**EnhW k7**	0.48	0.73	0.88	0.7

**EnhW k8**	1.37	0.63	0.63	0.6

**EnhA1**	0.18 2.03	2.18	0.8

**EnhA2**	0.33	1.1	1.2	0.6

**EnhA3**	0.19 0.85	0.9	0.5

**EnhA4**	0.3 0.69	0.82	0.4

**EnhA5**	0.71 0.75	0.77	0.6

**EnhA6**	0.56 1.13	1.1	0.7

**EnhA7**	0.39 2.32	2.42	1.0

**EnhA8**	0.25 1.69	1.72	0.8

**EnhA9**	0.16 1.77	1.81	0.7

**EnhA10**	0.39 0.82	0.93	0.7

**EnhA11**	0.72 0.73	0.72	0.8

**EnhA12**	0.33 1.34	1.24	0.6

**EnhA13**	0.76 0.76	0.8	0.7

**EnhA14**	0.37 1.08	1.07	0.5

**EnhA15**	1.02 0.81	0.84	0.7

**EnhA16**	0.65 0.85	0.7	0.9

**EnhA17**	0.53 0.81	0.73	0.5

**EnhA18**	0.46 1.15	1.24	0.5

**EnhA19**	0.26 2.26	2.52	0.8

**EnhA20**	0.35 0.78	0.76	0.7

**Tx Enh1**	0.39	0.75	0.68	0.5

**Tx Enh2**	0.39	0.55	0.61	0.4

**Tx Enh3**	0.25	1.3	2.04	0.7

**Tx Enh4**	0.27	3.12	2.59	1.0

**Tx Enh5**	0.5	2	1.65	0.8

**Tx Enh6**	0.19	1.26	1.15	0.6

**Tx Enh7**	0.27	2.57	2.69	0.9

**Tx Enh8**	0.24	0.89	0.77	0.4

**Tx W k 1**	2.8	0.59	0.56	0.8

**Tx W k 2**	0.84	1.35	1.18	0.9

**Tx 1**	0.82 0.53	0.54	0.5

**Tx 2**	1.58 0.72	0.76	0.9

**Tx 3**	0.51 1.47	1.64	0.9

**Tx 4**	0.47 0.44	0.45	0.4

**Tx 5**	0.94 0.54	0.59	0.5

**Tx 6**	1.11 0.47	0.41	0.5

**Tx 7**	0.82 0.39	0.39	0.3

**Tx 8**	0.68 0.65	0.64	0.5

**Tx Ex 1**	0.27 0.49	0.5	0.3

**Tx Ex 2**	0.56 0.92	0.66	0.4

**Tx Ex 3**	0.66 0.89	0.83	0.5

**Tx Ex 4**	0.1 1.33	1.29	0.5

**znf1**	0.41 0.66	0.61	0.6

**znf2**	0.15 1.57	1.26	0.6

**DNase1**	0.2 0.94	1.01	1.7

**Biv Prom 1**	0.15	1.95	1.72	1.3

**Biv Prom 2**	0.16	2.6	2.2	1.3

**Biv Prom 3**	0.29	2.52	2.28	1.3

**Biv Prom 4**	0.13	1.83	1.79	1.1

**Prom F 1**	0.2	2.9	2.83	1.0

**Prom F 2**	0.14	1.54	1.92	0.8

**Prom F 3**	0.15	0.8	1.22	0.5

**Prom F 4**	0.19	0.43	0.29	0.9

**Prom F 5**	0.14	1.79	1.73	1.1

**Prom F 6**	0.13	0.45	0.46	0.3

**Prom F 7**	0.16	0.46	0.45	0.4

**TSS 1**	0.12	0.42	0.52	2.8

**TSS 2**	0.11	0.67	0.52	5.6

100	0.1	0.1 0.84

**tRNA**

**state**

**G-rich**

**GC_rich**

**State**

**GapArtf2**

0.05 0.0

0.2

0.0	0.2	0.0	0.3	0.1	0.0	0.0	0.0	0.2 181.0 0.0	0.0

74.9

1.7	0.6	1.4	2.1

**GapArtf3**

0.01 0.0

0.2	0.0	0.2	0.0	0.3	0.0	0.0	0.0	0.0	0.3	144.8 0.0	0.0 579.7 0.0

1.4

0.0

4.1

**Q uies1**

9.88 1.5

1.1	3.0	1.4	1.2	0.7	0.1

1.4

0.4

1.2

0.6	0.1	0.4	1.2

0.5

0.4	0.6

0.1

0.6

**Q uies2**

3.07 0.9

1.2	3.8	0.9	2.6	2.4

0.0

1.0	1.2	0.5	0.9

0.3

4.7

1.3

1.1

0.2	0.5	0.9	0.8

**Q uies3**

12.2 1.2

1.3

0.6

1.1

0.5	0.6	1.7	1.5

0.4

1.0

1.7

0.1

0.1

1.0

0.6

1.3	1.3

0.6

0.7

**Q uies4 Q uies5 HET1**

4.45 0.2

1.69 1.8

0.71 0.3

0.8

1.2

0.8

0.0

0.0

0.0

2.8

1.5

1.3

0.0

0.6

0.0

1.3

1.1

3.5

4.5

0.2

0.1

0.5

1.3

0.4

0.1

1.1

6.5

0.1

1.0

0.0

0.6

1.1

0.5

1.6

0.2

0.1

0.2

1.1

6.2

0.4

1.1

0.6

0.5

0.6

2.0

0.5	0.7

1.2	1.3

0.0	0.3

0.2

0.4

2.3

0.5

0.8

0.4

**HET2**

0.69 0.8

0.7

0.0

0.5	2.2	1.2	0.1

0.2

2.3

0.9

0.4

0.1

4.9

0.7

0.8	0.0	0.1

1.2

0.2

**HET3**

1.36 0.1

1.2

1.7

3.4

0.0

1.4

0.1

0.2

0.2	0.4	0.1	0.9	0.0

0.4

0.5

0.6

1.1

0.2

0.6

**HET4**

0.56 0.2

0.8

0.0

2.0

0.0

2.3

0.1

0.3

0.3

0.0	0.2	56.3	0.0

0.3

1.2	1.6	1.1

0.5

1.5

**HET5**

0.25 0.0

0.5

0.0

1.0

0.0

4.7

0.4

0.2

4.0

0.0

0.6	9.1	0.0	0.1

3.4

0.6	0.5

3.1

1.8

**HET6**

0.58 0.1

0.4

0.0

1.1

0.0

4.3

6.1

0.1

2.5

0.0

0.7	12.3	0.0

0.1

6.8	0.5	0.3	0.9	0.9

**HET7**

1.02 0.0

0.6

0.0

1.2

0.2

3.8

12.4

0.3

1.8

0.0

1.7

0.9	0.0

0.2

1.3

1.1	0.6	1.2	0.4

**HET8**

0.43 0.2

0.9

0.0

1.1

1.2

4.1

0.4

0.8

4.1

0.0

0.4

0.8	0.3

0.7

3.5	0.7	1.3

1.1

1.1

**HET9**

1	0.3

0.8

0.0

1.2

0.8

3.3

0.6

0.6	0.4	0.3	0.6	13.0	0.0

0.5

0.9

1.3

0.8

0.2

1.4

**ReprPC1**

0.19 0.8

0.3

0.0	0.3	0.0	0.3	0.0	0.0	1.6

0.0

0.5

0.2	0.0

0.2

0.6

1.0	1.1

0.0

8.8

**ReprPC2**

0.32 2.5

0.9	3.7	0.6	0.0

0.7

0.0

0.6

1.8

1.5

1.0

0.7	0.0

1.0

0.8

2.0

1.2

1.3

5.7

**ReprPC3**

1.11 2.0

1.2

5.8	1.0

1.5

1.3

0.0

1.7

1.8

2.4	0.8	0.6

1.6

1.8

1.3

1.3

1.6

0.9

1.8

**ReprPC4**

3.93 1.4	1.3	2.4	1.2

1.1

1.0

0.0

1.7

0.8

1.3	1.2

0.3

0.4

1.4

0.8

0.9

1.3

1.0

0.8

**ReprPC5**

0.63 0.7

0.6

0.0

0.5

0.3

1.1

0.0

0.3

2.4

0.0

0.7

0.2

3.4

0.5

1.3

0.3	0.3

1.8

1.4

**ReprPC6**

1.51 0.6

0.9

0.6

0.5	1.9	1.4

0.1

0.8

1.8

0.5

1.3

0.2

0.2

1.0

1.2

0.1

0.7	3.5	0.6

**ReprPC7**

0.61 0.2

0.5

0.0

0.3	1.4	0.6	0.1	0.2	0.6	0.8

0.4

0.1	1.5	0.5	1.7	0.0	0.1	0.3

0.3

**ReprPC8**

0.48 1.3

1.1

0.0

1.0

0.2

2.0

0.1

0.8

2.1

0.0	0.8	0.1	0.0

1.6

2.0

1.3

1.0	2.4

1.6

**ReprPC9**

0.37 2.4	1.2

0.0

0.8

0.0

1.2

0.1

1.2

0.2	1.4	1.2	1.0

0.9

1.3

0.8

1.2

1.0	2.0

1.2

**Acet1**

0.18 0.0

0.5

0.0

0.8

0.0

2.0	1.0	0.4

7.1	0.0	0.2	0.6

2.6

0.1

2.9

0.2

0.2

2.1

0.4

**Acet2 Acet3 Acet4**

0.85 0.5

2.65 0.5

0.4 0.2

1.1

1.2

0.9

0.8

0.0

0.0

0.7

0.7

0.5

0.0

0.8

1.5

2.2

1.9

1.4

0.1

0.5

0.1

0.9

0.9

0.8

9.8

2.1

3.9

0.5

0.2

1.5

0.7

2.0

0.9

0.0

0.1

0.0

6.4

0.2

0.8

1.4

1.4

0.9

1.8

1.5

0.9

0.6	0.7

0.9	0.9

0.3	0.7

6.5

3.2

2.4

0.9

0.8

0.7

**Acet5**

0.86 1.1

1.4

0.0

0.7

1.1

1.5

0.2

1.4	2.6	0.5

1.3

0.0

0.9

1.8

1.3

1.5

1.2

1.5

0.5

**Acet6**

0.43 1.8

1.3

1.6

0.6

0.0

1.2

0.0

1.9	1.9	0.8	1.0

0.0

2.0

2.0

0.9

1.4

0.9

0.3

0.7

**Acet7 Acet8**

0.28 0.5

0.56 0.4

0.8

0.9

0.0

0.9

0.4

0.5

0.0

0.0

0.9

2.0

0.0

0.3

0.6

0.8

3.3

0.7

0.0

1.0

0.8

1.6

0.0

0.0

1.9

2.4

2.1

1.0

1.4

0.9

0.2	0.2

0.9	0.5

1.3

5.1

0.1

0.6

**EnhW k1**

1.54 2.1

1.5

1.5	0.8	2.1	0.8

0.2

1.7

0.2

2.7

0.8

0.1

1.6

1.7

0.6

1.2	1.4

0.2

0.4

**EnhW k2**

0.35 1.1

0.5

0.0

0.3

1.3

0.4

0.0

0.5

1.4

0.2

0.6

0.0

9.8

0.6

0.5

0.6

0.3

1.1

0.3

**EnhW k3**

0.83 2.7

1.0	3.8	0.4

2.3

0.8

0.1

1.1

2.1

3.6

1.0

0.0	7.8	2.6	0.5

0.7	0.7

0.7

0.4

**EnhW k4**

2.22 3.5

1.2	2.5	0.6	4.6

0.6

0.1

1.7

1.3

4.8

0.7

0.0

3.7

2.6

0.6

0.4

0.9

0.3

0.4

**EnhW k5**

0.99 1.2

1.3

1.0

0.8

3.4

1.0

0.2

1.8

1.1

1.3

1.5

0.1

0.9

1.4

0.5	1.4	1.4	0.8

0.3

**EnhW k6**

0.59 0.9

1.2

0.0

0.7

0.0

0.9

0.1

0.8	0.9	0.5	1.5

0.0	0.0

1.2

1.1

2.1

1.4

1.6

0.6

**EnhW k7**

0.48 1.4

1.3

0.0

0.8

0.0

1.1

0.1

1.4

1.7

0.0

1.5

0.0	0.0

1.3

0.9

2.3	1.8	1.2

0.9

**EnhW k8**

1.37 3.0

1.1

2.3	0.7	2.1	1.8

0.0

1.2

0.6

3.7

0.5

0.0

0.8

2.0

0.5

0.7	0.7

0.2

1.0

**EnhA 1**

0.18 0.5

0.4

0.0

0.2

0.0

0.3

0.0

0.5

0.3

0.0

0.5

0.0

4.6

0.9

0.7	0.0	0.2	0.0	0.5

**EnhA 2**

0.33 2.5

0.7

17.2

0.3	7.6	0.5

0.0

0.5

0.2

4.4

0.5

0.0

5.5

3.2

0.7

0.5	0.6

0.1	0.1

**EnhA 3**

0.19 2.3

0.9

0.0

0.3

1.0

0.4

0.0

0.9

0.9

2.8

0.5

0.0

7.7

2.2

0.2

1.3

1.2

0.2	0.3

**EnhA 4**

0.3 2.9

1.2

0.0	0.5	1.6	0.8	0.0

2.8

1.0

2.9

0.5

0.0	0.0

2.8

0.3

0.7

1.1

0.2	0.7

**EnhA 5 EnhA 6 EnhA 7 EnhA 8 EnhA 9 EnhA 10**

**EnhA 11**

**EnhA 12**

0.71 3.0

0.56 1.8

0.39 0.2

0.25 0.8

0.16 0.2

0.39 1.8

0.72 1.5

0.33 0.9

1.4

1.1

0.8

0.8

0.7

1.1

1.2

0.8

0.0

0.0

0.0

0.0

0.0

0.0

0.0

0.0

0.6

0.5

0.5

0.4

0.3

0.5

0.7

0.5

3.8

4.6

0.0

2.2

0.0

4.2

0.0

3.4

0.8

0.7

1.2

0.7

0.4

0.9

1.1

1.4

0.1

0.0

0.1

0.0

0.0

0.2

0.2

0.0

1.5

1.3

0.2

0.4

0.6

0.9

1.2

0.7

1.6

0.6

3.0

0.4

1.1

0.5

1.2

1.9

3.9

3.3

0.0

0.1

0.2

2.7

1.2

2.0

0.6

0.8

0.8

1.0

0.8

1.2

1.9

0.7

0.0

0.0

0.1

0.0

0.0

0.1

0.1

0.1

2.6

5.9

6.5

0.0

0.0

0.0

1.2

2.3

2.2

1.7

0.8

1.4

0.6

1.7

1.5

1.4

0.5

1.6

0.9

0.1

0.6

1.3

1.4

1.7

0.9

0.5

0.0

2.9

1.2

3.9

1.7

0.3

1.4

0.6

0.8

0.3

0.5

1.6

1.5

0.4

0.3	0.9

0.5	0.5

3.2	1.0

1.3	0.6

2.1	0.4

0.7	0.7

1.1	0.7

1.4	0.2

**EnhA 13**

0.76 2.3

1.4

0.0

0.7

1.5

1.3

0.1

1.0

0.8	1.1	1.0	0.1

1.1

2.0

1.5

1.7

1.5

0.9

0.7

**EnhA 14**

0.37 1.4

0.9

0.0

0.4

0.0

1.2

0.1

2.1

2.0

0.0

1.1

0.1

0.3

1.4

1.1

1.2

0.9

2.1

1.0

**EnhA 15**

1.02 1.6

1.2	2.4	0.6	1.7

1.3

0.2

2.4

0.2	1.9	1.5	0.1

0.5

1.6

1.0

2.4

1.1

0.8

0.7

**EnhA 16**

0.65 1.5

1.2

0.3

0.7

1.9

1.6

0.9

1.9

0.7

1.0

1.1

0.3

1.0

1.6

0.9

1.9

1.7

1.1

3.6

**EnhA 17**

0.53 3.1

0.9

2.6

0.4

4.5

1.0

0.0

1.2

1.4

3.4

0.4

0.0

2.9

2.2

0.6

1.0

0.6	0.3	1.1

**EnhA 18**

0.46 0.7

0.8

0.0

0.6

3.3

2.9

0.0

0.9

1.8

1.9

0.6

0.1

1.1

1.3

0.7	0.4	0.4	0.8	2.2

**EnhA 19**

**EnhA 20 TxEnh1 TxEnh2**

0.26 0.6

0.35 2.5

0.39 1.9

0.39 0.6

0.6

1.1

1.5

1.5

0.0

0.0

0.0

2.0

0.4

0.8

0.6

0.7

0.0

4.4

2.5

0.7

1.2

0.7

0.3

0.4

0.0

0.1

0.0

0.0

0.7

1.0

1.3

0.8

0.8

0.9

0.3

0.4

0.9

2.7

2.0

0.6

0.5

0.7

0.7

1.3

0.1

0.0

0.0

0.0

2.1

0.5

0.0

0.1

1.2

1.7

1.5

0.9

0.7

0.4

0.4

3.6

1.4

0.8

1.6

3.9

0.2

1.7

1.6

1.4

1.8

0.5

0.2

1.2

0.6

0.8

0.3

0.6

**TxEnh3**

0.25 0.1

1.2

0.0

0.6

0.0

0.9

0.2

0.4

7.4

0.1

1.7

0.1

8.7

0.5

3.4

1.2

0.8	9.1	0.6

**TxEnh4 TxEnh5**

0.27 0.2

0.5 0.5

0.5

0.6

0.0

0.0

0.3

0.4

0.0

0.2

0.3

0.6

0.0

0.1

0.3

0.3

2.0

2.1

0.0

0.0

0.5

0.7

0.0	0.0

0.1	2.2

0.7

0.3

0.9

1.2

0.0

0.9

0.6

0.5

2.1

2.7

0.0

0.5

**TxEnh6**

0.19 0.0

0.8

0.0

0.4

0.0

0.5

0.0	0.4	1.3	0.0

1.0

0.1	0.0

0.4

0.3	1.8	1.2

1.7

0.4

**TxEnh7**

0.27 0.5

0.7

0.0

0.4

9.4

0.5

0.0

0.8	0.7	0.5	0.8

0.0	0.0

0.9

2.8	0.3	0.4	1.9

0.4

**TxEnh8**

0.24 0.4

1.0

0.0

0.4

0.0

0.5

0.0

0.7

3.7

1.1

0.7

0.0	0.0

1.8

0.6

2.1	0.8	1.1	0.9

**TxW k1**

2.8 0.7

1.2

0.0

0.8	0.8	0.4	1.1	0.8

0.5

0.8

2.1

0.0	0.0

0.8

0.8

2.1

1.7

0.8

0.6

**TxW k2**

0.84 0.3

0.9

0.0

0.5

0.4

0.9

1.0

0.7

2.0

0.4

1.2

0.1	0.0

0.8

1.1

0.5	0.5	2.5

0.4

**Tx1**

0.82 0.7

1.7

1.4

1.1	2.5	0.4

0.1

1.5

0.9

0.8

1.7

0.0

0.4

0.7

1.4

2.9	3.0

1.1

0.5

**Tx2 Tx3**

1.58 0.8

0.51 0.3

1.4

1.2

1.4

0.0

1.0

0.6

0.9

0.2

0.4

0.9

0.5

0.2

1.3

1.7

0.5

3.2

0.4

0.2

1.9

1.9

0.0	0.1

0.0	2.1

0.8

0.9

0.7

1.0

2.5

1.0

1.8

0.6

0.8

5.0

0.5

0.5

**Tx4**

0.47 0.2

1.7

0.0

0.9

0.0

0.4

0.1

0.7	1.1	0.0

1.5

0.0

0.5

0.8

2.1

4.2

3.2

2.1

0.9

**Tx5**

0.94 0.2

0.9

0.0

0.4

0.0

0.5

0.8	0.5	1.6	0.0

3.7

0.1	0.0

0.6

1.2

1.7	1.4	2.9	1.0

**Tx6 Tx7**

1.11 0.8

0.82 0.2

1.5

1.7

0.0

0.0

0.9

0.7

0.0	0.2	0.1

0.0	0.3	0.0

1.8

1.2

0.4

0.7

0.9

1.6

0.5

0.6

0.0	0.0

0.1	0.0

1.0

1.1

0.6

1.2

3.1

3.4

2.4

2.8

0.3

0.9

1.3

0.8

**Tx8**

0.68 1.3

1.4

1.2

0.6	0.9	0.5	0.0

1.6

0.6

3.0

1.0

0.0

2.7

1.1

1.3

2.1

1.7

0.9

0.7

TxEx1

0.27 0.4

1.3

0.0

0.5

0.0

0.4

0.0

1.0

0.2

1.2

1.1

0.0	0.0

0.8

2.2

2.3

1.9

6.5

1.6

TxEx2

0.56 0.0

0.8

0.0	0.3	0.0

0.4

0.0	0.4	1.7	0.0

0.9

0.1	0.0

0.5

1.3

1.6

1.1

5.0

1.4

TxEx3 TxEx4

0.66 0.1

0.1 0.5

0.7

0.5

0.0

0.0

0.3	0.8

0.3	0.0

0.5

0.2

0.3

0.0

0.7

0.0

2.0

0.7

0.0

1.2

2.0

0.7

0.1	0.0

0.0	0.0

0.6

0.8

1.3

3.4

1.6

0.1

0.8

2.1

4.2

3.1

1.3

0.0

**znf1**

0.41 0.1

1.5

0.0

2.0

0.0

2.2	3.6	0.2

1.7

0.0

1.0

0.9	1.1	0.4	2.1

1.3	1.3

0.6

1.4

**znf2**

0.15 0.0

1.1

0.0

1.1

0.0

2.2

1.2

0.0

0.9

0.0

1.0	0.2	0.0	0.1	4.8

1.6

0.7

0.7

1.8

**DNase 1**

0.2 0.6

0.9

0.0

0.3

0.0

0.7

0.1

0.3

1.7

2.6

0.5

1.0	0.0

1.7

8.5

1.6	2.9	0.2	13.5

**BivProm 1**

0.15 0.3

0.1	0.0	0.1

5.2

0.1

0.0	0.0	0.0	0.0	0.2

0.5	0.0	0.2	1.3	0.0	0.5	0.8	11.6

**BivProm 2**

0.16 0.0	0.2	0.0	0.1	0.0	0.1

0.0	0.0	0.0

1.5	0.2	0.4	0.0	0.1	7.4	0.2	0.9

0.1

24.9

**BivProm 3**

0.29 1.0

0.4

0.0

0.3

0.0	0.4	0.0	0.5	1.3 0.8	0.5

0.3

4.5

0.5

1.5

0.6	0.3	0.0

7.7

**Biv Prom 4**

0.13 0.5	0.4

0.0

0.2	0.0	0.2	0.1

0.0	0.0

1.5

0.4

0.2	0.0

0.7

0.5

1.8	0.7	1.3	3.1

**Prom F1**

0.2 0.5

0.4

0.0

0.3

2.8

0.6

0.0	0.0

1.8

0.0

0.6

0.1

3.6

0.5

0.7

0.4

1.2

0.1

1.0

**Prom F2**

0.14 1.0

0.4

0.0

0.2

0.0	0.3	0.0

0.7

1.2

0.3

0.7

0.1	0.0

0.6

0.1

1.8

3.0

0.6

1.6

**Prom F3**

0.15 0.0

0.3

0.0

0.2	0.0	0.2	0.0	0.6	1.0

1.6

0.6	0.2	0.0	0.1	0.7

2.5

1.6

0.4

4.5

**Prom F4 Prom F5 Prom F6**

0.19 0.1

0.14 0.1

0.13 0.0

0.1	0.0	0.0	0.0	0.0	0.0	0.0	0.0	0.0	0.2	0.1	0.0	0.1	0.6	0.0

0.1	0.0	0.1	0.0	0.1	0.0	0.0	0.0	0.0	0.2	0.1	0.0	0.0	0.5	0.3

0.6	0.0	0.3	0.0	0.2	0.0	0.1	0.2	2.9	0.9	0.1	0.0	0.4	0.2	2.5

0.8

1.2

1.0

0.9

0.8

0.2

4.1

2.6

0.9

**Prom F7**

0.16 0.6	1.0	0.0

0.4

0.0

0.3

0.1

0.2	0.9

0.0

1.3

0.0	0.0	0.6	0.6

2.9

0.9

0.3	0.7

**TSS1**

0.12 0.3

0.1

0.0	0.0	0.0

0.1

0.0

0.3

0.0

0.6	0.3	0.2	0.0	0.1	0.0

2.1

4.3

1.0

49.7

**TSS2**

0.11 0.0	0.2	0.0	0.1	0.0

0.3

0.2

0.3	1.1

0.0

0.5

1.0	0.0	0.3	0.4

2.0

3.6	1.9	60.7

100 0.01 3.17 0.0003 20.37 0.001	8.52 0.13 0.01 0.004 0.001 12.65	0.40 0.001	0.04 0.01 0.004	0.01 0.01 0.003

**Supplementary Figure 28: Full stack states enrichments with RepeatMasker classes of repeats (A), low-complexity repeats and GC content (B), simple repeats (C)**. This is an extended version of Fig. 4C. In each panel, the rows correspond to full-stack states. The second column reports the percentage of the

genome that each full-stack state occupies. In **(A)**, columns 3-21 correspond to different repeat classes. In **(B)**, columns 3-7 correspond to 5bp windows in the genome are stratified by the number of G/C bases in them, columns 8-10 correspond to regions enriched with C-rich, G-rich, and GC-rich low complexity sequences, respectively, and column 11 shows enrichments for all low complexity sequences from RepeatMasker. States TSS1-2 are most enriched with Low complexity repeat class, which is consistent with these states having a high enrichment (19-20 fold) for windows in which all bases are a G or C. In **(C)**, columns 3-4 correspond to simple repeats of repeated (CA) and (TG) sequences, and column 5 shows enrichments for all simple repeats. State Acet1 is most enriched with simple repeats and this enrichment is mostly driven by enrichments with repeated CA and TG dinucleotides. In each panel, the values in all columns except the first and second columns correspond to fold enrichment for different repeat contexts in the full-stack states. Values are colored on a column-specific color scale. The last row gives the percentage of the genome that each repeat class occupies.

*Line 233-237: "We also saw specific states associated with the largest repeat classes of the genome, SINEs, LINEs, and LTRs. SINE repeats were most enriched in state Tx5 (3.7 fold) (Fig. 5C), which had high emission of H3K36me3 (Fig. 2A-B, Supp. Fig. 4-5). LINEs and LTRs repeats were most enriched for states in the H3K9me3-associated heterochromatin group with LINE most enriched in HET3 (3.4 fold), while LTRs were most enriched in HET5 (4.7 fold)" What's the biological explanation for these specific states enriched with specific repeats? Are there any published analyses that support these enrichments?*

We thank the reviewer for these questions.

The high enrichment of SINEs in a state associated with H3K36me3, a transcription-associated histone modification, is consistent with SINEs’ preference for gene-rich regions of the genome, while LINEs are more sparse in genic regions potentially due to negative selection against insertion of longer sequences [25]. The pattern of enrichment for SINEs in transcription-associated states is also consistent with previous analyses based on concatenated models for 127 reference epigenomes from Roadmap Epigenomics [26] and previous work based on a chromatin state model in a single cell type, human CD4T, using 41 chromatin marks [8]. State Tx5 is more enriched (3.7 fold) with SINEs compared to other full-stack states in the transcription group (the next highest enrichment is 2.1, some transcription states are depleted with SINEs). This higher enrichment in Tx5 may be related to this state not showing signals of H3K4me1 and acetylation marks, and showing moderate signal of H3K36me3 consistently across cell/tissue types.

The association of LTRs and LINEs with H3K9me3-associated states is also consistent with previous analyses based on a single cell type model and concatenated models [8,26]. HET5, the state with the greatest enrichment for LTR repeats, showed the strongest signals of H3K9me3 compared to other states in the heterochromatin group. LTRs’ strong enrichment with states showing high signals of H3K9me3 is also observed in previous analyses based on a single cell type (human CD4T) [8] and per-cell type analysis of the Roadmap Epigenomics data [26]. LINEs showed the strongest enrichment for HET3, while other states in the heterochromatin group showed weaker enrichments/depletions with LINEs. A notable property of HET3 is that it does not show signals of H3K27me3 and acetylation marks in any cell types. This property is something that would be difficult to recognize without the stacked modeling, and was only shared with HET4 and HET9 among states in the heterochromatin group. HET4 was also the second most enriched state in the heterochromatin group for LTRs (2.0-fold) while HET9 was not enriched, but is distinct in that it identifies regions where H3K9me3 is relatively specific to cell types in the embryonic and iPSC groups.

Our updated portion of the ‘Full-stack states show distinct enrichments for repeat elements’ results section discussing SINEs, LINEs, and LTRs now reads:

“We also saw specific states associated with the largest repeat classes of the genome, SINEs, LINEs, and LTRs. SINE repeats were most enriched in state Tx5 (3.7 fold) (**Fig. 5C**), which had high emission of H3K36me3 (**Fig. 2A-B, Supp. Fig. 4-5**), consistent with previous studies showing that SINEs are more enriched in gene-rich regions and in transcription-related states based on per-cell-type annotations [8,25,26]. In contrast, LINEs are depleted in most transcription-related states, reflecting the negative selection against long-sequence insertions in or near genes [25]. LINEs are most enriched in state HET3 (3.4 folds) (**Supp. Fig. 28**), and a notable property of this state is it does not show signals of H3K27me3 and acetylation marks across cell/tissue types. This property of HET3 is a pattern that would be difficult to recognize without stacked-modeling, and was only shared with HET4 and HET9 among states in the heterochromatin group. HET4 was also the second most enriched state in the heterochromatin group for LTRs (2.0-fold) while HET9 was not enriched, but is distinct in that it identifies regions where H3K9me3 is relatively specific to cell types in the embryonic and iPSC groups. LTRs are most enriched in state HET5 (4.7 fold), and this state is marked by its highest signals of H3K9me3 compared to other states in the heterochromatin group (**Fig. 4C, Supp. Fig. 28**). LTRs showing strong enrichment with states associated with strong presence of H3K9me3 is consistent with per-cell-type chromatin state analyses [8,26]. We also confirmed that the increased predictive power of the full-stack model over per-cell-type annotations, which was previously seen for repeat elements overall, also held for most of the individual repeat classes (**Supp. Fig. 29**). Overall, results of enrichment of full-stack state annotations for repeat classes offer further details in stratifying the states’ characteristics, and concurrently confirm and refine existing knowledge about different repeat classes chromatin state associations. “

*Line 271-278: "This is consistent with previous analysis using cell-type-specific annotations showing that ConsHMM state 100 was enriched in a ZNF gene-associated chromatin state [32]. Interestingly though, another full-stack state (ZNF2) that was even more strongly enriched for ZNF genes (68.6 folds), had 0.4 fold enrichment for ConsHMM state 100, and instead was most enriched with ConsHMM state 1 (Fig.*

*3A- B, Supp. Fig. 22). Therefore, the full-stack annotation helped distinguish two ZNF-gene associated states that are associated with distinct conservation states." Indeed ZNF1 and ZNF2 differ in their enrichments with ConsHMM state 100, but what does this result mean biologically? Do ZNF1 and ZNF2 belong to different classes of ZNF proteins with different biology?*

We thank the reviewer for this question. We realized that we did not correctly describe the results in the figure as the enrichment in these two states and a third state got confused. This is now corrected in the revised version and we have conducted additional analyses on the ZNF named genes. Specifically, we broke the ZNF named genes into the C2H2-type and non-C2H2-type sets of genes, and found the overall enrichment with znf2 and znf1 to be stronger for the C2H2-type genes (86.7 and 25.1 fold) compared to for non-ZNF genes (17.8 and 8.9 fold). In both cases, state znf2 showed stronger enrichments compared to znf1. The znf1 and znf2 states also have a large difference in enrichments for coding genes (10.4 vs. 1.1).

In our revised text we now state:

“ConsHMM state 100, a conservation state associated with alignment artifacts, was 10.9 fold and 2.1 fold enriched for full-stack state ZNF2 and ZNF1, respectively (**Fig. 3A-B, Supp. Fig. 30**). This is consistent with previous analysis using per-cell-type annotations showing that ConsHMM state 100 was most enriched in a ZNF gene-associated chromatin state [27]. State znf2 also showed a 5.4-fold enrichment for ConsHMM state 1 which contrasts with state znf1, which showed a 0.8 fold enrichment for ConsHMM state 1. This

difference is consistent with the znf2 state’s larger fold enrichment for coding exons than znf1 (10.4 vs 1.1). The znf2 state also had a greater fold enrichment for ZNF named genes in general (68.6 vs. 20.8 fold), with those enrichment stronger and the difference greater when restricting to C2H2 annotated genes (86.8 vs.

25.1 fold) (**Fig. 3A-B, Supp. Fig 51**). Therefore, the full-stack annotation helped distinguish two ZNF-gene associated states, which are associated with distinct conservation states. As this example illustrates, the full- stack annotation captured conservation state enrichments that were generally consistent with those seen in per-cell-type annotations, but could also identify additional refined enrichment patterns.”

**Enrichment of full-stack states major ZNF gene states**

| **State** | **Genome %** | **ZNF* gene** | **ZNF* C2H2 gene** | **ZNF* not C2H2** |
| --- | --- | --- | --- | --- |
| **G apArtf1** | 11.86  0.05  0.01  9.88  3.07  12.23  4.45  1.69  0.71  0.69  1.36  0.56  0.25  0.58  1.02  0.43  1.00  0.19  0.32  1.11  3.93  0.63  1.51  0.61  0.48  0.37  0.18  0.85  2.65  0.40  0.86  0.43  0.28  0.56  1.54  0.35  0.83  2.22  0.99  0.59  0.48  1.37  0.18  0.33  0.19  0.30  0.71  0.56  0.39  0.25  0.16  0.39  0.72  0.33  0.76  0.37  1.02  0.65  0.53  0.46  0.26  0.35  0.39  0.39  0.25  0.27  0.50  0.19  0.27  0.24  2.80  0.84  0.82  1.58  0.51  0.47  0.94  1.11  0.82  0.68  0.27  0.56  0.66  0.10  0.41  0.15  0.20  0.15  0.16  0.29  0.13  0.20  0.14  0.15  0.19  0.14  0.13  0.16  0.12  0.11 | 0.21 | 0.14 | 0.42 |
| **G apArtf2** |  | 1.44 | 1.68 | 0.79 |
| **G apArtf3** |  |  |  |  |
|  |  | 1.26 | 1.54 | 0.60 |
| **Quies1** |  | 0.40 | 0.03 | 1.51 |
|  |  | 0.29 | 0.11 | 0.83 |
| **Quies2** |  |  |  |  |
|  |  | 0.55 | 0.41 | 0.99 |
| **Quies3** |  |  |  |  |
|  |  | 0.80 | 0.66 | 1.20 |
| **Quies4** |  |  |  |  |
|  |  | 0.20 | 0.22 | 0.13 |
| **Quies5** |  |  |  |  |
| **H ET1** |  | 0.33 | 0.11 | 1.00 |
|  |  | 0.66 | 0.80 | 0.17 |
| **H ET2** |  |  |  |  |
|  |  | 1.77 | 1.83 | 1.58 |
| **H ET3** |  |  |  |  |
|  |  | 6.03 | 7.02 | 3.68 |
| **H ET4** |  |  |  |  |
|  |  | 13.97 | 16.70 | 7.34 |
| **H ET5** |  |  |  |  |
|  |  | 2.58 | 2.93 | 1.43 |
| **H ET6** |  |  |  |  |
|  |  | 2.04 | 2.15 | 1.70 |
| **H ET7** |  |  |  |  |
|  |  | 0.87 | 0.81 | 0.98 |
| **H ET8** |  |  |  |  |
|  |  | 1.44 | 1.56 | 1.00 |
| **H ET9** |  |  |  |  |
| **ReprPC1** |  | 0.30 | 0.12 | 0.86 |
|  |  | 0.28 | 0.17 | 0.59 |
| **ReprPC2** |  |  |  |  |
|  |  | 0.36 | 0.28 | 0.59 |
| **ReprPC3** |  |  |  |  |
|  |  | 0.29 | 0.23 | 0.46 |
| **ReprPC4** |  |  |  |  |
|  |  | 0.38 | 0.27 | 0.70 |
| **ReprPC5** |  |  |  |  |
|  |  | 0.32 | 0.25 | 0.52 |
| **ReprPC6** |  |  |  |  |
| **ReprPC7** |  | 0.61 | 0.61 | 0.58 |
|  |  | 0.37 | 0.31 | 0.56 |
| **ReprPC8** |  |  |  |  |
|  |  | 0.53 | 0.47 | 0.70 |
| **ReprPC9** |  |  |  |  |
| **Acet1** |  | 1.09 | 1.00 | 1.42 |
| **Acet2** |  | 0.37 | 0.24 | 0.73 |
| **Acet3** |  | 0.40 | 0.28 | 0.74 |
| **Acet4** |  | 0.38 | 0.41 | 0.25 |
| **Acet5** |  | 0.32 | 0.30 | 0.36 |
| **Acet6** |  | 0.46 | 0.48 | 0.38 |
| **Acet7** |  | 0.39 | 0.45 | 0.19 |
| **Acet8** |  |  |  |  |
|  |  | 0.31 | 0.26 | 0.45 |
| **EnhW k1** |  | 0.59 | 0.35 | 1.30 |
|  |  | 0.66 | 0.64 | 0.75 |
| **EnhW k2** |  |  |  |  |
|  |  | 0.72 | 0.62 | 1.02 |
| **EnhW k3** |  |  |  |  |
|  |  | 0.59 | 0.42 | 1.08 |
| **EnhW k4** |  |  |  |  |
|  |  | 0.60 | 0.49 | 0.91 |
| **EnhW k5** |  |  |  |  |
|  |  | 0.72 | 0.62 | 0.99 |
| **EnhW k6** |  |  |  |  |
|  |  | 0.50 | 0.58 | 0.21 |
| **EnhW k7** |  |  |  |  |
|  |  | 0.42 | 0.28 | 0.84 |
| **EnhW k8** |  |  |  |  |
| **EnhA1** |  | 0.73 | 0.88 | 0.28 |
|  |  | 0.80 | 0.85 | 0.61 |
| **EnhA2** |  |  |  |  |
|  |  | 0.82 | 1.00 | 0.20 |
| **EnhA3** |  |  |  |  |
|  |  | 0.54 | 0.58 | 0.37 |
| **EnhA4** |  |  |  |  |
|  |  | 0.55 | 0.47 | 0.77 |
| **EnhA5** |  |  |  |  |
|  |  | 0.91 | 0.98 | 0.65 |
| **EnhA6** |  |  |  |  |
|  |  | 0.50 | 0.39 | 0.80 |
| **EnhA7** |  |  |  |  |
|  |  | 0.71 | 0.56 | 1.09 |
| **EnhA8** |  |  |  |  |
|  |  | 0.89 | 1.14 | 0.07 |
| **EnhA9** |  |  |  |  |
| **EnhA10** |  | 0.92 | 1.03 | 0.61 |
|  |  | 0.73 | 0.78 | 0.55 |
| **EnhA11** |  |  |  |  |
|  |  | 0.31 | 0.24 | 0.50 |
| **EnhA12** |  |  |  |  |
|  |  | 0.27 | 0.19 | 0.52 |
| **EnhA13** |  |  |  |  |
|  |  | 0.34 | 0.29 | 0.52 |
| **EnhA14** |  |  |  |  |
|  |  | 0.42 | 0.32 | 0.71 |
| **EnhA15** |  |  |  |  |
|  |  | 0.41 | 0.35 | 0.57 |
| **EnhA16** |  |  |  |  |
| **EnhA17** |  | 0.94 | 0.99 | 0.73 |
| **EnhA18** |  | 0.49 | 0.49 | 0.47 |
|  |  | 1.12 | 1.25 | 0.64 |
| **EnhA19** |  |  |  |  |
| **EnhA20** |  | 0.77 | 0.77 | 0.69 |
| **Tx Enh1** |  | 1.14 | 1.40 | 0.27 |
|  |  | 1.95 | 2.14 | 1.24 |
| **Tx Enh2** |  |  |  |  |
|  |  | 3.15 | 3.76 | 1.05 |
| **Tx Enh3** |  |  |  |  |
|  |  | 1.47 | 1.76 | 0.58 |
| **Tx Enh4** |  |  |  |  |
|  |  | 1.42 | 1.69 | 0.61 |
| **Tx Enh5** |  |  |  |  |
|  |  | 1.14 | 1.44 | 0.11 |
| **Tx Enh6** |  |  |  |  |
|  |  | 1.28 | 1.59 | 0.24 |
| **Tx Enh7** |  |  |  |  |
|  |  | 1.22 | 1.41 | 0.57 |
| **Tx Enh8** |  |  |  |  |
| **Tx W k1** |  | 1.71 | 1.84 | 1.37 |
|  |  | 1.27 | 1.28 | 1.21 |
| **Tx W k2** |  |  |  |  |
| **Tx 1** |  | 2.24 | 2.25 | 2.11 |
|  |  | 1.74 | 1.88 | 1.28 |
| **Tx 2** |  |  |  |  |
|  |  | 1.64 | 2.03 | 0.32 |
| **Tx 3** |  |  |  |  |
|  |  | 2.70 | 3.01 | 1.55 |
| **Tx 4** |  |  |  |  |
|  |  | 2.17 | 2.51 | 1.03 |
| **Tx 5** |  |  |  |  |
|  |  | 1.71 | 1.79 | 1.58 |
| **Tx 6** |  |  |  |  |
|  |  | 1.91 | 2.13 | 1.23 |
| **Tx 7** |  |  |  |  |
|  |  | 1.29 | 1.40 | 0.92 |
| **Tx 8** |  |  |  |  |
| TxEx1 |  | 2.61 | 2.97 | 1.27 |
| TxEx2 |  | 2.45 | 2.85 | 1.10 |
| TxEx3 |  | 2.60 | 3.14 | 0.94 |
| TxEx4 |  |  |  |  |
|  |  | 2.17 | 2.55 | 0.91 |
| **znf1** |  | 20.83 | 25.12 | 8.91 |
|  |  | 68.59 | 86.77 | 17.81 |
| **znf2** |  |  |  |  |
| **D N ase1** |  | 0.80 | 0.77 | 0.86 |
| **B iv Prom 1** |  | 0.87 | 0.66 | 1.44 |
|  |  | 0.50 | 0.34 | 0.95 |
| **B iv Prom 2** |  |  |  |  |
|  |  | 0.82 | 0.80 | 0.83 |
| **BivProm 3** |  |  |  |  |
|  |  | 1.28 | 1.13 | 1.78 |
| **BivProm 4** |  |  |  |  |
| **Prom F 1** |  | 0.81 | 0.76 | 0.91 |
|  |  | 1.23 | 1.34 | 0.85 |
| **Prom F 2** |  |  |  |  |
|  |  | 3.39 | 3.95 | 1.60 |
| **Prom F 3** |  |  |  |  |
| **Prom F 4** |  | 4.78 | 5.61 | 2.20 |
|  |  | 2.27 | 2.34 | 2.19 |
| **Prom F 5** |  |  |  |  |
|  |  | 4.34 | 5.14 | 2.05 |
| **Prom F 6** |  |  |  |  |
|  |  | 4.41 | 5.24 | 1.76 |
| **Prom F 7** |  |  |  |  |
| **TSS1** |  | 3.75 | 4.31 | 1.98 |
|  |  | 2.13 | 2.27 | 1.76 |
| **TSS2** |  |  |  |  |
| Base % | 100 | 0.73 | 0.57 | 0.18 |

### **Supplementary Figure 51: Full-stack states enrichments with different subsets of ZNF genes.** The rows correspond to full-stack states. The first column presents the state labels, the second presents the percentage of the genome that each state occupies, and the remaining three columns enrichments for different subsets of zinc finger genes. The first of these is all genes with a ZNF symbol. The second is the subset of ZNF genes also annotated as C2H2 genes and the third those that are not C2H2 genes. The values correspond to the full-stack states’ fold enrichment for the ZNF gene families. Values are colored on a column-specific color scale. The last row gives the percentage of the genome that each type of ZNF gene family occupies

*Line 288-290: "Consistent with those previous results, using the full-stack model, we observed that of the 13 states that were in the top 10 maximally enriched states with either deletions or duplications (1.18 fold or greater), seven were in the heterochromatin group (HET1-2,4-7,9) and one was in the ZNF gene state (ZNF2)". Why are these states enriched in deletions or duplications? Any biological explanation? Or do you mean these regions have annotation artifacts?*

We thank the reviewer for these questions. The enrichment of HET states makes sense biologically. As structural variation can have large effects, they would be expected to experience negative selection in more conserved and functional parts of the genome, while being more tolerated in the heterochromatic regions of the genome marked by H3K9me3. These states also showed higher enrichment for common SNPs (**Supp. Fig. 34**). There is less of a clear biological explanation for the ZNF2 enrichment and there is the possibility the enrichment could be related to technical issues as this state was also the state most enriched for a conservation state associated with putative alignment artifacts. We had also noted that Abel et al., 2020 [28], deletion and duplication were also most enriched in heterochromatin and znf-gene states in per-cell-type chromatin state annotations.

We note that we made small updates to this analysis since we realized that we included bases in hg38 where multiple bases in hg19 could map to, but did not intend to. We updated **Fig. 5A-B** to reflect this, and still observed states associated with alignment artifacts, heterochromatin and znf- genes to be among the top 10 enriched states.

In the revised text we have now updated our discussion of the top 10 most enriched states to now read:

“Consistent with those previous results, using the full-stack model, we observed that of the 13 states that were in the top 10 maximally enriched states with either deletions or duplications (1.18 fold or greater), seven were in the heterochromatin group (HET1-2,4-7,9) and one was the ZNF2 state (**Fig. 5A, Supp. Fig. 32**). The enrichment of structural variation in HET states is consistent with the notion that potentially larger effect structural variants would less likely experience negative selection in these regions of the genome. As the ZNF2 state is most enriched for a conservation state associated with putative alignment artifacts this raises the possibility that technical issues may be contributing to the SV enrichments (**Supp. Fig. 30**). The other five states included two artifact states (GapArtf2-3) and three quiescent states (Quies1-2,4) (**Fig. 5A**). The quiescent states Quies1-2,4, despite the generally low frequencies for all marks, did have higher emission probabilities for H3K9me3 compared to other chromatin marks (**Fig. 5B**).”

*Line 329-331: "Some other scores, however, showed relatively weak enrichments or even depletions for these promoter- and TSS- associated states." Why do these other scores have lower enrichment for promoters?*

We thank the reviewer for the question. In general, the relative enrichment of prioritized bases of a score for different states will depend on the specifics of features the method considers and how they are integrated to derive a score. We have now added a discussion of the fitCons and FIRE scores in this context as they both showed depletions of prioritized bases in TSS- and promoter-associated states. The fitCons score showed depletions for three of these states and relatively weaker enrichment for the other promoter states. One possible explanation is that fitCons uses information on depletion of human polymorphisms to score annotations and promoters can have increased mutation rates (associated with their high CpG content, see response to comment below). The prioritized variants from the FIRE method are depleted in the bivalent promoter states (BivProm) and PromF5, which are generally associated with low average expression **(Fig. 3C)**. This depletion is consistent with the fact that FIRE method prioritizes variants based on annotations predictive of eQTLs active in LCLs, and few eQTLs are expected to be found proximal to genes with no or limited expression in LCLs.

We have revised the text to now state:

“Seven scores (DANN, Eigen, Eigen_PC, funSeq2, CDTS, CADD and REMM) had their top five enriched states exclusively associated with promoter and TSS states, with enrichments ranging between 8.6 and 70 fold (PromF2-5, TSS1-2, BivProm1-2,4) (**Fig. 5C**). In contrast, the fitCons score showed depletions for three of these states and relatively weaker enrichment for the others. This difference might be related to fitCons’ approach of prioritizing bases showing depletion of human genetic polymorphisms, potentially without sufficiently accounting for the increased mutation rates in regions with high CpG content that are observed in promoter-associated states (**Supp. Fig. 42**) [29]. FIRE’s prioritized variants showed depletions in the bivalent promoter states (BivProm) and PromF5, which have generally lower average gene expression across cell types (**Fig. 3C**). This depletion is reflective of the fact that FIRE was trained to prioritize variants in cis-expression quantitative trait loci (cis-eQTLs) in one cell type (LCL) [30], and few eQTLs are expected to be proximal to genes with limited or no expression in that cell type. “

*Line 335-344: "Enhancer states EnhA2-3,17 were among the states in the top five most enriched for FATHMM, GERP++, LINSIGHT, PhastCons, and PhyloP prioritized non-coding variants. These states also showed consistent enrichments with variants prioritized by Eigen, funSeq2, CADD, and REMM, though those scores showed even stronger relative enrichments for promoter states. In contrast, FIRE, DANN and CDTS were depleted for prioritized variants in all these enhancer states, and Eigen_PC showed both enrichments and depletions (Fig. 6C). FIRE and fitCons showed strong enrichment for exon states (TxEx1-3), which are associated with coding regions, even though coding bases were excluded in this analysis (Fig. 6C). FATHMM had the greatest relative enrichment for the primary DNase state associated with CTCF cell type-specific chromatin states (DNase1) (~10 fold), and was the only score for which this state was among the top five most enriched states (Fig. 6C, Supp. Fig. 28)." What do*

*these different enrichment biases mean? Do these means promoter/enhancer/exon are with different conservation patterns biologically or some technically score bias?*

We thank the reviewer for the questions. These enrichment biases mean different scores tend to prioritize different types of genomic regions. As the different scores vary in the types of genomic annotation features they take as input and the predictive model used to map those input annotations to prioritization scores, it is expected that different scores will have higher values in different classes of genomic regions. This

analysis is not directly trying to conclude one score is preferred over another, which will likely depend on the specifics of the application or evaluation criteria. However, by understanding the biases of different scores for different regions, one can gain an expectation for what applications that different scores can perform better. In terms of whether specific enrichments represent biological or technical biases, while in general it is difficult to conclude confidently one way or the other, one can gain insight into this by considering the biological interpretation of the state and the enrichment levels of the other scores. For example, the inconsistent enrichment of prioritized bases across scores for the GapArt2-3 states, associated with the characterization of this state as putative sequencing artifacts, is suggestive of technical biases in some scores. Similarly, DANN’s top 1% non-coding bases showing enrichment for five heterochromatin states, while not showing enrichment for any enhancer state, and no other scores showing enrichment for those heterochromatin states is also suggestive of a technical bias.

We have added a paragraph to this results section discussing these points:

“Overall, this analysis shows that the scores tend to prioritize bases in different epigenetic contexts. As these scores vary in the genomic features selected as input, and the predictive model for scoring bases, it is expected that different methods may show higher scores for in different classes of genomic contexts. By analyzing the state enrichments, one can gain some expectations for what types of evaluation criteria different scores might perform better, but we note that this analysis is not trying to directly conclude one method is preferred. Also, while in general it is difficult to conclude confidently what enrichments are due to technical or biological biases, by comparing enrichments across scores and considering what else is known about the states, one can still gain insights into this. For example, the inconsistent enrichments of different methods for prioritized variants in GapArtf2-3 states (**Supp. Fig. 36**), along with these states’ association with sequencing artifacts, is suggestive of technical biases. Similarly, DANN’s top 1% non- coding bases showing enrichments in five heterochromatin states, while not showing any enrichments in enhancer states, and no other scores showing enrichments in heterochromatin states, is also suggestive of technical biases **(Supp. Fig. 37)**.

*Line 370-371: "TSS and promoters associated states, PromF4 and TSS1- 2, were maximally enriched for variants in the lowest range of MAF (0 < MAF <= 0.0001), 1.5-1.7 fold." Why would these highly functional states be enriched in rare variants? Shouldn't these states be depleted in rare variants? As shown in Fig. 3A, these states are highly conserved evolutionarily and, as a corollary, one would think they should be depleted of rare variants.*

We thank the reviewer for this question. The presence of rare variants can also be heavily influenced by the underlying mutation rates associated with different parts of the genome. As increased CG dinucleotide content has previously been associated with higher mutation rates [29], in response to this question, we directly analyzed the state enrichments for CG dinucleotides. These states (TSS1-2, PromF4) are the three most enriched states for CG dinucleotides (**Supp. Fig. 42**; also shown below), therefore, they are expected to show higher mutation rates. At the same time, purifying selection is more effective at eliminating large- effect common variants than large-effect rare variants. We think these two reasons may explain why PromF4 and TSS1-2 were maximally enriched for variants in the lowest range of MAF (0 < MAF <= 0.0001).

We modified the text to now state:

“The high enrichment for states PromF4 and TSS1-2 for rare variants, despite their being the most enriched states with PhastCons conserved elements, can be explained by these states’ high enrichment of CpG dinucleotides, which are associated with higher mutation rates (**Fig. 3A, Supp. Fig. 42) [29]**. At the same

time, purifying selection can have a weaker effect on large-effect rare variants than on large-effect common variants.”

We added subpanel B to supplementary figure 42 showing CG dinucleotide enrichments, also shown here:

A	**Enrichment of full-stack states with GNOMAD variants**

0.2S	0.35	0.34	0.35	0.34

0.74	U	1.22	1.21	1.17

0.59 1.J7 I.II 121 1.21

LU	1.1	1.12	1.1'	1.15	1.17

Ill	112	1.1	111

1.22 l .JJ 1.21 1.Jl 1.16 1.16 1.16 1.16 0.9 0.87 0.87	0.85 1.11 1.11 1.15

1.25 1 21 1.25

1	0.99	0.97	0.96

1.11	1 oa 1.12	1.01

1.05	1.03	1.03

Ill	121 121

**lU	LM UI**

1.06 1.06	UM 1.05 1.04 1.13 1.14 1.13	I.DI

1.1	107	1.09	1.06	I .OS	1.04	0.99	103	1.1

1.01	1	0.96	0.93	0.97	0.93	0.95	0.94	0.87

0 .99	0 .99	0.99	0 99	UM	1.08	l 05	0 .97	l

0.94	0.94	0.93	0.95	0.96	l	1.02	l

1 03	1 CM	1.06	1.01	1.12	UC	1.16	1.17	111

111	1.11	1.08	1.09	1.07	1.12	1.16	1.14	1.11

I 11	1.11	1.09	1.12	1.13	1.17	1.11	1.2	1 lt

1.19	1.17	1.11	1.12	1.07	1.12	1.09	1.12	1.12

0.68 0.86 0 .83	0.8	0.75	0.72	0.65	0.58	0.57	0.58	0.55

1.05	1.03	104	I .OS 1.09	1.01	1.11	1.13

=1m	1m 1 =

LU	1.1 1.1	111	1.11 1.13	1.16	1.17	1.16	1.15	116

1 = 1m 1m 1 =

=1u

1 1 =

11	1 ,04	UM 1 OS 1 .03 1 .06 UM	I. I	l 09 1.11	1.09

= = 1m **1	1m**	=

1 D7	0.99	0.99	0.98	0.97	1 0.97	0.99	0.98	1.01

LM 1 .05	1.03	1.02	0.99	0.98	0.94	0.97	0.99	1.06	1.02

U7	1.14	1.11	1.13	1.11 1.U	1.12	US	1.17	1.11	l.2

l 04 0.91 0.91	1	1	1	1 1.03 1.02 103	1.04

us	1.12	1.08	1.02	103	0.93	0.96	1 0.96 0 .99

l l	1 .03	1.01	1.01	0.99	1	0.99	1	0.99	1.06

= =	=	=	= = =	= 1	=

l 07	1	1	1	0.99	1	1	1.01	1	0.99	1

l 07	1.07	1.09	11 1 .13 U4	1.16	1.16	1.17	l lt	117

l 07	1.04	1.06	1 04	1.08	1.07	1.12	1.13	12	1.16	116

l 07	1	1	0.99	0.97	t	1.03	1.04	1.04

I.II 1.09 1.05 1 02 0.97 0 92 0.84 0.86 0.87	0.83	0 .84

l l	1.01	0.98	0.98	0.94	0.92	0.89	0.88	0.94	0.86	0.86

l 05 0.94 0.91 0.91 0.89 0.87 0.83 0.81 0.84 0.86 0 .82

1.04	0.94	0.92	0.93	0.91	0.9	0.87	0.89	0.89	0.9	0.85

l 03 0.94	0.95	0.94	o.94	O 92	0 .93	0 .94 0.92 0.94 0 .94

Lot	1	0.99	0.98	0.95	0.93	0.9 0.93	0.96 0.94	0 .9

U1 1.11	1.07	1 07	1.06	1.06	1 04	1.1 1 .09	1.1 1 .11

LU 1 .06 1.04 1.02 1.03	1.02	1	1.01	1.06	1.02	0.99

LM	1.05	102	0.98	0.94	0.91	087	0.89	0.87	o.78	o.79

l.05	1 1.01 0.99 0.99 0.99	0.98	l	0.99 0.99 0.96

l 07	1.07	1.09	11 1 .13	1.14	1.19	1.2	1.11	1.22	117

1.12 1.05	1.03	1 01	1 0.97	0.98	1.04	1	1.03

LOI	1	1.01	1	1.02	1.02	1.05	1.07	1.06	1.07

LU	1.04	1.02	1.01	1	0.97	1.01	1.02	1.04	1.07

l 07	1.03	1.04	1 OS	1.06	1.07	1.08	1.11	1.11	US	116

107	1.06	108	108	1.1	1.1	1.12	1.13	1 .15	1.14	l .U

LOI	0.95	0.94	0.93	0.91	0.9	0.86	0.88	0.89	0.86	0.87

UI	1.11	I 09	1.09	1.01 1.01 1.07	1.09	1.1	1.09	1.11

LM	1.05	1 03	1.02	0.96	0.97	0.92	0.94	0.94	0.92	0.93

l 04	0.96	0.97	0.97	0.97	0.97	0.95	0.97	1	102	1.04

l 03	0,93	0.92	0.91	0.89	0.86	0.81	0 .81	0.77	0.8	0.81

Lot 0.98	0.97	0.95	0.93	0.86	0.82	0.79	0.76	0 .71	0.73

1.11 1.08	104	1.01 0.93	0.83	0.84	0.8	0.77	0.76

1.13	105	0.99	0.9	0.85	0.75	0.72	0.66	0 .71	0.72

1.17	Ul	1 06	1	0.97	0.89	0 .88	0.87	0.9	0.94

LM 1.05	1 0.96	0.92	0.89	0.9	0.85	0.8	0.8	o 8

I.II 1.11 1.06 1.03 0.98 0.93 0.85 0.81 0.87 0.87 0.86

l l	0.98	0.93	0.92	0.86	0.81	0.75	0.78	0.75	0.77	0.75

l 06	1.01	I 09	1.09	1.11	1.09	1.08	1.04	1.03	1.02	1.01

U1 1.12	I 09	107	1.04	1.02	0.98	0.99	0.98	0.95	o.96

Lot	1.04	1.03	1.02	1.01	0.96	0.91	0.88	0.84	0.84	0.8

1 DI 1.09	1.1	11	1.12	1.1	1.09	1.04	1.03	1.02	1.01

LM 1.09	1.06	1.06	1.01	1.01 0.96	0.93	0.96	0.96	0.95

l 01	t 0.98 0 .95 0.94 0.87 0.85	0.8	0.78	0 .75	0.72

LU	l .lt	1.17	1.15	1.16	1.11	1.09	1.06	1.03	1.04	1 .02

1	0.9	0.88	0.88	0.86	0.8	0.77	0.73	0.73	0.71	0.74

I.OJ	0.9	0.87	0.84	0.81	0.75	0.68	0.67	0.68	0.63	0.64

0.95	0.94	0.91	0.91	0 .88	0.82	0.82	0.83	0 82	0.82

1 0 .96	0 .9	0.85	0.79	0.72	0.67	0.65	0.61	0 .66

1	0 94	0.88	0.82	0.76	0.67	0.66	0.63	0.6	0.62

11 1 OS	1.01	0.95	0.89	0.85	0.87	0.84	0.83

1.02 0 .93 0.88	0.8	0.73	0.72	0.69	0 .64	0.68

1 OS	1 OS	1.01	0.98	0.94	0.91	0.92	0.96	0 .93

0 .99	1 01	0.97	0.9 0.84 0.94 0.82	0 .95	0 .93

1.15	1.14	1.13	1.15	1.14	1.14	1.14	1.12	1.11

1.05	1.03	0.97	0.95	0.9 0.9 0.82 0.81	0.87

1.06	1.03	0.97	0.94	0.95	0.89	0.87	0.85	0.83

1.1	109	1.05	1.05	1.02	1.04	1 0 .91 0.97

0.97	0.96	0.94	0.93	0.9	0.9	0.88	0.84	0.84

1.08	1.06	1.04	1	0.97	0.96	0.96	0.94	0.97

1.01	0.95	0.91	0.89	0.81	0.8	0.81	0.78	0 .8

0 97	0.94	0.89	0.84	0.77	0.77	0.68	0.71	0.69

l .2J	1.16	1.05	0.95	0.86	0.8 0.79 0 .74	o 8

0.96	0.95	0.92	0.85	0.83	0.85	0.82

0.83	0.78	0.77	0.71	0.76

0.88	0.83	0.84	0 84	0.82

1.02	1.02	1.1

1.11 1.15

0.04 0.03	0.02

E

·

;,

0 . 29

1.18

117

1.21

118

0.78

1"

1.39

1.03

121

13

1.39

1.37

1.17

12

0.98

103

1.01

111

1.19

12

1.21

0.66

1.1

11

115

11

1 ..

1.02

1.05

1.18

1.01

1.07

1.02

1.12

1.04

1.11

104

0.95

0.93

0.86

0.92

0.94

0.95

116

1.07

093

0.98

1.13

1.05

1.04

1.05

1.08

1.11

0.91

115

1.01

1

0.86

0.83

0.92

086

1.01

0.92

0.95

086

1.04

1.06

0 .88

1.01

1.02

086

1.05

0.81

0.75

088

08

0.76

0.95

084

0.97

1.16

0.99

11

09

1.05

0.89

0.86

0.98

0.98

0.86

089

1.28

1.35

0.52

**B CG Sites**

**enrichments**

*:;;*

8

0.4

1.9

0.6

0.9

0.7

0.8

1.2

o.s

o.s

1.1

1.S

1.1

0.7

0.9

l .S

1

1.8

0.6

0.7

0.9

1.3

1.7

2.2

1 .8

1

### **Supplementary Figure 42: Full-stack states enrichments with variants from GNOMAD stratified by minor allele frequencies, common variants (A) and CG dinucleotides (B**). In each subpanel, each row corresponds to a full-stack state. The first column gives the state labels, the second gives the percent of the genome that each state covers. The heatmap colors are on a column specific coloring scale. The last row shows the percentage of the genome that each group of variants occupy. **(A)** The last column shows the enrichment of full-stack states with common variants from UCSC Genome Browser’ snp151 track (Methods). Other columns show fold enrichments of full-stack states for GNOMAD variants with the specified ranges of MAF, which are ordered in increasing MAF (Karczewski et al., 2020). **(B)** The last column shows fold enrichment of full-stack states with CG dinucleotides**.** The three states showing highest enrichment if variants of lowest MAF (0 < MAF <= 0.0001) (TSS1-2, PromF4) are also the states most enriched states with CG dinucleotide sites, likely reflecting the higher mutation rates associated with CG dinucleotide sites (Karczewski et al., 2020).

Line 424-445 and FigS39: why are the H3K9me3 states and GapArtf states so enriched in cancer- associated variants, much higher than enhancer/promoter states? Any biological reasons? One would think that states that are of regulatory functions (e.g., enhancers and promoters) should be enriched in cancer-associated variants.

We thank the reviewer for the questions. If the analysis was specifically focused on enrichment of driving non-coding mutations relative to all mutations then we agree with the expectation to see enrichment of those mutations in enhancers and promoters. However, for this analysis, we focused only on the enrichment of all identified somatic mutations, given the lack of a large set of verified driving non-coding cancer mutations. The enrichment in cancer mutations in regions associated with H3K9me3 is consistent with previous observation in the literature on this: (1) *Schuster-Böckler and Lehner, 2012* [31] *showed that H3K9me3 signal is the feature showing highest correlation with single-nucleotide variant (SNV) density at the megabase-scale; (2) Parker et al., 2012 [32] showed that somatic mutations in melanoma is enriched in Heterochromatin-low-signal state across all nine human cell types; (3) Polak et al., 2015 [33] analyzed 173 cancer genomes of 8 cancer types and showed that H3K9me3 was consistently most predictive of local mutation density in different cancer types.* One explanation for this is less DNA mismatch repair occurring in closed chromatin regions [34,35]. We suspect the enrichment in the GapArtf states is driven by false variant calls because these regions of the genome are prone to likely sequence mapping errors.

In response to this comment, we have revised the text to now state:

This pattern of H3K9me3-associated states being enriched with somatic mutations in cancer is previously confirmed in multiple studies where H3K9me3 and other repressive epigenetic features showed positive association with mutation density across different types of cancer cells [31–33]. One possible explanation for this association is the more limited access of DNA mismatch repair machinery in these regions due to the tightly packed nature of the genome in heterochromatin [34,35]. Notably, the GapArtf2- 3 states, which had strong satellite repeats enrichments (**Fig. 4C, Supp. Fig. 28**) were the top two most enriched states with somatic variants associated with liver, pancreas and haematopoietic and lymphoid tissue (haem-lymphoid) cancers (2.0-5.6 folds enriched) (**Fig. 5F, Supp. Fig. 47**). We suspect that the enrichments in these putative alignment artifact states are driven at least in part by false variant calls due to sequence mapping errors associated with these regions. Similarly, enrichments of somatic mutations in haem-lymphoid cancer in state Acet1 is also suggestive of the possibility of false calls given this state’s

combination of H3K9me3 and acetylation signal and enrichment for simple repeats (**Fig. 2, 4C, Supp. Fig. 42**).

In addition to the revisions described above in response to specific questions of the reviewer about the biological significance of our enrichment results, we also expanded our discussion of the GWAS depletions to note Quies5 and ReprPC8 are highly specific to chrX. We extended **Supp. Fig 43** to now show the autosomal, chrX, and chrY enrichment of each state.

We have modified the main text to state:

“On the other hand, four states were more than two-fold depleted for GWAS catalog variants, and were associated with artifacts (GapArtf2-3), or quiescent and polycomb repressed states with weak signals of H3K9me3 (Quies5) or H3K27me3 (ReprPC8) (**Supp. Fig. 43**). Both Quies5 and ReprPC8 are highly specific to chrX, 18.3 and 19.1 fold enriched respectively (**Supp. Fig. 43**).”

The updated supplementary figure is shown below:

background: genome

background: common snps

background: genome

| state | % b a c k g ro u n d | g w a s _ c a ta lo g |
| --- | --- | --- |
| GapArtf1 | 11.861 | 0.254 |
| GapArtf2 | 0.050 | 0.267 |
| GapArtf3 | 0.012 | 0.279 |
| Quies1 | 9.883 | 0.737 |
| Quies2 | 3.070 | 0.793 |
| Quies3 | 12.226 | 1.098 |
| Quies4 | 4.452 | 0.803 |
| Quies5 | 1.692 | 0.180 |
| HET1 | 0.706 | 0.738 |
| HET2 | 0.692 | 1.021 |
| HET3 | 1.359 | 0.950 |
| HET4 | 0.563 | 0.949 |
| HET5 | 0.249 | 0.940 |
| HET6 | 0.581 | 0.723 |
| HET7 | 1.023 | 0.952 |
| HET8 | 0.435 | 1.228 |
| HET9 | 0.998 | 0.637 |
| ReprPC1 | 0.191 | 1.540 |
| ReprPC2 | 0.325 | 1.602 |
| ReprPC3 | 1.107 | 1.536 |
| ReprPC4 | 3.935 | 1.262 |
| ReprPC5 | 0.628 | 1.547 |
| ReprPC6 | 1.513 | 1.356 |
| ReprPC7 | 0.614 | 1.573 |
| ReprPC8 | 0.477 | 0.173 |
| ReprPC9 | 0.375 | 1.025 |
| Acet1 | 0.184 | 1.154 |
| Acet2 | 0.855 | 1.209 |
| Acet3 | 2.649 | 1.113 |
| Acet4 | 0.403 | 1.348 |
| Acet5 | 0.860 | 1.214 |
| Acet6 | 0.428 | 1.210 |
| Acet7 | 0.285 | 1.479 |
| Acet8 | 0.562 | 1.386 |
| EnhWk1 | 1.540 | 1.046 |
| EnhWk2 | 0.352 | 1.535 |
| EnhWk3 | 0.830 | 1.289 |
| EnhWk4 | 2.216 | 1.038 |
| EnhWk5 | 0.994 | 1.171 |
| EnhWk6 | 0.588 | 1.472 |
| EnhWk7 | 0.484 | 1.509 |
| EnhWk8 | 1.369 | 0.945 |
| EnhA1 | 0.179 | 1.605 |
| EnhA2 | 0.328 | 1.315 |
| EnhA3 | 0.194 | 1.478 |
| EnhA4 | 0.301 | 1.245 |
| EnhA5 | 0.714 | 1.158 |
| EnhA6 | 0.563 | 1.111 |
| EnhA7 | 0.393 | 1.647 |
| EnhA8 | 0.255 | 1.747 |
| EnhA9 | 0.162 | 1.568 |
| EnhA10 | 0.395 | 1.486 |
| EnhA11 | 0.715 | 1.217 |
| EnhA12 | 0.332 | 1.376 |
| EnhA13 | 0.764 | 1.149 |
| EnhA14 | 0.366 | 1.474 |
| EnhA15 | 1.017 | 1.214 |
| EnhA16 | 0.645 | 1.137 |
| EnhA17 | 0.526 | 1.215 |
| EnhA18 | 0.457 | 1.114 |
| EnhA19 | 0.257 | 1.372 |
| EnhA20 | 0.346 | 1.052 |
| TxEnh1 | 0.391 | 1.265 |
| TxEnh2 | 0.391 | 1.384 |
| TxEnh3 | 0.250 | 1.603 |
| TxEnh4 | 0.267 | 1.858 |
| TxEnh5 | 0.498 | 1.484 |
| TxEnh6 | 0.189 | 1.531 |
| TxEnh7 | 0.270 | 1.526 |
| TxEnh8 | 0.243 | 1.603 |
| TxWk1 | 2.800 | 1.152 |
| TxWk2 | 0.842 | 1.336 |
| Tx1 | 0.824 | 1.279 |
| Tx2 | 1.580 | 1.208 |
| Tx3 | 0.508 | 1.297 |
| Tx4 | 0.470 | 1.326 |
| Tx5 | 0.942 | 1.393 |
| Tx6 | 1.109 | 1.183 |
| Tx7 | 0.819 | 1.209 |
| Tx8 | 0.681 | 1.302 |
| TxEx1 | 0.265 | 1.600 |
| TxEx2 | 0.556 | 1.630 |
| TxEx3 | 0.663 | 1.606 |
| TxEx4 | 0.098 | 1.952 |
| znf1 | 0.406 | 1.247 |
| znf2 | 0.152 | 1.087 |
| DNase1 | 0.201 | 1.371 |
| BivProm1 | 0.145 | 1.760 |
| BivProm2 | 0.159 | 1.536 |
| BivProm3 | 0.286 | 1.483 |
| BivProm4 | 0.130 | 1.550 |
| PromF1 | 0.201 | 1.775 |
| PromF2 | 0.138 | 2.049 |
| PromF3 | 0.153 | 1.847 |
| PromF4 | 0.190 | 1.453 |
| PromF5 | 0.135 | 1.667 |
| PromF6 | 0.129 | 1.331 |
| PromF7 | 0.160 | 1.545 |
| TSS1 | 0.123 | 1.686 |
| TSS2 | 0.114 | 1.567 |
|  | 100 | 0.0029 |

| % b a c k g ro u n d | g w a s _ c a t a lo g b g :  u c s c _ s n p 1 5 1 _ c o m m o n |
| --- | --- |
| 3.449 | 0.873 |
| 0.125 | 0.107 |
| 0.031 | 0.106 |
| 11.671 | 0.624 |
| 3.586 | 0.679 |
| 14.755 | 0.910 |
| 5.259 | 0.679 |
| 1.323 | 0.230 |
| 0.874 | 0.597 |
| 0.964 | 0.733 |
| 1.397 | 0.925 |
| 0.683 | 0.784 |
| 0.325 | 0.721 |
| 0.808 | 0.520 |
| 1.405 | 0.693 |
| 0.508 | 1.050 |
| 1.195 | 0.531 |
| 0.188 | 1.565 |
| 0.334 | 1.560 |
| 1.124 | 1.513 |
| 4.385 | 1.132 |
| 0.746 | 1.302 |
| 1.818 | 1.129 |
| 0.739 | 1.305 |
| 0.314 | 0.263 |
| 0.412 | 0.933 |
| 0.386 | 0.552 |
| 0.936 | 1.104 |
| 3.035 | 0.971 |
| 0.443 | 1.224 |
| 0.934 | 1.117 |
| 0.435 | 1.190 |
| 0.298 | 1.415 |
| 0.661 | 1.179 |
| 1.561 | 1.033 |
| 0.377 | 1.435 |
| 0.844 | 1.267 |
| 2.209 | 1.042 |
| 1.114 | 1.045 |
| 0.612 | 1.414 |
| 0.540 | 1.354 |
| 1.419 | 0.912 |
| 0.170 | 1.689 |
| 0.307 | 1.407 |
| 0.166 | 1.725 |
| 0.275 | 1.361 |
| 0.671 | 1.233 |
| 0.537 | 1.164 |
| 0.454 | 1.425 |
| 0.272 | 1.633 |
| 0.151 | 1.678 |
| 0.389 | 1.509 |
| 0.807 | 1.078 |
| 0.347 | 1.316 |
| 0.795 | 1.105 |
| 0.385 | 1.401 |
| 1.097 | 1.126 |
| 0.715 | 1.026 |
| 0.477 | 1.338 |
| 0.526 | 0.969 |
| 0.260 | 1.354 |
| 0.346 | 1.054 |
| 0.335 | 1.476 |
| 0.325 | 1.666 |
| 0.230 | 1.741 |
| 0.229 | 2.164 |
| 0.502 | 1.472 |
| 0.174 | 1.661 |
| 0.257 | 1.606 |
| 0.210 | 1.856 |
| 2.899 | 1.113 |
| 0.893 | 1.260 |
| 0.728 | 1.447 |
| 1.602 | 1.192 |
| 0.517 | 1.275 |
| 0.402 | 1.548 |
| 0.989 | 1.326 |
| 0.899 | 1.459 |
| 0.617 | 1.606 |
| 0.599 | 1.480 |
| 0.212 | 2.005 |
| 0.424 | 2.137 |
| 0.633 | 1.683 |
| 0.083 | 2.314 |
| 0.407 | 1.242 |
| 0.147 | 1.119 |
| 0.232 | 1.187 |
| 0.144 | 1.776 |
| 0.159 | 1.539 |
| 0.315 | 1.343 |
| 0.117 | 1.718 |
| 0.212 | 1.684 |
| 0.123 | 2.301 |
| 0.132 | 2.137 |
| 0.187 | 1.477 |
| 0.133 | 1.694 |
| 0.111 | 1.551 |
| 0.143 | 1.732 |
| 0.158 | 1.315 |
| 0.154 | 1.160 |
| 100 | 0.5556 |

| a u t o s o m a l | c h r X | c h r Y |
| --- | --- | --- |
| 0.871 | 1.142 | 6.888 |
| 0.911 | 0.716 | 6.047 |
| 0.923 | 0.473 | 6.106 |
| 1.048 | 0.349 | 0.355 |
| 1.054 | 0.323 | 0.131 |
| 1.057 | 0.240 | 0.235 |
| 0.930 | 2.184 | 1.317 |
| 0.087 | 18.310 | 0.044 |
| 1.053 | 0.389 | 0.028 |
| 1.058 | 0.301 | 0.004 |
| 1.005 | 1.273 | 0.035 |
| 1.024 | 0.808 | 0.319 |
| 1.053 | 0.251 | 0.384 |
| 1.006 | 1.196 | 0.174 |
| 0.980 | 1.533 | 0.581 |
| 1.044 | 0.541 | 0.047 |
| 0.929 | 2.391 | 0.831 |
| 1.056 | 0.339 | 0.000 |
| 1.051 | 0.429 | 0.000 |
| 1.062 | 0.239 | 0.003 |
| 1.069 | 0.108 | 0.008 |
| 1.065 | 0.176 | 0.001 |
| 1.073 | 0.032 | 0.003 |
| 1.065 | 0.178 | 0.000 |
| 0.046 | 19.086 | 0.011 |
| 1.018 | 1.016 | 0.094 |
| 1.047 | 0.494 | 0.024 |
| 1.069 | 0.096 | 0.018 |
| 1.064 | 0.123 | 0.201 |
| 1.064 | 0.186 | 0.001 |
| 1.059 | 0.293 | 0.000 |
| 1.057 | 0.322 | 0.002 |
| 1.064 | 0.190 | 0.000 |
| 1.051 | 0.388 | 0.113 |
| 1.044 | 0.529 | 0.115 |
| 1.066 | 0.165 | 0.000 |
| 1.051 | 0.426 | 0.012 |
| 1.057 | 0.313 | 0.033 |
| 1.024 | 0.815 | 0.329 |
| 1.030 | 0.766 | 0.140 |
| 1.047 | 0.496 | 0.034 |
| 1.010 | 1.129 | 0.165 |
| 1.071 | 0.068 | 0.000 |
| 1.061 | 0.258 | 0.000 |
| 1.059 | 0.293 | 0.000 |
| 1.052 | 0.424 | 0.002 |
| 1.042 | 0.578 | 0.056 |
| 1.051 | 0.429 | 0.001 |
| 1.045 | 0.541 | 0.021 |
| 1.041 | 0.611 | 0.040 |
| 1.057 | 0.333 | 0.002 |
| 1.044 | 0.542 | 0.044 |
| 1.041 | 0.604 | 0.033 |
| 1.051 | 0.444 | 0.004 |
| 1.037 | 0.689 | 0.041 |
| 1.056 | 0.344 | 0.019 |
| 1.040 | 0.599 | 0.105 |
| 1.024 | 0.905 | 0.075 |
| 1.045 | 0.533 | 0.017 |
| 1.013 | 1.120 | 0.060 |
| 1.054 | 0.383 | 0.001 |
| 1.026 | 0.807 | 0.243 |
| 1.060 | 0.268 | 0.012 |
| 1.056 | 0.339 | 0.023 |
| 1.063 | 0.214 | 0.005 |
| 1.069 | 0.102 | 0.000 |
| 1.061 | 0.245 | 0.012 |
| 1.058 | 0.293 | 0.012 |
| 1.070 | 0.077 | 0.000 |
| 1.066 | 0.151 | 0.000 |
| 1.029 | 0.766 | 0.215 |
| 1.039 | 0.649 | 0.028 |
| 1.049 | 0.448 | 0.060 |
| 1.035 | 0.673 | 0.144 |
| 1.058 | 0.300 | 0.019 |
| 1.054 | 0.349 | 0.098 |
| 1.044 | 0.538 | 0.097 |
| 1.038 | 0.647 | 0.067 |
| 1.061 | 0.255 | 0.000 |
| 1.055 | 0.343 | 0.041 |
| 1.064 | 0.190 | 0.000 |
| 1.066 | 0.152 | 0.000 |
| 1.052 | 0.410 | 0.002 |
| 1.065 | 0.181 | 0.000 |
| 1.039 | 0.623 | 0.072 |
| 1.063 | 0.212 | 0.000 |
| 1.032 | 0.768 | 0.037 |
| 1.054 | 0.388 | 0.002 |
| 1.051 | 0.434 | 0.002 |
| 1.045 | 0.525 | 0.040 |
| 1.032 | 0.777 | 0.021 |
| 1.057 | 0.333 | 0.002 |
| 1.058 | 0.313 | 0.002 |
| 1.063 | 0.220 | 0.000 |
| 1.046 | 0.536 | 0.000 |
| 1.043 | 0.589 | 0.002 |
| 1.043 | 0.581 | 0.000 |
| 1.038 | 0.665 | 0.029 |
| 1.011 | 1.162 | 0.033 |
| 0.991 | 1.375 | 0.450 |
| 93.07 | 5.02 | 1.92 |

**Supplementary Figure 43: Full-stack states enrichments with GWAS catalog variants (Welter et al., 2014) and sex chromosomes.** Each row corresponds to a full-stack state. The first column gives the state labels and the second column shows the percentage in the genome that each full-stack state occupies. The third column shows the fold enrichments of full-stack states with GWAS catalog variants against the whole- genome. The fourth column shows the percentage of the background context (UCSC snp151 common variants) that each full-stack state occupies. The fifth column shows fold enrichments with GWAS catalog variants against the background of common variants (**Methods**). The sixth, seventh and eight columns report the autosomal, chrX, and chrY enrichments, respectively. Columns are colored on a column specific coloring scale. The last row reports the percent of the background context (whole genome and set of common variants) that the GWAS catalog variants cover.

We also present below three supplementary figures that we have used to further characterize the full-stack states’ biological significance. The first shows the full-stack state varying levels of DNA methylation.

The second and third show the full-stack enrichment for overlapping binding regions with proteins that are part of the PRC1 and PRC2 binding complex, and enrichment of states with regions surrounding such bindings.

**Average DNA methylation in different cell types**

**Cell groups State groups**

**Supplementary Figure 15: Full-stack states’ average DNA methylation in different cell types.** Each row corresponds to one of the 100 full-stacked states grouped into state groups as indicated by the legend at the bottom. Each column corresponds to one cell type whose DNA methylation

| **Blood & T-cell** | **H SC & B-cell** | **Neurosph** | **Brain** | **Thym us** | **Adipose** | **Epithelial** | **Myosat** | **Muscle** | **Sm . Muscle** |
| --- | --- | --- | --- | --- | --- | --- | --- | --- | --- |
|  | **IMR90** | **Mesench** | **iPSC** | **ESC** | **D igestive** | **ES-deriv** | **ENCODE2012** | **Other** |  |

| **Assembly Gaps**  **& artifacts** | **quiescent** | **HET** | **polycomb repressed** | **acetylations** | **weak**  **enhancers** | **enhancers** | **transcribed & enhancer** | **weak**  **transcription** | **transcription** | **exon &**  **transcription** | **ZNF genes** | **DNase** | **bivalent promoter** | **flanking promoter** | **TSS** |
| --- | --- | --- | --- | --- | --- | --- | --- | --- | --- | --- | --- | --- | --- | --- | --- |

### data were available from Roadmap Epigenomics. The columns are grouped based on their associated tissue group as indicated by the legend at the bottom. Each column shows the average DNA methylation level in the respective cell type that overlaps with each full-stack state (**Methods**). Among promoters-associated states, those most enriched with CpG islands also show lowest average DNA methylation levels (**Fig. 3A**), consistent with expectation (Weber et al., 2007, Jones et al., 2001).

**mammary_epithelial_cell**

**skeletal_muscle_myoblast**

**endothelial_cell_of_umbilical_vein**

**BMI1**

**State**

**% genome**

**GM12878**

**K562**

**MCF-7**

**K562**

**K562**

**K562**

**K562**

**A673**

**B_cell**

**DOHH2**

**GM12878**

**HCT116**

**HeLa-S3**

**PC-3**

**PC-9**

**PC-9**

**SK-N-SH**

**SU-DHL-6**

**bipolar_neuron**

**fibroblast_of_dermis**

**fibroblast_of_lung**

**neural_progenitor_cell**

**K562**

**RING1**

**RNF2**

**A549**

**H1**

**HepG2**

**K562**

**EZH2**

**HepG2**

**Loucy**

**MM.1S**

**MM.1S**

**OCI-LY3**

**OCI-LY7**

**SUZ12**

**H1**

**H1**

**HEK293T**

**HepG2**

**K562**

**EED**

**MCF-7**

**NT2**

**GM12878**

**GM12878**

**GM12892**

**HepG2**

**K562**

**K562**

GapArtf1 11.86	0.003	0	0.009

0	0.006	2E-04	0.002	0.043	0.007	2E-04	0

3E-04	0	0	0	0	4E-04	0	0.001	0	0	0	0.011	0.015	0.013	0.009	5E-04	0.009	0.008	7E-04	0	0	0.001	0

0 5E-04	0.115	0	0	0	0.017	6E-04 2E-04	0	0	0.001	0	0.002	0

GapArtf2

0.05	0.487	0.778	1.128	0.533	0.838	3.745	1.053	0.534	0.457	0.734	0.533

0.138	0.899	0.351	0 3.784	0.244	0.214	3.99	1.348	0.061	1.237	0	0.391	3.536	4.279	3.991	0	0.332	0.135	0.334	0.15	0.178	4.801	2.614	0	26.22	1.963	0.431	1.664	1.891	2.031	0.438	0.417	0.999	0.716	0.358	1.217	0.118

GapArtf3

0.01	1.136	16.25	9.154	16.48	12.48	15.52	4.103	1.39	1.127	1.428	16.48

3.939	3.508	0.205	3.073	34.87

2.787 25.75	0	0	0	0	5.486	17.14	19.77	61.6	17.51	2.261	4.416	5.631	7.856	5.281	0.124	18.61	1.995	27.88	32.4	13.87	42.17	14.97	11.32	2.478	9.118	12	2.424	13.11	2.005	3.184

Quies1 Quies2 Quies3 Quies4

9.88	0.084	0.002	0.095	1E-04	0.066	6E-04	0.001	0.039	0.008	9E-04 1E-04

3.07	0.186	0.026	0.278	8E-05	0.056	0.002	0.016	0.055	0.026	0.013	8E-05

12.23	0.116	0.102	0.202	0.017	0.244	8E-05	0.016	0.794	0.168	0.031	0.017

4.45 0.05	0.026	0.11	0.009	0.147	0	0.003	0.265	0.049	0.008	0.009

0.035	0.004	0.003	0	0.013	0.086	0.001	0.163	0.041	0.005	0.208	0.193	0.156	0.143	0.106	0.023	0.075	0.092	0.002	3E-04	9E-05	0.06	0	0.002	9E-04	2.285	0.003	0.003	3E-04	0.064	0	0.013	8E-05	0	0.005	1E-04	0.002	0

0.043	0.008	0.004	0	0.133	0.448	0.026	0.144	0.018	0.005	0.27	0.001	0.105	0.721	0.607	9E-04	0.075	0.145	1E-03 3E-04	0.016	0.163	0	0.027	0.003	0.738	0.015	0.044	0.001	0.23	0	0.071	0.006	0	0.042	0	0.019	0

8.591

0.092	0.01	0.015	0.003	0.147	0.186	0.017	0.263	0.151	0.035	0.281	0.119	0.294	0.254	0.241	0.107	0.122	0.086	0.004	0.002	0.001	0.06	0.015	9E-04	0.006	0.686	0.006	0.035	0.001	0.289	0.001	0.039	0.004	1E-03	0.058	0.004	0.04 8E-04

0.033	0.009	6E-04	0	0.09	0.058	8E-04	0.236	0.087	0.026	0.125	0.041	0.168	0.22	0.197	0.033	0.041	0.034	0.002	0	0	0.021	0	0.003	0.003	0.813	0	0	0.002	0.225	3E-04	0.009	0.003	1E-04	0.037	0	0.019	0

Quies5

1.69	0.063	0.043	0.072	0.017	0.155	0.006	0.005	0.312	0.064	0.01	0.017

0.036	0	0	0	0.037	0.213	0.004	0.049	0	0.021	0.136	0	0.027	0.098	0.127	0	0	0.087	0.006	0.002	7E-04	0.072	0

0	0.009	0.925	0	0	0	0.064	0.005	0.016	0	0	0.025	0	0.016	0

HET1

11.15

0.71	0.216	0	0.83	0.051	0.01	0.032	0.045	0.092	0.15	0.167	0.051

0.064	0.026	0	0	0	0.352	9E-04	0.127	0.042	0.033	0.224	0	0.072	0.809	0.943	0	0.043	0.171	0	0	0.011	0.194	0	0.535	0.013	0.674	0.012	0.078	0.01	0.779	0	0.107	0.02	0	0.342	0.015	0.228	0

HET2

0.69	0.109	0.215	1.189	0.115	0.442	0.052	0.208	0.217	0.091	0.105	0.115

0.85	0	0.03	0 3.726

2.286	0.049	0.223	2.31	0.236	0	1.026	18.52	20.2	0.198	0.408	0.323	0	0.007	0.05	0.235	0	0.868	0.081	0.848	0.083	3.817	0.335	0.998	0.016	0.136	0.37	0.004	0.224	0.041	0.141	0

HET3

1.36 0.16	0.065	0.253	0.014	0.416	0	0.007	0.447	0.086	0.024	0.014

0.03	0	0.019	0	0.309	0.175	1E-04	0.373	0.489	0.076	0.538	0.088	0.206	0.206	0.296	0.094	0.245	0.029	0.009	0	0	0.038	0

0	0.004	0.805	0	0	0.033	0.349	0.002	0.038	0.012	0	0.098	0	0.07	0

HET4

0.56	0.125	0.001	0.459

0	0.15	0.006	0.013	0.244	0.17	0.068	0

0.037	0.032	0.071	0	0	0.047	0.006	0.326	0.503	0.208	0.243	0	0.141	0.19	0.243	0.412	0.108	0.03	0	0	0	0.029	0.231	0.059	0.013	1.686	0	0	0.046	0.413	0.005	0.02	0	0.012	0.085	0.001	0.218	0

HET5 HET6

0.25	0.163	0.154	0.993	0.184	0.105	0.434	0.467	0.146	0.428	0.427	0.184

0.58	0.072	0	0.411	0.022	0.11	0.155	0.053	0.079	0.132	0.057	0.022

0.004	0	0.041	0	0	0.363	0.213	0.431	0.428	0.234	0.25	0	0.148	1.828	1.159	0.638	0.127	0.09	0.115	0.139	0.212	0.051	0.356	1.136	0.02	1.073	0.324	0.501	1.653	1.903	0.406	0.232	0.192	0.119	0.655	0.375	0.872	0

0.01	0	0	0	0	0.25	0.02	0.017	9E-04	0.204	0	0	0.033	2.462	2.021	0.277	0.066	0.061	0	0	0.028	0.003	0	0.359	0.009	0.144	0.033	0.4	0.268	0.417	0.092	0.045	0.004	0	0.243	0.078	0.149	0

HET7

1.02	0.064	0.04	0.363

0	0.057	0.004	0.057	0.165	0.121	0.049	0

0.026	0	0.015	0	0.136	0.277	0	0.098	0.01	0.101	0.005	0.039	0.08	0.729	0.813	0.019	0	0.041	0.005	0	0.017	0.022	0	0.053	0.011	0.161	0.044	0.12	0.076	0.652	0.025	0.055	0.011	0	0.324	0.004	0.101	0

HET8

0.43	0.547	0.395	1.835	0.343	0.782	0.091	0.512	1.055	0.873	0.703	0.343

0.396	0	0.117	0.198	1.543	1.714	0.434	0.825	0.83	1.918	1.51	0.41	0.588	2.337	2.523	0.381	0.751	0.348	0.092	0.244	0.132	0.187	0.057	0.194	0.111	1.064	0.944	0.377	2.634	2.959	0.295	0.679	0.508	0.069	1.936	0.836	1.121	0.02

HET9

1.00	0.177	0.067	0.264

0	0.339	0.025	0.015	0.279	0.096	0.032	0

0.063	0	0	0.023	0	0.32	0.01	0.201	0.069	0.171	0.29	0	0.203	1.305	1.418	0.134	0.04	0.022	0	0	0	0.029	0.021	0.025	0.002	0.696	0	0.009	0.02	0.268	0.027	0.041	0.004	0.01	0.081	0.028	0.104	0

ReprPC1 ReprPC2 ReprPC3

0.19	2.183	6.851	2.451	4.093	12.31	120	2.617	9.961	2.303	1.782	4.093

0.32	1.096	6.178	2.06	2.081	7.823	8.509	0.644	8.247	1.816	0.965	2.081

1.11 0.33	2.091	1.222	0.728	4.337	0.015	0.166	5.109	1.159	0.619	0.728

75.28	43.74	24.7	58.44	14.02	9.626	50.09 0.734 33.45 39.09	4.03	32.59	12.25	11.6	12.44	84.89	8.751	74.98	69.12 90.61	107.6	74.3	90.43	108.9	99.97	3.55	1.234	28.55	3.192	2.752	100.3	0.527	0.708	0.114	0.623	1.311	1.601	0.053

10.76	2.292	5.04	2.982	15.67	7.351	5.182	1.205	5.65	7.799	3.877	8.419	2.387	4.557	5.397	8.132	4.804	22.92	8.305	10.11	5.849	8.735	2.548	1.265	10.1	0.564	0.113	4.764	0.615	1.998	8.271	0.389	0.127	0.078	0.442	1.316	0.803	0.024

2.025	0.019	0.529	0.091	11.99	10.38	0.954	0.395	2.142	2.139	3.341	1.579	1.373	3.345	3.578	0.596	2.87	0.654	0.584	1.3	0.496	0.413	0.138	0.102	0.15	0.795	0.118	0.721	0.251	1.431	0.026	0.23	0.036	0.018	0.437	0.165	0.416	4E-04

ReprPC4 ReprPC5 ReprPC6 ReprPC7

3.93	0.107	0.52	0.39	0.176	2.036	0.005	0.041	2.549	0.355	0.111	0.176

0.63	0.283	0.826	2.103	1.066	0.868	0.206	0.657	1.497	0.707	0.685	1.066

1.51	0.142	0.217	1.12	0.097	0.346	0.054	0.19	1.009	0.249	0.121	0.097

0.61	0.397	0.507	2.274	1.063	0.128	0.109	2.236	0.469	0.664	0.478	1.063

0.483	0	0.065	0.015	3.212	2.473	0.189	0.261	0.491	0.386	0.957	0.46	0.722	1.622	1.377	0.188	0.832	0.181	0.049	0.081	0.038	0.111	0.01	0.006	0.03	0.431	0.016	0.226	0.021	0.613	0.005	0.044	0.004	0.006	0.061	0.005	0.037	0

3.342	0.039	0.523	0.054	10.57	19.09	10.19	0.432	7.946	12.38	1.121	0.478	1.949	13.2	14.17	2.41	4.829	0.75	2.653	4.601	2.457	0.356	0.324	1.82	0.537	0.638	0.544	8.035	1.218	2.39	0.168	0.377	0.489	0.09	0.469	1.117	0.556	0

0.352	0	0.124	0	2.533	4.57	1.512	0.26	1.1	2.509	0.361	0.091	0.438	3.717	3.776	0.213	0.94	0.22	0.206	0.522	0.142	0.144	0	0.224	0.111	0.2	0.11	2.337	0.646	1.605	0.037	0.137	0.139	0.016	0.283	0.206	0.106	0

0.544	0	0.325	0 0.549	4.64	5.624	0.089	2.88 9.089 0.205	0.131	1.274	5.389	5.903	0.255	2.453	0.612	1.155	2.067	0.717	0.434	0.44	0.752	0.246	0.215	0.346	9.596	1.88	1.489	0.05	0.266	0.426	0.023	2.002	1.584	0.55	0.036

ReprPC8 ReprPC9 Acet1 Acet2 Acet3 Acet4 Acet5

0.48	0.136	0.192	0.461	0.056	0.306	0	0.009	0.411	0.302	0.246	0.056

0.37	0.325	0.992	0.789	0.217	1.66	1.521	0.239	3.158	0.898	0.503	0.217

0.18	0.923	0.303	14.29	1.058	0.077	1.744	1.485	1.296	1.165	1.563	1.058

0.85 0.32	0.351	1.334	0.366	0.155	0.045	0.15	0.759	0.592	0.405	0.366

2.65	0.129	0.113	0.407	0.036	0.141	0.005	0.029	0.633	0.152	0.049	0.036

0.40	0.684	0.579	1.426	0.908	0.141	0.111	0.993	1.124	1.691	1.423	0.908

0.86 0.32	0.199	0.462	0.121	0.13	0.005	0.079	0.635	0.413	0.143	0.121

0.074	0.021	0	0	0.181	1.098	0.077	0.172	0	0	0.096	0	0.091	0.326	0.249	0	0	0.083	0.017	0.052	0.038	0.124	0	0.047	0.011	1.349	0	0	0.052	0.521	0.013	0.098	0.016	0	0.185	0.338	0.284	0

1.154	0.061	0.342	0.135	3.007	1.455	0.646	0.155	0.35	0.436	1.453	1.09	0.446	1.239	0.97	0.882	1.232	4.394	0.675	0.704	0.277	2.199	0.092	0.337	3.006	0.641	0	0.965	0.375	1.227	2.363	0.188	0.05	0.045	0.557	0.981	0.495	0.025

0.103	0.004	0.124	1.138	1.315	3.245	1.29	0	2.01	1.218	0.582	0	0.391	2.615	2.562	0.42	0.554	0.588	0.31	0.914	2.222	0.253	0.684	76.12	0.392 8.968	1.958	2.661	3.336	35.9	0.122	0.748	2.219	0.189	2.296	1.851	2.806	0.055

0.179	0	0.015	0.086	1.129	1.457	0.137	0.526	0.327	0.035	0.441	0	0.284	0.38	0.32	0	0.679	0.248	0.007	0.056	0.18	0.261	0	0.851	0.025	0.503	0.139	0.319	0.45	2.093	0.005	0.398	0.186	0.054	1.307	0.61	0.579	0

0.055	0	0.02	0	0.308	0.385	0.031	0.188	0.183	0.018	0.266	0.032	0.095	0.263	0.246	0.04	0.245	0.102	0.003	0.007	0.013	0.076	0.019	0.069	0.01	0.313	0	0.069	0.025	0.781	0	0.087	0.019	0.006	0.179	0.013	0.05	0.003

0.128	0	0.652	0.272	0.642	0.943	0.911	1.019	0.643	1.744	0.631	0.038	0.187	0.486	0.414	0.024	1.87	0.296	0.039	0.249	0.378	0.374	0	0.708	0.167	0.379	0.11	2.642	0.574	2.472	0	1.387	0.515	0.197	3.537	2.152	1.914	0.132

0.177	0	0.103	0.002	0.288	0.292	0.163	0.68	0.15	0.137	0.843	0.298	0.183	0.102	0.117	0.05	0.709	0.143	0.017	0.024	0.031	0.155	0	0.01	0.034	0.476	0.086	0.121	0.163	0.789	0	0.343	0.042	0.026	0.63	0.054	0.23	0.014

Acet6

0.43	0.964	0.865	0.866	0.352	0.227	0.013	0.161	1.169	1.307	0.758	0.352

0.193	0	0.427	0.689	1.605	0.398	0.173	1.175	0	0.212	0.885	0.184	0.452	0.2	0.06	0.273	0.774	0.225	0.024	0.048	0.119	0.274	0.231

0	0.038	0.753	1E-03	0.352	0.097	0.612	0	0.913	0.09	0.055	1.513	0.571	0.796	0.197

Acet7

0.28	1.882	2.832	4.032	3.652

0	0.09	3.958	1.436	5.595 4.801	3.652

0.19	0	0.289	0.196	1.181	0.626	1.429	1.174	2.972	2.784	0.848	0.663	5.211	0.566	0.206	0	2.502	0.666	0.084	0.391	0.493	0.645	0.018	0.316	0.304	0	1.291	1.963	1.145	3.129	0.056	3.428	0.873	0.799	9.661	5.88	5.53	1.772

Acet8

0.56	0.368	0.236	1.105	0.258	0.146	0.057	0.357	0.59	0.832	0.628	0.258

0.174	0	0.074	0	0.725	1.064	0.276	0.101	1.038	0.328	0.347	0.152	0.42	0.323	0.377	0.06	0.479	0.212	0.039	0.244	0.094	0.23	0	0.052	0.039	0	0.21	0.857	0.285	1.427	0	0.449	0.24	0.019	1.186	0.664	0.661	0.019

EnhWk1

1.54	0.506	0.289	0.348	0.072	0.428	0.002	0.03	0.777	0.262	0.086	0.072

0.211	0	0.097	0.035	0.837	0.298	0.054	1.215	0.24	0.056	0.787	0.167	0.524	0.127	0.132	0.321	0.512	0.248	0.006	0.006	0.005	0.106	0.107

0	0.022	0.754	0.023	0.07	0.01	0.3	0	0.172	0.006	0.003	0.173	0.011	0.065	0.008

EnhWk2 EnhWk3 EnhWk4 EnhWk5

0.35	1.528	1.319	3.442	1.749	0.039	0.237	3.862	0.447	1.771	1.999	1.749

0.83	0.505	0.81	1.143	0.427	0.334	0.014	0.257	1.26	0.753	0.559	0.427

2.22	0.415	0.282	1.106	0.057	0.418	0.002	0.031	0.616	0.143	0.058	0.057

0.99	0.804	0.219	0.542	0.143	0.541	0.008	0.064	1.196	0.545	0.328	0.143

0.246	0.116	0.828	0.254	0.224	2.408	2.539	0.98	2.857	4.228	1.55	0.123	0.924	1.428	1.501	0.076	2.881	0.912	0.666	2.323	0.971	0.488	0.271	0.68	0.427	0.089	0.722	3.93	1.12	1.319	0.132	1.975	1.177	0.517	5.123	4.972	1.983	0.74

0.328	0.03	0.213	0.01	2.167	2.788	0.579	1.276	0.781	0.495	0.778	0.473	0.751	1.613	1.17	0.019	1.344	0.672	0.146	0.449	0.206	0.315	0.269	0.114	0.102	0.099	0.092	0.982	0.125	0.67	0.015	0.526	0.229	0.066	1.054	0.434	0.499	0.059

0.262	0	0.041	0.009	1.913	1.027	0.051	0.367	0.114	0.111	0.835	0.117	0.464	0.578	0.646	0.056	0.533	0.487	0.012	0.026	0.023	0.484	0.054	0.025	0.017	0.664	0.002	0.123	0.026	0.226	0	0.094	0.003	0.002	0.11	0.01	0.039	0.009

0.106	0	0.146	0.125	0.93	0.71	0.065	8.21	1.115	0.168	1.478	0.302	0.288	0.561	0.587	0.046	0.216	0.146	0.058	0.043	0.014	0.142	0.016	0.026	0.02	0.689	0	0.209	0.006	0.589	0	0.384	0.081	0.007	0.145	0.137	0.346	0.006

EnhWk6

0.59 6.16	0.947	0.556

0.6	0.747	0.01	0.18 1.746	1.372	1.139	0.6

0.185	0	0.503	2.699	0.867	1.062	0.151	15.67	4.766	0.149	8.012	0.776	0.151	0.433	0.404	0.151	1.679	0.167	0.038	0.098	0.01	0.128	0.1	0.091	0.031	0.366	0	0.332	0.127	0.787	0	4.644	1.096	0.558	0.469	0.756	1.137	0.018

EnhWk7

0.48	17.33	0.529	0.476	0.17	0.198	0.007	0.084	1.592	0.581	0.205	0.17

0.053	0	1.204	2.693	1.282	0.732	0.003	1.385	5.476	0.022	16.15	0.613	0.109	0.19	0.143	0	1.439	0.207	0.066	0.061	0.025	0.184	0.089	0.034	0.042	0.064	0	0.036	0.061	0.6	0	13.62	2.672	1.335	0.372	0.117	0.217	0.006

EnhWk8 EnhA1 EnhA2 EnhA3 EnhA4 EnhA5 EnhA6 EnhA7 EnhA8 EnhA9 EnhA10 EnhA11 EnhA12 EnhA13 EnhA14 EnhA15 EnhA16 EnhA17 EnhA18 EnhA19

1.37	0.454	0.333	0.502	0.064	0.63	0.009	0.023	0.768	0.252	0.089	0.064

0.18	4.593	6.156	8.782	10.35	0.828	1.404	13.58	2.726	10.4	11.92	10.35

0.33	2.418	2.492	3.891	1.354	0.254	0.071	1.683	2.912	2.803	2.178	1.354

0.19	20.03	15.32	10.27	10.33	0.484	0.063	4.636	4.826	16.46	15.39	10.33

0.30	5.115	2.769	3.894	1.531	0.084	0.031	1.155	2.295	7.032	4.321	1.531

0.71	2.695	0.972	1.822	0.554	0.398	0.002	0.344	1.463	2.304	1.187	0.554

0.56	1.032	0.425	2.283	0.273	0.722	0.029	0.285	1.221	0.571	0.326	0.273

0.39	7.696	2.057	1.833	2.327	0.632	0.318	1.138	2.13	2.922	3.485	2.327

0.25	22.87	3.565	2.199	4.686	1.325	0.559	1.434	3.246	5.482	8.409	4.686

0.16	22.26	5.868	4.315	11.13	0.093	0.442	4.385	3.212	9.868	15.26	11.13

0.39	15.95	3.125	2.185	2.918	0.866	0.158	1.25	2.981	8.298	7.85	2.918

0.72	3.289	0.714	0.647	0.283	0.642	0	0.125	1.44	1.37	0.882	0.283

0.33	1.923	0.994	4.782	1.928	0.258	0.078	1.67	1.322	3.441	2.786	1.928

0.76 0.88	0.45	1.132	0.113	0.415	0	0.073	0.834	0.529	0.181	0.113

0.37	1.912	1.814	3.591	2.495	0.433	0.084	8.398	1.311 4.538	3.895	2.495

1.02	0.492	0.237	0.778	0.108	0.304	0	1.31	1.057	0.689	0.266	0.108

0.65	0.732	2.523	1.536	2.18	0.328	0.01	0.535	3.178 10.72	7.645	2.18

0.53	2.627	2.136	3.238	0.631	0.597	0.045	0.408	1.835	1.763	1.328	0.631

0.46 0.67	0.997	1.185	0.257	0.703	0.143	0.246	1.655	1.15	0.746	0.257

0.26 3.04	3.465	5.195	2.997	0.347	0.552	3.424	2.426	4.11	4.777	2.997

0.238	0	0.104	0	1.254	0.886	0.078	0.109	0.408	0.113	0.644	0.167	0.552	0.644	0.575	0.114	0.391	0.499	0.01	0.011	0.02	2.004	0.003	0.015	0.026	0.537	0.021	0.094	0.056	0.177	0.009	0.119	0.026	0.006	0.118	0.013	0.076	0

0.218	0.25	1.662	1.418	0	0.56	2.962	1.83	3.617	3.673	2.26	1.45 11.81	0.315	0.279	0.025	3.588	2.297	0.377	0.447	0.835	2.579	0.272	1.62	1.757	0.121	4.111	2.928	4.374	4.405	0.751	8.42	2.969	2.606	19.03	15.42	13.3	8.507

0.522	0	0.438	0.698	2.515	2.675	1.668	3.227	2.577	0.945	1.136	0.957	1.501	1.329	1.151	0	2.65	1.477	0.398	0.88	0.592	1.424	0.529	0.082	0.261	0.72	0.613	2.645	0.469	0.562	0.032	1.706	0.088	0.091	5.699	1.297	1.858	0.379

0.24	0	2.321	7.969	0.407	0.168	0.736	18.51	6.051	0.974	13.44	0.92	6.218	0.293	0	0.456	2.709	1.012	0.127	0.073	0.105	1.456	0.235	0.092	0.336	2.401	3.68	1.19	5.42	2.497	0.156	17.94	2.884	3.393	16.9 9.459 16.07	3.701

0.168	0	0.508	1.767	1.788	0.217	0.334	5.946	1.038	0.215	5.005	1.155	3.275	0.054	0	0.378	1.47	0.467	0.067	0.115	0.2	0.829	0.233	0.083	0.111	0.543	0.024	0.413	0.773	0.734	0.023	4.354	0.272	0.243	8.549	1.887	4.937	1.36

0.215	0	0.305	0.63	1.765	0.612	0.131	5.091	1.436	0.073	3.313	0.479	1.136	0.265	0.238	0.447	1.248	0.372	0.018	0.033	0.055	0.297	0.304	0.016	0.041	0.69	0.137	0.204	0.255	0.566	0.002	1.656	0.07	0.054	2.566	0.748	1.403	0.19

0.411	0	0.195	0.097	3.154	3.773	0.699	0.556	0.863	0.876	1.453	0.183	0.593	2.316	2.998	0.295	0.933	0.616	0.144	0.34	0.229	0.967	0.139	0.293	0.139	0.656	0	1.425	0.922	0.807	0.095	0.499	0.107	0.045	1.07	0.554	0.337	0.018

0.466	0.094	2.301	3.387	3.19	4.726	2.497	11.87	4.522	2.985	9.673	0.964	0.436	3.131	2.762	1.106	5.12	0.849	1.112	1.641	0.408	0.484	0.006	1.004	0.388	0	0.487	3.757	1.434	1.876	0.262	11.09	7.236	3.452	1.631	4.885	3.161	0.174

0.576	0.031	5.12	11.86	1.322	1.841	1.753	22.75	15.78	1.023	20.72	1.885	1.118	2.3	1.225	0.405	7.76	1.798	1.08	1.92	0.842	0.856	0.626	0.39	1.031	0.286	0.52	2.222	1.459	1.261	0.591	26.37	18.79	16.63	2.508	6.81	6.386	0.759

0	0	4.623	10.62	0.804	0.108	0.838	21.52	9.001	0.267	14.7	1.605	4.123	0.319	0	0.475	5.477	0.927	0.305	0.441	0.279	0.926	0	0.76	0.514	0.513	1.62	0.586	5.228	2.507	0.126	35.36	24.69 20.79	8.408	18.21	14.46	3.025

0.221	0.094	2.413	7.412	0.227	0.456	0.549	21.19	7.254	0.272	17.86	1.428	1.574	0.096	0.011	0.068	3.011	0.706	0.144	0.173	0.105	0.549	0.051	0.018	0.253	0.43	0.382	0.402	1.463	1.736	0.088	14.63	3.284	2.962	4.517	4.79 8.235	0.431

0.092	0	0.24	0.51	0.847	0.701	0.113	2.326	1.623	0.156	4.16	1.027	0.225	0.854	0.572	0.277	0.732	0.183	0.017	0.063	0.037	0.129	0.075	0.023	0.051	0.063	0.048	0.203	0.146	0.824	0	2.448	0.108	0.091	0.903	0.402	0.932	0.018

0.209	0	0.732	0.118	0.626	1.135	0.872	2.075	0.618	0.809	2.07 0.188	4.265	0.839	1.105	0.099	2.342	0.427	0.173	0.646	0.361	0.394	0	0.196	0.287	0.492	0.386	2.534	0.809	2.517	0.09	2.478	0.963	0.248	4.454	2.988	3.154	0.118

0.145	0	0.035	0.084	0.348	0.346	0.101	1.41	1.009	0.318	1.981	0.418	0.521	0.428	0.285	0	0.489	0.163	0.024	0.056	0.016	0.198	0	0.045	0.041	0.242	0.121	0.183	0.025	0.604	0.008	0.586	0.132	0.012	0.48	0.067	0.186	0.007

0.114	0.035	0.201	0.34	0.486	1.124	0.678	2.298	2.138	2.071	1.961	0.279	1.944	0.644	0.933	0.117	1.941	0.487	0.289	0.775	0.163	0.456	0	0.195	0.201	0.289	1.541	1.839	0.846	2.022	0.108	1.487	1.336	0.163	22.54	5.487	4.52	0.071

0.118	0	0.067	0.031	0.749	0.617	0.069	0.844	1.028	0.566	0.634	0.171	0.532	0.292	0.379	0.08	0.828	0.187	0.038	0.042	0.008	0.237	0.026	0.002	0.028	0.172	0.174	0.199	0.003	0.655	0	0.173	0.015	0	8.43	0.141	0.259	0.002

0.214	0.011	0.269	0.043	0.215	0.388	0.2	1.451	0.499	0	0.456	0.284	0.424	0.372	0.266	0.007	0.79	0.313	0.144	0.131	0.042	0.274	0.08	0.025	0.103	0.996	0.864	0.167	1.575	1.36	0.07	0.372	0.211	0.04	2.247	5.288	11.26	0.039

0.418	0.007	0.484	0.364	2.648	2.757	0.371 3.079	1.272	0.249	2.159	0.448	0.578	1.148	0.704	0.108	1.785	1.605	0.132	0.242	0.168	5.88	0.119	0.012	0.239	0.467	0.143	0.622	0.513	0.537	0.021	1.492	0.313	0.13	2.375	0.885	1.197	0.01

0.467	0	0.146	0	1.421	2.69	0.438	0.573	0.439	0.011	0.926	0.121	0.456	2.476	2.294	0.063	1.116	1.509	0.145	0.247	0.241	3.888	0	0.187	0.427	0.634	0.114	0.845	0.433	0.873	0.126	0.536	0.235	0.046	0.783	0.466	1.139	0

0.283	0	0.616	0.554	0.94	2.794	1.313	3.187	2.283	1.352	2.608	1.036	0.916	1.579	1.884	0	2.431	2.469	0.504	1.036	0.711	6.286	0.21	0.774	1.354	0.463	0.852	3.332	1.343	3.037	0.209	3.562	1.426	0.675	8.168	4.212	4.227	0.413

EnhA20

0.35	0.614	0.984	0.564	0.119	0.302	0	0.01	1.305	0.869	0.252	0.119

0.119	0	0.123	0.064	0.692	0.519	0.022	4.211	0.89	0.18	0.588	0.542	0.427	0.148	0.344	0	0.724	0.156	0	0.056	0	0.266	0

0	0.012	1.502	0.021	0.114	0.196	0.348	0	0.321	0.002	0	0.296	0.072	0.519	0.366

TxEnh1

0.39	1.722	1.365	0.959	0.357	0.143	0.08	0.126	2.15	1.513	0.617	0.357

0.259	0.051	0.638	0.305	0.911	0.248	0.092	3.319	1.245	0.351	2.272	0.518	0.172	0.081	0	0.078	0.887	0.5	0.017	0.025	0.016	0.554	0.275	0.008	0.135	0.722	0	0.049	0.006	0.596	0.074	0.989	0.048	0.086	2.453	0.14	0.732	0.152

TxEnh2

0.39	4.134	0.863	1.142	0.346	0.13	0.028	0.153	0.804	3.586	1.92	0.346

0	0	0.496	0.676	0	0.032	0.045	6.25	1.955

0	2.752	0.417	0.074	0	0	0	0.623	0.221	0	0	0	0.333	0.138

0	0.023	0.605	0.225	0	0.308	0.77

0	4.713	1.047	0.446	2.735	1.195	2.931	0.015

TxEnh3

0.25	0.914	0.392	0.902	0.485

0	0.074	0.432	0.389	1	1.03	0.485

0.022	0	0	0.295	0	0	0.064	0.209	0.504	0	0.584	0	0.155	0	0	0	0.613	0.148	0.01	0.013	0	0.055	0	1.092	0.021	0.059	0.214	0	0.319	1.289	0.036	1.465	1.044	0.256	2.512	1.879	1.537	0.006

TxEnh4 TxEnh5 TxEnh6 TxEnh7

0.27	1.631	0.84	1.957	1.29	0.021	0.317	1.562	0.234	1.143	1.793	1.29

0.50	0.704	0.741	1.713	1.013	0.056	0.329	1.199	0.695	0.957	1.097	1.013

0.19	8.495	0.412	1.399	1.321	0.042	0.149	0.777	0.551	1.913	2.863	1.321

0.27	2.321	1.582	3.542	1.673	0.101	0.714	2.483	0.781	2.885	2.885	1.673

0	0	0.065	0.932	0	0.17	0.033	1.012	0.187	0.134	0.277	0	0.56	0.105	0.082	0	0.092	0.261	0	0.061	0.119	0.31	0	3.841	0.108	0.575	1.318	0.565	1.726	1.88	0.044	2.004	1.987	0.885	3.413	5.833	2.337	0.842

0.011	0	0.081	0.127	0.008	0.493	1.036	0.173	1.808	2.121	0.502	0.08	0.446	0.254	0.455	0.108	0.6	0.546	0.092	0.12	0.179	0.654	0.185	1.931	0.323	0.482	0.74	2.573	1.529	1.651	0.063	0.871	1.228	0.302	1.936	1.993	1.487	0.216

0.129	0	1.062	3.455	0	0.459	0.176	13.71	2.077	0.452	4.858	0.496	0.752	0.389	0.99	0	0.776	0.284	0	0.068	0.084	0.235	0	0.511	0.066	0.389	0.767	1.157	1.41	1.055	0	10.98	6.078	3.64	1.642	4.384	3.212	0.241

0.021	0	1.045	1.392	0.18	0.204	0.538	2.05	1.701	1.814	1.612	0.687	1.397	0.056	0	0	2.294	0.731	0.037	0.132	0.072	0.93	0	1.781	0.638	0.565	0.609	0.192	0.644	2.499	0.22	4.335	2.365	0.847	7.528	4.636	3.894	0.57

TxEnh8

0.24	2.203	1.334	2.462	1.993	0.445	0	1.401	1.086	3.487	2.904	1.993

0.098	0	0.131	0.487	0	0.207	0.453	3.962	1.052	0.886	2.182	0.16	3.92	0.17	0.19	0	0.378	0.404	0	0.042	0.264	0.369	0	0.094	0.101	0.374	0.89	0.729	0.594	1.011

0	3.257	0.765	0.429

5.4	3.636	3.533	1.692

TxWk1

2.80	0.133	0.148	0.231	0.032

0.1	9E-04	0.017	0.535	0.173	0.058	0.032

0.023	0.014	0.017	3E-04 0.295	0.343	0.02	0.317	0.137	0.071	0.23	0.013	0.047	0.117	0.14	0.028	0.124	0.081	5E-04	0.001	0.005	0.049	0.032	0.015	0.017	0.218	0.016	0.091	0.005	0.22	0.003	0.071	0.025	6E-04	0.116	0.014	0.074	0.004

TxWk2

0.84	0.147	0.194	0.732	0.314	0.105	0.027	0.227	0.887	0.412	0.374	0.314

0.065	0.044	0.041	0	1.416	1.784	0.94	0.2	1.09	2.224	0.181	0.171	0.049	1.65	1.39	0.221	0.516	0.187	0.06	0.183	0.106	0.106	0.026	0.413	0.061	0.646	0.196	3.337	0.538	0.774	0	0.226	0.273	0.062	0.508	0.543	0.366	0.023

Tx1

0.82	0.597	0.135	0.261	0.026

0	0	0.026	0.497	0.764	0.236	0.026

0.055	0	0.069	0.05	0	0	0	1.236	0.221	0.035	0.208	0.045	0.066	0	0	0	0.057	0.059	0.012	0	0	0.05	0.076

0	0.014	0.19	0	0.014	0.04	0.432	0	0.58	0.077	0.01	0.547	0.075	0.615	0

Tx2

1.58	0.195	0.169	0.248	0.023	0.046	0.003	0.012	0.754	0.253	0.067	0.023

0.036	0.026	0.031	0.025	0.287	0.245	0.035	0.547	0.3	0.161	0.13	0.174	0.139	0.094	0.034	0.023	0.273	0.118	0	0.002	0.007	0.074	0.038	5E-04	0.017	0.487	0	0.07 4E-04	0.452	0	0.148	0.02	0.004	0.258	0.017	0.137	0.016

Tx3

0.51	0.335	0.398	0.828	0.329

0	0.121	0.219	0.721	0.858	0.739	0.329

0.01	0	0.068	0.078	0.124	0.405	0.371	0.271	1.08	0.66	0.511	0.252	0.049	0.208	0.083	0	0.268	0.24	0.011	0.038	0.072	0.247	0	0.502	0.094	0	0.172	0.871	0.137	0.976	0.139	0.966	0.519	0.071	1.604	0.669	1.104	0.012

Tx4

0.47	0.631	0	0.203	0.039	0.023	0.007	0.033	0.161	0.399	0.203	0.039

0	0	0	0	0	0	0	0.419	0.452	0.307	0.445	0.161	0	0	0	0.132	0.065	0.084	0	0	0	0.079	0.035	0.016	0.021	0.3	0	0.089	0	0.354	0

0.4	0.11	0.034	0.441	0.131	0.412	0.018

Tx5

0.94	0.076	0.113	0.31	0.043

0	0.01	0.021	0.253	0.154	0.096	0.043

0.018	0.039	0	0.008	0	0.085	0.066	0.067	0.25	0.268	0.124	0	0.031	0.096	0.043	0.13	0.097	0.065	0	0.003	5E-04	0.022	0.022	0.06	0.022	0.068	0	0.646	0.065	0.355	0	0.131	0.081	0.006	0.188	0.051	0.146	0.015

Tx6

1.11	0.269	0.082	0.148	0.018

0	0	0.004	0.295	0.143	0.043	0.018

0	0	0	0.168	0	0.083	0	0.335	0.234	0	0.69	0.016	0.098	0.02	0	0.163	0.065	0.079	0	0	0.003	0.044	0.049	0.007	0.012	0.489	0	0.009	0.007	0.134	0

0.1	0.007	0.007	0.07	0	0.055	0.015

Tx7

0.82	0.528	0.014	0.274	0.012

0	0.005	0.047	0.095	0.297	0.155	0.012

0	0.051	0	0.071	0	0.093	0	0.618	0.44	0	0.721	0	0.078	0	0	0.033	0.048	0.052	0	0.002	0	0.078	0.073	0.034	0.012	0.326	0.008	0.025	0	0.266	0	0.296	0.015	0.012	0.377	0.114	0.298	0.029

Tx8

0.68	1.762	0.666	0.897	0.254	0.186	0.006	0.214	0.808	1.394	0.904	0.254

0.04	0	0.098	0.275	0.311	0.446	0.077	2.216 1.49	0.116	1.865	0.059	0.449	0.094	0.12	0.091	0.193	0.277	0.003	0.043	0.022	0.434	0.036

0	0.051	0.548	0.044	0.273	0.146	0.6	0	1.433	0.178	0.174	1.731	0.846	1.194	0.216

TxEx1

0.27	1.118	0	0.684	0.359

0	0	0.164	0.104	0.496	0.375	0.359

0	0	0	0	0	0	0	0.921	0	0	0.529	0	0.101	0	0	0	0	0.07	0	0	0	0.078	0.181	0.465	0	0.835	0	0	0	0.891	0	0.728	0.446	0.087	0.957	0.908	0.741	0.174

TxEx2

0.56	0.273	0.063	0.457	0

0	0.099	0.148	0.023	0.267	0.302	0

0	0	0	0.034	0	0	0	0.148	0	0	0.374	0	0.155	0	0	0	0	0.061	0	0	0	0.034	0	0.609	0.025	0.225	0.257	0	0.085	0.308	0.04	0.378	0.258	0.09	0.414	1.053	0.367	0.083

TxEx3

0.66	0.185	0.033	0.535	0.186

0	0.037	0.137	0.128	0.249	0.221	0.186

0	0	0.015	0.055	0	0.079	0.05	0.164	0.307	0.228	0.142	0	0.051	0.023	0	0.02	0.103	0.075	0.018	0.005	0	0.037	0	0.293	0.078	0	0.06	0.356	0.248	0.291	0.041	0.343	0.36	0.155	0.451	0.976	0.431	0.096

TxEx4

0.10	7.604	3.006	2.98	2.542	0.818	0.391	2.934	0.371	3.397	4.401	2.542

0.088	0	0.771	1.047	0	0	0.228	3.535	0.069	0.49	0.411	0	1.768	0	0	0.713	0.712	0.589	0.238	0.284	0.065	0.686	0	2.546	0.405	1.854	0.849	0	1.058	2.562	0.146	6.963	6.197	3.287	6.093 10.67 4.656	5.13

znf1

0.41	0.245	0.12	0.429	0.051

0	0	0.056	0.084	0.295	0.104	0.051

0	0.148	0	0	0	0.071	0	0.203	0	0.294	0.123	0	0	0.118	0.147	0	0.134	0.051	0	0	0	0.045	0.133	0.109	0	0.841	0.132	0.234	0.026	0.628	0	0.242	0.102	0.027	0.62	0.071	0.461	0.023

znf2

0.15

0.1	0.256	0.409	0.083

0	0.099	0.082	0.071	0.294	0.249	0.083

0	0	0	0	0	0	0	0	0	0	0.185

0	0	0	0	0	0	0.006	0	0	0	0.026	0	1.512	0.001	0.656	0	0	0.199	0.814	0	0.134	0	0.169	0.653	0.208	1.055	0.055

DNase1

0.20	6.651	40.35	12.62	24.9	1.133	0.722	11.76	2.912	7.11	7.091	24.9

0.755	0.971	1.797	5.201	0.836	2.385	4.937	2.139	2.053	0.314	3.177	1.682	2.092	1.122	0.539	0.439	2.99	2.443	1.792	2.197	1.531	1.966	0.562	0.719	1.15	8.359	41.71	2.301	54.17	12.88	1.055	8.169	17.2	2.471	18.35	9.677	10.85	3.648

BivProm1	0.15	8.585	15.19	16.56	23.32	88.57	127.1	33.06	14.65	13.26 13.12	23.32	157.4	233.4	151.3	139	33.6	5.471	127.8	0.791	53.38	113.7	10.44	131.1 101.4	55.56	59.66	184.5	76.8	87.72	187.5	160.7	171.4	104.9	214	106.1	125.9	5.505	13.76	94.16	19.45 16.07	155.4	7.117	23.7	5.71	3.716	7.578	7.301	5.057

BivProm2	0.16	4.512	9.763	6.154	8.13	26.39	163.1	9.696	10.84	4.728	4.914	8.13

BivProm3	0.29	2.666	7.001	6.598	4.201	4.153	7.097	3.693	4.596	3.035	2.784	4.201

BivProm4	0.13	3.818	10.01	5.934	4.943	12.24	32.33	3.091	14.23	4.667	4.259	4.943

169.3 175.1 70.51 111.2	11.72	4.191	104.2	1.162	61.08	99.37	7.635	77.81	51.68	21.97	23.79	189.3 20.86 91.83	176.1	168.8	199	117.2	235.8	179.5	142.1	8.731	6.359	66.19	10.6	5.362	202.2	1.944	5.01	1.182	2.072	3.542	4.618	0.74

12.79 3.805	6.35	4.105	14.17	12.04	16.57	0.951	14.28	19.18	5.351	9.187	5.391	11.44	12.1 8.043	6.712	15.02	19.71	20.8	12.13	9.806	7.657	2.695	11.47	0.566	2.228	13.33	5.857	3.219	10.9	1.458	1.881	0.414	3.676	3.484	3.386	0.334

17.31 13.58 18.76 24.04 7.964	2.911	21.06	1.567	12.57	12.59	7.675	24.8	7.147	4.432	3.505	24.52 8.332 28.38	8.175	8.187	11.84	22.69	4.847	20.41	29.87	0.801	0.616	8.924	2.242	2.573	28.07	1.831	0.498	0.69	2.896	2.456	2.079	4.353

PromF1 PromF2 PromF3

0.20	9.726	15.28	12.46	25.06	2.418	2.462	20.94	4.701	13.51 17.23	25.06

0.14	22.13	20.96	13.01	30.89	9.378	7.606	20.31	11.01	22.32	29.61	30.89

0.15	21.45	25.43	21.79	53.87	57.31	16.49	48.16	28.31	38.44	38.32	53.87

0.726	0.679	7.081	5.075	0.429	1.183	8.061	7.733	6.828	6.323	4.998	3.448	10.63	1.416	0.65	0.482	6.693	4.46	4.245	5.635	2.331	3.147	0.815	1.954	4.068	1.957	14.4	3.981	18.27	8.485	1.388	15.66	16.69	6.525	19.43	23.99	17.62	3.175

0.551	1.445	14.11	10.22	1.297	0.037	5.197	8.39	5.976	2.431	12.07	8.155	13.4	0.46	0.1	0.313	8.799	7.8	1.59	1.451	1.893	6.158	0.278	1.592	7.357	4.501	10.81	2.922	15.65	12.3	3.343	31.61	27.16	27.98	16.89	29.33	29.14	17.73

0.876	4.722	29.55	13.5	3.611	0.395	7.222	1.803	4.384	3.848	3.003	24.63	18.37	0.905	0.452	0.82	24.76	14.18	2.615	1.358	0.95	13.39	0.674	1.578	16.41	9.463	20.27	7.381	18.01	25.03	5.998	29.88	45.36	41.75	13.3 37.37 43.58	38.03

PromF4

0.19	26.25	53.96	38.43	93.38

47	11.82	127.8	16.21	53.44	92.16	93.38

1.091	6.588	21.64	9.649	1.111	0.577	4.35	1.119	0.982	1.373	0.573	14.23	36.81	1.764	1.073	1.225	20.7	13.77	1.596	0.603	0.422	14.35	0.619	0.441	14.84	45.67	118.3	17.88	89.7	54.13	3.793	45.28	116.8	203.8	22.58	102	85.15	197.3

PromF5

0.14	13.89	25.67	25.48	36.09	114.2	66.3	52.49	19.44	24.08	21.34	36.09

54.14 140.2 168.4	107	29.99	3.075	78.88	1.828	39.32

44	11.74	147.1	63.57	35.21	35.96	38.48	100.1	61.27	59.29	36.42	46.15	55.78	29.6	23.75	73.86	5.956	17.39	71.49	22.49	24.7	52.77	16.22	37.51	16.84	9.31	17.42	13.87	35.47

PromF6

0.13	8.555	4.343	3.58	2.671	4.552	2.262	3.147	7.664	11.96	4.269	2.671

0.224	0.94	4.561	5.694	0.225	0.095	1.173	2.558	1.247	0.555	2.74	9.424	0.241	0.375	0	2.645	3.918	3.938	0.32	0.272	0.203	3.256	0.716	0.134	2.86	1.038	2.392	1.106	3.243	5.679	1.838	4.755	5.139	3.442	2.994	3.304	7.049	1.945

PromF7

0.16	3.894	2.096	1.183	0.676	1.524	0.755	0.741	3.635	6.005	1.568	0.676

0.29	0	2.001	1.747	0.394	0.038	0.56	2.099	2.318	0.224	3.394	3.699	0.05	0.013	0	0.673	2.895	1.656	0.271	0.095	0.081	1.489	0.406

0	0.852	0.435	0.068	0.23	0.311	1.784	0.498	1.787	0.707	0.336	1.716	0.992	3.199	0.194

TSS1

0.12	31.43	114.5	50.57	105.5	43.56	16.84	106.5	11.09	37.96 83.09	105.5

5.059	12.11	35.61	11.42	2.808	0.855	9.455	0.914	1.517	1.62	1.263	26.64	57.48	5.721	4.582	2.653	35.53	20.57	9.499	3.275	2.055	18.67	3.756	0.97	22.86	75.02	245.9	24.76	175.7	69.54	11.01	33.33	127.9	181.8	23.26	79.06	51.6	235.5

TSS2

0.11	16.31	60.38	24.6	56.86	22.14	15.94	44.59	7.96	19.25	34.91	56.86

18.76	21.14	28.21	12.7	2.941	2.556	14.77	0.046	5.123	5.909	1.616	15.7	35.87	11.47	11.35	6.503	19.03	27.05	37.02	17.52	10.16	21.08	10.57	2.976	24.36	41.13	128.7	19.99	98.65	32.97	21.22	14.12	54.74

55	11.39	25.93	18.29	87.7

100.0	0.178	0.026	0.114	0.052	0.059	0.185	0.182	0.095	0.607	0.225	0.052	0.117	0.035	0.051	0.018	0.01	0.053	0.094	0.011	0.013	0.018	0.023	0.016	0.052	0.054	0.032	0.024	0.021	0.816	0.222	0.202	0.101	0.416	0.026	0.036	0.359	0.012	0.015	0.037	0.022	0.07	0.101	0.462	0.073	0.115	0.26	0.036	0.191	0.109

**Supplementary Figure 16: Full-stack states enrichments with Polycomb Repressive protein complexes PRC1 (green column headers) and PRC2 (orange column headers).** The heatmap shows enrichment values for the full-stack states (rows) and the binding sites of subunits of PRC1 and PRC2 in different cell types (columns) that were available from ENCODE project (**Methods**). Coloring of enrichments is column specific with highest and lowest enrichment values in each column are colored red and white, respectively. The first two columns show state names, and

### their percentage of genome coverage. The last row shows the percentage of genome coverage for each type of PRCs.

**Top enriched states with PRC1 and PRC2**

**State	%**

**genom e**

**BMI1**

**RIN G 1**

**RN F2**

**EZH2**

**SUZ12**

**EED**

**GapArtf3**	0.01	1.1	16	9.2	16	12	16	4.1	1.4	1.1	1.4	16	3.9	3.5	0.2	3.1	35	8.6	2.8	26	0	0	0	0	5.5	17	20	62	18	2.3	4.4	5.6	7.9	5.3	0.1	19	2	28	32	14	42	15	11	2.5	9.1	12	2.4	13	2	3.2

**HET2**	0.69	0.1	0.2	1.2	0.1	0.4	0.1	0.2	0.2	0.1	0.1	0.1	0.8	0	0	0	3.7	11	2.3	0	0.2	2.3	0.2	0	1	19	20	0.2	0.4	0.3	0	0	0.1	0.2	0	0.9	0.1	0.8	0.1	3.8	0.3	1	0	0.1	0.4	0	0.2	0	0.1	0

**ReprPC1**	0.19	2.2	6.9	2.5	4.1	12	120	2.6	10	2.3	1.8	4.1	75	44	25	58	14	9.6	50	0.7	33	39	4	33	12	12	12	85	8.8	75	69	91	108	74	90	109	100	3.5	1.2	29	3.2	2.8	100	0.5	0.7	0.1	0.6	1.3	1.6	0.1

**ReprPC5**	0.63	0.3	0.8	2.1	1.1	0.9	0.2	0.7	1.5	0.7	0.7	1.1	3.3	0	0.5	0.1	11	19	10	0.4	7.9	12	1.1	0.5	1.9	13	14	2.4	4.8	0.7	2.7	4.6	2.5	0.4	0.3	1.8	0.5	0.6	0.5	8	1.2	2.4	0.2	0.4	0.5	0.1	0.5	1.1	0.6	0

**Acet1**	0.18	0.9	0.3	14	1.1	0.1	1.7	1.5	1.3	1.2	1.6	1.1	0.1	0	0.1	1.1	1.3	3.2	1.3	0	2	1.2	0.6	0	0.4	2.6	2.6	0.4	0.6	0.6	0.3	0.9	2.2	0.3	0.7	76	0.4	9	2	2.7	3.3	36	0.1	0.7	2.2	0.2	2.3	1.9	2.8	0.1

**EnhWk7**	0.48	17	0.5	0.5	0.2	0.2	0	0.1	1.6	0.6	0.2	0.2	0.1	0	1.2	2.7	1.3	0.7	0	1.4	5.5	0	16	0.6	0.1	0.2	0.1	0	1.4	0.2	0.1	0.1	0	0.2	0.1	0	0	0.1	0	0	0.1	0.6	0	14	2.7	1.3	0.4	0.1	0.2	0

**EnhA 8**	0.25	23	3.6	2.2	4.7	1.3	0.6	1.4	3.2	5.5	8.4	4.7	0.6	0	5.1	12	1.3	1.8	1.8	23	16	1	21	1.9	1.1	2.3	1.2	0.4	7.8	1.8	1.1	1.9	0.8	0.9	0.6	0.4	1	0.3	0.5	2.2	1.5	1.3	0.6	26	19	17	2.5	6.8	6.4	0.8

**EnhA 9**	0.16	22	5.9	4.3	11	0.1	0.4	4.4	3.2	9.9	15	11	0	0	4.6	11	0.8	0.1	0.8	22	9	0.3	15	1.6	4.1	0.3	0	0.5	5.5	0.9	0.3	0.4	0.3	0.9	0	0.8	0.5	0.5	1.6	0.6	5.2	2.5	0.1	35	25	21	8.4	18	14	3

**Enh A 10**	0.39	16	3.1	2.2	2.9	0.9	0.2	1.2	3	8.3	7.9	2.9	0.2	0.1	2.4	7.4	0.2	0.5	0.5	21	7.3	0.3	18	1.4	1.6	0.1	0	0.1	3	0.7	0.1	0.2	0.1	0.5	0.1	0	0.3	0.4	0.4	0.4	1.5	1.7	0.1	15	3.3	3	4.5	4.8	8.2	0.4

**Enh A 14**	0.37	1.9	1.8	3.6	2.5	0.4	0.1	8.4	1.3	4.5	3.9	2.5	0.1	0	0.2	0.3	0.5	1.1	0.7	2.3	2.1	2.1	2	0.3	1.9	0.6	0.9	0.1	1.9	0.5	0.3	0.8	0.2	0.5	0	0.2	0.2	0.3	1.5	1.8	0.8	2	0.1	1.5	1.3	0.2	23	5.5	4.5	0.1

**BivProm1**	0.15	8.6	15	17	23	89	127	33	15	13	13	23	157	233	151	139	34	5.5	128	0.8	53	114	10	131	101	56	60	185	77	88	188	161	171	105	214	106	126	5.5	14	94	19	16	155	7.1	24	5.7	3.7	7.6	7.3	5.1

**BivProm2**	0.16	4.5	9.8	6.2	8.1	26	163	9.7	11	4.7	4.9	8.1	169	175	71	111	12	4.2	104	1.2	61	99	7.6	78	52	22	24	189	21	92	176	169	199	117	236	180	142	8.7	6.4	66	11	5.4	202	1.9	5	1.2	2.1	3.5	4.6	0.7

**BivProm3**	0.29	2.7	7	6.6	4.2	4.2	7.1	3.7	4.6	3	2.8	4.2	13	3.8	6.3	4.1	14	12	17	1	14	19	5.4	9.2	5.4	11	12	8	6.7	15	20	21	12	9.8	7.7	2.7	11	0.6	2.2	13	5.9	3.2	11	1.5	1.9	0.4	3.7	3.5	3.4	0.3

**Prom F3**	0.15	21	25	22	54	57	16	48	28	38	38	54	0.9	4.7	30	13	3.6	0.4	7.2	1.8	4.4	3.8	3	25	18	0.9	0.5	0.8	25	14	2.6	1.4	0.9	13	0.7	1.6	16	9.5	20	7.4	18	25	6	30	45	42	13	37	44	38

**Prom F4**	0.19	26	54	38	93	47	12	128	16	53	92	93	1.1	6.6	22	9.6	1.1	0.6	4.3	1.1	1	1.4	0.6	14	37	1.8	1.1	1.2	21	14	1.6	0.6	0.4	14	0.6	0.4	15	46	118	18	90	54	3.8	45	117	204	23	102	85 197

**Prom F5**	0.14	14	26	25	36	114	66	52	19	24	21	36	54	140	168	107	30	3.1	79	1.8	39	44	12	147	64	35	36	38	100	61	59	36	46	56	30	24	74	6	17	71	22	25	53	16	38	17	9.3	17	14	35

**TSS1**	0.12	31	115	51	106	44	17	106	11	38	83	106	5.1	12	36	11	2.8	0.9	9.5	0.9	1.5	1.6	1.3	27	57	5.7	4.6	2.7	36	21	9.5	3.3	2.1	19	3.8	1	23	75	246	25	176	70	11	33	128	182	23	79	52 236

**TSS2**	0.11	16	60	25	57	22	16	45	8	19	35	57	19	21	28	13	2.9	2.6	15	0	5.1	5.9	1.6	16	36	11	11	6.5	19	27	37	18	10	21	11	3	24	41	129	20	99	33	21	14	55	55	11	26	18	88

GM12878

K562

MCF-7

K562

A549

H1

HepG2

K562

K562

K562

K562

A673

B_cell

DOHH2

GM12878

HCT116

HeLa-S3

HepG2

Loucy

MM.1S

MM.1S

OCI-LY3

OCI-LY7

PC-3

PC-9

PC-9

SK-N-SH

SU-DHL-6

bipolar_neuron

fibroblast_of_dermis

fibroblast_of_lung

mammary_epithelial_cell

neural_progenitor_cell

skeletal_muscle_myoblast

H1

H1

HEK293T

HepG2

K562

K562

MCF-7

NT2

GM12878

GM12878

GM12892

HepG2

K562

K562

endothelial_cell_of_umbilical_vei

State rank

1

2

3

**EZH2**

**rank**

**-10000**

**-9800**

**-9600**

**-9400**

**-9200**

**-9000**

**-8800**

**-8600**

**-8400**

**-8200**

**-8000**

**-7800**

**-7600**

**-7400**

**-7200**

**-7000**

**-6800**

**-6600**

**-6400**

**-6200**

**-6000**

**-5800**

**-5600**

**-5400**

**-5200**

**-5000**

**-4800**

**-4600**

**-4400**

**-4200**

**-4000**

**-3800**

**-3600**

**-3400**

**-3200**

**-3000**

**-2800**

**-2600**

**-2400**

**-2200**

**-2000**

**-1800**

**-1600**

**-1400**

**-1200**

**-1000**

**-800**

**-600**

**-400**

**-200**

**0**

**200**

**400**

**600**

**800**

**1000**

**1200**

**1400**

**1600**

**1800**

**2000**

**2200**

**2400**

**2600**

**2800**

**3000**

**3200**

**3400**

**3600**

**3800**

**4000**

**4200**

**4400**

**4600**

**4800**

**5000**

**5200**

**5400**

**5600**

**5800**

**6000**

**6200**

**6400**

**6600**

**6800**

**7000**

**7200**

**7400**

**7600**

**7800**

**8000**

**8200**

**8400**

**8600**

**8800**

**9000**

**9200**

**9400**

**9600**

**9800**

**10**

**Neighborhood enrichments with PRC1 and PRC2: ranked states by descending enrichments**

1

2

3

4

5

6

7

8

9

10

**SUZ12**

1

2

3

4

5

6

7

8

9

10

**rank**

**-10000**

**-9800**

**-9600**

**-9400**

**-9200**

**-9000**

**-8800**

**-8600**

**-8400**

**-8200**

**-8000**

**-7800**

**-7600**

**-7400**

**-7200**

**-7000**

**-6800**

**-6600**

**-6400**

**-6200**

**-6000**

**-5800**

**-5600**

**-5400**

**-5200**

**-5000**

**-4800**

**-4600**

**-4400**

**-4200**

**-4000**

**-3800**

**-3600**

**-3400**

**-3200**

**-3000**

**-2800**

**-2600**

**-2400**

**-2200**

**-2000**

**-1800**

**-1600**

**-1400**

**-1200**

**-1000**

**-800**

**-600**

**-400**

**-200**

**0**

**200**

**400**

**600**

**800**

**1000**

**1200**

**1400**

**1600**

**1800**

**2000**

**2200**

**2400**

**2600**

**2800**

**3000**

**3200**

**3400**

**3600**

**3800**

**4000**

**4200**

**4400**

**4600**

**4800**

**5000**

**5200**

**5400**

**5600**

**5800**

**6000**

**6200**

**6400**

**6600**

**6800**

**7000**

**7200**

**7400**

**7600**

**7800**

**8000**

**8200**

**8400**

**8600**

**8800**

**9000**

**9200**

**9400**

**9600**

**9800**

**1**

**EED**

1

2

3

4

5

6

7

8

9

10

**rank**

**-10000**

**-9800**

**-9600**

**-9400**

**-9200**

**-9000**

**-8800**

**-8600**

**-8400**

**-8200**

**-8000**

**-7800**

**-7600**

**-7400**

**-7200**

**-7000**

**-6800**

**-6600**

**-6400**

**-6200**

**-6000**

**-5800**

**-5600**

**-5400**

**-5200**

**-5000**

**-4800**

**-4600**

**-4400**

**-4200**

**-4000**

**-3800**

**-3600**

**-3400**

**-3200**

**-3000**

**-2800**

**-2600**

**-2400**

**-2200**

**-2000**

**-1800**

**-1600**

**-1400**

**-1200**

**-1000**

**-800**

**-600**

**-400**

**-200**

**0**

**200**

**400**

**600**

**800**

**1000**

**1200**

**1400**

**1600**

**1800**

**2000**

**2200**

**2400**

**2600**

**2800**

**3000**

**3200**

**3400**

**3600**

**3800**

**4000**

**4200**

**4400**

**4600**

**4800**

**5000**

**5200**

**5400**

**5600**

**5800**

**6000**

**6200**

**6400**

**6600**

**6800**

**7000**

**7200**

**7400**

**7600**

**7800**

**8000**

**8200**

**8400**

**8600**

**8800**

**9000**

**9200**

**9400**

**9600**

**9800**

**1**

**RNF2**

1

2

3

4

5

6

7

8

9

10

**rank**

**-10000**

**-9800**

**-9600**

**-9400**

**-9200**

**-9000**

**-8800**

**-8600**

**-8400**

**-8200**

**-8000**

**-7800**

**-7600**

**-7400**

**-7200**

**-7000**

**-6800**

**-6600**

**-6400**

**-**

**6200**

**-6000**

**-5800**

**-5600**

**-**

**5400**

**-5200**

**-5000**

**-4800**

**-**

**4600**

**-**

**4400**

**-4200**

**-4**

**000**

**-3800**

**-3600**

**-3400**

**-**

**3200**

**-**

**3000**

**-**

**2800**

**-2600**

**-**

**2400**

**-2200**

**-2000**

**-1800**

**-**

**1600**

**-**

**1400**

**-1200**

**-1**

**000**

**-800**

**-600**

**-400**

**-200**

**0**

**200**

**400**

**600**

**800**

**1000**

**1200**

**1400**

**1600**

**1800**

**2000**

**2200**

**2400**

**2600**

**2800**

**3000**

**3200**

**3400**

**3600**

**3800**

**4000**

**4200**

**4400**

**4600**

**4800**

**5000**

**5200**

**5400**

**5600**

**5800**

**6000**

**6200**

**6400**

**6600**

**6800**

**7000**

**7200**

**7400**

**7600**

**7800**

**8000**

**8200**

**8400**

**8600**

**8800**

**9000**

**9200**

**9400**

**9600**

**9800**

**10**

**RING1**

1

2

3

4

5

6

7

8

9

10

**rank**

**-10000**

**-9800**

**-9600**

**-9400**

**-9200**

**-9000**

**-8800**

**-8600**

**-8400**

**-82 0**

**0**

**-8000**

**-7800**

**-7600**

**-7400**

**-7200**

**-7000**

**-6800**

**-66 0**

**0**

**-6400**

**-6200**

**-6000**

**-5800**

**-5600**

**-5400**

**-52 0**

**0**

**-5000**

**-4800**

**-4600**

**-4400**

**-4200**

**-4000**

**-3800**

**-3600**

**-3400**

**-3200**

**-3000**

**-2800**

**-2600**

**-2400**

**-2200**

**-2000**

**-1800**

**-1600**

**-1400**

**-1200**

**-1000**

**-800**

**-600**

**-400**

**-200**

**0**

**200**

**400**

**600**

**8 0**

**0**

**100**

**0**

**1200**

**1400**

**1600**

**1800**

**2000**

**2200**

**2400**

**2600**

**2800**

**3000**

**3200**

**3400**

**3600**

**3800**

**4000**

**4200**

**4400**

**4600**

**4800**

**5000**

**5200**

**5400**

**5600**

**5800**

**6000**

**6200**

**6400**

**6600**

**6800**

**7000**

**7200**

**7400**

**7600**

**7800**

**8000**

**8200**

**8400**

**8600**

**8800**

**9000**

**9200**

**9400**

**9600**

**9800**

**10**

**BMI1**

**rank**

**-10000**

**-9800**

**-9600**

**-9400**

**-9200**

**-9000**

**-8800**

**-8600**

**-8400**

**-8200**

**-8000**

**-7800**

**-7600**

**-7400**

**-7200**

**-7000**

**-6800**

**-6600**

**-6400**

**-6200**

**-6000**

**-5800**

**-5600**

**-5400**

**-5200**

**-5000**

**-4800**

**-4600**

**-4400**

**-4200**

**-4000**

**-3800**

**-3600**

**-3400**

**-3200**

**-3000**

**-2800**

**-2600**

**-2400**

**-2200**

**-2000**

**-1800**

**-1600**

**-1400**

**-1200**

**-1000**

**-800**

**-600**

**-400**

**-200**

**0**

**200**

**400**

**600**

**800**

**1000**

**1200**

**1400**

**1600**

**1800**

**2000**

**2200**

**2400**

**2600**

**2800**

**3000**

**3200**

**3400**

**3600**

**3800**

**4000**

**4200**

**4400**

**4600**

**4800**

**5000**

**5200**

**5400**

**5600**

**5800**

**6000**

**6200**

**6400**

**6600**

**6800**

**7000**

**7200**

**7400**

**7600**

**7800**

**8000**

**8200**

**8400**

**8600**

**8800**

**9000**

**9200**

**9400**

**9600**

**9800**

**10**

1

2

3

4

5

6

7

8

9

10

### **Supplementary Figure 17: Enrichments of selected full-stack states with PRC1 and PRC2. (A)** A subset of Supp. Fig. 16 showing fold enrichment of full-stack states for binding sites of polycomb repressive protein complexes in different cell types from ENCODE (Methods). The column names highlighted in green (PRC1) and orange (PRC2) show the subunits of PRCs (BMI1, RING1 and RNF2 in PRC1, and EZH2, SUZ12, EED in PRC2) and the cell types where the PRCs were profiled in the

second and first rows of column names, respectively. Each row corresponds to a state, and only states that were among the top three with greatest enrichments for at least one category of PRC complexes are shown. Top enrichment values are colored red based on the rank of the state for each score as indicated in the color legend at the bottom. Some states that show strong consensus across cell types in enrichments with PRC1 and PRC2 include ReprPC1, BivProm1-2, PromF4-5, TSS1-2. ReprPC1 and BivProm1-2 all show strong signals of H3K27me3. **(B)** Neighborhood enrichments of full-stack states with binding sites of PRC1 and PRC2 complexes. In each subpanel, each column corresponds to a 200-bp bin across the 20,000-bp regions overlapping and surrounding annotated PRC1&2 subunit complexes. Within each column, the top 10 states most enriched at the corresponding 200-bp position (within the 20,000bp window) are shown, in descending order of enrichments, and colored based on the state groups as presented throughout the paper. **Supp. Data. File 2** accompanies this figure to show full state names and rankings.

We added two sentences to the section “Results/Major groups of full-stack states” that reads: “Compared to other full-stack state groups, those associated with promoters (TSS, flanking promoters, bivalent promoters) and the DNase group showed lower average DNA methylation levels across cell types (**Supp. Fig 15**). Among promoter-associated states, those showing stronger enrichments with CpG Islands also show lowest methylation levels (**Fig. 3A, Supp. Fig. 15**), consistent with previous studies [36,37]. Some promoter-associated states (TSS1-2, PromF3-5, BivProm1-2) are most enriched at the center of binding regions of polycomb repressed complex 1 and 2 (PRC1 and and PRC2) sub-units. In addition, several ReprPC and BivProm states are among the most enriched states in windows surrounding binding regions of the EZH2 and SUZ12 subunits of PRC2, consistent with these states’ association with H3K27me3 (**Supp. Fig. 16-17**).”

*The comparison of full-stack and concatenated models on predicting external genomic annotations is like attacking a strawman. For example, in predicting annotated exons (Fig. 4A), the full-stack annotation would obviously do better than annotations in individual samples since only a subset of genes is expressed in a particular sample. But what does this prove? It is hardly a useful application to predict exons using chromatin states since the former is better annotated than the latter. I suggest the authors tone down the expectation in this section and keep Figure 4 in Supplementary Materials.*

We thank the reviewer for the feedback. Our intent with using well annotated genomic features such as exons for the evaluations is *not* to argue that chromatin state approaches are the best way to recover these features. Our intent rather was to suggest that because the full-stack annotations performed better for these well annotated features where it was easier to evaluate, we would expect that for other less well-annotated features, where chromatin states are more commonly relied on for annotations (e.g. enhancers), we would expect the full-stack annotations to also be more informative. We have modified the text to clarify our motivations with using well annotated features in the evaluations.

We agree that since the full stack annotations use information from multiple cell types/biosamples, it is not particularly surprising that it performs better in most of the evaluations. We do think this point should be made quantitatively to rigorously confirm an expected strength of the full-stack annotations. We agree with

the reviewer’s advice and moved the comparison figure to the supplementary materials and clarified the expectations from this analysis. We also shortened the coverage in the main text by removing the section ‘Results/Full-stack states are more predictive of external annotations than cell-type-specific models’, and instead replacing it as part of one paragraph at the end of the section ‘Results/Major groups of full-stack states’

“We verified that enrichments computed based on hg19 were highly similar to those computed for full- stack annotations mapped to hg38 (average correlation 0.99; **Methods, Supp. Fig. 18)**, ensuring the applicability of state annotations in hg38. We also confirmed that the full-stack annotations were generally more predictive of the positions of a variety of external genome annotations considered in **Fig. 3A** than two sets of per-cell-type annotations, a previous 18-state per-cell-type chromatin state annotation based on concatenated models from 127 cell types [3] and 100-state per-cell-type annotations learned independently in each cell type (**Methods**). As expected, since the full-stack model uses more data representing more cell types, the full-stack annotations had greater predictive performance in most cases (**Supp. Fig. 19-22, Supplementary Data 4)**. One of the exceptions to this was lambin B1 associated domains from Tig3 human lung fibroblasts [5], where six per-cell type annotations were more predictive, three of which are annotations for fibroblasts. We note that these evaluations were done under the assumption that a chromatin state annotation that is more predictive of well-established external genomic elements will also be more informative of less well-established classes of elements. These results suggest that the full-stack annotations will, in most cases, have greater information than any single per-cell-type annotations about localization patterns of some target genomic elements, with likely exceptions when the target of interest is specific to a certain cell type. In such cases, the corresponding per-cell type chromatin state annotation may show better recovery.”

We also updated the Methods section ‘Receiver operator characteristic curve analysis for predicting external annotations’, which now reads:

“To evaluate how well the chromatin state annotations from different ChromHMM models can inform us about the position of external genomic annotations, we computed the Receiver Operator Characteristic (ROC) in a procedure as follows: First, we divided the genome into 200bp bins, and randomly partitioned 50% of the bins for training and the remaining 50% for testing. Second, we computed the enrichment of the target external annotation with each chromatin state on the training data, and ranked states in decreasing order of such enrichments. We used this ranking of states to iteratively add genomic bases assigned to the states as our predictions of bases that overlap the target annotation in the testing dataset. Based on the overlap of the predictions and the target annotation at each iteration, we plotted ROC curves and summarized the information by computing area under the ROC curves (AUROC).”

*The authors performed all the segmentation in hg19 and then lifted-over chromatin annotations in each 200-bp bin to GRCh38. Currently, almost all annotations are done on GRCh38. In order for the full-stack annotations to be a more useful resource, can the authors also run the whole pipeline in GRCh38 from reads alignment to segmentation? Please also provide a comparison between the state annotations in hg19 and GRCh38 to show whether the full-stack modeling is sensitive to a change in the version of the reference genome.*

We agree with the reviewer that having a full-stack chromatin state annotation learned directly in GRCh38 would be useful and is something we plan to provide in the future, but feel that it should be considered out of scope in this paper for several reasons.

First, hg19 was the assembly that was used by the Roadmap Epigenomics Consortium integrated analysis efforts. Training the stacked model in hg19 allowed us to build off the data processing and metadata that went into that work, and to directly compare the full-stack annotations against the per-cell-type annotations based on concatenated models that are widely used by the community. To reproduce exactly the same data processing as used in the Roadmap Epigenomics Integrative analysis efforts for hg38 would be a massive undertaking outside the timeframe for revisions. This is the case since in addition to doing computationally intensive read mappings of thousands of libraries, we would also need to reconstruct and execute a detailed and complex set of preprocessing steps involving pooling libraries, read filtering, and down sampling previously done by other people.

Second, we expect that the liftOver annotations (from hg19 to hg38) we are providing can already be a valuable resource for the vast majority of applications in hg38. We realized, based on a comment from reviewer 1, that the high number of bins that we reported as not mapping from hg19 to hg38 might have given the impression that the liftOver is losing more information than it actually is since the vast majority of the unmapped bases from hg19 to hg38 are in assembly gaps of hg19 (98.7%) and the GapArtf1 state (99.7%). Conversely, in total 97.1% of the hg38 reference genome that is not in an assembly gap received a state annotation. In our revised version, we have conducted enrichment analysis for a variety of different genomic contexts in both hg19 and hg38 and see almost identical enrichments with an average correlation of 0.99 across states (**Supplementary Figure 18**). This added analysis validates the quality of our liftOver and suggests most genome wide analyses should be unaffected by using the liftOver annotations.

Third, if the suggestion is to also relearn a new model directly in hg38, then in general one would not expect exactly the same set of states to be learned. This would then also require reanalyzing and reinterpreting each state of the model. We feel the presence of two similar, but not identical models, would overcomplicate this manuscript and create confusion for the community.

Given the large amounts of additional data that has emerged from various epigenome consortia mapping efforts, we feel that for a new model in hg38 the community would be best served if it incorporated this additional data. We are currently working as part of a large collaborative effort that is reprocessing such data in a unified manner, and we are planning to provide in the future a stacked model based on such data.

The supplementary figure that we have added comparing enrichments for the hg19 and the hg38 liftOver based annotations is shown below.

**hg19 state enrichments with hg19 genome contexts	hg38 (liftedOver) state enrichments with hg38 genome contexts**

1.34	1.11

1.12

1.06

1.07

1.02

0.89

0.7

1.27

1.44

0.74

0.04	0.39	0.65

0.48	0.23	0.45

0	0.14	0.25	0

0	0.28	0.37	0

0.02	0.34	0.57	0

0.6	1.27	1.3	0

1.19	0.73	0.76	0

2.73	0.6	0.78	0

1.99	4	0

0.3	0.23	0.4	0

0.73

46.7

1.94

5.17

7.57

3.41

0.001

0.68

0.001

42.1

2.45

1.15

100

2.13

0.57

1.74

4.46

75.4	106	20.1

1.15

8.12

5.53

0.29

0.95

4.89

25.9

101 152

1.44

12.6

6.48

**TSS1 TSS2**

0.68

1.53

20.4

2.5

2.63

2.44

1.72	2.04

0.48

1.95

26.4

1.55	2.74

0.09	1.05

3.64

2.1

2.08

3.69	3.25

22.4

31.9

71

8.49	1.56

7.33

4.77

2.27

4.34

4.41

3.75

0.12

0.16

0.57

0.72

0.61

0.71

4.13

3.95

28

62.2

96.7

2.52

2.42

1.89

13.5

10.2

25.7

13.3

25.3

1.52

5.09

4.63

2.34

2.19

13.9

4.35

4.37

1.38

3.21

2.4

1.85

6.46

2.35

1

**PromF1 PromF2 PromF3 PromF4 PromF5 PromF6 PromF7**

3.91

14.5

2.99

1.74

0.8	0.42	0.82

0.6	0.3	1.28

0.63	0.45	0.81

0.46	0.39	1.23

0.53	0.31	3.39

2.47

7.02

7.19	6.18

3.75	8.11

2.6	6.51

3.88	8.11

2.23

1.16

2.95	2.8

2.55	3.39

2.16	2.41

3.63

14.9

14.8

54.5

4.03

1.32

7.56	6.41

0.16	0.87

0.18	0.5

1.02

1.03

3.94

19.3

28.7

77

9.39	8.49	1.43

**BivProm1 BivProm2 BivProm3 BivProm4**

0.8

1.1	0.53

2.04

2.42

2.57

1.07

1.51

0.99

**DNase1**

68.6

1.21

1.88

1.16

0

0

0

0

0

0

0

0

0

0

0

0

0

0

0

0.92	0.93	0.84

3.59	5.59	1.88

3.28

7.13

10.4

20.8

1.67

1.33

0.6

1.39

2.13

2.14

1.06	1.22

**znf1 znf2**

0.41

0.37

3.09

9.28

7.83

10.7

0.83

0.9

2.21

7.04

9.74

0.54

3.65

9.97

15.8

2.9

2.32

2.32

2.25

2.26

6.47

10.4

**TxEx1 TxEx2 TxEx3 TxEx4**

1.74

4.03

3.88

4.1

4.26

2.2

3.51

2.51

2.46

6.81	5.82

0.69

0.67

0.74

0.72

0.4

0.38

0.51

0.5

0.57

2.03

4.91

4.92

4.39

3.6

1.35

0.96

0.95

2.65

2.89	2.87

0.6

1.33

1.38

2.66	2.15

1.04

1.01

0.57	0.48

0.84	0.61

1.15

1.17

0.38

0.85

0.3	0.01	0.18	0.67

0.35	0.04	0.15	0.48

0.29	0.19	0.39	0.63

2.31

2.27

2.26

2.31

2.21

2.27

2.29

0.2	0.21

0.18	0.25

0.3	0.36

**Tx1 Tx2 Tx3 Tx4 Tx5 Tx6 Tx7 Tx8**

0.77

0.87

1.66

2.51

4.8

6.61

1.11

1.16	0.88

3.09

2.28

1.39

**TxWk1 TxWk2**

0.48

2.11

4.27

4.96

4.84

0.26

1.11

0.42	1.09	1.62

0.23	0.82	0.81

0.08	0.18	0.35

1.58	0.71	0.72

0.73

1.02	0.99

0.61

0.55

0.56

1.81

0

0

0

0

0

0

0

0

0

0

1.49

0.7	0.94

5.84

6.29	5.96

0.5

2.25

3.79

7.17

9.39

0.37

0.51

0.56

0.46

3.37

1.3

1.03

2.58

2.76

6.15

10.4

14.2

1.01

0.86	0.68

1.27

1.62

1.17

1.55	1.14

0.85

1.08

0.65

0.31

0.27

2.3

2.3

2.31

2.26

2.18

2.21

2.28

2.22

2.15

2.11

**TxEnh1 TxEnh2 TxEnh3 TxEnh4 TxEnh5 TxEnh6 TxEnh7 TxEnh8**

0.76

0.72

1.53

1.64

1.08

0.28	0.49

0.35	0.6

0.37	0.45

**EnhA20**

0.61

0.47

1.95

1.99

0.52

1.2

2.74

0.91

0.96

0.69

0.5

0.65

1.32

1.27

1.03

0.7

1.23

0.85

0.61

0.85

0.53

0.45

3.15

1.36

**EnhA17 EnhA18 EnhA19**

1.34

1.01

2.09

1.15

0.37	0.89

0.05	0.82

0.14	0.84

0.43	2.25

0.01	0.19

0	0.35

0.01	0.43

0.58	0.69

0.91

1.01

1.04

1.05	0.95

0.03	0.72	1.42

1.02

0.6

0.59

0.65

0.54

0.78

1.16

3.02

2.84

0.18

1.28

1.06

1.01

1

1.14

1.22

1.09

0.43

0.42

0.3

0.47

1.05

0.96

0.88

0.29	0.42

0.75

0.9

0.69

1.14

1.31

1.61

0.72

0.98

0.92

0.8

1.48	0.74

0.81	1

1.17	0.74

1.06	0.76

1.39

1.15

1.19

0.42	0.89

0.4	0.67

1	1.08

**EnhA10 EnhA11 EnhA12 EnhA13 EnhA14 EnhA15 EnhA16**

0.5

0.23

4.24

4.58

1.86

1.78

1.24

0.55

5.54

1.45

0.71

0.67

0.94	0.89

2.48

1.07

2.15	1.83

1.57	1.78

0.43

1.85

1.61

0.68

0.65

0.6

2.35

2.2

0.91

0.62

1.31

1.14

1.09

3.05

1.61

0.42

0.43

0.54

0.46

0.61

0.66

3.36

1.31

0.47	0.94

0.41	0.87

0.34	0.68

0.29	0.58

0.74	1.01

0.32

0.33

2.32

3.3

1.72

2.22

2.1

1.2

1.4

1.05

1.16

1.19

1.32

1.76

2.12

1.67

1.9

1.37

**EnhA1 EnhA2 EnhA3 EnhA4 EnhA5 EnhA6 EnhA7 EnhA8 EnhA9**

0.85

0.57

1.66

0.61

0.94

0.99

1.12

1.58

1.46

1.06

1

1.06

0.98	0.95

0.96	0.65

0.94	0.84

2.31

1.12

1.04

0.69

0.66

0.57

0.48

1.88

1.15

0.45

1.11

1.58

0.97

0.35

1.03

0.68

0.99

1.25

0.48	0.73

0.19	0.38

0.31	0.54

0.52	0.87

0.38	0.55

0.19	0.35

0.42

0.36

1.39

1.76

1.64

1.95	2.03

0.81

1.57

0.95

**EnhWk1 EnhWk2 EnhWk3 EnhWk4 EnhWk5 EnhWk6 EnhWk7 EnhWk8**

1.09

0.58

0.76

0.64

1.13

0.87

1.06

0.83

0.78	0.82

0.75	0.86

1.13	0.68

1.25	0.58

0.99

0.89

1.04

1.29

0.54

0.05	0.24

0.1	0.66

0.01	0.28

0	0.47

0.13	0.98

0.21	0.58

0.01	0.16

0.77	1.85

0.07	0.69

0.02	0.22

0.05	0.27

0.04	0.41

0.05	0.32

0.01	0.22

0.69	3.72

0.13	1.97

0.01	1.98

0	0.91

0.01	0.43

0.14	0.93

0.58	1.97

0.57	4.94

0.39	2.78

0.21	1.41

0.03	0.58

0.22	1.76

0.01	0.43

0.34

0.56

0.48

0.76

0.61

0.53

0.76

1.01

0.81

0.25	0.11	0.52

0.83

0.83

0.77	0.59

0.36	0.36

0.76	0.81

0.29	0.4

0.36	0.56

0.63	0.89

0.53	0.58

0.18	0.39

1.24

1.64

0.47

1.09

2.58	1.68

**Acet1 Acet2 Acet3 Acet4 Acet5 Acet6 Acet7 Acet8**

1.02

1.26

3.03

1.1

0.55

0.45

0.93

0.48

0.63

1.29

0.81

0.38

2.21

1.64

1.12

0.77

0.8

0.79

1.07	0.94

0.43	0.78

1.31	0.9	0.4

1.21

2.85

3.99

0.91

1.5

0.79

1.42	1.18

1.34	0.68

0.84

0.85

2.25	1.77

1.16

1.21

1

1.39

1.16

0.92

0.98

1.14

0.55

1.45

0.77

1.54

0.73

1.06

0.77

1.26

1.22

1.54

0.71

0.69

0.68

0.41

0.78

1.66	1.19

5.5

0.52	0.77

0	0.4

0.03	0.21

1.02	1.24

0.3	0.62

1.56	1.64

0.07	0.57

0.44	0.36

3.23	1.75

2.72

0.87

1.4

0.87

0.55

0.32

0.37

0.89

2.51

0

0

0

0

0

0

0

0

0

0

0

0

0

0

0

0

0

0

0

0

0

0

0

0

0

0

0

0

0

0

0

0

0

0

0

0

0

0

0

0

0

0

0

0

0

0

0

0

0

0

8.51

3.04

8.86

1.05	3.68

3.07

2.91

**ReprPC1 ReprPC2 ReprPC3 ReprPC4 ReprPC5 ReprPC6 ReprPC7 ReprPC8 ReprPC9**

3.53

0.51

0.02	0.44	0.6	0

0.75

0.42

1.44

1.49

1.78

1.63

1.57

0.4	0.64	0.43	0.41	0	0.32

0.6	0.47	0.71	0.91	0	0.47

0.68

0.64

0.36	0.35

0.89	0.72

0.35	0.37

2.58

2.04

0.87

1.44

0.3

0.28

0.36

0.29

0.38

0.32

0.61

0.37

0.53

1.09

0.37

0.4

0.38

0.32

0.46

0.39

0.31

0.59

0.66

0.72

0.59

0.6

0.72

0.5

0.42

0.73

0.8

0.82

0.54

0.55

0.91

0.5

0.71

0.89

0.92

0.73

0.31

0.27

0.34

0.42

0.41

0.94

0.49

1.12

0.77

1.14

1.95

3.15

1.47

1.42

1.14

1.28

1.22

1.71

1.27

2.24

1.74

1.64

2.7

2.17

1.71

1.91

1.29

2.61

2.45

2.6

2.17

2.85

1.28	0.86	0.65	0

0.63

0.48

0.86	0.67

14

1.58

1.66

2.52

0.59

0.46

1.28	0.65	0.86	0

1.42

0.8

1.76	1.95

6.03

1.35

0	0.08	0.21	0	0.33

0.74

0.57

0.23	0.47

1.86

1.87

0.72

0.38	0	0.06	0.17	0	0.31

0.61

0.72	0.58

0.42	1.25	0.54	0.52	0

0.79

0.79

1.41

0.95

0.47

0.2	0.29	0.53	0.28	0

0.55

**HET1 HET2 HET3 HET4 HET5 HET6 HET7 HET8 HET9**

1.26

1.21	0.85

0.06	0.17	0.41	0

0.7

1.73

1.46

0.35

0.09	0.11	0.21	0

0.59

1.24

0.82

0.07	0.14	0.44	0

0.76

0.28

0.62

1.01

1.18

1.19

0.58

1.03

0.42

0.52

0.19	0.02	0.06	0.13	0

0.22	0.04	0.19	0.17	0

0.54

0.56

0.05	0.09

0.15	0.17

0.22	0.39

0.07	0.12

0.22	0.33

0.43	0.34

1.01	0.79

0.08	0.16

**Quies1**

**Quies2 Quies3 Quies4 Quies5**

1.7

0.71

0.7

0.01

2.23

5.01	4.87

2.03

0.91	0.3

0.69

1.85

0.02

0.21

1.44

1.26

0.4

0.29

0.55

0.8

0.2

0.33

0.66

1.77

0.56

1.59

1.65

1.74

0.21

0.35

8.43

0.23	0.19	0.27	0.31	0.26	0.25	0.27

0.26	0.33	0.15	0.42	1.25	1.6	0.8

11.86

0.05

0.012

9.883

3.07

12.23

4.452

1.692

0.706

0.692

1.359

0.563

0.249

0.581

1.023

0.435

0.998

0.191

0.325

1.107

3.935

0.628

1.513

0.614

0.477

0.375

0.184

0.855

2.649

0.403

0.86

0.428

0.285

0.562

1.54

0.352

0.83

2.216

0.994

0.588

0.484

1.369

0.179

0.328

0.194

0.301

0.714

0.563

0.393

0.255

0.162

0.395

0.715

0.332

0.764

0.366

1.017

0.645

0.526

0.457

0.257

0.346

0.391

0.391

0.25

0.267

0.498

0.189

0.27

0.243

2.8

0.842

0.824

1.58

0.508

0.47

0.942

1.109

0.819

0.681

0.265

0.556

0.663

0.098

0.406

0.152

0.201

0.145

0.159

0.286

0.13

0.201

0.138

0.153

0.19

0.135

0.129

0.16

0.123

0.114

**GapArtf1 GapArtf2 GapArtf3**

8.1

**GapArtf1**

**GapArtf2 GapArtf3 Quies1 Quies2 Quies3 Quies4 Quies5 HET1 HET2 HET3 HET4 HET5 HET6 HET7 HET8 HET9**

**ReprPC1 ReprPC2 ReprPC3 ReprPC4 ReprPC5 ReprPC6 ReprPC7 ReprPC8 ReprPC9 Acet1 Acet2 Acet3 Acet4 Acet5 Acet6 Acet7 Acet8 EnhWk1 EnhWk2 EnhWk3 EnhWk4 EnhWk5 EnhWk6 EnhWk7 EnhWk8 EnhA1 EnhA2 EnhA3 EnhA4 EnhA5 EnhA6 EnhA7 EnhA8 EnhA9 EnhA10 EnhA11 EnhA12 EnhA13 EnhA14 EnhA15 EnhA16 EnhA17 EnhA18 EnhA19 EnhA20 TxEnh1 TxEnh2 TxEnh3 TxEnh4 TxEnh5 TxEnh6 TxEnh7 TxEnh8 TxWk1 TxWk2 Tx1**

**Tx2 Tx3 Tx4 Tx5 Tx6 Tx7 Tx8 TxEx1 TxEx2 TxEx3 TxEx4 znf1 znf2**

**DNase1**

4.885	0.38	0.39	0.55	0.69	0.57	0.53	0.58	19.9	0.44	3.33	1.29	0.37

0.053	0.24	0.3	0.14	0.39	1.18	1.45	0.72

0.012	0.86	0.84	0.28	1.98	4.59	4.25	2.05

10.66	0.04	0.08	0.5	0.18	0.02	0.06	0.12

3.314	0.14	0.16	0.52	0.21	0.03	0.18	0.16

0

0

0

0

0.8	1.59	1.4	1.37

0.89	1.67	1.39	1.24

0.66	0.4	0.95	0.37

0.55	0.5	1.1	0.27

13.17	0.21	0.36	0.94	0.71	0.08	0.12	0.41	0.02	0.76	1.17	1.11	0.52

4.795	0.07	0.11	0.55	0.27	0.08	0.11	0.19

1.82	0.21	0.3	0.66	0.59	0.05	0.16	0.38

0.762	0.4	0.32	0.51	0.18	0.27	0.5	0.26

0.746	0.94	0.73	0.75	0.38	1.17	0.5	0.48

1.466	0.07	0.15	0.74	0.35

0	0.07	0.16

0.606	0.21	0.43	0.52	0.69	0	0.08	0.2

0.267	1.65	1.83	0.75	1.33	1.17	0.62	0.81

0

0

0

0

0

0

0

0.32	1.41	1.6	0.75

1.13	0.78	1.17	0.19

0.43	0.91	1.31	0.31

0.66	0.55	0.57	0.62

0.29	0.69	1.72	1.65

0.28	1.3	1.73	5.66

0.49	2.39	1.46	13.1

0.627	0.8	0.62	0.45	0.58	1.13	0.83	0.61	0.99	0.37	2.75	1.52	2.41

1.103	0.34	0.33	0.63	0.38	0.59	0.41	0.38

0	0.28	1.57	1.64	1.9

0.47	0.83	0.67	0.59	0.56	0.43	0.64	0.84	0.66	0.42	1.49	1.33	0.81

1.069	0.33	0.35	0.39	0.71	0.02	0.41	0.56	0	0.46	3.41	1.38	1.35

0.206	2.71	2.84	0.98	3.37	7.93	2.82	7.89

0	2.34	0.85	0.35	0.28

0.353	0.81	1.29	0.8	2.5	0.74	0.71	5.08	0.88	1.54	1.18	0.72	0.26

1.194	0.52	0.63	0.65	0.98

0	0.38	1.06

4.243	0.3	0.38	0.64	0.71	0.03	0.19	0.51

0.677	2.11	1.65	0.79	1.17	0.94	1.15	1.34

1.631	1.33	1.09	0.79	1.13	0.28	0.58	0.72

0.662	3.73	2.65	1.13	1.43	1.45	1.51	1.43

0.514	1	0.88	0.74	0.71	0.06	0.53	0.68

0.403	0.4	0.73	0.73	1.54	0.4	0.33	2.81

0

0

0

0

0

0

0

1.09	1.34	0.94	0.34

0.87	1.16	1.08	0.27

0.91	1.31	0.63	0.36

0.73	1.45	0.85	0.3

1.21	0.86	0.38	0.57

1.25	1.02	1.04	0.35

1.04	1.19	0.95	0.5

0.204	2.37	1.55	1.05	0.46	3.1	1.57	0.97	3.04	0.81	1.54	1.04

1

0.922	0.72	0.55	0.77	0.23	0.11	0.49	0.51

2.856	0.34	0.34	0.77	0.32	0.05	0.23	0.42

0.434	0.71	0.75	0.92	0.51	0.1	0.63	0.87

0.927	0.27	0.37	0.82	0.44	0.01	0.26	0.45

0.462	0.34	0.52	0.96	0.7	0	0.43	0.59

0.307	0.59	0.82	1.06	0.58	0.11	0.91	1.2

0.606	0.49	0.54	0.71	0.49	0.18	0.53	0.74

1.661	0.17	0.36	0.88	0.7	0.01	0.15	0.35

0.38	1.82	1.89	1.53	1.63	0.73	1.7	2.05

0.895	0.44	0.68	1.16	0.95	0.07	0.65	1.07

2.39	0.17	0.35	1.05	0.63	0.01	0.2	0.42

1.069	0.29	0.5	0.97	0.92	0.05	0.27	1.04

0.721	0.47	0.78	1.25	1.37	0.11	0.39	1.36

0.523	0.35	0.51	1.04	0.91	0.04	0.3

0.9

1.476	0.18	0.33	0.87	0.58	0.01	0.21	0.32

0.193	1.28	1.76	1.57	1.95	0.65	3.47	3.07

0.354	0.43	0.87	1.22	1.11	0.11	1.84	1.6

0.209	0.38	0.81	1.5	1.3	0.01	1.84	2.07

0.324	0.32	0.64	1.06	0.97

0	0.83	0.84

0.77	0.27	0.53	1.01	1.07	0.01	0.39	0.57

0.607	0.7	0.93	1.51	1.1	0.13	0.87	1.22

0.423	2	1.71	1	1.2	0.55	1.79	2.3

0.274	1.46	1.66	1.35	1.63	0.51	4.61	5.15

0.175	1.16	1.66	1.73	1.95	0.36	2.6	3.96

0.425	0.39	0.83	1.11	1.3	0.18	1.31	4.27

0.77	0.38	0.62	0.91	1.05	0.04	0.52	1.21

0.358 0.94

1	0.85	0.84	0.2	1.63	1.49

0.824	0.27	0.39	0.81	0.65	0.01	0.41	0.67

0.395	1.02	1.13	1.07	1.19	0.18	2.64	2.81

1.097	0.4	0.56	0.95	0.99	0.03	0.66	1.32

0.695	0.39	0.55	0.84	0.94	0.38	0.83	1.95

0.567	0.28	0.61	1.27	0.91	0.04	0.77	1.07

0

0

0

0

0

0

0

0

0

0

0

0

0

0

0

0

0

0

0

0

0

0

0

0

0

0

0

0

0

0

0

0

0.75	1.03	0.94	0.34

0.5	0.97	1.2	0.37

0.73	0.78	0.78	0.36

0.71	0.82	0.99	0.3

1.06	0.64	0.81	0.43

1.19	0.55	0.6	0.37

0.54	0.85	1.02	0.29

1.47	0.65	0.76	0.55

1.29	0.33	0.4	0.62

1.78	0.55	0.65	0.68

2.18	0.46	0.63	0.55

0.91	0.91	0.99	0.56

0.85	1.83	0.88	0.59

0.89	0.8	1	0.46

1.57	0.54	0.8	0.39

2.18	0.23	0.31	0.69

3.19	0.5	0.41	0.75

2.88	0.39	0.42	0.77

2.22	0.57	0.57	0.5

2.07	0.62	0.65	0.52

1.75	0.41	0.62	0.85

0.87	0.87	0.67	0.47

1.18	0.52	0.63	0.66

1.35	0.22	0.48	0.83

1.39	0.72	0.76	0.86

0.76	0.93	1.06	0.68

1.1	0.73	0.71	0.29

1	0.73	0.9	0.26

1.09	0.71	0.73	0.32

0.98	0.91	0.95	0.4

0.94	1.26	0.97	0.38

2.98	0.42	0.51	0.88

0.497	0.43	0.5	0.78	0.58	0.23	0.79	0.78	1.25	1.16	0.67	0.96	0.45

0.277	0.99	1.19	1.24	0.84	0.39	2.08	1.85

0.373	0.26	0.45	1.01	0.9	0.01	0.17	0.47

0.422	0.32	0.56	2.15	0.64

0	0.34	1.11

0.422	0.34	0.41	2.16	0.46	0.01	0.41	2.55

0.269	1.45	1.06	2.16	0.6	0.51	0.63	1.18

0.288	13.3	9.69	2.11	5.69	2.4	1.22	1.51

0.537	8.78	6.66	2.04	3.52	2.57	0.96	1.09

0.204	5.89	5.54	2.07	5.41	0.65	0.87	1.38

0.291	0.95	0.92	2.14	0.68	0.38	1.01	1.5

0.262	4.52	4.59	2.08	3.95	0.2	0.76	0.75

3.019	1.3	2.12	2.01	2.87	0.08	0.17	0.33

0.909	6.2	4.46	1.97	2.32	1.46	0.66	0.66

0.888	0.18	0.2	2.16	0.27	0.01	0.16	0.63

1.704	0.17	0.23	2.13	0.33	0.04	0.14	0.45

0.548	0.28	0.33	2.12	0.27	0.2	0.36	0.59

0.507 2.48

2	2.16	1.3	0.04	0.39	0.61

1.016	2.7	2.66	2.07	2.46	0.45	0.21	0.41

1.196	3.37	4.08	2.12	4.56

0	0.14	0.23

0.884	6.37	5.41	2.14	4.58	0	0.27	0.34

0.735	2.35	3.26	2.06	3.72	0.02	0.32	0.53

0.286	9.69	6.01	2.17	3.63	0.54	1.2	1.22

0.6	14.8	9.27	2.17	3.79	1.06	0.68	0.71

0.716	9.11	6.54	2.1	3.95	2.5	0.56	0.73

0.106 10	7.28	2.12	8.75	1.81	3.83

0.438	0.99	1.14	1.99	1.31	0.28	0.22	0.37

0.164	9.69	6.63	2	3.04	0.92	0.85	0.78

0.216	0.93	1.41	1	2.28	3.89	5.19	1.75

**BivProm1**	0.156	8.78	7.89	1.33	2.36	70.4	26.7	17.9

**BivProm2**	0.172	7.04	5.92	1.23	3.72	49.6	13.7	13.8

**BivProm3**	0.308	2.76	2.6	1.08	2.06	6.76	5.73	6.52

**BivProm4**	0.14	2.38	3.15	1.62	2.78	3.42	7.52	13.5

**PromF1**	0.217	2.02	2.24	0.94	2.17	2.43	6.07	6.02

**PromF2**	0.149	2.24	2.99	1.29	4.09	3.49	7.58	12.9

**PromF3**	0.165	4.33	4.73	1.42	4.15	23.4	12.4	23.9

**PromF4**	0.205	9.54	12.5	1.76	2.34	88.3	57.9	26.1

**PromF5**	0.146	6.85	7.89	1.45	2.2	65.1	29.6	20.8

**PromF6**	0.139	3.45	3.02	1.96	3.41	1.45	2.56	24.6

**PromF7**	0.173	1.61	1.89	1.95	2.3	0.08	0.98	18.9

**TSS1**	0.132	6.08	11.7	1.34	2.82	93.9	141	24.2

**TSS2**	0.122	5.19	7.58	1.07	2.54	74.2	98.8	18.7

0

0

0

0

0

0

0

0

0

0

0

0

0

0

0

0

0

0

0

0

0

0

0

0

0

0

0

0

0

0

0

0

0

0

0

0

0

0

0

0

1.84	0.42	0.57	1.05

1.44	0.66	0.71	0.72

1.53	0.24	0.62	1.07

1.01	0.26	0.8	1.82

0.78	0.65	0.94	2.94

3.16	0.42	0.34	1.37

2.1	0.52	0.47	1.33

1.69	0.42	0.58	1.06

1.03	0.19	0.52	1.2

1.98	0.38	0.53	1.14

1.07	0.81	1.04	1.6

1.55	0.82	0.72	1.19

0.78	0.36	1.08	2.09

0.78	0.55	1.09	1.62

0.53	0.45	0.97	1.53

1.24	0.57	0.94	2.52

0.89	0.9	1.25	2.02

1.88	0.36	0.65	1.6

2.31	0.33	0.63	1.78

1.63	0.45	0.69	1.2

2.73	0.45	0.68	2.43

3.45	0.52	0.51	2.29

2.07	0.82	0.77	2.43

2.88	0.31	0.39	2.02

0.53	1.26	1.54	19.4

0.97	1.77	1.12	64

1.87	1.04	0.48	0.75

3.45	0.96	0.16	0.81

3.24	0.97	0.17	0.46

2.26	0.75	0.39	0.77

3.66	0.53	0.29	1.2

1.69	0.6

2.15	0.44

0.76

1.15

1.98	0.48	0.3	3.17

3.64	0.52	0.12	4.46

3.48	0.65	0.15	2.12

1.81	0.55	0.45	4.05

1.41	0.66	0.64	4.12

4.01	0.88	0.24	3.51

3.46	1.57	0.43	2

100 1.23	2.64	45	0	0.75	0	3.67 0.56 5.42 2.03 50.7 0.78

0.42

0.37

7.55

**state**

**% Genome**

**GENCODE_coding**

**RefSeqExon**

**RefSeqGene**

**RefSeqTES**

**CpGIsland**

**RefSeqTSS**

**RefSeqTSS2kb**

**assembly_gap**

**phastCons_elements**

**pseudogene**

**repeat_elements**

**znf_genes**

**state**

**% Genome**

**GENCODE_coding**

**RefSeqExon**

**RefSeqGene**

**RefSeqTES**

**CpGIsland**

**RefSeqTSS**

**RefSeqTSS2kb**

**assembly_gap**

**phastCons_elements**

**pseudogene**

**repeat_elements**

**znf_genes**

**Corr. Enr. Hg19 &hg38**

**Avg. acros**

| 1 | 1 | 1 | 1 | 1 | 1 | 1 | 0.98 | 0.99 | 0.93 | 0.97 | 1 | **0.99** |
| --- | --- | --- | --- | --- | --- | --- | --- | --- | --- | --- | --- | --- |

**Supplementary Figure 18: Comparison of hg19 and hg38 full-stack states enrichments with annotated genomic contexts.** The heatmaps show enrichment values for the full-stack states (rows) and different external genome annotations from Fig. 3A (columns) in hg19 (A) and hg38 (B) (**Methods**). Panel

**(A)** is similar to **Fig. 3A**, but we present it here for better comparison with the hg38 enrichment heatmap. Results in **(B)** are based on (1) lifting over the full-stack annotation from hg19 to hg38 (**Methods**), and (2) doing enrichment analysis with annotated genome contexts derived from various databases in hg38 (**Methods**). In each heatmap, coloring of enrichments is column specific with highest and lowest enrichment values in each column are colored red and white, respectively. The first two columns of each heatmap show state labels and their percentage of genome coverage. The last row of each heatmap shows the percentage of genome coverage for each type of genome contexts. Below the heatmap in **(B)** is the correlation of the enrichments across states based on hg19 and hg38 for each corresponding annotation column as well as the average of them.

We also added a sentence to the results section summarizing these results:

“We verified that enrichments computed based on hg19 were highly similar to those computed for full- stack annotations mapped to hg38 (average correlation 0.99; **Methods, Supp. Fig. 18)**, ensuring the applicability of state annotations in hg38.”

In response to this comment and a comment from reviewer 1 we have revised the methods section ‘Lifting chromatin state annotations to hg38’, which now reads:

“The full stack chromatin state annotations were learned directly in hg19, as this was the assembly for which uniformly processed data from the Roadmap Epigenomics integrative analysis was available. Learning full-stack annotation directly in hg19 allowed direct comparison with existing per-cell-type annotations inferred. We also generated a version of full-stack annotation in hg38 by lifting over the original annotation from hg19 to hg38. To do this, we first wrote the hg19 chromatin state annotation into .bed format such that each line corresponds to a 200bp interval. We then used the liftOver tool [14] with default parameters to generate the annotation in hg38. We did not annotate bases in hg38, if multiple bases in hg19 mapped to it. In total, there are 1,186,379 200-bp segments that were not mapped from hg19 to hg38, of which 98.7% fall into an assembly gap and 99.6% fall into the full-stack state primarily associated assembly gaps (GapArtf1) (**Supp. Fig. 50**). In hg38 on chr1-22, X, and Y, 92.9% of bases are annotated to a state, and that number increases to 97.1% when excluding assembly gaps. We verified that we saw highly similar state fold enrichments for similar annotations between hg19 and hg38 (**Supp. Fig. 18**). The sources of external annotations from hg38 are outlined in section “External annotation sources” below.”

We have also added a **Supplementary Figure** showing the state enrichments of the full-stack states with bases that were unmapped to hg38, and providing additional information about them with respect to annotated assembly gaps:

## A

| **state** | **percent_in_genome** | **unmapped_hg19 _to_hg38** |  | **percent_unmapped_in_stat** |
| --- | --- | --- | --- | --- |
| GapArtf1 | 11.86 | 8.40 |  | 99.59 |
| GapArtf2 | 0.05 | 0.14 |  | 0.01 |
| GapArtf3 | 0.01 | 0.35 |  | 0.00 |
| Quies1 | 9.88 | 0.00 |  | 0.02 |
| Quies2 | 3.07 | 0.00 |  | 0.00 |
| Quies3 | 12.23 | 0.01 |  | 0.09 |
| Quies4 | 4.45 | 0.01 |  | 0.05 |
| Quies5 | 1.69 | 0.01 |  | 0.02 |
| HET1 | 0.71 | 0.00 |  | 0.00 |
| HET2 | 0.69 | 0.01 |  | 0.00 |
| HET3 | 1.36 | 0.00 |  | 0.00 |
| HET4 | 0.56 | 0.01 |  | 0.01 |
| HET5 | 0.25 | 0.01 |  | 0.00 |
| HET6 | 0.58 | 0.01 |  | 0.01 |
| HET7 | 1.02 | 0.01 |  | 0.01 |
| HET8 | 0.43 | 0.01 |  | 0.00 |
| HET9 | 1.00 | 0.02 |  | 0.02 |
| ReprPC1 | 0.19 | 0.01 |  | 0.00 |
| ReprPC2 | 0.32 | 0.01 |  | 0.00 |
| ReprPC3 | 1.11 | 0.00 |  | 0.00 |
| ReprPC4 | 3.93 | 0.00 |  | 0.01 |
| ReprPC5 | 0.63 | 0.00 |  | 0.00 |
| ReprPC6 | 1.51 | 0.00 |  | 0.01 |
| ReprPC7 | 0.61 | 0.00 |  | 0.00 |
| ReprPC8 | 0.48 | 0.01 |  | 0.00 |
| ReprPC9 | 0.37 | 0.01 |  | 0.00 |
| Acet1 | 0.18 | 0.02 |  | 0.00 |
| Acet2 | 0.85 | 0.00 |  | 0.00 |
| Acet3 | 2.65 | 0.00 |  | 0.01 |
| Acet4 | 0.40 | 0.00 |  | 0.00 |
| Acet5 | 0.86 | 0.00 |  | 0.00 |
| Acet6 | 0.43 | 0.00 |  | 0.00 |
| Acet7 | 0.28 | 0.00 |  | 0.00 |
| Acet8 | 0.56 | 0.00 |  | 0.00 |
| EnhWk1 | 1.54 | 0.00 |  | 0.00 |
| EnhWk2 | 0.35 | 0.00 |  | 0.00 |
| EnhWk3 | 0.83 | 0.00 |  | 0.00 |
| EnhWk4 | 2.22 | 0.00 |  | 0.00 |
| EnhWk5 | 0.99 | 0.01 |  | 0.01 |
| EnhWk6 | 0.59 | 0.01 |  | 0.01 |
| EnhWk7 | 0.48 | 0.00 |  | 0.00 |
| EnhWk8 | 1.37 | 0.00 |  | 0.00 |
| EnhA1 | 0.18 | 0.00 |  | 0.00 |
| EnhA2 | 0.33 | 0.00 |  | 0.00 |
| EnhA3 | 0.19 | 0.00 |  | 0.00 |
| EnhA4 | 0.30 | 0.00 |  | 0.00 |
| EnhA5 | 0.71 | 0.00 |  | 0.00 |
| EnhA6 | 0.56 | 0.00 |  | 0.00 |
| EnhA7 | 0.39 | 0.00 |  | 0.00 |
| EnhA8 | 0.25 | 0.01 |  | 0.00 |
| EnhA9 | 0.16 | 0.00 |  | 0.00 |
| EnhA10 | 0.39 | 0.00 |  | 0.00 |
| EnhA11 | 0.72 | 0.01 |  | 0.00 |
| EnhA12 | 0.33 | 0.00 |  | 0.00 |
| EnhA13 | 0.76 | 0.00 |  | 0.00 |
| EnhA14 | 0.37 | 0.00 |  | 0.00 |
| EnhA15 | 1.02 | 0.00 |  | 0.00 |
| EnhA16 | 0.65 | 0.01 |  | 0.01 |
| EnhA17 | 0.53 | 0.00 |  | 0.00 |
| EnhA18 | 0.46 | 0.00 |  | 0.00 |
| EnhA19 | 0.26 | 0.00 |  | 0.00 |
| EnhA20 | 0.35 | 0.00 |  | 0.00 |
| TxEnh1 | 0.39 | 0.00 |  | 0.00 |
| TxEnh2 | 0.39 | 0.00 |  | 0.00 |
| TxEnh3 | 0.25 | 0.00 |  | 0.00 |
| TxEnh4 | 0.27 | 0.00 |  | 0.00 |
| TxEnh5 | 0.50 | 0.00 |  | 0.00 |
| TxEnh6 | 0.19 | 0.00 |  | 0.00 |
| TxEnh7 | 0.27 | 0.00 |  | 0.00 |
| TxEnh8 | 0.24 | 0.00 |  | 0.00 |
| TxWk1 | 2.80 | 0.00 |  | 0.01 |
| TxWk2 | 0.84 | 0.00 |  | 0.00 |
| Tx1 | 0.82 | 0.00 |  | 0.00 |
| Tx2 | 1.58 | 0.00 |  | 0.00 |
| Tx3 | 0.51 | 0.00 |  | 0.00 |
| Tx4 | 0.47 | 0.00 |  | 0.00 |
| Tx5 | 0.94 | 0.00 |  | 0.00 |
| Tx6 | 1.11 | 0.00 |  | 0.00 |
| Tx7 | 0.82 | 0.00 |  | 0.00 |
| Tx8 | 0.68 | 0.00 |  | 0.00 |
| TxEx1 | 0.27 | 0.00 |  | 0.00 |
| TxEx2 | 0.56 | 0.00 |  | 0.00 |
| TxEx3 | 0.66 | 0.00 |  | 0.00 |
| TxEx4 | 0.10 | 0.00 |  | 0.00 |
| znf1 | 0.41 | 0.00 |  | 0.00 |
| znf2 | 0.15 | 0.00 |  | 0.00 |
| DNase1 | 0.20 | 0.01 |  | 0.00 |
| BivProm1 | 0.15 | 0.01 |  | 0.00 |
| BivProm2 | 0.16 | 0.01 |  | 0.00 |
| BivProm3 | 0.29 | 0.01 |  | 0.00 |
| BivProm4 | 0.13 | 0.01 |  | 0.00 |
| PromF1 | 0.20 | 0.00 |  | 0.00 |
| PromF2 | 0.14 | 0.00 |  | 0.00 |
| PromF3 | 0.15 | 0.00 |  | 0.00 |
| PromF4 | 0.19 | 0.00 |  | 0.00 |
| PromF5 | 0.14 | 0.00 |  | 0.00 |
| PromF6 | 0.13 | 0.00 |  | 0.00 |
| PromF7 | 0.16 | 0.00 |  | 0.00 |
| TSS1 | 0.12 | 0.01 |  | 0.00 |
| TSS2 | 0.11 | 0.03 |  | 0.00 |

**percent in genome**

100	7.665	100

**Enrichment of full-stack states with bases in hg19 that were unmapped to hg38, and analysis of unmapped bases with assembly gaps (all analyses in chr1-22,X,Y)**

## B

| # bp in hg38 not mapped to a state | 218,892,096 |
| --- | --- |
| # bp in hg38 not mapped to a state AND overlapping hg38  assembly gaps | 134,483,364 |
| Fraction of hg38 bases that are unannotated to a state after  liftOver from hg19 AND overlap hg38 assembly gaps | 0.61 |
| # bp in hg19 unmapped to hg38 | 237,275,800 |
| # bp in hg19 unmapped to hg38 AND overlapping assembly gaps | 234,342,292 |
| Fraction of unmapped_to_hg38 bp in hg19 that also overlap  hg19 assembly gaps | 0.99 |
| # bp in hg38 that are not assembly gaps | 2,937,659,104 |
| # bp in hg38 that are not assembly gaps AND annotated as a  state after liftOver | 2,853,200,372 |
| Fraction of hg38 bases that are non-assembly gaps AND  annotated to a state after liftOver | 0.97 |

**Supplementary Figure 50: Full-stack states enrichments of bases that were not lifted over from hg19 to hg38. (A)** The heatmap shows enrichment values for the full-stack states (rows) of genomic bases that were unmapped when lifting the state annotation from hg19 to hg38 (Methods). The first column shows the state label and the second column shows the percentage of the genome that each state covers. The third column shows enrichment values, colored such that highest enrichment values are colored red and lowest ones are colored white. The fourth column shows the percentage of the unmapped regions (from hg19 to hg38) in each state. **(B)** Table showing details of numbers of bases involved in liftOver procedure, highlighting the overlap between the unmapped and unannotated regions with assembly gaps. As part of the liftOver procedure, bases in hg38 that are mapped to from multiple bases in hg19 are left unannotated to any state in hg38 (Methods). All results are reported in chromosomes 1-22, X, Y.

*Minor changes:*

*Line 57-60: "Variants of this approach allow information from other cell types to influence the state annotations in one cell type at a position, but still produce cell-type-specific state annotations [26,27]. These models that produce cell-type-specific annotations are natural for cell-type-specific analyses." These sentences are confusing. What do you mean by "other cell types to influence one cell type ... still produce cell type-specific annotations"? What is "natural" about the analyses?*

We thank the reviewer for pointing out these sentences were unclear and we apologize for that. By “cell- type-specific annotations” we mean that the methods produce per-cell type annotation, i.e. a chromatin state for each cell type. Methods such as TreeHMM [38] and IDEAS [39] are similar to the concatenated ChromHMM in that their goal is to produce per-cell type annotations. These methods differ from concatenated ChromHMM in that they use data from other cell types at the same genomic position as a way to try to reduce the effect of noise when producing per-cell type annotations.

By “natural” we mean that if a researcher is interested in a specific cell type then we expect focusing on annotations that are for that cell type specifically will be the preferred and effective analysis approach.

We rephrased these sentences to clarify on these points, which now read as follows:

“Variants of the concatenated approach attempt to use information from related cell types to reduce the effect of noise, but still output per-cell-type annotations [38,39]. These models that produce per-cell-type annotations tend to be most appropriate in studies where researchers are interested in studying individual cell types.”

*Line 80-83: The authors compared the number of states (100) in the full-stack model to the number of states theoretically possible (M^K or even M*K) in a concatenated model, where M = 15 or 18 and K = 127. This comparison indicates that the total number of states in the full-stack model (100) is very small. Does that mean that most of the states in the concatenated models are not occupied? Can the authors show this using the enhancer states as an example? How many genomic bins belong to one of the enhancer states in the concatenated model and which states do these genomic bins take on in the full-stack model?*

We thank the reviewer for these questions. In the concatenated models, while in any one cell type every state will be occupied, when considering combinations of state assignments across a large number of cell types most combinations will be unoccupied. For example, for a concatenated model with 25 states that annotates 127 cell types, there are 3.5E177 (25^127^) combinations of state assignments, while there are only about 1.5E7 200bp windows in the genome. It thus follows that most combinations will not be occupied,

but there will likely be many more than 100 combinations of chromatin states that will be sparsely occupied. Furthermore, the difference between many of these combinations, which might differ by a single chromatin state assignment in one cell type, could be explained by noise.

Here, we wanted to indicate that if a researcher wants to analyze the set of chromatin state assignments at a single genomic position across the cell types with per-cell-type annotations from a concatenated model, they will need to reason about the M possible state assignments per cell type, across the K cell types. The full-stack annotation offers an alternative approach that can be more straightforward for such scenarios. Researchers can simply look up one full-stack state annotation at the position of interest. Of the subset of the 𝑀^𝐾^ possible combinations of chromatin state assignments across the K cell types, many will be sparsely present in the genome, and thus difficult to associate with any meaningful biological phenomenon. The full-stack annotation has advantages in this scenario, as it offers a systematic annotation of the epigenomic landscapes into 100 states that show various constitutive or cell-type-specific patterns, none of which are sparsely occupied.

We have revised this part of the introduction to try to make it clearer and now state:

“Third, if one tries to analyze per-cell-type annotations across cell types, one would need a post- hoc method to reason about an exponentially large number of possible combinations of chromatin states across cell types (if each of 𝐾 cell types has 𝑀 states, there are 𝑀^𝐾^ possible combinations of states for a genomic position) many of which would likely lack biologically meaningful distinctions. In contrast, for the stacked model, there will be a single annotation per position out of a possibly much smaller fixed number of states (compared to 𝑀^𝐾^). These states are directly informative of cross-cell type activity, though the state definitions can be more complex.”

Individual active and flanking enhancers states have a median genome-wide coverage of 0.22-0.47% across cell types (% reported for all states **Supp. Data. File 3**). We show median enrichments of all per-cell type concatenated model chromatin states, including enhancer states, for the stacked model states in **Supp. Data. File 3**.

*The authors described the improvement on scalability for ChromHMM which makes full-stacked modeling possible. How does computing time grow with the number of input tracks? How does computing time grow with genome size?*

We thank the reviewer for the questions. We have now included an asymptotic worst time and memory analysis that we have added to the **Supplementary Information**. Worst case compute time per training iteration will grow linearly with the total length of the genome sampled per iteration. When the number of input tracks are sufficiently large, the worst-case compute time will also grow linearly in the number of input tracks.

The full details can be found in the Supplementary Information. We added a sentence to the methods referring the reader to the Supplementary Information on this:

“The asymptotic worst-case time and memory of the model learning is discussed in the **Supplementary Information.**”

*The assembly gap states seem to be new for the full-stack modeling. Why were such states not identified previous by concatenated modeling?*

We thank the reviewer for this question. In previous concatenated models, the reason an assembly gap state did not appear was likely that there were too few input features given the number of states in the models. In such models the assembly gaps typically were incorporated into states that have low signal for most marks (e.g. ‘Quiescent’ state). We do note that in the original paper presenting the chromatin state modeling approach that ChromHMM utilizes [8], which was based on 41 inputs tracks and 51-chromatin states in human CD4 T-cells an ‘unmappable’ state was described, which also largely captured assembly gaps.

In response to this point we have revised the text to state:

“In previous concatenated models based on the Roadmap Epigenomics data [3], no specific state was associated with assembly gaps likely due to the limited number of input chromatin mark signals compared to the number of states, leading to assembly gaps to be incorporated in the general quiescent state.”

*Line 148-149: "States in the transcription groups (TxEnh1-8, TxWk1-2, Tx1- 8, TxEx1-4) were all at least*

*2.1 fold enriched for annotated genes, which covered 88.8-97.5% of the states." What do you mean by "which"?*

We thank the reviewer for the question and apologize that the sentence was not clear. Here, ‘which’ refers to annotated gene bodies. We have revised the sentence to now state:

“States in the transcription groups (TxEnh1-8, TxWk1-2, Tx1-8, TxEx1-4) were all at least 2.1 fold enriched for annotated gene bodies; these genes bodies covered 88.8–97.5% of the states.”

*By annotated genes, I assume you mean exon plus intron positions. Which genome annotation are you using?*

We thank the reviewer for this question. Yes, annotated genes include exons and introns. We used the gene annotations included in ChromHMM, which are RefSeq annotations available through UCSC Genome Browser on July 26th, 2015. We updated the sentence about RefSeq annotations in section ‘Methods/External annotation sources’ as follows:

“Annotations of CpG islands, exon, gene bodies (exons and introns), transcription start (TSS), and transcription end sites (TES), 2kb windows surrounding TSSs (TSS2kb) in hg19 and hg38 were RefSeq annotations included in ChromHMM (v1.18) and originally based on annotations obtained from the UCSC genome browser on July 26th 2015.”

We also edited Fig. 3 legend to state:

“gene bodies (exons and introns)”

*In total, what fraction of the genome does "annotated genes" cover?*

We thank the reviewer for this question. For this analysis, annotated genes cover 42.1% of the genome. We include information about the percentage of the genome that each external genomic context occupies in the last row of Fig.3, and have revised the legends to highlight that. We present the updated **Fig. 3A** legend after the comment below.

*How is the color scale in Fig 3A defined? It seems to differ across the columns; some small enrichments (like 2.2 in RefSeqGene column) are in bright red while other even higher enrichments (like 62.2 TSS enrichment in PromF4) are in light red.*

We thank the reviewer for the question. In **Fig. 3A**, each column is on a separate color scale. Specifically, we color each column such that the cell with highest enrichment value in the column will be colored red and the cell with the smallest enrichment values will be colored white. We have revised our wording in this figure legend to make that clearer. We show below the revised **Fig. 3A** legend.

“**Figure 3: Full stack states enrichments for external genomic annotations. (A)** Fold enrichments of full-stack states with external genome annotations (**Methods**). Each row corresponds to a state and each column corresponds to one external genomic annotation: CpG Islands, Exons, coding sequences, gene bodies (exon and introns), transcription end sites (TES), transcription start sites (TSS), TSS and 2kb surrounding regions, lamina associated domains (laminB1lads), assembly gaps, annotated ZNF genes, repeat elements and PhastCons constrained element (**Methods**). The last row shows the percentage of the genome that each external genome annotation covers. The heatmap colors are column-normalized, i.e. within each column, the color of the cells are such that highest values are colored red and lowest values are colored white.”

*Line 223: the cited supplementary figure seems wrong. Should it be Fig. S20 & S21?*

We thank the reviewer for pointing that out. We have updated the numbering of figures throughout the paper to also incorporate all the changes we made in response to reviewers’ comments.

1. Ernst J, Kellis M. ChromHMM: automating chromatin-state discovery and characterization. Nat Methods. Nature Publishing Group; 2012;9:215–6.
2. Ernst J, Kellis M. Chromatin-state discovery and genome annotation with ChromHMM. Nat Protoc. Nature Publishing Group; 2017;12:2478.
3. Kundaje A, Meuleman W, Ernst J, Bilenky M, Yen A, Heravi-Moussavi A, et al. Integrative analysis of 111 reference human epigenomes. Nature. Nature Publishing Group; 2015;518:317–30.
4. Ernst J, Kheradpour P, Mikkelsen TS, Shoresh N, Ward LD, Epstein CB, et al. Mapping and analysis of chromatin state dynamics in nine human cell types. Nature. Nature Publishing Group; 2011;473:43–9.
5. Guelen L, Pagie L, Brasset E, Meuleman W, Faza MB, Talhout W, et al. Domain organization of human chromosomes revealed by mapping of nuclear lamina interactions. Nature. Nature Publishing Group; 2008;453:948–51.
6. Sakamoto Y, Ishiguro M, Kitagawa G. Akaike information criterion statistics. Dordr Neth Reidel. Taylor & Francis; 1986;81:26853.
7. Neath AA, Cavanaugh JE. The Bayesian information criterion: background, derivation, and applications. Wiley Interdiscip Rev Comput Stat. Wiley Online Library; 2012;4:199–203.
8. Ernst J, Kellis M. Discovery and characterization of chromatin states for systematic annotation of the human genome. Nat Biotechnol. Nature Publishing Group; 2010;28:817–25.
9. Ernst J, Kellis M. Large-scale imputation of epigenomic datasets for systematic annotation of diverse human tissues. Nat Biotechnol. Nature Publishing Group; 2015;33:364–76.
10. Stunnenberg HG, Abrignani S, Adams D, de Almeida M, Altucci L, Amin V, et al. The International Human Epigenome Consortium: a blueprint for scientific collaboration and discovery. Cell. Elsevier; 2016;167:1145–9.
11. Li CZ, Haghani A, Robeck TR, Villar D, Lu AT, Zhang J, et al. Epigenetic predictors of maximum lifespan and other life history traits in mammals. bioRxiv. Cold Spring Harbor Laboratory; 2021;
12. Horvath S, Haghani A, Peng S, Hales EN, Zoller JA, Raj K, et al. DNA methylation aging and transcriptomic studies in horses. Biorxiv. Cold Spring Harbor Laboratory; 2021;
13. Arneson A, Haghani A, Thompson MJ, Pellegrini M, Kwon SB, Vu HT, et al. A mammalian methylation array for profiling methylation levels at conserved sequences. Biorxiv. Cold Spring Harbor Laboratory; 2021;
14. Kent WJ, Sugnet CW, Furey TS, Roskin KM, Pringle TH, Zahler AM, et al. The human genome browser at UCSC. Genome Res. Cold Spring Harbor Lab; 2002;12:996–1006.
15. Bernstein BE, Mikkelsen TS, Xie X, Kamal M, Huebert DJ, Cuff J, et al. A bivalent chromatin structure marks key developmental genes in embryonic stem cells. Cell. Elsevier; 2006;125:315–26.
16. McLean CY, Bristor D, Hiller M, Clarke SL, Schaar BT, Lowe CB, et al. GREAT improves functional interpretation of cis-regulatory regions. Nat Biotechnol. Nature Publishing Group; 2010;28:495–501.
17. Hoffman MM, Ernst J, Wilder SP, Kundaje A, Harris RS, Libbrecht M, et al. Integrative annotation of chromatin elements from ENCODE data. Nucleic Acids Res. Oxford University Press; 2013;41:827–41.
18. Dixon JR, Selvaraj S, Yue F, Kim A, Li Y, Shen Y, et al. Topological domains in mammalian genomes identified by analysis of chromatin interactions. Nature. Nature Publishing Group; 2012;485:376–80.
19. Phillips JE, Corces VG. CTCF: Master Weaver of the Genome. Cell. 2009;137:1194–211.
20. Wang Y, Liu Y, Xu Q, Xu Y, Cao K, Deng N, et al. TAD boundary and strength prediction by integrating sequence and epigenetic profile information. Brief Bioinform. 2021;
21. McArthur E, Capra JA. Topologically associating domain boundaries that are stable across diverse cell types are evolutionarily constrained and enriched for heritability. Am J Hum Genet. Elsevier; 2021;108:269–83.
22. Dib C, Fauré S, Fizames C, Samson D, Drouot N, Vignal A, et al. A comprehensive genetic map of the human genome based on 5,264 microsatellites. Nature. Nature Publishing Group; 1996;380:152–4.
23. Lowe TM, Eddy SR. tRNAscan-SE: a program for improved detection of transfer RNA genes in genomic sequence. Nucleic Acids Res. Oxford University Press; 1997;25:955–64.
24. Navarro Gonzalez J, Zweig AS, Speir ML, Schmelter D, Rosenbloom KR, Raney BJ, et al. The UCSC genome browser database: 2021 update. Nucleic Acids Res. Oxford University Press; 2021;49:D1046–57.
25. Elbarbary RA, Lucas BA, Maquat LE. Retrotransposons as regulators of gene expression. Science. American Association for the Advancement of Science; 2016;351.
26. Pehrsson EC, Choudhary MN, Sundaram V, Wang T. The epigenomic landscape of transposable elements across normal human development and anatomy. Nat Commun. Nature Publishing Group; 2019;10:1–16.
27. Arneson A, Ernst J. Systematic discovery of conservation states for single-nucleotide annotation of the human genome. Commun Biol. Nature Publishing Group; 2019;2:1–14.
28. Abel HJ, Larson DE, Regier AA, Chiang C, Das I, Kanchi KL, et al. Mapping and characterization of structural variation in 17,795 human genomes. Nature. Nature Publishing Group; 2020;583:83–9.
29. Karczewski KJ, Francioli LC, Tiao G, Cummings BB, Alföldi J, Wang Q, et al. The mutational constraint spectrum quantified from variation in 141,456 humans. Nature. Nature Publishing Group; 2020;581:434–43.
30. Ioannidis NM, Davis JR, DeGorter MK, Larson NB, McDonnell SK, French AJ, et al. FIRE: functional inference of genetic variants that regulate gene expression. Bioinformatics. Oxford University Press; 2017;33:3895–901.
31. Schuster-Böckler B, Lehner B. Chromatin organization is a major influence on regional mutation rates in human cancer cells. nature. Nature Publishing Group; 2012;488:504–7.
32. Parker SC, Gartner J, Cardenas-Navia I, Wei X, Abaan HO, Ajay SS, et al. Mutational signatures of de-differentiation in functional non-coding regions of melanoma genomes. PLoS Genet. Public Library of Science; 2012;8:e1002871.
33. Polak P, Karlić R, Koren A, Thurman R, Sandstrom R, Lawrence MS, et al. Cell-of-origin chromatin organization shapes the mutational landscape of cancer. Nature. Nature Publishing Group; 2015;518:360– 4.
34. Martincorena I, Campbell PJ. Somatic mutation in cancer and normal cells. Science. American Association for the Advancement of Science; 2015;349:1483–9.
35. Supek F, Lehner B. Differential DNA mismatch repair underlies mutation rate variation across the human genome. Nature. Nature Publishing Group; 2015;521:81–4.
36. Jones PA, Takai D. The role of DNA methylation in mammalian epigenetics. Science. American Association for the Advancement of Science; 2001;293:1068–70.
37. Weber M, Hellmann I, Stadler MB, Ramos L, Pääbo S, Rebhan M, et al. Distribution, silencing potential and evolutionary impact of promoter DNA methylation in the human genome. Nat Genet. Nature Publishing Group; 2007;39:457–66.
38. Biesinger J, Wang Y, Xie X. Discovering and mapping chromatin states using a tree hidden Markov model. BMC Bioinformatics. Springer; 2013. p. S4.
39. Zhang Y, An L, Yue F, Hardison RC. Jointly characterizing epigenetic dynamics across multiple human cell types. Nucleic Acids Res. Oxford University Press; 2016;44:6721–31.

**Second round of review**

**Reviewer 1**

Manuscript review, revision I
Title: Universal annotation of the human genome through integration of over a thousand epigenomic datasets
Authors: Vu & Ernst
Journal: Genome Biology

----------------------------------------------------------------------------------------------

The authors have put in significant effort to address the concerns of both reviewers, which is appreciated.
With the exception of a few relatively minor items, the authors have addressed my concerns to my satisfaction.
Below, I briefly comment on each response of the authors point-by-point.

1. Stacked model learning
OK. Thanks for clarifying and commenting on the sampling approach.
2. Terminology of non-stacked models
I appreciate the line of reasoning and explanation provided by the authors, however I want to push on this
a bit more since the use of "cell-type-specific" does not resolve my concerns.
The term 'cell-type-specific' introduces multiple levels of ambiguity, namely:
- the meaning of "cell type", which the authors mention also includes "tissue type", and likely
"biological condition", "cellular state", "biosample", etcetera
- the meaning of "specific", suggesting these annotations are "exclusive" to a given cellular context,
which is hardly ever the case.
Instead, I suggest using a simple but concrete term for each type of model /and/ annotation, namely:
"independent", "concatenated", and "stacked".
The latter two immediately suggest these are based on multiple biosamples.
Having three clear single-word terms to refer to both models and associated annotations will
dramatically increase the readability of the manuscript.

3. Benefits of stacked models
Unfortunately, the explanation concerning "a systematic and global view of the data" provided by the
authors does not fully clarify things for me.
Would clustering based on all states provide a "systematic and global view of the data"?
Can the authors be more explicit on what "unbiased", "systematic" and "global" mean in this context?

Thank you for clarifying some of the benefits of stacked models relative to more conventional approaches,
and confirming the LMNB1 vs. fibroblast suspicion and incorporating this finding into the manuscript.
I appreciate the changes made to the text and supplementary figures.

4. Stacked vs. concatenated models for characterizing dynamic state behavior
I'd like to note that the "x out of n samples are in state i" example was merely that: an example.
Naturally there are more sophisticated ways to undertake such analyses, which could also account for
imbalanced cell type representations, state similarities, etcetera.
That said, with the changes made to the manuscript the authors now make a more convincing case for
using a stacked model.

5. Choice of number of chromatin states
Thanks to the authors for addressing this point in detail.
I was hoping to see more of a general recommendation for future stacked model users to choose a
reasonable number of states for their purpose, but agree with the authors that this is often
dependent on the type of application.
I appreciate the addition of new Supplemental figures to illustrate the choice of number of states.
Unfortunately the text in the new Supp. Figure 3B is not legible, in particular the x-axis.
The analysis presented in the new Supp. Figure 3C is a bit unfair: by virtue of comparing models with
different state numbers to a mode with 100 states, the 100-state model will always look favorable.
It does little in terms of saying anything about the quality of the 100-state model;
rather, it merely says something about how similar other models are to a 100-state model.
The analysis presented in the new Supp. Figure 3D is more interesting in my opinion.
Although limited to the H3K4me1 mark, this is a relevant mark to consider in this case.
It would benefit the figure and interpretation if the authors would indicate below each
column how often its values were row-wise maximal.

6. The role of data imputation
I appreciate, and agree with, the comments made by the authors and would only advise to include a
brief remark on the technical concern of artificially high correlation structures.

7. Extendability to new datasets and establishing standard annotations
I agree with the authors' points, and would suggest that the authors more explicitly argue that perhaps
the stacked models may provide a more universal set of annotations for use in e.g. genome browsers.

In conclusion:
The authors have put in significant effort to improve the manuscript in terms of relevance for
Biological interpretation, and have addressed most of my (major and minor) concerns to satisfaction.
The manuscript can still be improved in terms of readability and terminology,
to which I have made some suggestions above.

**Reviewer 2**

The authors have addressed all the questions, significantly improving the manuscript. I am in support of this manuscript being accepted for publication given that the minor comments below are addressed.

Response to major comments: 1. It is the underlying assumption of ChromHMM that each genomic bin can only be assigned to one chromatin state. When we consider the concatenated model, for the genomic bins that are in one state (say, TSS, the gene is expressed) in one sample but in another state in another sample (say, ReprPC, the gene is repressed), and in a third state in yet another sample (say, Bivalent TSS, the gene is poised for expression), what would this genomic bin be assigned to in the full-stack model? Can the authors perform a detailed analysis on this subset of genomic bins so that the reader can get a better understanding of the full-stack model? *Well addressed*

2. In Fig1, the authors showed two loci of full-stack modeling annotations. It is indeed an advantage of the full-stack model to provide a big-picture view of the epigenomes, as illustrated by these two loci. However, there are many genomic regions with frequent state transitions as shown by the concatenated approach; can the authors provide two examples of such regions and illustrate their state assignment in the full-stack model? *Well addressed. It is great to have these examples*.

3. Are different TSS states (TSS1-2, BivProm1-4) in the full-stack model associated with different classes of genes? Can the author perform GO enrichment for each subset of genes? *Thank you for providing the GO enrichment results. However, the top enriched GO terms in each state are likely to be paternal GO nodes, which didn’t show biological significance (as “biological regulation”, “cell”, “cellular process”, etc. shown in Supplementary Figure 13). It will be helpful to show the readers more enriched terms to help distinguish the difference among similar states. Can the authors make a barplot or word cloud for top N enriched GO terms in each state instead? For example, bivalent promoters are known to regulate during development, so it is good to see development-related GO terms enriched in one or more bivalent states, or different bivalent states enriched for the development of different organs, etc.*

4. The paper provides many numbers for enrichment, but very often, it is unclear whether the enrichment has any biological significance. For example: a) Line 130-131: Acetylation is an informative histone modification. For those regions assigned as acetylation groups, do you have any biological explanation? What’s their role in gene regulation? *Well addressed.*

b) Line 138-139: “A DNase group consists of one state (DNase1) with strong emission of only DNase in all profiled cell types.” What’s the biological function of the DNase group since other active states are also expected to have high DNase signals? *Well addressed.*

c) In Fig 3A, Why are the TSS enrichments in PromF4 and PromF5 so high (62.2 and 31.9, respectively), even higher than BivProm1-4 (enrichment 8-28)? Would these two PromF states actually be a weak TSS state instead of flanking? Or are they actually located in gene-dense regions with many TSSs*? It makes sense now. It is interesting in biological terms to see different PromF states are enriched in different TSS-proximal distances, like PromF4, are most likely to be TSS downstream 200 ~ 400bp, and PromF6 are most likely to be TSS downstream 600bp ~1200bp as shown in Supp. Fig. 12. I think it is a great point to mention in the text as well.*

d) Line 228-229: “Acet1, a state associated with various acetylation marks and H3K9me3 had a 23-fold enrichment for simple repeats (Supp. Fig. 20).” What is the biological implication of this enrichment? *Well addressed. It is good to have this (CA)n and (TG)n enrichment state.*

e) Line 231-232: “States in the transcription start site group, TSS1-2 were most strongly enriched with tRNA and low complexity repeat class 232 (~10-60 fold) (Supp. Fig. 20).” What does this mean biologically? *Well addressed. It is very helpful to see the G-, C-, and GC- enrichment for each state. Besides high GC content in TSS states, it is also good to see slightly higher GC in DNase I states, which might be related to the DNase sensitivity of CTCF binding.*

f) Line 233-237: “We also saw specific states associated with the largest repeat classes of the genome, SINEs, LINEs, and LTRs. SINE repeats were most enriched in state Tx5 (3.7 fold) (Fig. 5C), which had high emission of H3K36me3 (Fig. 2A-B, Supp. Fig. 4-5). LINEs and LTRs repeats were most enriched for states in the H3K9me3-associated heterochromatin group with LINE most enriched in HET3 (3.4 fold), while LTRs were most enriched in HET5 (4.7 fold)” What’s the biological explanation for these specific states enriched with specific repeats? Are there any published analyses that support these enrichments? *Well addressed*.

g) Line 271-278: “This is consistent with previous analysis using cell-type-specific annotations showing that ConsHMM state 100 was enriched in a ZNF gene-associated chromatin state [32]. Interestingly though, another full-stack state (ZNF2) that was even more strongly enriched for ZNF genes (68.6 folds), had 0.4 fold enrichment for ConsHMM state 100, and instead was most enriched with ConsHMM state 1 (Fig. 3A- B, Supp. Fig. 22). Therefore, the full-stack annotation helped distinguish two ZNF-gene associated states that are associated with distinct conservation states.” Indeed ZNF1 and ZNF2 differ in their enrichments with ConsHMM state 100, but what does this result mean biologically? Do ZNF1 and ZNF2 belong to different classes of ZNF proteins with different biology? Indeed, there is a big enrichment difference on ZNF genes between states znf1 and znf2 (20.8 vs. 68.6). *It might be that the state znf1 are those newly evolving ZNF genes (with lower phyloP scores as well ). Also, one minor comment here, it would be good to stick with either “state ZNF1” or “state znf1” in the text.*

h) Line 288-290: “Consistent with those previous results, using the full-stack model, we observed that of the 13 states that were in the top 10 maximally enriched states with either deletions or duplications (1.18 fold or greater), seven were in the heterochromatin group (HET1-2,4-7,9) and one was in the ZNF gene state (ZNF2)”. Why are these states enriched in deletions or duplications? Any biological explanation? Or do you mean these regions have annotation artifacts? *Well addressed.*

i) Line 329-331: “Some other scores, however, showed relatively weak enrichments or even depletions for these promoter- and TSS- associated states.” Why do these other scores have lower enrichment for promoters? *Well addressed. It is good that the authors also add more detailed descriptions about the relationship of other variant prioritization scores and enriched states. For the following sentence in the newly added text (Page 19, line 60) “FATHMM had the greatest relative enrichment for the primary DNase state associated with CTCF cell type-specific chromatin states (DNaseI)”, is the “CTCF cell type-specific chromatin states” a typo?*

j) Line 335-344: “Enhancer states EnhA2-3,17 were among the states in the top five most enriched for FATHMM, GERP++, LINSIGHT, PhastCons, and PhyloP prioritized non-coding variants. These states also showed consistent enrichments with variants prioritized by Eigen, funSeq2, CADD, and REMM, though those scores showed even stronger rela tive enrichments for promoter states. In contrast, FIRE, DANN and CDTS were depleted for prioritized variants in all these enhancer states, and Eigen_PC showed both enrichments and depletions (Fig. 6C). FIRE and fitCons showed strong enrichment for exon states (TxEx1-3), which are associated with coding regions, even though coding bases were excluded in this analysis (Fig. 6C). FATHMM had the greatest relative enrichment for the primary DNase state associated with CTCF cell type-specific chromatin states (DNase1) (~10 fold), and was the only score for which this state was among the top five most enriched states (Fig. 6C, Supp. Fig. 28).” What do these different enrichment biases mean? Do these means promoter/enhancer/exon are with different conservation patterns biologically or some technically score bias? *Well addressed.*

k) Line 370-371: “TSS and promoters associated states, PromF4 and TSS1- 2, were maximally enriched for variants in the lowest range of MAF (0 < MAF <= 0.0001), 1.5-1.7 fold.” Why would these highly functional states be enriched in rare variants? Shouldn’t these states be depleted in rare variants? As shown in Fig. 3A, these states are highly conserved evolutionarily and, as a corollary, one would think they should be depleted of rare variants. *For the enrichment of CG sites in each state, what’s the difference between Panel B in Supplementary Figure 42 and Supplementary Figure 28?*

l) Line 424-445 and FigS39: why are the H3K9me3 states and GapArtf states so enriched in cancer-associated variants, much higher than enhancer/promoter states? Any biological reasons? One would think that states that are of regulatory functions (e.g., enhancers and promoters) should be enriched in cancer-associated variants. *Well addressed. A follow-up question: in the newly added DNAme level in each state (Supplementary Figure 15), why are the DNAme levels of most st ates in IMR-90 lower than all other cell lines (states such as Quiescent and Het by eyes). Is there any IMR-90 specific biological reason or because of data quality?*

5. The comparison of full-stack and concatenated models on predicting external genomic annotations is like attacking a strawman. For example, in predicting annotated exons (Fig. 4A), the full-stack annotation would obviously do better than annotations in individual samples since only a subset of genes is expressed in a particular sample. But what does this prove? It is hardly a useful application to predict exons using chromatin states since the former is better annotated than the latter. I suggest the authors tone down the expectation in this section and keep Figure 4 in Supplementary Materials. *Well addressed*.

6. The authors performed all the segmentation in hg19 and then lifted-over chromatin annotations in each 200-bp bin to GRCh38. Currently, almost all annotations are done on GRCh38. In order for the full-stack annotations to be a more useful resource, can the authors also run the whole pipeline in GRCh38 from reads alignment to segmentation? Please also provide a comparison between the state annotations in hg19 and GRCh38 to show whether the full-stack modeling is sensitive to a change in the version of the reference genome. *The authors have their point for the segment in hg19, and I agree it will be a ton of work to reprocess. For the scope of this work, it is fine to have everything as it is now.*

Response to Minor comments: *All comments here are well addressed.*

**Authors’ response to reviewers**

Reviewer report
Reviewer #1:
The authors have put in significant effort to address the concerns of both reviewers,
which is appreciated.

With the exception of a few relatively minor items, the authors have addressed my
concerns to my satisfaction.

Below, I briefly comment on each response of the authors point-by-point.

RESPONSE: We thank the reviewer for the positive comment. We also appreciate the
reviewer’s comments and suggestions that strengthened the manuscript. We

addressed the minor items as described below.
1. Stacked model learning
OK. Thanks for clarifying and commenting on the sampling approach.
RESPONSE: We thank the reviewer for this comment.
2. Terminology of non-stacked models
I appreciate the line of reasoning and explanation provided by the authors, however I
want to push on this a bit more since the use of "cell-type-specific" does not resolve my
concerns.
The term 'cell-type-specific' introduces multiple levels of ambiguity, namely:
- the meaning of "cell type", which the authors mention also includes "tissue type", and
likely
"biological condition", "cellular state", "biosample", etcetera
- the meaning of "specific", suggesting these annotations are "exclusive" to a given
cellular context, which is hardly ever the case.
Instead, I suggest using a simple but concrete term for each type of model /and/
annotation, namely:
"independent", "concatenated", and "stacked".
The latter two immediately suggest these are based on multiple biosamples.
Having three clear single-word terms to refer to both models and associated
annotations will
dramatically increase the readability of the manuscript.
RESPONSE: We thank the reviewer for this comment. We agree with the reviewer that
using the terms ‘concatenated’, ‘independent’ will increase the readability of the
manuscript. Therefore, we made changes to the manuscript by using these
terminologies instead where it is possible. We also note that in our previously
submitted revised manuscript, instead of using ‘cell-type-specific’, we used the terms
‘concatenated per-cell-type’ and ‘independent per-cell-type’ to refer to the annotations
that were generated using the concatenated and independent modeling approaches. In
this revised manuscript, we still opted to use ‘per-cell-type’ annotations where we want
to highlight the distinctions between the full-stack annotation and the other two types of
annotations (concatenated and independent), notably in the Background section,
before we introduce the concatenated and independent modeling approach.
3. Benefits of stacked models
Unfortunately, the explanation concerning "a systematic and global view of the data"
provided by the authors does not fully clarify things for me.
Would clustering based on all states provide a "systematic and global view of the
data"?
Can the authors be more explicit on what "unbiased", "systematic" and "global" mean
in this context?
RESPONSE: We thank the reviewer for this question. The stacked modelling allows us
to learn the genome-wide patterns of all the datasets and to annotate the genome all at
once, hence allowing us to get a ‘global’ view of datasets and the genome. At the same
time, since the full-stack states are defined based on all datasets, and not by solely
looking at characteristics associated with one known element (such as enhancer,
promoter), we can ensure that the full-stack annotation is ‘unbiased’ in characterizing
the genome through only one type of genomic element or cell type. Lastly, by virtue of
the model learning states in an unsupervised way with data from all cell types, we can
obtain a model where states are ‘systematically’ learned based on the frequently
observed epigenetic signals patterns across the genome in all cell types. In contrast,
for example, we referred to the study of (Ernst et al., 2011) to show that the full-stack
annotation can provide complementary benefits of “a more systematic and global view
of the data”. In (Ernst et al., 2011), 200-bp bins in the genome are selected for an
analysis if at least one reference epigenome is assigned to a particular chromatin
state, e.g. a strong active promoter state. Then, k-means clustering is used to classify
these genomic regions into clusters, based on the cell types that are assigned to the
selected state at each 200-bp bin. This approach can only allow one to investigate the
cell-type-specific patterns of one chromatin state at a time.

We also note that in our previously revised manuscript, based on the reviewers
comment we had opted to no longer describe the full-stack model as providing an
‘unbiased, systematic and global’ view of the data given the ambiguous nature of this
wording.
Thank you for clarifying some of the benefits of stacked models relative to more
conventional approaches, and confirming the LMNB1 vs. fibroblast suspicion and
incorporating this finding into the manuscript. I appreciate the changes made to the text
and supplementary figures.
RESPONSE: We thank the reviewer for this positive comment.
4. Stacked vs. concatenated models for characterizing dynamic state behavior
I'd like to note that the "x out of n samples are in state i" example was merely that: an
example.
Naturally there are more sophisticated ways to undertake such analyses, which could
also account for imbalanced cell type representations, state similarities, etcetera.
That said, with the changes made to the manuscript the authors now make a more
convincing case for using a stacked model.
RESPONSE: We thank the reviewer for the positive comment and clarification. We
agree with the reviewer that there are ways to analyze the cell-type-specificity of
concatenated chromatin states where we can take into account the imbalanced
numbers of samples from different cell types, and the similarities between states. We
have edited a sentence to the discussion to make reference that there are other
potential ways for post-hoc analysis.
“We also note that post-hoc concatenated state annotations can also be used for
cross-cell type analyses, particularly on a per-state basis by analyzing the frequency of
a specific state across cell types or potentially in other ways.”
5. Choice of number of chromatin states
Thanks to the authors for addressing this point in detail. I was hoping to see more of a
general recommendation for future stacked model users to choose a reasonable
number of states for their purpose, but agree with the authors that this is often
dependent on the type of application.
I appreciate the addition of new Supplemental figures to illustrate the choice of number
of states.
Unfortunately the text in the new Supp. Figure 3B is not legible, in particular the x-axis.
The analysis presented in the new Supp. Figure 3C is a bit unfair: by virtue of
comparing models with different state numbers to a mode with 100 states, the 100-
state model will always look favorable. It does little in terms of saying anything about
the quality of the 100-state model;
rather, it merely says something about how similar other models are to a 100-state
model.
The analysis presented in the new Supp. Figure 3D is more interesting in my opinion.
Although limited to the H3K4me1 mark, this is a relevant mark to consider in this case.
It would benefit the figure and interpretation if the authors would indicate below each
column how often its values were row-wise maximal.
RESPONSE: We thank the reviewer for this comment. We have modified the figure to
give more space for panel B, and modified panel D to include one more column
showing the row-wise maximum correlation, shown below. We find that the analysis of
Additional File 1: Fig S3C can be effective at establishing biologically motivated lower
bounds since one can potentially identify a biologically important state in a larger
model, but this state may not be adequately captured by a model with fewer states. For
example, this analysis shows that state EnhA20, a HUVEC active enhancer state, is
not captured in models with fewer than 100 states. We acknowledge that this does not
necessarily establish upper bounds since it does not guarantee a new state of interest
will not emerge from a model with an even greater number of states. We have updated
the legend for this figure to clarify on this point, which now reads:

Additional File 1 Figure S3: Evaluation of full-stack model’s number of states. (A)
AUCs of full-stack models with varying number of states in recovering external
genomic annotations. The figure shows the AUC of full-stack models with 20, 40, 60,
80, 100, and 120 states at predicting the genomic locations of multiple different
external genomic annotations (CpG Islands, lamina associated domains (laminB1lads),
Exon, Gene body, TES, TSS, and TSS2kb regions) (Methods). As the number of
chromatin states increases, the AUC increases, but the level of the AUC increases
diminishes. (B) The estimated AIC-BIC curves for models with the number of states
ranging from 10 to 120 (5 states apart). We calculated the AIC and BIC based on
ChromHMM’s output reporting the log-likelihood of observed data for 300 1-Mb
regions. Neg2_log_llh: -2 * negative log likelihood of observed data. (C) Maximum
correlations of emission parameters between each state in the 100-state model and
any state for each other model. This is output from ChromHMM’s CompareModels
command. Rows correspond to the states of the 100-state model. Columns correspond
to models with varying numbers of states. Values are the maximum correlation of any
state from the model in the column (with varying number of states) with the state from
the 100-state in the row. The 100-state model is boxed. This analysis can be effective
at establishing some biologically motivated lower bounds on the number of states. For
example, state EnhA20, a HUVEC specific enhancer state, is not captured in models
with fewer than 100 states. (D) Maximum correlations of emission parameters
associated with H3K4me1 (an enhancer mark available in all cell types) and the binary
vector indicating whether the cell type associated with an emission parameter is in a
tissue group (1) or not (0). The rows correspond to different tissue groups from
Roadmap Epigenomics Consortium (Kundaje et al., 2015). The columns correspond to
different models with varying numbers of states. The values show the maximum
correlations mentioned above across all states within a model. The 100-state model is
boxed. The last column shows the maximum correlations observed for each tissue type
(each row) across all the models.
6. The role of data imputation
I appreciate, and agree with, the comments made by the authors and would only
advise to include a brief remark on the technical concern of artificially high correlation
structures.
RESPONSE: We thank the reviewer for this suggestion. We now added a paragraph at
the end of the ‘Methods / Input data and processing’ section, which reads:
“We note that we chose not to use imputed data as input to the full-stack model. A
main reason is, as noted above, imputed data would still be based on observed data
from the 1032 datasets used for stacked modelling. Another reason is that imputed
data may have artificially high correlations across the cell types, which can particularly
be the case for marks that were experimentally mapped in few cell types. This could
potentially cause the stacked modeling to devote many states that correspond to
heavily correlated and less informative tracks of imputed data.”
7. Extendability to new datasets and establishing standard annotations
I agree with the authors' points, and would suggest that the authors more explicitly
argue that perhaps the stacked models may provide a more universal set of
annotations for use in e.g. genome browsers.
RESPONSE: We thank the reviewer for this comment. We appreciate the reviewer’s
suggestion to more explicitly state the use of the full-stack annotation. We added an
additional sentence on a paragraph in the discussion related to this to state:
“Overall, the full-stack model provides a universal annotation of the genome that can
be viewed as a single track in a genome browser or used for a variety of downstream
bioinformatic analyses.”
In conclusion:
The authors have put in significant effort to improve the manuscript in terms of
relevance for
Biological interpretation, and have addressed most of my (major and minor) concerns

to satisfaction.
The manuscript can still be improved in terms of readability and terminology, to which I
have made some suggestions above.
RESPONSE: We thank the reviewer for the positive comments and the additional
suggestions on how we can improve the readability and terminology, which we
addressed as described above.
Reviewer #2
The authors have addressed all the questions, significantly improving the manuscript. I
am in support of this manuscript being accepted for publication given that the minor
comments below are addressed.
RESPONSE: We thank the reviewer for the positive comments and the constructive
review. As described below, we have now addressed the minor comments.
Response to major comments: 1. It is the underlying assumption of ChromHMM that
each genomic bin can only be assigned to one chromatin state. When we consider the
concatenated model, for the genomic bins that are in one state (say, TSS, the gene is
expressed) in one sample but in another state in another sample (say, ReprPC, the
gene is repressed), and in a third state in yet another sample (say, Bivalent TSS, the
gene is poised for expression), what would this genomic bin be assigned to in the full-
stack model? Can the authors perform a detailed analysis on this subset of genomic
bins so that the reader can get a better understanding of the full-stack model? Well
addressed
RESPONSE: We thank the reviewer for the positive comment.
2. In Fig1, the authors showed two loci of full-stack modeling annotations. It is indeed
an advantage of the full-stack model to provide a big-picture view of the epigenomes,
as illustrated by these two loci. However, there are many genomic regions with
frequent state transitions as shown by the concatenated approach; can the authors
provide two examples of such regions and illustrate their state assignment in the full-
stack model? Well addressed. It is great to have these examples.
RESPONSE: We thank the reviewer for the positive comment.
3. Are different TSS states (TSS1-2, BivProm1-4) in the full-stack model associated
with different classes of genes? Can the author perform GO enrichment for each
subset of genes? Thank you for providing the GO enrichment results. However, the top
enriched GO terms in each state are likely to be paternal GO nodes, which didn’t show
biological significance (as “biological regulation”, “cell”, “cellular process”, etc. shown in
Supplementary Figure 13). It will be helpful to show the readers more enriched terms to
help distinguish the difference among similar states. Can the authors make a barplot or
word cloud for top N enriched GO terms in each state instead? For example, bivalent
promoters are known to regulate during development, so it is good to see
development-related GO terms enriched in one or more bivalent states, or different
bivalent states enriched for the development of different organs, etc.
RESPONSE: We thank the reviewer for the suggestion. We have now modified
Supplementary Figure 13 such that each subpanel shows the top 5 most significantly
enriched GO Biological Process and GO Molecular Function terms in each state
(displaying a total of 10 GO terms per state). Development-related GO terms were
observed in the top 5 terms for 3 out of 4 states in the Bivalent Promoter group
(Additional File 1: Figure S13). We only include the plots corresponding to states in the
TSS, flanking promoter and bivalent promoter groups in Supplementary Figure 13. The
plots associated with all states are provided in Additional File 2. The updated Additional
File 1: Figure S13 legend is shown below:
Figure S13: Top GO terms for states in promoter-associated states. Each subpanel
corresponds to a full-stack state (state names are shown in the plot title). In each
subpanel, the top 5 most significantly enriched GO Biological Process and GO
Molecular Function Terms are shown on the y-axis (showing a total of 10 GO terms).

The length of horizontal bars show the negative log10 (p value) of the GO enrichment,
based on the Binomial Test and outputted by GREAT (McLean et al., 2010). The
equivalent plots for all full-stack states are available in Additional File 2.
4. The paper provides many numbers for enrichment, but very often, it is unclear
whether the enrichment has any biological significance. For example: a) Line 130-131:
Acetylation is an informative histone modification. For those regions assigned as
acetylation groups, do you have any biological explanation? What’s their role in gene
regulation? Well addressed.
RESPONSE: We thank the reviewer for the positive comment.
b) Line 138-139: “A DNase group consists of one state (DNase1) with strong emission
of only DNase in all profiled cell types.” What’s the biological function of the DNase
group since other active states are also expected to have high DNase signals? Well
addressed.
RESPONSE: We thank the reviewer for the positive comment.
c) In Fig 3A, Why are the TSS enrichments in PromF4 and PromF5 so high (62.2 and
31.9, respectively), even higher than BivProm1-4 (enrichment 8-28)? Would these two
PromF states actually be a weak TSS state instead of flanking? Or are they actually
located in gene-dense regions with many TSSs? It makes sense now. It is interesting
in biological terms to see different PromF states are enriched in different TSS-proximal
distances, like PromF4, are most likely to be TSS downstream 200 ~ 400bp, and
PromF6 are most likely to be TSS downstream 600bp ~1200bp as shown in Supp. Fig.
12. I think it is a great point to mention in the text as well.
RESPONSE: We thank the reviewer for the positive comment. We have now modified
the sentences mentioning the neighborhood enrichments of flanking promoter states
around TSS to state:
“States in the flanking promoter group (PromF1-7) showed 6.5-28 fold enrichment for
being within 2kb of annotated TSS, with distinctions among states in terms of their
relative distance from the TSS (Fig. 3A, C, Additional File 1: Fig.S12A). Genes whose
TSS regions overlapped these states had higher average gene expression across
different cell types (Fig. 3A, C, Additional File 1: Fig. S10-11).”
d) Line 228-229: “Acet1, a state associated with various acetylation marks and
H3K9me3 had a 23-fold enrichment for simple repeats (Supp. Fig. 20).” What is the
biological implication of this enrichment? Well addressed. It is good to have this (CA)n
and (TG)n enrichment state.
RESPONSE: We thank the reviewer for the positive comment.
e) Line 231-232: “States in the transcription start site group, TSS1-2 were most
strongly enriched with tRNA and low complexity repeat class 232 (~10-60 fold) (Supp.
Fig. 20).” What does this mean biologically? Well addressed. It is very helpful to see
the G-, C-, and GC- enrichment for each state. Besides high GC content in TSS states,
it is also good to see slightly higher GC in DNase I states, which might be related to the
DNase sensitivity of CTCF binding.
RESPONSE: We thank the reviewer for the positive comment.
f) Line 233-237: “We also saw specific states associated with the largest repeat
classes of the genome, SINEs, LINEs, and LTRs. SINE repeats were most enriched in
state Tx5 (3.7 fold) (Fig. 5C), which had high emission of H3K36me3 (Fig. 2A-B, Supp.
Fig. 4-5). LINEs and LTRs repeats were most enriched for states in the H3K9me3-
associated heterochromatin group with LINE most enriched in HET3 (3.4 fold), while
LTRs were most enriched in HET5 (4.7 fold)” What’s the biological explanation for
these specific states enriched with specific repeats? Are there any published analyses
that support these enrichments? Well addressed.
RESPONSE: We thank the reviewer for the positive comments.

g) Line 271-278: “This is consistent with previous analysis using cell-type-specific
annotations showing that ConsHMM state 100 was enriched in a ZNF gene-associated
chromatin state [32]. Interestingly though, another full-stack state (ZNF2) that was even
more strongly enriched for ZNF genes (68.6 folds), had 0.4 fold enrichment for
ConsHMM state 100, and instead was most enriched with ConsHMM state 1 (Fig. 3A-
B, Supp. Fig. 22). Therefore, the full-stack annotation helped distinguish two ZNF-gene
associated states that are associated with distinct conservation states.” Indeed ZNF1
and ZNF2 differ in their enrichments with ConsHMM state 100, but what does this
result mean biologically? Do ZNF1 and ZNF2 belong to different classes of ZNF
proteins with different biology? Indeed, there is a big enrichment difference on ZNF
genes between states znf1 and znf2 (20.8 vs. 68.6). It might be that the state znf1 are
those newly evolving ZNF genes (with lower phyloP scores as well ). Also, one minor
comment here, it would be good to stick with either “state ZNF1” or “state znf1” in the
text.
RESPONSE: We thank the reviewer for this comment. We have now updated the state
names to be znf1 and znf2, consistently. We agree with the reviewer’s comment that
state znf1 may be associated with newly evolving ZNF genes and have added a
remark about this possibility.
Specifically, we modified the paragraph discussion these two states to now read:
“ConsHMM state 100, a conservation state associated with alignment artifacts, was
10.9 fold and 2.1 fold enriched for full-stack state znf2 and znf1, respectively (Fig. 3A-
B, Additional File 1: Fig. S30). This is consistent with previous analysis using
concatenated annotations showing that ConsHMM state 100 was most enriched in a
ZNF gene-associated chromatin state (Arneson & Ernst, 2019). State znf2 also showed
a 5.4-fold enrichment for ConsHMM state 1 which contrasts with state znf1, which
showed a 0.8 fold enrichment for ConsHMM state 1, suggesting a stronger association
of state znf1 with newly evolving ZNF genes or those under less constraint. This
difference is consistent with the znf2 state’s larger fold enrichment for coding exons
than znf1 (10.4 vs 1.1). The znf2 state also had a greater fold enrichment for ZNF
named genes in general (68.6 vs. 20.8 fold), with those enrichment stronger and the
difference greater when restricting to C2H2 annotated genes (86.8 vs. 25.1 fold) (Fig.
3A-B, Additional File 1: Fig.S51). Therefore, the full-stack annotation helped distinguish
two ZNF-gene associated states, which are associated with distinct conservation
states. As this example illustrates, the full-stack annotation captured conservation state
enrichments that were generally consistent with those seen in concatenated
annotations, but could also identify additional refined enrichment patterns.”
h) Line 288-290: “Consistent with those previous results, using the full-stack model, we
observed that of the 13 states that were in the top 10 maximally enriched states with
either deletions or duplications (1.18 fold or greater), seven were in the
heterochromatin group (HET1-2,4-7,9) and one was in the ZNF gene state (ZNF2)”.
Why are these states enriched in deletions or duplications? Any biological explanation?
Or do you mean these regions have annotation artifacts? Well addressed.
RESPONSE: We thank the reviewer for the positive comment.
i) Line 329-331: “Some other scores, however, showed relatively weak enrichments or
even depletions for these promoter- and TSS- associated states.” Why do these other
scores have lower enrichment for promoters? Well addressed. It is good that the
authors also add more detailed descriptions about the relationship of other variant
prioritization scores and enriched states. For the following sentence in the newly added
text (Page 19, line 60) “FATHMM had the greatest relative enrichment for the primary
DNase state associated with CTCF cell type-specific chromatin states (DNaseI)”, is the
“CTCF cell type-specific chromatin states” a typo?
RESPONSE: We thank the reviewer for pointing out this. We intended to say that
DNase1 is associated with CTCF states observed in multiple per-cell-type annotations
(Supp. Fig. 14), hence making DNase1 a full-stack state strongly associated with
CTCFs across cell types. We have revised the sentences to now read:
“FATHMM had the greatest relative enrichment (~10 fold) for the primary DNase state
(DNase1), which is associated with a CTCF state from a concatenated model defined

in 6 cell types (Hoffman et al., 2013), and was the only score for which this state was
among the top five most enriched states (Fig. 5C, Additional File 1: Fig. S14, 36).”
j) Line 335-344: “Enhancer states EnhA2-3,17 were among the states in the top five
most enriched for FATHMM, GERP++, LINSIGHT, PhastCons, and PhyloP prioritized
non-coding variants. These states also showed consistent enrichments with variants
prioritized by Eigen, funSeq2, CADD, and REMM, though those scores showed even
stronger rela tive enrichments for promoter states. In contrast, FIRE, DANN and CDTS
were depleted for prioritized variants in all these enhancer states, and Eigen_PC
showed both enrichments and depletions (Fig. 6C). FIRE and fitCons showed strong
enrichment for exon states (TxEx1-3), which are associated with coding regions, even
though coding bases were excluded in this analysis (Fig. 6C). FATHMM had the
greatest relative enrichment for the primary DNase state associated with CTCF cell
type-specific chromatin states (DNase1) (~10 fold), and was the only score for which
this state was among the top five most enriched states (Fig. 6C, Supp. Fig. 28).” What
do these different enrichment biases mean? Do these means promoter/enhancer/exon
are with different conservation patterns biologically or some technically score bias?
Well addressed.
RESPONSE: We thank the reviewer for the positive comments.
k) Line 370-371: “TSS and promoters associated states, PromF4 and TSS1- 2, were
maximally enriched for variants in the lowest range of MAF (0 < MAF <= 0.0001), 1.5-
1.7 fold.” Why would these highly functional states be enriched in rare variants?
Shouldn’t these states be depleted in rare variants? As shown in Fig. 3A, these states
are highly conserved evolutionarily and, as a corollary, one would think they should be
depleted of rare variants. For the enrichment of CG sites in each state, what’s the
difference between Panel B in Supplementary Figure 42 and Supplementary Figure
28?
RESPONSE: We thank the reviewer for the question. Supplementary Figure 28B
shows the enrichments of full-stack states with the GC content, i.e. genomic regions
that are rich with C, G, or both bases. This figure shows that states associated with
TSSs (TSS1-2) are enriched with low complexity repeats, which can be attributed to
the fact that these states are concurrently enriched with GC rich sequences.
Meanwhile, Supplementary Figure 42 shows the enrichment of full-stack states with
CpG dinucleotides (C followed by G), which has been associated with higher mutation
rates (cite). To make this distinction more explicit we have replaced CG dinucleotides
with CpG dinucleotides. To better clarify the distinctions between these two figures, we
have revised the legend of Additional File 1: Figure S42 to now read:
Figure S42: Full-stack states enrichments with variants from GNOMAD stratified by
minor allele frequencies, common variants (A) and CpG dinucleotides (B). In each
subpanel, each row corresponds to a full-stack state. The first column gives the state
labels, the second gives the percent of the genome that each state covers. The
heatmap colors are on a column specific coloring scale. The last row shows the
percentage of the genome that each group of variants occupy. (A) The last column
shows the enrichment of full-stack states with common variants from UCSC Genome
Browser’ snp151 track (Methods). Other columns show fold enrichments of full-stack
states for GNOMAD variants with the specified ranges of MAF, which are ordered in
increasing MAF (Karczewski et al., 2020). (B) The last column shows fold enrichment
of full-stack states with CpG dinucleotides. The three states showing highest
enrichment if variants of lowest MAF (0 < MAF <= 0.0001) (TSS1-2, PromF4) are also
the states most enriched states with CpG dinucleotide sites, likely reflecting the higher
mutation rates associated with CpG dinucleotide sites (Karczewski et al., 2020). We
note the distinction between this panel, which shows the enrichments of states with
CpG dinucleotide sites, and Fig. S28B, which highlights the relative higher enrichments
of TSS-associated states with regions that are rich with G, C or both nucleotides and
hence with low-complexity repeat class.
l) Line 424-445 and FigS39: why are the H3K9me3 states and GapArtf states so
enriched in cancer-associated variants, much higher than enhancer/promoter states?
Any biological reasons? One would think that states that are of regulatory functions
(e.g., enhancers and promoters) should be enriched in cancer-associated variants.

Well addressed. A follow-up question: in the newly added DNAme level in each state
(Supplementary Figure 15), why are the DNAme levels of most st ates in IMR-90 lower
than all other cell lines (states such as Quiescent and Het by eyes). Is there any IMR-
90 specific biological reason or because of data quality?
RESPONSE: We thank the reviewer for the positive comment and the follow-up
question. The IMR90 DNA Methylation datasets was one of two original WGBS
datasets collected (Lister et al., 2009) so a technical batch effect is a likely explanation
for the difference. It is, though, difficult to be certain from the available data. We added
a sentence to the legend of this figure to better clarify this point, which now reads:
Figure S15: Full-stack states’ average DNA methylation in different cell types. Each
row corresponds to one of the 100 full-stack states grouped into state groups as
indicated by the legend at the bottom. Each column corresponds to one cell type
whose DNA methylation data were available from Roadmap Epigenomics. The
columns are grouped based on their associated tissue group as indicated by the
legend at the bottom. Each column shows the average DNA methylation level in the
respective cell type that overlaps with each full-stack state (Methods). Among
promoters-associated states, those most enriched with CpG islands also show lowest
average DNA methylation levels (Fig. 3A), consistent with expectation (Jones & Takai,
2001; Weber et al., 2007). The lower DNAme levels of IMR90 compared to other cell
types might be related to a technical batch effect since it was one of two original
WGBS datasets collected (Lister et al., 2009).
5. The comparison of full-stack and concatenated models on predicting external
genomic annotations is like attacking a strawman. For example, in predicting annotated
exons (Fig. 4A), the full-stack annotation would obviously do better than annotations in
individual samples since only a subset of genes is expressed in a particular sample.
But what does this prove? It is hardly a useful application to predict exons using
chromatin states since the former is better annotated than the latter. I suggest the
authors tone down the expectation in this section and keep Figure 4 in Supplementary
Materials. Well addressed.
RESPONSE: We thank the reviewer for the positive comment.
6. The authors performed all the segmentation in hg19 and then lifted-over chromatin
annotations in each 200-bp bin to GRCh38. Currently, almost all annotations are done
on GRCh38. In order for the full-stack annotations to be a more useful resource, can
the authors also run the whole pipeline in GRCh38 from reads alignment to
segmentation? Please also provide a comparison between the state annotations in
hg19 and GRCh38 to show whether the full-stack modeling is sensitive to a change in
the version of the reference genome. The authors have their point for the segment in
hg19, and I agree it will be a ton of work to reprocess. For the scope of this work, it is
fine to have everything as it is now.
RESPONSE: We thank the reviewer for recognizing this.
Response to Minor comments: All comments here are well addressed.
RESPONSE: We thank the reviewer for the positive comment.

References
Arneson, A., & Ernst, J. (2019). Systematic discovery of conservation states for single-
nucleotide annotation of the human genome. Communications Biology, 2(1), 1–14.
Ernst, J., Kheradpour, P., Mikkelsen, T. S., Shoresh, N., Ward, L. D., Epstein, C. B.,
Zhang, X., Wang, L., Issner, R., & Coyne, M. (2011). Mapping and analysis of
chromatin state dynamics in nine human cell types. Nature, 473(7345), 43–49.
Hoffman, M. M., Ernst, J., Wilder, S. P., Kundaje, A., Harris, R. S., Libbrecht, M.,
Giardine, B., Ellenbogen, P. M., Bilmes, J. A., & Birney, E. (2013). Integrative
annotation of chromatin elements from ENCODE data. Nucleic Acids Research, 41(2),
827–841.
Jones, P. A., & Takai, D. (2001). The role of DNA methylation in mammalian
epigenetics. Science, 293(5532), 1068–1070.

Karczewski, K. J., Francioli, L. C., Tiao, G., Cummings, B. B., Alföldi, J., Wang, Q.,
Collins, R. L., Laricchia, K. M., Ganna, A., & Birnbaum, D. P. (2020). The mutational
constraint spectrum quantified from variation in 141,456 humans. Nature, 581(7809),
434–443.
Kundaje, A., Meuleman, W., Ernst, J., Bilenky, M., Yen, A., Heravi-Moussavi, A.,
Kheradpour, P., Zhang, Z., Wang, J., & Ziller, M. J. (2015). Integrative analysis of 111
reference human epigenomes. Nature, 518(7539), 317–330.
Lister, R., Pelizzola, M., Dowen, R. H., Hawkins, R. D., Hon, G., Tonti-Filippini, J.,
Nery, J. R., Lee, L., Ye, Z., & Ngo, Q.-M. (2009). Human DNA methylomes at base
resolution show widespread epigenomic differences. Nature, 462(7271), 315–322.
McLean, C. Y., Bristor, D., Hiller, M., Clarke, S. L., Schaar, B. T., Lowe, C. B., Wenger,
A. M., & Bejerano, G. (2010). GREAT improves functional interpretation of cis-
regulatory regions. Nature Biotechnology, 28(5), 495–501.
Weber, M., Hellmann, I., Stadler, M. B., Ramos, L., Pääbo, S., Rebhan, M., &
Schübeler, D. (2007). Distribution, silencing potential and evolutionary impact of
promoter DNA methylation in the human genome. Nature Genetics, 39(4), 457–466.
